# Quality of Cancer-Related Information on New Media (2014-2023): Systematic Review and Meta-Analysis

**DOI:** 10.2196/73185

**Published:** 2025-10-08

**Authors:** Xue-Jing Liu, Danny Valdez, Maria A Parker, Andi Mai, Eric R Walsh-Buhi

**Affiliations:** 1 Applied Health Science School of Public Health Indiana University Bloomington Bloomington, IN United States; 2 Epidemiology and Biostatistics School of Public Health Indiana University Bloomington Bloomington, IN United States

**Keywords:** social media, cancer, consumer health information, social communication, misinformation, health literacy, systematic review

## Abstract

**Background:**

New media have become vital sources of cancer-related health information. However, concerns about the quality of that information persist.

**Objective:**

This study aims to identify characteristics of studies considering cancer-related information on new media (including social media and artificial intelligence chatbots); analyze patterns in information quality across different platforms, cancer types, and evaluation tools; and synthesize the quality levels of the information.

**Methods:**

We systematically searched PubMed, Web of Science, Scopus, and Medline databases for peer-reviewed studies published in English between 2014 and 2023. The validity of the included studies was assessed based on risk of bias, reporting quality, and ethical approval, using the Joanna Briggs Institute Critical Appraisal and the STROBE (Strengthening the Reporting of Observational Studies in Epidemiology) checklists. Features of platforms, cancer types, evaluation tools, and trends were summarized. Ordinal logistic regression was used to estimate the associations between the conclusion of quality assessments and study features. A random-effects meta-analysis of proportions was conducted to synthesize the overall levels of information quality and corresponding 95% CIs for each assessment indicator.

**Results:**

A total of 75 studies were included, encompassing 297,519 posts related to 17 cancer types across 15 media platforms. Studies focusing on video-based media (odds ratio [OR] 0.02, 95% CI 0.01-0.12), rare cancers (OR 0.32, 95% CI 0.16-0.65), and combined cancer types (OR 0.04, 95% CI 0.01-0.14) were statistically less likely to yield higher quality conclusions compared to those on text-based media and common cancers. The pooled estimates reported moderate overall quality (DISCERN 43.58, 95% CI 37.80-49.35; Global Quality Score 49.91, 95% CI 43.31-56.50), moderate technical quality (Journal of American Medical Association Benchmark Criteria 46.13, 95% CI 38.87-53.39; Health on the Net Foundation Code of Conduct 49.68, 95% CI 19.68-79.68), moderate-high understandability (Patient Education Material Assessment Tool for Understandability 66.92, 95% CI 59.86-73.99), moderate-low actionability (Patient Education Materials Assessment Tool for Actionability 37.24, 95% CI 18.08-58.68; usefulness 48.86, 95% CI 26.24-71.48), and moderate-low completeness (34.22, 95% CI 27.96-40.48). Furthermore, 27.15% (95% CI 21.36-33.35) of posts contained misinformation, 21.15% (95% CI 8.96-36.50) contained harmful information, and 12.46% (95% CI 7.52-17.39) contained commercial bias. Publication bias was detected only in misinformation studies (Egger test: bias –5.67, 95% CI –9.63 to –1.71; *P*=.006), with high heterogeneity across most outcomes (*I*²>75%).

**Conclusions:**

Meta-analysis results revealed that the overall quality of cancer-related information on social media and artificial intelligence chatbots was moderate, with relatively higher scores for understandability but lower scores for actionability and completeness. A notable proportion of content contained misleading, harmful, or commercially biased information, posing potential risks to users. To support informed decision-making in cancer care, it is essential to improve the quality of information delivered through these media platforms.

**Trial Registration:**

PROSPERO CRD420251058032; https://www.crd.york.ac.uk/PROSPERO/view/CRD420251058032

## Introduction

### Background

Digital new media have become indispensable tools for health information and network support, notably among individuals experiencing chronic and terminal health conditions, such as many forms of cancer [1,2]. New media platforms, represented by social media and artificial intelligence (AI) chatbots, offer collaborative and participatory environments that serve as valuable resources for patients with cancers, survivors of cancer, and caregivers of patients [3,4]. These platforms facilitate the exchange of information [5], provide opportunities to seek external support [6], and share personal experiences of cancer [7]. The rise of AI chatbots has attracted considerable interest in their use for health information [8], with studies indicating that they can provide higher-quality information and more empathetic responses than physicians [9]. Further, among the US adults who report being on the internet almost constantly, specifically on social media [10], 72% report leveraging their preferred platform, and increasingly generative AI, to learn about health conditions diagnosed in themselves and their friends and family [11].

Research shows that turning to web-based outlets can improve psychosocial outcomes across various acute and chronic conditions [12]. Access to high-quality information further enhances health literacy, decision-making, health behaviors, outcomes, and health care experiences [13-17]. Conversely, low-quality information can promote trust in unproven, ineffective, and harmful treatments, as well as fake news, conspiracy theories, and misleading testimonials about “natural” or alternative cures [18]. Kington et al [19] described “high-quality information” as science-based and aligned with the best available evidence. Huang et al [20] further identified 15 key attributes of information quality, including accuracy, objectivity, accessibility, completeness, and clarity.

As cancer remains a leading cause of death globally, with rising incidence rates [21], related discussions are widespread on social media and AI chatbots [22,23], making the topic a target for commercial and political interests [24,25]. Substantial financial incentives drive the promotion of treatments [26], and patients, often desperate for hope, are particularly susceptible to misleading information circulated in digital spaces [18]. Limitations in models, databases, and algorithms pose challenges to the accuracy and credibility of the information provided by AI chatbots [27,28]. Survivors also face cognitive challenges such as fatigue, memory deficits, and reduced executive function [29,30], which may impair their ability to critically evaluate the information quality they received [31]. Consequently, low-quality information may lure patients into submitting to costly and harmful treatments, undermining the potential benefits of digital support networks [32].

To date, systematic, scoping, and other literature reviews generally report inconclusive and nondefinitive findings on the quality of cancer-related information on social media [22,33,34]. Most cross-sectional studies indicate considerable variability in information quality across platforms, content formats (ie, text-based or video-based), and cancer types [35]. This inconsistency may stem from differences in cancer types studied, platforms analyzed, and quality assessment approaches used. A critical gap remains the breadth of literature on this topic that directly accounts for (1) a wide variety of cancer types, (2) a multitude of platforms, and (3) differences in findings over time. Social media, in particular, has evolved considerably in the past two decades, giving rise to AI chatbots that now represent a new source of health information. Accounting for factors such as users’ shift in medium preference (text-based vs video-based), and platform changes (Twitter to X, and at the time of writing, the potential closure of TikTok) may reveal temporal trends that implicate improvement or worsening of information quality over time.

### Purpose

This paper presents a systematic review of empirical studies published between 2014 and 2023 that address cancer-related information on new media. It explores the growing reliance on these platforms for cancer health information, trends in platform use, and myriad tools used in the included studies to determine the information quality. Specifically, we addressed the following research questions (RQs): (RQ1) What are the key characteristics of studies evaluating the quality of cancer-related information on social media and AI chatbots, and how have these characteristics evolved? (RQ2) What factors influence the conclusion of quality assessments, and how do they vary across platforms, assessment tools, and cancer types? (RQ3) What patterns emerge in the assessment findings of new media cancer-related information quality?

Findings from this review contribute to further supporting our understanding of new media’s role as a source of cancer-related health information. By adopting a longitudinal perspective, the study identifies trends in quality assessment studies using data from social media and AI chatbots and offers evidence-based recommendations for future research on this vital topic.

## Methods

### Guidelines and Ethical Considerations

This review was reported according to the PRISMA (Preferred Reporting Items for Systematic Reviews and Meta-Analyses) guidelines (Multimedia Appendix 1). Our protocol was retrospectively registered on PROSPERO (CRD420251058032) on May 23, 2025.

As a review study of published studies, our research is considered nonhuman participant research and is exempt from review by an Institutional Review Board.

### Search Strategy

Between January and September 2024, we conducted a systematic literature search on four electronic databases (PubMed, Web of Science, Scopus, and Medline) for studies. Full strategies for the databases are available in Table S1 in Multimedia Appendix 2. We only included studies that underwent peer review and publication between 2014 and 2023 and presented empirical qualitative, quantitative, or computational findings in English. We also reviewed the reference lists of included studies to identify any additional publications that met the inclusion criteria but were not retrieved in the original search. Three researchers conducted independent examinations of the selection criteria, search terms, and the records returned by the search. Disagreements were resolved by discussion among the three researchers until a consensus was reached.

### Selection Criteria

We initially included studies if they met three broad categories of inclusion: (1) they contained a synthesis of cancer-related new media data, (2) they used an information quality assessment to rate the quality of data along varying criteria, and (3) they were published in English between 2014 and 2023. Cancer types were defined according to the International Classification of Diseases for Oncology. Likewise, we considered three broad categories of new media for inclusion: (1) text and graph-based media, such as Facebook, Instagram, Pinterest, Reddit, Twitter (now X), WeChat, and Weibo; (2) video-based media such as YouTube, TikTok, and Xigua; and (3) generative AI-based media, such as ChatGPT, Chatsonic, Microsoft Bing AI, and Perplexity.

We excluded studies that did not present original findings, unpublished manuscripts from preprint repositories such as arXiv, conference abstracts, narratives, literature reviews, and editorials. Studies were also excluded if they discussed the quality of information but did not elaborate on how it was assessed, for example, without conducting a comprehensive and systematic data collection and analysis, without referring to any quality criteria.

### Assessments of Risk of Bias, Quality, and Certainty

Two researchers independently assessed the quality and risk of bias of the included studies using the Joanna Briggs Institute Critical Appraisal Checklist for Analytical Cross-Sectional Studies [36] and the STROBE (Strengthening the Reporting of Observational Studies in Epidemiology) statement checklist [37]. Studies evaluated by the Joanna Briggs Institute were considered to have a yes or no risk of bias or to have some concerns in study design, conduct, and analysis. The STROBE checklist assessed and illustrated the quality of reporting in key sections of studies. Additionally, we recorded whether each study reported an ethical approval process, including compliance with human and animal rights standards, informed consent procedures, and data privacy protections.

Two researchers independently rated the certainty of evidence of outcome using GRADE (Grading of Recommendations Assessment, Development and Evaluation) via the GRADEpro Guideline Development Tool online platform, including assessment domains of risk of bias, inconsistency, indirectness, imprecision, and publication bias.

Disagreements between the two raters were discussed until a consensus was reached.

### Data Extraction

We extracted the following data from the included studies: (1) authors, (2) year of publication, (3) journal name, (4) study design (ie, quantitative, qualitative, or computational), (5) sample size, (6) sample genders, (7) study location, (8) authors’ identities (individual or constitute, nonmedical professional or medical professional), (7) social media platform, (8) search strategies, (9) cancer types, (10) the language of the original data, (11) the profession of the raters, (12) contents of the posts, and (13) study results.

We extracted the information assessment tools used in the included studies, such as the DISCERN [38] tool, along with the outcomes measured by each. Despite the variation in tools, most studies assessed at least one of the following: (1) overall quality: a holistic assessment of the posts; (2) technical criteria: an assessment of the layout and functionality of the data; (3) readability and understandability: an assessment of reading and comprehension level; (4) accuracy and misinformation: an evaluation of scientific validity of the content; (5) completeness and coverage: the scope of topics; (6) actionability and usefulness: an assessment of the practical nature of the content; (7) harmfulness: whether the content can cause or promote harm, and (8) commercial bias: evidence of the direct influence of commercial interests. We categorized the 75 studies based on the tools and metrics used to evaluate the quality of information and draw comparisons across studies. Data were extracted by one researcher and independently verified by a second researcher. All analyses were performed using Stata/SE (version 18.0; StataCorp LLC) and RStudio (version 4.3; Posit PBC).

### Analysis

#### Descriptive Analysis

First, we illustrated several granular details for all studies about the search process, rating process, and review process. Second, we documented the frequencies and percentages of all mentioned media platforms, cancer types, and assessment tools for information quality, as well as changes in those patterns over the study period.

#### Statistical Analysis

For a single study reporting information quality for stratified groups, such as different posters (eg, individual or institute or with or without medical professional), content types (eg, prevention or treatment), or other specified categories, we calculated the pooled mean and pooled standard deviation. For a study that reported a median instead of a mean for a quality indicator, we estimated the mean and SD from the median [39].

We standardized quality indicators across different scales by mapping them onto a proportional rate system. For example, a score of 2 in a 1-5 scale system would be standardized as 40% in the proportional rating system. Then those quality scores are classified as follows: “low quality” for scores within 0%-30%, “median quality” for scores within 30%-70%, and “high quality” for scores within 70%-100%. For negative indicators (misinformation, harmfulness, and commercial bias), the coding system was the opposite. To categorize the conclusion of each study based on quality indicators used, we adopted the following criteria: The overall conclusion of information in a study was deemed “positive” if it reported more “high-quality” than “low-quality” results. It was considered “negative” if it reported more “low quality” results than “high quality” results. Studies reporting exclusively “medium quality” results, or equal numbers of “high” and “low quality” results, were classified as “neutral.”

We then used ordinal logistic regression to estimate the associations between study conclusions and media type and cancer type, as well as characteristics of studies based on the search, rating, and report processes [40]. Regression models were weighted by the natural log of each study’s sample size [41], since studies on some kinds of platforms, such as Twitter and Reddit, included larger sample sizes than others, such as YouTube. This analysis reported odds ratios (ORs), where estimates greater than 1 indicate a positive association between the predictor variables and higher quality conclusions.

#### Meta-Analysis

We used a meta-analysis of proportions to test the homogeneity of results obtained using the same quality assessment tool. Only results reported by three or more studies using the same tool were included in the analysis. Statistical heterogeneity was assessed via *I*^2^ and Cochran *Q* test values, where an *I*^2^ value of 25% represents low heterogeneity, 50% represents moderate heterogeneity, and 75% represents high heterogeneity. Analysis of proportions was pooled with a random-effect model with DerSimonian-Laird intervals when the test of heterogeneity was moderate or high [42]. For studies reporting indicators with 0% or 100% estimates, we used the Freeman-Tukey double arcsine transformation to stabilize the variance [43]. We calculated 95% CIs with an α level of .05 to estimate statistical significance.

Assessment of publication bias was done via an Egger test and a visual inspection of funnel plot asymmetry, respectively, for each indicator. Because publication bias was suspected, a sensitivity analysis was conducted using leave-one-out analysis and evaluate the influence of each study on the overall estimate.

## Results

### Overall Characteristics of Included Studies

**Figure 1:**
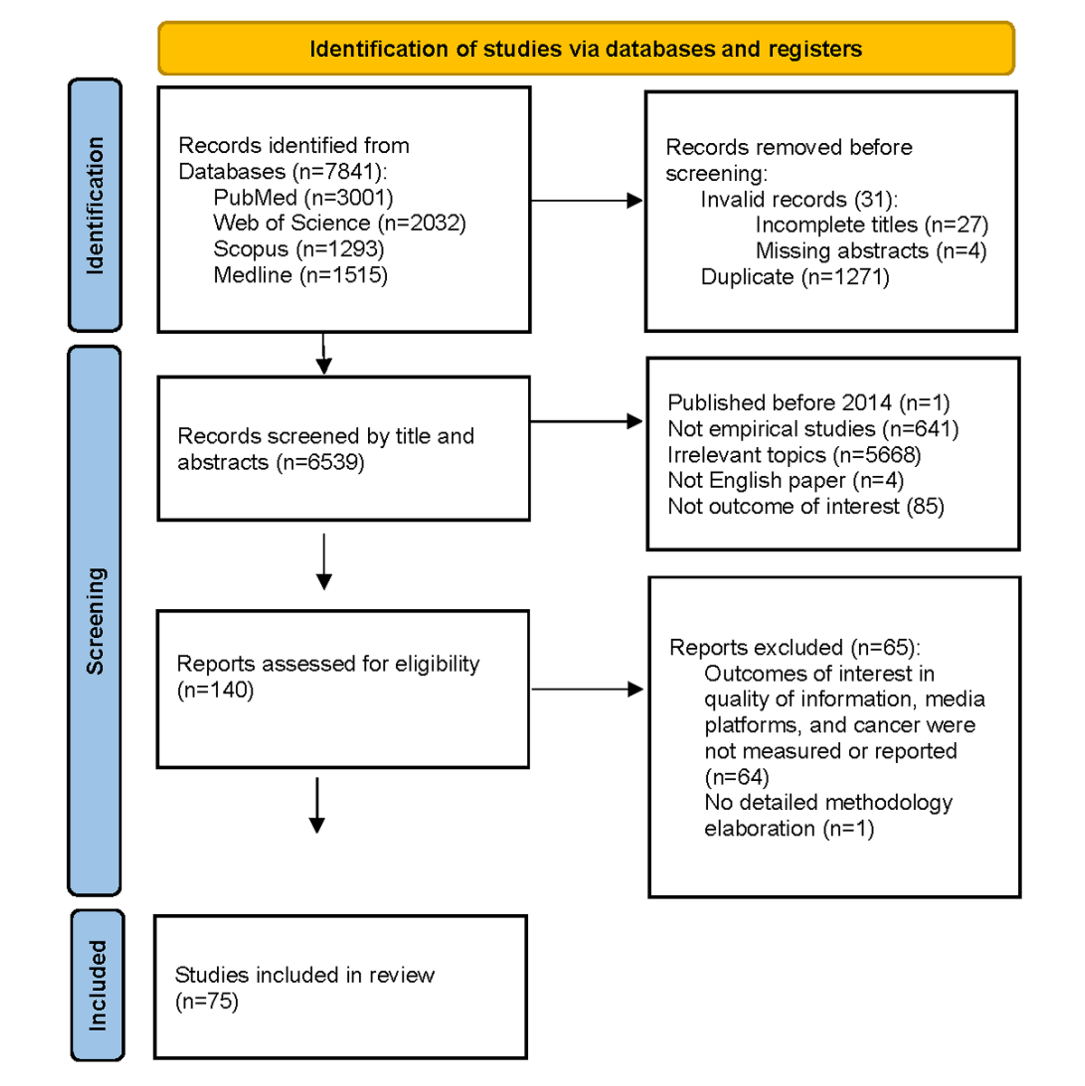
PRISMA (Preferred Reporting Items for Systematic Reviews and Meta-Analyses) flowchart.

This review summarizes the body of literature on the quality of cancer-related information on new media, published from 2014 to 2023. Our initial search identified 7841 studies, including (1) 3001 records from PubMed, (2) 2032 records from Web of Science, (3) 1293 records from Scopus, and (4) 1515 records from Medline. After screening papers by title and abstract, 140 potentially eligible papers remained for full-text review. Through full-text review, we excluded 65 additional studies because they lacked analytic rigor or did not include quality of information as a component of results. Our final sample size comprised 75 individual studies. Figure 1 depicts our filtering process according to the PRISMA guidelines.

We present our results below in relation to each of our RQs.

(RQ1) What are the key characteristics of studies evaluating the quality of cancer-related information on social media and AI chatbots, and how have these characteristics evolved?

Table 1 summarizes characteristics of included studies, including binary classifications of whether certain features were present or not. More than 80% of studies followed similar practices in the search process, including documenting their search period (70/75, 93%), search tool (69/75, 92%), query terms (73/75, 97%), and only considering one search tool (62/75, 83%), single platform (67/75, 89%). A total of 20 studies assessed content in languages other than English, including Chinese (n=6), German (n=5), Arabic (n=3), Japanese (n=2), French (n=2), and Spanish (n=2), and several other languages were examined in single studies, such as Korean, Italian, Turkish, Swedish, and Danish. During the rating process, 73 (97%) studies used a nonblinded rating process, 66 (88%) studies documented the number of raters, and 69 (92%) studies relied on more than one rater. However, across studies that reported content distributor information, 14 (19%) studies defined their rating criteria a priori, and 42 (56%) studies included a rater with a medical background. In the reporting process, more than 60% of studies reported “views,” “like,” or “unlike,” and “comment” behaviors, while a few tracked posts sharing behavior (11/75, 15%). About 70% of studies reported the posters’ identity, but few provided information on their demographics (17/75, 23%). Among the included studies, 67 (89%) reported the contents or topics discussed in the posts.

**Table 1:** Summary of characteristics across search, rating, and reporting processes of included studies (n=75).

Study characteristics		Yes, n (%)	No, n (%)	Other, n (%)
**Search process**				
	Date or period mentioned	70 (93)	5 (7)	N/A^a^
	Search tools mentioned	69 (92)	6 (8)	N/A
	More than one search tool used	13 (17)	62 (83)	N/A
	Search terms mentioned	73 (97)	2 (3)	N/A
	Initial hits reported	41 (55)	34 (45)	N/A
	Assessed language other than English	20 (27)	55 (73)	N/A
	More than one social media platform examined	8 (11)	67 (89)	N/A
	Cancer type more than one	30 (40)	45 (60)	N/A
**Rating process**				
	Raters blinded for the source	2 (3)	73 (97)	N/A
	Number of raters reported	66 (88)	9 (12)	N/A
	More than one rater	69 (92)	6 (8)	N/A
	Rater independently	38 (51)	37 (49)	N/A
	Interrater reliability figures for evaluation determined	34 (45)	41 (55)	N/A
	Process graph contained	34 (45)	41 (55)	N/A
	Medical professional background for rater	42 (56)	17 (23)	16 (21)^b^
	A priori criteria defined for quality	14 (19)	52 (69)	9 (12)^c^
**Reporting process**				
	Engagement: view	46 (61)	29 (39)	N/A
	Engagement: like or unlike	56 (75)	19 (25)	N/A
	Engagement: forward or share	11 (15)	64 (85)	N/A
	Engagement: comment	47 (63)	28 (37)	N/A
	Poster characteristics reported (gender, or age, or ethnicity, or country)	17 (23)	58 (77)	N/A
	Poster identity reported (personal, or institute, or medical professional)	53 (71)	22 (29)	N/A
	Contents or topics mentioned	67 (89)	8 (11)	N/A

^a^Not applicable.

^b^Raters are authors of the study, and it was unclear if they were medical professionals.

^c^Generally mentioned using literature or publication as criteria.

### Results of Risk of Bias, Quality, and Certainty Assessments

**Figure 2:**
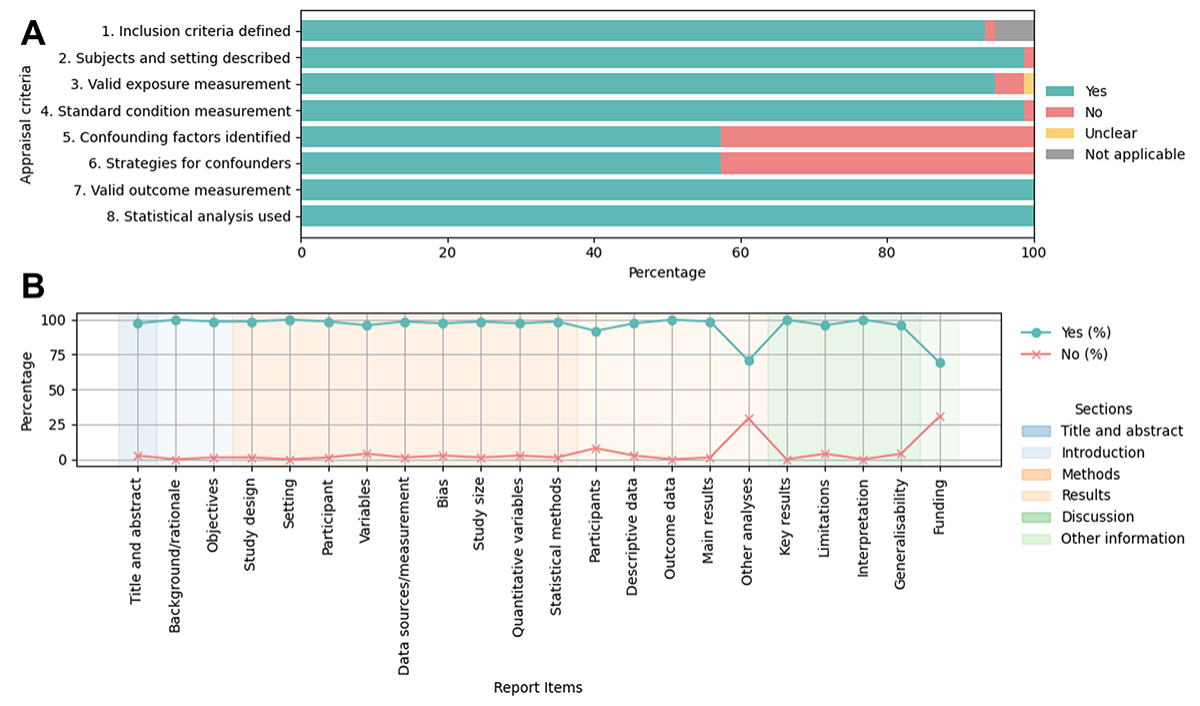
Distribution of risk of bias ratings and quality evaluation outcomes. (A) Risk of bias was assessed by the JBI Checklist. (B) Quality evaluation was assessed by the STROBE checklist. JBI: Joanna Briggs Institute; STROBE: Strengthening the Reporting of Observational Studies in Epidemiology.

A majority of studies demonstrated strong methodological quality, with 74 (98%) studies appropriately describing subjects and setting, 74 (98%) using standard condition measurement, all using valid outcome measurements, and using appropriate statistical analyses. However, we identified a considerable risk of bias in the handling of confounding factors; only 43 (57%) studies identified potential confounders and implemented strategies to address them (Figure 2A). Most studies reported key elements, while 22 (29%) studies did not include other analyses (eg, analysis of subgroup and interactions and sensitivity analysis), and 23 (31%) studies did not list funding sources (Figure 2B). Of the included studies, 34 (45%) reported ethical approval consideration, whereas 41 (55%) did not mention ethical approval. Among those that did report ethical approval, most stated that it was not required as the research only involved publicly available data, excluded human or animal participants, and removed identifiable information (eg, user IDs, links, and contact details), as is common with social media research. Only 3 studies obtained formal approval from research ethics committees [44-46]. Tables S2-S152 in Multimedia Appendix 2 [5,44-117] present details of the risk of bias and quality evaluation for individual studies.

Funnel plot and Egger test revealed significant asymmetry (bias coefficient –5.67, 95% CI –9.63 to –1.71; *P*=.006) only for studies evaluating misinformation, suggesting that studies could be missing in the literature that reported null or negative findings of misinformation or small study effects. No bias was detected for other indicators (Figures S1-S11 in Multimedia Appendix 2).

Since all included studies were observational in design, they were initially rated as low certainty according to the GRADE framework. Subsequently, the certainty of evidence for all evaluated outcomes was downgraded to very low due to one or more of the following reasons: high inconsistency (*I*²>75% in most outcomes), risk of indirectness (due to heterogeneous platforms or measurement definitions and tools), and, in some cases, imprecision (wide CIs and small sample sizes). These limitations significantly reduce confidence in the pooled estimates based on estimates. Details for each assessment and the reason for downgrading are in Table S153 in Multimedia Appendix 2.

### Individual Study Characteristics

#### Media Platforms

**Figure 3:**
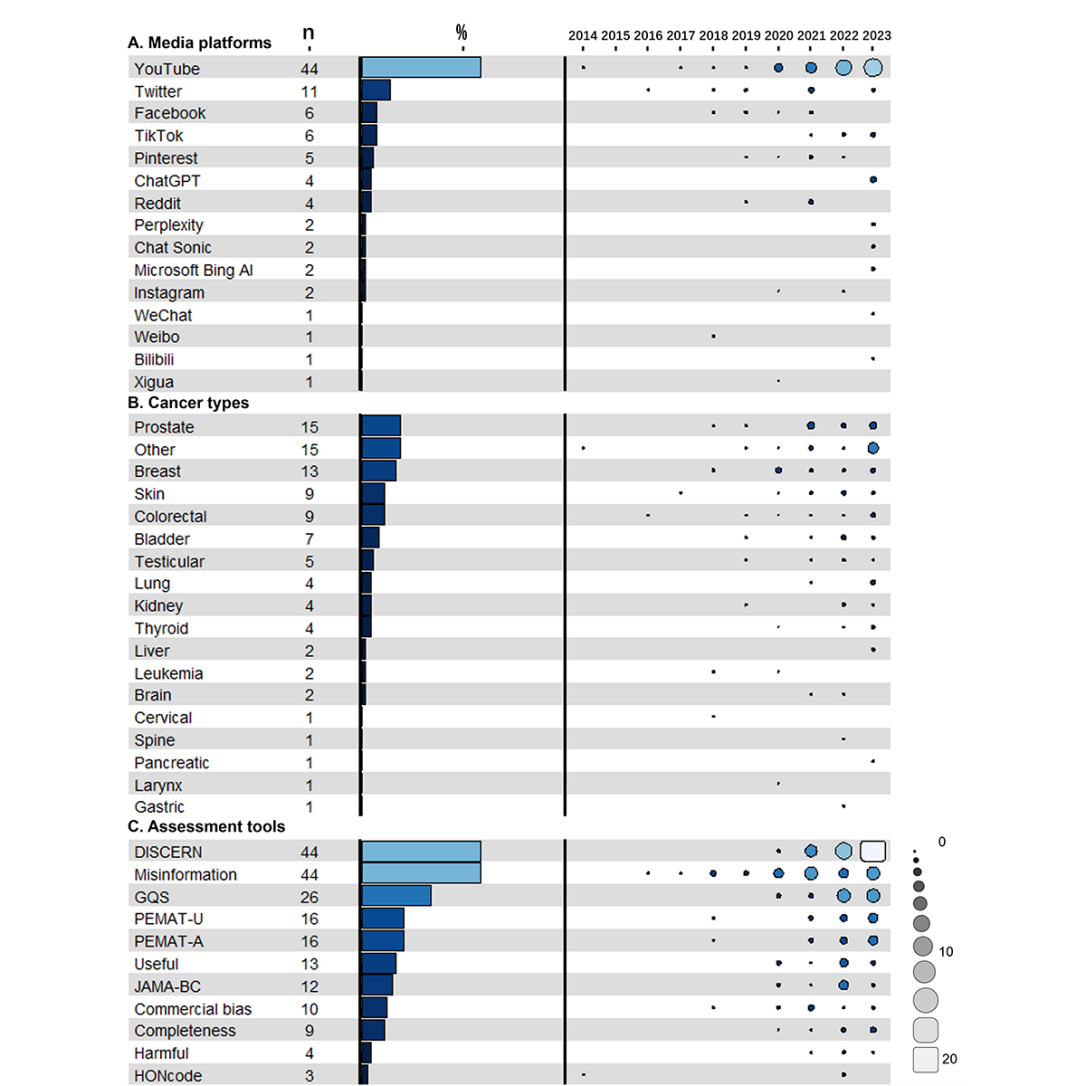
Frequencies and percentages of studies evaluating information quality across (A) media platforms, (B) cancer types, and (C) assessment tools (2014-2023). GQS: Global Quality Score; HONcode: Health on the Net Foundation Code of Conduct; JAMA-BC: Journal of the American Medical Association Benchmark Criteria; PEMAT-A: Patient Education Materials Assessment Tool for Actionability; PEMAT-U: Patient Education Materials Assessment Tool for Understandability.

Figure 3A illustrates the frequency of considered media platforms and changes in included platforms over time. A total of 20 studies investigated content from various text and graph platforms, including Twitter (n=11) [44,45,47-55], Facebook (n=6) [5,47-49,55,56], Pinterest (n=5) [47-49,57,58], Reddit (n=4) [47-49,59], and Instagram (n=2) [60,61], and 1 study each on WeChat [62] and Weibo [63]. A total of 51 records studied video-based media; of these, 44 examined YouTube [46,64-106], 6 focused on TikTok [107-112], and 1 study each on Bilibili [109] and Xigua [113]. Overall, 4 records studied data from generative AI-chatbot media, all of which specifically included ChatGPT [114-117]; 2 studies also included Perplexity, Chatsonic, and Bing AI [116,117].

We observed changes in the studied platforms over time. From 2014 to 2016, the number of included social media platforms was limited, with only 1 study investigating content from YouTube and 2 on Twitter. The scope expanded in 2018 and 2019, with increased research on other text- and graphic-based media, including 3 Facebook studies, 1 Pinterest study, 1 Reddit study, and 1 Weibo study. From 2020 to 2022, the number of studies increased, with 26 YouTube studies, while each of the other text- and graphic-based media platforms maintained approximately 3-4 publications. In 2023, this trend persisted, with video-based media, especially YouTube, continuing to be the primary focus of cancer information quality research, as evidenced by 14 studies. This period also marked the emergence of research on generative AI chatbot media, which was the subject of 4 studies.

#### Cancer Types

We captured 17 unique types of cancer within our data with varying levels of emphasis (Figure 3B). There were 15 research studied information about prostate cancer [47,49,58,61,75,81-83,88,91,110,111,114,116,117]; 13 about breast cancer; 9 about skin cancer [48,56,68,72,90,94,97,100,116]; 9 about colorectal cancer [47,53,54,66,67,73,105,114,116]; 7 about bladder cancer [49,58,84,85,111,114,117]; 5 about testicular cancer [46,49,58,93,117]; 4 each about lung cancer [47,71,95,116], thyroid cancer [86,96,107,108], and kidney cancer [49,111,114,117]; 2 each about liver cancer [78,109], leukemia [5,69], and brain cancer [87,101]; and 1 each about cervical [63], gastric [112], larynx [103], pancreatic [77], and spine [65] cancers. Another 15 studies were about cancer information in general or did not specify the cancer type [44,45,51,52,55,59,60,70,76,79,80,92,99,102,115]. Several studies were not limited to a single type of cancer but examined multiple types. Therefore, those studies were counted more than once.

#### Quality Assessment Tools

Figure 3C highlights the presence of formal and informal information quality tools used to evaluate data in a given study. We observed that a majority of studies used DISCERN (44/75, 59%) or evaluated misinformation (44/75, 59%), followed by Global Quality Score (GQS; n=26) and Patient Education Materials Assessment Tool (PEMAT; n=16). Tool use evolved over time. In 2014-2019, misinformation was the most common instrument, used in 10 studies. From 2020 to 2022, both misinformation and DISCERN saw increased use, each featuring in 24 studies. This trend persisted in 2023, with DISCERN remaining prominent in 20 studies published that year.

#### Content Distributors

Across 54 studies that reported content distributor information, medical professionals, such as surgeons, physicians, endocrinologists, sonographers, radiologists, and dietitians, were frequently identified as key contributors. Video-based studies consistently reported that the most-watched or -engaged videos were published by such medical professionals. Notably, they produced 80% of top TikTok videos on thyroid cancer [107,108] and 80% of leading YouTube posts on spine tumors [65] and hepatocellular carcinoma [78]. They also contributed over half of the top YouTube posts on rectal cancer surgery [66], nutrition [76], the mental health of patients with prostate cancer [81], radioactive iodine therapy [96], and larynx cancer [103], as well as on liver cancer [109] and gastric cancer [112] in Chinese on TikTok.

Institutional contributors, such as hospitals, clinics, academic centers, and universities, also played a role. For instance, they produced 92% of the top YouTube videos on pediatric cancer clinical trials [99] and 54% on Merkel cell carcinoma [100], though their contributions were lower across other topics.

In contrast, two studies of TikTok revealed that nonmedical individuals, including patients and their families or friends, accounted for over 93% of top videos on gastric cancer in English and Japanese [112] and 83% of prostate cancer videos [110]. Nonmedical contributors, such as media agencies, for-profit companies, herbalists, health websites, or lifestyle vloggers, contributed less than 61.1% of all topics.

#### Engagement Metrics

Studies analyzing video-based platforms frequently reported engagement metrics such as views, likes or dislikes, shares, and comments. Among 51 such studies, view counts ranged as low as 3 [83] to as high as 11 million [107], the latter for a thyroid cancer video posted by a medical professional on TikTok. YouTube videos on breast cancer and leukemia also reached high viewership, each exceeding 7 million views [69,73,106]. In contrast, videos on prostate cancer (TikTok) and pediatric cancer clinical trials (YouTube) averaged around 2000 views [99,110].

Likes and comments were lower than view counts, as has been noted previously. The most-liked video was a TikTok post on thyroid cancer, which garnered 308,000 likes [107,108], followed by a breast cancer video on YouTube with approximately 226,000 likes [106]. The most “disliked” video addressed herbal cancer treatments in Arabic, receiving an average of 994 dislikes on YouTube [70]. Comment activity varied widely. Thyroid cancer videos on TikTok averaged 1252 comments, with the most-commented video amassing over 73,000 comments [107,108]. Nutrition and lung cancer videos each had over 450 comments [92,95].

Of nonvideo, text-based entries, Facebook demonstrated the highest overall engagement, particularly in posts related to common cancers, including breast, prostate, colorectal, and lung cancers, as well as dermatological and genitourinary cancers [47-49].

### Factors Impacting Quality Conclusion

(RQ2) What factors influence the conclusion of quality assessments, and how do they vary across platforms, assessment tools, and cancer types?

The ordinal logistic regression analysis identified several factors associated with reporting higher quality conclusions (Table 2). Video-based media (OR 0.02, 95% CI 0.01-0.12), studies on rare cancers (OR 0.32, 95% CI 0.16-0.65), and studies on combined cancer types (OR 0.04, 95% CI 0.01-0.14) were less likely to yield high-quality conclusions than text-based media and studies on common cancers.

During the search process, studies using multiple search tools (OR 0.30, 95% CI 0.13-0.73), mentioning search terms (OR 0.13, 95% CI 0.02-0.81), reporting initial hits (OR 0.14, 95% CI 0.07-0.28), sourcing content in languages other than English (OR 0.35, 95% CI 0.16-0.76), and analyzing multiple media platforms (OR 0.06, 95% CI 0.02-0.27) were less likely to report higher quality conclusions.

During the rating process, studies disclosing the number of raters (OR 0.02, 95% CI 0.00-0.14) were less likely to report high-quality conclusions. In contrast, studies including process graphs (OR 3.06, 95% CI 1.61-5.79) and using literature-based assessments (OR 2.93, 95% CI 1.04-8.20) were more likely to report higher quality conclusions.

During the report process, studies reporting engagement metrics such as likes or dislikes (OR 3.35, 95% CI 1.20-9.38), forwards or shares (OR 6.17, 95% CI 1.61-23.65), and mentioning content or topics (OR 3.77, 95% CI 1.43-9.94), were associated with higher odds of reporting high-quality conclusions.

**Table 2:** Associations between study characteristics and quality of conclusions.

Characteristics of the study		OR^a^ (95% CI)	*P* value
**Media type (text-based media as reference)**			
	Video-based	0.02 (0.01-0.12)	<.001
	AI^b^-based	0.70 (0.10-5.01)	.72
**Cancer type^c^ (common cancer as reference)**			
	Rare cancer	0.32 (0.16-0.65)	.002
	Combined cancer	0.04 (0.01-0.14)	<.001
**Search process**			
	Date or period mentioned	1.75 (0.32-9.47)	.52
	Search tools mentioned	2.01 (0.46-8.89)	.36
	More than one search tool used	0.30 (0.13-0.73)	.008
	Search terms mentioned	0.13 (0.02-0.81)	.03
	Initial hits reported	0.14 (0.07-0.28)	<.001
	Assessed language other than English	0.35 (0.16-0.76)	.009
	Sites on more than one social media platform	0.06 (0.02-0.27)	<.001
	Cancer type more than one	1.41 (0.55-3.63)	.47
**Rating process**			
	Raters blinded for the source	11.16 (0.60-207.53)	.11
	Number of raters reported	0.02 (0.00-0.14)	<.001
	More than one rater	5.21 (0.76-35.75)	.09
	Rater working independently	0.88 (0.46-1.67)	.69
	Interrater reliability figures for evaluation determined	1.22 (0.62-2.43)	.56
	Process graph contained	3.06 (1.61-5.79)	<.001
**Medical professional background for rater (no professional as reference)**			
	Author as raters	0.43 (0.14-1.30)	.14
	Professional	0.52 (0.17-1.60)	.26
**A priori criteria defined for quality (no criteria mentioned as reference)**			
	Based on literature	2.93 (1.04-8.20)	.04
	Specific criteria mentioned	0.94 (0.41-2.14)	.88
**Reporting process**			
	Engagement: view	1.64 (0.40-6.70)	.49
	Engagement: like or dislike	3.35 (1.20-9.38)	.02
	Engagement: forward or share	6.17 (1.61-23.65)	.008
	Engagement: comment	1.97 (0.91-4.29)	.09
	Poster characteristics reported (gender, or age, or ethnicity, or country)	1.51 (0.63-3.64)	.36
	Poster identity reported (personal, or institute, or medical professional)	1.81 (0.77-4.28)	.18
	Contents or topics mentioned	3.77 (1.43-9.94)	.007

^a^OR: odds ratio.

^b^AI: artificial intelligence.

^c^Using GLOBOCAN 2020 statistics, we coded the top 10 most common cancers as “common cancer,” and others as “rare cancer” [118].

### Information Quality Criteria

(RQ3) What patterns emerge in the assessment findings of new media cancer-related information quality?

#### Overall Quality

Overall quality refers to a holistic assessment of content, considering its alignment with current scientific standards and whether content achieves educational aims. The most commonly used tools were DISCERN (44/75, 59%) and GQS (26/75, 35%). DISCERN is a 16-item tool evaluating publication reliability, quality of information on treatment choices, and a singular overall quality rating. GQS uses a 5-point Likert scale from 1=poor to 5=excellent.

Overall, content provided by medical individuals and institutions, such as hospitals, physicians, and dietitians, received higher DISCERN scores than that from nonprofessional sources [46,64,71,72,76,77,89,96,101,107,109,111-113]. Among these medical groups, hospitals provided higher-quality information than health organizations [107], and doctors specializing in modern medicine consistently scored higher than those in traditional medicine [109]. However, an exception was noted in one study on bladder cancer information on YouTube, where content from medical professionals scored lower [85]. Interestingly, TikTok videos by news agencies sometimes outperformed medical providers in quality, attributed to the absence of confusing jargon [111]. Comparisons between for-profit and nonprofit sources yielded mixed results: some studies reported higher DISCERN scores for for-profit sources [71,111], whereas others, particularly on colorectal cancer on YouTube, found no significant differences [66,67].

Studies using the DISCERN tool identified varying scores along different criteria. A total of 8 studies reported the highest scores for “explicit aims” [68,70,82,89,91,93,99,102], 6 for “aims achieved” [62,82,89,91,93,101], and 4 for “benefits of treatments” [70,91,93,99]. The most common reason for score deductions was the lack of “additional sources of information,” reported in 7 studies [62,68,72,82,91,93,101]. In total, 4 studies identified the lowest scores for failing to “describe what would happen if any treatment is not used” [82,89,91,101], and 3 studies noted deficiencies in “providing information source” [68,72,114]. Additionally, 2 studies each noted the lowest scores for reporting “currency of information” [70,93], “reference to areas of uncertainty” [87,89], “risks of treatment” [99,117], and “quality of life” [70,117].

DISCERN-based assessments also revealed regional and linguistic variations in content quality. A study of gastric cancer TikTok videos found that Chinese-language content was of higher quality than English and Japanese videos [112]. However, no significant quality differences were observed across prostate and thyroid cancer videos in English, French, German, Italian, and Turkish on YouTube [82,96]. Geographic comparisons of English-language videos showed that content from the United States consistently ranked higher in quality than that from other locations [89,101].

Three studies comparing AI-chatbot media on a range of platforms found that they generally provided moderate to high-quality information [114,116,117]. These chatbots frequently cited reputable sources such as the American Cancer Society and the Mayo Clinic.

Studies using GQS also found that quality was typically higher for videos produced by medical professionals [69,72,76,89,96,109,113] and for-profit medical providers [66,67]. For instance, YouTube content posted by medical providers on pediatric cancer clinical trials [99], liver cancer [78], and skin cancer [100] received GQS ratings of 4 or higher, indicating good quality. While YouTube videos on Merkel cell carcinoma [100], breast cancer originating from Australia [89], and breast cancer videos uploaded by medical advertisers on Xigua [113] received GQS scores below 2, indicating poor quality.

#### Technical Quality

Technical quality is the evaluation of disclosure ethics. The commonly used Journal of the American Medical Association Benchmark Criteria (JAMA-BC) critically assesses web content based on four key “transparency criteria”: authorship, attribution, disclosure, and currency. This tool, which has a high score of 4.0, was applied in five studies of YouTube content. The highest JAMA-BC score, 2.6, was reported for spine tumor videos [65], while the lowest score, 1.0, was for nutrition videos posted by independent users [76], indicating minimal adherence to reliability standards. Across included studies, content was rated high for “authorship” when clear information about contributors and their credentials was provided; the “disclosure” and “currency” indicators were rated lowest, reflecting a lack of transparency regarding sponsorship, commercial funding, potential conflicts of interest, and dates of posted and updated information [68,72,90].

Transparency of ethics for content was measured using other tools. Three studies used the Health on the Net Foundation Code of Conduct [119], which uses eight constructs: (1) authority, (2) complementarity, (3) privacy, (4) attribution, (5) justification, (6) contact details, (7) financial disclosure, and (8) advertising policy. Studies found that most cancer content videos disclosed authority but few disclosed source information, conflicts of interest, financial sources, or advertisement policy [89,102,111]. The Quality Evaluation Scoring Tool, used in one study, measures six aspects of web-based health information: (1) authorship, (2) attribution, (3) conflicts of interest, (4) currency, (5) complementarity, and (6) tone [120]. The study using the Quality Evaluation Scoring Tool examined TikTok videos on gastric cancer and found that Chinese-language videos scored higher than Japanese- and English-language videos [112]. Additionally, the Audiovisual Quality Score, which assesses the viewability, precision, and editing of audiovisual materials, revealed that larynx cancer videos from university sources showed clearer and more professional editing [103].

#### Readability and Understandability

Readability and understandability are metrics used to determine how effectively audiences can process information. PEMAT-U is the first part of the PEMAT toolkit to assess the understandability of print and audiovisual materials, which consists of 13 questions measuring the understandability of content’s language, organization, and visual design [121]. A total of 18 studies adapted PEMAT-U scores for digital content, with a majority reporting high understandability (above 70%). In general, higher scores were attributed to content with a clear purpose and use of accessible language [70,110]; and lower scores were attributed to content that lacked summaries or educational visual aids to help people understand the content [70,72,90,116,117]. The highest PEMAT-U score, 88%, was awarded to research on imaging information about prostate cancer on Instagram [61], followed by thyroid cancer videos from TikTok [108]. The lowest PEMAT-U scores (below 30%) were found in Arabic-language YouTube videos on herbal cancer treatments [70] and immunotherapy for renal cell cancer and prostate cancers [79]. Two studies using PEMAT-U found that content generated by AI chatbots often included medical jargon and concise terminology, making it difficult for lay audiences to understand [116,117].

Three studies measuring readability of content used the Flesch-Kincaid scale, which determines the average reading level needed to comprehend a written document on a continuum from 5 (signifying a fifth-grade reading level) to 16 (indicating a postgraduate reading level). One of these studies examined prostate cancer information on YouTube, reporting a 12th-grade readability level overall [75]. The other two focused on AI chatbots’ responses to cancer-related inquiries and found that the content was at a college-level readability [116,117].

#### Accuracy and Misinformation

Accuracy refers to the extent to which information aligns with established scientific or medical evidence. Terms such as misinformation, misleading content, false claims, and nonevidence-based claims are sometimes used to describe a lack of accuracy. Most studies did not use a standardized tool to evaluate accuracy. Rather, misinformation was typically assessed by identifying the proportion of content that deviated from scientific standards. Some studies applied predefined criteria, while others relied on expert reviewers to assess and classify content with subject experts.

One study analyzing the top 10 most-viewed YouTube videos on tetrahydrocannabinol oil and skin cancer concluded that all contained misinformation [94]. Similarly, a study of YouTube videos on prostate cancer flagged 76.25% as containing misinformation, with radiotherapy videos demonstrating less misinformation than surgery videos [91]. Over half of the videos on prostate cancer in Arabic [83], and in English on skin cancer [48], breast cancer [113], immunotherapy for urological tumors [79], and postsurgical exercise for breast cancer [104] were also identified to contain misinformation.

The misuse or discrediting of health services was the most common type of misinformation flagged by 17 studies, encompassing inappropriate use or dismissal of treatments [44,57,79,91,93,98,101,115], vaccines [51,52,56,59], screenings [49,67,98,105,110], and diagnostic tests [50,56,57,101]. For example, 25% of Facebook posts on acute lymphoblastic leukemia included disapproved treatment protocols and health services [5], and 16.5% of Pinterest pins undermined the accuracy and safety of mammograms, advocating instead for alternatives such as ultrasound or thermography, and spreading false claims about bioidentical hormones and breast tumors [57]. One study found that 12.5% of AI chatbot-generated cancer treatment responses contained hallucinated therapies, such as immunotherapy, which was not clinically recommended [115]. Additionally, 42% of Arabic-language videos on breast cancer called for inadequate screening and treatment protocols [98].

Another common type of misinformation mentioned in 15 studies involved unproven prevention and treatment modalities. For example, a study on Facebook pages about acute lymphoblastic leukemia found that all references to alternative and complementary therapies were related to unproven treatment modalities [5]. Similarly, 74.2% of alternative medicine content in dermatology-related posts was found to be misleading [48]. On Twitter, mentions of “alternative treatments” are often linked to external sources, such as hyperlinks, books, videos, or movies, without assessing the credibility of those materials [45]. Cannabis was frequently portrayed on Facebook and Twitter as an alternative cancer treatment, with 43.8% of such posts relying on anecdotal patient stories and over half using invalid scientific reasoning to support these claims [45,47,55]. One TikTok study found that about 5% of videos featured supernatural or heroic powers as potential cancer treatments [111]. Other content touting laetrile and colloidal silver on Twitter, despite their potential toxicity and lack of cancer-fighting benefits [45,57], as well as spiritual healing [45], acupuncture, chiropractic care, yoga [45], and escharotic black salve [56] as cancer treatments on social media.

Eight studies reported misinformation that overstated the effectiveness of certain foods and supplements in preventing or curing cancer. Information on social media falsely claimed some diet [45,50,56,95], various supplements [53,56,60] (eg, flaxseed, turmeric, IGF-1, vitamin D, slippery elm, probiotics, and coconut oil), specific fruits [54,57] (eg, pomegranates and mushroom), herbs [53,56,57] (eg, dandelions, curcumin, slippery elm, blood root, and gumby gumby), drinks [56,57,110] (eg, green tea, miracle beverage, and apple cider vinegar) as “natural remedies,” “cancer pills,” or “cures” for cancers.

#### Scope and Completeness

**Figure 4:**
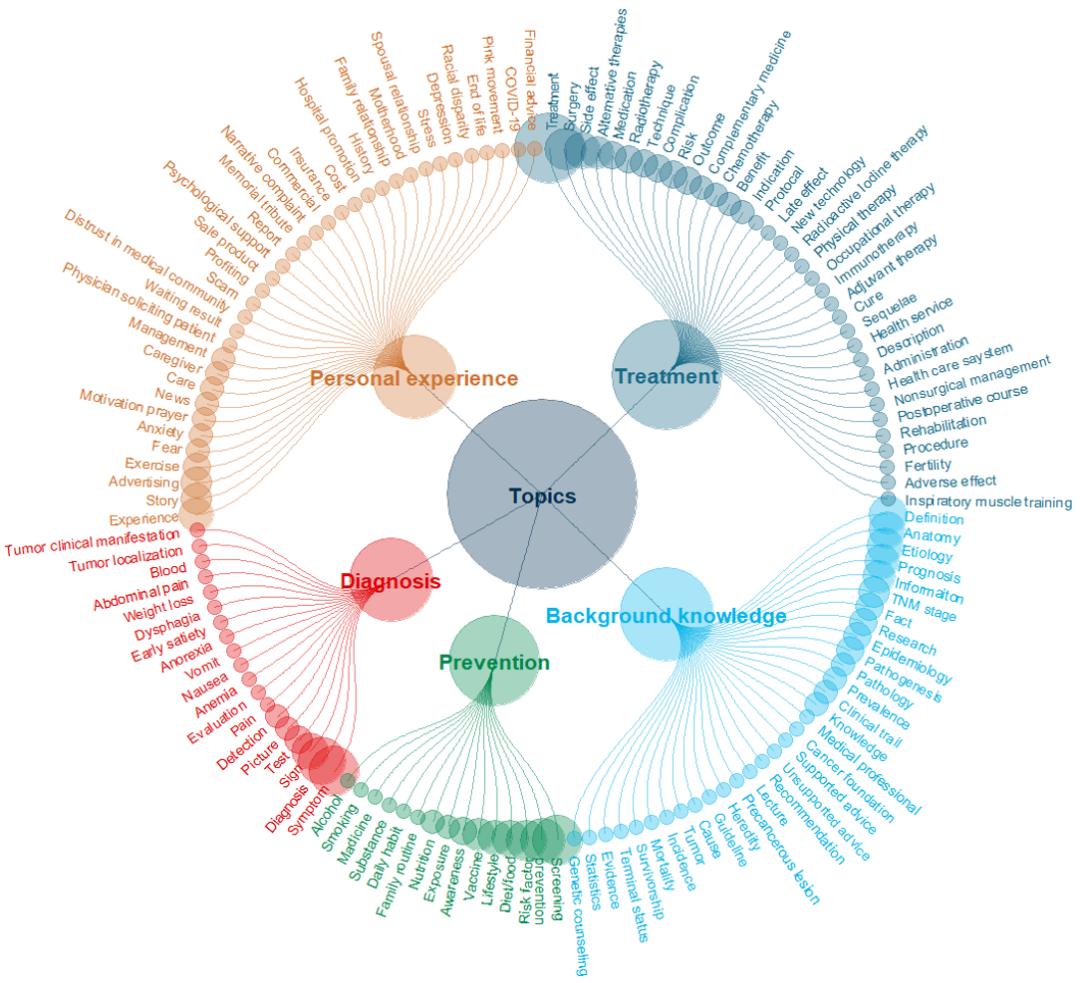
Overview of cancer-related topics examined across media platforms. Larger circles denote topics that were more frequently examined. The topics are not mutually exclusive and may overlap.

Scope refers to the range of topics covered. Figure 4 illustrates the topics identified across the included studies. The most frequently covered topic was treatment (50 studies), encompassing content related to surgery, medication, technique, side effects, and alternative therapies. Background knowledge was the focus of 36 studies and included cancer definitions, pathology, etiology, anatomy, epidemiology, and prognosis. Prevention, mentioned in 35 studies, focused on strategies to reduce cancer risk, such as screening, lifestyle changes, dietary modifications, vaccination, and raising awareness. Diagnosis, which was addressed in 30 studies, refers to identifying cancer through symptoms, tests, staging, and clinical manifestations. Personal experiences and others were the focus of 25 studies, featuring patient stories, psychological support, relationships, news coverage, and emotional responses such as fear, anxiety, and depression.

Completeness refers to how thoroughly health-related content is presented on new media platforms. Nine studies assessed completeness using predefined criteria within each study. One YouTube study used the Sahin critical appraisal tool, which consists of 12 questions covering primary, secondary, and tertiary prevention levels for pancreatic cancer [77]. Another study used Hexagonal Radar Charts to illustrate content balance and found TikTok videos on genitourinary cancer content adequately covered symptoms and examinations but lacked information on definitions and outcomes [111]. Several studies suggested the need to include adverse outcomes to ensure completeness. An evaluation of ChatGPT responses applied an informed consent measurement [122] found frequent omissions regarding treatments, risks, complications, quality-of-life impacts, and consequences of forgoing treatment for urological cancer [114]. Similarly, a study of YouTube videos on surgical treatments for spine tumors reported a greater emphasis on benefits than on complications or posttreatment sequelae, potentially biasing patients’ perceptions [65].

A study of completeness based on creators found that academic YouTube channels provide more complete information in their videos about colorectal cancer screening than other publisher types [67]. Completeness also varied by language. In a study of gastric cancer videos on TikTok, Chinese-language content from educational and health professionals was more comprehensive, while English-language videos by individual creators were more complete than their Chinese or Japanese counterparts [112].

#### Actionability and Usefulness

Actionability measures how well information enables individuals to take informed action, and usefulness indicates the extent to which the information benefits personal decision-making processes. The PEMAT-Actionability (PEMAT-A) tool, part of the PEMAT assessment, includes four strategies to evaluate this aspect, with higher scores indicating increased actionability [121]. PEMAT-A was used in 16 studies, 11 of which reported poor actionability (scores below 50%). Common reasons for low scores included missing figure interpretations and unclear or overly complex instructions [72,90]. There was some variation by cancer type. YouTube videos on urological and breast cancer [79,106] and TikTok videos on prostate cancer [110] found no actionable content. In contrast, a study of YouTube videos on testicular cancer yielded 100% actionability [93]. Further, prostate cancer videos on YouTube reported 75% [75], outperforming their TikTok counterparts, citing little or no actionability [110].

Further, 14 studies assessed the usefulness of new media information, with 4 studies reporting that over 80% of videos on hepatocellular carcinoma [78], Merkel cell carcinoma [100], Arabic-language prostate cancer [83], and Chinese-language gastric cancer [112] were deemed ‘useful’. In contrast, only 20.51% of English and 17.46% of Japanese TikTok videos on gastric cancer were rated as ‘useful’ [112], with similarly low rates (around 20%) for English and Japanese videos on skin, larynx, and gastric cancers [90,103,112]. Usefulness also varied by content source. Among YouTube videos on rectal cancer surgery and colorectal cancer screening, approximately 15% of videos by nonprofit posters and only 5.39% of videos by for-profit posters were rated as ‘useful’, according to the criteria [66,67].

#### Harmfulness

Studies evaluating the harmfulness of media content usually consider four harm-related constructs: (1) harmful actions, (2) harmful inaction, (3) harmful interactions, and (4) economic harm. Five studies used an “informative harm” assessment, calculating the ratio of harmful to positive messages. Harmful inaction was the most commonly identified issue. In one study, it accounted for 73% of harmful content in Japanese tweets [44]. Another study found that 31% of harmful messages on widely shared social media studies promoted rejecting conventional cancer treatments in favor of unproven alternatives [47]. The same study reported that economic harm (eg, out-of-pocket costs for unproven treatments or travel) comprised 27.7% of harmful content, while harmful actions (eg, suggesting potentially toxic tests or treatments) accounted for 17% [47]. In a study on YouTube videos on basal cell carcinoma, all harmful messages originated from laypersons [72].

#### Commercial Bias

Eight studies used commercial bias as a quality criterion by measuring the proportion of content originating from commercial, for-profit, or agenda-driven sources. On Pinterest, commercial bias was most prevalent in prostate cancer posts (14%), followed by bladder (7%) and kidney cancer (1%) [58]. On video-based platforms, commercial bias ranged from 10% to 27.33% [75,88,91,110]. Two studies specifically reported commercial bias in 13.2% and 17% of popular YouTube videos on bladder cancer [84,85].

#### Pooled Quality Results

Table 3 shows the pooled estimated scores for indicators for different quality criteria measurements. The pooled estimates reported moderate overall quality (DISCERN 43.58, 95% CI 37.80-49.35; GQS 49.91, 95% CI 43.31-56.50; *I*^2^=93.22%), and moderate technical quality (JAMA-BC 46.13, 95% CI 38.87-53.39; *I*^2^=75.29%; Health on the Net Foundation Code of Conduct 49.68, 95% CI 19.68-79.68; *I*^2^=94.35%). About 67% of the information was understandable to viewers (PEMAT-U 66.92, 95% CI 59.86-73.99; *I*^2^=88.69%). Approximately 27% of posts contained misinformation (27.15, 95% CI 21.36-33.35; *I*^2^=99.77%), 34% of essential topics defined by individual study were covered (completeness 34.22, 95% CI 27.96-40.48; *I*^2^=77.08%), less than half of the posts provided actionable message and were considered useful (PEMAT-A 37.24, 95% CI 18.08-58.68; *I*^2^= 98.53%; usefulness 48.86, 95% CI 26.24-71.48; *I*^2^=99.38%). Furthermore, 21% of posts contained harmful messages (harmfulness 21.15, 95% CI 8.96-36.50; *I*^2^=88.73%), and 12.46% of posts showed evidence of commercial bias (commercial bias 12.46, 95% CI 7.5-17.39; *I*^2^=94.82%). The forest plots for each indicator are in Figures S12-S22 in Multimedia Appendix 2 [5,44-117].

Results of leave-one-out sensitivity analysis were stable across all iterations, with no single study significantly altering the pooled effect. Details of the sensitivity analysis for each indicator are in Figures S23-S33 in Multimedia Appendix 2 [5,44-117].

**Table 3:** Pooled quality evaluation results using meta-analysis.

Criteria	Indicator	Study count	Sample size	Pooled quality, % (95% CI)	*P* value	*I*^2^ (%)
Overall quality	DISCERN^a^	44	3701	43.58 (37.80-49.35)	<.001	93.22
Overall quality	GQS^b,c^	26	1440	49.91 (43.31-56.50)	<.001	90.14
Technical quality	JAMA-BC^d^	12	746	46.13 (38.87-53.39)	<.001	75.29
Technical quality	HONcode^e^	3	155	49.68 (19.68-79.68)	<.001	94.35
Understandability	PEMAT-U^f,g^	16	1453	66.92 (59.86-73.99)	<.001	88.69
Accuracy	Misinformation^b^	44	345,169	27.15 (21.36-33.35)	<.001	99.77
Completeness	Completeness^a^	8	1036	34.22 (27.96-40.48)	<.001	77.08
Actionability	PEMAT-A^g,h^	16	1116	37.24 (18.08-58.68)	<.001	98.53
Actionability	Usefulness	13	1427	48.86 (26.24-71.48)	<.001	99.38
Harmfulness	Harmfulness^b^	5	395	21.15 (8.96-36.50)	<.001	88.73
Commercial bias	Commercial bias	11	4432	12.46 (7.52-17.39)	<.001	94.82

^a^Three papers were removed from the meta-analysis because they did not provide the exact score for the indicator.

^b^Contained papers with indicators reporting 0 or 100%.

^c^GQS: Global Quality Score.

^d^JAMA-BC: Journal of the American Medical Association Benchmark Criteria.

^e^HONcode: Health on the Net Foundation Code of Conduct.

^f^PEMAT-U: Patient Education Material Assessment Tool for Understandability.

^g^One paper was removed from the meta-analysis because it provided a combined score for PEMAT-U and PEMAT-A. The quality of information in individual studies in a proportional rating system is shown in Table S154.

^h^PEMAT-A: Patient Education Material Assessment Tool for Actionability.

## Discussion

### Principal Results

This systematic review and meta-analysis offer robust insight into new media-driven information quality studies pertaining to cancer. Our findings showcase a complex and diverse body of literature varying across multiple domains, including time, platform, quality assessment types, and findings therein. Several important patterns merited further discussion.

First, evidence of temporal changes highlights the evolution of media platforms over time. The studies evidenced clear changes in used platforms, reflecting their availability and popularity at the time. For example, early in the study period, we principally identified studies analyzing YouTube videos. Prior to 2021, there was also greater use of text-based media, including Twitter, Reddit, Pinterest, Facebook, and others. However, between 2021 and 2023, most studies shifted solely to video-based platforms. The shift in emphasis from text and image to video-based social media largely aligns with US patterns in that a majority of the user-generated social media content created daily since 2021 consists of videos [123,124]. This period also coincides with changes to data access policies on platforms such as Twitter (now X), which closed its application programming interface in 2022, limiting data collection efforts for research [125]. Another important point is that the second and third most popular social media platforms in the United States (Facebook and Instagram, respectively) [123] represented a small proportion of the total number of studies reviewed (6 studies on Facebook and 2 studies on Instagram). This represents a critical gap that warrants further investigation. The other temporal pattern is that the first cancer-related information quality assessment of generative AI chatbot content occurred in 2023 [117]. In the coming years, more studies are likely to expand in this area. Future research should compare the quality of information of multiple AI platforms and models—especially in the areas of response consistency and alignment with scientific best practices.

Given the proliferation of video-based social media over time, it is also critical to contextualize information quality patterns by cancer types. Our findings generally highlight that video-based cancer content is engaged but at notable risk for low-quality information. Indeed, our analysis presents high engagement (eg, views, likes, and comments) for video-based platforms, which may be due to their general accessibility, visual appeal, or targeted algorithmic marketing. This finding might suggest that cancer-related content on video-based platforms may have greater reach and visibility compared with text-based content, and empirical evidence supports this [64,126]. However, our analysis shows that information quality on video platforms is significantly lower than on text-based platforms (OR 0.02, 95% CI 0.01-0.12; *P*<.001), underscoring challenges in content moderation and infoveillance. Similarly, newly emerging AI chatbots exhibit potential to deliver cancer-related information of higher quality, as evidenced by higher DISCERN scores than video-based media [114,116,117]. However, limitations remain, particularly concerning understandability, actionability, and occasional misinformation [115-117], which may be driven by hallucinations. Future AI-development efforts should focus on ensuring more comprehensive and representative information, while improving the clarity and factual accuracy of AI-generated responses.

Second, this study highlights shifts in research priorities and interests, as well as changes in cancer types and the quality of information associated with them. Between 2014 and 2018, increased attention was given to certain cancer types, primarily breast, prostate, skin, and colorectal cancers. These cancers are all highly prevalent in the United States, and disparities in health care seeking, treatment, and outcomes affect each [127], which may explain their broad emphasis during this period. Studies of these cancers remained prevalent throughout the data collection period. However, between 2018 and 2023, research interest expanded to include rarer and less media-publicized cancers such as gastric, thyroid, and spinal cancers. This shift might reflect increasing interest in underrepresented cancers and the need to address gaps in communication and information quality across a wider range of cancer types; however, more research here is needed.

Our findings likewise demonstrated that information quality varied across the studied cancer types. Specifically, research focusing on rare cancers was of significantly lower quality compared to studies on common, highly prevalent cancers (OR 0.32, 95% CI 0.16-0.65). Indeed, studies of more common cancers such as skin, lung, breast, colorectal, prostate, liver, and thyroid consistently achieved the highest overall quality, understandability, completeness, and usefulness scores [67,68,71,72,78,83,100,104,107,116,117]. This conclusion may be explained by the relatively late initiation and limited volume of research focused on the quality of information regarding rare cancers.

Third, addressing the quality of cancer-related information on media platforms presents unique challenges and opportunities. Prior research attributes lower information quality on new media to several factors, including algorithmic amplification of popular content regardless of the source [128,129], brevity of text and short-form limiting content depth [130], and increased content from nonexpert creators with no moderation [131]. The literature highlights various recommendations for addressing these issues, including improving architecture for detecting and eliminating incorrect content [132], increasing mobile health (mHealth) and digital health media campaigns to promote health literacy [133], encouraging more medical entities to have a social media presence [133], and auditing risks and functionalities for AI-generated materials [134]. The success of these strategies is mixed, suggesting further work is needed to address the confounding issue of the reach and visibility of cancer information relative to information quality risk. Regardless, with the evident growth of health content on new media, timely attention to these issues remains a public health priority.

### Future Studies

Findings from this review offer four tangible recommendations for future research. First, while it is clear that new media information quality assessments are growing and popular fields, there is limited consensus on which information quality assessment is needed relative to the task. Based on our findings, we strongly encourage future researchers conducting such assessments to consider robust and validated tools such as DISCERN, PEMAT U/A, GQS, and the JAMA-BC criteria. Although application of these tools can be time-intensive, each provides an evaluation of health content in several domains simultaneously, ensuring thorough analysis of the data. Further, consistently using these tools for information quality assessments will allow for robust and streamlined meta-analyses, which would allow for even greater insights than cross-sectional designs alone. However, we also acknowledge that even when these tools are consistently applied, variations in study objectives, media platforms, and evolving content may still pose challenges in achieving standardized assessments across different research contexts.

Second, when conducting mHealth or digital health interventions, we advise researchers to launch them through video-based apps, such as YouTube and TikTok. Comparing studies consistently revealed greater engagement with data from these sources than text-based sources, regardless of cancer type or account attributes. This directly suggests that the broad access and ease-of-use of these videos may make health-related content more readily accessible to others, regardless of barriers to care. Prior research strongly recommends using innovative strategies such as narrative storytelling or personal testimonials to boost engagement. Regardless of the strategies, it is clear from our findings that the shift away from text-based media is not a trend but part of the shifting social media landscape. At the same time, researchers should also remain abreast of new media trends and adapt accordingly.

Third, mHealth or digital health interventions should be produced by or in partnership with specialists, clinicians, and hospitals. Studies considering content posted by professional entities consistently had better information quality across inventories, cancers, and platforms. Although in some cases, content by medical professionals was not as well-engaged as content from other nonmedical accounts, these cases may reflect content posted on text-based platforms where engagement was consistently lower. Specialists with direct expertise in the topic as video protagonists—including celebrities with lived cancer experience— may help extend the credibility of the content and transcend trust barriers for certain topics, including vaccinations and routine screenings.

Fourth, our results are only starting to show the emergence of AI as a health promotion tool. Professionals in the health and medical fields should continue researching generative AI content for information quality.

Fifth, our results equally highlight the need for licensed care providers to consider the impact of new media on patient health-related decision-making. To our knowledge, few providers, including nurse practitioners, physicians, or insurance representatives, currently inquire about how much new media patients consume during consultations. However, given that our findings indicate a wide array of information quality, coupled with profuse and growing use of new media in the United States and abroad, it may be imperative for providers to inquire about use in controlled settings.

### Limitations

First, while we believe we offered a thorough review of the new media cancer information literature, we were not able to consider other facets that may lead to varying information quality, such as the professional level of raters, content language, nation of origin, and different criteria in the searching and rating process. As this review focused only on English-language-based studies, similar reviews focusing on studies in other languages (such as Spanish, Arabic, and Chinese) and media data (such as LinkedIn, Snapchat, and other chatbots) are needed. Second, the inconsistent nomenclature of certain information quality and measurement used may be driving high levels of heterogeneity in our meta-analysis. For example, the measure of “actionability” was sometimes called “usefulness,” depending on the study. This inconsistent nomenclature made it difficult to categorize study findings, which may have resulted in a marginally biased result. Further, even in cases where studies used the same quality assessment, the diversity of platforms, cancer types, and scope of work may have also contributed to higher levels of heterogeneity. Subgroup analyses to explore the sources of heterogeneity were limited by the small number of records included in those subgroups and the poor quality of reporting in the available data. As such, pooled findings should be interpreted as indicative rather than definitive. Third, this study is minimally representative of generative AI as an information source. Given its growing popularity and strong body of literature considering generative AI as a health communication tool, we strongly recommend further systematic reviews specifically including information quality assessments using multiple generative AI tools. Meanwhile, we found a limited number of published studies specifically addressing certain platforms, such as Facebook and Instagram, during our search period. Finally, this review was prone to publication bias among studies on misinformation and other risks of bias.

### Conclusions

High-quality cancer information on new media contributed to informed cancer-related decision-making. However, the quality of this information remained at a moderate to moderate-low level, particularly in terms of overall quality, transparency, understandability, accuracy, actionability, harmfulness, and commercial bias. Our findings indicated that poster identity, medium (text-based, video-based, and AI media), and assessments of highly prevalent cancers were associated with higher information quality. Gaps in information quality, which were evident across all studies considered in our review, remain a critical and timely public health issue.

## Appendix

Multimedia Appendix 1 PRISMA 2020 checklist.

Multimedia Appendix 2 Research strategy, quality assessment results, risk of bias assessment, quality evaluation, ethical approval process report, certainty of evidence, forest plot, sensitivity analysis, funnel plot, and Egger regression test.

## Abbreviations

AI: artificial intelligence
GQS: Global Quality Score
GRADE: Grading of Recommendations Assessment, Development and Evaluation
JAMA-BC: Journal of the American Medical Association Benchmark Criteria
mHealth: mobile health
OR: odds ratio
PEMAT-U: Patient Education Materials Assessment Tool for Understandability
PEMAT-A: Patient Education Materials Assessment Tool for Actionability
PRISMA: Preferred Reporting Items for Systematic Reviews and Meta-Analyses
RQ: research question
STROBE: Strengthening the Reporting of Observational Studies in Epidemiology

## Data Availability

The datasets generated or analyzed during this study are available from the corresponding author on reasonable request.
