# Supplementary material for: Quality of Cancer-Related Information on New Media (2014-2023): Systematic Review and Meta-Analysis
Source: J Med Internet Res. 2025 Oct 8;27:e73185. doi: 10.2196/73185 (PMC12547337; doi:10.2196/73185)

Supplementary materials

Table S1. Research strategies

Tables S2-S76. Assessments of risk of bias using JBI tool

Tables S77-S151. Quality evaluation for included studies using STROBE

Table S152. Ethical approval process report

Table S153. Assessments of certainty of evidence using GRADE

Table S154. Quality assessment of information using different tools across studies in a proportional rating system

Figures S1-S11. Funnel plot for assessing publication bias in studies

Figures S12-S22. Forest plot of proportion of assessment tools in pooled analysis

Figures S23-33. Leave-one-out sensitivity analysis of studies using a random-Effects model

Table S1. Research strategies

Pubmed

(social media OR online OR twitter OR Facebook OR YouTube OR Whatsapp OR Instagram OR TikTok OR Snapchat OR Reddit OR Pinterest OR LinkedIn OR Messenger OR WeChat OR QQ OR Qzone OR Tumblr OR Weibo OR Flickr OR Tieba OR Viber OR Telegram OR Medium OR Douyin OR Quora OR Douban OR Foursquare OR Discord OR Sound Cloud OR Mix OR Next Door OR Badoo OR Deviantart OR Meetup OR kuaishou OR "Artificial Intelligence" OR "chatbot*" OR ChatGPT OR Chatsonic OR Microsoft Bing AI OR Perplexity OR "generative AI") AND

(misinformation OR fake news OR disinformation OR rumo* OR false OR mislead* OR Inaccurate OR poor quality* OR mislead* OR seeking information* OR rumer OR gossip OR hoax OR urban legend OR myth OR fallacy OR conspiracy OR malicious account* OR bots OR spam OR troll OR hate speech OR cyber bullying OR unverif* )

AND

("Neoplasms"[Majr])

Web of Science

TS=("social media" OR online OR twitter OR facebook OR youtube OR whatsapp OR instagram OR tiktok OR snapchat OR reddit OR pinterest OR linkedin OR messenger OR wechat OR qq OR qzone OR tumblr OR weibo OR flickr OR tieba OR viber OR telegram OR medium OR douyin OR quora OR douban OR foursquare OR discord OR "sound cloud" OR mix OR "next door" OR badoo OR deviantart OR meetup OR kuaishou OR "Artificial Intelligence" OR "chatbot*" OR ChatGPT OR Chatsonic OR Microsoft Bing AI OR Perplexity OR "generative AI")

AND

TS=(misinformation OR "fake news" OR disinformation OR rumor OR false OR mislead* OR inaccurate OR "poor quality*" OR "seeking information*" OR "information quality" OR rumer OR gossip OR hoax OR "urban legend" OR myth OR fallacy OR conspiracy OR "malicious account*" OR bots OR spam OR troll OR "hate speech" OR "cyber bullying" OR unverif*)

AND

TS=(cancer OR cancers OR tumor OR tumors OR neoplasm OR neoplasms OR neoplasia OR neoplasias OR malignant OR malignancy OR malignancies OR benign)

Medline

("social media"[tiab] OR online[tiab] OR twitter[tiab] OR facebook[tiab] OR youtube[tiab] OR whatsapp[tiab] OR instagram[tiab] OR tiktok[tiab] OR snapchat[tiab] OR reddit[tiab] OR pinterest[tiab] OR linkedin[tiab] OR messenger[tiab] OR wechat[tiab] OR qq[tiab] OR qzone[tiab] OR tumblr[tiab] OR weibo[tiab] OR flickr[tiab] OR tieba[tiab] OR viber[tiab] OR telegram[tiab] OR medium[tiab] OR douyin[tiab] OR quora[tiab] OR douban[tiab] OR foursquare[tiab] OR discord[tiab] OR soundcloud[tiab] OR mix[tiab] OR nextdoor[tiab] OR badoo[tiab] OR deviantart[tiab] OR meetup[tiab] OR kuaishou[tiab] OR OR "Artificial Intelligence"[tiab] OR "chatbot[tiab]" OR ChatGPT[tiab] OR Chatsonic[tiab] OR “Microsoft Bing AI”[tiab] OR Perplexity[tiab] OR "generative AI"[tiab])

AND

(misinformation[tiab] OR "fake news"[tiab] OR disinformation[tiab] OR rumor[tiab] OR rumours[tiab] OR false[tiab] OR mislead*[tiab] OR inaccurate[tiab] OR "poor quality"[tiab] OR "information quality"[tiab] OR "unverified information"[tiab] OR hoax[tiab] OR myth[tiab] OR fallacy[tiab] OR conspiracy[tiab] OR "malicious account*"[tiab] OR bots[tiab] OR spam[tiab] OR troll[tiab] OR "hate speech"[tiab] OR "cyber bullying"[tiab] OR gossip[tiab] OR urban legend[tiab] OR "health misinformation"[tiab])

AND

("Neoplasms"[Majr] OR cancer[tiab] OR cancers[tiab] OR tumor[tiab] OR tumors[tiab] OR neoplasm[tiab] OR neoplasms[tiab] OR neoplasia[tiab] OR neoplasias[tiab] OR malignancy[tiab] OR malignancies[tiab] OR carcinoma[tiab] OR malignant[tiab] OR benign[tiab])

Scopus

("social media" OR online OR twitter OR facebook OR youtube OR whatsapp OR instagram OR tiktok OR snapchat OR reddit OR pinterest OR linkedin OR messenger OR wechat OR qq OR qzone OR tumblr OR weibo OR flickr OR tieba OR viber OR telegram OR medium OR douyin OR quora OR douban OR foursquare OR discord OR soundcloud OR mix OR "next door" OR badoo OR deviantart OR meetup OR kuaishou OR "Artificial Intelligence" OR "chatbot*" OR ChatGPT OR Chatsonic OR Microsoft Bing AI OR Perplexity OR "generative AI"):ti,ab,kw

AND

(misinformation OR "fake news" OR disinformation OR rumor OR false OR mislead* OR inaccurate OR "poor quality*" OR "information quality" OR "seeking information" OR gossip OR hoax OR "urban legend" OR myth OR fallacy OR conspiracy OR "malicious account*" OR bots OR spam OR troll OR "hate speech" OR "cyber bullying" OR unverified):ti,ab,kw

AND

(cancer OR cancers OR tumor OR tumors OR neoplasm OR neoplasms OR neoplasia OR neoplasias OR malignant OR malignancy OR malignancies OR benign):ti,ab,kw

Table S2. Assessments of risk of bias using JBI tool

Author__Juliusz Jan Szczesniewski et al._Year_2023__ Record Number____1_

| | Yes | No | Unclear | Not applicable |
|---|---|---|---|---|
| 1. Were the criteria for inclusion in the sample clearly defined? | □ | □ | □ | √ |
| 2. Were the study subjects and the setting described in detail? | √ | □ | □ | □ |
| 3. Was the exposure measured in a valid and reliable way? | √ | □ | □ | □ |
| 4. Were objective, standard criteria used for measurement of the condition? | √ | □ | □ | □ |
| 5. Were confounding factors identified? | □ | √ | □ | □ |
| 6. Were strategies to deal with confounding factors stated? | □ | √ | □ | □ |
| 7. Were the outcomes measured in a valid and reliable way? | √ | □ | □ | □ |
| 8. Was appropriate statistical analysis used? | √ | □ | □ | □ |

Overall appraisal: Include √ Exclude □ Seek further info □

Comments (Including reason for exclusion)

Two urologists and DISCERN score makes the results more reliable.

Table S3. Assessments of risk of bias using JBI tool

Author___Shan chen et al.__Year____2023_____ Record Number____2_____

| | Yes | No | Unclear | Not applicable |
|---|---|---|---|---|
| 1. Were the criteria for inclusion in the sample clearly defined? | □ | □ | □ | √ |
| 2. Were the study subjects and the setting described in detail? | √ | □ | □ | □ |
| 3. Was the exposure measured in a valid and reliable way? | √ | □ | □ | □ |
| 4. Were objective, standard criteria used for measurement of the condition? | √ | □ | □ | □ |
| 5. Were confounding factors identified? | √ | □ | □ | □ |
| 6. Were strategies to deal with confounding factors stated? | √ | □ | □ | □ |
| 7. Were the outcomes measured in a valid and reliable way? | √ | □ | □ | □ |
| 8. Was appropriate statistical analysis used? | √ | □ | □ | □ |

Overall appraisal: Include √ Exclude □ Seek further info □

Comments (Including reason for exclusion)

Use a standardized approaches and scores to assess treatment________

Table S4. Assessments of risk of bias using JBI tool

Author__Alexander Pan et al.__Year____2023____ Record Number_____3____

| | Yes | No | Unclear | Not applicable |
|---|---|---|---|---|
| 1. Were the criteria for inclusion in the sample clearly defined? | □ | □ | □ | √ |
| 2. Were the study subjects and the setting described in detail? | √ | □ | □ | □ |
| 3. Was the exposure measured in a valid and reliable way? | √ | □ | □ | □ |
| 4. Were objective, standard criteria used for measurement of the condition? | √ | □ | □ | □ |
| 5. Were confounding factors identified? | √ | □ | □ | □ |
| 6. Were strategies to deal with confounding factors stated? | √ | □ | □ | □ |
| 7. Were the outcomes measured in a valid and reliable way? | √ | □ | □ | □ |
| 8. Was appropriate statistical analysis used? | √ | □ | □ | □ |

Overall appraisal: Include √ Exclude □ Seek further info □

Comments (Including reason for exclusion)

Use different chatbot platforms can be more reliable to make the conclusion.

Table S5. Assessments of risk of bias using JBI tool

Author_David Musheyev et al.___Year___2023____ Record Number____4_____

| | Yes | No | Unclear | Not applicable |
|---|---|---|---|---|
| 1. Were the criteria for inclusion in the sample clearly defined? | √ | □ | □ | □ |
| 2. Were the study subjects and the setting described in detail? | √ | □ | □ | □ |
| 3. Was the exposure measured in a valid and reliable way? | √ | □ | □ | □ |
| 4. Were objective, standard criteria used for measurement of the condition? | √ | □ | □ | □ |
| 5. Were confounding factors identified? | √ | □ | □ | □ |
| 6. Were strategies to deal with confounding factors stated? | √ | □ | □ | □ |
| 7. Were the outcomes measured in a valid and reliable way? | √ | □ | □ | □ |
| 8. Was appropriate statistical analysis used? | √ | □ | □ | □ |

Overall appraisal: Include √ Exclude □ Seek further info □

Comments (Including reason for exclusion)

This article indicated statistical method and utilized several quantitative score to measure, which makes analysis and interpretation of performance proper.

Table S6. Assessments of risk of bias using JBI tool

Author__EA Gage-Bouchard et al.___Year____2018_____ Record Number_____5____

| | Yes | No | Unclear | Not applicable |
|---|---|---|---|---|
| 1. Were the criteria for inclusion in the sample clearly defined? | √ | □ | □ | □ |
| 2. Were the study subjects and the setting described in detail? | √ | □ | □ | □ |
| 3. Was the exposure measured in a valid and reliable way? | √ | □ | □ | □ |
| 4. Were objective, standard criteria used for measurement of the condition? | √ | □ | □ | □ |
| 5. Were confounding factors identified? | √ | □ | □ | □ |
| 6. Were strategies to deal with confounding factors stated? | √ | □ | □ | □ |
| 7. Were the outcomes measured in a valid and reliable way? | √ | □ | □ | □ |
| 8. Was appropriate statistical analysis used? | √ | □ | □ | □ |

Overall appraisal: Include √ Exclude □ Seek further info □

Comments (Including reason for exclusion)

Cancer-related posts are rated by oncology experts using predefined coding rules in different categories, which might cause more bias.

Table S7. Assessments of risk of bias using JBI tool

Author__Tatyana A Petukhova et al.__Year__2020___ Record Number____6_____

| | Yes | No | Unclear | Not applicable |
|---|---|---|---|---|
| 1. Were the criteria for inclusion in the sample clearly defined? | √ | □ | □ | □ |
| 2. Were the study subjects and the setting described in detail? | √ | □ | □ | □ |
| 3. Was the exposure measured in a valid and reliable way? | √ | □ | □ | □ |
| 4. Were objective, standard criteria used for measurement of the condition? | √ | □ | □ | □ |
| 5. Were confounding factors identified? | □ | √ | □ | □ |
| 6. Were strategies to deal with confounding factors stated? | □ | √ | □ | □ |
| 7. Were the outcomes measured in a valid and reliable way? | √ | □ | □ | □ |
| 8. Was appropriate statistical analysis used? | √ | □ | □ | □ |

Overall appraisal: Include √ Exclude □ Seek further info □

Comments (Including reason for exclusion)

Modelling confounding factors like gender, age, ethnicity and time might be beneficial for the analysis.

Table S8. Assessments of risk of bias using JBI tool

Author__Skyler B Johnson et al._Year__2022____ Record Number____7_____

| | Yes | No | Unclear | Not applicable |
|---|---|---|---|---|
| 1. Were the criteria for inclusion in the sample clearly defined? | √ | □ | □ | □ |
| 2. Were the study subjects and the setting described in detail? | √ | □ | □ | □ |
| 3. Was the exposure measured in a valid and reliable way? | √ | □ | □ | □ |
| 4. Were objective, standard criteria used for measurement of the condition? | √ | □ | □ | □ |
| 5. Were confounding factors identified? | □ | √ | □ | □ |
| 6. Were strategies to deal with confounding factors stated? | □ | √ | □ | □ |
| 7. Were the outcomes measured in a valid and reliable way? | √ | □ | □ | □ |
| 8. Was appropriate statistical analysis used? | √ | □ | □ | □ |

Overall appraisal: Include √ Exclude □ Seek further info □

Comments (Including reason for exclusion)

This paper is impressive because it used statistical regression to analyse the relationship between harm and accuracy.

Table S9. Assessments of risk of bias using JBI tool

Author__Álvaro Iglesias-Puzas et al._ Year__2021___ Record Number____8_____

| | Yes | No | Unclear | Not applicable |
|---|---|---|---|---|
| 1. Were the criteria for inclusion in the sample clearly defined? | √ | □ | □ | □ |
| 2. Were the study subjects and the setting described in detail? | √ | □ | □ | □ |
| 3. Was the exposure measured in a valid and reliable way? | √ | □ | □ | □ |
| 4. Were objective, standard criteria used for measurement of the condition? | √ | □ | □ | □ |
| 5. Were confounding factors identified? | √ | □ | □ | □ |
| 6. Were strategies to deal with confounding factors stated? | √ | □ | □ | □ |
| 7. Were the outcomes measured in a valid and reliable way? | √ | □ | □ | □ |
| 8. Was appropriate statistical analysis used? | √ | □ | □ | □ |

Overall appraisal: Include √ Exclude □ Seek further info □

Comments (Including reason for exclusion)

The inclusion criteria and coding process were well defined, and outcome measures were clearly
described.

Table S10. Assessments of risk of bias using JBI tool

Author_Muhannad Alsyouf et al._ Year__2019___ Record Number____9_____

| | Yes | No | Unclear | Not applicable |
|---|---|---|---|---|
| 1. Were the criteria for inclusion in the sample clearly defined? | □ | □ | □ | √ |
| 2. Were the study subjects and the setting described in detail? | √ | □ | □ | □ |
| 3. Was the exposure measured in a valid and reliable way? | √ | □ | □ | □ |
| 4. Were objective, standard criteria used for measurement of the condition? | √ | □ | □ | □ |
| 5. Were confounding factors identified? | √ | □ | □ | □ |
| 6. Were strategies to deal with confounding factors stated? | √ | □ | □ | □ |
| 7. Were the outcomes measured in a valid and reliable way? | √ | □ | □ | □ |
| 8. Was appropriate statistical analysis used? | √ | □ | □ | □ |

Overall appraisal: Include √ Exclude □ Seek further info □

Comments (Including reason for exclusion)

Confounding factors (e.g., source reputation, posting platform, or demographic engagement variation) were not identified or adjusted for.

Table S11. Assessments of risk of bias using JBI tool

Author__Philip M Masseyet al.___Year__2020_____ Record Number____10_____

| | Yes | No | Unclear | Not applicable |
|---|---|---|---|---|
| 1. Were the criteria for inclusion in the sample clearly defined? | √ | □ | □ | □ |
| 2. Were the study subjects and the setting described in detail? | √ | □ | □ | □ |
| 3. Was the exposure measured in a valid and reliable way? | √ | □ | □ | □ |
| 4. Were objective, standard criteria used for measurement of the condition? | √ | □ | □ | □ |
| 5. Were confounding factors identified? | □ | √ | □ | □ |
| 6. Were strategies to deal with confounding factors stated? | □ | √ | □ | □ |
| 7. Were the outcomes measured in a valid and reliable way? | √ | □ | □ | □ |
| 8. Was appropriate statistical analysis used? | √ | □ | □ | □ |

Overall appraisal: Include √ Exclude □ Seek further info □

Comments (Including reason for exclusion)

The network metrics were attractive and appropriate for the study goals.

Table S12. Assessments of risk of bias using JBI tool

Author_Tamar Wilneret al._ Year__2020_____ Record Number____11_____

| | Yes | No | Unclear | Not applicable |
|---|---|---|---|---|
| 1. Were the criteria for inclusion in the sample clearly defined? | √ | □ | □ | □ |
| 2. Were the study subjects and the setting described in detail? | √ | □ | □ | □ |
| 3. Was the exposure measured in a valid and reliable way? | √ | □ | □ | □ |
| 4. Were objective, standard criteria used for measurement of the condition? | √ | □ | □ | □ |
| 5. Were confounding factors identified? | √ | □ | □ | □ |
| 6. Were strategies to deal with confounding factors stated? | √ | □ | □ | □ |
| 7. Were the outcomes measured in a valid and reliable way? | √ | □ | □ | □ |
| 8. Was appropriate statistical analysis used? | √ | □ | □ | □ |

Overall appraisal: Include √ Exclude □ Seek further info □

Comments (Including reason for exclusion)

The article defined clear inclusion criteria and used reliable, validated sources to classify the accuracy of content.

Table S13. Assessments of risk of bias using JBI tool

Author_Amber S Herbert et al.__ Year__2022_____ Record Number____12_____

| | Yes | No | Unclear | Not applicable |
|---|---|---|---|---|
| 1. Were the criteria for inclusion in the sample clearly defined? | √ | □ | □ | □ |
| 2. Were the study subjects and the setting described in detail? | √ | □ | □ | □ |
| 3. Was the exposure measured in a valid and reliable way? | √ | □ | □ | □ |
| 4. Were objective, standard criteria used for measurement of the condition? | √ | □ | □ | □ |
| 5. Were confounding factors identified? | √ | □ | □ | □ |
| 6. Were strategies to deal with confounding factors stated? | √ | □ | □ | □ |
| 7. Were the outcomes measured in a valid and reliable way? | √ | □ | □ | □ |
| 8. Was appropriate statistical analysis used? | √ | □ | □ | □ |

Overall appraisal: Include √ Exclude □ Seek further info □

Comments (Including reason for exclusion)

The authors clearly defined the inclusion criteria and used validated instruments (DISCERN, PEMAT) to evaluate outcomes.

Table S14. Assessments of risk of bias using JBI tool

Author_Jingcheng Du et al._ Year____2021_____ Record Number____13_____

| | Yes | No | Unclear | Not applicable |
|---|---|---|---|---|
| 1. Were the criteria for inclusion in the sample clearly defined? | √ | □ | □ | □ |
| 2. Were the study subjects and the setting described in detail? | √ | □ | □ | □ |
| 3. Was the exposure measured in a valid and reliable way? | √ | □ | □ | □ |
| 4. Were objective, standard criteria used for measurement of the condition? | √ | □ | □ | □ |
| 5. Were confounding factors identified? | □ | √ | □ | □ |
| 6. Were strategies to deal with confounding factors stated? | □ | √ | □ | □ |
| 7. Were the outcomes measured in a valid and reliable way? | √ | □ | □ | □ |
| 8. Was appropriate statistical analysis used? | √ | □ | □ | □ |

Overall appraisal: Include √ Exclude □ Seek further info □

Comments (Including reason for exclusion)

This article is appealing for applying different machine learning models to compare their evaluation for misinformation about HPV vaccine and the analytic process was robust.

Table S15. Assessments of risk of bias using JBI tool

Author__Li Wang et al._ Year____2023_____ Record Number____14_____

| | Yes | No | Unclear | Not applicable |
|---|---|---|---|---|
| 1. Were the criteria for inclusion in the sample clearly defined? | √ | □ | □ | □ |
| 2. Were the study subjects and the setting described in detail? | √ | □ | □ | □ |
| 3. Was the exposure measured in a valid and reliable way? | √ | □ | □ | □ |
| 4. Were objective, standard criteria used for measurement of the condition? | √ | □ | □ | □ |
| 5. Were confounding factors identified? | □ | √ | □ | □ |
| 6. Were strategies to deal with confounding factors stated? | □ | √ | □ | □ |
| 7. Were the outcomes measured in a valid and reliable way? | √ | □ | □ | □ |
| 8. Was appropriate statistical analysis used? | √ | □ | □ | □ |

Overall appraisal: Include √ Exclude □ Seek further info □

Comments (Including reason for exclusion)

The statistical methods used were appropriate and the data were well presented.

Table S16. Assessments of risk of bias using JBI tool

Author_Shijie Yang et al._Year____2022_____ Record Number____15_____

| | Yes | No | Unclear | Not applicable |
|---|---|---|---|---|
| 1. Were the criteria for inclusion in the sample clearly defined? | √ | □ | □ | □ |
| 2. Were the study subjects and the setting described in detail? | √ | □ | □ | □ |
| 3. Was the exposure measured in a valid and reliable way? | √ | □ | □ | □ |
| 4. Were objective, standard criteria used for measurement of the condition? | √ | □ | □ | □ |
| 5. Were confounding factors identified? | √ | □ | □ | □ |
| 6. Were strategies to deal with confounding factors stated? | √ | □ | □ | □ |
| 7. Were the outcomes measured in a valid and reliable way? | √ | □ | □ | □ |
| 8. Was appropriate statistical analysis used? | √ | □ | □ | □ |

Overall appraisal: Include √ Exclude □ Seek further info □

Comments (Including reason for exclusion)

The study clearly used different reliable assessment tools, and applied appropriate statistical tests.

Table S17. Assessments of risk of bias using JBI tool

Author_Shusen Zhenget al._ Year____2020_____ Record Number____16_____

| | Yes | No | Unclear | Not applicable |
|---|---|---|---|---|
| 1. Were the criteria for inclusion in the sample clearly defined? | √ | □ | □ | □ |
| 2. Were the study subjects and the setting described in detail? | √ | □ | □ | □ |
| 3. Was the exposure measured in a valid and reliable way? | √ | □ | □ | □ |
| 4. Were objective, standard criteria used for measurement of the condition? | √ | □ | □ | □ |
| 5. Were confounding factors identified? | √ | □ | □ | □ |
| 6. Were strategies to deal with confounding factors stated? | √ | □ | □ | □ |
| 7. Were the outcomes measured in a valid and reliable way? | √ | □ | □ | □ |
| 8. Was appropriate statistical analysis used? | √ | □ | □ | □ |

Overall appraisal: Include √ Exclude □ Seek further info □

Comments (Including reason for exclusion)

The inclusion criteria and coding procedures were well defined, and interrater reliability was strong. Statistical analysis was appropriate,

Table S18. Assessments of risk of bias using JBI tool

Author__Alex J. Xu et al.__ Year____2021_____ Record Number____17_____

| | Yes | No | Unclear | Not applicable |
|---|---|---|---|---|
| 1. Were the criteria for inclusion in the sample clearly defined? | √ | □ | □ | □ |
| 2. Were the study subjects and the setting described in detail? | √ | □ | □ | □ |
| 3. Was the exposure measured in a valid and reliable way? | √ | □ | □ | □ |
| 4. Were objective, standard criteria used for measurement of the condition? | √ | □ | □ | □ |
| 5. Were confounding factors identified? | □ | √ | □ | □ |
| 6. Were strategies to deal with confounding factors stated? | □ | √ | □ | □ |
| 7. Were the outcomes measured in a valid and reliable way? | √ | □ | □ | □ |
| 8. Was appropriate statistical analysis used? | √ | □ | □ | □ |

Overall appraisal: Include √ Exclude □ Seek further info □

Comments (Including reason for exclusion)

Potential confounding factors such as video source influence and visibility were not identified or adjusted for.________________________________________________________________

Table S19. Assessments of risk of bias using JBI tool

Author_Xiaoqiang Xue et al.__ Year____2022_____ Record Number____18_____

| | Yes | No | Unclear | Not applicable |
|---|---|---|---|---|
| 1. Were the criteria for inclusion in the sample clearly defined? | √ | □ | □ | □ |
| 2. Were the study subjects and the setting described in detail? | √ | □ | □ | □ |
| 3. Was the exposure measured in a valid and reliable way? | √ | □ | □ | □ |
| 4. Were objective, standard criteria used for measurement of the condition? | √ | □ | □ | □ |
| 5. Were confounding factors identified? | √ | □ | □ | □ |
| 6. Were strategies to deal with confounding factors stated? | √ | □ | □ | □ |
| 7. Were the outcomes measured in a valid and reliable way? | √ | □ | □ | □ |
| 8. Was appropriate statistical analysis used? | √ | □ | □ | □ |

Overall appraisal: Include √ Exclude □ Seek further info □

Comments (Including reason for exclusion)

It applied validated instruments such as DISCERN and HONCode to evaluate quality and reliability. The HONCode is a straightforward graphic way to show the reliability.

Table S20. Assessments of risk of bias using JBI tool

Author__Ren-hao Hu et al._Year____2022_____ Record Number____19_____

| | Yes | No | Unclear | Not applicable |
|---|---|---|---|---|
| 1. Were the criteria for inclusion in the sample clearly defined? | √ | □ | □ | □ |
| 2. Were the study subjects and the setting described in detail? | √ | □ | □ | □ |
| 3. Was the exposure measured in a valid and reliable way? | √ | □ | □ | □ |
| 4. Were objective, standard criteria used for measurement of the condition? | √ | □ | □ | □ |
| 5. Were confounding factors identified? | √ | □ | □ | □ |
| 6. Were strategies to deal with confounding factors stated? | √ | □ | □ | □ |
| 7. Were the outcomes measured in a valid and reliable way? | √ | □ | □ | □ |
| 8. Was appropriate statistical analysis used? | √ | □ | □ | □ |

Overall appraisal: Include √ Exclude □ Seek further info □

Comments (Including reason for exclusion)

Statistical analysis methods were appropriate for the study design and listed clearly in the Methods part.

Table S21. Assessments of risk of bias using JBI tool

Author_Anthony Nastasi et al._ Year____2018_____ Record Number____20_____

| | Yes | No | Unclear | Not applicable |
|---|---|---|---|---|
| 1. Were the criteria for inclusion in the sample clearly defined? | √ | □ | □ | □ |
| 2. Were the study subjects and the setting described in detail? | √ | □ | □ | □ |
| 3. Was the exposure measured in a valid and reliable way? | √ | □ | □ | □ |
| 4. Were objective, standard criteria used for measurement of the condition? | √ | □ | □ | □ |
| 5. Were confounding factors identified? | □ | √ | □ | □ |
| 6. Were strategies to deal with confounding factors stated? | □ | √ | □ | □ |
| 7. Were the outcomes measured in a valid and reliable way? | √ | □ | □ | □ |
| 8. Was appropriate statistical analysis used? | √ | □ | □ | □ |

Overall appraisal: Include √ Exclude □ Seek further info □

Comments (Including reason for exclusion)

The focus on both content accuracy and user demographics are appealing and persuading.________________________________________________________________
________________________________________________________________________
________________________________________________________________

Table S22. Assessments of risk of bias using JBI tool

Author_Melanie L. Kornides et al. Year____2022_____ Record Number____21_____

| | Yes | No | Unclear | Not applicable |
|---|---|---|---|---|
| 1. Were the criteria for inclusion in the sample clearly defined? | √ | □ | □ | □ |
| 2. Were the study subjects and the setting described in detail? | √ | □ | □ | □ |
| 3. Was the exposure measured in a valid and reliable way? | √ | □ | □ | □ |
| 4. Were objective, standard criteria used for measurement of the condition? | √ | □ | □ | □ |
| 5. Were confounding factors identified? | √ | □ | □ | □ |
| 6. Were strategies to deal with confounding factors stated? | √ | □ | □ | □ |
| 7. Were the outcomes measured in a valid and reliable way? | √ | □ | □ | □ |
| 8. Was appropriate statistical analysis used? | √ | □ | □ | □ |

Overall appraisal: Include √ Exclude □ Seek further info □

Comments (Including reason for exclusion)

The paper controlled for confounders and run multivariate analysis to study the relationship of audience engagement. It's more thorough and reliable.

Table S23. Assessments of risk of bias using JBI tool

Author_Nari Kureyama et al._ Year___2023_____ Record Number____22_____

| | Yes | No | Unclear | Not applicable |
|---|---|---|---|---|
| 1. Were the criteria for inclusion in the sample clearly defined? | √ | □ | □ | □ |
| 2. Were the study subjects and the setting described in detail? | √ | □ | □ | □ |
| 3. Was the exposure measured in a valid and reliable way? | √ | □ | □ | □ |
| 4. Were objective, standard criteria used for measurement of the condition? | √ | □ | □ | □ |
| 5. Were confounding factors identified? | □ | √ | □ | □ |
| 6. Were strategies to deal with confounding factors stated? | □ | √ | □ | □ |
| 7. Were the outcomes measured in a valid and reliable way? | √ | □ | □ | □ |
| 8. Was appropriate statistical analysis used? | √ | □ | □ | □ |

Overall appraisal: Include √ Exclude □ Seek further info □

Comments (Including reason for exclusion)

The paper was a well-designed cross-sectional study with clearly defined eligibility criteria and a rigorous approach to assessing misinformation and harmful content.________________________________________________________________

Table S24. Assessments of risk of bias using JBI tool

Author__Tre Tomaszewski et al._ Year__2021___ Record Number____23_____

| | Yes | No | Unclear | Not applicable |
|---|---|---|---|---|
| 1. Were the criteria for inclusion in the sample clearly defined? | √ | □ | □ | □ |
| 2. Were the study subjects and the setting described in detail? | √ | □ | □ | □ |
| 3. Was the exposure measured in a valid and reliable way? | √ | □ | □ | □ |
| 4. Were objective, standard criteria used for measurement of the condition? | √ | □ | □ | □ |
| 5. Were confounding factors identified? | □ | √ | □ | □ |
| 6. Were strategies to deal with confounding factors stated? | □ | √ | □ | □ |
| 7. Were the outcomes measured in a valid and reliable way? | √ | □ | □ | □ |
| 8. Was appropriate statistical analysis used? | √ | □ | □ | □ |

Overall appraisal: Include √ Exclude □ Seek further info □

Comments (Including reason for exclusion)

It's good to comprehensively and systematically using machine learning and NLP for misinformation detection and causality mining. And the article also creatively use predicted model.___________________________________________________________________
__________________________________________________________________________
__________________________________________________________________________
__________________________________________________________________________
________

Table S25. Assessments of risk of bias using JBI tool

Author__SoHyun Park et al.__Year__2016___ Record Number____24_____

| | Yes | No | Unclear | Not applicable |
|---|---|---|---|---|
| 1. Were the criteria for inclusion in the sample clearly defined? | √ | □ | □ | □ |
| 2. Were the study subjects and the setting described in detail? | √ | □ | □ | □ |
| 3. Was the exposure measured in a valid and reliable way? | √ | □ | □ | □ |
| 4. Were objective, standard criteria used for measurement of the condition? | √ | □ | □ | □ |
| 5. Were confounding factors identified? | √ | □ | □ | □ |
| 6. Were strategies to deal with confounding factors stated? | √ | □ | □ | □ |
| 7. Were the outcomes measured in a valid and reliable way? | √ | □ | □ | □ |
| 8. Was appropriate statistical analysis used? | √ | □ | □ | □ |

Overall appraisal: Include √ Exclude □ Seek further info □

Comments (Including reason for exclusion)

The study utilized different categorization to evaluate tweet quality, which is informative and
promote.

Table S26. Assessments of risk of bias using JBI tool

Author__Keun Chul Lee et al. Year__2016___ Record Number____25_____

| | Yes | No | Unclear | Not applicable |
|---|---|---|---|---|
| 1. Were the criteria for inclusion in the sample clearly defined? | √ | □ | □ | □ |
| 2. Were the study subjects and the setting described in detail? | √ | □ | □ | □ |
| 3. Was the exposure measured in a valid and reliable way? | √ | □ | □ | □ |
| 4. Were objective, standard criteria used for measurement of the condition? | √ | □ | □ | □ |
| 5. Were confounding factors identified? | □ | √ | □ | □ |
| 6. Were strategies to deal with confounding factors stated? | □ | √ | □ | □ |
| 7. Were the outcomes measured in a valid and reliable way? | √ | □ | □ | □ |
| 8. Was appropriate statistical analysis used? | √ | □ | □ | □ |

Overall appraisal: Include √ Exclude □ Seek further info □

Comments (Including reason for exclusion)

The article utilized prospective study to study the quality of tweets, which would relatively objectively observe the future trend.

Table S27. Assessments of risk of bias using JBI tool

Author__Anita Lavorgna et al. Year____2021_____ Record Number____26_____

| | Yes | No | Unclear | Not applicable |
|---|---|---|---|---|
| 1. Were the criteria for inclusion in the sample clearly defined? | √ | □ | □ | □ |
| 2. Were the study subjects and the setting described in detail? | √ | □ | □ | □ |
| 3. Was the exposure measured in a valid and reliable way? | √ | □ | □ | □ |
| 4. Were objective, standard criteria used for measurement of the condition? | √ | □ | □ | □ |
| 5. Were confounding factors identified? | □ | √ | □ | □ |
| 6. Were strategies to deal with confounding factors stated? | □ | √ | □ | □ |
| 7. Were the outcomes measured in a valid and reliable way? | √ | □ | □ | □ |
| 8. Was appropriate statistical analysis used? | √ | □ | □ | □ |

Overall appraisal: Include √ Exclude □ Seek further info □

Comments (Including reason for exclusion)

The article creatively utilized network data analysis to evaluate the content and the analysis was comprehensive.

Table S28. Assessments of risk of bias using JBI tool

Author__Siyu Shi et al._ Year__2019___ Record Number____27_____

| | Yes | No | Unclear | Not applicable |
|---|---|---|---|---|
| 1. Were the criteria for inclusion in the sample clearly defined? | √ | □ | □ | □ |
| 2. Were the study subjects and the setting described in detail? | √ | □ | □ | □ |
| 3. Was the exposure measured in a valid and reliable way? | √ | □ | □ | □ |
| 4. Were objective, standard criteria used for measurement of the condition? | √ | □ | □ | □ |
| 5. Were confounding factors identified? | □ | √ | □ | □ |
| 6. Were strategies to deal with confounding factors stated? | □ | √ | □ | □ |
| 7. Were the outcomes measured in a valid and reliable way? | √ | □ | □ | □ |
| 8. Was appropriate statistical analysis used? | √ | □ | □ | □ |

Overall appraisal: Include √ Exclude □ Seek further info □

Comments (Including reason for exclusion)

The article creatively utilized RSV trend to study ___ Cannabis Cancer Cure. The statistical methods were appropriate.

Table S29. Assessments of risk of bias using JBI tool

Author_Wenjuan Yang et al.___Year__2023___ Record Number____28_____

| | Yes | No | Unclear | Not applicable |
|---|---|---|---|---|
| 1. Were the criteria for inclusion in the sample clearly defined? | √ | □ | □ | □ |
| 2. Were the study subjects and the setting described in detail? | √ | □ | □ | □ |
| 3. Was the exposure measured in a valid and reliable way? | √ | □ | □ | □ |
| 4. Were objective, standard criteria used for measurement of the condition? | √ | □ | □ | □ |
| 5. Were confounding factors identified? | √ | □ | □ | □ |
| 6. Were strategies to deal with confounding factors stated? | √ | □ | □ | □ |
| 7. Were the outcomes measured in a valid and reliable way? | √ | □ | □ | □ |
| 8. Was appropriate statistical analysis used? | √ | □ | □ | □ |

Overall appraisal: Include √ Exclude □ Seek further info □

Comments (Including reason for exclusion)

The article thoroughly extracted and studied WPA and their articles, giving useful and inferable suggestions for future promotion.

Table S30. Assessments of risk of bias using JBI tool

Author_Liang Chen et al. Year____2018_____ Record Number____29_____

| | Yes | No | Unclear | Not applicable |
|---|---|---|---|---|
| 1. Were the criteria for inclusion in the sample clearly defined? | √ | □ | □ | □ |
| 2. Were the study subjects and the setting described in detail? | √ | □ | □ | □ |
| 3. Was the exposure measured in a valid and reliable way? | √ | □ | □ | □ |
| 4. Were objective, standard criteria used for measurement of the condition? | √ | □ | □ | □ |
| 5. Were confounding factors identified? | √ | □ | □ | □ |
| 6. Were strategies to deal with confounding factors stated? | √ | □ | □ | □ |
| 7. Were the outcomes measured in a valid and reliable way? | √ | □ | □ | □ |
| 8. Was appropriate statistical analysis used? | √ | □ | □ | □ |

Overall appraisal: Include √ Exclude □ Seek further info □

Comments (Including reason for exclusion)

The article thoroughly listed background and create different angles to study the tweets. The statistical methods were appropriate and discussion was insightful.

Table S31. Assessments of risk of bias using JBI tool

Author__Peng Pan et al._Year____2020_____ Record Number____30_____

| | Yes | No | Unclear | Not applicable |
|---|---|---|---|---|
| 1. Were the criteria for inclusion in the sample clearly defined? | √ | □ | □ | □ |
| 2. Were the study subjects and the setting described in detail? | √ | □ | □ | □ |
| 3. Was the exposure measured in a valid and reliable way? | √ | □ | □ | □ |
| 4. Were objective, standard criteria used for measurement of the condition? | √ | □ | □ | □ |
| 5. Were confounding factors identified? | □ | √ | □ | □ |
| 6. Were strategies to deal with confounding factors stated? | □ | √ | □ | □ |
| 7. Were the outcomes measured in a valid and reliable way? | √ | □ | □ | □ |
| 8. Was appropriate statistical analysis used? | √ | □ | □ | □ |

Overall appraisal: Include √ Exclude □ Seek further info □

Comments (Including reason for exclusion)

The article studied a Chinese vudeo app and utilized validated DISCERN tool to evaluate video quality. The sample size is decent for analysis.

Table S32. Assessments of risk of bias using JBI tool

Author_Isil Yurdaisik et al._ Year____2020_____ Record Number____31_____

| | Yes | No | Unclear | Not applicable |
|---|---|---|---|---|
| 1. Were the criteria for inclusion in the sample clearly defined? | √ | □ | □ | □ |
| 2. Were the study subjects and the setting described in detail? | √ | □ | □ | □ |
| 3. Was the exposure measured in a valid and reliable way? | √ | □ | □ | □ |
| 4. Were objective, standard criteria used for measurement of the condition? | √ | □ | □ | □ |
| 5. Were confounding factors identified? | □ | √ | □ | □ |
| 6. Were strategies to deal with confounding factors stated? | □ | √ | □ | □ |
| 7. Were the outcomes measured in a valid and reliable way? | √ | □ | □ | □ |
| 8. Was appropriate statistical analysis used? | √ | □ | □ | □ |

Overall appraisal: Include √ Exclude □ Seek further info □

Comments (Including reason for exclusion)

The article utilized DISCERN and JAMA score to evaluate video quality, although the sample size is not large, the logic and results were impressive.

Table S33. Assessments of risk of bias using JBI tool

Author_Michelle A. Richardson et al._Year__2022___ Record Number____32_____

| | Yes | No | Unclear | Not applicable |
|---|---|---|---|---|
| 1. Were the criteria for inclusion in the sample clearly defined? | √ | □ | □ | □ |
| 2. Were the study subjects and the setting described in detail? | √ | □ | □ | □ |
| 3. Was the exposure measured in a valid and reliable way? | √ | □ | □ | □ |
| 4. Were objective, standard criteria used for measurement of the condition? | √ | □ | □ | □ |
| 5. Were confounding factors identified? | □ | √ | □ | □ |
| 6. Were strategies to deal with confounding factors stated? | □ | √ | □ | □ |
| 7. Were the outcomes measured in a valid and reliable way? | √ | □ | □ | □ |
| 8. Was appropriate statistical analysis used? | √ | □ | □ | □ |

Overall appraisal: Include √ Exclude □ Seek further info □

Comments (Including reason for exclusion)

The article utilized validated tools to evaluate video quality and reliability, and the discussion part is insightful.

Table S34. Assessments of risk of bias using JBI tool

Author_Emir Çapkınoğlu et al._Year__2023_____ Record Number____33_____

| | Yes | No | Unclear | Not applicable |
|---|---|---|---|---|
| 1. Were the criteria for inclusion in the sample clearly defined? | √ | □ | □ | □ |
| 2. Were the study subjects and the setting described in detail? | √ | □ | □ | □ |
| 3. Was the exposure measured in a valid and reliable way? | √ | □ | □ | □ |
| 4. Were objective, standard criteria used for measurement of the condition? | √ | □ | □ | □ |
| 5. Were confounding factors identified? | √ | □ | □ | □ |
| 6. Were strategies to deal with confounding factors stated? | √ | □ | □ | □ |
| 7. Were the outcomes measured in a valid and reliable way? | √ | □ | □ | □ |
| 8. Was appropriate statistical analysis used? | √ | □ | □ | □ |

Overall appraisal: Include √ Exclude □ Seek further info □

Comments (Including reason for exclusion)

The article used different comparisons to indicate and infer how to build better video platform.

Table S35. Assessments of risk of bias using JBI tool

Author_Omer Yalkin et al._Year____2022_____ Record Number____34_____

| | Yes | No | Unclear | Not applicable |
|---|---|---|---|---|
| 1. Were the criteria for inclusion in the sample clearly defined? | √ | □ | □ | □ |
| 2. Were the study subjects and the setting described in detail? | √ | □ | □ | □ |
| 3. Was the exposure measured in a valid and reliable way? | √ | □ | □ | □ |
| 4. Were objective, standard criteria used for measurement of the condition? | √ | □ | □ | □ |
| 5. Were confounding factors identified? | √ | □ | □ | □ |
| 6. Were strategies to deal with confounding factors stated? | √ | □ | □ | □ |
| 7. Were the outcomes measured in a valid and reliable way? | √ | □ | □ | □ |
| 8. Was appropriate statistical analysis used? | √ | □ | □ | □ |

Overall appraisal: Include √ Exclude □ Seek further info □

Comments (Including reason for exclusion)

The article utilized validated tools to evaluate video quality an reliability, giving informative suggestions for future promotion.________________________________________________________________

Table S36. Assessments of risk of bias using JBI tool

Author_Lydia Reinhardt et al.__Year____2022_____ Record Number____35_____

| | Yes | No | Unclear | Not applicable |
|---|---|---|---|---|
| 1. Were the criteria for inclusion in the sample clearly defined? | √ | □ | □ | □ |
| 2. Were the study subjects and the setting described in detail? | √ | □ | □ | □ |
| 3. Was the exposure measured in a valid and reliable way? | √ | □ | □ | □ |
| 4. Were objective, standard criteria used for measurement of the condition? | √ | □ | □ | □ |
| 5. Were confounding factors identified? | □ | √ | □ | □ |
| 6. Were strategies to deal with confounding factors stated? | □ | √ | □ | □ |
| 7. Were the outcomes measured in a valid and reliable way? | √ | □ | □ | □ |
| 8. Was appropriate statistical analysis used? | √ | □ | □ | □ |

Overall appraisal: Include √ Exclude □ Seek further info □

Comments (Including reason for exclusion)

Although the article didn't separate each part, the flow of analysis is straightforward and reliable.___________________________________________________________________
_____________________________________________________________________________
_____________________________________________________________________________
_____________________________________________________________________________
__________

Table S37. Assessments of risk of bias using JBI tool

Author_Gülay Altan Şallı et al._Year____2020_____ Record Number____36_____

| | Yes | No | Unclear | Not applicable |
|---|---|---|---|---|
| 1. Were the criteria for inclusion in the sample clearly defined? | √ | □ | □ | □ |
| 2. Were the study subjects and the setting described in detail? | √ | □ | □ | □ |
| 3. Was the exposure measured in a valid and reliable way? | √ | □ | □ | □ |
| 4. Were objective, standard criteria used for measurement of the condition? | √ | □ | □ | □ |
| 5. Were confounding factors identified? | √ | □ | □ | □ |
| 6. Were strategies to deal with confounding factors stated? | √ | □ | □ | □ |
| 7. Were the outcomes measured in a valid and reliable way? | √ | □ | □ | □ |
| 8. Was appropriate statistical analysis used? | √ | □ | □ | □ |

Overall appraisal: Include √ Exclude □ Seek further info □

Comments (Including reason for exclusion)

The article explained from different context to evaluate video quality and give suggestion, which made the results more thorough and cautious.

Table S38. Assessments of risk of bias using JBI tool

Author_Faten F. Kharbat et al.__Year____2023_____ Record Number____37_____

| | Yes | No | Unclear | Not applicable |
|---|---|---|---|---|
| 1. Were the criteria for inclusion in the sample clearly defined? | √ | □ | □ | □ |
| 2. Were the study subjects and the setting described in detail? | √ | □ | □ | □ |
| 3. Was the exposure measured in a valid and reliable way? | √ | □ | □ | □ |
| 4. Were objective, standard criteria used for measurement of the condition? | √ | □ | □ | □ |
| 5. Were confounding factors identified? | □ | √ | □ | □ |
| 6. Were strategies to deal with confounding factors stated? | □ | √ | □ | □ |
| 7. Were the outcomes measured in a valid and reliable way? | √ | □ | □ | □ |
| 8. Was appropriate statistical analysis used? | √ | □ | □ | □ |

Overall appraisal: Include √ Exclude □ Seek further info □

Comments (Including reason for exclusion)

The article used Phython to extract videos, making the selected videos more thorough. And it also came up with a framework to help with future promotion.

Table S39. Assessments of risk of bias using JBI tool

Author_Brandon S. Chai et al.__Year____2023_____ Record Number____38____

| | Yes | No | Unclear | Not applicable |
|---|---|---|---|---|
| 1. Were the criteria for inclusion in the sample clearly defined? | √ | □ | □ | □ |
| 2. Were the study subjects and the setting described in detail? | √ | □ | □ | □ |
| 3. Was the exposure measured in a valid and reliable way? | √ | □ | □ | □ |
| 4. Were objective, standard criteria used for measurement of the condition? | √ | □ | □ | □ |
| 5. Were confounding factors identified? | √ | □ | □ | □ |
| 6. Were strategies to deal with confounding factors stated? | √ | □ | □ | □ |
| 7. Were the outcomes measured in a valid and reliable way? | √ | □ | □ | □ |
| 8. Was appropriate statistical analysis used? | √ | □ | □ | □ |

Overall appraisal: Include √ Exclude □ Seek further info □

Comments (Including reason for exclusion)

The article utilized DISCERN to evaluate video quality and give useful advice and suggestions for future video publication.

Table S40. Assessments of risk of bias using JBI tool

Author_Theresa Steeb et al._ Year____2022_____ Record Number____39_____

| | Yes | No | Unclear | Not applicable |
|---|---|---|---|---|
| 1. Were the criteria for inclusion in the sample clearly defined? | √ | □ | □ | □ |
| 2. Were the study subjects and the setting described in detail? | √ | □ | □ | □ |
| 3. Was the exposure measured in a valid and reliable way? | √ | □ | □ | □ |
| 4. Were objective, standard criteria used for measurement of the condition? | √ | □ | □ | □ |
| 5. Were confounding factors identified? | √ | □ | □ | □ |
| 6. Were strategies to deal with confounding factors stated? | √ | □ | □ | □ |
| 7. Were the outcomes measured in a valid and reliable way? | √ | □ | □ | □ |
| 8. Was appropriate statistical analysis used? | √ | □ | □ | □ |

Overall appraisal: Include √ Exclude □ Seek further info □

Comments (Including reason for exclusion)

The article had clear logic and thorough statistical analysis for evaluation. Subtitle helped readers understand and catch the content.

Table S41. Assessments of risk of bias using JBI tool

Author__Dhruvil Radadiya et al._ Year____2020_____ Record Number____40_____

| | Yes | No | Unclear | Not applicable |
|---|---|---|---|---|
| 1. Were the criteria for inclusion in the sample clearly defined? | √ | □ | □ | □ |
| 2. Were the study subjects and the setting described in detail? | √ | □ | □ | □ |
| 3. Was the exposure measured in a valid and reliable way? | √ | □ | □ | □ |
| 4. Were objective, standard criteria used for measurement of the condition? | √ | □ | □ | □ |
| 5. Were confounding factors identified? | √ | □ | □ | □ |
| 6. Were strategies to deal with confounding factors stated? | √ | □ | □ | □ |
| 7. Were the outcomes measured in a valid and reliable way? | √ | □ | □ | □ |
| 8. Was appropriate statistical analysis used? | √ | □ | □ | □ |

Overall appraisal: Include √ Exclude □ Seek further info □

Comments (Including reason for exclusion)

The article used a very robust methodology C-DQS scoring to validate the video quality. And reviewer agreement was validated statistically.

Table S42. Assessments of risk of bias using JBI tool

Author_Nina Morena et al._Year____2023_____ Record Number____41_____

| | Yes | No | Unclear | Not applicable |
|---|---|---|---|---|
| 1. Were the criteria for inclusion in the sample clearly defined? | √ | □ | □ | □ |
| 2. Were the study subjects and the setting described in detail? | √ | □ | □ | □ |
| 3. Was the exposure measured in a valid and reliable way? | √ | □ | □ | □ |
| 4. Were objective, standard criteria used for measurement of the condition? | √ | □ | □ | □ |
| 5. Were confounding factors identified? | □ | √ | □ | □ |
| 6. Were strategies to deal with confounding factors stated? | □ | √ | □ | □ |
| 7. Were the outcomes measured in a valid and reliable way? | √ | □ | □ | □ |
| 8. Was appropriate statistical analysis used? | √ | □ | □ | □ |

Overall appraisal: Include √ Exclude □ Seek further info □

Comments (Including reason for exclusion)

The article had focused topic and use different tools to compare. The outcomes were reliable.

Table S43. Assessments of risk of bias using JBI tool

Author_Stacy Loeb et al. Year____2018_____ Record Number____42_____

| | Yes | No | Unclear | Not applicable |
|---|---|---|---|---|
| 1. Were the criteria for inclusion in the sample clearly defined? | √ | □ | □ | □ |
| 2. Were the study subjects and the setting described in detail? | √ | □ | □ | □ |
| 3. Was the exposure measured in a valid and reliable way? | □ | √ | □ | □ |
| 4. Were objective, standard criteria used for measurement of the condition? | √ | □ | □ | □ |
| 5. Were confounding factors identified? | □ | √ | □ | □ |
| 6. Were strategies to deal with confounding factors stated? | □ | √ | □ | □ |
| 7. Were the outcomes measured in a valid and reliable way? | √ | □ | □ | □ |
| 8. Was appropriate statistical analysis used? | √ | □ | □ | □ |

Overall appraisal: Include √ Exclude □ Seek further info □

Comments (Including reason for exclusion)

Though the study didn't mention limitation and generalization, the study utilized validated tools to evaluate video quality.________________________________________________

Table S44. Assessments of risk of bias using JBI tool

Author_Osman S€utc€uo glu et al._Year____2023_____ Record Number____43_____

| | Yes | No | Unclear | Not applicable |
|---|---|---|---|---|
| 1. Were the criteria for inclusion in the sample clearly defined? | √ | □ | □ | □ |
| 2. Were the study subjects and the setting described in detail? | √ | □ | □ | □ |
| 3. Was the exposure measured in a valid and reliable way? | √ | □ | □ | □ |
| 4. Were objective, standard criteria used for measurement of the condition? | √ | □ | □ | □ |
| 5. Were confounding factors identified? | √ | □ | □ | □ |
| 6. Were strategies to deal with confounding factors stated? | √ | □ | □ | □ |
| 7. Were the outcomes measured in a valid and reliable way? | √ | □ | □ | □ |
| 8. Was appropriate statistical analysis used? | √ | □ | □ | □ |

Overall appraisal: Include √ Exclude □ Seek further info □

Comments (Including reason for exclusion)

The article used appropriate statistical approaches and have excellent attention to scientific
accuracy.___________________________________________________________________

Table S45. Assessments of risk of bias using JBI tool

Author__Alexander J. Didier et al._ Year____2023_____ Record Number____44____

| | Yes | No | Unclear | Not applicable |
|---|---|---|---|---|
| 1. Were the criteria for inclusion in the sample clearly defined? | √ | □ | □ | □ |
| 2. Were the study subjects and the setting described in detail? | √ | □ | □ | □ |
| 3. Was the exposure measured in a valid and reliable way? | √ | □ | □ | □ |
| 4. Were objective, standard criteria used for measurement of the condition? | √ | □ | □ | □ |
| 5. Were confounding factors identified? | √ | □ | □ | □ |
| 6. Were strategies to deal with confounding factors stated? | √ | □ | □ | □ |
| 7. Were the outcomes measured in a valid and reliable way? | √ | □ | □ | □ |
| 8. Was appropriate statistical analysis used? | √ | □ | □ | □ |

Overall appraisal: Include √ Exclude □ Seek further info □

Comments (Including reason for exclusion)

The article had thorough and comprehensive analysis process. Limited sample size might affect the generalization of the result.

Table S46. Assessments of risk of bias using JBI tool

Author_Kuntay Kaplan et al._ Year____2022_____ Record Number____45_____

| | Yes | No | Unclear | Not applicable |
|---|---|---|---|---|
| 1. Were the criteria for inclusion in the sample clearly defined? | √ | □ | □ | □ |
| 2. Were the study subjects and the setting described in detail? | √ | □ | □ | □ |
| 3. Was the exposure measured in a valid and reliable way? | √ | □ | □ | □ |
| 4. Were objective, standard criteria used for measurement of the condition? | √ | □ | □ | □ |
| 5. Were confounding factors identified? | √ | □ | □ | □ |
| 6. Were strategies to deal with confounding factors stated? | √ | □ | □ | □ |
| 7. Were the outcomes measured in a valid and reliable way? | √ | □ | □ | □ |
| 8. Was appropriate statistical analysis used? | √ | □ | □ | □ |

Overall appraisal: Include √ Exclude □ Seek further info □

Comments (Including reason for exclusion)

The article well conducted video content assessment but was limited by single-language analysis in a non-English country.

Table S47. Assessments of risk of bias using JBI tool

Author_Francesco Di Bello et al._Year____2022_____ Record Number____46_____

| | Yes | No | Unclear | Not applicable |
|---|---|---|---|---|
| 1. Were the criteria for inclusion in the sample clearly defined? | √ | □ | □ | □ |
| 2. Were the study subjects and the setting described in detail? | √ | □ | □ | □ |
| 3. Was the exposure measured in a valid and reliable way? | √ | □ | □ | □ |
| 4. Were objective, standard criteria used for measurement of the condition? | √ | □ | □ | □ |
| 5. Were confounding factors identified? | √ | □ | □ | □ |
| 6. Were strategies to deal with confounding factors stated? | √ | □ | □ | □ |
| 7. Were the outcomes measured in a valid and reliable way? | √ | □ | □ | □ |
| 8. Was appropriate statistical analysis used? | √ | □ | □ | □ |

Overall appraisal: Include √ Exclude □ Seek further info □

Comments (Including reason for exclusion)

The article has large sample size to support the analysis and conclusion. Appropriate categorization of video sources and topics were discussed.___________________________________________________________

Table S48. Assessments of risk of bias using JBI tool

Author__Yeliz Bahar-Ozdemir et al._ Year__2022___ Record Number____47_____

| | Yes | No | Unclear | Not applicable |
|---|---|---|---|---|
| 1. Were the criteria for inclusion in the sample clearly defined? | √ | □ | □ | □ |
| 2. Were the study subjects and the setting described in detail? | √ | □ | □ | □ |
| 3. Was the exposure measured in a valid and reliable way? | √ | □ | □ | □ |
| 4. Were objective, standard criteria used for measurement of the condition? | √ | □ | □ | □ |
| 5. Were confounding factors identified? | □ | √ | □ | □ |
| 6. Were strategies to deal with confounding factors stated? | □ | √ | □ | □ |
| 7. Were the outcomes measured in a valid and reliable way? | √ | □ | □ | □ |
| 8. Was appropriate statistical analysis used? | √ | □ | □ | □ |

Overall appraisal: Include √ Exclude □ Seek further info □

Comments (Including reason for exclusion)

The article had well-structured analysis process and strong exposure and outcome validity.

Table S49. Assessments of risk of bias using JBI tool

Author_Benedetta Muzii et al._ Year____2023_____ Record Number____48_____

| | Yes | No | Unclear | Not applicable |
|---|---|---|---|---|
| 1. Were the criteria for inclusion in the sample clearly defined? | √ | □ | □ | □ |
| 2. Were the study subjects and the setting described in detail? | √ | □ | □ | □ |
| 3. Was the exposure measured in a valid and reliable way? | √ | □ | □ | □ |
| 4. Were objective, standard criteria used for measurement of the condition? | √ | □ | □ | □ |
| 5. Were confounding factors identified? | √ | □ | □ | □ |
| 6. Were strategies to deal with confounding factors stated? | √ | □ | □ | □ |
| 7. Were the outcomes measured in a valid and reliable way? | √ | □ | □ | □ |
| 8. Was appropriate statistical analysis used? | √ | □ | □ | □ |

Overall appraisal: Include √ Exclude □ Seek further info □

Comments (Including reason for exclusion)

The article defined clear video classification, and the outcomes were robust for video assessment
purposes.

Table S50. Assessments of risk of bias using JBI tool

Author_Manolis Pratsinis et al. Year__2021___ Record Number____49_____

| | Yes | No | Unclear | Not applicable |
|---|---|---|---|---|
| 1. Were the criteria for inclusion in the sample clearly defined? | √ | □ | □ | □ |
| 2. Were the study subjects and the setting described in detail? | √ | □ | □ | □ |
| 3. Was the exposure measured in a valid and reliable way? | √ | □ | □ | □ |
| 4. Were objective, standard criteria used for measurement of the condition? | √ | □ | □ | □ |
| 5. Were confounding factors identified? | √ | □ | □ | □ |
| 6. Were strategies to deal with confounding factors stated? | √ | □ | □ | □ |
| 7. Were the outcomes measured in a valid and reliable way? | √ | □ | □ | □ |
| 8. Was appropriate statistical analysis used? | √ | □ | □ | □ |

Overall appraisal: Include √ Exclude □ Seek further info □

Comments (Including reason for exclusion)

The article evaluated video quality for different languages, making results more thorough and general.___________________________________________________________

Table S51. Assessments of risk of bias using JBI tool

Author__Mesut Berkan Duran et al._ Year__2021___ Record Number____50_____

| | Yes | No | Unclear | Not applicable |
|---|---|---|---|---|
| 1. Were the criteria for inclusion in the sample clearly defined? | √ | □ | □ | □ |
| 2. Were the study subjects and the setting described in detail? | √ | □ | □ | □ |
| 3. Was the exposure measured in a valid and reliable way? | √ | □ | □ | □ |
| 4. Were objective, standard criteria used for measurement of the condition? | √ | □ | □ | □ |
| 5. Were confounding factors identified? | √ | □ | □ | □ |
| 6. Were strategies to deal with confounding factors stated? | √ | □ | □ | □ |
| 7. Were the outcomes measured in a valid and reliable way? | √ | □ | □ | □ |
| 8. Was appropriate statistical analysis used? | √ | □ | □ | □ |

Overall appraisal: Include √ Exclude □ Seek further info □

Comments (Including reason for exclusion)

The article used three validated tools to evaluate video quality and reliability and also study their correlation, which made results more reliable.

Table S52. Assessments of risk of bias using JBI tool

Author__Laith Baqain et al._Year____2023_____ Record Number____51_____

| | Yes | No | Unclear | Not applicable |
|---|---|---|---|---|
| 1. Were the criteria for inclusion in the sample clearly defined? | √ | □ | □ | □ |
| 2. Were the study subjects and the setting described in detail? | √ | □ | □ | □ |
| 3. Was the exposure measured in a valid and reliable way? | √ | □ | □ | □ |
| 4. Were objective, standard criteria used for measurement of the condition? | √ | □ | □ | □ |
| 5. Were confounding factors identified? | √ | □ | □ | □ |
| 6. Were strategies to deal with confounding factors stated? | √ | □ | □ | □ |
| 7. Were the outcomes measured in a valid and reliable way? | √ | □ | □ | □ |
| 8. Was appropriate statistical analysis used? | √ | □ | □ | □ |

Overall appraisal: Include √ Exclude □ Seek further info □

Comments (Including reason for exclusion)

The article properly characterized videos into several groups, then utilized validated tools to evaluate quality and reliability of videos.

Table S53. Assessments of risk of bias using JBI tool

Author__Stacy Loeb et al. Year____2021_____ Record Number____52_____

| | Yes | No | Unclear | Not applicable |
|---|---|---|---|---|
| 1. Were the criteria for inclusion in the sample clearly defined? | √ | □ | □ | □ |
| 2. Were the study subjects and the setting described in detail? | √ | □ | □ | □ |
| 3. Was the exposure measured in a valid and reliable way? | □ | √ | □ | □ |
| 4. Were objective, standard criteria used for measurement of the condition? | √ | □ | □ | □ |
| 5. Were confounding factors identified? | □ | √ | □ | □ |
| 6. Were strategies to deal with confounding factors stated? | □ | √ | □ | □ |
| 7. Were the outcomes measured in a valid and reliable way? | √ | □ | □ | □ |
| 8. Was appropriate statistical analysis used? | √ | □ | □ | □ |

Overall appraisal: Include √ Exclude □ Seek further info □

Comments (Including reason for exclusion)

Although the article was short and didn't include every detail, the logic is straightforward and the results were reliable.________________________________________________________________

Table S54. Assessments of risk of bias using JBI tool

Author_Alba Maria García-Cano-Fernández1 et al. Year_2020_ Record Number_53_

| | Yes | No | Unclear | Not applicable |
|---|---|---|---|---|
| 1. Were the criteria for inclusion in the sample clearly defined? | √ | □ | □ | □ |
| 2. Were the study subjects and the setting described in detail? | √ | □ | □ | □ |
| 3. Was the exposure measured in a valid and reliable way? | √ | □ | □ | □ |
| 4. Were objective, standard criteria used for measurement of the condition? | √ | □ | □ | □ |
| 5. Were confounding factors identified? | √ | □ | □ | □ |
| 6. Were strategies to deal with confounding factors stated? | √ | □ | □ | □ |
| 7. Were the outcomes measured in a valid and reliable way? | √ | □ | □ | □ |
| 8. Was appropriate statistical analysis used? | √ | □ | □ | □ |

Overall appraisal: Include √ Exclude □ Seek further info □

Comments (Including reason for exclusion)

Although the analysis process was relatively simple, the logic was straightforward. And the writing flow was so clear.

Table S55. Assessments of risk of bias using JBI tool

Author_Mehmet Akif Aydin et al._ Year____2020_____ Record Number____54_____

| | Yes | No | Unclear | Not applicable |
|---|---|---|---|---|
| 1. Were the criteria for inclusion in the sample clearly defined? | √ | □ | □ | □ |
| 2. Were the study subjects and the setting described in detail? | √ | □ | □ | □ |
| 3. Was the exposure measured in a valid and reliable way? | √ | □ | □ | □ |
| 4. Were objective, standard criteria used for measurement of the condition? | √ | □ | □ | □ |
| 5. Were confounding factors identified? | □ | √ | □ | □ |
| 6. Were strategies to deal with confounding factors stated? | □ | √ | □ | □ |
| 7. Were the outcomes measured in a valid and reliable way? | √ | □ | □ | □ |
| 8. Was appropriate statistical analysis used? | √ | □ | □ | □ |

Overall appraisal: Include √ Exclude □ Seek further info □

Comments (Including reason for exclusion)

The article was written logically with subtitle, which is clear for audience to catch the main points. The discussion was insightful.____________________________________________

Table S56. Assessments of risk of bias using JBI tool

Author_Paulina_ S ledzi nska et al. Year__2021___ Record Number____55_____

| | Yes | No | Unclear | Not applicable |
|---|---|---|---|---|
| 1. Were the criteria for inclusion in the sample clearly defined? | √ | □ | □ | □ |
| 2. Were the study subjects and the setting described in detail? | √ | □ | □ | □ |
| 3. Was the exposure measured in a valid and reliable way? | √ | □ | □ | □ |
| 4. Were objective, standard criteria used for measurement of the condition? | √ | □ | □ | □ |
| 5. Were confounding factors identified? | √ | □ | □ | □ |
| 6. Were strategies to deal with confounding factors stated? | √ | □ | □ | □ |
| 7. Were the outcomes measured in a valid and reliable way? | √ | □ | □ | □ |
| 8. Was appropriate statistical analysis used? | √ | □ | □ | □ |

Overall appraisal: Include √ Exclude □ Seek further info □

Comments (Including reason for exclusion)

The article had smooth and straight flow to evaluate video quality. The statistical methods were approapriate.

Table S57. Assessments of risk of bias using JBI tool

Author_Nicholas Shungu et al. Year__2021___ Record Number____56_____

| | Yes | No | Unclear | Not applicable |
|---|---|---|---|---|
| 1. Were the criteria for inclusion in the sample clearly defined? | √ | □ | □ | □ |
| 2. Were the study subjects and the setting described in detail? | √ | □ | □ | □ |
| 3. Was the exposure measured in a valid and reliable way? | √ | □ | □ | □ |
| 4. Were objective, standard criteria used for measurement of the condition? | √ | □ | □ | □ |
| 5. Were confounding factors identified? | √ | □ | □ | □ |
| 6. Were strategies to deal with confounding factors stated? | √ | □ | □ | □ |
| 7. Were the outcomes measured in a valid and reliable way? | √ | □ | □ | □ |
| 8. Was appropriate statistical analysis used? | √ | □ | □ | □ |

Overall appraisal: Include √ Exclude □ Seek further info □

Comments (Including reason for exclusion)

The article utilized validated tools to evaluate video quality for one of races, and also give comparison for other races.

Table S58. Assessments of risk of bias using JBI tool

Author_Alvaro Manuel Rodriguez Rodriguez et al. Year_2021_ Record Number_57_

| | Yes | No | Unclear | Not applicable |
|---|---|---|---|---|
| 1. Were the criteria for inclusion in the sample clearly defined? | √ | □ | □ | □ |
| 2. Were the study subjects and the setting described in detail? | √ | □ | □ | □ |
| 3. Was the exposure measured in a valid and reliable way? | √ | □ | □ | □ |
| 4. Were objective, standard criteria used for measurement of the condition? | √ | □ | □ | □ |
| 5. Were confounding factors identified? | √ | □ | □ | □ |
| 6. Were strategies to deal with confounding factors stated? | √ | □ | □ | □ |
| 7. Were the outcomes measured in a valid and reliable way? | √ | □ | □ | □ |
| 8. Was appropriate statistical analysis used? | √ | □ | □ | □ |

Overall appraisal: Include √ Exclude □ Seek further info □

Comments (Including reason for exclusion)

The article utilized machine learning methods to characterize videos into two groups incorporating several scales, which made results more reliable.

Table S59. Assessments of risk of bias using JBI tool

Author_Lydia Reinhardt et al._Year__2023___ Record Number____58_____

| | Yes | No | Unclear | Not applicable |
|---|---|---|---|---|
| 1. Were the criteria for inclusion in the sample clearly defined? | √ | □ | □ | □ |
| 2. Were the study subjects and the setting described in detail? | √ | □ | □ | □ |
| 3. Was the exposure measured in a valid and reliable way? | √ | □ | □ | □ |
| 4. Were objective, standard criteria used for measurement of the condition? | √ | □ | □ | □ |
| 5. Were confounding factors identified? | √ | □ | □ | □ |
| 6. Were strategies to deal with confounding factors stated? | √ | □ | □ | □ |
| 7. Were the outcomes measured in a valid and reliable way? | √ | □ | □ | □ |
| 8. Was appropriate statistical analysis used? | √ | □ | □ | □ |

Overall appraisal: Include √ Exclude □ Seek further info □

Comments (Including reason for exclusion)

The author utilized validated tools PEMAT/DISCERN to evaluate quality of videos. And it also had clear categorization of results.___________________________________________

Table S60. Assessments of risk of bias using JBI tool

Author_Erwin Vu et al.___Year____2021_____ Record Number____59_____

| | Yes | No | Unclear | Not applicable |
|---|---|---|---|---|
| 1. Were the criteria for inclusion in the sample clearly defined? | √ | □ | □ | □ |
| 2. Were the study subjects and the setting described in detail? | √ | □ | □ | □ |
| 3. Was the exposure measured in a valid and reliable way? | √ | □ | □ | □ |
| 4. Were objective, standard criteria used for measurement of the condition? | √ | □ | □ | □ |
| 5. Were confounding factors identified? | √ | □ | □ | □ |
| 6. Were strategies to deal with confounding factors stated? | √ | □ | □ | □ |
| 7. Were the outcomes measured in a valid and reliable way? | √ | □ | □ | □ |
| 8. Was appropriate statistical analysis used? | √ | □ | □ | □ |

Overall appraisal: Include √ Exclude □ Seek further info □

Comments (Including reason for exclusion)

The article clearly compare between surgery and radiotherapy by using different validated tools.

Table S61. Assessments of risk of bias using JBI tool

Author_Sergio Segado-Fernández et al._Year__2023__ Record Number___60_____

| | Yes | No | Unclear | Not applicable |
|---|---|---|---|---|
| 1. Were the criteria for inclusion in the sample clearly defined? | √ | □ | □ | □ |
| 2. Were the study subjects and the setting described in detail? | √ | □ | □ | □ |
| 3. Was the exposure measured in a valid and reliable way? | √ | □ | □ | □ |
| 4. Were objective, standard criteria used for measurement of the condition? | √ | □ | □ | □ |
| 5. Were confounding factors identified? | √ | □ | □ | □ |
| 6. Were strategies to deal with confounding factors stated? | √ | □ | □ | □ |
| 7. Were the outcomes measured in a valid and reliable way? | √ | □ | □ | □ |
| 8. Was appropriate statistical analysis used? | √ | □ | □ | □ |

Overall appraisal: Include √ Exclude □ Seek further info □

Comments (Including reason for exclusion)

The article has clear methods definition and well-categorized uploader types. The discussion and conclusions are thorough and insightful.

Table S62. Assessments of risk of bias using JBI tool

Author_Francesco Di Bello et al. Year__2022___ Record Number____61_____

| | Yes | No | Unclear | Not applicable |
|---|---|---|---|---|
| 1. Were the criteria for inclusion in the sample clearly defined? | √ | □ | □ | □ |
| 2. Were the study subjects and the setting described in detail? | √ | □ | □ | □ |
| 3. Was the exposure measured in a valid and reliable way? | √ | □ | □ | □ |
| 4. Were objective, standard criteria used for measurement of the condition? | √ | □ | □ | □ |
| 5. Were confounding factors identified? | □ | √ | □ | □ |
| 6. Were strategies to deal with confounding factors stated? | □ | √ | □ | □ |
| 7. Were the outcomes measured in a valid and reliable way? | √ | □ | □ | □ |
| 8. Was appropriate statistical analysis used? | √ | □ | □ | □ |

Overall appraisal: Include √ Exclude □ Seek further info □

Comments (Including reason for exclusion)

The article used a smooth flow to evaluate the video quality. The statistical methods were appropriate.

Table S63. Assessments of risk of bias using JBI tool

Author_Andrina Mamo1 et al._ Year__2021___ Record Number____62_____

| | Yes | No | Unclear | Not applicable |
|---|---|---|---|---|
| 1. Were the criteria for inclusion in the sample clearly defined? | □ | √ | □ | □ |
| 2. Were the study subjects and the setting described in detail? | √ | □ | □ | □ |
| 3. Was the exposure measured in a valid and reliable way? | □ | √ | □ | □ |
| 4. Were objective, standard criteria used for measurement of the condition? | √ | □ | □ | □ |
| 5. Were confounding factors identified? | □ | √ | □ | □ |
| 6. Were strategies to deal with confounding factors stated? | □ | √ | □ | □ |
| 7. Were the outcomes measured in a valid and reliable way? | √ | □ | □ | □ |
| 8. Was appropriate statistical analysis used? | √ | □ | □ | □ |

Overall appraisal: Include √ Exclude □ Seek further info □

Comments (Including reason for exclusion)

Although the methodololy is proper for assessment the information accuracy, the sample size is too limited. And it didn't mention the limitation in the article. There should be more description of videos to let audience know more information.

Table S64. Assessments of risk of bias using JBI tool

Author_Hanieh Meteran et al._ Year__2023___ Record Number____63_____

| | Yes | No | Unclear | Not applicable |
|---|---|---|---|---|
| 1. Were the criteria for inclusion in the sample clearly defined? | √ | □ | □ | □ |
| 2. Were the study subjects and the setting described in detail? | √ | □ | □ | □ |
| 3. Was the exposure measured in a valid and reliable way? | √ | □ | □ | □ |
| 4. Were objective, standard criteria used for measurement of the condition? | √ | □ | □ | □ |
| 5. Were confounding factors identified? | √ | □ | □ | □ |
| 6. Were strategies to deal with confounding factors stated? | √ | □ | □ | □ |
| 7. Were the outcomes measured in a valid and reliable way? | √ | □ | □ | □ |
| 8. Was appropriate statistical analysis used? | √ | □ | □ | □ |

Overall appraisal: Include √ Exclude □ Seek further info □

Comments (Including reason for exclusion)

The article had robust design for video quality evaluation. If using some validated toolds like DISCERN might be more efficient to explain results.

Table S65. Assessments of risk of bias using JBI tool

Author_Hüseyin Şan et al.__Year____2021_____ Record Number____64_____

| | Yes | No | Unclear | Not applicable |
|---|---|---|---|---|
| 1. Were the criteria for inclusion in the sample clearly defined? | √ | □ | □ | □ |
| 2. Were the study subjects and the setting described in detail? | √ | □ | □ | □ |
| 3. Was the exposure measured in a valid and reliable way? | √ | □ | □ | □ |
| 4. Were objective, standard criteria used for measurement of the condition? | √ | □ | □ | □ |
| 5. Were confounding factors identified? | √ | □ | □ | □ |
| 6. Were strategies to deal with confounding factors stated? | √ | □ | □ | □ |
| 7. Were the outcomes measured in a valid and reliable way? | √ | □ | □ | □ |
| 8. Was appropriate statistical analysis used? | √ | □ | □ | □ |

Overall appraisal: Include √ Exclude □ Seek further info □

Comments (Including reason for exclusion)

The study well defined many proper continuous variables and utilized many validation tools to evaluate the videos.______________________________________________________________

Table S66. Assessments of risk of bias using JBI tool

Author_Linda Ruppert et al._Year____2017_____ Record Number____65_____

| | Yes | No | Unclear | Not applicable |
|---|---|---|---|---|
| 1. Were the criteria for inclusion in the sample clearly defined? | √ | □ | □ | □ |
| 2. Were the study subjects and the setting described in detail? | √ | □ | □ | □ |
| 3. Was the exposure measured in a valid and reliable way? | √ | □ | □ | □ |
| 4. Were objective, standard criteria used for measurement of the condition? | √ | □ | □ | □ |
| 5. Were confounding factors identified? | □ | √ | □ | □ |
| 6. Were strategies to deal with confounding factors stated? | □ | √ | □ | □ |
| 7. Were the outcomes measured in a valid and reliable way? | √ | □ | □ | □ |
| 8. Was appropriate statistical analysis used? | √ | □ | □ | □ |

Overall appraisal: Include √ Exclude □ Seek further info □

Comments (Including reason for exclusion)

The article developed assessing the quality of YouTube videos with health information content checklist, which was thorough for evaluation.

Table S67. Assessments of risk of bias using JBI tool

Author_Georges Ayoub et al._Year____2021_____ Record Number____66_____

| | Yes | No | Unclear | Not applicable |
|---|---|---|---|---|
| 1. Were the criteria for inclusion in the sample clearly defined? | √ | □ | □ | □ |
| 2. Were the study subjects and the setting described in detail? | √ | □ | □ | □ |
| 3. Was the exposure measured in a valid and reliable way? | √ | □ | □ | □ |
| 4. Were objective, standard criteria used for measurement of the condition? | √ | □ | □ | □ |
| 5. Were confounding factors identified? | √ | □ | □ | □ |
| 6. Were strategies to deal with confounding factors stated? | √ | □ | □ | □ |
| 7. Were the outcomes measured in a valid and reliable way? | √ | □ | □ | □ |
| 8. Was appropriate statistical analysis used? | √ | □ | □ | □ |

Overall appraisal: Include √ Exclude □ Seek further info □

Comments (Including reason for exclusion)

The flow of this article is smooth and clear. Different validated tools were properly used to analyse collected video data.

Table S68. Assessments of risk of bias using JBI tool

Author_Tove Godskesen et al. Year____2021_____ Record Number____67_____

| | Yes | No | Unclear | Not applicable |
|---|---|---|---|---|
| 1. Were the criteria for inclusion in the sample clearly defined? | √ | □ | □ | □ |
| 2. Were the study subjects and the setting described in detail? | √ | □ | □ | □ |
| 3. Was the exposure measured in a valid and reliable way? | √ | □ | □ | □ |
| 4. Were objective, standard criteria used for measurement of the condition? | √ | □ | □ | □ |
| 5. Were confounding factors identified? | □ | √ | □ | □ |
| 6. Were strategies to deal with confounding factors stated? | □ | √ | □ | □ |
| 7. Were the outcomes measured in a valid and reliable way? | √ | □ | □ | □ |
| 8. Was appropriate statistical analysis used? | √ | □ | □ | □ |

Overall appraisal: Include √ Exclude □ Seek further info □

Comments (Including reason for exclusion)

The flow of this article is clear and logical. Every content was used subsection to highlight.

Table S69. Assessments of risk of bias using JBI tool

Author Abdullah Evren Yetişir et al.___Year____2023_____ Record Number____68_____

| | Yes | No | Unclear | Not applicable |
|---|---|---|---|---|
| 1. Were the criteria for inclusion in the sample clearly defined? | √ | □ | □ | □ |
| 2. Were the study subjects and the setting described in detail? | √ | □ | □ | □ |
| 3. Was the exposure measured in a valid and reliable way? | √ | □ | □ | □ |
| 4. Were objective, standard criteria used for measurement of the condition? | √ | □ | □ | □ |
| 5. Were confounding factors identified? | √ | □ | □ | □ |
| 6. Were strategies to deal with confounding factors stated? | √ | □ | □ | □ |
| 7. Were the outcomes measured in a valid and reliable way? | √ | □ | □ | □ |
| 8. Was appropriate statistical analysis used? | √ | □ | □ | □ |

Overall appraisal: Include √ Exclude □ Seek further info □

Comments (Including reason for exclusion)

Although the article had short result text, it had thorough tables to show the results, which was straightforward.

Table S70. Assessments of risk of bias using JBI tool

Author_Michał Krakowiaket al._ Year__2020___ Record Number____69_____

| | Yes | No | Unclear | Not applicable |
|---|---|---|---|---|
| 1. Were the criteria for inclusion in the sample clearly defined? | √ | □ | □ | □ |
| 2. Were the study subjects and the setting described in detail? | √ | □ | □ | □ |
| 3. Was the exposure measured in a valid and reliable way? | √ | □ | □ | □ |
| 4. Were objective, standard criteria used for measurement of the condition? | √ | □ | □ | □ |
| 5. Were confounding factors identified? | □ | √ | □ | □ |
| 6. Were strategies to deal with confounding factors stated? | □ | √ | □ | □ |
| 7. Were the outcomes measured in a valid and reliable way? | √ | □ | □ | □ |
| 8. Was appropriate statistical analysis used? | √ | □ | □ | □ |

Overall appraisal: Include √ Exclude □ Seek further info □

Comments (Including reason for exclusion)

The article utilized validated tools to evaluate video quality.

Table S71. Assessments of risk of bias using JBI tool

Author_Elaine Wittenberg-Lyles et al.__Year__2014___ Record Number____70_____

| | Yes | No | Unclear | Not applicable |
|---|---|---|---|---|
| 1. Were the criteria for inclusion in the sample clearly defined? | √ | □ | □ | □ |
| 2. Were the study subjects and the setting described in detail? | √ | □ | □ | □ |
| 3. Was the exposure measured in a valid and reliable way? | √ | □ | □ | □ |
| 4. Were objective, standard criteria used for measurement of the condition? | √ | □ | □ | □ |
| 5. Were confounding factors identified? | □ | √ | □ | □ |
| 6. Were strategies to deal with confounding factors stated? | □ | √ | □ | □ |
| 7. Were the outcomes measured in a valid and reliable way? | √ | □ | □ | □ |
| 8. Was appropriate statistical analysis used? | √ | □ | □ | □ |

Overall appraisal: Include √ Exclude □ Seek further info □

Comments (Including reason for exclusion)

This study coded from several subtypes to thoroughly validate current YouTube videos and made the result more reliable.

Table S72. Assessments of risk of bias using JBI tool

Author_Necati Enver et al.__Year____2020_____ Record Number____71_____

| | Yes | No | Unclear | Not applicable |
|---|---|---|---|---|
| 1. Were the criteria for inclusion in the sample clearly defined? | √ | □ | □ | □ |
| 2. Were the study subjects and the setting described in detail? | √ | □ | □ | □ |
| 3. Was the exposure measured in a valid and reliable way? | □ | □ | √ | □ |
| 4. Were objective, standard criteria used for measurement of the condition? | √ | □ | □ | □ |
| 5. Were confounding factors identified? | □ | √ | □ | □ |
| 6. Were strategies to deal with confounding factors stated? | □ | √ | □ | □ |
| 7. Were the outcomes measured in a valid and reliable way? | √ | □ | □ | □ |
| 8. Was appropriate statistical analysis used? | √ | □ | □ | □ |

Overall appraisal: Include √ Exclude □ Seek further info □

Comments (Including reason for exclusion)

This study provided a rigorous evaluation of YouTube larynx cancer videos with inter-rater reliability. Different kinds of tools were utilized to make the inference.______________________________________________________________

Table S73. Assessments of risk of bias using JBI tool

Author_Selen Gülo  glu et al._ Year__2022___ Record Number____72_____

| | Yes | No | Unclear | Not applicable |
|---|---|---|---|---|
| 1. Were the criteria for inclusion in the sample clearly defined? | √ | □ | □ | □ |
| 2. Were the study subjects and the setting described in detail? | √ | □ | □ | □ |
| 3. Was the exposure measured in a valid and reliable way? | √ | □ | □ | □ |
| 4. Were objective, standard criteria used for measurement of the condition? | √ | □ | □ | □ |
| 5. Were confounding factors identified? | √ | □ | □ | □ |
| 6. Were strategies to deal with confounding factors stated? | √ | □ | □ | □ |
| 7. Were the outcomes measured in a valid and reliable way? | √ | □ | □ | □ |
| 8. Was appropriate statistical analysis used? | √ | □ | □ | □ |

Overall appraisal: Include √ Exclude □ Seek further info □

Comments (Including reason for exclusion)

The study showed positive attitude of YouTube videos using validated tools and classification
comparison.

Table S74. Assessments of risk of bias using JBI tool

Author_Alain Nathan Sahin et al._ Year____2019_____ Record Number____73_____

| | Yes | No | Unclear | Not applicable |
|---|---|---|---|---|
| 1. Were the criteria for inclusion in the sample clearly defined? | √ | □ | □ | □ |
| 2. Were the study subjects and the setting described in detail? | √ | □ | □ | □ |
| 3. Was the exposure measured in a valid and reliable way? | √ | □ | □ | □ |
| 4. Were objective, standard criteria used for measurement of the condition? | √ | □ | □ | □ |
| 5. Were confounding factors identified? | □ | √ | □ | □ |
| 6. Were strategies to deal with confounding factors stated? | □ | √ | □ | □ |
| 7. Were the outcomes measured in a valid and reliable way? | √ | □ | □ | □ |
| 8. Was appropriate statistical analysis used? | √ | □ | □ | □ |

Overall appraisal: Include √ Exclude □ Seek further info □

Comments (Including reason for exclusion)

Authors devised a critical appraisal tool with a list of criteria to assess the videos_specifically for Colorectal Cancer____________________________________________________________

Table S75. Assessments of risk of bias using JBI tool

Author__Travis Brachtenbacha et al._ Year__2020___ Record Number____74_____

| | Yes | No | Unclear | Not applicable |
|---|---|---|---|---|
| 1. Were the criteria for inclusion in the sample clearly defined? | √ | □ | □ | □ |
| 2. Were the study subjects and the setting described in detail? | √ | □ | □ | □ |
| 3. Was the exposure measured in a valid and reliable way? | √ | □ | □ | □ |
| 4. Were objective, standard criteria used for measurement of the condition? | √ | □ | □ | □ |
| 5. Were confounding factors identified? | √ | □ | □ | □ |
| 6. Were strategies to deal with confounding factors stated? | √ | □ | □ | □ |
| 7. Were the outcomes measured in a valid and reliable way? | √ | □ | □ | □ |
| 8. Was appropriate statistical analysis used? | √ | □ | □ | □ |

Overall appraisal: Include √ Exclude □ Seek further info □

Comments (Including reason for exclusion)

The authors constructed a novel scoring system and contains thorough information about the validation of Youtube videos and the score is continuous and can be applied to use t-test.

Table S76. Assessments of risk of bias using JBI tool

Author_Alex J. Xu et al._ Year__2022___ Record Number____75_____

| | Yes | No | Unclear | Not applicable |
|---|---|---|---|---|
| 1. Were the criteria for inclusion in the sample clearly defined? | √ | □ | □ | □ |
| 2. Were the study subjects and the setting described in detail? | □ | √ | □ | □ |
| 3. Was the exposure measured in a valid and reliable way? | √ | □ | □ | □ |
| 4. Were objective, standard criteria used for measurement of the condition? | □ | √ | □ | □ |
| 5. Were confounding factors identified? | □ | √ | □ | □ |
| 6. Were strategies to deal with confounding factors stated? | □ | √ | □ | □ |
| 7. Were the outcomes measured in a valid and reliable way? | √ | □ | □ | □ |
| 8. Was appropriate statistical analysis used? | √ | □ | □ | □ |

Overall appraisal: Include □ Exclude √ Seek further info □

Comments (Including reason for exclusion)

Although the paper used validated tools to analyse the collected data, variables listed in the articles are not clear to audience if they didn't read the previous studies. However, the main results showed in summarized figure is appealing and straightforward.

Table S77. Quality Evaluation for Included Studies Using STROBE

1.STROBE Statement—checklist of items that should be included in reports of observational studies

| | Item No. | Recommendation | Page No. | Relevant text from manuscript |
|---|---|---|---|---|
| **Title and abstract** | 1 | (*a*) Indicate the study's design with a commonly used term in the title or the abstract | NA | |
| | | (*b*) Provide in the abstract an informative and balanced summary of what was done and what was found | 1 | From Abstract Methods to Results: "Questions about… DISCERN score of 4" |
| **Introduction** | | | | |
| Background/rationale | 2 | Explain the scientific background and rationale for the investigation being reported | 1 | Introduction paragraph 1-2<br>"ChatGPT is a software… a risk of biased information has been observed" |
| Objectives | 3 | State specific objectives, including any prespecified hypotheses | 2 | Introduction paragraph 2<br>"Our objective was to assess the quality of the information provided by AI like ChatGPT and establish if it is a reliable source of information for our patients." |
| **Methods** | | | | |
| Study design | 4 | Present key elements of study design early in the paper | NA | |
| Setting | 5 | Describe the setting, locations, and relevant dates, including periods of recruitment, exposure, follow-up, and data collection | 2 | Method paragraph 1<br>On March 18th, 2023, we formulated several questions in English to the latest version of ChatGPT 4.0 about the following urologic diseases |
| Participants | 6 | (*a*) *Cohort study*—Give the eligibility criteria, and the sources and methods of selection of participants. Describe methods of follow-up<br>*Case-control study*—Give the eligibility criteria, and the sources and methods of case ascertainment and control selection. Give the rationale for the choice of cases and controls | 2 | No patients were enrolled in our study |

| | | | | |
|---|---|---|---|---|
| | | *Cross-sectional study*—Give the eligibility criteria, and the sources and methods of selection of participants | | |
| | | (*b*) *Cohort study*—For matched studies, give matching criteria and number of exposed and unexposed<br><br>*Case-control study*—For matched studies, give matching criteria and the number of controls per case | | |
| Variables | 7 | Clearly define all outcomes, exposures, predictors, potential confounders, and effect modifiers. Give diagnostic criteria, if applicable | 2 | Method paragraph 1<br><br>Outcome is “We collected the answers… DISCERN quality questionnaire” |
| Data sources/ measurement | 8* | For each variable of interest, give sources of data and details of methods of assessment (measurement). Describe comparability of assessment methods if there is more than one group | 2 | Answers from ChatGPT 4.0 |
| Bias | 9 | Describe any efforts to address potential sources of bias | NA | |
| Study size | 10 | Explain how the study size was arrived at | NA | |

| | | | | |
|---|---|---|---|---|
| Quantitative variables | 11 | Explain how quantitative variables were handled in the analyses. If applicable, describe which groupings were chosen and why | 2 | DISCERN score (1-5 scale), word count, informed consent instrument |
| Statistical methods | 12 | (*a*) Describe all statistical methods, including those used to control for confounding | 2 | DISCERN and the informed consent instrument were obtained by performing a mean of both scores. |
| | | (*b*) Describe any methods used to examine subgroups and interactions | NA | |
| | | (*c*) Explain how missing data were addressed | NA | |
| | | (*d*) *Cohort study*—If applicable, explain how loss to follow-up was addressed<br><br>*Case-control study*—If applicable, explain how matching of cases and controls was addressed<br><br>*Cross-sectional study*—If applicable, describe analytical methods taking account of sampling strategy | NA | |
| | | (*e*) Describe any sensitivity analyses | NA | |

**Results**

| | | | | |
|---|---|---|---|---|
| Participants | 13* | (a) Report numbers of individuals at each stage of study—eg numbers potentially eligible, examined for eligibility, confirmed eligible, included in the study, completing follow-up, and analysed | NA | |
| | | (b) Give reasons for non-participation at each stage | NA | |
| | | (c) Consider use of a flow diagram | NA | |
| Descriptive data | 14* | (a) Give characteristics of study participants (eg demographic, clinical, social) and information on exposures and potential confounders | NA | |
| | | (b) Indicate number of participants with missing data for each variable of interest | NA | |
| | | (c) *Cohort study*—Summarise follow-up time (eg, average and total amount) | | |
| Outcome data | 15* | *Cohort study*—Report numbers of outcome events or summary measures over time | | |
| | | *Case-control study*—Report numbers in each exposure category, or summary measures of exposure | | |
| | | *Cross-sectional study*—Report numbers of outcome events or summary measures | 2 | Results paragraph 2-3 and table 1<br>"Prostate cancer is the only area with an error... DISCERN-16 scale" |
| Main results | 16 | (*a*) Give unadjusted estimates and, if applicable, confounder-adjusted estimates and their precision (eg, 95% confidence interval). Make clear which confounders were adjusted for and why they were included | NA | |
| | | (*b*) Report category boundaries when continuous variables were categorized | NA | |
| | | (*c*) If relevant, consider translating estimates of relative risk into absolute risk for a meaningful time period | NA | |

| | | | | |
|---|---|---|---|---|
| Other analyses | 17 | Report other analyses done—eg analyses of subgroups and interactions, and sensitivity analyses | NA | |
| **Discussion** | | | | |
| Key results | 18 | Summarise key results with reference to study objectives | 4 | Discussion paragraph 3<br>"Our study suggests that the quality of the … newer |

| | | | | |
|---|---|---|---|---|
| | | | | procedures may have lower-quality information” |
| Limitations | 19 | Discuss limitations of the study, taking into account sources of potential bias or imprecision. Discuss both direction and magnitude of any potential bias | 4 | Discussion last paragraph<br><br>“Our study has a few limitations…” |
| Interpretation | 20 | Give a cautious overall interpretation of results considering objectives, limitations, multiplicity of analyses, results from similar studies, and other relevant evidence | 4 | Discussion paragraph 2<br><br>ChatGPT has been trained using a dialogue model that encompasses all freely available information on the internet, including human conversations. |
| Generalisability | 21 | Discuss the generalisability (external validity) of the study results | 4 | Discussion paragraph 6<br><br>Although our study is limited to the patient's inquiries to ChatGPT, it could potentially be utilized to facilitate healthcare professionals' daily work and decision-making. |
| **Other information** | | | | |
| Funding | 22 | Give the source of funding and the role of the funders for the present study and, if applicable, for the original study on which the present article is based | 5 | All authors certify that they have no affiliations with or involvement in any organization or entity with any financial interest or non-financial interest in the subject matter or materials discussed in this manuscript. |

Table S78. Quality Evaluation for Included Studies Using STROBE

2.STROBE Statement—checklist of items that should be included in reports of observational studies

| | Item No. | Recommendation | Page No. | Relevant text from manuscript |
|---|---|---|---|---|
| **Title and abstract** | 1 | (*a*) Indicate the study's design with a commonly used term in the title or the abstract | NA | |
| | | (*b*) Provide in the abstract an informative and balanced summary of what was done and what was found | NA | No abstract |
| **Introduction** | | | | |
| Background/rationale | 2 | Explain the scientific background and rationale for the investigation being reported | 1 | "These properties can obscure that chatbots… amplification of misinformation" |
| Objectives | 3 | State specific objectives, including any prespecified hypotheses | 1 | We evaluated an LLM chatbot's performance to provide breast, prostate, and lung cancer treatment recommendations concordant with National Comprehensive Cancer Network (NCCN) guidelines. |
| **Methods** | | | | |
| Study design | 4 | Present key elements of study design early in the paper | 2 | "We developed 4zero-shot prompt templates… for a total of 104 prompts" |
| Setting | 5 | Describe the setting, locations, and relevant dates, including periods of recruitment, exposure, follow-up, and data collection | 2 | "Data were analyzed between March 2 and March 14, 2023"<br>"Prompts were input to the GPT-3.5-turbo-0301 model via the ChatGPT (OpenAI) interface" |
| Participants | 6 | (*a*) *Cohort study*—Give the eligibility criteria, and the sources and methods of selection of participants. Describe methods of follow-up<br>*Case-control study*—Give the eligibility criteria, and the sources and methods of case ascertainment and control selection. Give the rationale for the choice of cases and controls | 2 | institutional review board approval was not needed since human participants were not involved |

| | | | | |
|---|---|---|---|---|
| | | *Cross-sectional study*—Give the eligibility criteria, and the sources and methods of selection of participants | | |
| | | (*b*) *Cohort study*—For matched studies, give matching criteria and number of exposed and unexposed<br>*Case-control study*—For matched studies, give matching criteria and the number of controls per case | | |
| Variables | 7 | Clearly define all outcomes, exposures, predictors, potential confounders, and effect modifiers. Give diagnostic criteria, if applicable | 2 | Outcome: Five scoring criteria were developed to assess guideline concordance |
| Data sources/ measurement | 8* | For each variable of interest, give sources of data and details of methods of assessment (measurement). Describe comparability of assessment methods if there is more than one group | 2 | Prompts were input to the GPT-3.5-turbo- 0301 model via the ChatGPT (OpenAI) interface. |
| Bias | 9 | Describe any efforts to address potential sources of bias | 2 | Method paragraph 2<br>"The output did not have to recommend all possible… the oncologist who had not previously seen the output adjudicated " |
| Study size | 10 | Explain how the study size was arrived at | 2 | Templates were used to create 4 prompt variations for 26 diagnosis descriptions (cancer types with or without relevant extent of disease modifiers) for a total of 104 prompts. |

| | | | | |
|---|---|---|---|---|
| Quantitative variables | 11 | Explain how quantitative variables were handled in the analyses. If applicable, describe which groupings were chosen and why | 2 | Treatment provided, NCCN-concordant treatment, hallucinated treatment |
| Statistical methods | 12 | (*a*) Describe all statistical methods, including those used to control for confounding | 2 | majority rule was taken as the final score proportion of count |
| | | (*b*) Describe any methods used to examine subgroups and interactions | 2 | Table 1 |
| | | (*c*) Explain how missing data were addressed | NA | |

| | | | | |
|---|---|---|---|---|
| | | (*d*) *Cohort study*—If applicable, explain how loss to follow-up was addressed<br><br>*Case-control study*—If applicable, explain how matching of cases and controls was addressed<br><br>*Cross-sectional study*—If applicable, describe analytical methods taking account of sampling strategy | NA | |
| | | (*e*) Describe any sensitivity analyses | NA | |
| **Results** | | | | |
| Participants | 13* | (a) Report numbers of individuals at each stage of study—eg numbers potentially eligible, examined for eligibility, confirmed eligible, included in the study, completing follow-up, and analysed | NA | |
| | | (b) Give reasons for non-participation at each stage | | |
| | | (c) Consider use of a flow diagram | | |
| Descriptive data | 14* | (a) Give characteristics of study participants (eg demographic, clinical, social) and information on exposures and potential confounders | 2 | Results paragraph 1<br><br>“Outputs of 104 unique… was unclear” |
| | | (b) Indicate number of participants with missing data for each variable of interest | | |
| | | (c) *Cohort study*—Summarise follow-up time (eg, average and total amount) | | |
| Outcome data | 15* | *Cohort study*—Report numbers of outcome events or summary measures over time | *2* | *Results paragraph 1-2*<br><br>“Table 1 shows agreement… targeted therapy, or immunotherapy” |
| | | *Case-control study*—Report numbers in each exposure category, or summary measures of exposure | | |
| | | *Cross-sectional study*—Report numbers of outcome events or summary measures | | |
| Main results | 16 | (*a*) Give unadjusted estimates and, if applicable, confounder-adjusted estimates and their precision (eg, 95% confidence interval). Make clear which confounders were adjusted for and why they were included | 2 | *Results paragraph 1-2*<br><br>“Outputs of 104 unique… was unclear … targeted therapy, or immunotherapy” |
| | | (*b*) Report category boundaries when continuous variables were categorized | | |
| | | (*c*) If relevant, consider translating estimates of relative risk into absolute risk for a meaningful time period | | |

| | | | | |
|---|---|---|---|---|
| Other analyses | 17 | Report other analyses done—eg analyses of subgroups and interactions, and sensitivity analyses | 2 | Table1 by cancer type |
| **Discussion** | | | | |
| Key results | 18 | Summarise key results with reference to study objectives | 2 | One-third of treatments recommended by the chatbot were at least partially non concordant with NCCN guidelines; recommendations varied based on how the question was posed. |
| Limitations | 19 | Discuss limitations of the study, taking into account sources of potential bias or imprecision. Discuss both direction and magnitude of any potential bias | 3 | A study limitation is that… |
| Interpretation | 20 | Give a cautious overall interpretation of results considering objectives, limitations, multiplicity of analyses, results from similar studies, and other relevant evidence | 3 | "However, the chatbot did not perform well … difficult even for experts to detect." |
| Generalisability | 21 | Discuss the generalisability (external validity) of the study results | 3 | That we evaluated 1 model at a snapshot in time |
| **Other information** | | | | |
| Funding | 22 | Give the source of funding and the role of the funders for the present study and, if applicable, for the original study on which the present article is based | 4 | This work was supported by the Woods Foundation. |

Table S79. Quality Evaluation for Included Studies Using STROBE

3.STROBE Statement—checklist of items that should be included in reports of observational studies

| | Item No. | Recommendation | Page No. | Relevant text from manuscript |
|---|---|---|---|---|
| **Title and abstract** | 1 | (*a*) Indicate the study's design with a commonly used term in the title or the abstract | NA | |
| | | (*b*) Provide in the abstract an informative and balanced summary of what was done and what was found | 1 | Main outcomes and measures to Results |
| **Introduction** | | | | |
| Background/rationale | 2 | Explain the scientific background and rationale for the investigation being reported | 2 | Introduction paragraph 2-3<br>"Artificial intelligence chatbots are rapidly … may influence health-related behavior."<br>"However, the quality of AI chatbot responses to … yet been evaluated through validated instruments." |
| Objectives | 3 | State specific objectives, including any prespecified hypotheses | 2 | To this end, we assessed the quality of responses generated by AI chatbots to the most common search queries concerning the 5 most common cancers in the US |
| **Methods** | | | | |
| Study design | 4 | Present key elements of study design early in the paper | 2 | cross-sectional study was exempt from review and informed consent in accordance with the Common Rule given its use of publicly available data |
| Setting | 5 | Describe the setting, locations, and relevant dates, including periods of recruitment, exposure, follow-up, and data collection | 2 | The top Google (Alphabet, Inc) search queries related to lung, skin, colorectal, breast, and prostate cancers in the US |

| | | | | |
|---|---|---|---|---|
| | | | | From January 1, 2021, to January 1, 2023,were identified using Google Trends |
| Participants | 6 | (*a*) *Cohort study*—Give the eligibility criteria, and the sources and methods of selection of participants. Describe methods of follow-up<br>*Case-control study*—Give the eligibility criteria, and the sources and methods of case ascertainment and control selection. Give the rationale for the choice of cases and controls<br>*Cross-sectional study*—Give the eligibility criteria, and the sources and methods of selection of participants | NA | |
| | | (*b*) *Cohort study*—For matched studies, give matching criteria and number of exposed and unexposed<br>*Case-control study*—For matched studies, give matching criteria and the number of controls per case | | |
| Variables | 7 | Clearly define all outcomes, exposures, predictors, potential confounders, and effect modifiers. Give diagnostic criteria, if applicable | 2 | Outcome: DISCERN score, PEMAT understandability and actionability, 5-point Likert scale, Flesch-Kincaid Grade Level |
| Data sources/ measurement | 8* | For each variable of interest, give sources of data and details of methods of assessment (measurement). Describe comparability of assessment methods if there is more than one group | | The top Google (Alphabet, Inc) using Google Trends |
| Bias | 9 | Describe any efforts to address potential sources of bias | 2 | Methods paragraph 2<br>"The top 5 search queries… exact phrasing from Google Trends. " |
| Study size | 10 | Explain how the study size was arrived at | 2 | A total of 100 chatbot responses to the top 5 search queries for skin, colorectal, prostate, lung, or breast cancer were analyzed. |

| | | | | |
|---|---|---|---|---|
| Quantitative variables | 11 | Explain how quantitative variables were handled in the analyses. If applicable, describe which groupings were chosen and why | 2 | Methods paragraph 3<br>"*Each response was evaluated… Flesch-Kincaid* |

| | | | | |
|---|---|---|---|---|
| | | | | *Grade Level (range, 5 [easy to read] to 16 [most difficult to read]).*" |
| Statistical methods | 12 | (*a*) Describe all statistical methods, including those used to control for confounding | 2 | Excel for descriptive statistics |
| | | (*b*) Describe any methods used to examine subgroups and interactions | 2 | Method |
| | | (*c*) Explain how missing data were addressed | NA | |
| | | (*d*) *Cohort study*—If applicable, explain how loss to follow-up was addressed<br>*Case-control study*—If applicable, explain how matching of cases and controls was addressed<br>*Cross-sectional study*—If applicable, describe analytical methods taking account of sampling strategy | NA | |
| | | (*e*) Describe any sensitivity analyses | NA | |
| **Results** | | | | |
| Participants | 13* | (a) Report numbers of individuals at each stage of study—eg numbers potentially eligible, examined for eligibility, confirmed eligible, included in the study, completing follow-up, and analysed | NA | |
| | | (b) Give reasons for non-participation at each stage | | |
| | | (c) Consider use of a flow diagram | | |
| Descriptive data | 14* | (a) Give characteristics of study participants (eg demographic, clinical, social) and information on exposures and potential confounders | 2 | Results paragraph 2- and table<br>"*The 4 AI chatbots… college reading level (Table).*" |
| | | (b) Indicate number of participants with missing data for each variable of interest | | |
| | | (c) *Cohort study*—Summarise follow-up time (eg, average and total amount) | | |
| Outcome data | 15* | *Cohort study*—Report numbers of outcome events or summary measures over time | *2* | Results paragraph 2- and table<br>"*The 4 AI chatbots… college reading level (Table).*" |
| | | *Case-control study*—Report numbers in each exposure category, or summary measures of exposure | | |
| | | *Cross-sectional study*—Report numbers of outcome events or summary measures | | |

| | | | | |
|---|---|---|---|---|
| Main results | 16 | (*a*) Give unadjusted estimates and, if applicable, confounder-adjusted estimates and their precision (eg, 95% confidence interval). Make clear which confounders were adjusted for and why they were included | 2 | Results paragraph 2- and table "*The 4 AI chatbots… college reading level (Table).*" |
| | | (*b*) Report category boundaries when continuous variables were categorized | NA | |
| | | (*c*) If relevant, consider translating estimates of relative risk into absolute risk for a meaningful time period | NA | |
| Other analyses | 17 | Report other analyses done—eg analyses of subgroups and interactions, and sensitivity analyses | 2 | Compare with different cancer type and chatbot platform |
| **Discussion** | | | | |
| Key results | 18 | Summarise key results with reference to study objectives | 3 | Discussion paragraph 1 "According to DISCERN… cancer through social networks" |
| Limitations | 19 | Discuss limitations of the study, taking into account sources of potential bias or imprecision. Discuss both direction and magnitude of any potential bias | 3 | Strength and limitations paragraph 2 "This study was limited to queries… " |
| Interpretation | 20 | Give a cautious overall interpretation of results considering objectives, limitations, multiplicity of analyses, results from similar studies, and other relevant evidence | 3 | AI chatbots should be used supplementarily and not as a primary source for medical information. |
| Generalisability | 21 | Discuss the generalisability (external validity) of the study results | 3 | AI chatbots based on the most popular internet searches according to Google Trends because search trends of the AI chatbots themselves are not publicly available |
| **Other information** | | | | |
| Funding | 22 | Give the source of funding and the role of the funders for the present study and, if applicable, for the original study on which the present article is based | 4 | Conflict of Interest Disclosures… |

Table S80. Quality Evaluation for Included Studies Using STROBE

4.STROBE Statement—checklist of items that should be included in reports of observational studies

| | Item No. | Recommendation | Page No. | Relevant text from manuscript |
|---|---|---|---|---|
| **Title and abstract** | 1 | (*a*) Indicate the study's design with a commonly used term in the title or the abstract | NA | |
| | | (*b*) Provide in the abstract an informative and balanced summary of what was done and what was found | 1 | "We used the top five…for consumer health information." |
| **Introduction** | | | | |
| Background/rationale | 2 | Explain the scientific background and rationale for the investigation being reported | 1 | Paragraph 2<br>"With more than 175 million users, the AI chatbot ChatGPT is …provided by AI chatbots. " |
| Objectives | 3 | State specific objectives, including any prespecified hypotheses | 2 | to assess the responses produced by four AI chatbots for the top Google-searched medical queries related to the four urological malignancies with the highest incidence rates |
| **Methods** | | | | |
| Study design | 4 | Present key elements of study design early in the paper | 2 | Cross-sectional study to input, using the exact query phrasing |
| Setting | 5 | Describe the setting, locations, and relevant dates, including periods of recruitment, exposure, follow-up, and data collection | 2 | The top five Google search queries for each cancer were then input, using the exact query phrasing, into the latest publicly accessible version of four different AI chatbots on April 10, 2023: |
| Participants | 6 | (*a*) *Cohort study*—Give the eligibility criteria, and the sources and methods of selection of participants. Describe methods of follow-up<br>*Case-control study*—Give the eligibility criteria, and the sources and methods of case ascertainment and control selection. Give the rationale for the choice of cases and controls | 2 | We used Google Trends to identify the most popular search queries related to prostate, bladder, kidney, and testicular cancers in the USA from January 1, 2021 to January 1, |

| | | | | |
|---|---|---|---|---|
| | | *Cross-sectional study*—Give the eligibility criteria, and the sources and methods of selection of participants | | 2023 (Supplementary Table 1).<br>The top five Google search queries for each cancer were then input, using the exact query phrasing, into the latest publicly accessible version |
| | | (*b*) *Cohort study*—For matched studies, give matching criteria and number of exposed and unexposed<br>*Case-control study*—For matched studies, give matching criteria and the number of controls per case | | |
| Variables | 7 | Clearly define all outcomes, exposures, predictors, potential confounders, and effect modifiers. Give diagnostic criteria, if applicable | 2 | Outcome: *DISCERN, PEMAT-P, Flesch-Kincaid, word count, and Likert scores* |
| Data sources/ measurement | 8* | For each variable of interest, give sources of data and details of methods of assessment (measurement). Describe comparability of assessment methods if there is more than one group | *2* | *the latest publicly accessible version of four different AI chatbots on April 10, 2023: ChatGPT v3.5, Perplexity, Chat Sonic, and Microsoft Bing AI.* |
| Bias | 9 | Describe any efforts to address potential sources of bias | 2 | The settings used were default for ChatGPT, concise for Perplexity, Google-integrated concise for Chat Sonic, and balanced results for Microsoft Bing AI. The memory was cleared for all AI chatbots to prevent previous queries from affecting follow-up outputs. |
| Study size | 10 | Explain how the study size was arrived at | | 20: The top five Google search queries for each cancer were then input, using the exact query phrasing, into the latest publicly accessible version of four different AI chatbots |

| | | | | |
|---|---|---|---|---|
| Quantitative variables | 11 | Explain how quantitative variables were handled in the analyses. If applicable, describe which groupings were chosen and why | 2 | *DISCERN, PEMAT-P, Flesch-Kincaid, word count, and Likert scores* |
| Statistical methods | 12 | (*a*) Describe all statistical methods, including those used to control for confounding | 2 | *Descriptive statistics, including the median and range* |
| | | (*b*) Describe any methods used to examine subgroups and interactions | 3 | Table 1 |
| | | (*c*) Explain how missing data were addressed | NA | |
| | | (*d*) *Cohort study*—If applicable, explain how loss to follow-up was addressed<br>*Case-control study*—If applicable, explain how matching of cases and controls was addressed<br>*Cross-sectional study*—If applicable, describe analytical methods taking account of sampling strategy | NA | |
| | | (*e*) Describe any sensitivity analyses | NA | |
| **Results** | | | | |
| Participants | 13* | (a) Report numbers of individuals at each stage of study—eg numbers potentially eligible, examined for eligibility, confirmed eligible, included in the study, completing follow-up, and analysed | NA | |
| | | (b) Give reasons for non-participation at each stage | NA | |
| | | (c) Consider use of a flow diagram | NA | |
| Descriptive data | 14* | (a) Give characteristics of study participants (eg demographic, clinical, social) and information on exposures and potential confounders | 2 | Paragraph 5<br>"Among all the AI chatbot responses… some scores as low as 2." |
| | | (b) Indicate number of participants with missing data for each variable of interest | NA | |
| | | (c) *Cohort study*—Summarise follow-up time (eg, average and total amount) | NA | |
| Outcome data | 15* | *Cohort study*—Report numbers of outcome events or summary measures over time | | |
| | | *Case-control study*—Report numbers in each exposure category, or summary measures of exposure | | |
| | | *Cross-sectional study*—Report numbers of outcome events or summary measures | *2* | Paragraph 5 |

| | | | | |
|---|---|---|---|---|
| | | | | "Among all the AI chatbot responses… some scores as low as 2." |
| Main results | 16 | (*a*) Give unadjusted estimates and, if applicable, confounder-adjusted estimates and their precision (eg, 95% confidence interval). Make clear which confounders were adjusted for and why they were included | 2 | Across all four cancers, the quality of information was high (median DISCERN 4–5) and lacked misinformation (Likert score 1). |
| | | (*b*) Report category boundaries when continuous variables were categorized | NA | |
| | | (*c*) If relevant, consider translating estimates of relative risk into absolute risk for a meaningful time period | NA | |
| Other analyses | 17 | Report other analyses done—eg analyses of subgroups and interactions, and sensitivity analyses | 2 | Compare results by cancer type |
| **Discussion** | | | | |
| Key results | 18 | Summarise key results with reference to study objectives | 3 | Results for total DISCERN scores revealed moderate to high quality for consumer health information provided by the four AI chatbots. |
| Limitations | 19 | Discuss limitations of the study, taking into account sources of potential bias or imprecision. Discuss both direction and magnitude of any potential bias | 3 | Our study is limited by the use of query entries from top Google searches, as the search metrics for the AI chatbots are not publicly available. A mismatch in input phrasing is possible and may affect the quality of the output information. Future studies are warranted to evaluate if alternative responses from AI chatbots would impact scores. |
| Interpretation | 20 | Give a cautious overall interpretation of results considering objectives, limitations, multiplicity of analyses, results from similar studies, and other relevant evidence | 3 | AI chatbots are a more accurate source of medical information on urological malignancies in comparison to other online platforms such as TikTok, Instagram, and YouTube. The |

| | | | | |
|---|---|---|---|---|
| | | | | performance of AI chatbots could be further improved |
| Generalisability | 21 | Discuss the generalisability (external validity) of the study results | 3 | A mismatch in input phrasing is possible and may affect the quality of the output information. Future studies are warranted to evaluate if alternative responses from AI chatbots would impact scores. |
| **Other information** | | | | |
| Funding | 22 | Give the source of funding and the role of the funders for the present study and, if applicable, for the original study on which the present article is based | 4 | Stacy Loeb is supported by the National Cancer Institute |

Table S81. Quality Evaluation for Included Studies Using STROBE

5.STROBE Statement—checklist of items that should be included in reports of observational studies

| | Item No. | Recommendation | Page No. | Relevant text from manuscript |
|---|---|---|---|---|
| **Title and abstract** | 1 | (*a*) Indicate the study's design with a commonly used term in the title or the abstract | NA | |
| | | (*b*) Provide in the abstract an informative and balanced summary of what was done and what was found | 1 | *"We conducted a content analysis… 14%described unproven treatment modalities."* |
| **Introduction** | | | | |
| Background/rationale | 2 | Explain the scientific background and rationale for the investigation being reported | 2 | *"Patients and their caregivers are increasingly using social media as a way to share their illness experiences"*<br>*"Health professionals express mixed reactions to the increase in health communication on social media."* |
| Objectives | 3 | State specific objectives, including any prespecified hypotheses | 2 | *systematically characterizing (1) the types and (2) scientific accuracy of medically-oriented cancer information posted on personal Facebook Pages of cancer care givers.* |
| **Methods** | | | | |
| Study design | 4 | Present key elements of study design early in the paper | 2 | *Cross-sectional: each Page from May 2012 to May 2013 extracted a total of 15,852 unique posts* |
| Setting | 5 | Describe the setting, locations, and relevant dates, including periods of recruitment, exposure, follow-up, and data collection | 2 | *This process yielded 18 Facebook Pages for analysis.*<br>*We extracted the content from the information and wall* |

| | | | | |
|---|---|---|---|---|
| | | | | *sections of each Page from May 2012 to May 2013* |
| Participants | 6 | (*a*) *Cohort study*—Give the eligibility criteria, and the sources and methods of selection of participants. Describe methods of follow-up<br>*Case-control study*—Give the eligibility criteria, and the sources and methods of case ascertainment and control selection. Give the rationale for the choice of cases and controls<br>*Cross-sectional study*—Give the eligibility criteria, and the sources and methods of selection of participants | 2 | Methods paragraph 1<br>"Due to the intensive… *were comments from other Facebook users.*" |
| | | (*b*) *Cohort study*—For matched studies, give matching criteria and number of exposed and unexposed<br>*Case-control study*—For matched studies, give matching criteria and the number of controls per case | | |
| Variables | 7 | Clearly define all outcomes, exposures, predictors, potential confounders, and effect modifiers. Give diagnostic criteria, if applicable | 2 | Methods last paragraph<br>*"The two experts then independently… unproven treatment modalities."*<br>Findings paragraph 1<br>"T*he 25 codes were condensed… and (6) other*" |
| Data sources/ measurement | 8* | For each variable of interest, give sources of data and details of methods of assessment (measurement). Describe comparability of assessment methods if there is more than one group | *2* | *Posts and comments from the Facebook Pages.* |
| Bias | 9 | Describe any efforts to address potential sources of bias | 3 | *The oncology experts verified the medical and scientific accuracy of information using peer-reviewed publications and national clinical guidelines. After independent evaluation, the two oncology experts met to discuss and resolve any conflicts and achieve consensus on the final data analysis.* |
| Study size | 10 | Explain how the study size was arrived at | 2 | *We extracted a total of 15,852 unique posts and comments from the* |

| | | | | |
|---|---|---|---|---|
| | | | | *Facebook Pages. Of these posts, 2030 were posts by the Page administrators (the parent of the cancer patient) and 13,822 were comments from other Facebook users.* |
| Quantitative variables | 11 | Explain how quantitative variables were handled in the analyses. If applicable, describe which groupings were chosen and why | 3 | Findings paragraph 1<br><br>Proportion and frequency |
| Statistical methods | 12 | (*a*) Describe all statistical methods, including those used to control for confounding | 3 | Descriptive |
| | | (*b*) Describe any methods used to examine subgroups and interactions | 4 | Findings |
| | | (*c*) Explain how missing data were addressed | NA | |
| | | (*d*) *Cohort study*—If applicable, explain how loss to follow-up was addressed<br><br>*Case-control study*—If applicable, explain how matching of cases and controls was addressed<br><br>*Cross-sectional study*—If applicable, describe analytical methods taking account of sampling strategy | NA | |
| | | (*e*) Describe any sensitivity analyses | NA | |
| **Results** | | | | |
| Participants | 13* | (a) Report numbers of individuals at each stage of study—eg numbers potentially eligible, examined for eligibility, confirmed eligible, included in the study, completing follow-up, and analysed | 3 | "*Of the 15,852 total posts, 171 posts contained medically oriented cancer information. Twenty-five codes were identified to categorize the cancer information exchanged on cancer caregivers' personal Facebook Pages*" |
| | | (b) Give reasons for non-participation at each stage | 3 | Findings paragraph 1<br><br>"*Examples include it's not… treatment modalities (Table 3).* " |
| | | (c) Consider use of a flow diagram | | |
| Descriptive data | 14* | (a) Give characteristics of study participants (eg demographic, clinical, social) and information on exposures and potential confounders | 3 | Findings paragraph 1 |

| | | | | |
|---|---|---|---|---|
| | | | | "*The most frequent type of cancer… be medically/scientifically accurate*" |
| | | (b) Indicate number of participants with missing data for each variable of interest | | |
| | | (c) *Cohort study*—Summarise follow-up time (eg, average and total amount) | | |
| Outcome data | 15* | *Cohort study*—Report numbers of outcome events or summary measures over time | | |
| | | *Case-control study*—Report numbers in each exposure category, or summary measures of exposure | | |
| | | *Cross-sectional study*—Report numbers of outcome events or summary measures | *3* | *"Seventy-eight percent of information… unproven treatment modalities."* |
| Main results | 16 | (*a*) Give unadjusted estimates and, if applicable, confounder-adjusted estimates and their precision (eg, 95% confidence interval). Make clear which confounders were adjusted for and why they were included | 3 | *Overall, 67% of all cancer information exchanged was deemed to be medically/scientifically accurate.* |
| | | (*b*) Report category boundaries when continuous variables were categorized | NA | |
| | | (*c*) If relevant, consider translating estimates of relative risk into absolute risk for a meaningful time period | NA | |

| | | | | |
|---|---|---|---|---|
| Other analyses | 17 | Report other analyses done—eg analyses of subgroups and interactions, and sensitivity analyses | 3 | Findings paragraph 1<br><br>Subgroup into 6 category to get accuracy categories. "*As shown in Table 1…*" |
| **Discussion** | | | | |
| Key results | 18 | Summarise key results with reference to study objectives | 4 | "*Of the 15,852 total posts,… should be cautioned that some information shared on social media is incorrect."* |
| Limitations | 19 | Discuss limitations of the study, taking into account sources of potential bias or imprecision. Discuss both direction and magnitude of any potential bias | 4 | *Some data limitations should be noted when interpreting these findings…* |
| Interpretation | 20 | Give a cautious overall interpretation of results considering objectives, limitations, multiplicity of analyses, results from similar studies, and other relevant evidence | 4 | Discussion paragraph 2<br><br>"*Some data limitations should be noted when interpreting these findings. There may be* |

| | | | | |
|---|---|---|---|---|
| | | | | *differences in the types and scientific accuracy of cancer information users exchange…*” |
| Generalisability | 21 | Discuss the generalisability (external validity) of the study results | 4 | *“There may be differences in the types and scientific accuracy of cancer … cancer-related support*” |
| **Other information** | | | | |
| Funding | 22 | Give the source of funding and the role of the funders for the present study and, if applicable, for the original study on which the present article is based | 5 | *This work was supported by Roswell Park Cancer Institute and National Cancer Institute (NCI) grant* |

Table S82. Quality Evaluation for Included Studies Using STROBE

6.STROBE Statement—checklist of items that should be included in reports of observational studies

| | Item No. | Recommendation | Page No. | Relevant text from manuscript |
|---|---|---|---|---|
| **Title and abstract** | 1 | (*a*) Indicate the study's design with a commonly used term in the title or the abstract | NA | |
| | | (*b*) Provide in the abstract an informative and balanced summary of what was done and what was found | 1 | *Although the majority of messages included sharing personal experience and provided psychosocial support (50%), there were a significant number of posts offering medical advice (35%), with the majority of such replies being unsupported by evidence-based medicine (87%).* |
| **Introduction** | | | | |
| Background/rationale | 2 | Explain the scientific background and rationale for the investigation being reported | 1 | Introduction paragraph 1<br>"*With rising rates of social media…available live or on the Internet*" |
| Objectives | 3 | State specific objectives, including any prespecified hypotheses | 1 | *The goal of this study was to systematically review and categorize available patient-driven support and education resources focused on keratinocyte carcinoma on Facebook.* |
| **Methods** | | | | |
| Study design | 4 | Present key elements of study design early in the paper | 1 | *retrospective review* |
| Setting | 5 | Describe the setting, locations, and relevant dates, including periods of recruitment, exposure, follow-up, and data collection | 1 | *from 01/01/2018 to 06/30/2018, up to 500 consecutive posts in each group were analyzed by a single reviewer.* |
| Participants | 6 | (*a*) *Cohort study*—Give the eligibility criteria, and the sources and methods of selection of participants. Describe methods of follow-up | 1 | *Methods paragraph 1*<br>*"Facebook was searched with key terms, … meeting* |

| | | | | |
|---|---|---|---|---|
| | | *Case-control study*—Give the eligibility criteria, and the sources and methods of case ascertainment and control selection. Give the rationale for the choice of cases and controls<br><br>*Cross-sectional study*—Give the eligibility criteria, and the sources and methods of selection of participants | | *criteria, requests to join were granted by 7."* |
| | | (*b*) *Cohort study*—For matched studies, give matching criteria and number of exposed and unexposed<br><br>*Case-control study*—For matched studies, give matching criteria and the number of controls per case | | |
| Variables | 7 | Clearly define all outcomes, exposures, predictors, potential confounders, and effect modifiers. Give diagnostic criteria, if applicable | 2 | *Qualitative themes according to content as initial forum posts (e.g. sharing experiences, posting photos, seeking support, asking medical advice) and comments responding to posts (e.g. providing psychosocial support, offering treatment or diagnostic advice, advertising products* |
| Data sources/ measurement | 8* | For each variable of interest, give sources of data and details of methods of assessment (measurement). Describe comparability of assessment methods if there is more than one group | *1* | *Post from Facebook* |
| Bias | 9 | Describe any efforts to address potential sources of bias | 1 | *Qualitative themes according to content as initial forum posts (e.g. sharing experiences, posting photos, seeking support, asking medical advice) and comments responding to posts (e.g. providing psychosocial support, offering treatment or diagnostic advice, advertising products* |
| Study size | 10 | Explain how the study size was arrived at | 3 | *A total of 3,130 posts were catalogued with 444 posts* |

| | | | | |
|---|---|---|---|---|
| Quantitative variables | 11 | Explain how quantitative variables were handled in the analyses. If applicable, describe which groupings were chosen and why | 3 | The number and proportion of each theme and |
| Statistical methods | 12 | (*a*) Describe all statistical methods, including those used to control for confounding | 3 | Descriptive statistics |
| | | (*b*) Describe any methods used to examine subgroups and interactions | 3 | Table 1 |
| | | (*c*) Explain how missing data were addressed | NA | |
| | | (*d*) *Cohort study*—If applicable, explain how loss to follow-up was addressed<br>*Case-control study*—If applicable, explain how matching of cases and controls was addressed<br>*Cross-sectional study*—If applicable, describe analytical methods taking account of sampling strategy | NA | |
| | | (*e*) Describe any sensitivity analyses | NA | |
| **Results** | | | | |
| Participants | 13* | (a) Report numbers of individuals at each stage of study—eg numbers potentially eligible, examined for eligibility, confirmed eligible, included in the study, completing follow-up, and analysed | 3 | A total of 3,130 posts were catalogued with 444 posts initiating a conversation thread and an average of 6 reply comments |
| | | (b) Give reasons for non-participation at each stage | NA | |
| | | (c) Consider use of a flow diagram | NA | |
| Descriptive data | 14* | (a) Give characteristics of study participants (eg demographic, clinical, social) and information on exposures and potential confounders | 3 | Results paragraph 1<br>"Of these posts, 40%... based alternative therapies" |
| | | (b) Indicate number of participants with missing data for each variable of interest | NA | |
| | | (c) *Cohort study*—Summarise follow-up time (eg, average and total amount) | NA | |
| Outcome data | 15* | *Cohort study*—Report numbers of outcome events or summary measures over time | | |
| | | *Case-control study*—Report numbers in each exposure category, or summary measures of exposure | | |
| | | *Cross-sectional study*—Report numbers of outcome events or summary measures | *3* | Results paragraph 1<br>"Of these posts, 40%... based alternative therapies" |

| | | | | |
|---|---|---|---|---|
| Main results | 16 | (*a*) Give unadjusted estimates and, if applicable, confounder-adjusted estimates and their precision (eg, 95% confidence interval). Make clear which confounders were adjusted for and why they were included | 4 | Of the 35% of posts offering medical advice, only 13% included supported information from primary literature or medical personnel |
| | | (*b*) Report category boundaries when continuous variables were categorized | NA | |
| | | (*c*) If relevant, consider translating estimates of relative risk into absolute risk for a meaningful time period | NA | |
| Other analyses | 17 | Report other analyses done—eg analyses of subgroups and interactions, and sensitivity analyses | 3 | Image subtype from table 1 |
| **Discussion** | | | | |
| Key results | 18 | Summarise key results with reference to study objectives | 4 | Discussion paragraph 2<br>"Although images are an essential part of other social … from primary literature or medical personnel." |
| Limitations | 19 | Discuss limitations of the study, taking into account sources of potential bias or imprecision. Discuss both direction and magnitude of any potential bias | 4 | An advantage, as well as a limitation, of this study is the inclusion of closed groups for analysis. |
| Interpretation | 20 | Give a cautious overall interpretation of results considering objectives, limitations, multiplicity of analyses, results from similar studies, and other relevant evidence | 4 | to determine whether skin cancer patients would benefit from an Internet-based group that is secure, anonymous, and moderated for misinformation to provide emotional support, to improve patients' sense of community, and to offer education beyond the limited time allotted in clinical visits. |
| Generalisability | 21 | Discuss the generalisability (external validity) of the study results | 4 | Although not all Facebook groups meeting criteria could be analyzed owing to the inability to obtain permission for entry from the administrators, the groups that were analyzed provide insight in the level of information |

| | | | | |
|---|---|---|---|---|
| | | | | sharing among participants on a relatively secure platform. |
| **Other information** | | | | |
| Funding | 22 | Give the source of funding and the role of the funders for the present study and, if applicable, for the original study on which the present article is based | 4 | The authors declare no conflicts of interests. |

Table S83. Quality Evaluation for Included Studies Using STROBE

7.STROBE Statement—checklist of items that should be included in reports of observational studies

| | Item No. | Recommendation | Page No. | Relevant text from manuscript |
|---|---|---|---|---|
| **Title and abstract** | 1 | (*a*) Indicate the study's design with a commonly used term in the title or the abstract | NA | |
| | | (*b*) Provide in the abstract an informative and balanced summary of what was done and what was found | 1 | "Two cancer experts…1500 [810-4700], P..007)." |
| **Introduction** | | | | |
| Background/rationale | 2 | Explain the scientific background and rationale for the investigation being reported | 1 | Paragraph 1<br>"The internet is a leading source of health misinformation…associated with decreased survival" |
| Objectives | 3 | State specific objectives, including any prespecified hypotheses | 1 | quantify the accuracy of cancer treatment information on social media, its potential for harm, and how engagement differs by factualness and harm. |
| **Methods** | | | | |
| Study design | 4 | Present key elements of study design early in the paper | 1 | Retrospective cross-sectional study |
| Setting | 5 | Describe the setting, locations, and relevant dates, including periods of recruitment, exposure, follow-up, and data collection | | the most popular English language articles containing relevant keywords for the 4 most common cancers (breast, prostate, colorectal, and lung). These articles include any news article or blog posted on Facebook, Reddit, Twitter, or Pinterest between January 2018 and December 2019. |
| Participants | 6 | (*a*) *Cohort study*—Give the eligibility criteria, and the sources and methods of selection of participants. Describe methods of follow-up | 2 | The top 50 articles from each cancer type were collected, |

| | | | | |
|---|---|---|---|---|
| | | *Case-control study*—Give the eligibility criteria, and the sources and methods of case ascertainment and control selection. Give the rationale for the choice of cases and controls<br><br>*Cross-sectional study*—Give the eligibility criteria, and the sources and methods of selection of participants | | representing 200 unique articles |
| | | (*b*) *Cohort study*—For matched studies, give matching criteria and number of exposed and unexposed<br><br>*Case-control study*—For matched studies, give matching criteria and the number of controls per case | | |
| Variables | 7 | Clearly define all outcomes, exposures, predictors, potential confounders, and effect modifiers. Give diagnostic criteria, if applicable | 2 | Top 50 articles<br><br>outcome: 5-point Likert scale, Misinformation, Cohen kappa (OE) coefficient |
| Data sources/ measurement | 8* | For each variable of interest, give sources of data and details of methods of assessment (measurement). Describe comparability of assessment methods if there is more than one group | *1* | web-scraping software (Buzzsumo.com) to search for articles |
| Bias | 9 | Describe any efforts to address potential sources of bias | 2 | Inter-rater agreement was evaluated by Cohen kappa (OE) coefficient. |
| Study size | 10 | Explain how the study size was arrived at | 2 | 200 unique articles |

| | | | | |
|---|---|---|---|---|
| Quantitative variables | 11 | Explain how quantitative variables were handled in the analyses. If applicable, describe which groupings were chosen and why | 2 | 5-point Likert scale, Misinformation, Cohen kappa (OE) coefficient |
| Statistical methods | 12 | (*a*) Describe all statistical methods, including those used to control for confounding | 2 | The association of total and Facebook engagements between misinformation and harm was assessed using a 2-sample Wilcoxon rank-sum (Mann-Whitney) test |
| | | (*b*) Describe any methods used to examine subgroups and interactions | 2 | The association of total and Facebook engagements between misinformation and harm was assessed using a 2- |

| | | | | |
|---|---|---|---|---|
| | | | | sample Wilcoxon rank-sum (Mann-Whitney) test |
| | | (*c*) Explain how missing data were addressed | NA | |
| | | (*d*) *Cohort study*—If applicable, explain how loss to follow-up was addressed<br>*Case-control study*—If applicable, explain how matching of cases and controls was addressed<br>*Cross-sectional study*—If applicable, describe analytical methods taking account of sampling strategy | NA | |
| | | (*e*) Describe any sensitivity analyses | NA | |
| **Results** | | | | |
| Participants | 13* | (a) Report numbers of individuals at each stage of study—eg numbers potentially eligible, examined for eligibility, confirmed eligible, included in the study, completing follow-up, and analysed | 2 | Of the 200 articles, 37.5% (n.75), 41.5% (n.83), 1.0% (n.2), 3.0% (n.6), and 17.0% (n.34) were from traditional news (online versions of print and/or broadcast media), non-traditional news (digital only), personal blog, crowd-funding site, and medical journals, respectively |
| | | (b) Give reasons for non-participation at each stage | NA | |
| | | (c) Consider use of a flow diagram | NA | |
| Descriptive data | 14* | (a) Give characteristics of study participants (eg demographic, clinical, social) and information on exposures and potential confounders | 2 | Paragraph 6<br>"In total, 30.5%... contained harmful information." |
| | | (b) Indicate number of participants with missing data for each variable of interest | NA | |
| | | (c) *Cohort study*—Summarise follow-up time (eg, average and total amount) | NA | |
| Outcome data | 15* | *Cohort study*—Report numbers of outcome events or summary measures over time | | |
| | | *Case-control study*—Report numbers in each exposure category, or summary measures of exposure | | |
| | | *Cross-sectional study*—Report numbers of outcome events or summary measures | *2* | *Paragraph 7* |

| | | | | |
|---|---|---|---|---|
| | | | | "The median number of engagement… misinformation nor harm (all P>.63). |
| Main results | 16 | (*a*) Give unadjusted estimates and, if applicable, confounder-adjusted estimates and their precision (eg, 95% confidence interval). Make clear which confounders were adjusted for and why they were included | 2 | *The median engagement for articles with harmful information was statistically significantly greater than for safe articles* |
| | | (*b*) Report category boundaries when continuous variables were categorized | NA | |
| | | (*c*) If relevant, consider translating estimates of relative risk into absolute risk for a meaningful time period | NA | |
| Other analyses | 17 | Report other analyses done—eg analyses of subgroups and interactions, and sensitivity analyses | NA | |
| **Discussion** | | | | |
| Key results | 18 | Summarise key results with reference to study objectives | 2 | Between 2018 and 2019, nearly one-third of popular social media cancer articles contained misinformation and 76.9% of these contained harmful information. |
| Limitations | 19 | Discuss limitations of the study, taking into account sources of potential bias or imprecision. Discuss both direction and magnitude of any potential bias | 3 | Limitations of this study are that we included… |
| Interpretation | 20 | Give a cautious overall interpretation of results considering objectives, limitations, multiplicity of analyses, results from similar studies, and other relevant evidence | 2 | Paragraph 2<br>"Between 2018 and 2019… individuals susceptible to this influence." |
| Generalisability | 21 | Discuss the generalisability (external validity) of the study results | 3 | These findings could help lay the groundwork for future patient-specific tools and behavioral interventions to counter online cancer misinformation. |
| **Other information** | | | | |
| Funding | 22 | Give the source of funding and the role of the funders for the present study and, if applicable, for the original study on which the present article is based | 3 | This study was funded, in part, by the Huntsman Cancer |

Institute. Briony Swire-Thompson's, PhD effort was funded by an NIH Pathway to Independence Award.

Table S84. Quality Evaluation for Included Studies Using STROBE

8.STROBE Statement—checklist of items that should be included in reports of observational studies

| | Item No. | Recommendation | Page No. | Relevant text from manuscript |
|---|---|---|---|---|
| **Title and abstract** | 1 | (*a*) Indicate the study's design with a commonly used term in the title or the abstract | 1 | observational, cross-sectional study |
| | | (*b*) Provide in the abstract an informative and balanced summary of what was done and what was found | 2 | Materials and methods, results |
| **Introduction** | | | | |
| Background/rationale | 2 | Explain the scientific background and rationale for the investigation being reported | 1 | Introduction paragraph 1<br>"Social networks are increasingly…for the dissemination of misleading or imprecise information." |
| Objectives | 3 | State specific objectives, including any prespecified hypotheses | | The objective of our study is to describe the characteristics and analyze the veracity of the most frequently shared dermatological information on the most popular social networks. Knowing the prevalence and monitoring of this type of publication can help us understand how content is disseminated to detect gaps and guide future information campaigns. |
| **Methods** | | | | |
| Study design | 4 | Present key elements of study design early in the paper | 2 | Observational, cross-sectional |
| Setting | 5 | Describe the setting, locations, and relevant dates, including periods of recruitment, exposure, follow-up, and data collection | 2 | The 50 most shared websites between March 2019 and March 2020 |
| Participants | 6 | (*a*) *Cohort study*—Give the eligibility criteria, and the sources and methods of selection of participants. Describe methods of follow-up | 2 | Paragraph 2<br>"The 50 most shared websites between March |

| | | | | |
|---|---|---|---|---|
| | | *Case-control study*—Give the eligibility criteria, and the sources and methods of case ascertainment and control selection. Give the rationale for the choice of cases and controls<br><br>*Cross-sectional study*—Give the eligibility criteria, and the sources and methods of selection of participants | | 2019… no information about the disease was provided. |
| | | (*b*) *Cohort study*—For matched studies, give matching criteria and number of exposed and unexposed<br><br>*Case-control study*—For matched studies, give matching criteria and the number of controls per case | | |
| Variables | 7 | Clearly define all outcomes, exposures, predictors, potential confounders, and effect modifiers. Give diagnostic criteria, if applicable | 2 | classified the records according to their topic, origin, and level of certainty. The latter classification was made in three categories (imprecise, confusing, or precise) based on the scientific evidence available in PubMed, Scopus, Web of Science, and Google Scholar databases |
| Data sources/ measurement | 8* | For each variable of interest, give sources of data and details of methods of assessment (measurement). Describe comparability of assessment methods if there is more than one group | *1* | The data were obtained using the BuzzSumo application |
| Bias | 9 | Describe any efforts to address potential sources of bias | 2 | All data were analyzed independently by two dermatologists, and any discordance was evaluated by a third researcher |
| Study size | 10 | Explain how the study size was arrived at | 2 | 385 websites were included in the study, 50 for each entity studied except for "rosacea," where only 35 met the inclusion criteria. |

| | | | | |
|---|---|---|---|---|
| Quantitative variables | 11 | Explain how quantitative variables were handled in the analyses. If applicable, describe which groupings were chosen and why | 1 | *"likes" and "comments" related to one website* |
| Statistical methods | 12 | (*a*) Describe all statistical methods, including those used to control for confounding | 2 | Paragraph 3<br><br>Statistical analysis |

| | | | | |
|---|---|---|---|---|
| | | (*b*) Describe any methods used to examine subgroups and interactions | 3 | Table 1 |
| | | (*c*) Explain how missing data were addressed | NA | |
| | | (*d*) *Cohort study*—If applicable, explain how loss to follow-up was addressed<br>*Case-control study*—If applicable, explain how matching of cases and controls was addressed<br>*Cross-sectional study*—If applicable, describe analytical methods taking account of sampling strategy | NA | |
| | | (*e*) Describe any sensitivity analyses | NA | |
| **Results** | | | | |
| Participants | 13* | (a) Report numbers of individuals at each stage of study—eg numbers potentially eligible, examined for eligibility, confirmed eligible, included in the study, completing follow-up, and analysed | NA | |
| | | (b) Give reasons for non-participation at each stage | NA | |
| | | (c) Consider use of a flow diagram | NA | |
| Descriptive data | 14* | (a) Give characteristics of study participants (eg demographic, clinical, social) and information on exposures and potential confounders | 2 | Results paragraph 1<br>"*A total of 385 websites… 19.8% of the total*" |
| | | (b) Indicate number of participants with missing data for each variable of interest | NA | |
| | | (c) *Cohort study*—Summarise follow-up time (eg, average and total amount) | NA | |
| Outcome data | 15* | *Cohort study*—Report numbers of outcome events or summary measures over time | | |
| | | *Case-control study*—Report numbers in each exposure category, or summary measures of exposure | | |
| | | *Cross-sectional study*—Report numbers of outcome events or summary measures | *2* | Results paragraph 1<br>"*About 44.7% of content shared on social media... of the website (P = 0.041)*" |
| Main results | 16 | (*a*) Give unadjusted estimates and, if applicable, confounder-adjusted estimates and their precision (eg, 95% confidence interval). Make clear which confounders were adjusted for and why they were included | 2 | *About 44.7% of content shared on social media was rated as imprecise, 20% as confusing, and 35.3% as precise.* |
| | | (*b*) Report category boundaries when continuous variables were categorized | NA | |

| | | | | |
|---|---|---|---|---|
| | | (*c*) If relevant, consider translating estimates of relative risk into absolute risk for a meaningful time period | NA | |
| Other analyses | 17 | Report other analyses done—eg analyses of subgroups and interactions, and sensitivity analyses | 2 | Results paragraph 1<br>"*About 44.7% of content shared on social media... of the website (P = 0.041)*" |
| **Discussion** | | | | |
| Key results | 18 | Summarise key results with reference to study objectives | 3 | *All dermatoses studied presented a high proportion of information with a low level of evidence* |
| Limitations | 19 | Discuss limitations of the study, taking into account sources of potential bias or imprecision. Discuss both direction and magnitude of any potential bias | 3 | *However, some limitations…* |
| Interpretation | 20 | Give a cautious overall interpretation of results considering objectives, limitations, multiplicity of analyses, results from similar studies, and other relevant evidence | 3 | *Online information is not always precise nor does it come from reliable sources. In our study, 98% of imprecise articles were distributed through nonpeer-reviewed websites, compared to 69% for precise content (P < 0.001)* |
| Generalisability | 21 | Discuss the generalisability (external validity) of the study results | 4 | *Our study could contribute to the future development of health interventions and initiatives.* |
| **Other information** | | | | |
| Funding | 22 | Give the source of funding and the role of the funders for the present study and, if applicable, for the original study on which the present article is based | NA | |

Table S85. Quality Evaluation for Included Studies Using STROBE

9.STROBE Statement—checklist of items that should be included in reports of observational studies

| | Item No. | Recommendation | Page No. | Relevant text from manuscript |
|---|---|---|---|---|
| **Title and abstract** | 1 | (*a*) Indicate the study's design with a commonly used term in the title or the abstract | NA | |
| | | (*b*) Provide in the abstract an informative and balanced summary of what was done and what was found | 1 | Materials and Methods and Results |
| **Introduction** | | | | |
| Background/rationale | 2 | Explain the scientific background and rationale for the investigation being reported | 1 | *One in three adults in the USA searches online to diagnose a medical condition prior to seeking medical evaluation, suggesting that physicians are no longer the prime intermediary of health information [3]. While information is readily disseminated on social media platforms, it is essentially unregulated and lacks oversight. Concerns exist regarding the quality of health-related information* |
| Objectives | 3 | State specific objectives, including any prespecified hypotheses | 1 | *The aims of the present study were to evaluate the accuracy of the most popular articles shared across social media pertaining to genitourinary malignancy topics, and to identify the prevalence of misinformation available to patients on popular social media platforms.* |
| **Methods** | | | | |
| Study design | 4 | Present key elements of study design early in the paper | 2 | Retrospective cross-sectional |
| Setting | 5 | Describe the setting, locations, and relevant dates, including periods of recruitment, exposure, follow-up, and data collection | 2 | *Articles relating to prostate cancer, bladder cancer, kidney* |

| | | | | |
|---|---|---|---|---|
| | | | | *cancer, testis cancer, and PSA testing were identified between August 2017 and August 2018.* |
| Participants | 6 | (*a*) *Cohort study*—Give the eligibility criteria, and the sources and methods of selection of participants. Describe methods of follow-up<br>*Case-control study*—Give the eligibility criteria, and the sources and methods of case ascertainment and control selection. Give the rationale for the choice of cases and controls<br>*Cross-sectional study*—Give the eligibility criteria, and the sources and methods of selection of participants | 2 | *The 10 most shared articles relating to genitourinary malignancies on popular social media platforms* |
| | | (*b*) *Cohort study*—For matched studies, give matching criteria and number of exposed and unexposed<br>*Case-control study*—For matched studies, give matching criteria and the number of controls per case | NA | |
| Variables | 7 | Clearly define all outcomes, exposures, predictors, potential confounders, and effect modifiers. Give diagnostic criteria, if applicable | 2 | *accuracy* |
| Data sources/ measurement | 8* | For each variable of interest, give sources of data and details of methods of assessment (measurement). Describe comparability of assessment methods if there is more than one group | *2* | *Articles from BuzzSumo* |
| Bias | 9 | Describe any efforts to address potential sources of bias | | *Discordance between the independent reviewers was resolved by the principal investigator blinded to the decision of the reviewers* |
| Study size | 10 | Explain how the study size was arrived at | 2 | *A total of 50 articles* |

| | | | | |
|---|---|---|---|---|
| Quantitative variables | 11 | Explain how quantitative variables were handled in the analyses. If applicable, describe which groupings were chosen and why | | *Accuracy, Mann–Whitney score, Cohen's k coefficient* |
| Statistical methods | 12 | (*a*) Describe all statistical methods, including those used to control for confounding | 2 | *Analysis was performed using the Mann–Whitney U-test, with P values < 0.05 taken to indicate statistical significance. Inter-rater agreement between the independent reviewers was determined using Cohen's k coefficient.* |

| | | | | |
|---|---|---|---|---|
| | | (*b*) Describe any methods used to examine subgroups and interactions | | |
| | | (*c*) Explain how missing data were addressed | | |
| | | (*d*) *Cohort study*—If applicable, explain how loss to follow-up was addressed<br>*Case-control study*—If applicable, explain how matching of cases and controls was addressed<br>*Cross-sectional study*—If applicable, describe analytical methods taking account of sampling strategy | | |
| | | (*e*) Describe any sensitivity analyses | | |
| **Results** | | | | |
| Participants | 13* | (a) Report numbers of individuals at each stage of study—eg numbers potentially eligible, examined for eligibility, confirmed eligible, included in the study, completing follow-up, and analysed | NA | |
| | | (b) Give reasons for non-participation at each stage | | |
| | | (c) Consider use of a flow diagram | | |
| Descriptive data | 14* | (a) Give characteristics of study participants (eg demographic, clinical, social) and information on exposures and potential confounders | 2 | Results paragraph 2<br>"*Facebook was the most widely used social media platform on … bladder cancer (17 894), PSA testing (8827), and testicular cancer*" |
| | | (b) Indicate number of participants with missing data for each variable of interest | | |
| | | (c) *Cohort study*—Summarise follow-up time (eg, average and total amount) | | |
| Outcome data | 15* | *Cohort study*—Report numbers of outcome events or summary measures over time | | |
| | | *Case-control study*—Report numbers in each exposure category, or summary measures of exposure | | |
| | | *Cross-sectional study*—Report numbers of outcome events or summary measures | *2* | *A total of 35/50 articles (70%) were classified as accurate; only 40.0% (14/35) of the accurate articles were affiliated with an official institutional website, while 8.6% (3/35) were shared directly from a peer-reviewed journal website. The remaining articles (51.4%) were shared from non-affiliated and non-peer* |

| | | | | |
|---|---|---|---|---|
| | | | | *reviewed websites. A total of 15/50 articles (30%) were classified as inaccurate or misleading,* |
| Main results | 16 | (*a*) Give unadjusted estimates and, if applicable, confounder-adjusted estimates and their precision (eg, 95% confidence interval). Make clear which confounders were adjusted for and why they were included | 2 | *Overall, inaccurate articles were 28 times more likely to be shared than accurate articles. The majority of inaccurate or misleading articles were classified as such because they contained misinformation about diagnosis, prevention or treatment of a genitourinary malignancy* |
| | | (*b*) Report category boundaries when continuous variables were categorized | NA | |
| | | (*c*) If relevant, consider translating estimates of relative risk into absolute risk for a meaningful time period | NA | |
| Other analyses | 17 | Report other analyses done—eg analyses of subgroups and interactions, and sensitivity analyses | 2 | Results paragraph 3<br>"*The number of inaccurate articles was highest… PSA testing (1/10; Fig. 2).*" |
| **Discussion** | | | | |
| Key results | 18 | Summarise key results with reference to study objectives | 4 | Paragraph 1<br>"*The present study shows that in… other popular social media platforms.*" |
| Limitations | 19 | Discuss limitations of the study, taking into account sources of potential bias or imprecision. Discuss both direction and magnitude of any potential bias | 5 | *Paragraph 2*<br>*"however, the present study has some limitations that deserve mention…"* |
| Interpretation | 20 | Give a cautious overall interpretation of results considering objectives, limitations, multiplicity of analyses, results from similar studies, and other relevant evidence | 5 | *In conclusion, misleading or inaccurate information on genitourinary malignancies is commonly shared on social media platforms. This potential misinformation can have a* |

| | | | | |
|---|---|---|---|---|
| | | | | *detrimental impact on the public's or a patient's understanding of a disease and subsequent treatment decisions.* |
| Generalisability | 21 | Discuss the generalisability (external validity) of the study results | 5 | *In conclusion, misleading or inaccurate information on genitourinary malignancies is commonly shared on social media platforms. This potential misinformation can have a detrimental impact on the public's or a patient's understanding of a disease and subsequent treatment decisions.* |
| **Other information** | | | | |
| Funding | 22 | Give the source of funding and the role of the funders for the present study and, if applicable, for the original study on which the present article is based | 5 | *Conflicts of Interest None declared.* |

Table S86. Quality Evaluation for Included Studies Using STROBE

10.STROBE Statement—checklist of items that should be included in reports of observational studies

| | Item No. | Recommendation | Page No. | Relevant text from manuscript |
|---|---|---|---|---|
| **Title and abstract** | 1 | (*a*) Indicate the study's design with a commonly used term in the title or the abstract | 1 | Content and Network Analysis of Social Media Characteristics |
| | | (*b*) Provide in the abstract an informative and balanced summary of what was done and what was found | 1 | Methods and Results<br>"From April 2018…*by a health individual*" |
| **Introduction** | | | | |
| Background/rationale | 2 | Explain the scientific background and rationale for the investigation being reported | 2 | Introduction paragraph 2<br>"*Studies show that provaccine content on social media is … antivaccine content on social media.*" |
| Objectives | 3 | State specific objectives, including any prespecified hypotheses | 2 | Introduction last paragraph<br>"*To be proactive, we must … domains of misinformation?*" |
| **Methods** | | | | |
| Study design | 4 | Present key elements of study design early in the paper | 2 | Methods paragraph 1<br>"*Between April 2018 and December of 2018 … in prior Instagram research*" |
| Setting | 5 | Describe the setting, locations, and relevant dates, including periods of recruitment, exposure, follow-up, and data collection | 2 | *Between April 2018 and December of 2018, we used Netlytic software to collect public Instagram posts* |
| Participants | 6 | (*a*) *Cohort study*—Give the eligibility criteria, and the sources and methods of selection of participants. Describe methods of follow-up<br>*Case-control study*—Give the eligibility criteria, and the sources and methods of case ascertainment and control selection. Give the rationale for the choice of cases and controls | 2 | Methods paragraph 2-3<br>"*Drawing from prior social media … subsample included 605 posts.*" |

| | | | | |
|---|---|---|---|---|
| | | *Cross-sectional study*—Give the eligibility criteria, and the sources and methods of selection of participants | | |
| | | (*b*) *Cohort study*—For matched studies, give matching criteria and number of exposed and unexposed<br><br>*Case-control study*—For matched studies, give matching criteria and the number of controls per case | | |
| Variables | 7 | Clearly define all outcomes, exposures, predictors, potential confounders, and effect modifiers. Give diagnostic criteria, if applicable | 3 | *Manifest characteristics of posts' imagery, caption texts, and holistic post attributes (ie, source, context/style, and sentiment) were coded using a modified version of a codebook (see Multimedia Appendix 1) previously tested for reliability in analyzing HPV-related Instagram posts [10]. We organized elements of misinformation within four broad dimensions*<br><br>*The resultant visualization of these networks, produced by UCINET/Netdraw [26] software's graph theoretic spring-embedding algorithm, are shown in Figures 1 and 2.* |
| Data sources/ measurement | 8* | For each variable of interest, give sources of data and details of methods of assessment (measurement). Describe comparability of assessment methods if there is more than one group | *3* | *We organized elements of misinformation within four broad dimensions based on a review of the literature.*<br><br>*used Netlytic software to collect public Instagram posts.*<br><br>*The resultant visualization of these networks, produced by UCINET/Netdraw* |
| Bias | 9 | Describe any efforts to address potential sources of bias | 2 | *We randomly selected 1660 of the 16,607 posts (approximately* |

| | | | | |
|---|---|---|---|---|
| | | | | *10% of the final sample)* |
| Study size | 10 | Explain how the study size was arrived at | 3 | 580 posts |
| Quantitative variables | 11 | Explain how quantitative variables were handled in the analyses. If applicable, describe which groupings were chosen and why | 3, 5 | Categorical elements of misinformation<br>*'like' count,* |
| Statistical methods | 12 | (*a*) Describe all statistical methods, including those used to control for confounding | 5 | *Simple descriptive statistics were generated for pro- and antivaccine posts separately and in aggregate. Among both proand antivaccine posts, t tests and analysis of variance (ANOVA) assessed differences in 'like' count by post characteristics. chi-squared tests assessed differences in the distribution of characteristics between pro- and antivaccine posts.* |
| | | (*b*) Describe any methods used to examine subgroups and interactions | | |
| | | (*c*) Explain how missing data were addressed | | |
| | | (*d*) *Cohort study*—If applicable, explain how loss to follow-up was addressed<br>*Case-control study*—If applicable, explain how matching of cases and controls was addressed<br>*Cross-sectional study*—If applicable, describe analytical methods taking account of sampling strategy | | |
| | | (*e*) Describe any sensitivity analyses | | |
| **Results** | | | | |
| Participants | 13* | (a) Report numbers of individuals at each stage of study—eg numbers potentially eligible, examined for eligibility, confirmed eligible, included in the study, completing follow-up, and analysed | 5 | *Of the relevant and working 605 posts in our subsample, a small proportion (n=25, 4.1% coded posts) were determined to be neutral (ie, neither pro- nor antivaccine), and were therefore excluded from subsequent analyses. Thus, the final analytic sample included 256* |

| | | | | |
|---|---|---|---|---|
| | | | | *antivaccine posts and 324 provaccine posts (n=580 total).* |
| | | (b) Give reasons for non-participation at each stage | NA | |
| | | (c) Consider use of a flow diagram | NA | |
| Descriptive data | 14* | (a) Give characteristics of study participants (eg demographic, clinical, social) and information on exposures and potential confounders | 5 | Results: Content Analysis paragraph 1,2,3 |
| | | (b) Indicate number of participants with missing data for each variable of interest | NA | |
| | | (c) *Cohort study*—Summarise follow-up time (eg, average and total amount) | NA | |
| Outcome data | 15* | *Cohort study*—Report numbers of outcome events or summary measures over time | | |
| | | *Case-control study*—Report numbers in each exposure category, or summary measures of exposure | | |
| | | *Cross-sectional study*—Report numbers of outcome events or summary measures | 5 | Results: Content Analysis paragraph 4 |
| Main results | 16 | (*a*) Give unadjusted estimates and, if applicable, confounder-adjusted estimates and their precision (eg, 95% confidence interval). Make clear which confounders were adjusted for and why they were included | 5 | Anti-vaccine posts had higher engagement and were more likely to include conspiracy and anecdotal misinformation |
| | | (*b*) Report category boundaries when continuous variables were categorized | NA | |
| | | (*c*) If relevant, consider translating estimates of relative risk into absolute risk for a meaningful time period | NA | |

| | | | | |
|---|---|---|---|---|
| Other analyses | 17 | Report other analyses done—eg analyses of subgroups and interactions, and sensitivity analyses | 8 | Network analysis paragraph 1-3 |
| **Discussion** | | | | |
| Key results | 18 | Summarise key results with reference to study objectives | 8 | *The majority of Instagram posts in our HPV vaccine sample were provaccine and used hashtags*<br>*Our network diagram related to misinformation among antivaccine posts* |
| Limitations | 19 | Discuss limitations of the study, taking into account sources of potential bias or imprecision. Discuss both direction and magnitude of any potential bias | 9 | "*Our study has limitations worth noting…*" |

| | | | | |
|---|---|---|---|---|
| Interpretation | 20 | Give a cautious overall interpretation of results considering objectives, limitations, multiplicity of analyses, results from similar studies, and other relevant evidence | 9 | *Health misinformation on social media is diverse, tapping into states of reason to emotion. Identifying characteristics of health misinformation on social media will help inform targeted interventions and tailored messages to sow corrective information and stories* |
| Generalisability | 21 | Discuss the generalisability (external validity) of the study results | 10 | *Addressing misinformation on social media will require resource development and enthusiasm across multiple industries and health consumer types, including tech and health insurance companies, hospital and physician groups, and parent and cancer survivor advocates.* |
| **Other information** | | | | |
| Funding | 22 | Give the source of funding and the role of the funders for the present study and, if applicable, for the original study on which the present article is based | 10 | *This study was supported by the National Cancer Institute* |

Table S87. Quality Evaluation for Included Studies Using STROBE

11.STROBE Statement—checklist of items that should be included in reports of observational studies

| | Item No. | Recommendation | Page No. | Relevant text from manuscript |
|---|---|---|---|---|
| **Title and abstract** | 1 | (*a*) Indicate the study's design with a commonly used term in the title or the abstract | NA | |
| | | (*b*) Provide in the abstract an informative and balanced summary of what was done and what was found | 1 | *Methods. We performed a hand-coded content analysis on 797 Pinterest posts ("pins") mentioning the terms "breast cancer" or "breast" and "cancer," collected in November 2018.* <br> *Results. From the original sample of 797, 178 (22.3%) made a factual claim about what social media users could do to prevent or treat breast cancer. Of these, more than half—91 (51.1%)—contained misinformation. Therefore, 11.4% of the sample overall contained misinformation related to breast cancer prevention or treatment.* |
| **Introduction** | | | | |
| Background/rationale | 2 | Explain the scientific background and rationale for the investigation being reported | 1 | Paragraph 2 <br> "*Breast cancer misinformation … all US women use the site.*" |
| Objectives | 3 | State specific objectives, including any prespecified hypotheses | 2 | *The prevalence, typology, content, and sourcing of misinformation about breast cancer on Pinterest remain understudied. In our current study, we sought to fill this gap with a content analysis of 797 systematically selected pins.* |
| **Methods** | | | | |

| | | | | |
|---|---|---|---|---|
| Study design | 4 | Present key elements of study design early in the paper | 2 | Cross-sectional |
| Setting | 5 | Describe the setting, locations, and relevant dates, including periods of recruitment, exposure, follow-up, and data collection | 2 | *We used the software program ParseHub (ParseHub, Toronto, ON, Canada) to search the terms "breast cancer" and "breast + cancer," on November 14, 15, and 16, 2018, for a total of 6 searches* |
| Participants | 6 | (*a*) *Cohort study*—Give the eligibility criteria, and the sources and methods of selection of participants. Describe methods of follow-up<br>*Case-control study*—Give the eligibility criteria, and the sources and methods of case ascertainment and control selection. Give the rationale for the choice of cases and controls<br>*Cross-sectional study*—Give the eligibility criteria, and the sources and methods of selection of participants | | *We collected a sample of 838 pins, similar to previous research.15,16 This sample size was deemed large enough to perform the required statistical analyses (frequencies and c2s) while still being a manageable amount to code. We saved a screen capture of each pin. We found 41 of the links downloaded not to connect to extant pins, and we discarded these, leaving 797 pins for further analysis.* |
| | | (*b*) *Cohort study*—For matched studies, give matching criteria and number of exposed and unexposed<br>*Case-control study*—For matched studies, give matching criteria and the number of controls per case | | |
| Variables | 7 | Clearly define all outcomes, exposures, predictors, potential confounders, and effect modifiers. Give diagnostic criteria, if applicable | 2 | *2 phases, variables for the presence of misinformation in the image and in the text employed a grounded, qualitative coding approach to discover common themes in the types and content of misinformation in the posts.* |
| Data sources/ measurement | 8* | For each variable of interest, give sources of data and details of methods of assessment (measurement). Describe comparability of assessment methods if there is more than one group | *2* | *We used the software program ParseHub*<br>*We determined the presence of misinformation by comparing the content of the pins to reputable sources of* |

| | | | | |
|---|---|---|---|---|
| | | | | *information on breast cancer, including the Web sites of the Memorial Sloan Kettering Cancer Center (especially its guide to herbs and botanicals21), the World Cancer Research Fund (especially its guide to diet, nutrition, and physical activity22), the National Cancer Institute,23 BreastCancer.org, Susan G. Komen* |
| Bias | 9 | Describe any efforts to address potential sources of bias | 2 | *Using a random number generator, 80 pins were selected for a pretest, in which both researchers coded. Acceptable Krippendorff alphas were achieved for all quantitative variables* |
| Study size | 10 | Explain how the study size was arrived at | 2 | *797 pins* |

| | | | | |
|---|---|---|---|---|
| Quantitative variables | 11 | Explain how quantitative variables were handled in the analyses. If applicable, describe which groupings were chosen and why | 3 | *these variables (c2 [2; n = 178] = 8.51; Cramer's V = 0.219;* |
| Statistical methods | 12 | (*a*) Describe all statistical methods, including those used to control for confounding | 2 | *This sample size was deemed large enough to perform the required statistical analyses (frequencies and c2s) while still being a manageable amount to code* |
| | | (*b*) Describe any methods used to examine subgroups and interactions | 3 | "phase in posts" |
| | | (*c*) Explain how missing data were addressed | NA | |
| | | (*d*) *Cohort study*—If applicable, explain how loss to follow-up was addressed<br>*Case-control study*—If applicable, explain how matching of cases and controls was addressed<br>*Cross-sectional study*—If applicable, describe analytical methods taking account of sampling strategy | NA | |

| | | | | |
|---|---|---|---|---|
| | | (*e*) Describe any sensitivity analyses | NA | |
| **Results** | | | | |
| Participants | 13* | (a) Report numbers of individuals at each stage of study—eg numbers potentially eligible, examined for eligibility, confirmed eligible, included in the study, completing follow-up, and analysed | 2 | Results paragraph 1<br>"*Of pins that made a factual claim about … social media sites (2.2%), and other sites (2.2%).*" |
| | | (b) Give reasons for non-participation at each stage | NA | |
| | | (c) Consider use of a flow diagram | NA | |
| Descriptive data | 14* | (a) Give characteristics of study participants (eg demographic, clinical, social) and information on exposures and potential confounders | 2 | Results paragraph 1<br>"*Of pins that made a factual claim about … social media sites (2.2%), and other sites (2.2%).*" |
| | | (b) Indicate number of participants with missing data for each variable of interest | NA | |
| | | (c) *Cohort study*—Summarise follow-up time (eg, average and total amount) | NA | |
| Outcome data | 15* | *Cohort study*—Report numbers of outcome events or summary measures over time | *3* | *Phase in Posts and content of misinformation*<br>*"We performed a c2 test of independence to examine … herbs, or supplements (1.1%)."* |
| | | *Case-control study*—Report numbers in each exposure category, or summary measures of exposure | | |
| | | *Cross-sectional study*—Report numbers of outcome events or summary measures | | |
| Main results | 16 | (*a*) Give unadjusted estimates and, if applicable, confounder-adjusted estimates and their precision (eg, 95% confidence interval). Make clear which confounders were adjusted for and why they were included | 3 | *Phase in Posts*<br>*"Bonferroni pairwise comparisons showed that … and 8.0% related to both."* |
| | | (*b*) Report category boundaries when continuous variables were categorized | NA | |
| | | (*c*) If relevant, consider translating estimates of relative risk into absolute risk for a meaningful time period | NA | |

| | | | | |
|---|---|---|---|---|
| Other analyses | 17 | Report other analyses done—eg analyses of subgroups and interactions, and sensitivity analyses | 3 | *Subtype analysis:*<br>*Some of the less-common content of the misinformation pins included the stages of breast cancer (5.5%), symptoms (3.3%), surgery (3.3%), hormone treatment (2.2%), the role of faith in treatment (2.2%), breast selfexaminations (1.1%), benign lumps (1.1%), research organizations (1.1%), and alternative medicine, not including food, vitamins, minerals, herbs, or supplements (1.1%).* |
| **Discussion** | | | | |
| Key results | 18 | Summarise key results with reference to study objectives | 3 | Discussion paragraph 1, 3<br>"*These findings suggest that … influential over patient decision-making.* "<br>"*One encouraging finding was that conspiracy theories did not appear frequently among the misinformation*" |
| Limitations | 19 | Discuss limitations of the study, taking into account sources of potential bias or imprecision. Discuss both direction and magnitude of any potential bias | 4 | Public health implications paragraph 1<br>"*Health providers should be aware that many of their … more susceptible to misinformation*" |
| Interpretation | 20 | Give a cautious overall interpretation of results considering objectives, limitations, multiplicity of analyses, results from similar studies, and other relevant evidence | 3 | Discussion paragraph 2-3<br>"*The results demonstrate the complexity of the misinformation … appear frequently among the misinformation.*" |
| Generalisability | 21 | Discuss the generalisability (external validity) of the study results | 4 | *For public health advocates, the study suggests information to stress in public health campaigns, such as the need for* |

| | | | | |
|---|---|---|---|---|
| | | | | *mammograms and the importance of curbing established breast cancer risk factors.*<br><br>*This may begin to counteract misinformation present on Pinterest.* |
| **Other information** | | | | |
| Funding | 22 | Give the source of funding and the role of the funders for the present study and, if applicable, for the original study on which the present article is based | 4 | *The authors have no conflicts of interest to declare.* |

Table S88. Quality Evaluation for Included Studies Using STROBE

12.STROBE Statement—checklist of items that should be included in reports of observational studies

| | Item No. | Recommendation | Page No. | Relevant text from manuscript |
|---|---|---|---|---|
| **Title and abstract** | 1 | (*a*) Indicate the study's design with a commonly used term in the title or the abstract | 1 | Content analysis |
| | | (*b*) Provide in the abstract an informative and balanced summary of what was done and what was found | 1 | Methods and Results<br>"*We examined 540 Pinterest posts or pins, … 100% (n=359) of the pins, respectively.*" |
| **Introduction** | | | | |
| Background/rationale | 2 | Explain the scientific background and rationale for the investigation being reported | 2 | *Users are drawn to the easy accessibility of health care information. Unknowingly, much of the material they encounter is non–evidence-based, leaving them susceptible to misinformation.*<br>*Little is known about the quality of consumer-centric content about urological malignancies on Pinterest.* |
| Objectives | 3 | State specific objectives, including any prespecified hypotheses | 2 | *Our objective was to perform the first comprehensive study assessing the quality of content related to bladder, kidney, prostate, and testicular cancer on Pinterest.* |
| **Methods** | | | | |
| Study design | 4 | Present key elements of study design early in the paper | 2 | Cross-sectional content analysis |
| Setting | 5 | Describe the setting, locations, and relevant dates, including periods of recruitment, exposure, follow-up, and data collection | 2 | *reviewed 540 Pinterest pins, using the following search terms* |
| Participants | 6 | (*a*) *Cohort study*—Give the eligibility criteria, and the sources and methods of selection of participants. Describe methods of follow-up | 2 | *Pins were excluded if they did not contain relevant content (ie, if they did not* |

| | | | | |
|---|---|---|---|---|
| | | *Case-control study*—Give the eligibility criteria, and the sources and methods of case ascertainment and control selection. Give the rationale for the choice of cases and controls<br><br>*Cross-sectional study*—Give the eligibility criteria, and the sources and methods of selection of participants | | *mention gallbladder or thyroid cancer) or if they were not in English.* |
| | | (*b*) *Cohort study*—For matched studies, give matching criteria and number of exposed and unexposed<br><br>*Case-control study*—For matched studies, give matching criteria and the number of controls per case | | |
| Variables | 7 | Clearly define all outcomes, exposures, predictors, potential confounders, and effect modifiers. Give diagnostic criteria, if applicable | 2 | *2 validated questionnaires: the DISCERN quality criteria and Patient Education Materials Assessment Tool (PEMAT)*<br><br>*Misinformation was characterized using a previously published Likert scale presence of commercial bias dissemination of information by calculating the number of repins and followers associated with the Pinterest posts.*<br><br>*Classified people in pins based on perceived race and ethnicity* |
| Data sources/ measurement | 8* | For each variable of interest, give sources of data and details of methods of assessment (measurement). Describe comparability of assessment methods if there is more than one group | *2* | *Post from Pinterest,*<br><br>*Pins were assessed using 2 validated questionnaires: the DISCERN quality criteria and Patient Education Materials*<br><br>*Assessment Tool (PEMAT)* |
| Bias | 9 | Describe any efforts to address potential sources of bias | 2 | *Two reviewers independently scored each pin and linked content. Interrater discrepancies were addressed by group discussion.* |

| | | | | |
|---|---|---|---|---|
| Study size | 10 | Explain how the study size was arrived at | 2 | *359 pins* |
| Quantitative variables | 11 | Explain how quantitative variables were handled in the analyses. If applicable, describe which groupings were chosen and why | 2 | *2 validated questionnaires: the DISCERN quality criteria and Patient Education Materials*<br><br>*Assessment Tool (PEMAT)*<br><br>*Misinformation was characterized using a previously published Likert scale presence of commercial bias dissemination of information by calculating the number of repins and followers associated with the Pinterest posts.* |
| Statistical methods | 12 | (*a*) Describe all statistical methods, including those used to control for confounding | 2 | Descriptive statistics |
| | | (*b*) Describe any methods used to examine subgroups and interactions | NA | |
| | | (*c*) Explain how missing data were addressed | NA | |
| | | (*d*) *Cohort study*—If applicable, explain how loss to follow-up was addressed<br><br>*Case-control study*—If applicable, explain how matching of cases and controls was addressed<br><br>*Cross-sectional study*—If applicable, describe analytical methods taking account of sampling strategy | NA | |
| | | (*e*) Describe any sensitivity analyses | NA | |
| **Results** | | | | |
| Participants | 13* | (a) Report numbers of individuals at each stage of study—eg numbers potentially eligible, examined for eligibility, confirmed eligible, included in the study, completing follow-up, and analysed | 2 | Results paragraph 1<br><br>"*In total, 359 pins met the inclusion … doctors, academic journals, and medical education.*" |
| | | (b) Give reasons for non-participation at each stage | NA | |
| | | (c) Consider use of a flow diagram | 4 | |
| Descriptive data | 14* | (a) Give characteristics of study participants (eg demographic, clinical, social) and information on exposures and potential confounders | 5 | Paragraph 2<br><br>*Among the 206 total people depicted across all pins, the* |

| | | | | |
|---|---|---|---|---|
| | | | | *majority were perceived as White (n=178, 86%) and non-Latinx (n=184, 89%). Only 3% (n=7) of people were perceived as Black.*<br><br>*Bladder cancer pins did not include a Black individual. Additionally, fewer than 1% (n=2) of individuals represented in pins were perceived as Asian.* |
| | | (b) Indicate number of participants with missing data for each variable of interest | NA | |
| | | (c) *Cohort study*—Summarise follow-up time (eg, average and total amount) | NA | |
| Outcome data | 15* | *Cohort study*—Report numbers of outcome events or summary measures over time | | |
| | | *Case-control study*—Report numbers in each exposure category, or summary measures of exposure | | |
| | | *Cross-sectional study*—Report numbers of outcome events or summary measures | *5* | *Paragraph 1*<br><br>*"The overall quality … readily actionable information for users"* |
| Main results | 16 | (*a*) Give unadjusted estimates and, if applicable, confounder-adjusted estimates and their precision (eg, 95% confidence interval). Make clear which confounders were adjusted for and why they were included | 5 | *Misinformation ranged from 4% (n=4) in testicular cancer to 26% (n=16) in bladder cancer pins (eg, cow urine for the treatment of bladder cancer).* |
| | | (*b*) Report category boundaries when continuous variables were categorized | | |
| | | (*c*) If relevant, consider translating estimates of relative risk into absolute risk for a meaningful time period | | |

| | | | | |
|---|---|---|---|---|
| Other analyses | 17 | Report other analyses done—eg analyses of subgroups and interactions, and sensitivity analyses | 3 | Table 1<br><br>Analysis by cancer type and race |
| **Discussion** | | | | |
| Key results | 18 | Summarise key results with reference to study objectives | 5 | Discussion paragraph 1 Principle findings |

| | | | | |
|---|---|---|---|---|
| Limitations | 19 | Discuss limitations of the study, taking into account sources of potential bias or imprecision. Discuss both direction and magnitude of any potential bias | 6 | Limitation: *Our study is limited to Pinterest…* |
| Interpretation | 20 | Give a cautious overall interpretation of results considering objectives, limitations, multiplicity of analyses, results from similar studies, and other relevant evidence | 6 | *In summary, there is a vast array of urological oncology information available on Pinterest, but most of it is of moderate to very poor quality.* |
| Generalisability | 21 | Discuss the generalisability (external validity) of the study results | 6 | *Our study is limited to Pinterest, which is just one of many web-based networks.* *Also, the application of the validated questionaries to the Pinterest interface is a limitation.* |
| **Other information** | | | | |
| Funding | 22 | Give the source of funding and the role of the funders for the present study and, if applicable, for the original study on which the present article is based | 6 | *ASH, AM, and NH declare they have no conflicts of interest. RDM is an advisor for Urovant. SL declares equity in Gilead and is supported by Sanofi.* |

Table S89. Quality Evaluation for Included Studies Using STROBE

13.STROBE Statement—checklist of items that should be included in reports of observational studies

| | Item No. | Recommendation | Page No. | Relevant text from manuscript |
|---|---|---|---|---|
| **Title and abstract** | 1 | (*a*) Indicate the study's design with a commonly used term in the title or the abstract | 1 | Infodemiology Study |
| | | (*b*) Provide in the abstract an informative and balanced summary of what was done and what was found | 1 | Methods and Results<br>*"Reddit posts (from 2007 to 2017, N=28,121) … of misinformed posts (2666/7207, 36.99%)."* |
| **Introduction** | | | | |
| Background/rationale | 2 | Explain the scientific background and rationale for the investigation being reported | 2 | *Mitigation of medical and public health misinformation on social media is important; however, the sheer amount of information makes it challenging to identify these posts efficiently and accurately* |
| Objectives | 3 | State specific objectives, including any prespecified hypotheses | 2 | *We report the utility of various conventional ML and DL algorithms to automatically identify and categorize misinformation on the HPV vaccine using posts on Reddit,* |
| **Methods** | | | | |
| Study design | 4 | Present key elements of study design early in the paper | 3 | Retrospective cross-sectional data |
| Setting | 5 | Describe the setting, locations, and relevant dates, including periods of recruitment, exposure, follow-up, and data collection | 3 | *collected Reddit discussions related to HPV vaccination*<br>*from 2007 to 2017 (N=28,121) using Pushshift* |
| Participants | 6 | (*a*) *Cohort study*—Give the eligibility criteria, and the sources and methods of selection of participants. Describe methods of follow-up | 3 | *collected Reddit discussions related to HPV vaccination from 2007 to 2017 (N=28,121) using Pushshift* |

| | | | | |
|---|---|---|---|---|
| | | *Case-control study*—Give the eligibility criteria, and the sources and methods of case ascertainment and control selection. Give the rationale for the choice of cases and controls<br><br>*Cross-sectional study*—Give the eligibility criteria, and the sources and methods of selection of participants | | |
| | | (*b*) *Cohort study*—For matched studies, give matching criteria and number of exposed and unexposed<br><br>*Case-control study*—For matched studies, give matching criteria and the number of controls per case | | |
| Variables | 7 | Clearly define all outcomes, exposures, predictors, potential confounders, and effect modifiers. Give diagnostic criteria, if applicable | 3,4,5 | Outcome: *The resultant decision rules were that if a Reddit post contained one or more types of vaccine misinformation, it was considered an instance of misinformation*<br><br>*Misinformation Identification*<br><br>We performed stemming for each word to remove morphological affixes (eg, dies to die and denied to deni). The number of topics |
| Data sources/ measurement | 8* | For each variable of interest, give sources of data and details of methods of assessment (measurement). Describe comparability of assessment methods if there is more than one group | *3,4* | *Discussion from Reddit*<br><br>*We adopted spaCy tokenization [51] to split the post text into separate words* |
| Bias | 9 | Describe any efforts to address potential sources of bias | 4 | *The gold standard posts (ie, Reddit posts with expert-assigned labels) were randomly split into train, validation, and test sets in a ratio of 7:1:2.* |
| Study size | 10 | Explain how the study size was arrived at | 5 | *28,121 Reddit posts* |

| | | | | |
|---|---|---|---|---|
| Quantitative variables | 11 | Explain how quantitative variables were handled in the analyses. If applicable, describe which groupings were chosen and why | 4,5 | *Text classification is a fundamental task of natural language processing (NLP)*<br>*There were 207,651 upvotes (a user likes the post) and 10,700 downvotes* |
| Statistical methods | 12 | (*a*) Describe all statistical methods, including those used to control for confounding | 4 | *We evaluated 5 ML-based algorithms: 3 conventional and 2 DL algorithms* |
| | | (*b*) Describe any methods used to examine subgroups and interactions | NA | |
| | | (*c*) Explain how missing data were addressed | NA | |
| | | (*d*) *Cohort study*—If applicable, explain how loss to follow-up was addressed<br>*Case-control study*—If applicable, explain how matching of cases and controls was addressed<br>*Cross-sectional study*—If applicable, describe analytical methods taking account of sampling strategy | NA | |
| | | (*e*) Describe any sensitivity analyses | NA | |
| **Results** | | | | |
| Participants | 13* | (a) Report numbers of individuals at each stage of study—eg numbers potentially eligible, examined for eligibility, confirmed eligible, included in the study, completing follow-up, and analysed | 5 | *In total, 28,121 Reddit posts were collected from 2007 to 2017 from more than 16,633 unique users.* |
| | | (b) Give reasons for non-participation at each stage | NA | |
| | | (c) Consider use of a flow diagram | 3 | |
| Descriptive data | 14* | (a) Give characteristics of study participants (eg demographic, clinical, social) and information on exposures and potential confounders | 5 | Results paragraph 1<br>"*In total, 28,121 Reddit posts were collected … high performance for the classification algorithms.*" |
| | | (b) Indicate number of participants with missing data for each variable of interest | NA | |
| | | (c) *Cohort study*—Summarise follow-up time (eg, average and total amount) | NA | |
| Outcome data | 15* | *Cohort study*—Report numbers of outcome events or summary measures over time | *6* | *Paragraph 1-2* |

| | | | | |
|---|---|---|---|---|
| | | | | *"The LR algorithm demonstrated the highest AUC value … subset of posts were classified as vaccine misinformation."* |
| | | *Case-control study*—Report numbers in each exposure category, or summary measures of exposure | | |
| | | *Cross-sectional study*—Report numbers of outcome events or summary measures | | |
| Main results | 16 | (*a*) Give unadjusted estimates and, if applicable, confounder-adjusted estimates and their precision (eg, 95% confidence interval). Make clear which confounders were adjusted for and why they were included | 6 | *The precision and recall curves of the CNN model are shown in Figure 2.* |
| | | (*b*) Report category boundaries when continuous variables were categorized | NA | |
| | | (*c*) If relevant, consider translating estimates of relative risk into absolute risk for a meaningful time period | NA | |

| | | | | |
|---|---|---|---|---|
| Other analyses | 17 | Report other analyses done—eg analyses of subgroups and interactions, and sensitivity analyses | 7 | *Misinformation Network Analysis* |
| **Discussion** | | | | |
| Key results | 18 | Summarise key results with reference to study objectives | 7-8 | *Principal Findings…* |
| Limitations | 19 | Discuss limitations of the study, taking into account sources of potential bias or imprecision. Discuss both direction and magnitude of any potential bias | 8 | *Limitations and Future Work…* |
| Interpretation | 20 | Give a cautious overall interpretation of results considering objectives, limitations, multiplicity of analyses, results from similar studies, and other relevant evidence | 9 | *Our ML-based approaches demonstrated efficacy in the automated identification and classification of HPV vaccine misinformation in discussions on the social media platform Reddit.* |
| Generalisability | 21 | Discuss the generalisability (external validity) of the study results | 9 | *Although our ML algorithm does not solve the problem of health and vaccine misinformation single-handedly, we provide an innovative stepping stone that may bridge multiple approaches for combating this invasive and growing public health concern.* |
| **Other information** | | | | |

| | | | | |
|---|---|---|---|---|
| Funding | 22 | Give the source of funding and the role of the funders for the present study and, if applicable, for the original study on which the present article is based | 9 | *This research was supported by the National Institutes of Health under award numbers* |

Table S90. Quality Evaluation for Included Studies Using STROBE

14. STROBE Statement—checklist of items that should be included in reports of observational studies

| | Item No. | Recommendation | Page No. | Relevant text from manuscript |
|---|---|---|---|---|
| **Title and abstract** | 1 | (*a*) Indicate the study's design with a commonly used term in the title or the abstract | 1 | Quality analysis |
| | | (*b*) Provide in the abstract an informative and balanced summary of what was done and what was found | 1 | Methods and Results<br>"A search of TikTok was performed … content scores were not correlated with VPI." |
| **Introduction** | | | | |
| Background/rationale | 2 | Explain the scientific background and rationale for the investigation being reported | 2 | Educational healthcare content has also become an important part of TikTok's content ecosystem (20). However, the unregulated content of TikTok video and the lack of peer review process increases the likelihood of dissemination of inaccurate and large volumes of information with varying quality and credibility, raising a significant challenge in the provision of optimal healthcare |
| Objectives | 3 | State specific objectives, including any prespecified hypotheses | 2 | the quality of short video content regarding to TC has not yet been analyzed. To address this gap, this study aims to assess the quality of the short videos about TC from TikTok. |
| **Methods** | | | | |
| Study design | 4 | Present key elements of study design early in the paper | 2 | Cross-sectional content analysis |

| | | | | |
|---|---|---|---|---|
| Setting | 5 | Describe the setting, locations, and relevant dates, including periods of recruitment, exposure, follow-up, and data collection | 2 | ("Thyroid cancer" and "thyroid neoplasm" in Chinese) was searched within TikTok app on March 20, 2022. |
| Participants | 6 | (*a*) *Cohort study*—Give the eligibility criteria, and the sources and methods of selection of participants. Describe methods of follow-up<br>*Case-control study*—Give the eligibility criteria, and the sources and methods of case ascertainment and control selection. Give the rationale for the choice of cases and controls<br>*Cross-sectional study*—Give the eligibility criteria, and the sources and methods of selection of participants | 2 | The first 100 videos that appeared on each search were reviewed. A total of 56 videos were finally included for analyzing in this study after excluding videos with advertising content ($n$ = 8), duplicated ($n$ = 36), |
| | | (*b*) *Cohort study*—For matched studies, give matching criteria and number of exposed and unexposed<br>*Case-control study*—For matched studies, give matching criteria and the number of controls per case | | |
| Variables | 7 | Clearly define all outcomes, exposures, predictors, potential confounders, and effect modifiers. Give diagnostic criteria, if applicable | 2 | Information of videos was extracted and coded, including source of video, the content of the videos, the presence of animation, duration (in seconds), the upload date, and other viewer interactive quality markers including number of views, likes, and comments. The VPI was calculated using the formula "(number of likes/number of views) . 100)" |
| Data sources/ measurement | 8* | For each variable of interest, give sources of data and details of methods of assessment (measurement). Describe comparability of assessment methods if there is more than one group | *2* | *Data was* searched within TikTok app |
| Bias | 9 | Describe any efforts to address potential sources of bias | 2 | All selected videos were categorized into three groups according to source: physicians, hospital channel (non-profit organization), and |

| | | | | |
|---|---|---|---|---|
| | | | | health organizations (for-profit organizations).<br>Two reviewers independently appraised the quality of the included videos. |
| Study size | 10 | Explain how the study size was arrived at | | A total of 56 videos |
| Quantitative variables | 11 | Explain how quantitative variables were handled in the analyses. If applicable, describe which groupings were chosen and why | 2 | The quality of information was rated using an adapted DISCERN tool<br>Content score was rated in terms of six predefined questions |
| Statistical methods | 12 | (*a*) Describe all statistical methods, including those used to control for confounding | 2 | include descriptive statistical analysis, Pearson correlation analysis, and one-way ANOVA. The continuous variables were expressed as mean ± standard deviation, median (min-max), while nominal variables were given as frequency and percentage. $p < 0.05$ was considered statistically significant. |
| | | (*b*) Describe any methods used to examine subgroups and interactions | NA | |
| | | (*c*) Explain how missing data were addressed | NA | |
| | | (*d*) *Cohort study*—If applicable, explain how loss to follow-up was addressed<br>*Case-control study*—If applicable, explain how matching of cases and controls was addressed<br>*Cross-sectional study*—If applicable, describe analytical methods taking account of sampling strategy | NA | |
| | | (*e*) Describe any sensitivity analyses | NA | |
| **Results** | | | | |

| | | | | |
|---|---|---|---|---|
| Participants | 13* | (a) Report numbers of individuals at each stage of study—eg numbers potentially eligible, examined for eligibility, confirmed eligible, included in the study, completing follow-up, and analysed | 2 | Of the 56 videos included in this study, 49 (87.5%) were uploaded by physicians, 4 (7.1%) by health organizations, and 3 (5.4%) by hospital channels. There wasn't video from patients or their relatives. Forty three (76.8%) videos were real content videos, 13 (23.2%) were videos with animation. |
| | | (b) Give reasons for non-participation at each stage | NA | |
| | | (c) Consider use of a flow diagram | NA | |
| Descriptive data | 14* | (a) Give characteristics of study participants (eg demographic, clinical, social) and information on exposures and potential confounders | 3 | The duration of the videos varied from 5 to 111 s. The least online days of video were 2 days prior to data collection, whereas the most online days were nearly 3 years. Median (min-max) number of views, likes, comments, collection, sharing, and VPI were 46,211 (578–11,088,000), 1378.5 (22–308,000), 83 (0–73,000), 82.5 (1–1775), 130.5 (15–5,796) and 2.5 (2–8.82) prior to data collection. There were no significant differences among different sources of video (physician, hospital channel, and health organization) regarding such video characteristics mentioned above except for VPI between health organization and hospital channel ($p = 0.002$), physicians ($p < 0.001$). |
| | | (b) Indicate number of participants with missing data for each variable of interest | NA | |
| | | (c) *Cohort study*—Summarise follow-up time (eg, average and total amount) | NA | |
| Outcome data | 15* | *Cohort study*—Report numbers of outcome events or summary measures over time | | |

| | | | | |
|---|---|---|---|---|
| | | *Case-control study*—Report numbers in each exposure category, or summary measures of exposure | | |
| | | *Cross-sectional study*—Report numbers of outcome events or summary measures | *3* | Quality assessment of video… |
| Main results | 16 | (*a*) Give unadjusted estimates and, if applicable, confounder-adjusted estimates and their precision (eg, 95% confidence interval). Make clear which confounders were adjusted for and why they were included | 3 | The average DISCERN score given by the two reviewers was 3.44 ± 0.72, while the DISCERN score for individual reviewers was 3.5 ± 0.71 and 3.38 ± 0.73, respectively.<br>most of videos sufficiently addressed definition (53/56, 94.6%), signs and symptoms (50/56, 89.3%), management (52/56, 92.9%), evaluation (51/56, 91.1%) and outcomes (54/56, 96.4%), while risk factors (32/56, 57.1%) were absent in 42.9% videos. |
| | | (*b*) Report category boundaries when continuous variables were categorized | NA | |
| | | (*c*) If relevant, consider translating estimates of relative risk into absolute risk for a meaningful time period | NA | |

| | | | | |
|---|---|---|---|---|
| Other analyses | 17 | Report other analyses done—eg analyses of subgroups and interactions, and sensitivity analyses | 4 | The duration of the video and presence of animation were positively correlated with VPI of videos (duration: $r = 0.40$, $p = 0.002$; animation: $r = 0.52$, $p < 0.001$). Both DISCERN score and content score were not correlated with VPI, and no correlation was noted between DISCERN score and content score |
| **Discussion** | | | | |
| Key results | 18 | Summarise key results with reference to study objectives | 4 | Our results suggest that the overall quality of TC-related videos from TikTok was satisfactory |

| | | | | |
|---|---|---|---|---|
| Limitations | 19 | Discuss limitations of the study, taking into account sources of potential bias or imprecision. Discuss both direction and magnitude of any potential bias | 5 | Limitations to this study should be mentioned. First, the search results on TikTok are dynamic over time; the results thus might vary with the use of different search dates and time. Second, the research results may vary according to the geographical location of the viewer. Third, video sampling retrieves only the first 100 in each search which can lead to inadequate coverage. |
| Interpretation | 20 | Give a cautious overall interpretation of results considering objectives, limitations, multiplicity of analyses, results from similar studies, and other relevant evidence | 5 | The overall quality of information based on DISCERN scores and content scores was satisfactory. Our finding also supports TikTok as a new source of information on TC, |
| Generalisability | 21 | Discuss the generalisability (external validity) of the study results | 5 | physicians and hospitals should embrace this evolving technology and provide videos with higher quality to improve patients' awareness about TC. Patients should also be cautious when watching TC relevant videos on TikTok due to the complexity of VPI |
| **Other information** | | | | |
| Funding | 22 | Give the source of funding and the role of the funders for the present study and, if applicable, for the original study on which the present article is based | 5 | This work was supported by Science and Technology Department of Zhejiang Province |

Table S91. Quality Evaluation for Included Studies Using STROBE

15. STROBE Statement—checklist of items that should be included in reports of observational studies

| | Item No. | Recommendation | Page No. | Relevant text from manuscript |
|---|---|---|---|---|
| **Title and abstract** | 1 | (*a*) Indicate the study's design with a commonly used term in the title or the abstract | 1 | Quality analysis |
| | | (*b*) Provide in the abstract an informative and balanced summary of what was done and what was found | 1 | Methods and Results<br>"*A search was performed on TikTok … assess the quality of videos"* |
| **Introduction** | | | | |
| Background/rationale | 2 | Explain the scientific background and rationale for the investigation being reported | 2 | Paragraph 2<br>"With the popularity of TikTok, studies have … with some misleading videos |
| Objectives | 3 | State specific objectives, including any prespecified hypotheses | 2 | This study aims to evaluate the quality of thyroid cancer<br>Related videos on the TikTok application. |
| **Methods** | | | | |
| Study design | 4 | Present key elements of study design early in the paper | 2 | Cross-sectional content analysis |
| Setting | 5 | Describe the setting, locations, and relevant dates, including periods of recruitment, exposure, follow-up, and data collection | 2 | *On May 4, 2022, a search was performed on TikTok with the keyword "thyroid cancer".* |
| Participants | 6 | (*a*) *Cohort study*—Give the eligibility criteria, and the sources and methods of selection of participants. Describe methods of follow-up<br>*Case-control study*—Give the eligibility criteria, and the sources and methods of case ascertainment and control selection. Give the rationale for the choice of cases and controls<br>*Cross-sectional study*—Give the eligibility criteria, and the sources and methods of selection of participants | 2 | *The top 100 videos in the search results were selected for the study.* |
| | | (*b*) *Cohort study*—For matched studies, give matching criteria and number of exposed and unexposed | | |

| | | | | |
|---|---|---|---|---|
| | | *Case-control study*—For matched studies, give matching criteria and the number of controls per case | | |
| Variables | 7 | Clearly define all outcomes, exposures, predictors, potential confounders, and effect modifiers. Give diagnostic criteria, if applicable | 2 | *Characteristics of videos were collected on May 4, 2022,including video length, duration on TikTok, thumbs up, thumbs up/day, comments, comments/day, favorites, favorites/day, reposts, and reposts/day. Two authors (S Y and J Z) independently viewed the videos between May 5 and May 11, assessed the quality of the videos, and categorized the videos based on uploaders and content.* |
| Data sources/ measurement | 8* | For each variable of interest, give sources of data and details of methods of assessment (measurement). Describe comparability of assessment methods if there is more than one group | *2* | *Videos are from TikTok Two authors (S Y and J Z) categorized the videos based on uploaders and content.* |
| Bias | 9 | Describe any efforts to address potential sources of bias | 2 | *To avoid bias from personalized recommendations, we logged out of the TikTok account and cleared the search history before searching. The search results were presented in the default collation without any filtering criteria.* |
| Study size | 10 | Explain how the study size was arrived at | 2 | 100 videos |
| Quantitative variables | 11 | Explain how quantitative variables were handled in the analyses. If applicable, describe which groupings were chosen and why | 2 | *the Patient Education Materials Assessment Tool (PEMAT), modified DISCERN (mDISCERN), Video Information and Quality Index (VIQI), and Global Quality Score (GQS) to perform a comprehensive* |

| | | | | |
|---|---|---|---|---|
| | | | | *assessment of information quality* |
| Statistical methods | 12 | (*a*) Describe all statistical methods, including those used to control for confounding | 2 | *Continuous variables were reported as the mean (SD) and min-max values for descriptive data, and categorical variables were reported as rates. The Kruskal–Wallis test was applied for comparisons between more than two groups, and the Bonferroni post hoc test was applied for comparisons between two groups. The chi-square test and Fisher's exact test were used to compare categorical variables.* |
| | | (*b*) Describe any methods used to examine subgroups and interactions | 2 | *The video uploaders were categorized, and the content of the videos was categorized* |
| | | (*c*) Explain how missing data were addressed | NA | |
| | | (*d*) *Cohort study*—If applicable, explain how loss to follow-up was addressed<br>*Case-control study*—If applicable, explain how matching of cases and controls was addressed<br>*Cross-sectional study*—If applicable, describe analytical methods taking account of sampling strategy | NA | |
| | | (*e*) Describe any sensitivity analyses | NA | |
| **Results** | | | | |
| Participants | 13* | (a) Report numbers of individuals at each stage of study—eg numbers potentially eligible, examined for eligibility, confirmed eligible, included in the study, completing follow-up, and analysed | 3 | Video characteristics paragraph 1 |
| | | (b) Give reasons for non-participation at each stage | NA | |
| | | (c) Consider use of a flow diagram | NA | |
| Descriptive data | 14* | (a) Give characteristics of study participants (eg demographic, clinical, social) and information on exposures and potential confounders | 3 | Video characteristics paragraph 2,3 |
| | | (b) Indicate number of participants with missing data for each variable of interest | NA | |

| | | | | |
|---|---|---|---|---|
| | | (c) *Cohort study*—Summarise follow-up time (eg, average and total amount) | NA | |
| Outcome data | 15* | *Cohort study*—Report numbers of outcome events or summary measures over time | NA | |
| | | *Case-control study*—Report numbers in each exposure category, or summary measures of exposure | NA | |
| | | *Cross-sectional study*—Report numbers of outcome events or summary measures | *3-4* | *Last paragraph*<br>*"As shown in Table 2, the mean PEMAT score for the 100 videos was 71.18 ± 16.56 … mean GQS score of the videos was 3.72 ± 1.00."* |
| Main results | 16 | (*a*) Give unadjusted estimates and, if applicable, confounder-adjusted estimates and their precision (eg, 95% confidence interval). Make clear which confounders were adjusted for and why they were included | 4-5 | *Last paragraph*<br>*"In contrast, videos by different uploaders differed … score of the VIQI tool depending on uploaders"* |
| | | (*b*) Report category boundaries when continuous variables were categorized | NA | |
| | | (*c*) If relevant, consider translating estimates of relative risk into absolute risk for a meaningful time period | NA | |

| | | | | |
|---|---|---|---|---|
| Other analyses | 17 | Report other analyses done—eg analyses of subgroups and interactions, and sensitivity analyses | 4 | *the association between the characteristics and quality of videos and different uploaders. There were no significant differences in video length.* |
| **Discussion** | | | | |
| Key results | 18 | Summarise key results with reference to study objectives | 6 | Paragraph 1<br>*Although recommended by the video application's algorithm to the top of the search results, these videos are less informative and not of high quality.* |
| Limitations | 19 | Discuss limitations of the study, taking into account sources of potential bias or imprecision. Discuss both direction and magnitude of any potential bias | 6 | Last paragraph<br>"There are several limitations in this study…" |

| | | | | |
|---|---|---|---|---|
| Interpretation | 20 | Give a cautious overall interpretation of results considering objectives, limitations, multiplicity of analyses, results from similar studies, and other relevant evidence | 7 | *although the prevalence and public attention to thyroid cancer are increasing, the quality of relevant information on the most popular short-video application remains substandard* |
| Generalisability | 21 | Discuss the generalisability (external validity) of the study results | 6 | Last paragraph<br><br>*since we only examined videos from a single platform, TikTok, and did not include other popular short-form video applications, such as Kwai, Xigua Video, and social media platforms used by people in other countries, the generalizability needs to be investigated in further studies.* |
| **Other information** | | | | |
| Funding | 22 | Give the source of funding and the role of the funders for the present study and, if applicable, for the original study on which the present article is based | 7 | *This study is supported by the National Natural Science Foundation of China (32071436) and the Beijing Municipal Natural Science Foundation (7222127).* |

Table S92. Quality Evaluation for Included Studies Using STROBE

16. STROBE Statement—checklist of items that should be included in reports of observational studies

| | Item No. | Recommendation | Page No. | Relevant text from manuscript |
|---|---|---|---|---|
| **Title and abstract** | 1 | (*a*) Indicate the study's design with a commonly used term in the title or the abstract | 1 | Cross-Sectional Content Analysis |
| | | (*b*) Provide in the abstract an informative and balanced summary of what was done and what was found | 2 | Methods and Results<br>"*In March 2023, we assessed … and no video variables could predict the video quality*" |
| **Introduction** | | | | |
| Background/rationale | 2 | Explain the scientific background and rationale for the investigation being reported | 2 | Introduction paragraph 2<br>"*Short-form video-sharing platforms have become more popular in recent years, resulting in an increase in the number of health-related short videos available to the public. Nevertheless, concerns have been expressed regarding the quality and content of these short videos*" |
| Objectives | 3 | State specific objectives, including any prespecified hypotheses | 2 | *Our study aims to evaluate the content, quality, and reliability of short videos on liver cancer in Bilibili and TikTok.* |
| **Methods** | | | | |
| Study design | 4 | Present key elements of study design early in the paper | 2 | *cross-sectional study* |
| Setting | 5 | Describe the setting, locations, and relevant dates, including periods of recruitment, exposure, follow-up, and data collection | 2 | *search the top 100 videos in the Chinese versions of TikTok and Bilibili on March 2, 2023* |
| Participants | 6 | (*a*) *Cohort study*—Give the eligibility criteria, and the sources and methods of selection of participants. Describe methods of follow-up | 2 | *search the top 100 videos* |

| | | | | |
|---|---|---|---|---|
| | | *Case-control study*—Give the eligibility criteria, and the sources and methods of case ascertainment and control selection. Give the rationale for the choice of cases and controls<br><br>*Cross-sectional study*—Give the eligibility criteria, and the sources and methods of selection of participants | | |
| | | (*b*) *Cohort study*—For matched studies, give matching criteria and number of exposed and unexposed<br><br>*Case-control study*—For matched studies, give matching criteria and the number of controls per case | | |
| Variables | 7 | Clearly define all outcomes, exposures, predictors, potential confounders, and effect modifiers. Give diagnostic criteria, if applicable | 2 | *The name of the video; the name and identity of the uploader; the length of the video; the content delivered; the number of likes, comments, shares, and saves it received; and the number of days since it was published* |
| Data sources/ measurement | 8* | For each variable of interest, give sources of data and details of methods of assessment (measurement). Describe comparability of assessment methods if there is more than one group | *2* | *Video is come from Chinese versions of TikTok and Bilibili* |
| Bias | 9 | Describe any efforts to address potential sources of bias | 3 | *Video Quality and Reliability Assessments paragraph 2*<br><br>*"New accounts were registered and … agreement between the 2 raters."* |
| Study size | 10 | Explain how the study size was arrived at | 4 | *200 videos* |

| | | | | |
|---|---|---|---|---|
| Quantitative variables | 11 | Explain how quantitative variables were handled in the analyses. If applicable, describe which groupings were chosen and why | 3 | *The quality of the information in the videos was assessed using GQS, and the reliability was evaluated using DISCERN.* |
| Statistical methods | 12 | (*a*) Describe all statistical methods, including those used to control for confounding | 4 | *the median (IQR) was used for the descriptive statistics. The Kruskal-Wallis test was used to assess the differences between groups, and Dunn multiple comparison test was used for 2-* |

| | | | | |
|---|---|---|---|---|
| | | | | *way intergroup comparisons. We used Cohen κ to quantify the agreement between the 2 raters. We performed Spearman correlation analysis to evaluate the relationship between quantitative variables. Poisson regression models were used to assess the effects of video variables on video quality* |
| | | (*b*) Describe any methods used to examine subgroups and interactions | 6 | Table 6 |
| | | (*c*) Explain how missing data were addressed | NA | |
| | | (*d*) *Cohort study*—If applicable, explain how loss to follow-up was addressed<br>*Case-control study*—If applicable, explain how matching of cases and controls was addressed<br>*Cross-sectional study*—If applicable, describe analytical methods taking account of sampling strategy | NA | |
| | | (*e*) Describe any sensitivity analyses | NA | |
| **Results** | | | | |
| Participants | 13* | (a) Report numbers of individuals at each stage of study—eg numbers potentially eligible, examined for eligibility, confirmed eligible, included in the study, completing follow-up, and analysed | 4 | *Video Characteristics* |
| | | (b) Give reasons for non-participation at each stage | NA | |
| | | (c) Consider use of a flow diagram | 3 | |
| Descriptive data | 14* | (a) Give characteristics of study participants (eg demographic, clinical, social) and information on exposures and potential confounders | 5 | "*Table 5, Table 6, and Figure 2 show the video source … those covering news and reports (42/100, 42%).*" |
| | | (b) Indicate number of participants with missing data for each variable of interest | NA | |
| | | (c) *Cohort study*—Summarise follow-up time (eg, average and total amount) | NA | |
| Outcome data | 15* | *Cohort study*—Report numbers of outcome events or summary measures over time | | |
| | | *Case-control study*—Report numbers in each exposure category, or summary measures of exposure | | |

| | | | | |
|---|---|---|---|---|
| | | *Cross-sectional study*—Report numbers of outcome events or summary measures | *6,7* | *Video Quality and Reliability Assessments* |
| Main results | 16 | (*a*) Give unadjusted estimates and, if applicable, confounder-adjusted estimates and their precision (eg, 95% confidence interval). Make clear which confounders were adjusted for and why they were included | 6 | *the TikTok videos were of fair quality and reliability.*<br>*most Bilibili videos were of poor quality and low reliability.* |
| | | (*b*) Report category boundaries when continuous variables were categorized | NA | |
| | | (*c*) If relevant, consider translating estimates of relative risk into absolute risk for a meaningful time period | NA | |
| Other analyses | 17 | Report other analyses done—eg analyses of subgroups and interactions, and sensitivity analyses | 7,9 | *To explore whether different types of professional individuals impacted the quality and reliability of the videos, we further divided the professional individual–sourced videos into 4 groups.*<br>*Correlation Analysis and Poisson Regression Analysis* |
| **Discussion** | | | | |
| Key results | 18 | Summarise key results with reference to study objectives | 11 | *The overall quality of TikTok and Bilibili short videos related to liver cancer was unsatisfactory according to the GQS and DISCERN scores, which may be attributed to the relatively low standards set for access to these platforms and the lack of video censorship* |
| Limitations | 19 | Discuss limitations of the study, taking into account sources of potential bias or imprecision. Discuss both direction and magnitude of any potential bias | 12 | However, there are some limitations in our study…. |
| Interpretation | 20 | Give a cautious overall interpretation of results considering objectives, limitations, multiplicity of analyses, results from similar studies, and other relevant evidence | 13 | *Overall, videos by medical professionals were more instructive in terms of the comprehensiveness, quality, and reliability of content than videos by nonmedical professionals. Videos by professionals conveying knowledge such as* |

| | | | | |
|---|---|---|---|---|
| | | | | *disease knowledge are likely to be of higher quality* |
| Generalisability | 21 | Discuss the generalisability (external validity) of the study results | 12 | *he findings may not be generalizable to other language platforms. Subsequent cross-linguistic research is required to fill this gap.* |
| **Other information** | | | | |
| Funding | 22 | Give the source of funding and the role of the funders for the present study and, if applicable, for the original study on which the present article is based | 13 | *Conflicts of Interest None declared.* |

Table S93. Quality Evaluation for Included Studies Using STROBE

17. STROBE Statement—checklist of items that should be included in reports of observational studies

| | Item No. | Recommendation | Page No. | Relevant text from manuscript |
|---|---|---|---|---|
| **Title and abstract** | 1 | (*a*) Indicate the study's design with a commonly used term in the title or the abstract | 1 | misinformation and quality of information |
| | | (*b*) Provide in the abstract an informative and balanced summary of what was done and what was found | NA | |
| **Introduction** | | | | |
| Background/rationale | 2 | Explain the scientific background and rationale for the investigation being reported | 1 | *Paragraph 3*<br>*"We have previously reported a significant amount of biased and misinformative … plays in disseminating quality PCa information"* |
| Objectives | 3 | State specific objectives, including any prespecified hypotheses | 1 | *Our objective was to review the nature and quality of TikTok videos about PCa using validated metrics.* |
| **Methods** | | | | |
| Study design | 4 | Present key elements of study design early in the paper | 1 | Cross-sectional content analysis |
| Setting | 5 | Describe the setting, locations, and relevant dates, including periods of recruitment, exposure, follow-up, and data collection | 1 | *all TikTok videos (n = 65) with the hashtag #prostatecancer between 12 June 2016 and 30 June 2020* |
| Participants | 6 | (*a*) *Cohort study*—Give the eligibility criteria, and the sources and methods of selection of participants. Describe methods of follow-up<br>*Case-control study*—Give the eligibility criteria, and the sources and methods of case ascertainment and control selection. Give the rationale for the choice of cases and controls<br>*Cross-sectional study*—Give the eligibility criteria, and the sources and methods of selection of participants | 1 | *Ten were excluded (seven private and three non-English), leaving 55 for analysis.* |
| | | (*b*) *Cohort study*—For matched studies, give matching criteria and number of exposed and unexposed | | |

| | | | | |
|---|---|---|---|---|
| | | *Case-control study*—For matched studies, give matching criteria and the number of controls per case | | |
| Variables | 7 | Clearly define all outcomes, exposures, predictors, potential confounders, and effect modifiers. Give diagnostic criteria, if applicable | 1 | *video length, number of views and comments, associated description, and hashtags were collected* |
| Data sources/ measurement | 8* | For each variable of interest, give sources of data and details of methods of assessment (measurement). Describe comparability of assessment methods if there is more than one group | *1* | *Video from TikTok* |
| Bias | 9 | Describe any efforts to address potential sources of bias | 1 | *Inter-rater reliability was 99.6% between two coders with PCa expertise.* |
| Study size | 10 | Explain how the study size was arrived at | 1 | 55 |

| | | | | |
|---|---|---|---|---|
| Quantitative variables | 11 | Explain how quantitative variables were handled in the analyses. If applicable, describe which groupings were chosen and why | 1 | *CERN quality criteria misinformation, using a published five-point Likert scale* |
| Statistical methods | 12 | (*a*) Describe all statistical methods, including those used to control for confounding | 1 | reviewers annotated the topic and target audience, as well as the perceived demographics descriptive statistics |
| | | (*b*) Describe any methods used to examine subgroups and interactions | NA | |
| | | (*c*) Explain how missing data were addressed | NA | |
| | | (*d*) *Cohort study*—If applicable, explain how loss to follow-up was addressed<br>*Case-control study*—If applicable, explain how matching of cases and controls was addressed<br>*Cross-sectional study*—If applicable, describe analytical methods taking account of sampling strategy | NA | |
| | | (*e*) Describe any sensitivity analyses | NA | |
| **Results** | | | | |
| Participants | 13* | (a) Report numbers of individuals at each stage of study—eg numbers potentially eligible, examined for eligibility, confirmed eligible, included in the study, completing follow-up, and analysed | 1 | *The 55 videos comprised a total of 134 944 individual views. The median length was 17.7 s with* |

| | | | | |
|---|---|---|---|---|
| | | | | *202 views, 15 likes, and 0 comments.* |
| | | (b) Give reasons for non-participation at each stage | NA | |
| | | (c) Consider use of a flow diagram | NA | |
| Descriptive data | 14* | (a) Give characteristics of study participants (eg demographic, clinical, social) and information on exposures and potential confounders | 1 | Paragraph 5<br>“*Content was primarily … patient (26%), for profit companies (4%), and patients (6%).”* |
| | | (b) Indicate number of participants with missing data for each variable of interest | NA | |
| | | (c) *Cohort study*—Summarise follow-up time (eg, average and total amount) | NA | |
| Outcome data | 15* | *Cohort study*—Report numbers of outcome events or summary measures over time | *1* | *“The median scores on PEMAT were 75% … advertisements in the comments.”* |
| | | *Case-control study*—Report numbers in each exposure category, or summary measures of exposure | *NA* | |
| | | *Cross-sectional study*—Report numbers of outcome events or summary measures | *NA* | |
| Main results | 16 | (*a*) Give unadjusted estimates and, if applicable, confounder-adjusted estimates and their precision (eg, 95% confidence interval). Make clear which confounders were adjusted for and why they were included | 1 | *Fifty-four videos (98.2%) were moderate to poor quality, accounting for 134 752 or >99% of total views* |
| | | (*b*) Report category boundaries when continuous variables were categorized | NA | |
| | | (*c*) If relevant, consider translating estimates of relative risk into absolute risk for a meaningful time period | NA | |

| | | | | |
|---|---|---|---|---|
| Other analyses | 17 | Report other analyses done—eg analyses of subgroups and interactions, and sensitivity analyses | NA | |
| **Discussion** | | | | |
| Key results | 18 | Summarise key results with reference to study objectives | 2 | *Most posts lacked substantive information for health consumers. Of the few with educational information, about half contained significant misinformation.* |

| | | | | |
|---|---|---|---|---|
| Limitations | 19 | Discuss limitations of the study, taking into account sources of potential bias or imprecision. Discuss both direction and magnitude of any potential bias | 2 | *the number of videos analysed was low, indicating that TikTok is not currently a common platform for dissemination of PCa information* |
| Interpretation | 20 | Give a cautious overall interpretation of results considering objectives, limitations, multiplicity of analyses, results from similar studies, and other relevant evidence | 2 | *TikTok videos about PCa are primarily casual content that may raise awareness but do not provide high quality educational material.* |
| Generalisability | 21 | Discuss the generalisability (external validity) of the study results | 1 | *We found that that given the format of TikTok videos, it was difficult to apply pre-existing validated measures meant for longer audiovisual content that is intended to provide patient education.* |
| **Other information** | | | | |
| Funding | 22 | Give the source of funding and the role of the funders for the present study and, if applicable, for the original study on which the present article is based | 2 | *This study was supported by the Prostate Cancer Foundation and a Department of Defense Health Disparity Research Award to Stacy Loeb.* |

Table S94. Quality Evaluation for Included Studies Using STROBE

18. STROBE Statement—checklist of items that should be included in reports of observational studies

| | Item No. | Recommendation | Page No. | Relevant text from manuscript |
|---|---|---|---|---|
| **Title and abstract** | 1 | (*a*) Indicate the study's design with a commonly used term in the title or the abstract | 1 | Evaluation of the Quality and Reliability |
| | | (*b*) Provide in the abstract an informative and balanced summary of what was done and what was found | 2 | Materials and methods, Results<br>"*We retrieved 167 videos on bladder, prostate … to use professional terms most (mean = 5.28 words).*" |
| **Introduction** | | | | |
| Background/rationale | 2 | Explain the scientific background and rationale for the investigation being reported | 2 | *Notwithstanding the rich and varied digital resources on TikTok, its role in healthcare promotion remains inceptive.* |
| Objectives | 3 | State specific objectives, including any prespecified hypotheses | 2 | *This study attempted to evaluate the functional quality and reliability of GUCa-related videos on TikTok and offer some facts-based advice on better public health engagement.* |
| **Methods** | | | | |
| Study design | 4 | Present key elements of study design early in the paper | 2 | Cross-sectional |
| Setting | 5 | Describe the setting, locations, and relevant dates, including periods of recruitment, exposure, follow-up, and data collection | 2 | *A comprehensive search was conducted on TikTok (both International and Chinese versions) from 13th to 20th September 2021* |
| Participants | 6 | (*a*) *Cohort study*—Give the eligibility criteria, and the sources and methods of selection of participants. Describe methods of follow-up<br>*Case-control study*—Give the eligibility criteria, and the sources and methods of case ascertainment and control selection. Give the rationale for the choice of cases and controls | 2 | *A total of 167 videos were retrieved. Further evaluations were made to exclude videos with potential commercial promotions,*<br>*incomplete content, linguistic barriers, or copyright disputes. It should be noted* |

| | | | | |
|---|---|---|---|---|
| | | *Cross-sectional study*—Give the eligibility criteria, and the sources and methods of selection of participants | | *that some excluded commercial videos contained disinformation, such as exaggerating the efficacy of products and creating anxiety. After preliminary screening, 61 videos were qualified as candidates.* |
| | | (*b*) *Cohort study*—For matched studies, give matching criteria and number of exposed and unexposed<br>*Case-control study*—For matched studies, give matching criteria and the number of controls per case | | |
| Variables | 7 | Clearly define all outcomes, exposures, predictors, potential confounders, and effect modifiers. Give diagnostic criteria, if applicable | 2 | *Objective data were collected, including the length and descriptions of each video, their hashtags, number of views/likes/comments, forms of expression, and the uploader's profile. functional quality* |
| Data sources/ measurement | 8* | For each variable of interest, give sources of data and details of methods of assessment (measurement). Describe comparability of assessment methods if there is more than one group | *2* | *Videos are from TikTok* |
| Bias | 9 | Describe any efforts to address potential sources of bias | 2 | *We erased all histories and settings on a smartphone to avoid potential pre-buffered cache-induced directional information recommendations. The location services were enabled while the activity tracking feature was disabled, and the language was set to Simplified Chinese by default to simulate daily life scenarios.*<br>*All sample videos were renamed and de-identified.* |
| Study size | 10 | Explain how the study size was arrived at | 2 | 61 videos |

| | | | | |
|---|---|---|---|---|
| Quantitative variables | 11 | Explain how quantitative variables were handled in the analyses. If applicable, describe which groupings were chosen and why | 2-3 | *HONCode scoring, hexagonal chart , and the DISCERN, ANOVA* |
| Statistical methods | 12 | (*a*) Describe all statistical methods, including those used to control for confounding | 4 | *descriptive stats, and post hoc tests.* |
| | | (*b*) Describe any methods used to examine subgroups and interactions | NA | |
| | | (*c*) Explain how missing data were addressed | NA | |
| | | (*d*) *Cohort study*—If applicable, explain how loss to follow-up was addressed<br>*Case-control study*—If applicable, explain how matching of cases and controls was addressed<br>*Cross-sectional study*—If applicable, describe analytical methods taking account of sampling strategy | NA | |
| | | (*e*) Describe any sensitivity analyses | NA | |
| **Results** | | | | |
| Participants | 13* | (a) Report numbers of individuals at each stage of study—eg numbers potentially eligible, examined for eligibility, confirmed eligible, included in the study, completing follow-up, and analysed | 4 | Results paragraph 1<br>"*We performed the global search … Chinese while four (6.56%) were in English.*" |
| | | (b) Give reasons for non-participation at each stage | NA | |
| | | (c) Consider use of a flow diagram | 3 | |
| Descriptive data | 14* | (a) Give characteristics of study participants (eg demographic, clinical, social) and information on exposures and potential confounders | 4 | Results paragraph 2-3<br>"*All videos were classified into two types … Detailed characteristics of all sample videos are presented in Table 1.*" |
| | | (b) Indicate number of participants with missing data for each variable of interest | NA | |
| | | (c) *Cohort study*—Summarise follow-up time (eg, average and total amount) | NA | |
| Outcome data | 15* | *Cohort study*—Report numbers of outcome events or summary measures over time | | |
| | | *Case-control study*—Report numbers in each exposure category, or summary measures of exposure | | |

| | | | | |
|---|---|---|---|---|
| | | *Cross-sectional study*—Report numbers of outcome events or summary measures | *4* | Video content and information paragraph 1-5 |
| Main results | 16 | (*a*) Give unadjusted estimates and, if applicable, confounder-adjusted estimates and their precision (eg, 95% confidence interval). Make clear which confounders were adjusted for and why they were included | 4 | *Twenty-two videos (36.07%) had obvious misinformation the imbalance of information on TikTok* |
| | | (*b*) Report category boundaries when continuous variables were categorized | NA | |
| | | (*c*) If relevant, consider translating estimates of relative risk into absolute risk for a meaningful time period | NA | |
| Other analyses | 17 | Report other analyses done—eg analyses of subgroups and interactions, and sensitivity analyses | 4 | *Further univariate analysis of variance indicated significant differences in the reliability …* |
| **Discussion** | | | | |
| Key results | 18 | Summarise key results with reference to study objectives | 6 | *Amongst 61 evaluatedsample videos, 22 (36.07%)had unequivocal misinformation, which was in line with previous data (7, 15, 18), indicating that the correctness of online health promotion videos might not have been improved in recent years* |
| Limitations | 19 | Discuss limitations of the study, taking into account sources of potential bias or imprecision. Discuss both direction and magnitude of any potential bias | 7 | "*Several limitations should be noted …*" |
| Interpretation | 20 | Give a cautious overall interpretation of results considering objectives, limitations, multiplicity of analyses, results from similar studies, and other relevant evidence | 8 | *To better deliver the information to the broad public, medical practitioners may need to strengthen their cooperation with media agencies and avoid turgid technical terminologies in their posts.* |
| Generalisability | 21 | Discuss the generalisability (external validity) of the study results | 7 | *High-quality videos in other languages might be omitted. Studies on videos in other languages are welcomed, as they could contribute a more profound and comprehensive picture.* |

| | | | |
|---|---|---|---|
| **Other information** | | | |
| Funding | 22 | Give the source of funding and the role of the funders for the present study and, if applicable, for the original study on which the present article is based | NA |

Table S95. Quality Evaluation for Included Studies Using STROBE

19.STROBE Statement—checklist of items that should be included in reports of observational studies

| | Item No. | Recommendation | Page No. | Relevant text from manuscript |
|---|---|---|---|---|
| **Title and abstract** | 1 | (*a*) Indicate the study's design with a commonly used term in the title or the abstract | 1 | Quality and accuracy analysis |
| | | (*b*) Provide in the abstract an informative and balanced summary of what was done and what was found | 1 | Methods and Results<br>"*The terms "gastric cancer" was searched … Chinese (47.3%) were the most frequently covered topic.*" |
| **Introduction** | | | | |
| Background/rationale | 2 | Explain the scientific background and rationale for the investigation being reported | 2 | *As the fastest-growing social media applications, their potential as educational tools for health-related content cannot be overlooked. However, videos posted on social media are not peer-reviewed and are commonly ranked according to popularity.* |
| Objectives | 3 | State specific objectives, including any prespecified hypotheses | 2 | *this study aims to assess the content, accuracy, and completeness of social media about gastric cancer on TikTok in multiple countries. We also want to share our thoughts on important future directions for managing social media for gastric cancer.* |
| **Methods** | | | | |
| Study design | 4 | Present key elements of study design early in the paper | 2 | Cross-sectional study |
| Setting | 5 | Describe the setting, locations, and relevant dates, including periods of recruitment, exposure, follow-up, and data collection | 2 | *queried the TikTok and Douyin mobile application on August 17, 2021* |

| | | | | |
|---|---|---|---|---|
| Participants | 6 | (*a*) *Cohort study*—Give the eligibility criteria, and the sources and methods of selection of participants. Describe methods of follow-up<br>*Case-control study*—Give the eligibility criteria, and the sources and methods of case ascertainment and control selection. Give the rationale for the choice of cases and controls<br>*Cross-sectional study*—Give the eligibility criteria, and the sources and methods of selection of participants | 2 | *The first 100 most popular videos were gathered and analyzed. The languages were limited to English and Japanese in TikTok and Chinese in Doyin. Videos that were duplicated, had no sound and were not directly related to gastric cancer were excluded.* |
| | | (*b*) *Cohort study*—For matched studies, give matching criteria and number of exposed and unexposed<br>*Case-control study*—For matched studies, give matching criteria and the number of controls per case | NA | |
| Variables | 7 | Clearly define all outcomes, exposures, predictors, potential confounders, and effect modifiers. Give diagnostic criteria, if applicable | 2 | *Quality*<br>*The videos were further categorized as useful or useless according to educational content. (QUEST) and DISCERN, Completeness checklist for evaluating gastric cancer video quality* |
| Data sources/ measurement | 8* | For each variable of interest, give sources of data and details of methods of assessment (measurement). Describe comparability of assessment methods if there is more than one group | *2* | *Videos are from TikTok and Douyin* |
| Bias | 9 | Describe any efforts to address potential sources of bias | 2 | *Videos that were duplicated, had no sound and were not directly related to gastric cancer were excluded.*<br>*Each video was assessed for content quality by two independent gastroenterological surgeons. All coders had studied in Japan at least one year and had sufficient experience in the diagnosis and management of gastric cancer. Any disagreements* |

| | | | | |
|---|---|---|---|---|
| | | | | *were discussed until a consensus was reached* |
| Study size | 10 | Explain how the study size was arrived at | 3 | 240 videos |
| Quantitative variables | 11 | Explain how quantitative variables were handled in the analyses. If applicable, describe which groupings were chosen and why | 2 | *(QUEST) and DISCERN* |
| Statistical methods | 12 | (*a*) Describe all statistical methods, including those used to control for confounding | 3 | *frequencies (n) and percentages (%) for categorical variables and means or medians (standard deviations or ranges) for continuous and ordinal variables, respectively. The one-way ANOVA was used to compare the differences between the groups.* |
| | | (*b*) Describe any methods used to examine subgroups and interactions | 3 | *The one-way ANOVA was used to compare the differences between the groups* |
| | | (*c*) Explain how missing data were addressed | NA | |
| | | (*d*) *Cohort study*—If applicable, explain how loss to follow-up was addressed<br>*Case-control study*—If applicable, explain how matching of cases and controls was addressed<br>*Cross-sectional study*—If applicable, describe analytical methods taking account of sampling strategy | NA | |
| | | (*e*) Describe any sensitivity analyses | NA | |
| **Results** | | | | |
| Participants | 13* | (a) Report numbers of individuals at each stage of study—eg numbers potentially eligible, examined for eligibility, confirmed eligible, included in the study, completing follow-up, and analysed | 3 | *Video selection process* |
| | | (b) Give reasons for non-participation at each stage | | |
| | | (c) Consider use of a flow diagram | 3 | |
| Descriptive data | 14* | (a) Give characteristics of study participants (eg demographic, clinical, social) and information on exposures and potential confounders | 3 | *Video characteristics* |

| | | | | |
|---|---|---|---|---|
| | | (b) Indicate number of participants with missing data for each variable of interest | | |
| | | (c) *Cohort study*—Summarise follow-up time (eg, average and total amount) | | |
| Outcome data | 15* | *Cohort study*—Report numbers of outcome events or summary measures over time | *3* | *Information reliability Educational content* |
| | | *Case-control study*—Report numbers in each exposure category, or summary measures of exposure | | |
| | | *Cross-sectional study*—Report numbers of outcome events or summary measures | | |
| Main results | 16 | (*a*) Give unadjusted estimates and, if applicable, confounder-adjusted estimates and their precision (eg, 95% confidence interval). Make clear which confounders were adjusted for and why they were included | 3 | *Among the useful videos, the videos published in Chinese had the highest QUEST ($p < 0.05$) and DISCERN scores ($p < 0.05$), followed by those published in Japanese.* |
| | | (*b*) Report category boundaries when continuous variables were categorized | | |
| | | (*c*) If relevant, consider translating estimates of relative risk into absolute risk for a meaningful time period | | |

| | | | | |
|---|---|---|---|---|
| Other analyses | 17 | Report other analyses done—eg analyses of subgroups and interactions, and sensitivity analyses | 2 | Completeness checklist for evaluating gastric cancer video quality |

| | | | | |
|---|---|---|---|---|
| Key results | 18 | Summarise key results with reference to study objectives | 6 | *The average scores for completeness in our study were not high. Most of the analyzed videos only included one or two categories.* |
| Limitations | 19 | Discuss limitations of the study, taking into account sources of potential bias or imprecision. Discuss both direction and magnitude of any potential bias | 6 | *There are limitations to our study….* |
| Interpretation | 20 | Give a cautious overall interpretation of results considering objectives, limitations, multiplicity of analyses, results from similar studies, and other relevant evidence | 7 | *TikTok in English and Japanese might not fully meet the gastric cancer information needs of public, but Douyin in Chinese was the opposite.* |
| Generalisability | 21 | Discuss the generalisability (external validity) of the study results | 6 | *content in other platforms may show different results and conclusions the study data were* |

| | | | | |
|---|---|---|---|---|
| | | | | *collected and may change due to new videos being uploaded or removed with time.* |
| **Other information** | | | | |
| Funding | 22 | Give the source of funding and the role of the funders for the present study and, if applicable, for the original study on which the present article is based | 7 | *The study was supported by Key Characteristic Disease …* |

Table S96. Quality Evaluation for Included Studies Using STROBE

20.STROBE Statement—checklist of items that should be included in reports of observational studies

| | Item No. | Recommendation | Page No. | Relevant text from manuscript |
|---|---|---|---|---|
| **Title and abstract** | 1 | (*a*) Indicate the study's design with a commonly used term in the title or the abstract | 1 | Content analysis |
| | | (*b*) Provide in the abstract an informative and balanced summary of what was done and what was found | 1 | "*To characterize the Twitter conversation … while 14.6% claimed to be confused by them.*" |
| **Introduction** | | | | |
| Background/rationale | 2 | Explain the scientific background and rationale for the investigation being reported | 2 | *"Twitter, a microblogging site in which users … and update clinicians and patients"*<br>*"the type and quality of references that … breast cancer screening, are not well understood."* |
| Objectives | 3 | State specific objectives, including any prespecified hypotheses | 2 | "*In order to better understand how … about the guidelines and current state of evidence* " |
| **Methods** | | | | |
| Study design | 4 | Present key elements of study design early in the paper | 2 | Cross-sectional content analysis |
| Setting | 5 | Describe the setting, locations, and relevant dates, including periods of recruitment, exposure, follow-up, and data collection | 2 | *from Twitter between the dates of 5 November 2015 to 11 December 2015* |
| Participants | 6 | (*a*) *Cohort study*—Give the eligibility criteria, and the sources and methods of selection of participants. Describe methods of follow-up<br>*Case-control study*—Give the eligibility criteria, and the sources and methods of case ascertainment and control selection. Give the rationale for the choice of cases and controls<br>*Cross-sectional study*—Give the eligibility criteria, and the sources and methods of selection of participants | 2 | *tweets containing one or more of the hashtags "mammo", "mammography", "mammogram" and "mammograms"* |
| | | (*b*) *Cohort study*—For matched studies, give matching criteria and number of exposed and unexposed | NA | |

| | | | | |
|---|---|---|---|---|
| | | *Case-control study*—For matched studies, give matching criteria and the number of controls per case | | |
| Variables | 7 | Clearly define all outcomes, exposures, predictors, potential confounders, and effect modifiers. Give diagnostic criteria, if applicable | 2 | *including user type, gender, whether or not the user was likely a woman aged 40 years or older, and location.*<br>*data on language; type of tweet; number of retweets; number of likes; tweet content; and name of guideline, journal, or news source provided in the tweet.* |
| Data sources/ measurement | 8* | For each variable of interest, give sources of data and details of methods of assessment (measurement). Describe comparability of assessment methods if there is more than one group | *2* | *a data abstraction form with qualitative and quantitative fields* |
| Bias | 9 | Describe any efforts to address potential sources of bias | 2 | *two researchers (AN, TB) independently analyzed 25 tweets.*<br>*Frequent communication occurred between coders throughout the process in the event of uncertainties or questions regarding the standard of abstraction so as to maximize inter-rater reliability* |
| Study size | 10 | Explain how the study size was arrived at | 3 | *1345 tweets* |

| | | | | |
|---|---|---|---|---|
| Quantitative variables | 11 | Explain how quantitative variables were handled in the analyses. If applicable, describe which groupings were chosen and why | 2 | *number of retweets; number of likes* |
| Statistical methods | 12 | (*a*) Describe all statistical methods, including those used to control for confounding | 2 | *"Descriptive statistics were calculated in order to … (ANOVA) with Bonferroni correction was utilized.* |
| | | (*b*) Describe any methods used to examine subgroups and interactions | NA | |
| | | (*c*) Explain how missing data were addressed | NA | |
| | | (*d*) *Cohort study*—If applicable, explain how loss to follow-up was addressed | NA | |

| | | | | |
|---|---|---|---|---|
| | | *Case-control study*—If applicable, explain how matching of cases and controls was addressed<br>*Cross-sectional study*—If applicable, describe analytical methods taking account of sampling strategy | | |
| | | (e) Describe any sensitivity analyses | | |
| **Results** | | | | |
| Participants | 13* | (a) Report numbers of individuals at each stage of study—eg numbers potentially eligible, examined for eligibility, confirmed eligible, included in the study, completing follow-up, and analysed | 3 | *A total of 1345 (1.82%) out of the 73,487 breast cancer related tweets were regarding mammography, and these 1345 tweets originated from 995 unique users.* |
| | | (b) Give reasons for non-participation at each stage | NA | |
| | | (c) Consider use of a flow diagram | 3 | |
| Descriptive data | 14* | (a) Give characteristics of study participants (eg demographic, clinical, social) and information on exposures and potential confounders | 3 | "*The largest user type was Bnon-healthcare^ making … users whose locations were provided (Table 1).*" |
| | | (b) Indicate number of participants with missing data for each variable of interest | NA | |
| | | (c) *Cohort study*—Summarise follow-up time (eg, average and total amount) | NA | |
| Outcome data | 15* | *Cohort study*—Report numbers of outcome events or summary measures over time | | |
| | | *Case-control study*—Report numbers in each exposure category, or summary measures of exposure | | |
| | | *Cross-sectional study*—Report numbers of outcome events or summary measures | *4* | *Tweet Characteristics*<br>*Reference Usage*<br>*Guideline Tweets*<br>*Personal Tweets* |
| Main results | 16 | (*a*) Give unadjusted estimates and, if applicable, confounder-adjusted estimates and their precision (eg, 95% confidence interval). Make clear which confounders were adjusted for and why they were included | 5 | *Tweets that explicitly provided references were retweeted more (1.25 vs. 0.53, $p < 0.001$) on average compared to those that did not provide them;* |
| | | (*b*) Report category boundaries when continuous variables were categorized | NA | |

| | | | | |
|---|---|---|---|---|
| | | (*c*) If relevant, consider translating estimates of relative risk into absolute risk for a meaningful time period | NA | |
| Other analyses | 17 | Report other analyses done—eg analyses of subgroups and interactions, and sensitivity analyses | 5 | Subgroups of user type |
| Key results | 18 | Summarise key results with reference to study objectives | 6 | *The largest user type tweeting about mammography was Bnon-healthcare^ users (32.5%), indicating the general population's importance in the dialog on breast cancer screening* |
| Limitations | 19 | Discuss limitations of the study, taking into account sources of potential bias or imprecision. Discuss both direction and magnitude of any potential bias | 7 | *The key limitation of this study is the subjectivity …* |
| Interpretation | 20 | Give a cautious overall interpretation of results considering objectives, limitations, multiplicity of analyses, results from similar studies, and other relevant evidence | 7 | *The non-health care user's substantial role in tweeting about mammography, the lack of appropriate scientific support in many of their claims, and the overall low approval for the current ACS guidelines indicate that the potential of social media platforms to widely disseminate accurate* |
| Generalisability | 21 | Discuss the generalisability (external validity) of the study results | 7 | *limited to Twitter users and English-language tweets.* |
| **Other information** | | | | |
| Funding | 22 | Give the source of funding and the role of the funders for the present study and, if applicable, for the original study on which the present article is based | NA | |

Table S97. Quality Evaluation for Included Studies Using STROBE

21.STROBE Statement—checklist of items that should be included in reports of observational studies

| | Item No. | Recommendation | Page No. | Relevant text from manuscript |
|---|---|---|---|---|
| **Title and abstract** | 1 | (*a*) Indicate the study's design with a commonly used term in the title or the abstract | 1 | *retrospective content analysis* |
| | | (*b*) Provide in the abstract an informative and balanced summary of what was done and what was found | 1 | "*We conducted a retrospective content analysis…5.44 (95% CI 5.33–5.56) times the incidence rate of retweet.*" |
| **Introduction** | | | | |
| Background/rationale | 2 | Explain the scientific background and rationale for the investigation being reported | 2 | *Paragraph 2-last paragraph*<br>*"Social media use is particularly … an emerging parental barrier surrounding vaccine hesitancy."* |
| Objectives | 3 | State specific objectives, including any prespecified hypotheses | 3 | Paragraph 2<br>"*the objectives of this study were to identify … in order to identify potential targets for corrective intervention.*" |
| **Methods** | | | | |
| Study design | 4 | Present key elements of study design early in the paper | 3 | *retrospective content analysis* |
| Setting | 5 | Describe the setting, locations, and relevant dates, including periods of recruitment, exposure, follow-up, and data collection | 3 | *The search was done through the retrieval of Tweets posted between December 15, 2019, through March 31, 2020, from the Twitter application program interface (API) stream.* |
| Participants | 6 | (*a*) *Cohort study*—Give the eligibility criteria, and the sources and methods of selection of participants. Describe methods of follow-up<br>*Case-control study*—Give the eligibility criteria, and the sources and methods of case ascertainment and control selection. Give the rationale for the choice of cases and controls | 3 | *applied an English language filter, a filter to exclude retweets, and used the hashtag '#HPV' to perform a keyword search.* |

| | | | | |
|---|---|---|---|---|
| | | *Cross-sectional study*—Give the eligibility criteria, and the sources and methods of selection of participants | | *Posts eligible for inclusion had to contain English text; links to external sites and text contained within images in the Tweets were not evaluated.* |
| | | (*b*) *Cohort study*—For matched studies, give matching criteria and number of exposed and unexposed<br><br>*Case-control study*—For matched studies, give matching criteria and the number of controls per case | | |
| Variables | 7 | Clearly define all outcomes, exposures, predictors, potential confounders, and effect modifiers. Give diagnostic criteria, if applicable | 3-5 | *Concern, confidence, type of concern, gender or age group, if the concern was presented as a personal narrative/story, Injury to child, Flagging, Audience engagement outcome variable of interest was audience engagement* |
| Data sources/ measurement | 8* | For each variable of interest, give sources of data and details of methods of assessment (measurement). Describe comparability of assessment methods if there is more than one group | *3* | *Tweets posted from the Twitter application program interface (API) stream.*<br><br>*All coding was completed using REDCap electronic data capture tools* |
| Bias | 9 | Describe any efforts to address potential sources of bias | 3 | *Amongst the four annotators, a Kappa score of 0.91 was achieved, indicating almost perfect agreement between individual annotators. After the process of developing the codebook and determining interrater reliability was complete, each of the four annotators independently coded* |
| Study size | 10 | Explain how the study size was arrived at | 3 | *randomly selected 4,258 tweets (80%) out of the remaining 5,341 for manual annotation.* |

| | | | | |
|---|---|---|---|---|
| | | | | *After data cleaning, the final sample size was n = 3876 posts by 1780 unique users (n = 3710 unique text posts).* |
| Quantitative variables | 11 | Explain how quantitative variables were handled in the analyses. If applicable, describe which groupings were chosen and why | 5 | *Retweet count, Reply count, and Favorite count.* |
| Statistical methods | 12 | (*a*) Describe all statistical methods, including those used to control for confounding | 6 | *calculated the mean (SD) of the summed audience engagement measure (Retweet count + Reply count + Favorites count) and assessed the audience engagement metrics across categorical variables.*<br><br>*A negative binomial regression by building a GLM model* |
| | | (*b*) Describe any methods used to examine subgroups and interactions | 6 | *misinformation posts, we controlled for gender, age, and personal narrative in a multivariable model.* |
| | | (*c*) Explain how missing data were addressed | 5-6 | *Among the concern posts, 1% (n = 10) were missing a combination of gender, age, and/or personal narrative data. These posts were excluded from the analyses on these variables.* |
| | | (*d*) *Cohort study*—If applicable, explain how loss to follow-up was addressed<br><br>*Case-control study*—If applicable, explain how matching of cases and controls was addressed<br><br>*Cross-sectional study*—If applicable, describe analytical methods taking account of sampling strategy | NA | |
| | | (*e*) Describe any sensitivity analyses | 6 | *We conducted sensitivity analyses for by creating indicator variables for each month that the Tweets were posted and including them in the final model.* |
| **Results** | | | | |

| | | | | |
|---|---|---|---|---|
| Participants | 13* | (a) Report numbers of individuals at each stage of study—eg numbers potentially eligible, examined for eligibility, confirmed eligible, included in the study, completing follow-up, and analysed | 5 | *Data cleaning…* |
| | | (b) Give reasons for non-participation at each stage | 5-6 | *Data cleaning and Missing and outliers* |
| | | (c) Consider use of a flow diagram | NA | |
| Descriptive data | 14* | (a) Give characteristics of study participants (eg demographic, clinical, social) and information on exposures and potential confounders | 6 | *Frequency of misinformation* |
| | | (b) Indicate number of participants with missing data for each variable of interest | NA | |
| | | (c) *Cohort study*—Summarise follow-up time (eg, average and total amount) | NA | |
| Outcome data | 15* | *Cohort study*—Report numbers of outcome events or summary measures over time | | |
| | | *Case-control study*—Report numbers in each exposure category, or summary measures of exposure | | |
| | | *Cross-sectional study*—Report numbers of outcome events or summary measures | *6* | *Audience engagement* |
| Main results | 16 | (*a*) Give unadjusted estimates and, if applicable, confounder-adjusted estimates and their precision (eg, 95% confidence interval). Make clear which confounders were adjusted for and why they were included | 8 | *Certain types of misinformation were less likely to be retweeted; Tweets containing misinformation about Pharma [IRR 0.68 (95% CI 0.63–0.74)], common concerns from the literature [IRR 0.42 (95% CI 0.31–0.56)], and government [IRR 0.40 (95% 0.37–0.43)] were all less likely to be retweeted* |
| | | (*b*) Report category boundaries when continuous variables were categorized | NA | |
| | | (*c*) If relevant, consider translating estimates of relative risk into absolute risk for a meaningful time period | NA | |

| | | | | |
|---|---|---|---|---|
| Other analyses | 17 | Report other analyses done—eg analyses of subgroups and interactions, and sensitivity analyses | 5-7 | Sensitivity analysis,<br>Analysis by *Types of misinformation, gender, age, personal narrative* |

| | | | | |
|---|---|---|---|---|
| Key results | 18 | Summarise key results with reference to study objectives | 8 | *In our review of HPV-related tweets posted in a three and a half-month period, nearly a quarter contained misinformation about the HPV vaccine, with the most prevalent category of misinformation pertaining to adverse health effects from vaccination.* |
| Limitations | 19 | Discuss limitations of the study, taking into account sources of potential bias or imprecision. Discuss both direction and magnitude of any potential bias | 11 | *We note several limitations to our study…* |
| Interpretation | 20 | Give a cautious overall interpretation of results considering objectives, limitations, multiplicity of analyses, results from similar studies, and other relevant evidence | 11 | *Given that many parents rely on social media for health-related information, future interventions, such as those that prebunk misinformation to prevent the harmful effects on vaccination acceptance are urgently needed.* |
| Generalisability | 21 | Discuss the generalisability (external validity) of the study results | 11 | *restricted our analysis to Twitter data. Twitter is not the most commonly used social media platform among parents.*<br><br>*we limited our search term to "hpv" and the time period to three and a half months thus further limiting the scope of and generalizability of our study* |
| **Other information** | | | | |
| Funding | 22 | Give the source of funding and the role of the funders for the present study and, if applicable, for the original study on which the present article is based | 11 | *Dr. Kornides and this research were supported by an award from the National Institute of Child Health and Human Development and Office of Women's Research* |

Table S98. Quality Evaluation for Included Studies Using STROBE

22.STROBE Statement—checklist of items that should be included in reports of observational studies

| | Item No. | Recommendation | Page No. | Relevant text from manuscript |
|---|---|---|---|---|
| **Title and abstract** | 1 | (*a*) Indicate the study's design with a commonly used term in the title or the abstract | 1 | Retrospective Study |
| | | (*b*) Provide in the abstract an informative and balanced summary of what was done and what was found | 1 | "*Using the Twitter app programming interface … information (median 35.0, IQR 0-502 vs 8.0, IQR 0-2197; P=.002).*" |
| **Introduction** | | | | |
| Background/rationale | 2 | Explain the scientific background and rationale for the investigation being reported | 2 | *In Japan, fewer hospitals and clinics use social media in comparison with other countries because some content disseminated by these medical institutions' conflicts with information in medical advertising. This state of affairs can lead to an increased prevalence of inaccurate information on social media platforms.* |
| Objectives | 3 | State specific objectives, including any prespecified hypotheses | 2 | *The objective of our research was to examine the prevalence of misinformation and harmful cancer-related content on Twitter and to further clarify attributes that increase the likelihood of the dissemination of such content in Japan. The findings of our study can assist decision-making among individuals diagnosed with cancer.* |
| **Methods** | | | | |
| Study design | 4 | Present key elements of study design early in the paper | 2 | Retrospective cross-sectional |

| | | | | |
|---|---|---|---|---|
| Setting | 5 | Describe the setting, locations, and relevant dates, including periods of recruitment, exposure, follow-up, and data collection | 2 | *Tweet data were retrospectively collected from August 2022 to September 2022 by querying the Twitter app programming interface with the keyword "cancer" in Japanese* |
| Participants | 6 | (*a*) *Cohort study*—Give the eligibility criteria, and the sources and methods of selection of participants. Describe methods of follow-up<br>*Case-control study*—Give the eligibility criteria, and the sources and methods of case ascertainment and control selection. Give the rationale for the choice of cases and controls<br>*Cross-sectional study*—Give the eligibility criteria, and the sources and methods of selection of participants | 2 | *The eligibility criteria were original tweets or retweets with comments with the following information…* |
| | | (*b*) *Cohort study*—For matched studies, give matching criteria and number of exposed and unexposed<br>*Case-control study*—For matched studies, give matching criteria and the number of controls per case | | |
| Variables | 7 | Clearly define all outcomes, exposures, predictors, potential confounders, and effect modifiers. Give diagnostic criteria, if applicable | 2 | *The Twitter data set contained tweet-level data including the date or time, account's screen name, tweet description, number of the account's followers, "likes" count, and retweet count at the time of data acquisition.*<br>*claims in each tweet and completed 4-question assessments adapted from assessments of factuality and credibility* |
| Data sources/ measurement | 8* | For each variable of interest, give sources of data and details of methods of assessment (measurement). Describe comparability of assessment methods if there is more than one group | *2* | *Tweet data were from Twitter app*<br>*Two independent reviewers, who were physicians specializing in oncology and with a clinical practice in a cancer center or university hospital in Japan, reviewed the medical claims in each* |

| | | | | |
|---|---|---|---|---|
| | | | | *tweet and completed 4-question assessments adapted from assessments of factuality and credibility* |
| Bias | 9 | Describe any efforts to address potential sources of bias | 2 | *The reviewers provided the reasons for selecting "Probably harmful" and "Definitely harmful" for the content, as follows*<br><br>*The level of agreement between raters was assessed using the Cohen κ coefficient* |
| Study size | 10 | Explain how the study size was arrived at | 3 | *We chose the 100 tweets with the most "likes" from among these 276 tweets* |

| | | | | |
|---|---|---|---|---|
| Quantitative variables | 11 | Explain how quantitative variables were handled in the analyses. If applicable, describe which groupings were chosen and why | 2 | *number of the account's followers, "likes" count, and retweet count at the time of data acquisition.*<br><br>*4-question assessments adapted from assessments of factuality and credibility* |
| Statistical methods | 12 | (*a*) Describe all statistical methods, including those used to control for confounding | 3 | *the number of times a tweet was*<br><br>*reposted on Twitter (ie, retweeted). The Mann-Whitney U test* |
| | | (*b*) Describe any methods used to examine subgroups and interactions | NA | |
| | | (*c*) Explain how missing data were addressed | NA | |
| | | (*d*) *Cohort study*—If applicable, explain how loss to follow-up was addressed<br><br>*Case-control study*—If applicable, explain how matching of cases and controls was addressed<br><br>*Cross-sectional study*—If applicable, describe analytical methods taking account of sampling strategy | NA | |
| | | (*e*) Describe any sensitivity analyses | NA | |

| | | | | |
|---|---|---|---|---|
| **Results** | | | | |
| Participants | 13* | (a) Report numbers of individuals at each stage of study—eg numbers potentially eligible, examined for eligibility, confirmed eligible, included in the study, completing follow-up, and analysed | 3 | *"A total of 69,857 tweets in 100 tweets with the most "likes" from among these 276 tweets"* |
| | | (b) Give reasons for non-participation at each stage | 3 | *"A total of 69,857 tweets in 100 tweets with the most "likes" from among these 276 tweets"* |
| | | (c) Consider use of a flow diagram | 4 | Figure 1 |
| Descriptive data | 14* | (a) Give characteristics of study participants (eg demographic, clinical, social) and information on exposures and potential confounders | 4 | *"Following expert review, 44% of the leading 100 tweets … Other harmful information (1/59, 1.7%; Table 2)."* |
| | | (b) Indicate number of participants with missing data for each variable of interest | NA | |
| | | (c) *Cohort study*—Summarise follow-up time (eg, average and total amount) | NA | |
| Outcome data | 15* | *Cohort study*—Report numbers of outcome events or summary measures over time | *NA* | |
| | | *Case-control study*—Report numbers in each exposure category, or summary measures of exposure | *NA* | |
| | | *Cross-sectional study*—Report numbers of outcome events or summary measures | *5* | *"In the analysis of accuracy and harm combined … Safe information (median 35.0, IQR 0-502 vs 8.0, IQR 0-2197; P=.002; Figure 3D)."* |
| Main results | 16 | (*a*) Give unadjusted estimates and, if applicable, confounder-adjusted estimates and their precision (eg, 95% confidence interval). Make clear which confounders were adjusted for and why they were included | 4 | *44% of the leading 100 tweets contained misinformation (n=44; κ=0.50, 95% CI 0.38-0.65* |
| | | (*b*) Report category boundaries when continuous variables were categorized | NA | |
| | | (*c*) If relevant, consider translating estimates of relative risk into absolute risk for a meaningful time period | NA | |

| | | | | |
|---|---|---|---|---|
| Other analyses | 17 | Report other analyses done—eg analyses of subgroups and interactions, and sensitivity analyses | NA | |

| | | | | |
|---|---|---|---|---|
| Key results | 18 | Summarise key results with reference to study objectives | 6 | *we found that 44% (44/100) of the top 100 tweets in Japanese containing cancer-related* |

| | | | | |
|---|---|---|---|---|
| | | | | *information included misinformation.*<br><br>*We also evaluated the safety of the information disseminated on Twitter. We found that 31% of tweets contained harmful content, and 68.9% of tweets containing misinformation included harmful information* |
| Limitations | 19 | Discuss limitations of the study, taking into account sources of potential bias or imprecision. Discuss both direction and magnitude of any potential bias | 7 | *The main limitation of this study is that …* |
| Interpretation | 20 | Give a cautious overall interpretation of results considering objectives, limitations, multiplicity of analyses, results from similar studies, and other relevant evidence | 7 | *We demonstrated a high prevalence of misinformation and harmful information related to cancer on Twitter in Japan. It is crucial to improve health literacy by raising awareness about the prevalence of cancer-related misinformation.* |
| Generalisability | 21 | Discuss the generalisability (external validity) of the study results | 7 | *the short data collection period (2 months) and limited number of included tweets are also considered limitations. high variability in the number of "likes" and retweets among the leading 100 tweets,* |
| **Other information** | | | | |
| Funding | 22 | Give the source of funding and the role of the funders for the present study and, if applicable, for the original study on which the present article is based | 8 | *Conflicts of Interest*<br><br>*None declared.* |

Table S99. Quality Evaluation for Included Studies Using STROBE

23.STROBE Statement—checklist of items that should be included in reports of observational studies

| | Item No. | Recommendation | Page No. | Relevant text from manuscript |
|---|---|---|---|---|
| **Title and abstract** | 1 | (*a*) Indicate the study's design with a commonly used term in the title or the abstract | NA | |
| | | (*b*) Provide in the abstract an informative and balanced summary of what was done and what was found | 1 | Methods and Results |
| **Introduction** | | | | |
| Background/rationale | 2 | Explain the scientific background and rationale for the investigation being reported | 2 | *Several works have targeted health misinformation [58,59], with most studies using descriptive approaches to study known health misinformation and performing analysis to uncover the common misbeliefs,* |
| Objectives | 3 | State specific objectives, including any prespecified hypotheses | 2 | *we combined a classification model for identifying false HPV vaccine information with unsupervised causality mining to extract the risk perceptions considered to be the attributable causes of HPV antivaccine health concerns based on the content expressed in Twitter messages.* |
| **Methods** | | | | |
| Study design | 4 | Present key elements of study design early in the paper | 2 | Cross-sectional Epidemiology |
| Setting | 5 | Describe the setting, locations, and relevant dates, including periods of recruitment, exposure, follow-up, and data collection | 2 | *corpus related to HPV vaccines with tweets published from December 2013 until December 2017* |
| Participants | 6 | (*a*) *Cohort study*—Give the eligibility criteria, and the sources and methods of selection of participants. Describe methods of follow-up | 3 | *randomly sampled 1000 tweets per year and passed them to 2 annotators in 2 rounds. a list of HPV-related search terms, including, but* |

| | | | | |
|---|---|---|---|---|
| | | *Case-control study*—Give the eligibility criteria, and the sources and methods of case ascertainment and control selection. Give the rationale for the choice of cases and controls<br><br>*Cross-sectional study*—Give the eligibility criteria, and the sources and methods of selection of participants | | *not limited to, "HPV vaccine," "papillomavirus vaccine," "cervical cancer vaccine," "HPV shot," "cervical cancer shot," and "Gardasil." Our modeling pipeline consists of several steps: sampling, annotation and data preprocessing, training, and analysis* |
| | | (*b*) *Cohort study*—For matched studies, give matching criteria and number of exposed and unexposed<br><br>*Case-control study*—For matched studies, give matching criteria and the number of controls per case | NA | |
| Variables | 7 | Clearly define all outcomes, exposures, predictors, potential confounders, and effect modifiers. Give diagnostic criteria, if applicable | 3 | Exposure: tweet text content. Outcomes: classification as true/false misinformation. Risk perceptions (effects) mined via causality analysis. |
| Data sources/ measurement | 8* | For each variable of interest, give sources of data and details of methods of assessment (measurement). Describe comparability of assessment methods if there is more than one group | 2 | *corpus related to HPV vaccines with tweets used the formerly known Crimson Hexagon's (now Brandwatch) social media analytics application programming* |
| Bias | 9 | Describe any efforts to address potential sources of bias | 3 | *Any discrepancies of the ratings from the 2 annotators were reconciled through discussion. For the interrater reliability, a Cohen's kappa coefficient (κ) of 0.75, was considered to indicate good agreement on the task* |
| Study size | 10 | Explain how the study size was arrived at | 3 | *The resulting data set consisted of 5000 labeled and 702,858 unlabeled tweets* |

| | | | | |
|---|---|---|---|---|
| Quantitative variables | 11 | Explain how quantitative variables were handled in the analyses. If applicable, describe which groupings were chosen and why | 2 | we combined a classification model for identifying false HPV vaccine information with |

| | | | | |
|---|---|---|---|---|
| | | | | unsupervised causality mining to extract the risk perceptions |
| Statistical methods | 12 | (*a*) Describe all statistical methods, including those used to control for confounding | 4 | Used convolutional neural networks (CNN), BiLSTM, SVM, and Naive Bayes. Evaluation metrics: F1 score, precision, recall, accuracy. |
| | | (*b*) Describe any methods used to examine subgroups and interactions | NA | |
| | | (*c*) Explain how missing data were addressed | NA | |
| | | (*d*) *Cohort study*—If applicable, explain how loss to follow-up was addressed<br>*Case-control study*—If applicable, explain how matching of cases and controls was addressed<br>*Cross-sectional study*—If applicable, describe analytical methods taking account of sampling strategy | NA | |
| | | (<u>*e*</u>) Describe any sensitivity analyses | NA | |
| **Results** | | | | |
| Participants | 13* | (a) Report numbers of individuals at each stage of study—eg numbers potentially eligible, examined for eligibility, confirmed eligible, included in the study, completing follow-up, and analysed | 2 | Paragraph 2-3 |
| | | (b) Give reasons for non-participation at each stage | NA | |
| | | (c) Consider use of a flow diagram | 3 | |
| Descriptive data | 14* | (a) Give characteristics of study participants (eg demographic, clinical, social) and information on exposures and potential confounders | NA | |
| | | (b) Indicate number of participants with missing data for each variable of interest | NA | |
| | | (c) *Cohort study*—Summarise follow-up time (eg, average and total amount) | NA | |
| Outcome data | 15* | *Cohort study*—Report numbers of outcome events or summary measures over time | *3-4* | *Machine learning result* |
| | | *Case-control study*—Report numbers in each exposure category, or summary measures of exposure | *NA* | |
| | | *Cross-sectional study*—Report numbers of outcome events or summary measures | *NA* | |
| Main results | 16 | (*a*) Give unadjusted estimates and, if applicable, confounder-adjusted estimates and their precision (eg, 95% confidence interval). Make clear which confounders were adjusted for and why they were included | 4 | *Our experimental evaluation showed that CNNs performed better than did the other models* |

| | | | | |
|---|---|---|---|---|
| | | (*b*) Report category boundaries when continuous variables were categorized | | |
| | | (*c*) If relevant, consider translating estimates of relative risk into absolute risk for a meaningful time period | | |
| Other analyses | 17 | Report other analyses done—eg analyses of subgroups and interactions, and sensitivity analyses | 5 | *Causality Mining* |
| Key results | 18 | Summarise key results with reference to study objectives | 6 | *The performance of the CNN and BiLSTM models used in this study showed the feasibility of discerning misinformation from factual information regarding HPV vaccines using the text of tweets* |
| Limitations | 19 | Discuss limitations of the study, taking into account sources of potential bias or imprecision. Discuss both direction and magnitude of any potential bias | 8 | *Limitations*<br>*One common bottleneck…* |
| Interpretation | 20 | Give a cautious overall interpretation of results considering objectives, limitations, multiplicity of analyses, results from similar studies, and other relevant evidence | 8 | *The study has demonstrated a systematic, automatic approach to developing computational models for identifying false HPV vaccine–related information and its associated effects on social media.* |
| Generalisability | 21 | Discuss the generalisability (external validity) of the study results | 8 | *This approach could be generalized to other social media health information and provide insights into estimating the potential effects of a given health topic.* |
| **Other information** | | | | |
| Funding | 22 | Give the source of funding and the role of the funders for the present study and, if applicable, for the original study on which the present article is based | 8 | *This study was supported by a grant from the National Institutes of Health* |

Table S100. Quality Evaluation for Included Studies Using STROBE

24.STROBE Statement—checklist of items that should be included in reports of observational studies

| | Item No. | Recommendation | Page No. | Relevant text from manuscript |
|---|---|---|---|---|
| **Title and abstract** | 1 | (*a*) Indicate the study's design with a commonly used term in the title or the abstract | 1 | Cross-sectional search |
| | | (*b*) Provide in the abstract an informative and balanced summary of what was done and what was found | 1 | We collected Twitter messages containing colorectal … randomly selected tweets (90.7% vs 83.2%; P<0.01). |
| **Introduction** | | | | |
| Background/rationale | 2 | Explain the scientific background and rationale for the investigation being reported | 1 | *the organs related to colorectal cancer often cause myths and misconceptions that are refrained from being discussed in public.12 Twitter, as one of the most vibrant online social media, can be used for an unobtrusive monitoring and discovering of the public awareness of the disease.* |
| Objectives | 3 | State specific objectives, including any prespecified hypotheses | 1 | investigate the information sources and attempt to evaluate the credibility of colorectal cancer information in tweet content. |
| **Methods** | | | | |
| Study design | 4 | Present key elements of study design early in the paper | 1 | Cross-sectional search |
| Setting | 5 | Describe the setting, locations, and relevant dates, including periods of recruitment, exposure, follow-up, and data collection | 1 | We collected tweets containing colorectal cancer keywords for 3 months, from August 1, 2014, to October 31, 2014. |
| Participants | 6 | (*a*) *Cohort study*—Give the eligibility criteria, and the sources and methods of selection of participants. Describe methods of follow-up | 2 | A total of 11 categories were finalized; 7 belonged to the ‘‘medically relevant’’ meta- |

| | | | | |
|---|---|---|---|---|
| | | *Case-control study*—Give the eligibility criteria, and the sources and methods of case ascertainment and control selection. Give the rationale for the choice of cases and controls<br><br>*Cross-sectional study*—Give the eligibility criteria, and the sources and methods of selection of participants | | category and the rest to "medically irrelevant." … |
| | | (*b*) *Cohort study*—For matched studies, give matching criteria and number of exposed and unexposed<br><br>*Case-control study*—For matched studies, give matching criteria and the number of controls per case | NA | |
| Variables | 7 | Clearly define all outcomes, exposures, predictors, potential confounders, and effect modifiers. Give diagnostic criteria, if applicable | 2 | *User Categorization, content categorization, Automatic Classification of Users and Contents* |
| Data sources/ measurement | 8* | For each variable of interest, give sources of data and details of methods of assessment (measurement). Describe comparability of assessment methods if there is more than one group | *2* | *Tweets from Twitter*<br><br>*All members of the committee reviewed each tweet and agreed upon the category to which the tweet belonged* |
| Bias | 9 | Describe any efforts to address potential sources of bias | 2 | *For the undecided tweets, they cross-examined the information against published peer-reviewed journals, preferably of reputation.* |
| Study size | 10 | Explain how the study size was arrived at | 3 | *76,119 tweets authored by 43,365 unique users* |

| | | | | |
|---|---|---|---|---|
| Quantitative variables | 11 | Explain how quantitative variables were handled in the analyses. If applicable, describe which groupings were chosen and why | NA | |
| Statistical methods | 12 | (*a*) Describe all statistical methods, including those used to control for confounding | 3 | A comparison between categorical variables was made using the x2 test or Fisher exact test, as appropriate. |
| | | (*b*) Describe any methods used to examine subgroups and interactions | 4 | Table 2 |

| | | | | |
|---|---|---|---|---|
| | | (*c*) Explain how missing data were addressed | NA | |
| | | (*d*) *Cohort study*—If applicable, explain how loss to follow-up was addressed<br><br>*Case-control study*—If applicable, explain how matching of cases and controls was addressed<br><br>*Cross-sectional study*—If applicable, describe analytical methods taking account of sampling strategy | NA | |
| | | (*e*) Describe any sensitivity analyses | NA | |
| **Results** | | | | |
| Participants | 13* | (a) Report numbers of individuals at each stage of study—eg numbers potentially eligible, examined for eligibility, confirmed eligible, included in the study, completing follow-up, and analysed | 3 | A total of 76,119 tweets authored by 43,365 unique users were collected and analyzed. An average of 827.4 tweets related to colorectal cancer were authored per day (Figure 2). Tweets peaked on certain days when there were important events or news related to the topic, such as the Food and Drug Administration giving approval of an at-home screening test, or the ‘‘Get Your Rear in Gear’’ marathon by the Colon Cancer Coalition. |
| | | (b) Give reasons for non-participation at each stage | NA | |
| | | (c) Consider use of a flow diagram | 3 | |
| Descriptive data | 14* | (a) Give characteristics of study participants (eg demographic, clinical, social) and information on exposures and potential confounders | 3-4 | Table 1 shows a comparison of … *comprised only 0.6% of total*<br><br>*tweets (n.417).* |
| | | (b) Indicate number of participants with missing data for each variable of interest | NA | |
| | | (c) *Cohort study*—Summarise follow-up time (eg, average and total amount) | NA | |
| Outcome data | 15* | *Cohort study*—Report numbers of outcome events or summary measures over time | | |
| | | *Case-control study*—Report numbers in each exposure category, or summary measures of exposure | | |

| | | | | |
|---|---|---|---|---|
| | | *Cross-sectional study*—Report numbers of outcome events or summary measures | 4-5 | Content analysis, *Frequently Shared URLs in Tweets, Information Credibility Analysis … many users, included more medically correct information.* |
| Main results | 16 | (*a*) Give unadjusted estimates and, if applicable, confounder-adjusted estimates and their precision (eg, 95% confidence interval). Make clear which confounders were adjusted for and why they were included | 4 | *Table 2 and Table 3 list both medically relevant and medically irrelevant tweets and their subcategories for individuals and organizations. News articles/research findings were tweeted the most by both individual and organizational users.* |
| | | (*b*) Report category boundaries when continuous variables were categorized | NA | |
| | | (*c*) If relevant, consider translating estimates of relative risk into absolute risk for a meaningful time period | NA | |
| Other analyses | 17 | Report other analyses done—eg analyses of subgroups and interactions, and sensitivity analyses | NA | |
| Key results | 18 | Summarise key results with reference to study objectives | 5 | News articles and/or research findings related to colorectal cancer are most frequently shared; the push of information about colorectal cancer is mainly from users who are ordinary individuals; but organizations tend to tweet information, more so than individuals. The users frequently use shortened URLs in tweets, which lead to external links that are often news and medical resource websites. As for credibility, more medically correct information was found in frequently shared (‘‘retweeted’’) |

| | | | | |
|---|---|---|---|---|
| | | | | tweets than in randomly selected tweets. |
| Limitations | 19 | Discuss limitations of the study, taking into account sources of potential bias or imprecision. Discuss both direction and magnitude of any potential bias | 6 | We acknowledge limitations with our work. … |
| Interpretation | 20 | Give a cautious overall interpretation of results considering objectives, limitations, multiplicity of analyses, results from similar studies, and other relevant evidence | 6 | *Our work suggests Twitter as a promising venue for monitoring public discourse on health.* |
| Generalisability | 21 | Discuss the generalisability (external validity) of the study results | 6 | *Twitter is not without selection bias, although a large-scale data analysis is possible.* |
| **Other information** | | | | |
| Funding | 22 | Give the source of funding and the role of the funders for the present study and, if applicable, for the original study on which the present article is based | NA | |

Table S101. Quality Evaluation for Included Studies Using STROBE

25.STROBE Statement—checklist of items that should be included in reports of observational studies

| | Item No. | Recommendation | Page No. | Relevant text from manuscript |
|---|---|---|---|---|
| **Title and abstract** | 1 | (*a*) Indicate the study's design with a commonly used term in the title or the abstract | 1 | Prospective data collection |
| | | (*b*) Provide in the abstract an informative and balanced summary of what was done and what was found | 1 | Methods and Results |
| **Introduction** | | | | |
| Background/rationale | 2 | Explain the scientific background and rationale for the investigation being reported | 2 | *Users can also pass others' messages on to their followers (called "retweets") as well as make explicit references to others by username (called "mentions"), which puts the tweet into an additional subscription feed.* |
| Objectives | 3 | State specific objectives, including any prespecified hypotheses | 2 | analyze the users and the contents of Korean tweets regarding CRC, and then to estimate the transmissibility of the awareness campaign among Twitter users. |
| **Methods** | | | | |
| Study design | 4 | Present key elements of study design early in the paper | 2 | Prospective data |
| Setting | 5 | Describe the setting, locations, and relevant dates, including periods of recruitment, exposure, follow-up, and data collection | 2 | collected and analyzed tweets related to CRC written in Korean from August 1, 2014, through September 30, 2014<br><br>Utilizing Twitter's public Application Programming Interface (API) and streaming API for real-time updates scripted in Python language, we were able to collect publicly available |

| | | | | |
|---|---|---|---|---|
| | | | | tweets containing colorectal-cancer-related keywords. |
| Participants | 6 | (*a*) *Cohort study*—Give the eligibility criteria, and the sources and methods of selection of participants. Describe methods of follow-up<br>*Case-control study*—Give the eligibility criteria, and the sources and methods of case ascertainment and control selection. Give the rationale for the choice of cases and controls<br>*Cross-sectional study*—Give the eligibility criteria, and the sources and methods of selection of participants | 2 | Each tweet is limited to 140 characters or less. |
| | | (*b*) *Cohort study*—For matched studies, give matching criteria and number of exposed and unexposed<br>*Case-control study*—For matched studies, give matching criteria and the number of controls per case | NA | |
| Variables | 7 | Clearly define all outcomes, exposures, predictors, potential confounders, and effect modifiers. Give diagnostic criteria, if applicable | NA | |
| Data sources/ measurement | 8* | For each variable of interest, give sources of data and details of methods of assessment (measurement). Describe comparability of assessment methods if there is more than one group | *2* | *Tweets from Twitter*<br>*Analysed by a committee of physicians to review the contents of informative tweets* |
| Bias | 9 | Describe any efforts to address potential sources of bias | 2 | *Because a shortened URL may refer to another shortened URL and so on, we expanded the shortened URLs into their original forms by following HTTP redirects.* |
| Study size | 10 | Explain how the study size was arrived at | 3 | 10,387 from 1,452 distinct users, consisting of 9,665 original tweets and 722 retweets. |

| | | | | |
|---|---|---|---|---|
| Quantitative variables | 11 | Explain how quantitative variables were handled in the analyses. If applicable, describe which groupings were chosen and why | NA | |
| Statistical methods | 12 | (*a*) Describe all statistical methods, including those used to control for confounding | 3 | *Categorical variables were compared using the chi-square test or Fisher exact test, as* |

| | | | | |
|---|---|---|---|---|
| | | | | *appropriate. Continuous variables were compared using the Student unpaired t-test.* |
| | | (*b*) Describe any methods used to examine subgroups and interactions | NA | |
| | | (*c*) Explain how missing data were addressed | NA | |
| | | (*d*) *Cohort study*—If applicable, explain how loss to follow-up was addressed<br>*Case-control study*—If applicable, explain how matching of cases and controls was addressed<br>*Cross-sectional study*—If applicable, describe analytical methods taking account of sampling strategy | NA | |
| | | (*e*) Describe any sensitivity analyses | NA | |
| **Results** | | | | |
| Participants | 13* | (a) Report numbers of individuals at each stage of study—eg numbers potentially eligible, examined for eligibility, confirmed eligible, included in the study, completing follow-up, and analysed | 3 | Using the above listed terms, the total number of collected tweet was 10,387 from 1,452 distinct users, consisting of 9,665 original tweets and 722 retweets. The number of tweets from individual users was 6,005 (57.8%), followed by spambots (n = 3,780, 36.4%), and organizational users (n = 602, 5.8%). |
| | | (b) Give reasons for non-participation at each stage | NA | |
| | | (c) Consider use of a flow diagram | NA | |
| Descriptive data | 14* | (a) Give characteristics of study participants (eg demographic, clinical, social) and information on exposures and potential confounders | 3 | In terms of the tweet account analysis (n = 1,452), the number of individual user accounts was 794, among which 786 (98.9%) had user profiles belonging to laypersons, and only 8 (1.1%) had user profiles belonging to medical professionals (Table 1). |
| | | (b) Indicate number of participants with missing data for each variable of interest | NA | |
| | | (c) *Cohort study*—Summarise follow-up time (eg, average and total amount) | NA | |

| | | | | |
|---|---|---|---|---|
| Outcome data | 15* | *Cohort study*—Report numbers of outcome events or summary measures over time | | |
| | | *Case-control study*—Report numbers in each exposure category, or summary measures of exposure | | |
| | | *Cross-sectional study*—Report numbers of outcome events or summary measures | *3-4* | *The total numbers of tweets for each content category were … compared to August (n = 524, 60.7%) (Fig. 3).* |
| Main results | 16 | (*a*) Give unadjusted estimates and, if applicable, confounder-adjusted estimates and their precision (eg, 95% confidence interval). Make clear which confounders were adjusted for and why they were included | 3 | *The total numbers of tweets for each content category were as follows: 8,736 (84.1%) for spam, 1,304 (12.6%) for informative tweets and 347 (3.3%) for communicative tweets. Excluding spam, 1,651 relevant tweets, including informative and communicative tweets, remained.* |
| | | (*b*) Report category boundaries when continuous variables were categorized | NA | |
| | | (*c*) If relevant, consider translating estimates of relative risk into absolute risk for a meaningful time period | NA | |

| | | | | |
|---|---|---|---|---|
| Other analyses | 17 | Report other analyses done—eg analyses of subgroups and interactions, and sensitivity analyses | NA | |

| | | | | |
|---|---|---|---|---|
| Key results | 18 | Summarise key results with reference to study objectives | 5 | the majority of the tweets written in Korean regarding CRC were mostly commercial spam or had inappropriate content. An extremely small percentage consisted of informative public tweets. |
| Limitations | 19 | Discuss limitations of the study, taking into account sources of potential bias or imprecision. Discuss both direction and magnitude of any potential bias | 5 | This study had some limitations. … |
| Interpretation | 20 | Give a cautious overall interpretation of results considering objectives, limitations, multiplicity of analyses, results from similar studies, and other relevant evidence | 5 | *If the reach of credible medical information on CRC is to be extended, public health institutions and organizations* |

| | | | | |
|---|---|---|---|---|
| | | | | *need to pay greater attention to social media.* |
| Generalisability | 21 | Discuss the generalisability (external validity) of the study results | 5 | *The main limitation is the small sample size, which may not be representative of social media for health communication.* |
| **Other information** | | | | |
| Funding | 22 | Give the source of funding and the role of the funders for the present study and, if applicable, for the original study on which the present article is based | NA | |

Table S102. Quality Evaluation for Included Studies Using STROBE

26.STROBE Statement—checklist of items that should be included in reports of observational studies

| | Item No. | Recommendation | Page No. | Relevant text from manuscript |
|---|---|---|---|---|
| **Title and abstract** | 1 | (*a*) Indicate the study's design with a commonly used term in the title or the abstract | 1 | Content analysis |
| | | (*b*) Provide in the abstract an informative and balanced summary of what was done and what was found | 1 | This study combines criminological and computer… transient collectives rather than identifiable groups. |
| **Introduction** | | | | |
| Background/rationale | 2 | Explain the scientific background and rationale for the investigation being reported | 4 | *Online communities active on Twitter have an important role in the proliferation of medical misinformation.* |
| Objectives | 3 | State specific objectives, including any prespecified hypotheses | 2 | *further this path of inquiry by combining criminological and computer science expertise to investigate the existence of structural relationships and to analyze the characteristics of popular actors active in promoting harmful alternative health practices via the online microblogging and social media networking service Twitter, with a specific focus on anticancer treatments in the English-speaking online community.* |
| **Methods** | | | | |
| Study design | 4 | Present key elements of study design early in the paper | 4 | Cross-sectional data collection |
| Setting | 5 | Describe the setting, locations, and relevant dates, including periods of recruitment, exposure, follow-up, and data collection | 4 | Data Collection<br>Tweets were collected for a 5 months period of observation (January 2019–May 2019). |

| | | | | |
|---|---|---|---|---|
| Participants | 6 | (*a*) *Cohort study*—Give the eligibility criteria, and the sources and methods of selection of participants. Describe methods of follow-up<br><br>*Case-control study*—Give the eligibility criteria, and the sources and methods of case ascertainment and control selection. Give the rationale for the choice of cases and controls<br><br>*Cross-sectional study*—Give the eligibility criteria, and the sources and methods of selection of participants | 4 | After the initial data set was created, the criminologist provided incremental feedback to the computer scientist suggesting words and/or users to be used for filtering as manifestly irrelevant for the scope of this work (e.g., "cancer" can refer to horoscope-related jargon, or combination of keywords such as "cancer + alternative" can lead to tweets posted by reputable publishing houses promoting new science-based academic articles on cancer treatments). |
| | | (*b*) *Cohort study*—For matched studies, give matching criteria and number of exposed and unexposed<br><br>*Case-control study*—For matched studies, give matching criteria and the number of controls per case | NA | |
| Variables | 7 | Clearly define all outcomes, exposures, predictors, potential confounders, and effect modifiers. Give diagnostic criteria, if applicable | 4 | *number of retweets, likes, and responses, 85 authors were above this average, and also their tweets were considered for qualitative analysis.*<br><br>*Relevant author; to be dismissed), type of treatment, motivation, and attitude* |
| Data sources/ measurement | 8* | For each variable of interest, give sources of data and details of methods of assessment (measurement). Describe comparability of assessment methods if there is more than one group | *4* | *Tweets from Twitter*<br><br>*Study has been carried out by a criminologist and a computer scientist.* |
| Bias | 9 | Describe any efforts to address potential sources of bias | 5 | *collected data by using a software tool compatible with Twitter policies, and we collected information that* |

| | | | | |
|---|---|---|---|---|
| | | | | *had already been posted on the Twitter platform (hence, the participants had already accepted Twitter's Terms and Conditions). For concerns related to Twitter users' anonymity, we did not use personal identifiers and are not quoting directly the content of the tweets* |
| Study size | 10 | Explain how the study size was arrived at | 4 | 7,676 relevant tweets from 5,615 users |
| Quantitative variables | 11 | Explain how quantitative variables were handled in the analyses. If applicable, describe which groupings were chosen and why | 4 | *number of retweets, likes, and responses*<br><br>*85 authors were above this average, and also their tweets were considered for qualitative analysis.* |
| Statistical methods | 12 | (*a*) Describe all statistical methods, including those used to control for confounding | 4 | *Network Data Analysis*<br><br>*One part of the analysis was dedicated to investigating the relationships among the promoters of alternative anticancer treatments—whether they grouped together (how interlinked was the network), how they grouped together (how did those links structure the network), and why they grouped together (what factors encouraged them to group).* |
| | | (*b*) Describe any methods used to examine subgroups and interactions | NA | |
| | | (*c*) Explain how missing data were addressed | NA | |
| | | (*d*) *Cohort study*—If applicable, explain how loss to follow-up was addressed<br><br>*Case-control study*—If applicable, explain how matching of cases and controls was addressed | NA | |

| | | | | |
|---|---|---|---|---|
| | | *Cross-sectional study*—If applicable, describe analytical methods taking account of sampling strategy | | |
| | | (e) Describe any sensitivity analyses | NA | |
| **Results** | | | | |
| Participants | 13* | (a) Report numbers of individuals at each stage of study—eg numbers potentially eligible, examined for eligibility, confirmed eligible, included in the study, completing follow-up, and analysed | 5 | As is indicated by the ratio of relevant tweets (7,676) to relevant users (5,615), the overall network is sparse with the vast majority of users (5,181) responsible for only a single tweet. In addition, most tweets (4,680) are isolated, connecting to no other users via mention or reply-to relationships. |
| | | (b) Give reasons for non-participation at each stage | NA | |
| | | (c) Consider use of a flow diagram | NA | |
| Descriptive data | 14* | (a) Give characteristics of study participants (eg demographic, clinical, social) and information on exposures and potential confounders | 6 | the features of the social network observed suggest that, contrary to previous research describing supporters of non-science-based medical treatments online (including in the Twittersphere, in the case of anti-vaxxers) as part of a community or a movement, most users tend to cluster in transient and loosely formed collectives around a common interest or activity |
| | | (b) Indicate number of participants with missing data for each variable of interest | NA | |
| | | (c) *Cohort study*—Summarise follow-up time (eg, average and total amount) | NA | |
| Outcome data | 15* | *Cohort study*—Report numbers of outcome events or summary measures over time | | |
| | | *Case-control study*—Report numbers in each exposure category, or summary measures of exposure | | |
| | | *Cross-sectional study*—Report numbers of outcome events or summary measures | *7-8* | *Content analysis* |

| | | | | |
|---|---|---|---|---|
| Main results | 16 | (*a*) Give unadjusted estimates and, if applicable, confounder-adjusted estimates and their precision (eg, 95% confidence interval). Make clear which confounders were adjusted for and why they were included | 8 | *Twitter users active in sharing cancer-related research-based information might form relatively more active and more stable networks on Twitter, and hence their presence is higher among the "more popular" tweets or among the authors with more network capital; providers of non-science-based anticancer treatments and those sympathizing with them tend on the contrary to be very disperse in the Twittersphere* |
| | | (*b*) Report category boundaries when continuous variables were categorized | NA | |
| | | (*c*) If relevant, consider translating estimates of relative risk into absolute risk for a meaningful time period | NA | |
| Other analyses | 17 | Report other analyses done—eg analyses of subgroups and interactions, and sensitivity analyses | NA | |
| Key results | 18 | Summarise key results with reference to study objectives | 10 | Social media analytics have allowed us to explore the existence of structural relationships among actors involved in relevant discourses in the Twittersphere, and have shown the lack of a proper community of interest, and rather the existence of transient collectives clustering around specific and popular discussions, themes, or actors in the social network. |
| Limitations | 19 | Discuss limitations of the study, taking into account sources of potential bias or imprecision. Discuss both direction and magnitude of any potential bias | 10 | *study focused only on anticancer treatments* |
| Interpretation | 20 | Give a cautious overall interpretation of results considering objectives, limitations, multiplicity of analyses, results from similar studies, and other relevant evidence | 10 | *a better understanding of the unique characteristics of specific online social networks and of the features and motivations of* |

| | | | | |
|---|---|---|---|---|
| | | | | *their most successful actors can have a fundamental role to better focus and tailor awareness-raising efforts* |
| Generalisability | 21 | Discuss the generalisability (external validity) of the study results | 10 | *study focused only on anticancer treatments, which traditionally have been (and still are) a particularly fertile ground for harmful non-science-based anticancer treatments because of the public panic toward cancer and the lack of a general, simple, or painless cure* |
| **Other information** | | | | |
| Funding | 22 | Give the source of funding and the role of the funders for the present study and, if applicable, for the original study on which the present article is based | 11 | *The author(s) disclosed receipt of the following financial support for the research, authorship* |

Table S103. Quality Evaluation for Included Studies Using STROBE

27.STROBE Statement—checklist of items that should be included in reports of observational studies

| | Item No. | Recommendation | Page No. | Relevant text from manuscript |
|---|---|---|---|---|
| **Title and abstract** | 1 | (*a*) Indicate the study's design with a commonly used term in the title or the abstract | 1 | Cross-sectional search |
| | | (*b*) Provide in the abstract an informative and balanced summary of what was done and what was found | 1 | *Materials & methods*<br>Results |
| **Introduction** | | | | |
| Background/rationale | 2 | Explain the scientific background and rationale for the investigation being reported | 1 | *Viral news and advertisements target cancer patients with inaccurate claims about alternative cancer treatments and may mislead some patients into forgoing conventional therapies [6], potentially resulting in avoidable deaths in patients with curable cancers* |
| Objectives | 3 | State specific objectives, including any prespecified hypotheses | 1 | characterize the growing online interest in using cannabis as a cancer cure, the relationship between cannabis legalization and online interest, and the role of physicians and leading cancer organizations in clarifying misinformation. |
| **Methods** | | | | |
| Study design | 4 | Present key elements of study design early in the paper | 2 | Cross-sectional search |
| Setting | 5 | Describe the setting, locations, and relevant dates, including periods of recruitment, exposure, follow-up, and data collection | 2 | Using Google Trends, we characterized the global internet interest in cannabis and cancer from January 2011 through July 2018, |
| Participants | 6 | (*a*) *Cohort study*—Give the eligibility criteria, and the sources and methods of selection of participants. Describe methods of follow-up | 2 | Google Trends reports a time-series of RSV for each search term [10]. A search |

| | | | | |
|---|---|---|---|---|
| | | *Case-control study*—Give the eligibility criteria, and the sources and methods of case ascertainment and control selection. Give the rationale for the choice of cases and controls<br>*Cross-sectional study*—Give the eligibility criteria, and the sources and methods of selection of participants | | term's monthly RSV is the normalized fraction of all Google searches containing that search term; the normalization constant is chosen such that the maximum RSV achieved across all searched terms is 100. |
| | | (*b*) *Cohort study*—For matched studies, give matching criteria and number of exposed and unexposed<br>*Case-control study*—For matched studies, give matching criteria and the number of controls per case | NA | |
| Variables | 7 | Clearly define all outcomes, exposures, predictors, potential confounders, and effect modifiers. Give diagnostic criteria, if applicable | 2 | *relative search volume (RSV)* |
| Data sources/ measurement | 8* | For each variable of interest, give sources of data and details of methods of assessment (measurement). Describe comparability of assessment methods if there is more than one group | *2* | *Data were from Google Trends* |
| Bias | 9 | Describe any efforts to address potential sources of bias | 2 | *We also compared the mean rate of change in RSV in states that legalized recreational cannabis after 2011 (no state legalized recreational cannabis before 2011), and states that never legalized recreational cannabis.* |
| Study size | 10 | Explain how the study size was arrived at | 2 | 32 states' RSV |

| | | | | |
|---|---|---|---|---|
| Quantitative variables | 11 | Explain how quantitative variables were handled in the analyses. If applicable, describe which groupings were chosen and why | 2 | *RSV trend* |
| Statistical methods | 12 | (*a*) Describe all statistical methods, including those used to control for confounding | 2 | *We compared trends over time for the RSV of ''cannabis cancer' versus the RSV of 'standard cancer therapy' using least squares linear regression (RSV = β0 + β1 * time (month)).* |

| | | | | |
|---|---|---|---|---|
| | | | | *Analysis of covariance (ANCOVA) was used to test between group differences based on their legalization group classification.* |
| | | (*b*) Describe any methods used to examine subgroups and interactions | NA | |
| | | (*c*) Explain how missing data were addressed | NA | |
| | | (*d*) *Cohort study*—If applicable, explain how loss to follow-up was addressed<br>*Case-control study*—If applicable, explain how matching of cases and controls was addressed<br>*Cross-sectional study*—If applicable, describe analytical methods taking account of sampling strategy | NA | |
| | | (*e*) Describe any sensitivity analyses | NA | |
| **Results** | | | | |
| Participants | 13* | (a) Report numbers of individuals at each stage of study—eg numbers potentially eligible, examined for eligibility, confirmed eligible, included in the study, completing follow-up, and analysed | 3 | From January 2011 to July 2018, the RSV of 'cannabis cancer' queries increased from 10.3 to 19.3, while the RSV for 'standard cancer therapy' queries changed little, from 87.6 to 88.5 (Figure 1). During the study period, the RSV of 'cannabis cancer' queries increased at a rate 10 times faster than the RSV of 'standard cancer therapies' queries (0.10/month versus 0.01/month, p<0.001). |
| | | (b) Give reasons for non-participation at each stage | NA | |
| | | (c) Consider use of a flow diagram | NA | |
| Descriptive data | 14* | (a) Give characteristics of study participants (eg demographic, clinical, social) and information on exposures and potential confounders | 3 | *"During the study period, the RSV of … recreational cannabis (0.15/month, Figure 2B, p=0.004)."* |
| | | (b) Indicate number of participants with missing data for each variable of interest | NA | |

| | | | | |
|---|---|---|---|---|
| | | (c) *Cohort study*—Summarise follow-up time (eg, average and total amount) | NA | |
| Outcome data | 15* | *Cohort study*—Report numbers of outcome events or summary measures over time | | |
| | | *Case-control study*—Report numbers in each exposure category, or summary measures of exposure | | |
| | | *Cross-sectional study*—Report numbers of outcome events or summary measures | *4-5* | *"The first social media analysis, … legalized had no Facebook posts or tweets on cannabis during this period."* |
| Main results | 16 | (*a*) Give unadjusted estimates and, if applicable, confounder-adjusted estimates and their precision (eg, 95% confidence interval). Make clear which confounders were adjusted for and why they were included | 3 | *States that had legalized medical cannabis before 2011 had the highest RSV growth (0.22/month) over the study period, followed by states that had legalized medical cannabis after 2011 (0.18/month); those states that had never legalized medical cannabis had the lowest RSV growth (0,12/month, Figure 2A, p = 0.04).* |
| | | (*b*) Report category boundaries when continuous variables were categorized | NA | |
| | | (*c*) If relevant, consider translating estimates of relative risk into absolute risk for a meaningful time period | NA | |

| | | | | |
|---|---|---|---|---|
| Other analyses | 17 | Report other analyses done—eg analyses of subgroups and interactions, and sensitivity analyses | 3 | Similarly, states that legalized recreational cannabis showed an RSV growth of 0.25 per month, significantly greater than states that never legalized recreational cannabis (0.15/month, Figure 2B, p=0.004). |

| | | | | |
|---|---|---|---|---|
| Key results | 18 | Summarise key results with reference to study objectives | 5 | In this study, we found a rising online interest in using cannabis for cancer, more so in states that had legalized medical or recreational cannabis. We also found that the most popular |

| | | | | |
|---|---|---|---|---|
| | | | | news stories shared on social media on this topic misleadingly described cannabis as a cancer cure. |
| Limitations | 19 | Discuss limitations of the study, taking into account sources of potential bias or imprecision. Discuss both direction and magnitude of any potential bias | 6 | This study has several limitations |
| Interpretation | 20 | Give a cautious overall interpretation of results considering objectives, limitations, multiplicity of analyses, results from similar studies, and other relevant evidence | 6 | *a crucial opportunity for the oncology community to correct this misinformation and communicate accurate information to patient and caregiver communities.* |
| Generalisability | 21 | Discuss the generalisability (external validity) of the study results | 6 | *Further, not all states had RSV data available due to periods with low search volume, limiting generalizability.* |
| **Other information** | | | | |
| Funding | 22 | Give the source of funding and the role of the funders for the present study and, if applicable, for the original study on which the present article is based | 6 | *All authors have declared that they have no financial relationships at present or within the previous three years with any organizations that might have an interest in the submitted work.* |

Table S104. Quality Evaluation for Included Studies Using STROBE

28.STROBE Statement—checklist of items that should be included in reports of observational studies

| | Item No. | Recommendation | Page No. | Relevant text from manuscript |
|---|---|---|---|---|
| **Title and abstract** | 1 | (*a*) Indicate the study's design with a commonly used term in the title or the abstract | 1 | Quality evaluation |
| | | (*b*) Provide in the abstract an informative and balanced summary of what was done and what was found | 1 | Methods and Results |
| **Introduction** | | | | |
| Background/rationale | 2 | Explain the scientific background and rationale for the investigation being reported | 2 | *WPA has become an indispensable information dissemination platform for Chinese government agencies, medical institutions, enterprises and individuals [13]. A large amount of health information generated on WPAs has immense potential to affect the public's health.* |
| Objectives | 3 | State specific objectives, including any prespecified hypotheses | 2 | Evaluate their overall quality, find their merits and demerits, and accordingly, provide references for improving the quality of health information on BC treatment. |
| **Methods** | | | | |
| Study design | 4 | Present key elements of study design early in the paper | 2 | Cross-sectional search |
| Setting | 5 | Describe the setting, locations, and relevant dates, including periods of recruitment, exposure, follow-up, and data collection | 2 | In this study, WPAs related to BC were collected on April 11, 2021. We included the WPAs those WeChat profile page clearly indicated that they provided health information for patients with BC. |

| | | | | |
|---|---|---|---|---|
| Participants | 6 | (*a*) *Cohort study*—Give the eligibility criteria, and the sources and methods of selection of participants. Describe methods of follow-up<br><br>*Case-control study*—Give the eligibility criteria, and the sources and methods of case ascertainment and control selection. Give the rationale for the choice of cases and controls<br><br>*Cross-sectional study*—Give the eligibility criteria, and the sources and methods of selection of participants | 2 | The exclusion criteria were as follows: the WPAs (1) were duplicate; (2) had not updated for more than one year; (3) delivered only academic research information to professionals; (4) whose target population were not only patients with BC. |
| | | (*b*) *Cohort study*—For matched studies, give matching criteria and number of exposed and unexposed<br><br>*Case-control study*—For matched studies, give matching criteria and the number of controls per case | NA | |
| Variables | 7 | Clearly define all outcomes, exposures, predictors, potential confounders, and effect modifiers. Give diagnostic criteria, if applicable | 3 | *WPA names, account subjects, article titles, and treatment options.* |
| Data sources/ measurement | 8* | For each variable of interest, give sources of data and details of methods of assessment (measurement). Describe comparability of assessment methods if there is more than one group | *2-3* | *Extract data from WPA*<br><br>*Two researchers collected the information from every selected article and reached agreements through discussion.* |
| Bias | 9 | Describe any efforts to address potential sources of bias | 3 | *To avoid bias, up to four newly published articles were included from WPAs as required below: articles on health information for BC treatment, and excluding (1) duplicate articles; (2) publications with only pictures, videos, or links; (3) news reports or notices; (4) academic articles.*<br><br>*If there was an inconsistency in the scores of an item, the two raters would reach a consensus; otherwise, the research group reached a* |

| | | | | |
|---|---|---|---|---|
| | | | | *final decision through discussion.* |
| Study size | 10 | Explain how the study size was arrived at | 3 | 37 WPAs, 136 articles |
| Quantitative variables | 11 | Explain how quantitative variables were handled in the analyses. If applicable, describe which groupings were chosen and why | 3-4 | *DISCERN, 5-point Likert scale* |
| Statistical methods | 12 | (*a*) Describe all statistical methods, including those used to control for confounding | 4 | *The quality scores of 136 articles were described using median and interquartile range (IQR). The statistics were described using frequency, constituent ratio, and rate. Rank-sum test was used to compare the scores of article qualities and DISCERN items between the diverse categories of account subjects and different treatment options.* |
| | | (*b*) Describe any methods used to examine subgroups and interactions | NA | |
| | | (*c*) Explain how missing data were addressed | NA | |
| | | (*d*) *Cohort study*—If applicable, explain how loss to follow-up was addressed<br>*Case-control study*—If applicable, explain how matching of cases and controls was addressed<br>*Cross-sectional study*—If applicable, describe analytical methods taking account of sampling strategy | NA | |
| | | (*e*) Describe any sensitivity analyses | NA | |
| **Results** | | | | |
| Participants | 13* | (a) Report numbers of individuals at each stage of study—eg numbers potentially eligible, examined for eligibility, confirmed eligible, included in the study, completing follow-up, and analysed | 4 | Characteristics of included WPAs and articles In the aspect of account subject, the 37 WPAs were classified into four categories: individual users, accounting for the largest proportion (20, 54%), of which 14 were operated by clinicians; enterprise (8, 22%); institution |

| | | | | |
|---|---|---|---|---|
| | | | | (6, 16%); non-profit organization (3, 8%). |
| | | (b) Give reasons for non-participation at each stage | | |
| | | (c) Consider use of a flow diagram | 3 | Figure 1 |
| Descriptive data | 14* | (a) Give characteristics of study participants (eg demographic, clinical, social) and information on exposures and potential confounders | 4 | The number of individual users' publications also occupied … mostly surgical and medical treatments. |
| | | (b) Indicate number of participants with missing data for each variable of interest | | |
| | | (c) *Cohort study*—Summarise follow-up time (eg, average and total amount) | | |
| Outcome data | 15* | *Cohort study*—Report numbers of outcome events or summary measures over time | | |
| | | *Case-control study*—Report numbers in each exposure category, or summary measures of exposure | | |
| | | *Cross-sectional study*—Report numbers of outcome events or summary measures | *4-5* | *The total DISCERN scores of 136 articles range from 21 … were at the levels of "fair" and below.* |
| Main results | 16 | (*a*) Give unadjusted estimates and, if applicable, confounder-adjusted estimates and their precision (eg, 95% confidence interval). Make clear which confounders were adjusted for and why they were included | 4 | *the quality of BC treatment health information was "fair", of which only 28 (21%) were of "good" or higher quality.* |
| | | (*b*) Report category boundaries when continuous variables were categorized | NA | |
| | | (*c*) If relevant, consider translating estimates of relative risk into absolute risk for a meaningful time period | NA | |

| | | | | |
|---|---|---|---|---|
| Other analyses | 17 | Report other analyses done—eg analyses of subgroups and interactions, and sensitivity analyses | 6 | Statistical significant differences were not found in the DISCERN-part scores either among account subject categories or among treatment options |

| | | | | |
|---|---|---|---|---|
| Key results | 18 | Summarise key results with reference to study objectives | 7 | The quality of most BC treatment health information was at a medium level. |

| | | | | |
|---|---|---|---|---|
| | | | | this study found that WPAs lacked information reliability and sufficient details on treatment integrity. |
| Limitations | 19 | Discuss limitations of the study, taking into account sources of potential bias or imprecision. Discuss both direction and magnitude of any potential bias | 8 | Restricted by the characteristics and functions of the WeChat platform… |
| Interpretation | 20 | Give a cautious overall interpretation of results considering objectives, limitations, multiplicity of analyses, results from similar studies, and other relevant evidence | 8 | *To improve the quality of the articles, WPA producers should specify the sources of evidence for essential information, provide additional information about treatment options* |
| Generalisability | 21 | Discuss the generalisability (external validity) of the study results | 8 | *Restricted by the characteristics and functions of the WeChat platform, the search for WPAs pushing BC health information was not exhaustive.* |
| **Other information** | | | | |
| Funding | 22 | Give the source of funding and the role of the funders for the present study and, if applicable, for the original study on which the present article is based | 8 | This work was supported by the Anhui Medical University School of Nursing Graduate Youth Program Cultivation Project |

Table S105. Quality Evaluation for Included Studies Using STROBE

29.STROBE Statement—checklist of items that should be included in reports of observational studies

| | Item No. | Recommendation | Page No. | Relevant text from manuscript |
|---|---|---|---|---|
| **Title and abstract** | 1 | (*a*) Indicate the study's design with a commonly used term in the title or the abstract | 1 | Cross-sectional search |
| | | (*b*) Provide in the abstract an informative and balanced summary of what was done and what was found | 1 | Methods and Results |
| **Introduction** | | | | |
| Background/rationale | 2 | Explain the scientific background and rationale for the investigation being reported | 2 | *In the last decade, social media has exacerbated individuals' uncertainty about cancer. Unlike traditional media, most health-related content on social media is generated and shared by patients and caregivers based on their own personal experiences.* |
| Objectives | 3 | State specific objectives, including any prespecified hypotheses | 2 | *consider the types of misinformation and their diffusion characteristics [26]. To fill in the gaps in the literature, this study focuses on 2 gynecologic cancers, namely breast and cervical cancers,* |
| **Methods** | | | | |
| Study design | 4 | Present key elements of study design early in the paper | 2 | Cross-sectional search |
| Setting | 5 | Describe the setting, locations, and relevant dates, including periods of recruitment, exposure, follow-up, and data collection | 2 | Two keywords "乳癌/乳腺癌" [breast cancer] and "子宫癌/宫颈癌" [cervical cancer] were employed to search tweets about<br><br>breast cancer and cervical cancer on Weibo, one of the most popular social media platforms in China. |

| | | | | |
|---|---|---|---|---|
| Participants | 6 | (*a*) *Cohort study*—Give the eligibility criteria, and the sources and methods of selection of participants. Describe methods of follow-up<br><br>*Case-control study*—Give the eligibility criteria, and the sources and methods of case ascertainment and control selection. Give the rationale for the choice of cases and controls<br><br>*Cross-sectional study*—Give the eligibility criteria, and the sources and methods of selection of participants | 2 | Of the 2691 total tweets on Weibo, 1144 tweets (1144/2691, 42.51%) only expressed personal emotions and experiences that cannot be identified as truth or falsehood. These tweets were excluded from further analysis. |
| | | (*b*) *Cohort study*—For matched studies, give matching criteria and number of exposed and unexposed<br><br>*Case-control study*—For matched studies, give matching criteria and the number of controls per case | NA | |
| Variables | 7 | Clearly define all outcomes, exposures, predictors, potential confounders, and effect modifiers. Give diagnostic criteria, if applicable | 2-3 | *The content, post time, and diffusion path of each tweet were retrieved.*<br><br>*the scale of retweets, the range of retweets, the structural virality of retweets, the number of comments, and the number of likes.* |
| Data sources/ measurement | 8* | For each variable of interest, give sources of data and details of methods of assessment (measurement). Describe comparability of assessment methods if there is more than one group | *2* | *Tweets from Weibo*<br><br>*A total of 2 medical school graduate students with expertise in gynecologic diseases were recruited to complete the coding.* |
| Bias | 9 | Describe any efforts to address potential sources of bias | 2 | *To minimize the potential harm to Weibo users, all the data collected from Weibo were deindividualized to maintain the users' anonymity.*<br><br>*Krippendorff alpha [30] for this round coding was .95, which means that the intercoder reliability for thematic category is well accepted.* |

| | | | | |
|---|---|---|---|---|
| Study size | 10 | Explain how the study size was arrived at | 2 | 1547 tweets |
| Quantitative variables | 11 | Explain how quantitative variables were handled in the analyses. If applicable, describe which groupings were chosen and why | 3 | *the scale of retweets, the range of retweets, the structural virality of retweets, the number of comments, and the number of likes.* |
| Statistical methods | 12 | (*a*) Describe all statistical methods, including those used to control for confounding | 3 | *Descriptive statistics and MANOVA* |
| | | (*b*) Describe any methods used to examine subgroups and interactions | NA | |
| | | (*c*) Explain how missing data were addressed | NA | |
| | | (*d*) *Cohort study*—If applicable, explain how loss to follow-up was addressed<br>*Case-control study*—If applicable, explain how matching of cases and controls was addressed<br>*Cross-sectional study*—If applicable, describe analytical methods taking account of sampling strategy | NA | |
| | | (*e*) Describe any sensitivity analyses | NA | |
| **Results** | | | | |
| Participants | 13* | (a) Report numbers of individuals at each stage of study—eg numbers potentially eligible, examined for eligibility, confirmed eligible, included in the study, completing follow-up, and analysed | 3 | Among the 1547 medically oriented tweets, the most commonly exchanged type of cancer-related information was background knowledge (749/1547, 48.42%), followed by prevention (467/1547, 30.19%), treatment (189/1547, 12.21%), and diagnosis (142/1547, 9.18%). |
| | | (b) Give reasons for non-participation at each stage | NA | |
| | | (c) Consider use of a flow diagram | NA | |
| Descriptive data | 14* | (a) Give characteristics of study participants (eg demographic, clinical, social) and information on exposures and potential confounders | 3 | Moreover, 66.13% (1023/1547) of the tweets provided … *radiation therapy, drug therapy, and other therapies.* |

| | | | | |
|---|---|---|---|---|
| | | (b) Indicate number of participants with missing data for each variable of interest | NA | |
| | | (c) *Cohort study*—Summarise follow-up time (eg, average and total amount) | NA | |
| Outcome data | 15* | *Cohort study*—Report numbers of outcome events or summary measures over time | | |
| | | *Case-control study*—Report numbers in each exposure category, or summary measures of exposure | | |
| | | *Cross-sectional study*—Report numbers of outcome events or summary measures | *5-6* | *Diffusion Characteristics of Gynecologic Cancer–Related Information …* |
| Main results | 16 | (*a*) Give unadjusted estimates and, if applicable, confounder-adjusted estimates and their precision (eg, 95% confidence interval). Make clear which confounders were adjusted for and why they were included | 5 | *The popularity of misinformation in terms of its diffusion was unevenly distributed, with several tweets receiving a large number of retweets, whereas the majority received no retweets or likes.* |
| | | (*b*) Report category boundaries when continuous variables were categorized | NA | |
| | | (*c*) If relevant, consider translating estimates of relative risk into absolute risk for a meaningful time period | NA | |

| | | | | |
|---|---|---|---|---|
| Other analyses | 17 | Report other analyses done—eg analyses of subgroups and interactions, and sensitivity analyses | 6 | A between-subject multivariate analysis of variance (MANOVA) was performed to test the differences on the 5 diffusion characteristics between information in different thematic categories and those with different information veracity. |

| | | | | |
|---|---|---|---|---|
| Key results | 18 | Summarise key results with reference to study objectives | 8 | of the 2691 total tweets examined, more than half included medically oriented information about cancer. Although most of the medically oriented tweets provided accurate information, more than 30% contained misinformation. |

| | | | | |
|---|---|---|---|---|
| Limitations | 19 | Discuss limitations of the study, taking into account sources of potential bias or imprecision. Discuss both direction and magnitude of any potential bias | 8 | Several limitations of this study should be acknowledged. … |
| Interpretation | 20 | Give a cautious overall interpretation of results considering objectives, limitations, multiplicity of analyses, results from similar studies, and other relevant evidence | 8 | *Medical professionals should make efforts to correct misinformation regarding the appropriate ways of preventing gynecologic cancers and decrease the spread of cancer-preventing misinformation on social media.* |
| Generalisability | 21 | Discuss the generalisability (external validity) of the study results | 8 | *The data were extracted in July 2016, which were 2 years old. With the increasing popularity of social media, people's health literacy has been continuously improving over the last few years.* |
| **Other information** | | | | |
| Funding | 22 | Give the source of funding and the role of the funders for the present study and, if applicable, for the original study on which the present article is based | NA | |

Table S106. Quality Evaluation for Included Studies Using STROBE

30.STROBE Statement—checklist of items that should be included in reports of observational studies

| | Item No. | Recommendation | Page No. | Relevant text from manuscript |
|---|---|---|---|---|
| **Title and abstract** | 1 | (*a*) Indicate the study's design with a commonly used term in the title or the abstract | 1 | Content analysis |
| | | (*b*) Provide in the abstract an informative and balanced summary of what was done and what was found | 1 | Methods and Results |
| **Introduction** | | | | |
| Background/rationale | 2 | Explain the scientific background and rationale for the investigation being reported | 2 | *Xigua Video places emphasis on user-uploaded videos and has accumulated an average of 120 million daily views [6]. Although information from the website is easily accessible, users may be unable to judge the quality and accuracy of its contents* |
| Objectives | 3 | State specific objectives, including any prespecified hypotheses | 2 | To better understand how Chinese internet users discuss breast cancer on social media platforms such as Xigua Video |
| **Methods** | | | | |
| Study design | 4 | Present key elements of study design early in the paper | 2 | Cross-sectional search |
| Setting | 5 | Describe the setting, locations, and relevant dates, including periods of recruitment, exposure, follow-up, and data collection | 2 | On February 4, 2020, a Xigua Video search was performed using the keyword "breast cancer" for videos. |
| Participants | 6 | (*a*) *Cohort study*—Give the eligibility criteria, and the sources and methods of selection of participants. Describe methods of follow-up<br>*Case-control study*—Give the eligibility criteria, and the sources and methods of case ascertainment and control selection. Give the rationale for the choice of cases and controls<br>*Cross-sectional study*—Give the eligibility criteria, and the sources and methods of selection of participants | 2 | In the case of duplicate videos, only 1 was taken into consideration. Videos were excluded if they were irrelevant or lacked accompanying audio. |

| | | | | |
|---|---|---|---|---|
| | | (*b*) *Cohort study*—For matched studies, give matching criteria and number of exposed and unexposed<br><br>*Case-control study*—For matched studies, give matching criteria and the number of controls per case | NA | |
| Variables | 7 | Clearly define all outcomes, exposures, predictors, potential confounders, and effect modifiers. Give diagnostic criteria, if applicable | 2 | *content, duration in months on Xigua Video, number of views, length, and comments* |
| Data sources/ measurement | 8* | For each variable of interest, give sources of data and details of methods of assessment (measurement). Describe comparability of assessment methods if there is more than one group | *2* | *Videos were from Xigua Video, 2 physicians independently evaluated the overall quality of the videos* |
| Bias | 9 | Describe any efforts to address potential sources of bias | 2 | *The kappa statistic for the 2 reviewers was κ=0.78, which indicated substantial agreement* |
| Study size | 10 | Explain how the study size was arrived at | 2 | 170 videos |

| | | | | |
|---|---|---|---|---|
| Quantitative variables | 11 | Explain how quantitative variables were handled in the analyses. If applicable, describe which groupings were chosen and why | 2 | *DISCERN tool, 5-point global quality score, number of views, length* |
| Statistical methods | 12 | (*a*) Describe all statistical methods, including those used to control for confounding | 2 | *Numerical variables were reported as mean (SD) or median (IQR) values. The Student t test, the Mann-Whitney U test, analysis of variance, and the Kruskal-Wallis test were applied for the comparison of numerical variables. The Dwass-Steel-Critchlow-Fligner post hoc test was conducted after the Kruskal-Wallis test. Categorical variables were stated as number (n) and percentage (%). In the comparison of categorical variables, chi-square and Fisher exact tests were used.* |
| | | (*b*) Describe any methods used to examine subgroups and interactions | NA | |

| | | | | |
|---|---|---|---|---|
| | | (*c*) Explain how missing data were addressed | NA | |
| | | (*d*) *Cohort study*—If applicable, explain how loss to follow-up was addressed<br><br>*Case-control study*—If applicable, explain how matching of cases and controls was addressed<br><br>*Cross-sectional study*—If applicable, describe analytical methods taking account of sampling strategy | NA | |
| | | (*e*) Describe any sensitivity analyses | NA | |
| **Results** | | | | |
| Participants | 13* | (a) Report numbers of individuals at each stage of study—eg numbers potentially eligible, examined for eligibility, confirmed eligible, included in the study, completing follow-up, and analysed | 2 | The videos were analyzed based on the topics covered therein.<br><br>In all categories, the topic of treatments was the most frequently covered (119/170, 70%), followed by symptoms (56/170, 33%), prognosis (44/170, 26%), anatomy (34/170, 20%), prevention (25/170, 15%), and etiology (18/170, 11%). |
| | | (b) Give reasons for non-participation at each stage | NA | |
| | | (c) Consider use of a flow diagram | NA | |
| Descriptive data | 14* | (a) Give characteristics of study participants (eg demographic, clinical, social) and information on exposures and potential confounders | 2 | A total of 41.8% (71/170) of the videos were posted on the website by individuals. Medical advertisements and health information websites were responsible for uploading 25.9% (44/170) and 12.9% (22/170) of the total videos, respectively. The videos contributed by health care professionals accounted for only 19.4% (33/170). |
| | | (b) Indicate number of participants with missing data for each variable of interest | NA | |
| | | (c) *Cohort study*—Summarise follow-up time (eg, average and total amount) | NA | |

| | | | | |
|---|---|---|---|---|
| Outcome data | 15* | *Cohort study*—Report numbers of outcome events or summary measures over time | | |
| | | *Case-control study*—Report numbers in each exposure category, or summary measures of exposure | | |
| | | *Cross-sectional study*—Report numbers of outcome events or summary measures | *2-3* | *Information Reliability and Quality … groups in terms of uploading sources (P<.001).* |
| Main results | 16 | (*a*) Give unadjusted estimates and, if applicable, confounder-adjusted estimates and their precision (eg, 95% confidence interval). Make clear which confounders were adjusted for and why they were included | 2 | *Of the 170 selected videos, the number of videos containing misleading information was 106 (62.4%); 64 (37.6%) contained useful information* |
| | | (*b*) Report category boundaries when continuous variables were categorized | NA | |
| | | (*c*) If relevant, consider translating estimates of relative risk into absolute risk for a meaningful time period | NA | |

| | | | | |
|---|---|---|---|---|
| Other analyses | 17 | Report other analyses done—eg analyses of subgroups and interactions, and sensitivity analyses | 3 | *Statistically distinctive differences between the uploading sources were observed for the reliability and global quality scores* |

| | | | | |
|---|---|---|---|---|
| Key results | 18 | Summarise key results with reference to study objectives | 4 | nearly half of the videos generated by individuals covered the topic of treatment. Alarmingly, approximately two-thirds of the videos spread misleading information. Furthermore, the quality of the videos was low. |
| Limitations | 19 | Discuss limitations of the study, taking into account sources of potential bias or imprecision. Discuss both direction and magnitude of any potential bias | 4 | A limitation of this study is that only videos … |
| Interpretation | 20 | Give a cautious overall interpretation of results considering objectives, limitations, multiplicity of analyses, results from similar studies, and other relevant evidence | 4 | *For the sake of public awareness, health care and medical professionals should adopt this technology and take effective actions to provide accurate information about* |

| | | | | |
|---|---|---|---|---|
| | | | | *breast cancer on these video websites.* |
| Generalisability | 21 | Discuss the generalisability (external validity) of the study results | 4 | *only videos in the Chinese language were examined.* |
| **Other information** | | | | |
| Funding | 22 | Give the source of funding and the role of the funders for the present study and, if applicable, for the original study on which the present article is based | NA | |

Table S107. Quality Evaluation for Included Studies Using STROBE

31.STROBE Statement—checklist of items that should be included in reports of observational studies

| | Item No. | Recommendation | Page No. | Relevant text from manuscript |
|---|---|---|---|---|
| **Title and abstract** | 1 | (*a*) Indicate the study's design with a commonly used term in the title or the abstract | 1 | Cross-sectional search |
| | | (*b*) Provide in the abstract an informative and balanced summary of what was done and what was found | 1 | *"Breast cancer" keyword was entered into YouTube™ search bar … between the two observers in terms of both DISCERN and JAMA scores.* |
| **Introduction** | | | | |
| Background/rationale | 2 | Explain the scientific background and rationale for the investigation being reported | 2 | *it is known that the majority of these patients do not discuss the information, which they obtain from the Internet, with their physicians.* |
| Objectives | 3 | State specific objectives, including any prespecified hypotheses | 2 | *investigating the quality and scientific accuracy of the most viewed first 50 videos on You-Tube™ that were accessed by using the "breast cancer" keyword.* |
| **Methods** | | | | |
| Study design | 4 | Present key elements of study design early in the paper | 2 | Cross-sectional search |
| Setting | 5 | Describe the setting, locations, and relevant dates, including periods of recruitment, exposure, follow-up, and data collection | 2 | "Breast Cancer" keyword was entered into the search bar,<br>"the most viewed" option was chosen among the search options provided by YouTube™ |
| Participants | 6 | (*a*) *Cohort study*—Give the eligibility criteria, and the sources and methods of selection of participants. Describe methods of follow-up | 2 | Advertisements, duplicate videos, and non-English videos were excluded, and the remaining 50 videos |

| | | | | |
|---|---|---|---|---|
| | | *Case-control study*—Give the eligibility criteria, and the sources and methods of case ascertainment and control selection. Give the rationale for the choice of cases and controls<br><br>*Cross-sectional study*—Give the eligibility criteria, and the sources and methods of selection of participants | | were included in the analysis. |
| | | (*b*) *Cohort study*—For matched studies, give matching criteria and number of exposed and unexposed<br><br>*Case-control study*—For matched studies, give matching criteria and the number of controls per case | NA | |
| Variables | 7 | Clearly define all outcomes, exposures, predictors, potential confounders, and effect modifiers. Give diagnostic criteria, if applicable | 2 | *Whether videos have real or animation content, uploaders, video content, video length, and the number of views, comments, likes, and dislikes were Recorded, Video Power Index (VPI) values* |
| Data sources/ measurement | 8* | For each variable of interest, give sources of data and details of methods of assessment (measurement). Describe comparability of assessment methods if there is more than one group | 2 | *Videos were from YouTube Quality and scientific accuracy of the videos were evaluated by two independent experienced radiologists* |
| Bias | 9 | Describe any efforts to address potential sources of bias | 2 | *In order to avoid bias that may be resulted from the upload date of the video on YouTube™, the mean daily view number of the videos was calculated according to the following* |
| Study size | 10 | Explain how the study size was arrived at | 2 | 50 videos |
| Quantitative variables | 11 | Explain how quantitative variables were handled in the analyses. If applicable, describe which groupings were chosen and why | 2 | *video content, video length, and the number of views, comments, likes, and dislikes were Recorded, Video Power Index (VPI) values DISCERN, JAMA* |

| | | | | |
|---|---|---|---|---|
| Statistical methods | 12 | (*a*) Describe all statistical methods, including those used to control for confounding | 3 | *The normality of the variables was analyzed with the Kolmogorov-Smirnov test. Continuous variables are expressed as mean ± standard deviation, and median (minimum-maximum), while categorical variables are expressed by number (n) and percentage (%). The comparison of DISCERN and JAMA scores between physician and nonphysician video uploaders was performed with Mann-Whitney U test. The correlation between DISCERN and JAMA scores of the observers was examined with Spearman's correlation analysis.* |
| | | (*b*) Describe any methods used to examine subgroups and interactions | NA | |
| | | (*c*) Explain how missing data were addressed | NA | |
| | | (*d*) *Cohort study*—If applicable, explain how loss to follow-up was addressed<br>*Case-control study*—If applicable, explain how matching of cases and controls was addressed<br>*Cross-sectional study*—If applicable, describe analytical methods taking account of sampling strategy | NA | |
| | | (*e*) Describe any sensitivity analyses | NA | |
| **Results** | | | | |
| Participants | 13* | (a) Report numbers of individuals at each stage of study—eg numbers potentially eligible, examined for eligibility, confirmed eligible, included in the study, completing follow-up, and analysed | 3 | When the general contents of the examined videos were evaluated; 46% (n = 23) included patient experience, 38% (n = 19) diagnosis, 10 (n = 5) nonsurgical treatment, and 6% (n = 3) surgical treatment content. |
| | | (b) Give reasons for non-participation at each stage | NA | |

| | | | | |
|---|---|---|---|---|
| | | (c) Consider use of a flow diagram | NA | |
| Descriptive data | 14* | (a) Give characteristics of study participants (eg demographic, clinical, social) and information on exposures and potential confounders | 3 | Of all videos, 35% included real and 15% animated images. … the mean JAMA score as 2:23 ± 0:97. |
| | | (b) Indicate number of participants with missing data for each variable of interest | | |
| | | (c) *Cohort study*—Summarise follow-up time (eg, average and total amount) | | |
| Outcome data | 15* | *Cohort study*—Report numbers of outcome events or summary measures over time | *3-4* | *DISCERN and JAMA scores of the first and second observers are given … DISCERN, JAMA, and VPI values (P > 0:05).* |
| | | *Case-control study*—Report numbers in each exposure category, or summary measures of exposure | | |
| | | *Cross-sectional study*—Report numbers of outcome events or summary measures | | |
| Main results | 16 | (*a*) Give unadjusted estimates and, if applicable, confounder-adjusted estimates and their precision (eg, 95% confidence interval). Make clear which confounders were adjusted for and why they were included | 3 | *the quality was found as very poor in 66% (n = 33), poor in 20% (n = 10), average in 12% (n = 6), and excellent in 2% (n = 1) of the videos. The only video evaluated as excellent was uploaded by a physician.* |
| | | (*b*) Report category boundaries when continuous variables were categorized | NA | |
| | | (*c*) If relevant, consider translating estimates of relative risk into absolute risk for a meaningful time period | NA | |

| | | | | |
|---|---|---|---|---|
| Other analyses | 17 | Report other analyses done—eg analyses of subgroups and interactions, and sensitivity analyses | 4 | When DISCERN and JAMA scores of the observers were examined by the correlation analysis; a strong statistically significant correlation was found between the two observers in terms of both DISCERN and JAMA scores |

| | | | | |
|---|---|---|---|---|
| Key results | 18 | Summarise key results with reference to study objectives | 4 | In the present study, the overall quality of the viewed videos was |

| | | | | |
|---|---|---|---|---|
| | | | | poor. We think that the low rate of videos uploaded by physicians might have played a role in this result |
| Limitations | 19 | Discuss limitations of the study, taking into account sources of potential bias or imprecision. Discuss both direction and magnitude of any potential bias | 6 | *This study has some limitations …* |
| Interpretation | 20 | Give a cautious overall interpretation of results considering objectives, limitations, multiplicity of analyses, results from similar studies, and other relevant evidence | 6 | *It is important for healthcare professionals to be aware of the video content on YouTube™ which is used by their patients.* |
| Generalisability | 21 | Discuss the generalisability (external validity) of the study results | 6 | *continuous change of YouTube™ videos might make our instant search a limitation.* |
| **Other information** | | | | |
| Funding | 22 | Give the source of funding and the role of the funders for the present study and, if applicable, for the original study on which the present article is based | NA | |

Table S108. Quality Evaluation for Included Studies Using STROBE

32.STROBE Statement—checklist of items that should be included in reports of observational studies

| | Item No. | Recommendation | Page No. | Relevant text from manuscript |
|---|---|---|---|---|
| **Title and abstract** | 1 | (*a*) Indicate the study's design with a commonly used term in the title or the abstract | 1 | Cross-sectional search |
| | | (*b*) Provide in the abstract an informative and balanced summary of what was done and what was found | 1 | Methods and Results |
| **Introduction** | | | | |
| Background/rationale | 2 | Explain the scientific background and rationale for the investigation being reported | 2 | *Due to the high volume of user traffic to YouTube, it is of paramount importance that the medical information disseminated by the site be of high quality in order to avoid misinformation. Unfortunately, previous studies have demonstrated this to not be the case.* |
| Objectives | 3 | State specific objectives, including any prespecified hypotheses | 2 | evaluate the overall quality, reliability, and educational content of YouTube videos related to spine tumors using 2 npreviously validated assessment tools. |
| **Methods** | | | | |
| Study design | 4 | Present key elements of study design early in the paper | 2 | Cross-sectional search |
| Setting | 5 | Describe the setting, locations, and relevant dates, including periods of recruitment, exposure, follow-up, and data collection | 2 | The first 50 YouTube videos identified by the keyword search "spine tumor" on 7 July 2020 were included in the study. |
| Participants | 6 | (*a*) *Cohort study*—Give the eligibility criteria, and the sources and methods of selection of participants. Describe methods of follow-up<br>*Case-control study*—Give the eligibility criteria, and the sources and methods of case ascertainment and control selection. Give the rationale for the choice of cases and controls | 2 | The first 50 YouTube videos identified by the keyword search "spine tumor" |

| | | | | |
|---|---|---|---|---|
| | | *Cross-sectional study*—Give the eligibility criteria, and the sources and methods of selection of participants | | |
| | | (*b*) *Cohort study*—For matched studies, give matching criteria and number of exposed and unexposed<br>*Case-control study*—For matched studies, give matching criteria and the number of controls per case | NA | |
| Variables | 7 | Clearly define all outcomes, exposures, predictors, potential confounders, and effect modifiers. Give diagnostic criteria, if applicable | 2 | *The following video characteristics were recorded for each video: (1) video title, …(16) whether the tumor described was primary or metastatic.* |
| Data sources/ measurement | 8* | For each variable of interest, give sources of data and details of methods of assessment (measurement). Describe comparability of assessment methods if there is more than one group | *2* | *Videos were from YouTube* |
| Bias | 9 | Describe any efforts to address potential sources of bias | NA | |
| Study size | 10 | Explain how the study size was arrived at | 2 | 50 videos |
| Quantitative variables | 11 | Explain how quantitative variables were handled in the analyses. If applicable, describe which groupings were chosen and why | 2 | *(2) duration, (3) number of views, (4) number of likes, (5) number of dislikes, (6) number of comments, (7) days since upload, (8) like ratio (no. of likes/[no. of likes + no. of dislikes] × 100), (9) view ratio (no. of views/day), (10) the Video Power Index (VPI), JAMA, GQS* |
| Statistical methods | 12 | (*a*) Describe all statistical methods, including those used to control for confounding | 2 | *Descriptive statistics were calculated and reported for all video characteristics and scores. Continuous variables were reported as means with standard deviation or ranges, while categorical variables were reported as counts with percentages. Video characteristic associations with the JAMA score, GQS, and VPI* |

| | | | | |
|---|---|---|---|---|
| | | | | *were determined by multivariable linear regression.* |
| | | (*b*) Describe any methods used to examine subgroups and interactions | NA | |
| | | (*c*) Explain how missing data were addressed | NA | |
| | | (*d*) *Cohort study*—If applicable, explain how loss to follow-up was addressed<br>*Case-control study*—If applicable, explain how matching of cases and controls was addressed<br>*Cross-sectional study*—If applicable, describe analytical methods taking account of sampling strategy | NA | |
| | | (*e*) Describe any sensitivity analyses | NA | |
| **Results** | | | | |
| Participants | 13* | (a) Report numbers of individuals at each stage of study—eg numbers potentially eligible, examined for eligibility, confirmed eligible, included in the study, completing follow-up, and analysed | 2 | Fifty videos were included in the study. |
| | | (b) Give reasons for non-participation at each stage | NA | |
| | | (c) Consider use of a flow diagram | NA | |
| Descriptive data | 14* | (a) Give characteristics of study participants (eg demographic, clinical, social) and information on exposures and potential confounders | 2-3 | The mean video duration was 8.1 ± 12 minutes (range: 0.87−57 minutes), … while 44% reported potential risks or complications (Table 4). |
| | | (b) Indicate number of participants with missing data for each variable of interest | NA | |
| | | (c) *Cohort study*—Summarise follow-up time (eg, average and total amount) | NA | |
| Outcome data | 15* | *Cohort study*—Report numbers of outcome events or summary measures over time | | |
| | | *Case-control study*—Report numbers in each exposure category, or summary measures of exposure | | |
| | | *Cross-sectional study*—Report numbers of outcome events or summary measures | *4* | *The mean ± SD JAMA score and GQS were 3.1 ± 0.27 (range: 3–4) and 2.6 ± 1.3 (range: 1–5), respectively.* |

| | | | | |
|---|---|---|---|---|
| Main results | 16 | (*a*) Give unadjusted estimates and, if applicable, confounder-adjusted estimates and their precision (eg, 95% confidence interval). Make clear which confounders were adjusted for and why they were included | 4 | *Video duration and number of dislikes were associated with higher GQS (β = 0.041, P = 0.025) and lower GQS (β = −0.189, P = 0.04), respectively.* |
| | | (*b*) Report category boundaries when continuous variables were categorized | NA | |
| | | (*c*) If relevant, consider translating estimates of relative risk into absolute risk for a meaningful time period | NA | |
| Other analyses | 17 | Report other analyses done—eg analyses of subgroups and interactions, and sensitivity analyses | 4 | Multivariate linear regression analyses revealed that video duration (β = 0.00697, P = 0.04) and number of views (β= 0.000018, P = 0.001) were positively associated with JAMA score. |
| Key results | 18 | Summarise key results with reference to study objectives | 4 | the reliability, quality, and educational content of YouTube videos analyzed in this study were poor to suboptimal |
| Limitations | 19 | Discuss limitations of the study, taking into account sources of potential bias or imprecision. Discuss both direction and magnitude of any potential bias | 5 | *Readers should consider several limitations when interpreting the findings presented in this study …* |
| Interpretation | 20 | Give a cautious overall interpretation of results considering objectives, limitations, multiplicity of analyses, results from similar studies, and other relevant evidence | 5 | *Patients should be wary of the education provided by YouTube videos on spine tumors and seek out additional sources of information.* |
| Generalisability | 21 | Discuss the generalisability (external validity) of the study results | 5 | *the active nature of the internet means that certain video characteristics, including the top 50 videos as well as the number of likes, dislikes, and comments, are constantly subject to change.* |
| **Other information** | | | | |

| | | | | |
|---|---|---|---|---|
| Funding | 22 | Give the source of funding and the role of the funders for the present study and, if applicable, for the original study on which the present article is based | 6 | *The author(s) received no financial support for the research, authorship, and/or publication of this article.* |

Table S109. Quality Evaluation for Included Studies Using STROBE

33.STROBE Statement—checklist of items that should be included in reports of observational studies

| | Item No. | Recommendation | Page No. | Relevant text from manuscript |
|---|---|---|---|---|
| **Title and abstract** | 1 | (*a*) Indicate the study's design with a commonly used term in the title or the abstract | 1 | Cross-sectional search |
| | | (*b*) Provide in the abstract an informative and balanced summary of what was done and what was found | 1 | Method and Results |
| **Introduction** | | | | |
| Background/rationale | 2 | Explain the scientific background and rationale for the investigation being reported | 1 | *new and diverse techniques are being introduced regularly, making social media an affordable, easy-to-access continuous platform on which surgeons from different parts of the world can share their techniques and enhance their skills.* |
| Objectives | 3 | State specific objectives, including any prespecified hypotheses | 2 | assess the content, reliability and quality of the most-viewed YouTube videos teaching rectal cancer surgery techniques and determine whether watching these videos is useful to surgeons. |
| **Methods** | | | | |
| Study design | 4 | Present key elements of study design early in the paper | 2 | Cross-sectional search |
| Setting | 5 | Describe the setting, locations, and relevant dates, including periods of recruitment, exposure, follow-up, and data collection | 2 | search on https://www.youtube.com/ using the keywords "rectal cancer surgery" via open internet access on December 4, 2020. All videos were assessed independently by two physicians. |
| Participants | 6 | (*a*) *Cohort study*—Give the eligibility criteria, and the sources and methods of selection of participants. Describe methods of follow-up | 2 | Those unrelated to rectal cancer surgery were excluded from the study. Among those relevant to |

| | | | | |
|---|---|---|---|---|
| | | *Case-control study*—Give the eligibility criteria, and the sources and methods of case ascertainment and control selection. Give the rationale for the choice of cases and controls<br><br>*Cross-sectional study*—Give the eligibility criteria, and the sources and methods of selection of participants | | rectal cancer, any videos other than those related mainly to surgery, those focused on surgical techniques, and those of an instructive nature were also excluded. |
| | | (*b*) *Cohort study*—For matched studies, give matching criteria and number of exposed and unexposed<br><br>*Case-control study*—For matched studies, give matching criteria and the number of controls per case | NA | |
| Variables | 7 | Clearly define all outcomes, exposures, predictors, potential confounders, and effect modifiers. Give diagnostic criteria, if applicable | 2 | *Video length (min), number of views, peer-review status, source of upload (uploader: non-profit organizations/for-profit organizations/individual surgeons), video quality (good/moderate/poor), date of upload, quality score, reliability score, content (comprehensiveness) score, type of surgical technique (lap/robotic/open), number of likes, number of dislikes, number of comments, and duration on YouTube (days) were recorded.* |
| Data sources/ measurement | 8* | For each variable of interest, give sources of data and details of methods of assessment (measurement). Describe comparability of assessment methods if there is more than one group | *2* | *Videos were from YouTube* |
| Bias | 9 | Describe any efforts to address potential sources of bias | 2 | *Since search results on YouTube can change on a daily basis, the videos were saved in a playlist.* |
| Study size | 10 | Explain how the study size was arrived at | 3 | 167 videos |

| | | | | |
|---|---|---|---|---|
| Quantitative variables | 11 | Explain how quantitative variables were handled in the analyses. If applicable, describe which groupings were chosen and why | 2 | *Video length (min), number of views, number of likes, number of dislikes, number of* |

| | | | | |
|---|---|---|---|---|
| | | | | *comments, and duration on YouTube* |
| Statistical methods | 12 | (*a*) Describe all statistical methods, including those used to control for confounding | 2 | *Descriptive statistics (mean, standard deviation, …* variables without a normal distribution. |
| | | (*b*) Describe any methods used to examine subgroups and interactions | 3 | Table 2 |
| | | (*c*) Explain how missing data were addressed | NA | |
| | | (*d*) *Cohort study*—If applicable, explain how loss to follow-up was addressed<br>*Case-control study*—If applicable, explain how matching of cases and controls was addressed<br>*Cross-sectional study*—If applicable, describe analytical methods taking account of sampling strategy | NA | |
| | | (*e*) Describe any sensitivity analyses | NA | |
| **Results** | | | | |
| Participants | 13* | (a) Report numbers of individuals at each stage of study—eg numbers potentially eligible, examined for eligibility, confirmed eligible, included in the study, completing follow-up, and analysed | 3 | The study included 167 videos |
| | | (b) Give reasons for non-participation at each stage | NA | |
| | | (c) Consider use of a flow diagram | NA | |
| Descriptive data | 14* | (a) Give characteristics of study participants (eg demographic, clinical, social) and information on exposures and potential confounders | 3 | Of these, 64.1% (n=107) were uploaded by individual surgeons, 25.7% (n=43) by non-profit organizations, and 10.2% (n=17) by for-profit organizations. The rate of useful information and misleading information was 52.1% (n=87) and 47.9% (n=80), respectively. The YouTube duration was significantly longer for misleading videos than for useful information videos. |
| | | (b) Indicate number of participants with missing data for each variable of interest | NA | |
| | | (c) *Cohort study*—Summarise follow-up time (eg, average and total amount) | NA | |

| | | | | |
|---|---|---|---|---|
| Outcome data | 15* | *Cohort study*—Report numbers of outcome events or summary measures over time | | |
| | | *Case-control study*—Report numbers in each exposure category, or summary measures of exposure | | |
| | | *Cross-sectional study*—Report numbers of outcome events or summary measures | *3* | *Useful information videos had a significantly … videos, and 8 robotic videos in group 2.* |
| Main results | 16 | (*a*) Give unadjusted estimates and, if applicable, confounder-adjusted estimates and their precision (eg, 95% confidence interval). Make clear which confounders were adjusted for and why they were included | 3 | *The YouTube duration was significantly longer for misleading videos than for useful information videos. Useful information videos had a significantly higher number of views per day, likes, dislikes, and comments* |
| | | (*b*) Report category boundaries when continuous variables were categorized | NA | |
| | | (*c*) If relevant, consider translating estimates of relative risk into absolute risk for a meaningful time period | NA | |

| | | | | |
|---|---|---|---|---|
| Other analyses | 17 | Report other analyses done—eg analyses of subgroups and interactions, and sensitivity analyses | 3 | Subgroups for different variables |

| | | | | |
|---|---|---|---|---|
| Key results | 18 | Summarise key results with reference to study objectives | 4 | video uploads by individual surgeons were longer, videos by for-profit organizations had more total views, and videos by non-profit organizations had more views per day. |
| Limitations | 19 | Discuss limitations of the study, taking into account sources of potential bias or imprecision. Discuss both direction and magnitude of any potential bias | 5 | This study has several limitations… |
| Interpretation | 20 | Give a cautious overall interpretation of results considering objectives, limitations, multiplicity of analyses, results from similar studies, and other relevant evidence | 5 | *social media should be considered a common learning domain, and videos of high levels of instruction, reliability, and quality should be uploaded to social media platforms by competent people, groups, and institutions.* |

| | | | | |
|---|---|---|---|---|
| Generalisability | 21 | Discuss the generalisability (external validity) of the study results | 5 | *In addition to the low number of videos and the relative assessment of those videos, the study only included videos on YouTube in the English language.* |
| **Other information** | | | | |
| Funding | 22 | Give the source of funding and the role of the funders for the present study and, if applicable, for the original study on which the present article is based | 5 | The authors declared that this study received no financial support. |

Table S110. Quality Evaluation for Included Studies Using STROBE

34.STROBE Statement—checklist of items that should be included in reports of observational studies

| | Item No. | Recommendation | Page No. | Relevant text from manuscript |
|---|---|---|---|---|
| **Title and abstract** | 1 | (*a*) Indicate the study's design with a commonly used term in the title or the abstract | 1 | Cross-sectional search |
| | | (*b*) Provide in the abstract an informative and balanced summary of what was done and what was found | 1 | Methods and Results |
| **Introduction** | | | | |
| Background/rationale | 2 | Explain the scientific background and rationale for the investigation being reported | 1 | *The inadequate rate of CRC screening due to lack of social awareness presents a difficulty for some screening methods (e.g., preparation for colon cleansing is required before a colonoscopy) [9].*<br>*Today, the internet is almost the first point of reference for learning about health problems.* |
| Objectives | 3 | State specific objectives, including any prespecified hypotheses | 2 | assess the content, quality, and reliability of YouTube videos on colorectal cancer screening. |
| **Methods** | | | | |
| Study design | 4 | Present key elements of study design early in the paper | 2 | Cross-sectional search |
| Setting | 5 | Describe the setting, locations, and relevant dates, including periods of recruitment, exposure, follow-up, and data collection | 2 | A basic search was conducted on the YouTube website on November 19, 2020, using the English keywords "colorectal cancer screening," |
| Participants | 6 | (*a*) *Cohort study*—Give the eligibility criteria, and the sources and methods of selection of participants. Describe methods of follow-up<br>*Case-control study*—Give the eligibility criteria, and the sources and methods of case ascertainment and control selection. Give the rationale for the choice of cases and controls | 2 | Videos with no audio English narration (n=7), those not related to colorectal cancer screening (n=17), duplicate videos (n=16), image only videos without voice, or |

| | | | | |
|---|---|---|---|---|
| | | *Cross-sectional study*—Give the eligibility criteria, and the sources and methods of selection of participants | | audio only without images (n=12), and videos of less than 30 seconds (n=10) were not included in the study. Videos consisting of multiple parts were regarded as a single video (n=1). |
| | | (*b*) *Cohort study*—For matched studies, give matching criteria and number of exposed and unexposed<br>*Case-control study*—For matched studies, give matching criteria and the number of controls per case | NA | |
| Variables | 7 | Clearly define all outcomes, exposures, predictors, potential confounders, and effect modifiers. Give diagnostic criteria, if applicable | 2 | *The total number of views, the length, time elapsed after uploading and the numbers of likes, dislikes and comments were recorded for each video. The engagement of viewers with the video was calculated in terms of the number of views per day* |
| Data sources/ measurement | 8* | For each variable of interest, give sources of data and details of methods of assessment (measurement). Describe comparability of assessment methods if there is more than one group | *2* | *Videos were from YouTube*<br>*Evaluation of the videos' usefulness All 138 videos were independently evaluated* |
| Bias | 9 | Describe any efforts to address potential sources of bias | 2 | *Evaluation of the videos' usefulness All 138 videos were independently evaluated* |
| Study size | 10 | Explain how the study size was arrived at | 2 | 137 videos |
| Quantitative variables | 11 | Explain how quantitative variables were handled in the analyses. If applicable, describe which groupings were chosen and why | 2 | *and the numbers of likes, dislikes and comments were recorded for each video. The engagement of viewers with the video DISCERN scale and GQS* |
| | 12 | (*a*) Describe all statistical methods, including those used to control for confounding | 3 | *Since the normality distribution was not met by any variables,* |

| | | | | |
|---|---|---|---|---|
| Statistical methods | | | | *the comparisons were performed by using the non-parametric Mann-Whitney test. In the statistical evaluation conducted according to the source of the video, the multiple non-parametric Kruskal-Wallis test was preferred because no variables met the normality distribution assumption.* |
| | | (*b*) Describe any methods used to examine subgroups and interactions | 4 | Table 2 |
| | | (*c*) Explain how missing data were addressed | NA | |
| | | (*d*) *Cohort study*—If applicable, explain how loss to follow-up was addressed<br>*Case-control study*—If applicable, explain how matching of cases and controls was addressed<br>*Cross-sectional study*—If applicable, describe analytical methods taking account of sampling strategy | NA | |
| | | (*e*) Describe any sensitivity analyses | NA | |
| **Results** | | | | |
| Participants | 13* | (a) Report numbers of individuals at each stage of study—eg numbers potentially eligible, examined for eligibility, confirmed eligible, included in the study, completing follow-up, and analysed | 2 | Videos with no audio English narration (n=7), those not related to colorectal cancer screening (n=17), duplicate videos (n=16), image only videos without voice, or audio only without images (n=12), and videos of less than 30 seconds (n=10) were not included in the study. Videos consisting of multiple parts were regarded as a single video (n=1). As a result of the search, 137 videos were included in the study. |
| | | (b) Give reasons for non-participation at each stage | NA | |
| | | (c) Consider use of a flow diagram | NA | |

| | | | | |
|---|---|---|---|---|
| Descriptive data | 14* | (a) Give characteristics of study participants (eg demographic, clinical, social) and information on exposures and potential confounders | 3 | The comparison test in terms … than the videos with misleading information. |
| | | (b) Indicate number of participants with missing data for each variable of interest | NA | |
| | | (c) *Cohort study*—Summarise follow-up time (eg, average and total amount) | NA | |
| Outcome data | 15* | *Cohort study*—Report numbers of outcome events or summary measures over time | | |
| | | *Case-control study*—Report numbers in each exposure category, or summary measures of exposure | | |
| | | *Cross-sectional study*—Report numbers of outcome events or summary measures | *6-7* | A significant difference was observed between … public spotlight videos had the lowest. |
| Main results | 16 | (*a*) Give unadjusted estimates and, if applicable, confounder-adjusted estimates and their precision (eg, 95% confidence interval). Make clear which confounders were adjusted for and why they were included | 6-7 | *A significant difference was observed between the video upload source groups for the reliability score. It was observed that there was a significant difference between the video upload source groups in terms of the GQS quality score.* |
| | | (*b*) Report category boundaries when continuous variables were categorized | NA | |
| | | (*c*) If relevant, consider translating estimates of relative risk into absolute risk for a meaningful time period | NA | |

| | | | | |
|---|---|---|---|---|
| Other analyses | 17 | Report other analyses done—eg analyses of subgroups and interactions, and sensitivity analyses | 7 | there was a significant relationship between groups (useful information group and misleading information group) and the video sources |

| | | | | |
|---|---|---|---|---|
| Key results | 18 | Summarise key results with reference to study objectives | 7 | These studies conclude that more comprehensive videos are needed. In addition, they emphasize that the number of views for videos on CRC screening is low and should be increased. |

| | | | | |
|---|---|---|---|---|
| Limitations | 19 | Discuss limitations of the study, taking into account sources of potential bias or imprecision. Discuss both direction and magnitude of any potential bias | 8 | Study limitatinos… |
| Interpretation | 20 | Give a cautious overall interpretation of results considering objectives, limitations, multiplicity of analyses, results from similar studies, and other relevant evidence | 8 | *the CRC screening rate could increase by collaborating with YouTube (reaching more people by means of algorithms), enriching the public spotlight videos with useful information, keeping academic videos short and more viewable.* |
| Generalisability | 21 | Discuss the generalisability (external validity) of the study results | 8 | *only English language videos were evaluated, making generalization of the results difficult.* |
| **Other information** | | | | |
| Funding | 22 | Give the source of funding and the role of the funders for the present study and, if applicable, for the original study on which the present article is based | 8 | *Financial relationships: All authors have declared that they have no financial relationships at present or within the previous three years with any organizations that might have an interest in the submitted work.* |

Table S111. Quality Evaluation for Included Studies Using STROBE

35.STROBE Statement—checklist of items that should be included in reports of observational studies

| | Item No. | Recommendation | Page No. | Relevant text from manuscript |
|---|---|---|---|---|
| **Title and abstract** | 1 | (*a*) Indicate the study's design with a commonly used term in the title or the abstract | NA | |
| | | (*b*) Provide in the abstract an informative and balanced summary of what was done and what was found | NA | |
| **Introduction** | | | | |
| Background/rationale | 2 | Explain the scientific background and rationale for the investigation being reported | 1 | *Distinguishing between high- and low-quality content becomes more difficult as the number of videos increases. Recently, videos addressing patients with melanoma or basal cell carcinoma (BCC) were found to be of predominantly mediocre quality and poor reliability* |
| Objectives | 3 | State specific objectives, including any prespecified hypotheses | 1 | systematically identify and evaluate videos on cSCC, the worldwide second most common type of skin cancer after BCC |
| **Methods** | | | | |
| Study design | 4 | Present key elements of study design early in the paper | 1 | Cross-sectional search |
| Setting | 5 | Describe the setting, locations, and relevant dates, including periods of recruitment, exposure, follow-up, and data collection | 1 | In April 2021, we conducted a systematic search on www.youtube.com for cSCC videos. The search string included German synonyms of cSCC (Plattenepithelkarzinom |
| Participants | 6 | (*a*) *Cohort study*—Give the eligibility criteria, and the sources and methods of selection of participants. Describe methods of follow-up<br>*Case-control study*—Give the eligibility criteria, and the sources and methods of case ascertainment and control selection. Give the rationale for the choice of cases and controls | 1 | Videos had to be in German. Exclusion criteria were: (1) skin cancer in animals, (2) advertising, (3) videos without sound, (4) irrelevant content, e.g. music videos or cartoons, (5) videos for a |

| | | | | |
|---|---|---|---|---|
| | | *Cross-sectional study*—Give the eligibility criteria, and the sources and methods of selection of participants | | professional audience only, (6) duration less than one minute. |
| | | (*b*) *Cohort study*—For matched studies, give matching criteria and number of exposed and unexposed<br>*Case-control study*—For matched studies, give matching criteria and the number of controls per case | NA | |
| Variables | 7 | Clearly define all outcomes, exposures, predictors, potential confounders, and effect modifiers. Give diagnostic criteria, if applicable | NA | |
| Data sources/ measurement | 8* | For each variable of interest, give sources of data and details of methods of assessment (measurement). Describe comparability of assessment methods if there is more than one group | *1* | *Videos were from YouTube two researchers and one patient assessed the videos using validated instruments.* |
| Bias | 9 | Describe any efforts to address potential sources of bias | 1 | *two researchers and one patient assessed the videos using validated instruments* |
| Study size | 10 | Explain how the study size was arrived at | 1 | 16 videos |
| Quantitative variables | 11 | Explain how quantitative variables were handled in the analyses. If applicable, describe which groupings were chosen and why | 1 | *DISCERN and the Global Quality Scale (GQS), PEMAT* |
| Statistical methods | 12 | (*a*) Describe all statistical methods, including those used to control for confounding | 1 | *Descriptive analysis* |
| | | (*b*) Describe any methods used to examine subgroups and interactions | NA | |
| | | (*c*) Explain how missing data were addressed | NA | |
| | | (*d*) *Cohort study*—If applicable, explain how loss to follow-up was addressed<br>*Case-control study*—If applicable, explain how matching of cases and controls was addressed<br>*Cross-sectional study*—If applicable, describe analytical methods taking account of sampling strategy | NA | |
| | | (*e*) Describe any sensitivity analyses | NA | |

**Results**

| | | | | |
|---|---|---|---|---|
| Participants | 13* | (a) Report numbers of individuals at each stage of study—eg numbers potentially eligible, examined for eligibility, confirmed eligible, included in the study, completing follow-up, and analysed | 1 | *Sixteen out of 387 videos met the eligibility criteria* *(Figure 1).* |
| | | (b) Give reasons for non-participation at each stage | NA | |
| | | (c) Consider use of a flow diagram | 1 | Figure 1 |
| Descriptive data | 14* | (a) Give characteristics of study participants (eg demographic, clinical, social) and information on exposures and potential confounders | 2 | Most videos were uploaded by private health channels (6/16), followed by hospitals (4/16), non-commercial providers (2/16), private channels (2/16) and TV reports provided by private channels (1/16). One provider remained unclear. The number of views ranged from 62 to 56,291 (mean: 5,419), indicating a wide range of reach. |
| | | (b) Indicate number of participants with missing data for each variable of interest | NA | |
| | | (c) *Cohort study*—Summarise follow-up time (eg, average and total amount) | NA | |
| Outcome data | 15* | *Cohort study*—Report numbers of outcome events or summary measures over time | | |
| | | *Case-control study*—Report numbers in each exposure category, or summary measures of exposure | | |
| | | *Cross-sectional study*—Report numbers of outcome events or summary measures | *2* | *Videos achieved a mean DISCERN score of 28.5 point … None of the videos were rated as harmful.* |
| Main results | 16 | (*a*) Give unadjusted estimates and, if applicable, confounder-adjusted estimates and their precision (eg, 95% confidence interval). Make clear which confounders were adjusted for and why they were included | 2 | *Videos achieved a mean DISCERN score of 28.5 points out of 45 (Table 1), indicating a low to mediocre quality* |
| | | (*b*) Report category boundaries when continuous variables were categorized | NA | |
| | | (*c*) If relevant, consider translating estimates of relative risk into absolute risk for a meaningful time period | NA | |

| | | | | |
|---|---|---|---|---|
| Other analyses | 17 | Report other analyses done—eg analyses of subgroups and interactions, and sensitivity analyses | NA | |
| Key results | 18 | Summarise key results with reference to study objectives | 2 | the videos examined showed that the quality and especially the reliability of the videos were deficient. |
| Limitations | 19 | Discuss limitations of the study, taking into account sources of potential bias or imprecision. Discuss both direction and magnitude of any potential bias | NA | |
| Interpretation | 20 | Give a cautious overall interpretation of results considering objectives, limitations, multiplicity of analyses, results from similar studies, and other relevant evidence | 3 | *physicians should advise patients not to rely on YouTube videos on cSCC and encourage them to check the sources carefully as well as the timeliness of the videos.* |
| Generalisability | 21 | Discuss the generalisability (external validity) of the study results | NA | |
| **Other information** | | | | |
| Funding | 22 | Give the source of funding and the role of the funders for the present study and, if applicable, for the original study on which the present article is based | 3 | *Funding for the study was provided by a grant from the German Skin Cancer Council* |

Table S112. Quality Evaluation for Included Studies Using STROBE

36.STROBE Statement—checklist of items that should be included in reports of observational studies

| | Item No. | Recommendation | Page No. | Relevant text from manuscript |
|---|---|---|---|---|
| **Title and abstract** | 1 | (*a*) Indicate the study's design with a commonly used term in the title or the abstract | 1 | *cross-sectional study,* |
| | | (*b*) Provide in the abstract an informative and balanced summary of what was done and what was found | 1 | Method and materials<br>Results |
| **Introduction** | | | | |
| Background/rationale | 2 | Explain the scientific background and rationale for the investigation being reported | 2 | *YouTube has become an important way to provide health care information; it also offers the public an unlimited opportunity to express their views and feelings.20 The possibility of limited quality evaluation of the produced contents raises the risk of misleading information* |
| Objectives | 3 | State specific objectives, including any prespecified hypotheses | 2 | *assess videos about the oral care of leukemia patients on YouTube.* |
| **Methods** | | | | |
| Study design | 4 | Present key elements of study design early in the paper | 2 | Cross-sectional search |
| Setting | 5 | Describe the setting, locations, and relevant dates, including periods of recruitment, exposure, follow-up, and data collection | 2 | The data extraction from the YouTube API video feed was performed on 24 December 2018. |
| Participants | 6 | (*a*) *Cohort study*—Give the eligibility criteria, and the sources and methods of selection of participants. Describe methods of follow-up<br>*Case-control study*—Give the eligibility criteria, and the sources and methods of case ascertainment and control selection. Give the rationale for the choice of cases and controls<br>*Cross-sectional study*—Give the eligibility criteria, and the sources and methods of selection of participants | 2 | Videos that were partially or completely copied were discarded, as were videos other than English, silent videos, those deemed irrelevant, and videos over 15 minutes. Multipart videos were counted separately. |

| | | | | |
|---|---|---|---|---|
| | | (*b*) *Cohort study*—For matched studies, give matching criteria and number of exposed and unexposed<br>*Case-control study*—For matched studies, give matching criteria and the number of controls per case | | |
| Variables | 7 | Clearly define all outcomes, exposures, predictors, potential confounders, and effect modifiers. Give diagnostic criteria, if applicable | 2-3 | *resource locator*<br>*(URL), title, date of upload, duration, total views, number of likes, number of dislikes, and number of comments.*<br>*interaction index,16 viewing rate … and oral care of leukemia.* |
| Data sources/ measurement | 8* | For each variable of interest, give sources of data and details of methods of assessment (measurement). Describe comparability of assessment methods if there is more than one group | *3* | *Videos were from YouTube*<br>*Two reviewers (GAS, EE) assessed the videos independently and evaluated the characteristics and attributes of each video* |
| Bias | 9 | Describe any efforts to address potential sources of bias | 3 | *Two reviewers (GAS, EE) assessed the videos independently and evaluated the characteristics and attributes of each video* |
| Study size | 10 | Explain how the study size was arrived at | 4 | 80 videos |

| | | | | |
|---|---|---|---|---|
| Quantitative variables | 11 | Explain how quantitative variables were handled in the analyses. If applicable, describe which groupings were chosen and why | 3 | *GQS, Interaction index viewing rate, total views, number of likes, number of dislikes, and number of comments.* |
| Statistical methods | 12 | (*a*) Describe all statistical methods, including those used to control for confounding | 3 | *A descriptive study was made for each variable. Variables were tested for normality using the Shapiro-Wilk test. Differences between variables were assessed using the Kruskal-Wallis test. Statistical significance was set at P < .05.* |

| | | | | |
|---|---|---|---|---|
| | | | | *Post hoc analysis was assessed using the Mann-Whitney U test.* |
| | | (*b*) Describe any methods used to examine subgroups and interactions | 3 | *Differences between variables were assessed using the Kruskal-Wallis test* |
| | | (*c*) Explain how missing data were addressed | NA | |
| | | (*d*) *Cohort study*—If applicable, explain how loss to follow-up was addressed<br>*Case-control study*—If applicable, explain how matching of cases and controls was addressed<br>*Cross-sectional study*—If applicable, describe analytical methods taking account of sampling strategy | NA | |
| | | (*e*) Describe any sensitivity analyses | NA | |
| **Results** | | | | |
| Participants | 13* | (a) Report numbers of individuals at each stage of study—eg numbers potentially eligible, examined for eligibility, confirmed eligible, included in the study, completing follow-up, and analysed | 3 | *The first 200 videos were evaluated for relevance based on the selection criteria. The following categories were excluded: non-English videos (17), irrelevant videos (66), duplicates (whole/ partial) (2), silent video (1), and videos over 15 minutes (34). The remaining 80 videos were classified according to their video type as patient experience (12; 15.0%), educational (64; 80.0%), and scientifically erroneous or unproven information (4; 5.0%)* |
| | | (b) Give reasons for non-participation at each stage | NA | |
| | | (c) Consider use of a flow diagram | 4 | Fig 1 |
| Descriptive data | 14* | (a) Give characteristics of study participants (eg demographic, clinical, social) and information on exposures and potential confounders | 3-4 | The remaining 80 videos were classified according to their video type … classified as a patient's personal views (Table 2). |
| | | (b) Indicate number of participants with missing data for each variable of interest | NA | |

| | | | | |
|---|---|---|---|---|
| | | (c) *Cohort study*—Summarise follow-up time (eg, average and total amount) | NA | |
| Outcome data | 15* | *Cohort study*—Report numbers of outcome events or summary measures over time | | |
| | | *Case-control study*—Report numbers in each exposure category, or summary measures of exposure | | |
| | | *Cross-sectional study*—Report numbers of outcome events or summary measures | *4* | *The quality score of the videos revealed that 13 videos (16.3%) were assessed as poor, 17 (21.3%) as generally poor,…* |
| Main results | 16 | (*a*) Give unadjusted estimates and, if applicable, confounder-adjusted estimates and their precision (eg, 95% confidence interval). Make clear which confounders were adjusted for and why they were included | 4 | *Among the selected videos, 63 (78.8%) were classified as useful, 6 (7.5%) as misleading, and 11 (13.8%) were classified as a patient's personal views* |
| | | (*b*) Report category boundaries when continuous variables were categorized | NA | |
| | | (*c*) If relevant, consider translating estimates of relative risk into absolute risk for a meaningful time period | NA | |

| | | | | |
|---|---|---|---|---|
| Other analyses | 17 | Report other analyses done—eg analyses of subgroups and interactions, and sensitivity analyses | 4 | The Kruskal-Wallis test showed no significant correlation between the content of useful/misleading/personal experience and interaction index, viewing rate, and days since upload |

| | | | | |
|---|---|---|---|---|
| Key results | 18 | Summarise key results with reference to study objectives | 5 | In the present study, the included videos were recorded according to their mention of leukemia types. |
| Limitations | 19 | Discuss limitations of the study, taking into account sources of potential bias or imprecision. Discuss both direction and magnitude of any potential bias | 6 | *The present study has some limitations. …* |
| Interpretation | 20 | Give a cautious overall interpretation of results considering objectives, limitations, multiplicity of analyses, results from similar studies, and other relevant evidence | 7 | *Health care professionals should be aware that YouTube has the potential to impact patients, and should be responsible for* |

| | | | | |
|---|---|---|---|---|
| | | | | *improving the content of videos about the oral care of leukemia.* |
| Generalisability | 21 | Discuss the generalisability (external validity) of the study results | 6 | *YouTube is a highly dynamic platform and videos can be uploaded and deleted in a very short time.* |
| **Other information** | | | | |
| Funding | 22 | Give the source of funding and the role of the funders for the present study and, if applicable, for the original study on which the present article is based | 7 | *No funding sources supported the study.* |

Table S113. Quality Evaluation for Included Studies Using STROBE

37.STROBE Statement—checklist of items that should be included in reports of observational studies

| | Item No. | Recommendation | Page No. | Relevant text from manuscript |
|---|---|---|---|---|
| **Title and abstract** | 1 | (*a*) Indicate the study's design with a commonly used term in the title or the abstract | 1 | Quality assessment |
| | | (*b*) Provide in the abstract an informative and balanced summary of what was done and what was found | 1 | A simple Python tool was developed using YouTube API V3 … and the dimensions of the two tools. |
| **Introduction** | | | | |
| Background/rationale | 2 | Explain the scientific background and rationale for the investigation being reported | 2 | *It has been shown that such platforms influence treatment choices and affects the decision-making processes for patients and their relatives* |
| Objectives | 3 | State specific objectives, including any prespecified hypotheses | 5 | *analyze the accuracy of information and quality of Arabic YouTube video content related to herbal cancer treatment using two reliable instruments: the Patient Education Materials Assessment Tool (PEMAT) and the DISCERN tool.* |
| **Methods** | | | | |
| Study design | 4 | Present key elements of study design early in the paper | 6 | Cross-sectional search |
| Setting | 5 | Describe the setting, locations, and relevant dates, including periods of recruitment, exposure, follow-up, and data collection | 6 | The authors developed a Python tool using API V351 that automated the YouTube search to minimize human interaction and bias. The developed tool was programmed to retrieve the most relevant videos for a search term. |
| Participants | 6 | (*a*) *Cohort study*—Give the eligibility criteria, and the sources and methods of selection of participants. Describe methods of follow-up | 6 | Of the 200 selected videos, 90 videos have been excluded: 31 because they |

| | | | | |
|---|---|---|---|---|
| | | *Case-control study*—Give the eligibility criteria, and the sources and methods of case ascertainment and control selection. Give the rationale for the choice of cases and controls<br><br>*Cross-sectional study*—Give the eligibility criteria, and the sources and methods of selection of participants | | were advertisements, 18 repeated videos, 3 with errors, and 38 unrelated content. The remaining 110 video list was then subject to quantitative and qualitative analysis and assessment. |
| | | (*b*) *Cohort study*—For matched studies, give matching criteria and number of exposed and unexposed<br><br>*Case-control study*—For matched studies, give matching criteria and the number of controls per case | NA | |
| Variables | 7 | Clearly define all outcomes, exposures, predictors, potential confounders, and effect modifiers. Give diagnostic criteria, if applicable | 6 | *The coding extracted included the video title, URL, duration, upload date, number of views, likes, dislikes, comments, and associated tags.* |
| Data sources/ measurement | 8* | For each variable of interest, give sources of data and details of methods of assessment (measurement). Describe comparability of assessment methods if there is more than one group | *6* | *Videos were from YouTube*<br><br>*To ensure unbiased findings for this study, the quality of information for each video of the 110 was coded according to the DISCERN and PEMAT criteria by two researchers working independently* |
| Bias | 9 | Describe any efforts to address potential sources of bias | 6 | The authors developed a Python tool using API V351 that automated the YouTube search to minimize human interaction and bias.<br><br>*To ensure unbiased findings for this study, the quality of information for each video of the 110 was coded according to the DISCERN and PEMAT criteria by two researchers working independently* |
| Study size | 10 | Explain how the study size was arrived at | 6 | 110 videos |

| | | | | |
|---|---|---|---|---|
| Quantitative variables | 11 | Explain how quantitative variables were handled in the analyses. If applicable, describe which groupings were chosen and why | 6 | *duration, upload date, number of views, likes, dislikes, comments,*<br><br>*DISCERN, PEMAT* |
| Statistical methods | 12 | (*a*) Describe all statistical methods, including those used to control for confounding | 7 | *descriptive* |
| | | (*b*) Describe any methods used to examine subgroups and interactions | NA | |
| | | (*c*) Explain how missing data were addressed | NA | |
| | | (*d*) *Cohort study*—If applicable, explain how loss to follow-up was addressed<br><br>*Case-control study*—If applicable, explain how matching of cases and controls was addressed<br><br>*Cross-sectional study*—If applicable, describe analytical methods taking account of sampling strategy | NA | |
| | | (*e*) Describe any sensitivity analyses | NA | |
| **Results** | | | | |
| Participants | 13* | (a) Report numbers of individuals at each stage of study—eg numbers potentially eligible, examined for eligibility, confirmed eligible, included in the study, completing follow-up, and analysed | 8 | Based on the inclusion and exclusion criteria, 110 videos that were uploaded between 2009 and 2019 were evaluated. |
| | | (b) Give reasons for non-participation at each stage | NA | |
| | | (c) Consider use of a flow diagram | 6 | Figure 1 |
| Descriptive data | 14* | (a) Give characteristics of study participants (eg demographic, clinical, social) and information on exposures and potential confounders | 8 | The 110 videos had been watched a total of 8,633,569 times … *number of views/days since upload.* |
| | | (b) Indicate number of participants with missing data for each variable of interest | NA | |
| | | (c) *Cohort study*—Summarise follow-up time (eg, average and total amount) | NA | |
| Outcome data | 15* | *Cohort study*—Report numbers of outcome events or summary measures over time | | |
| | | *Case-control study*—Report numbers in each exposure category, or summary measures of exposure | | |
| | | *Cross-sectional study*—Report numbers of outcome events or summary measures | *9-10* | *The DISCERN health information scores … and understandability dimensions.* |

| | | | | |
|---|---|---|---|---|
| Main results | 16 | (*a*) Give unadjusted estimates and, if applicable, confounder-adjusted estimates and their precision (eg, 95% confidence interval). Make clear which confounders were adjusted for and why they were included | 9 | *Regarding the DISCERN scores for the 110 videos included in the analysis, there were insufficient results, with only 32 videos (29%) receiving 3 or more points out of 5.* *The overall score of the videos for DISCERN was only 27%.* *low scores regarding the actionability and understandability dimensions.* |
| | | (*b*) Report category boundaries when continuous variables were categorized | NA | |
| | | (*c*) If relevant, consider translating estimates of relative risk into absolute risk for a meaningful time period | NA | |
| Other analyses | 17 | Report other analyses done—eg analyses of subgroups and interactions, and sensitivity analyses | 10 | To evaluate the users' engagement with the provided videos regarding cancer treatment using herbs, a correlation test has been conducted. |
| Key results | 18 | Summarise key results with reference to study objectives | 11 | The reliability scores reveal that the source data is frequently absent, along with crucial financing and conflict of interest cues that can have an impact on how the general public interprets the provided health information. |
| Limitations | 19 | Discuss limitations of the study, taking into account sources of potential bias or imprecision. Discuss both direction and magnitude of any potential bias | 14 | *Limitations and future work* |
| Interpretation | 20 | Give a cautious overall interpretation of results considering objectives, limitations, multiplicity of analyses, results from similar studies, and other relevant evidence | 14 | *This study concludes that it is time for platform owners to introduce mechanisms for governing their e-platforms to reduce misleading and biased material and so enhance the quality of health-related content.* |

| | | | | |
|---|---|---|---|---|
| Generalisability | 21 | Discuss the generalisability (external validity) of the study results | 14 | *exploring the cultural settings of countries and nations regarding health information content on social media may add rich insights.* |
| **Other information** | | | | |
| Funding | 22 | Give the source of funding and the role of the funders for the present study and, if applicable, for the original study on which the present article is based | 15 | *The author(s) received no financial support for the research, authorship, and/or publication of this article.* |

Table S114. Quality Evaluation for Included Studies Using STROBE

38.STROBE Statement—checklist of items that should be included in reports of observational studies

| | Item No. | Recommendation | Page No. | Relevant text from manuscript |
|---|---|---|---|---|
| **Title and abstract** | 1 | (*a*) Indicate the study's design with a commonly used term in the title or the abstract | 1 | Video assessment |
| | | (*b*) Provide in the abstract an informative and balanced summary of what was done and what was found | 1 | "Using the search term "lung cancer," … including effective audio and visual channels. |
| **Introduction** | | | | |
| Background/rationale | 2 | Explain the scientific background and rationale for the investigation being reported | 2 | *over the use of YouTube due to the lack of content regulation and variable reliability [10–13]. Nonetheless, YouTube has tremendous potential in aiding patient education because of its accessibility, useability, and effectiveness as a learning tool* |
| Objectives | 3 | State specific objectives, including any prespecified hypotheses | 2 | assess the parameters and topics of the videos on YouTube for lung cancer information as reflected by an initial general search term and evaluate their characteristics against best learning practices. |
| **Methods** | | | | |
| Study design | 4 | Present key elements of study design early in the paper | 2 | Cross-sectional search |
| Setting | 5 | Describe the setting, locations, and relevant dates, including periods of recruitment, exposure, follow-up, and data collection | 2 | The term "lung cancer" was searched on YouTube on June 11, 2022 using Incognito mode (a clear cache and cookie browser) on Google Chrome. |
| Participants | 6 | (*a*) *Cohort study*—Give the eligibility criteria, and the sources and methods of selection of participants. Describe methods of follow-up | 2 | YouTube shorts, duplicates, non- English videos, paid videos, and videos not |

| | | | | |
|---|---|---|---|---|
| | | *Case-control study*—Give the eligibility criteria, and the sources and methods of case ascertainment and control selection. Give the rationale for the choice of cases and controls<br><br>*Cross-sectional study*—Give the eligibility criteria, and the sources and methods of selection of participants | | relevant to lung cancer were excluded. Only videos in English were included as the reviewers are monolingual. |
| | | (*b*) *Cohort study*—For matched studies, give matching criteria and number of exposed and unexposed<br><br>*Case-control study*—For matched studies, give matching criteria and the number of controls per case | NA | |
| Variables | 7 | Clearly define all outcomes, exposures, predictors, potential confounders, and effect modifiers. Give diagnostic criteria, if applicable | 2 | *(number of views, likes and dislikes, date of publication, and video length), video source parameters (country of origin, publisher affiliation, number of subscribers, and presenter type) and video content (subtitles, media type, target audience, lung cancer topic, number of comments, advertisements, and gross bias).* |
| Data sources/ measurement | 8* | For each variable of interest, give sources of data and details of methods of assessment (measurement). Describe comparability of assessment methods if there is more than one group | *2-3* | *Videos were from YouTube*<br><br>*Two researchers (PAI, a radiation oncologist with expertise in lung cancer and BC, who is a medical student research assistant with oncology research experience) reviewed a sample of 10 videos using the video assessment and Modified DISCERN Tool.* |
| Bias | 9 | Describe any efforts to address potential sources of bias | 2 | *Only the "Relevance" filter was used because it is the default filter which is most likely to be used by patients on their initial search.* |
| Study size | 10 | Explain how the study size was arrived at | 3 | 50 videos |

| | | | | |
|---|---|---|---|---|
| Quantitative variables | 11 | Explain how quantitative variables were handled in the analyses. If applicable, describe which groupings were chosen and why | 2 | *DISCERN* |
| Statistical methods | 12 | (*a*) Describe all statistical methods, including those used to control for confounding | 3 | *Descriptive statistics* |
| | | (*b*) Describe any methods used to examine subgroups and interactions | 4 | Video reliability |
| | | (*c*) Explain how missing data were addressed | NA | |
| | | (*d*) *Cohort study*—If applicable, explain how loss to follow-up was addressed<br>*Case-control study*—If applicable, explain how matching of cases and controls was addressed<br>*Cross-sectional study*—If applicable, describe analytical methods taking account of sampling strategy | NA | |
| | | (*e*) Describe any sensitivity analyses | NA | |
| **Results** | | | | |
| Participants | 13* | (a) Report numbers of individuals at each stage of study—eg numbers potentially eligible, examined for eligibility, confirmed eligible, included in the study, completing follow-up, and analysed | 3 | *A full list of the 50 videos and video characteristics can be viewed in Appendix 1.* |
| | | (b) Give reasons for non-participation at each stage | NA | |
| | | (c) Consider use of a flow diagram | NA | |
| Descriptive data | 14* | (a) Give characteristics of study participants (eg demographic, clinical, social) and information on exposures and potential confounders | 3 | The general parameters are summarized in Table 1 … *The presenter was unspecified in 14 videos.* |
| | | (b) Indicate number of participants with missing data for each variable of interest | NA | |
| | | (c) *Cohort study*—Summarise follow-up time (eg, average and total amount) | NA | |
| Outcome data | 15* | *Cohort study*—Report numbers of outcome events or summary measures over time | | |
| | | *Case-control study*—Report numbers in each exposure category, or summary measures of exposure | | |
| | | *Cross-sectional study*—Report numbers of outcome events or summary measures | *4* | *Most videos used only computer animations … (mean 1, SD 0) and commercial affiliations (mean 0.87, SD 0.35).* |

| | | | | |
|---|---|---|---|---|
| Main results | 16 | (*a*) Give unadjusted estimates and, if applicable, confounder-adjusted estimates and their precision (eg, 95% confidence interval). Make clear which confounders were adjusted for and why they were included | 4 | *Mean overall DISCERN score was 3.58 (SD 1.16), ranging from 2 to 5. The video presenters with the highest scores included physicians (mean 3.91, SD 1.20), PhDs (mean 4, SD 0) or unspecified presenters (mean 3.6, SD 1.18).* |
| | | (*b*) Report category boundaries when continuous variables were categorized | NA | |
| | | (*c*) If relevant, consider translating estimates of relative risk into absolute risk for a meaningful time period | NA | |
| Other analyses | 17 | Report other analyses done—eg analyses of subgroups and interactions, and sensitivity analyses | NA | |
| Key results | 18 | Summarise key results with reference to study objectives | 6 | Although videos with commercial affiliations may not necessarily have misleading information (as supported by high item 3 DISCERN scores), patients should be advised to be cautious when watching commercial videos and use other sources for supporting information. |
| Limitations | 19 | Discuss limitations of the study, taking into account sources of potential bias or imprecision. Discuss both direction and magnitude of any potential bias | 6 | *There are limitations to this study…* |
| Interpretation | 20 | Give a cautious overall interpretation of results considering objectives, limitations, multiplicity of analyses, results from similar studies, and other relevant evidence | 7 | *It highlights the need to encourage those producing health education videos to continue following best practices for video learning such as using optimal video length, effective visual aids, subtitles, and publishing up-to-date information to improve education.* |
| Generalisability | 21 | Discuss the generalisability (external validity) of the study results | 7 | *our search was only performed in English. Future research could investigate the impact of* |

| | | | | |
|---|---|---|---|---|
| | | | | *language on educational lung cancer videos.* |
| **Other information** | | | | |
| Funding | 22 | Give the source of funding and the role of the funders for the present study and, if applicable, for the original study on which the present article is based | 7 | *Funding This work was supported by the Faculty of Medicine Summer Student Research Program at the University of British Columbia.* |

Table S115. Quality Evaluation for Included Studies Using STROBE

39.STROBE Statement—checklist of items that should be included in reports of observational studies

| | Item No. | Recommendation | Page No. | Relevant text from manuscript |
|---|---|---|---|---|
| **Title and abstract** | 1 | (*a*) Indicate the study's design with a commonly used term in the title or the abstract | 1 | Cross-sectional web-based analysis |
| | | (*b*) Provide in the abstract an informative and balanced summary of what was done and what was found | 1 | Methods and Results |
| **Introduction** | | | | |
| Background/rationale | 2 | Explain the scientific background and rationale for the investigation being reported | 2 | *It is increasingly used to disseminate health-related information and has become an easily accessible source for patients to acquire information related to their diseases* |
| Objectives | 3 | State specific objectives, including any prespecified hypotheses | 2 | *Identify YouTube videos about BCC and to assess their quality, reliability, usability, and understandability.* |
| **Methods** | | | | |
| Study design | 4 | Present key elements of study design early in the paper | 2 | Cross-sectional search |
| Setting | 5 | Describe the setting, locations, and relevant dates, including periods of recruitment, exposure, follow-up, and data collection | 2 | A video search on YouTube was conducted in July 2020, using German BCC-related keywords (eg, "Basalzellkarzinom," "Basaliom," "weißer hautkrebs," and "heller hautkrebs"). |
| Participants | 6 | (*a*) *Cohort study*—Give the eligibility criteria, and the sources and methods of selection of participants. Describe methods of follow-up<br>*Case-control study*—Give the eligibility criteria, and the sources and methods of case ascertainment and control selection. Give the rationale for the choice of cases and controls | 2 | inclusion criteria: (1) contain information referring to BCC, (2) be accessible for free and for all users, and (3) provide information in the German language. Videos were excluded if they were commercials, they did not |

| | | | | |
|---|---|---|---|---|
| | | *Cross-sectional study*—Give the eligibility criteria, and the sources and methods of selection of participants | | have sound, they presented only photos, or if the duration was less than one minute. All search results were screened for duplicates, and the predefined eligibility criteria were applied. |
| | | (*b*) *Cohort study*—For matched studies, give matching criteria and number of exposed and unexposed<br>*Case-control study*—For matched studies, give matching criteria and the number of controls per case | NA | |
| Variables | 7 | Clearly define all outcomes, exposures, predictors, potential confounders, and effect modifiers. Give diagnostic criteria, if applicable | 2 | *The available baseline information (ie, URL, title, name of the provider, video length, and year of upload) of each selected video was documented. Additionally, the numbers of views, likes, and dislikes were extracted. With this information, we calculated the video power index (VPI) to assess the popularity of the videos.* |
| Data sources/ measurement | 8* | For each variable of interest, give sources of data and details of methods of assessment (measurement). Describe comparability of assessment methods if there is more than one group | *2* | *Videos were from YouTube*<br>*Two reviewers (TS and MH) independently assessed the videos' quality of information, reliability, and understandability.* |
| Bias | 9 | Describe any efforts to address potential sources of bias | 2 | *The first three pages (ie, 60 videos) were searched by two independent researchers for each keyword using Internet Explorer 11 (Microsoft).* |
| Study size | 10 | Explain how the study size was arrived at | 3 | 41 videos |

| | | | | |
|---|---|---|---|---|
| Quantitative variables | 11 | Explain how quantitative variables were handled in the analyses. If applicable, describe which groupings were chosen and why | 2 | *the numbers of views, likes, and dislikes were extracted. With this information, we calculated the video power index (VPI)*<br><br>*DISCERN, GQS, PEMAT-A/V* |
| Statistical methods | 12 | (*a*) Describe all statistical methods, including those used to control for confounding | 3 | *Descriptive analyses included mean (SD) or median (range). Subgroup differences were explored using the Kruskal-Wallis test. The relationship between the individual items of the tests was examined using Spearman correlation.* |
| | | (*b*) Describe any methods used to examine subgroups and interactions | 3 | *Subgroup differences were explored using the Kruskal-Wallis test.* |
| | | (*c*) Explain how missing data were addressed | NA | |
| | | (*d*) *Cohort study*—If applicable, explain how loss to follow-up was addressed<br><br>*Case-control study*—If applicable, explain how matching of cases and controls was addressed<br><br>*Cross-sectional study*—If applicable, describe analytical methods taking account of sampling strategy | NA | |
| | | (*e*) Describe any sensitivity analyses | NA | |
| **Results** | | | | |
| Participants | 13* | (a) Report numbers of individuals at each stage of study—eg numbers potentially eligible, examined for eligibility, confirmed eligible, included in the study, completing follow-up, and analysed | 3 | *Our search identified 659 videos. Following a multistep process, three review authors (TS, MH, and LR) screened the videos for duplicates and checked them for compliance with the predefined eligibility criteria. Finally, 41 individual videos were considered for assessment* |
| | | (b) Give reasons for non-participation at each stage | NA | |
| | | (c) Consider use of a flow diagram | 4 | Figure 1 |

| | | | | |
|---|---|---|---|---|
| Descriptive data | 14* | (a) Give characteristics of study participants (eg demographic, clinical, social) and information on exposures and potential confounders | 3-4 | *Most videos were provided by health… misleading information regarding the treatment of BCC.* |
| | | (b) Indicate number of participants with missing data for each variable of interest | NA | |
| | | (c) *Cohort study*—Summarise follow-up time (eg, average and total amount) | NA | |
| Outcome data | 15* | *Cohort study*—Report numbers of outcome events or summary measures over time | | |
| | | *Case-control study*—Report numbers in each exposure category, or summary measures of exposure | | |
| | | *Cross-sectional study*—Report numbers of outcome events or summary measures | *5* | *Quality: DISCERN and GQS Results*<br>*Understandability and Actionability: PEMAT-A/V*<br>*Results*<br>*Accuracy, Utility, and Reliability: JAMA Results* |
| Main results | 16 | (*a*) Give unadjusted estimates and, if applicable, confounder-adjusted estimates and their precision (eg, 95% confidence interval). Make clear which confounders were adjusted for and why they were included | 5 | *The mean DISCERN scores per video ranged from 1.31 to 4.38 points, with an average mean score of 3.31 (SD 0.80) points, indicating medium quality* |
| | | (*b*) Report category boundaries when continuous variables were categorized | NA | |
| | | (*c*) If relevant, consider translating estimates of relative risk into absolute risk for a meaningful time period | NA | |

| | | | | |
|---|---|---|---|---|
| Other analyses | 17 | Report other analyses done—eg analyses of subgroups and interactions, and sensitivity analyses | 6 | *A significant positive correlation was found between DISCERN and GQS values (r=0.836) as well as between DISCERN values and reliability and understandability criteria* |

| | | | | |
|---|---|---|---|---|
| Key results | 18 | Summarise key results with reference to study objectives | 8 | *currently available BCC videos were, overall, of medium to good quality and* |

| | | | | |
|---|---|---|---|---|
| | | | | *understandability but had low actionability and poor reliability.* |
| Limitations | 19 | Discuss limitations of the study, taking into account sources of potential bias or imprecision. Discuss both direction and magnitude of any potential bias | 8 | *YouTube search results are highly dynamic and will change when new videos are uploaded and when old videos are removed.*<br><br>*Additionally, we did not include videos with restricted access (eg, asking for log-in information).* |
| Interpretation | 20 | Give a cautious overall interpretation of results considering objectives, limitations, multiplicity of analyses, results from similar studies, and other relevant evidence | 8 | *As more and more patients use online material, including YouTube videos, for acquiring disease-specific knowledge, it is crucial to ensure good quality, understandability, and reliability prior to publication.* |
| Generalisability | 21 | Discuss the generalisability (external validity) of the study results | 8 | *did not include videos with restricted access (eg, asking for log-in information)* |
| **Other information** | | | | |
| Funding | 22 | Give the source of funding and the role of the funders for the present study and, if applicable, for the original study on which the present article is based | NA | |

Table S116. Quality Evaluation for Included Studies Using STROBE

40.STROBE Statement—checklist of items that should be included in reports of observational studies

| | Item No. | Recommendation | Page No. | Relevant text from manuscript |
|---|---|---|---|---|
| **Title and abstract** | 1 | (*a*) Indicate the study's design with a commonly used term in the title or the abstract | 1 | Cross-sectional search |
| | | (*b*) Provide in the abstract an informative and balanced summary of what was done and what was found | 1 | Methods and Results |
| **Introduction** | | | | |
| Background/rationale | 2 | Explain the scientific background and rationale for the investigation being reported | 2 | *YouTube videos have been previously evaluated for healthcare- related information of various diseases and procedures and were reported to be of variable educational quality* |
| Objectives | 3 | State specific objectives, including any prespecified hypotheses | 2 | *assess the educational quality of colonoscopy videos available through different sources on YouTube* |
| **Methods** | | | | |
| Study design | 4 | Present key elements of study design early in the paper | 2 | Cross-sectional search |
| Setting | 5 | Describe the setting, locations, and relevant dates, including periods of recruitment, exposure, follow-up, and data collection | 2 | *performed a YouTube search during September 2017, using the keyword 'colonoscopy' to identify all available videos on colonoscopy since the inception of the YouTube website* |
| Participants | 6 | (*a*) *Cohort study*—Give the eligibility criteria, and the sources and methods of selection of participants. Describe methods of follow-up<br>*Case-control study*—Give the eligibility criteria, and the sources and methods of case ascertainment and control selection. Give the rationale for the choice of cases and controls<br>*Cross-sectional study*—Give the eligibility criteria, and the sources and methods of selection of participants | 2 | *We included videos in English, which lasted ≤ 20 minutes and with good visual quality (defined as ≥ 240 pixels of progressive scan). We excluded videos in languages other than English, unrelated to* |

| | | | | |
|---|---|---|---|---|
| | | | | *colonoscopy, repeat videos, or with poor visual quality,* |
| | | (*b*) *Cohort study*—For matched studies, give matching criteria and number of exposed and unexposed<br><br>*Case-control study*—For matched studies, give matching criteria and the number of controls per case | NA | |
| Variables | 7 | Clearly define all outcomes, exposures, predictors, potential confounders, and effect modifiers. Give diagnostic criteria, if applicable | 2 | *Video characteristics included sex and race depicted, number of views, number of likes and dislikes, number of comments, and duration.* |
| Data sources/ measurement | 8* | For each variable of interest, give sources of data and details of methods of assessment (measurement). Describe comparability of assessment methods if there is more than one group | *2,4* | *Videos were from YouTube*<br><br>*Six blinded medical reviewers agreed to evaluate all the videos.* |
| Bias | 9 | Describe any efforts to address potential sources of bias | 4 | *Each reviewer was educated to score video accurately with standard ASGE video and was provided contact information of the senior author (K.D.) if any questions arose throughout the review process. All the reviewers scored each video independently and were blinded to each other's scoring and comments.* |
| Study size | 10 | Explain how the study size was arrived at | 5 | 255 videos |

| | | | | |
|---|---|---|---|---|
| Quantitative variables | 11 | Explain how quantitative variables were handled in the analyses. If applicable, describe which groupings were chosen and why | 2,4 | *number of views, number of likes and dislikes, number of comments, and duration.*<br><br>*rate the overall quality of health-care videos; global quality score (GQS)* |
| | 12 | (*a*) Describe all statistical methods, including those used to control for confounding | 4 | *Video characteristics were described using medians … with* |

| | | | | |
|---|---|---|---|---|
| Statistical methods | | | | *the Pearson correlation coefficient* |
| | | (*b*) Describe any methods used to examine subgroups and interactions | 4 | *One Way-Analysis of variance was used to compare mean scores (GQS and C-DQS) by the video source. Post-hoc analysis using fisher's test of least significant difference was performed to evaluate the exact difference between mean scores, and sources were grouped accordingly* |
| | | (*c*) Explain how missing data were addressed | NA | |
| | | (*d*) *Cohort study*—If applicable, explain how loss to follow-up was addressed<br>*Case-control study*—If applicable, explain how matching of cases and controls was addressed<br>*Cross-sectional study*—If applicable, describe analytical methods taking account of sampling strategy | NA | |
| | | (*e*) Describe any sensitivity analyses | NA | |
| **Results** | | | | |
| Participants | 13* | (a) Report numbers of individuals at each stage of study—eg numbers potentially eligible, examined for eligibility, confirmed eligible, included in the study, completing follow-up, and analysed | 4 | *Use of the keyword "colonoscopy" generated 429 videos, of which 174 were excluded based on our criteria. Two hundred fifty-five videos were included in the final analysis.* |
| | | (b) Give reasons for non-participation at each stage | NA | |
| | | (c) Consider use of a flow diagram | 2 | Fig. 1 |
| Descriptive data | 14* | (a) Give characteristics of study participants (eg demographic, clinical, social) and information on exposures and potential confounders | 5 | *"The most common video source was professional … and 1 dislikes, and had a median duration of 236 seconds."* |
| | | (b) Indicate number of participants with missing data for each variable of interest | NA | |
| | | (c) *Cohort study*—Summarise follow-up time (eg, average and total amount) | NA | |
| Outcome data | 15* | *Cohort study*—Report numbers of outcome events or summary measures over time | | |

| | | | | |
|---|---|---|---|---|
| | | *Case-control study*—Report numbers in each exposure category, or summary measures of exposure | | |
| | | *Cross-sectional study*—Report numbers of outcome events or summary measures | *5-6* | *“We also ranked the top 10 videos according to C-DQS score,.. representing moderate consistency.”* |
| Main results | 16 | (*a*) Give unadjusted estimates and, if applicable, confounder-adjusted estimates and their precision (eg, 95% confidence interval). Make clear which confounders were adjusted for and why they were included | 5 | *We also ranked the top 10 videos according to C-DQS score, most of them (75%) were uploaded by professional societies. These videos, along with their links and video source, can be found in Table 4. Overall mean scores for each video source was low,* |
| | | (*b*) Report category boundaries when continuous variables were categorized | NA | |
| | | (*c*) If relevant, consider translating estimates of relative risk into absolute risk for a meaningful time period | NA | |
| Other analyses | 17 | Report other analyses done—eg analyses of subgroups and interactions, and sensitivity analyses | 6 | *Correlation between mean C-DQS and GQS was performed using a scatter plot,* |
| Key results | 18 | Summarise key results with reference to study objectives | 7 | *our study also shows that YouTube is a poor and unreliable source of medical information* |
| Limitations | 19 | Discuss limitations of the study, taking into account sources of potential bias or imprecision. Discuss both direction and magnitude of any potential bias | 8 | *There were some limitations in our study…* |
| Interpretation | 20 | Give a cautious overall interpretation of results considering objectives, limitations, multiplicity of analyses, results from similar studies, and other relevant evidence | 8 | *The general population should rely on videos uploaded by professional societies and media if they chose to use YouTube to get more information on colonoscopy.* |
| Generalisability | 21 | Discuss the generalisability (external validity) of the study results | 8 | *Our study findings cannot be generalized to YouTube videos in a language other than* |

| | | | | |
|---|---|---|---|---|
| | | | | *English, longer than 20 minutes or that have poor image quality.* |
| **Other information** | | | | |
| Funding | 22 | Give the source of funding and the role of the funders for the present study and, if applicable, for the original study on which the present article is based | NA | |

Table S117. Quality Evaluation for Included Studies Using STROBE

41.STROBE Statement—checklist of items that should be included in reports of observational studies

| | Item No. | Recommendation | Page No. | Relevant text from manuscript |
|---|---|---|---|---|
| **Title and abstract** | 1 | (*a*) Indicate the study's design with a commonly used term in the title or the abstract | 1 | Content quality |
| | | (*b*) Provide in the abstract an informative and balanced summary of what was done and what was found | 1 | Methods and Results |
| **Introduction** | | | | |
| Background/rationale | 2 | Explain the scientific background and rationale for the investigation being reported | 2 | *factors pose a risk that young women with MBC will encounter health information that is incorrect, misleading, false, or removed from the appropriate context. Health information on social media is largely unregulated* |
| Objectives | 3 | State specific objectives, including any prespecified hypotheses | 2 | *assess the content quality of YouTube videos about and by young women with MBC and to identify common themes in MBC experiences based on video content.* |
| **Methods** | | | | |
| Study design | 4 | Present key elements of study design early in the paper | 2 | *systematic assessment* |
| Setting | 5 | Describe the setting, locations, and relevant dates, including periods of recruitment, exposure, follow-up, and data collection | 2 | *A systematic assessment of YouTube videos with the search term "metastatic breast cancer young" was conducted on August 3, 2021.* |
| Participants | 6 | (*a*) *Cohort study*—Give the eligibility criteria, and the sources and methods of selection of participants. Describe methods of follow-up<br>*Case-control study*—Give the eligibility criteria, and the sources and methods of case ascertainment and control selection. Give the rationale for the choice of cases and controls<br>*Cross-sectional study*—Give the eligibility criteria, and the sources and methods of selection of participants | 2 | *The search was performed in an incognito browser with no associated YouTube or Google account. Search results were placed in order from most to least views* |

| | | | | |
|---|---|---|---|---|
| | | (*b*) *Cohort study*—For matched studies, give matching criteria and number of exposed and unexposed<br>*Case-control study*—For matched studies, give matching criteria and the number of controls per case | | |
| Variables | 7 | Clearly define all outcomes, exposures, predictors, potential confounders, and effect modifiers. Give diagnostic criteria, if applicable | 2 | *The title, date uploaded, length, poster identity, number of likes, and number of comments were collected in a spreadsheet.*<br>*Several video characteristics were recorded. Videos included in playlists were recorded.* |
| Data sources/ measurement | 8* | For each variable of interest, give sources of data and details of methods of assessment (measurement). Describe comparability of assessment methods if there is more than one group | 2 | *Videos were from YouTube*<br>*A review of selected videos was performed by a communication studies researcher and two health care professionals.* |
| Bias | 9 | Describe any efforts to address potential sources of bias | 2 | *The search was performed in an incognito browser with no associated YouTube or Google account* |
| Study size | 10 | Explain how the study size was arrived at | 3 | 101 videos |

| | | | | |
|---|---|---|---|---|
| Quantitative variables | 11 | Explain how quantitative variables were handled in the analyses. If applicable, describe which groupings were chosen and why | 3 | *Patient Education Materials Assessment Tool (PEMAT) and DISCERN instruments* |
| Statistical methods | 12 | (*a*) Describe all statistical methods, including those used to control for confounding | 3 | Descriptive statistics only |
| | | (*b*) Describe any methods used to examine subgroups and interactions | NA | |
| | | (*c*) Explain how missing data were addressed | NA | |
| | | (*d*) *Cohort study*—If applicable, explain how loss to follow-up was addressed<br>*Case-control study*—If applicable, explain how matching of cases and controls was addressed | NA | |

| | | | | |
|---|---|---|---|---|
| | | *Cross-sectional study*—If applicable, describe analytical methods taking account of sampling strategy | | |
| | | (*e*) Describe any sensitivity analyses | NA | |
| **Results** | | | | |
| Participants | 13* | (a) Report numbers of individuals at each stage of study—eg numbers potentially eligible, examined for eligibility, confirmed eligible, included in the study, completing follow-up, and analysed | 3 | *In total, 101 videos were identified (Table 2). Of these, 61 (60.4%) videos were information-based and 59 (58.4%) were experience-based.* |
| | | (b) Give reasons for non-participation at each stage | NA | |
| | | (c) Consider use of a flow diagram | NA | |
| Descriptive data | 14* | (a) Give characteristics of study participants (eg demographic, clinical, social) and information on exposures and potential confounders | 3 | "*The average video length was 14.9 … videos consisted of news media.*" |
| | | (b) Indicate number of participants with missing data for each variable of interest | NA | |
| | | (c) *Cohort study*—Summarise follow-up time (eg, average and total amount) | NA | |
| Outcome data | 15* | *Cohort study*—Report numbers of outcome events or summary measures over time | *4* | *Assessment Using PEMAT and DISCERN …* |
| | | *Case-control study*—Report numbers in each exposure category, or summary measures of exposure | *NA* | |
| | | *Cross-sectional study*—Report numbers of outcome events or summary measures | *NA* | |
| Main results | 16 | (*a*) Give unadjusted estimates and, if applicable, confounder-adjusted estimates and their precision (eg, 95% confidence interval). Make clear which confounders were adjusted for and why they were included | 4 | *Overall, videos had moderate reliability and quality levels, and the mean DISCERN score was 2.44 (SD 0.7) out of 5.* |
| | | (*b*) Report category boundaries when continuous variables were categorized | NA | |
| | | (*c*) If relevant, consider translating estimates of relative risk into absolute risk for a meaningful time period | NA | |

| | | | | |
|---|---|---|---|---|
| Other analyses | 17 | Report other analyses done—eg analyses of subgroups and interactions, and sensitivity analyses | 4-5 | Comparison based on Themes, Narratives, and Sponsorships |

| | | | | |
|---|---|---|---|---|
| Key results | 18 | Summarise key results with reference to study objectives | 5 | *YouTube videos about MBC were very understandable but demonstrated low to moderate rates of actionability, with low reliability and quality scores.* |
| Limitations | 19 | Discuss limitations of the study, taking into account sources of potential bias or imprecision. Discuss both direction and magnitude of any potential bias | 6 | *Limitations of this study include restrictions based on language …* |
| Interpretation | 20 | Give a cautious overall interpretation of results considering objectives, limitations, multiplicity of analyses, results from similar studies, and other relevant evidence | 7 | *While web-based materials have limitations, including high rates of sponsorship bias and low levels of information quality, their potential to provide patient support is not fully developed.* |
| Generalisability | 21 | Discuss the generalisability (external validity) of the study results | 6 | *This study included only English-speaking videos, many of which originated in a US context, and therefore reflects specific social and geographical points of view.* |
| **Other information** | | | | |
| Funding | 22 | Give the source of funding and the role of the funders for the present study and, if applicable, for the original study on which the present article is based | 7 | *Acknowledgments*<br>*NM was supported by the Social Science and Humanities Research Council and* |

Table S118. Quality Evaluation for Included Studies Using STROBE

42.STROBE Statement—checklist of items that should be included in reports of observational studies

| | Item No. | Recommendation | Page No. | Relevant text from manuscript |
|---|---|---|---|---|
| **Title and abstract** | 1 | (*a*) Indicate the study's design with a commonly used term in the title or the abstract | 1 | Cross-sectional search |
| | | (*b*) Provide in the abstract an informative and balanced summary of what was done and what was found | 1 | We performed the largest, most comprehensive … with a total reach of >6 million viewers. |
| **Introduction** | | | | |
| Background/rationale | 2 | Explain the scientific background and rationale for the investigation being reported | 2 | *The extent to which the public currently receives biased, incorrect, or commercial information about PCa through videos and/or interactions on YouTube that could affect decision-making is unknown.* |
| Objectives | 3 | State specific objectives, including any prespecified hypotheses | 2 | perform a comprehensive study of YouTube videos on PCa that included validated instruments for content evaluation and comparisons between quality and user popularity and dissemination. |
| **Methods** | | | | |
| Study design | 4 | Present key elements of study design early in the paper | 2 | Cross-sectional review |
| Setting | 5 | Describe the setting, locations, and relevant dates, including periods of recruitment, exposure, follow-up, and data collection | 2 | reviewed the first 150 English-language YouTube videos on default searches for "prostate cancer screening" (75 of 173 000) and "prostate cancer treatment" (75 of 444 000) using the validated DISCERN quality criteria for consumer health information |

| | | | | |
|---|---|---|---|---|
| Participants | 6 | (*a*) *Cohort study*—Give the eligibility criteria, and the sources and methods of selection of participants. Describe methods of follow-up<br><br>*Case-control study*—Give the eligibility criteria, and the sources and methods of case ascertainment and control selection. Give the rationale for the choice of cases and controls<br><br>*Cross-sectional study*—Give the eligibility criteria, and the sources and methods of selection of participants | 2 | reviewed the first 150 English-language YouTube videos |
| | | (*b*) *Cohort study*—For matched studies, give matching criteria and number of exposed and unexposed<br><br>*Case-control study*—For matched studies, give matching criteria and the number of controls per case | NA | |
| Variables | 7 | Clearly define all outcomes, exposures, predictors, potential confounders, and effect modifiers. Give diagnostic criteria, if applicable | 2 | *the number of views per month (to account for different time intervals for which videos were online) and the ratio of the number of users who gave a video a thumbs up to the number of total viewers* |
| Data sources/ measurement | 8* | For each variable of interest, give sources of data and details of methods of assessment (measurement). Describe comparability of assessment methods if there is more than one group | *2* | *Videos were from YouTube* |
| Bias | 9 | Describe any efforts to address potential sources of bias | 2 | *None of these studies used a validated instrument for content evaluation or examined user interactions for the videos.* |
| Study size | 10 | Explain how the study size was arrived at | 2 | 150 videos |

| | | | | |
|---|---|---|---|---|
| Quantitative variables | 11 | Explain how quantitative variables were handled in the analyses. If applicable, describe which groupings were chosen and why | 2 | *the number of views per month (to account for different time intervals for which videos were online) and the ratio of the number of users who gave a video a thumbs up to the number of total viewers*<br><br>*DISCERN, PEMAT* |

| | | | | |
|---|---|---|---|---|
| Statistical methods | 12 | (*a*) Describe all statistical methods, including those used to control for confounding | 2 | *Pearson correlation coefficients between quality (DISCERN) and YouTube user popularity (views/month and thumbs up/views). In addition, we examined comments underneath each video to further characterize viewers' responses and the type of user-generated content being shared. Finally, we examined dissemination by calculating the number of views* |
| | | (*b*) Describe any methods used to examine subgroups and interactions | NA | |
| | | (*c*) Explain how missing data were addressed | NA | |
| | | (*d*) *Cohort study*—If applicable, explain how loss to follow-up was addressed<br>*Case-control study*—If applicable, explain how matching of cases and controls was addressed<br>*Cross-sectional study*—If applicable, describe analytical methods taking account of sampling strategy | NA | |
| | | (*e*) Describe any sensitivity analyses | NA | |
| **Results** | | | | |
| Participants | 13* | (a) Report numbers of individuals at each stage of study—eg numbers potentially eligible, examined for eligibility, confirmed eligible, included in the study, completing follow-up, and analysed | 3 | Table 1 shows the characteristics of the videos and user- generated comments (see Supplementary Table 1 for topics). |
| | | (b) Give reasons for non-participation at each stage | NA | |
| | | (c) Consider use of a flow diagram | NA | |
| Descriptive data | 14* | (a) Give characteristics of study participants (eg demographic, clinical, social) and information on exposures and potential confounders | 3 | Table 1 shows the characteristics of the videos and user- generated comments (see Supplementary Table 1 for topics). |
| | | (b) Indicate number of participants with missing data for each variable of interest | NA | |

| | | | | |
|---|---|---|---|---|
| | | (c) *Cohort study*—Summarise follow-up time (eg, average and total amount) | NA | |
| Outcome data | 15* | *Cohort study*—Report numbers of outcome events or summary measures over time | | |
| | | *Case-control study*—Report numbers in each exposure category, or summary measures of exposure | | |
| | | *Cross-sectional study*—Report numbers of outcome events or summary measures | *3* | *The median expert-rated quality of the videos with up to 1 348 172 … with a total reach of >6 million viewers.* |
| Main results | 16 | (*a*) Give unadjusted estimates and, if applicable, confounder-adjusted estimates and their precision (eg, 95% confidence interval). Make clear which confounders were adjusted for and why they were included | 3 | *the total reach for videos with poor quality or potentially misinformative or biased content in the video or comments.* |
| | | (*b*) Report category boundaries when continuous variables were categorized | NA | |
| | | (*c*) If relevant, consider translating estimates of relative risk into absolute risk for a meaningful time period | NA | |

| | | | | |
|---|---|---|---|---|
| Other analyses | 17 | Report other analyses done—eg analyses of subgroups and interactions, and sensitivity analyses | 3 | Statistical analysis revealed a significant negative correlation between scientific quality and viewer engagement |

| | | | | |
|---|---|---|---|---|
| Key results | 18 | Summarise key results with reference to study objectives | 3 | many popular videos about PCa on YouTube lack key elements of shared decision-making and contain biased content. |
| Limitations | 19 | Discuss limitations of the study, taking into account sources of potential bias or imprecision. Discuss both direction and magnitude of any potential bias | NA | |
| Interpretation | 20 | Give a cautious overall interpretation of results considering objectives, limitations, multiplicity of analyses, results from similar studies, and other relevant evidence | 3 | *The significant inverse relationship between expert ratings of information quality and the popularity of videos on YouTube is highly concerning because of the facilitation of wide dissemination of potentially misinformative content.* |
| Generalisability | 21 | Discuss the generalisability (external validity) of the study results | NA | |

| | | | | |
|---|---|---|---|---|
| **Other information** | | | | |
| Funding | 22 | Give the source of funding and the role of the funders for the present study and, if applicable, for the original study on which the present article is based | 3 | Funding/Support and role of the sponsor: Stacy Loeb is supported by a Tom Murphy Young Investigator Award from the Prostate Cancer Foundation |

Table S119. Quality Evaluation for Included Studies Using STROBE

43.STROBE Statement—checklist of items that should be included in reports of observational studies

| | Item No. | Recommendation | Page No. | Relevant text from manuscript |
|---|---|---|---|---|
| **Title and abstract** | 1 | (*a*) Indicate the study's design with a commonly used term in the title or the abstract | 1 | Evaluation of quality |
| | | (*b*) Provide in the abstract an informative and balanced summary of what was done and what was found | 1 | Methods and Results |
| **Introduction** | | | | |
| Background/rationale | 2 | Explain the scientific background and rationale for the investigation being reported | 2 | *Topics like "cancer" and "cancer and nutrition" are particularly popular in health-related YouTube videos. Moreover, videos can be uploaded to this platform by anyone and no reviewer approval is required. Patients may be misled by some adverts and posts meant for monetization in particular* |
| Objectives | 3 | State specific objectives, including any prespecified hypotheses | 2 | *assess the scientific reliability and quality of the most watched YouTube videos found using "cancer and nutrition" keywords, evaluated with internationally accepted scoring systems and to explore the relationships between them and video popularity.* |
| **Methods** | | | | |
| Study design | 4 | Present key elements of study design early in the paper | 2 | Cross-sectional search |
| Setting | 5 | Describe the setting, locations, and relevant dates, including periods of recruitment, exposure, follow-up, and data collection | 2 | *The videos were searched on the YouTube web page on March 1, 2022. The Google Trends application was used to determine the keywords to be searched. The terms "cancer and nutrition", "nutrition in cancer", "cancer food", "food* |

| | | | | |
|---|---|---|---|---|
| | | | | *for cancer", and "cancer diet" were chosen as keywords (* |
| Participants | 6 | (*a*) *Cohort study*—Give the eligibility criteria, and the sources and methods of selection of participants. Describe methods of follow-up<br><br>*Case-control study*—Give the eligibility criteria, and the sources and methods of case ascertainment and control selection. Give the rationale for the choice of cases and controls<br><br>*Cross-sectional study*—Give the eligibility criteria, and the sources and methods of selection of participants | 2 | *Videos whose language is not English, restricted videos, and videos shorter than 1 min were not included in the study. Recurrent versions of the same video were excluded to avoid duplication.* |
| | | (*b*) *Cohort study*—For matched studies, give matching criteria and number of exposed and unexposed<br><br>*Case-control study*—For matched studies, give matching criteria and the number of controls per case | NA | |
| Variables | 7 | Clearly define all outcomes, exposures, predictors, potential confounders, and effect modifiers. Give diagnostic criteria, if applicable | 2 | *video duration (seconds), video upload date, upload source, total number of views of the video, number of likes, number of dislikes, and number of comments. View rate (the number of*<br><br>*views divided by the number of days since the video was uploaded), the daily comment rate (the number of comments/d since uploaded), and the video-like rate—100 £ likes/(number of likes + dislikes)—were calculated. Additionally, the Video Power Index* |
| Data sources/ measurement | 8* | For each variable of interest, give sources of data and details of methods of assessment (measurement). Describe comparability of assessment methods if there is more than one group | *2* | *Videos were from YouTube*<br><br>*all preliminary searches were performed separately by two experienced oncologists.* |
| Bias | 9 | Describe any efforts to address potential sources of bias | 2 | *The URLs of all videos meeting the inclusion criteria were saved, and all preliminary searches were* |

| | | | | |
|---|---|---|---|---|
| | | | | *performed separately by two experienced oncologists.* |
| Study size | 10 | Explain how the study size was arrived at | 2 | 80 videos |
| Quantitative variables | 11 | Explain how quantitative variables were handled in the analyses. If applicable, describe which groupings were chosen and why | 2 | *The quality of the videos was evaluated with the DISCERN scoring system, modified DISCERN scoring system, Journal of the American Medical Association (JAMA) scoring system, and Global Quality Scale (GQS) total number of views of the video, number of likes, number of dislikes, and number of comments. View rate, the daily comment rate* |
| Statistical methods | 12 | (*a*) Describe all statistical methods, including those used to control for confounding | 2 | *Descriptive data were presented as numbers and minimum maximum (min-max) values. The distribution of data was evaluated with the Kolmogorov-Smirnov test. Comparison of the two groups for data that did not have normal distributions was made with the Mann-Whitney U test. The Kruskal-Wallis test was used to compare more than two groups. Spearman correlation analysis was performed for non-parametric variables.* |
| | | (*b*) Describe any methods used to examine subgroups and interactions | 2 | *Comparison of the two groups for data that did not have normal distributions was made with the Mann-Whitney U test. The Kruskal-Wallis test was used to compare more than two groups.* |
| | | (*c*) Explain how missing data were addressed | NA | |
| | | (*d*) *Cohort study*—If applicable, explain how loss to follow-up was addressed<br>*Case-control study*—If applicable, explain how matching of cases and controls was addressed | NA | |

| | | | | |
|---|---|---|---|---|
| | | *Cross-sectional study*—If applicable, describe analytical methods taking account of sampling strategy | | |
| | | (*e*) Describe any sensitivity analyses | NA | |
| **Results** | | | | |
| Participants | 13* | (a) Report numbers of individuals at each stage of study—eg numbers potentially eligible, examined for eligibility, confirmed eligible, included in the study, completing follow-up, and analysed | 2 | *A total of 80 videos were included in our study; 46 (58%) of the videos were uploaded to YouTube by health care providers (doctors or dietitians).* |
| | | (b) Give reasons for non-participation at each stage | NA | |
| | | (c) Consider use of a flow diagram | 3 | Figure 1 |
| Descriptive data | 14* | (a) Give characteristics of study participants (eg demographic, clinical, social) and information on exposures and potential confounders | 2 | *The median duration of the videos was 5.7 min (min max:1 122 min) … cruciferous vegetables (n = 16 [20%]) and berries (n = 12 [15%]).* |
| | | (b) Indicate number of participants with missing data for each variable of interest | NA | |
| | | (c) *Cohort study*—Summarise follow-up time (eg, average and total amount) | NA | |
| Outcome data | 15* | *Cohort study*—Report numbers of outcome events or summary measures over time | | |
| | | *Case-control study*—Report numbers in each exposure category, or summary measures of exposure | | |
| | | *Cross-sectional study*—Report numbers of outcome events or summary measures | *2-3* | *The median DISCERN score of all videos was … DISCERN score were found inversely proportional.* |
| Main results | 16 | (*a*) Give unadjusted estimates and, if applicable, confounder-adjusted estimates and their precision (eg, 95% confidence interval). Make clear which confounders were adjusted for and why they were included | 2 | *According to the DISCERN classification, 4% were "very poor," 50% were "poor," 35% were "moderate," 9% were "good," and only 2% were "excellent."* |
| | | (*b*) Report category boundaries when continuous variables were categorized | NA | |
| | | (*c*) If relevant, consider translating estimates of relative risk into absolute risk for a meaningful time period | NA | |

| | | | | |
|---|---|---|---|---|
| Other analyses | 17 | Report other analyses done—eg analyses of subgroups and interactions, and sensitivity analyses | 3 | Comparison between physician and independent uploader videos. |
| Key results | 18 | Summarise key results with reference to study objectives | 3 | *the amount of content that could benefit patients was quite low. We also found that video popularity increased as quality and reliability declined.* |
| Limitations | 19 | Discuss limitations of the study, taking into account sources of potential bias or imprecision. Discuss both direction and magnitude of any potential bias | 5 | *There are some limitations of our study…* |
| Interpretation | 20 | Give a cautious overall interpretation of results considering objectives, limitations, multiplicity of analyses, results from similar studies, and other relevant evidence | 5 | *There is a need to attach importance to the education given to patients by physicians and nutritionists specializing in cancer and nutrition and to produce quality content on YouTube or similar platforms.* |
| Generalisability | 21 | Discuss the generalisability (external validity) of the study results | 5 | *The fact that our study is restricted to English sources also can be a limitation. Finally, with the widespread use of the Internet and social media, applications such as Twitter and Instagram are also widely used, and these platforms are excluded from our study.* |
| **Other information** | | | | |
| Funding | 22 | Give the source of funding and the role of the funders for the present study and, if applicable, for the original study on which the present article is based | NA | |

Table S120. Quality Evaluation for Included Studies Using STROBE

44.STROBE Statement—checklist of items that should be included in reports of observational studies

| | Item No. | Recommendation | Page No. | Relevant text from manuscript |
|---|---|---|---|---|
| **Title and abstract** | 1 | (*a*) Indicate the study's design with a commonly used term in the title or the abstract | 1 | *Cross-sectional search query* |
| | | (*b*) Provide in the abstract an informative and balanced summary of what was done and what was found | 1 | *We designed a search query and inclusion/exclusion criteria … which could affect patients' perceptions of their disease or understanding of treatment options.* |
| **Introduction** | | | | |
| Background/rationale | 2 | Explain the scientific background and rationale for the investigation being reported | 1 | *These platforms have strong potential for use in patient education due to their ability to reach the masses and be viewed from a comfortable setting.* |
| Objectives | 3 | State specific objectives, including any prespecified hypotheses | 2 | *used an objective search strategy designed to reflect YouTube user behaviors to examine the quality and comprehensiveness of YouTube videos discussing pancreatic cancer* |
| **Methods** | | | | |
| Study design | 4 | Present key elements of study design early in the paper | 2 | Cross-sectional search |
| Setting | 5 | Describe the setting, locations, and relevant dates, including periods of recruitment, exposure, follow-up, and data collection | 2 | *On September 1, 2022, we conducted a search on YouTube for videos related to the diagnosis and treatment of pancreatic cancer* |
| Participants | 6 | (*a*) *Cohort study*—Give the eligibility criteria, and the sources and methods of selection of participants. Describe methods of follow-up<br>*Case-control study*—Give the eligibility criteria, and the sources and methods of case ascertainment and control selection. Give the rationale for the choice of cases and controls | 2 | *We excluded videos that were not in English, were longer than 25 min, were published as part of a series …* |

| | | | | |
|---|---|---|---|---|
| | | *Cross-sectional study*—Give the eligibility criteria, and the sources and methods of selection of participants | | |
| | | (*b*) *Cohort study*—For matched studies, give matching criteria and number of exposed and unexposed<br>*Case-control study*—For matched studies, give matching criteria and the number of controls per case | | |
| Variables | 7 | Clearly define all outcomes, exposures, predictors, potential confounders, and effect modifiers. Give diagnostic criteria, if applicable | 2 | *Number of views and likes for each video were extracted.*<br>*View and like count served as a measure … researcher not affiliated with a CC), or a non-expert (civilian or media).* |
| Data sources/ measurement | 8* | For each variable of interest, give sources of data and details of methods of assessment (measurement). Describe comparability of assessment methods if there is more than one group | 2 | *Videos were from YouTube*<br>*Two reviewers independently collected descriptive and quantitative data from each video that met inclusion criteria, with any disagreements resolved by a third reviewer.* |
| Bias | 9 | Describe any efforts to address potential sources of bias | 2 | *The search was conducted in an incognito Google Chrome browser to avoid the potential influence of prior browsing history on the results.* |
| Study size | 10 | Explain how the study size was arrived at | 3 | 39 videos |

| | | | | |
|---|---|---|---|---|
| Quantitative variables | 11 | Explain how quantitative variables were handled in the analyses. If applicable, describe which groupings were chosen and why | 2 | *DISCERN and JAMA*<br>*View and like count, video length* |
| Statistical methods | 12 | (*a*) Describe all statistical methods, including those used to control for confounding | 3 | *Chi-square analysis to compare categorical variables. Linear regression was used to assess for correlations between quantitative variables. ANOVA* |

| | | | | |
|---|---|---|---|---|
| | | | | *and independent samples t-test were used to compare means between groups.* |
| | | (*b*) Describe any methods used to examine subgroups and interactions | 3 | *ANOVA and independent samples t-test were used to compare means between groups.* |
| | | (*c*) Explain how missing data were addressed | NA | |
| | | (*d*) *Cohort study*—If applicable, explain how loss to follow-up was addressed<br>*Case-control study*—If applicable, explain how matching of cases and controls was addressed<br>*Cross-sectional study*—If applicable, describe analytical methods taking account of sampling strategy | NA | |
| | | (*e*) Describe any sensitivity analyses | NA | |
| **Results** | | | | |
| Participants | 13* | (a) Report numbers of individuals at each stage of study—eg numbers potentially eligible, examined for eligibility, confirmed eligible, included in the study, completing follow-up, and analysed | 3 | *After the initial search query, 120 YouTube videos were retrieved and 39 met inclusion criteria (Fig. 1).* |
| | | (b) Give reasons for non-participation at each stage | NA | |
| | | (c) Consider use of a flow diagram | 3 | Fig. 1 |
| Descriptive data | 14* | (a) Give characteristics of study participants (eg demographic, clinical, social) and information on exposures and potential confounders | 3 | *the videos were viewed a total of 6,175,376 times… Median views/day was 15.5 (0.03 – 7162.2), and median likes/day was 0.1 (0.0 – 27.4).* |
| | | (b) Indicate number of participants with missing data for each variable of interest | NA | |
| | | (c) *Cohort study*—Summarise follow-up time (eg, average and total amount) | NA | |
| Outcome data | 15* | *Cohort study*—Report numbers of outcome events or summary measures over time | | |
| | | *Case-control study*—Report numbers in each exposure category, or summary measures of exposure | | |
| | | *Cross-sectional study*—Report numbers of outcome events or summary measures | *3* | *Assessment of Quality and Comprehensiveness The median …* |

| | | | | |
|---|---|---|---|---|
| Main results | 16 | (*a*) Give unadjusted estimates and, if applicable, confounder-adjusted estimates and their precision (eg, 95% confidence interval). Make clear which confounders were adjusted for and why they were included | 3 | *Reviewer 1 indicated that 8 videos scored at least a 7 on the Sahin scale, indicating high quality, while reviewer 2 indicated that 11 videos were high quality.* |
| | | (*b*) Report category boundaries when continuous variables were categorized | NA | |
| | | (*c*) If relevant, consider translating estimates of relative risk into absolute risk for a meaningful time period | NA | |
| Other analyses | 17 | Report other analyses done—eg analyses of subgroups and interactions, and sensitivity analyses | 4 | *Correlational Analysis* |
| Key results | 18 | Summarise key results with reference to study objectives | 5 | *the quality of these videos was low to moderate. Of the 39 videos that met inclusion criteria, only 7 were considered high quality.* |
| Limitations | 19 | Discuss limitations of the study, taking into account sources of potential bias or imprecision. Discuss both direction and magnitude of any potential bias | 6 | *Our study has several limitations….* |
| Interpretation | 20 | Give a cautious overall interpretation of results considering objectives, limitations, multiplicity of analyses, results from similar studies, and other relevant evidence | 6 | *Physicians should consider recommending that patients opt to watch longer videos, as these were strongly correlated with higher quality information.* |
| Generalisability | 21 | Discuss the generalisability (external validity) of the study results | 6 | *our small sample size may not reflect the true status of quality in YouTube videos describing pancreatic cancer.* |
| **Other information** | | | | |
| Funding | 22 | Give the source of funding and the role of the funders for the present study and, if applicable, for the original study on which the present article is based | NA | |

Table S121. Quality Evaluation for Included Studies Using STROBE

45.STROBE Statement—checklist of items that should be included in reports of observational studies

| | Item No. | Recommendation | Page No. | Relevant text from manuscript |
|---|---|---|---|---|
| **Title and abstract** | 1 | (*a*) Indicate the study's design with a commonly used term in the title or the abstract | 1 | descriptive study |
| | | (*b*) Provide in the abstract an informative and balanced summary of what was done and what was found | 1 | Methods and Results |
| **Introduction** | | | | |
| Background/rationale | 2 | Explain the scientific background and rationale for the investigation being reported | 2 | YouTube does not provide a strict filtering facility, and anyone can effortlessly upload a video for free. Videos on YouTube vary greatly in reliability and quality. As a result, not only can these videos be deceiving or have a promotional agenda, but they can also potentially harm patient health. |
| Objectives | 3 | State specific objectives, including any prespecified hypotheses | 2 | assess the content, quality, and reliability of the most watched English-language YouTube videos about HCC |
| **Methods** | | | | |
| Study design | 4 | Present key elements of study design early in the paper | 2 | descriptive research |
| Setting | 5 | Describe the setting, locations, and relevant dates, including periods of recruitment, exposure, follow-up, and data collection | 2 | searched videos on YouTube (www.youtube.com) about HCC on March 15, 2022, using two different search terms |
| Participants | 6 | (*a*) *Cohort study*—Give the eligibility criteria, and the sources and methods of selection of participants. Describe methods of follow-up<br>*Case-control study*—Give the eligibility criteria, and the sources and methods of case ascertainment and control selection. Give the rationale for the choice of cases and controls | 2 | The top 100 videos were ranked and recorded for each search term. Internet research shows that users focus more on the first pages of the results and that 97.5% of Internet users look only at the first 10 pages.[9] We |

| | | | | |
|---|---|---|---|---|
| | | *Cross-sectional study*—Give the eligibility criteria, and the sources and methods of selection of participants | | included the 54 videos that were obtained in both searches once. Three of the remaining 146 videos were eliminated due to irrelevance, and we completed the study with the remaining 143 videos. |
| | | (*b*) *Cohort study*—For matched studies, give matching criteria and number of exposed and unexposed<br>*Case-control study*—For matched studies, give matching criteria and the number of controls per case | | |
| Variables | 7 | Clearly define all outcomes, exposures, predictors, potential confounders, and effect modifiers. Give diagnostic criteria, if applicable | 2 | Parameters such as the video length, likes, comments, number of views, and video upload date were recorded while we recorded the videos. We recorded the videos in a file, and two independent evaluators scored the information quality and reliability. |
| Data sources/ measurement | 8* | For each variable of interest, give sources of data and details of methods of assessment (measurement). Describe comparability of assessment methods if there is more than one group | *2* | *Videos were from YouTube* |
| Bias | 9 | Describe any efforts to address potential sources of bias | 2 | Cookies and the browsing history were cleared before each search so that they were not affected by previous search results |
| Study size | 10 | Explain how the study size was arrived at | 2 | 143 videos |

| | | | | |
|---|---|---|---|---|
| Quantitative variables | 11 | Explain how quantitative variables were handled in the analyses. If applicable, describe which groupings were chosen and why | 2 | video length, likes, comments, number of views, Global Quality Scale (GQS), modified DISCERN tool |
| Statistical methods | 12 | (*a*) Describe all statistical methods, including those used to control for confounding | 3 | Cohen's kappa coefficient was used for reliability. We performed a conformity-to- |

| | | | | |
|---|---|---|---|---|
| | | | | normal distribution test for the continuous variables with the Shapiro–Wilk test. Median (minimum–maximum) values were used for the non-normally distributed data. The Mann–Whitney *U* test was used to compare the mean values that did not show normal distribution for the two independent categories |
| | | (*b*) Describe any methods used to examine subgroups and interactions | 4 | Table 2, 3, 4 |
| | | (*c*) Explain how missing data were addressed | NA | |
| | | (*d*) *Cohort study*—If applicable, explain how loss to follow-up was addressed<br>*Case-control study*—If applicable, explain how matching of cases and controls was addressed<br>*Cross-sectional study*—If applicable, describe analytical methods taking account of sampling strategy | NA | |
| | | (*e*) Describe any sensitivity analyses | NA | |
| **Results** | | | | |
| Participants | 13* | (a) Report numbers of individuals at each stage of study—eg numbers potentially eligible, examined for eligibility, confirmed eligible, included in the study, completing follow-up, and analysed | 3 | In our study, the first 100 videos retrieved after searching for “hepatocellular cancer” and the first 100 videos retrieved after searching for “hepatocellular carcinoma” were examined. As 54 videos appeared in both searches, we excluded them from the study. In addition, we excluded 3 of the 146 videos in the study because they were irrelevant. We completed the study by examining 143 videos. |
| | | (b) Give reasons for non-participation at each stage | NA | |
| | | (c) Consider use of a flow diagram | 3 | Fig. 1 |

| | | | | |
|---|---|---|---|---|
| Descriptive data | 14* | (a) Give characteristics of study participants (eg demographic, clinical, social) and information on exposures and potential confounders | 4 | Most of the videos (56.25%; n = 81) related to treatment, followed by videos on the definition of hepatocellular cancer (32.64%; n = 47) and videos on risk factors (22.22%; n = 32). The lowest numbers were on surgical technique (6.25%; n = 9) and complications (10.42%; n = 15). The content distribution of the videos is shown in **Table 1**. |
| | | (b) Indicate number of participants with missing data for each variable of interest | NA | |
| | | (c) *Cohort study*—Summarise follow-up time (eg, average and total amount) | NA | |
| Outcome data | 15* | *Cohort study*—Report numbers of outcome events or summary measures over time | | |
| | | *Case-control study*—Report numbers in each exposure category, or summary measures of exposure | | |
| | | *Cross-sectional study*—Report numbers of outcome events or summary measures | *4* | "We evaluated 129 (89.58%) of the videos as useful and considered … We found a usefulness rate of 100% in the videos uploaded from the United Kingdom, Singapore, Canada, and Norway." |
| Main results | 16 | (*a*) Give unadjusted estimates and, if applicable, confounder-adjusted estimates and their precision (eg, 95% confidence interval). Make clear which confounders were adjusted for and why they were included | 4 | We found the GQS scores of the useful videos to be significantly higher than the misleading videos, with a median (min–max) score of 4 (2–5) ($P < 0.001$). When we compared the DISCERN scores, we found the scores of the useful videos to be significantly higher |
| | | (*b*) Report category boundaries when continuous variables were categorized | NA | |
| | | (*c*) If relevant, consider translating estimates of relative risk into absolute risk for a meaningful time period | NA | |

| | | | | |
|---|---|---|---|---|
| Other analyses | 17 | Report other analyses done—eg analyses of subgroups and interactions, and sensitivity analyses | NA | |
| Key results | 18 | Summarise key results with reference to study objectives | 5 | Studies show that 8 out of 10 Internet users use the Internet to search for health-related information.15 Patients who think that health professionals do not adequately inform them turn to online searches for additional information |
| Limitations | 19 | Discuss limitations of the study, taking into account sources of potential bias or imprecision. Discuss both direction and magnitude of any potential bias | 8 | The study's limitations include the potential for the videos to change widely due to the … |
| Interpretation | 20 | Give a cautious overall interpretation of results considering objectives, limitations, multiplicity of analyses, results from similar studies, and other relevant evidence | 8 | Although the rate of misleading videos in the current study was relatively low, Internet users should be careful when searching for information on YouTube. |
| Generalisability | 21 | Discuss the generalisability (external validity) of the study results | 8 | no opportunity to compare and discuss this with another study of the same content. |
| **Other information** | | | | |
| Funding | 22 | Give the source of funding and the role of the funders for the present study and, if applicable, for the original study on which the present article is based | NA | |

Table S122. Quality Evaluation for Included Studies Using STROBE

46.STROBE Statement—checklist of items that should be included in reports of observational studies

| | Item No. | Recommendation | Page No. | Relevant text from manuscript |
|---|---|---|---|---|
| **Title and abstract** | 1 | (*a*) Indicate the study's design with a commonly used term in the title or the abstract | 1 | Information-Quality Analysis |
| | | (*b*) Provide in the abstract an informative and balanced summary of what was done and what was found | 1 | "*Methods: YouTubeTM videos were searched using … we recorded the lowest median overall score for item 4 ("IMT in multimodality approach") and item 5 ("Future perspective").*" |
| **Introduction** | | | | |
| Background/rationale | 2 | Explain the scientific background and rationale for the investigation being reported | 2 | *The search for and spread of medical information on social media (SoMe) has grown rapidly [30]. Specifically, after COVID-19 outbreak, YouTube™ became one of the most widely used platforms to provide and obtain information [31]. More and more studies have revealed the low quality of medical content uploaded to YouTube* |
| Objectives | 3 | State specific objectives, including any prespecified hypotheses | 2 | *evaluate the quality of information on IMT in urological tumors, such as urothelial carcinoma (UC), RCC, and PCa, uploaded to YouTube™ during the last decades.* |
| **Methods** | | | | |
| Study design | 4 | Present key elements of study design early in the paper | 2 | Cross-sectional search |
| Setting | 5 | Describe the setting, locations, and relevant dates, including periods of recruitment, exposure, follow-up, and data collection | 2 | *On 25 March 2022, from 9:00 a.m. to 9:00 p.m. UTC-4, a YouTube™ systematic search was performed with 9 keyword combinations,* |

| | | | | |
|---|---|---|---|---|
| | | | | *examining the first 30 videos for each search* |
| Participants | 6 | (*a*) *Cohort study*—Give the eligibility criteria, and the sources and methods of selection of participants. Describe methods of follow-up<br>*Case-control study*—Give the eligibility criteria, and the sources and methods of case ascertainment and control selection. Give the rationale for the choice of cases and controls<br>*Cross-sectional study*—Give the eligibility criteria, and the sources and methods of selection of participants | 2 | *The following exclusion criteria were applied (Figure 1): (i) duplicate videos (n = 83), (ii) no information reported on immunotherapy for urological disease (n = 18); (iii) length > 50 min (n = 12); (iv) non-English language (n = 1).* |
| | | (*b*) *Cohort study*—For matched studies, give matching criteria and number of exposed and unexposed<br>*Case-control study*—For matched studies, give matching criteria and the number of controls per case | NA | |
| Variables | 7 | Clearly define all outcomes, exposures, predictors, potential confounders, and effect modifiers. Give diagnostic criteria, if applicable | 2 | *length (seconds), number of views, persistence on YouTube™ (days), number of thumbs-up, number of comments, number of channel subscribers, view ratio (defined as the ratio between number of views and persistence on YouTube™), whether comments were disabled, video author category (defined as a medical association [such as physicians' community channel, peer reviewed online journal, and cancer research associations]; medical center, hospital or university; or other [such as television channel and non profit foundations]), and video topic (defined as UC, RCC, general information on IMT, or PCa).* |
| Data sources/ measurement | 8* | For each variable of interest, give sources of data and details of methods of assessment (measurement). Describe comparability of assessment methods if there is more than one group | *2* | *Videos were from YouTube*<br>*The quality of videos was assessed by two* |

| | | | | |
|---|---|---|---|---|
| | | | | *investigators (a junior and a senior urology resident).* |
| Bias | 9 | Describe any efforts to address potential sources of bias | 2 | *third investigator (an Associate Professor) adjudicated any differences, and a consensus was achieved among all reviewers* |
| Study size | 10 | Explain how the study size was arrived at | 3 | 156 videos |

| | | | | |
|---|---|---|---|---|
| Quantitative variables | 11 | Explain how quantitative variables were handled in the analyses. If applicable, describe which groupings were chosen and why | 2 | *number of thumbs-up, number of comments, number of channel subscribers, view ratio PEMAT A/V, DISCERN questionnaire, 5-item Misinformation scale* |
| Statistical methods | 12 | (*a*) Describe all statistical methods, including those used to control for confounding | 4 | *Descriptive statistics are presented as medians and interquartile ranges (IQR) for continuously coded variables or counts and percentages for categorically coded variables. Kruskal–Wallis, Chi-square, and proportion tests examined the statistical significance in medians' and proportions' differences. Pearson's test was used to assess a potential correlation between the variables.* |
| | | (*b*) Describe any methods used to examine subgroups and interactions | 4 | *The overall collected videos were stratified into four groups, according to video topic (UC, RCC, PCa, or general information on IMT).* |
| | | (*c*) Explain how missing data were addressed | NA | |
| | | (*d*) *Cohort study*—If applicable, explain how loss to follow-up was addressed<br><br>*Case-control study*—If applicable, explain how matching of cases and controls was addressed | NA | |

| | | | | |
|---|---|---|---|---|
| | | *Cross-sectional study*—If applicable, describe analytical methods taking account of sampling strategy | | |
| | | (*e*) Describe any sensitivity analyses | NA | |
| **Results** | | | | |
| Participants | 13* | (a) Report numbers of individuals at each stage of study—eg numbers potentially eligible, examined for eligibility, confirmed eligible, included in the study, completing follow-up, and analysed | 5 | *Of all 270 videos examined, 156 were suitable for the analysis (Table 1).* |
| | | (b) Give reasons for non-participation at each stage | NA | |
| | | (c) Consider use of a flow diagram | 3 | Figure 1 |
| Descriptive data | 14* | (a) Give characteristics of study participants (eg demographic, clinical, social) and information on exposures and potential confounders | 5 | *The videos were stratified by topic: 67 (42.9%) were about UC, 38 (24.3%) about KC, 31 (19.8%) about general information on IMT, and 20 (12.8%) about PCa.* |
| | | (b) Indicate number of participants with missing data for each variable of interest | NA | |
| | | (c) *Cohort study*—Summarise follow-up time (eg, average and total amount) | NA | |
| Outcome data | 15* | *Cohort study*—Report numbers of outcome events or summary measures over time | *6* | *According to PEMAT A/V, the median Understandability score was 40% (IQR: 20–61.5) … No statistically significant results were achieved for the other correlations (all p > 0.05).* |
| | | *Case-control study*—Report numbers in each exposure category, or summary measures of exposure | | |
| | | *Cross-sectional study*—Report numbers of outcome events or summary measures | | |
| Main results | 16 | (*a*) Give unadjusted estimates and, if applicable, confounder-adjusted estimates and their precision (eg, 95% confidence interval). Make clear which confounders were adjusted for and why they were included | 6 | *According to PEMAT A/V, the median Understandability score was 40% (IQR: 20–61.5) and the median Actionability score was 0% (IQR: 0–0). According to DISCERN, the median overall score was 45 (IQR: 40—55.5).* |
| | | (*b*) Report category boundaries when continuous variables were categorized | NA | |
| | | (*c*) If relevant, consider translating estimates of relative risk into absolute risk for a meaningful time period | NA | |

| | | | | |
|---|---|---|---|---|
| Other analyses | 17 | Report other analyses done—eg analyses of subgroups and interactions, and sensitivity analyses | 6 | *A statistically significant positive correlation between view ratio and Understandability (r = 0.21, p = 0.006) and view ratio and Actionability (r = 0.21, p = 0.01) was recorded* |
| | | | | |
| Key results | 18 | Summarise key results with reference to study objectives | 7 | *regardless of the topic, YouTubeTM videos are not informative enough for applications by Internet users.* |
| Limitations | 19 | Discuss limitations of the study, taking into account sources of potential bias or imprecision. Discuss both direction and magnitude of any potential bias | 8 | *Our study is not devoid of limitations….* |
| Interpretation | 20 | Give a cautious overall interpretation of results considering objectives, limitations, multiplicity of analyses, results from similar studies, and other relevant evidence | 8 | *Official medical institutions should improve their multimedia content by producing easily understandable materials for Internet users and sharing evidence-based content.* |
| Generalisability | 21 | Discuss the generalisability (external validity) of the study results | 8 | *quality assessment of videos were subjective. To reduce this confounder, three investigators were involved to independently analyze video content. However, a multidisciplinary objective analysis with new quality-assessment tools is required in the future* |
| **Other information** | | | | |
| Funding | 22 | Give the source of funding and the role of the funders for the present study and, if applicable, for the original study on which the present article is based | 8 | *Funding: This research received no external funding.* |

Table S123. Quality Evaluation for Included Studies Using STROBE

47.STROBE Statement—checklist of items that should be included in reports of observational studies

| | Item No. | Recommendation | Page No. | Relevant text from manuscript |
|---|---|---|---|---|
| **Title and abstract** | 1 | (*a*) Indicate the study's design with a commonly used term in the title or the abstract | 1 | Cross-sectional search |
| | | (*b*) Provide in the abstract an informative and balanced summary of what was done and what was found | 1 | Methods and Results |
| **Introduction** | | | | |
| Background/rationale | 2 | Explain the scientific background and rationale for the investigation being reported | 2 | *As health information seeking on the Internet has become more popular, the number of patients using the online platforms as a source of medical information about diseases and treatment methods has increased accordingly.* |
| Objectives | 3 | State specific objectives, including any prespecified hypotheses | 2 | *evaluate the quality and reliability of the YouTube videos on CR as a source of information for cancer survivors.* |
| **Methods** | | | | |
| Study design | 4 | Present key elements of study design early in the paper | 2 | Cross-sectional search |
| Setting | 5 | Describe the setting, locations, and relevant dates, including periods of recruitment, exposure, follow-up, and data collection | 2 | *A video-based search on YouTube online hosting platform was performed on February 26th, 2021, by using the English keywords: "cancer rehabilitation" and "oncology rehabilitation."* |
| Participants | 6 | (*a*) *Cohort study*—Give the eligibility criteria, and the sources and methods of selection of participants. Describe methods of follow-up<br>*Case-control study*—Give the eligibility criteria, and the sources and methods of case ascertainment and control selection. Give the rationale for the choice of cases and controls<br>*Cross-sectional study*—Give the eligibility criteria, and the sources and methods of selection of participants | 2 | *Videos not related to cancer rehabilitation, patient testimonials, and in languages other than English were excluded in the evaluation. The videos with no sound were also removed from the sample.* |

| | | | | |
|---|---|---|---|---|
| | | (*b*) *Cohort study*—For matched studies, give matching criteria and number of exposed and unexposed<br><br>*Case-control study*—For matched studies, give matching criteria and the number of controls per case | NA | |
| Variables | 7 | Clearly define all outcomes, exposures, predictors, potential confounders, and effect modifiers. Give diagnostic criteria, if applicable | 2 | *the time to upload of the video, the video duration (second), the uploader, the number of views, likes, dislikes, and the interaction index—([number of likes-number of dislikes] / total number of views Å~ 100%). Profiles of the uploaders were recorded and classified under five categories: physician, physiotherapist, health-related website, academic institution/university, and news agency.* |
| Data sources/ measurement | 8* | For each variable of interest, give sources of data and details of methods of assessment (measurement). Describe comparability of assessment methods if there is more than one group | 2 | *Data were from YouTube*<br><br>*After evaluating the videos for eligibility, the sampled videos were shared with two Physical Medicine and Rehabilitation (PM&R) specialist researchers so that they rate the videos.* |
| Bias | 9 | Describe any efforts to address potential sources of bias | 2 | *Whenever there was a difference between ratings of the two researchers, a third independent researcher also evaluated the video.* |
| Study size | 10 | Explain how the study size was arrived at | 3 | *53 videos* |

| | | | | |
|---|---|---|---|---|
| Quantitative variables | 11 | Explain how quantitative variables were handled in the analyses. If applicable, describe which groupings were chosen and why | 2 | *the number of views, likes, dislikes, and the interaction index modified DISCERN, the Journal of the American Medical Association (JAMA), and Global* |

| | | | | |
|---|---|---|---|---|
| | | | | *Quality Scale (GQS) ranking systems were used.* |
| Statistical methods | 12 | (*a*) Describe all statistical methods, including those used to control for confounding | 3 | *"The Shapiro–Wilk test was used to assess the normality of the distribution. According to the results of normality analyses, the data … performed to analyze the association of the quantitative data."* |
| | | (*b*) Describe any methods used to examine subgroups and interactions | 6 | Table 2, 3 |
| | | (*c*) Explain how missing data were addressed | NA | |
| | | (*d*) *Cohort study*—If applicable, explain how loss to follow-up was addressed<br>*Case-control study*—If applicable, explain how matching of cases and controls was addressed<br>*Cross-sectional study*—If applicable, describe analytical methods taking account of sampling strategy | NA | |
| | | (*e*) Describe any sensitivity analyses | NA | |
| **Results** | | | | |
| Participants | 13* | (a) Report numbers of individuals at each stage of study—eg numbers potentially eligible, examined for eligibility, confirmed eligible, included in the study, completing follow-up, and analysed | 3 | *After excluding 147 videos (60 irrelevant, 87 repetitive), 53 videos were sampled for evaluation* |
| | | (b) Give reasons for non-participation at each stage | NA | |
| | | (c) Consider use of a flow diagram | 3 | Figure 1 |
| Descriptive data | 14* | (a) Give characteristics of study participants (eg demographic, clinical, social) and information on exposures and potential confounders | 3 | *The three most common uploader profiles were academic institute/university hospital, health-related website, and physiotherapist. The most of contents of the videos consisted of general description of CR, physical therapy and occupational therapy, and patient experiences.* |
| | | (b) Indicate number of participants with missing data for each variable of interest | NA | |
| | | (c) *Cohort study*—Summarise follow-up time (eg, average and total amount) | NA | |

| | | | | |
|---|---|---|---|---|
| Outcome data | 15* | *Cohort study*—Report numbers of outcome events or summary measures over time | | |
| | | *Case-control study*—Report numbers in each exposure category, or summary measures of exposure | | |
| | | *Cross-sectional study*—Report numbers of outcome events or summary measures | *3* | *"The mean modified DISCERN score was … quality and reliability tools were positively correlated among themselves"* |
| Main results | 16 | (*a*) Give unadjusted estimates and, if applicable, confounder-adjusted estimates and their precision (eg, 95% confidence interval). Make clear which confounders were adjusted for and why they were included | 3 | *According to the GQS scoring system, 51.8% of the videos were of low quality, 28.6% were of medium quality, and 14.4% were of high quality.* |
| | | (*b*) Report category boundaries when continuous variables were categorized | NA | |
| | | (*c*) If relevant, consider translating estimates of relative risk into absolute risk for a meaningful time period | NA | |
| Other analyses | 17 | Report other analyses done—eg analyses of subgroups and interactions, and sensitivity analyses | 3 | *According to the quality classification, the most frequent video uploader in all three groups was academic institute/university hospital.* |
| Key results | 18 | Summarise key results with reference to study objectives | 5 | *although academic institutions and university hospitals uploaded more videos compared to other uploaders, most of the videos in our sample were of low-quality according to the GSQ. They were also found to be similarly of poor quality in the other two assessment scales.* |
| Limitations | 19 | Discuss limitations of the study, taking into account sources of potential bias or imprecision. Discuss both direction and magnitude of any potential bias | 6 | *This study has several limitations…* |
| Interpretation | 20 | Give a cautious overall interpretation of results considering objectives, limitations, multiplicity of analyses, results from similar studies, and other relevant evidence | 6 | *Provided that the videos are chosen carefully and their content is used selectively, YouTube can be considered as a potential source of useful* |

| | | | | |
|---|---|---|---|---|
| | | | | *information for cancer survivors and caregivers who search Internet to have a better idea about cancer rehabilitation.* |
| Generalisability | 21 | Discuss the generalisability (external validity) of the study results | 6 | *the limited generalizability of the results due to sampling of only the videos in English* |
| **Other information** | | | | |
| Funding | 22 | Give the source of funding and the role of the funders for the present study and, if applicable, for the original study on which the present article is based | NA | |

Table S124. Quality Evaluation for Included Studies Using STROBE

48.STROBE Statement—checklist of items that should be included in reports of observational studies

| | Item No. | Recommendation | Page No. | Relevant text from manuscript |
|---|---|---|---|---|
| **Title and abstract** | 1 | (*a*) Indicate the study's design with a commonly used term in the title or the abstract | 1 | Content review |
| | | (*b*) Provide in the abstract an informative and balanced summary of what was done and what was found | 1 | *"We searched on YouTube™ for terms related to mental health … videos were rated as "generally poor" (21, 31.3%) or "poor" (12, 17.9%)."* |
| **Introduction** | | | | |
| Background/rationale | 2 | Explain the scientific background and rationale for the investigation being reported | 2 | *The recent literature highlights that YouTube™ has great potential for outreach since it is a platform that is often pre-installed on a massive range of smartphones on the market, freely available, and captivating [24,25].*<br>*This type of media channel can also disseminate biased, scarce, or low-quality information about mental health with respect to PCa patients [26], thus hindering the prevention and recognition of psychological distress.* |
| Objectives | 3 | State specific objectives, including any prespecified hypotheses | 2 | *assess the quality of the information available on YouTube™ about the prevalence, symptomatology, and potential treatments for mental health available in this specific population and whether this communication channel can be used as a reliable source.* |
| **Methods** | | | | |

| | | | | |
|---|---|---|---|---|
| Study design | 4 | Present key elements of study design early in the paper | 2 | Cross-sectional systematic search |
| Setting | 5 | Describe the setting, locations, and relevant dates, including periods of recruitment, exposure, follow-up, and data collection | 2 | *performed a systematic research on YouTube.com on 18 May 2022 at 9.50 a.m* |
| Participants | 6 | (*a*) *Cohort study*—Give the eligibility criteria, and the sources and methods of selection of participants. Describe methods of follow-up<br><br>*Case-control study*—Give the eligibility criteria, and the sources and methods of case ascertainment and control selection. Give the rationale for the choice of cases and controls<br><br>*Cross-sectional study*—Give the eligibility criteria, and the sources and methods of selection of participants | 2 | *A total of 1081 videos were collected and screened in the next few days through the following inclusion criteria: (1) videos had to reference mental health and (2) be in English.*<br><br>*After the exclusion of duplicate videos (n = 553, 51.16%), 528 videos were assessed for eligibility. The following exclusion criteria were applied: off-topic videos (n = 448, 97.18%), non-English language videos (n = 8, 1.74%), and video not available (n = 5, 1.08%). A total of 67 (12.7%) videos were eligible for the analyses* |
| | | (*b*) *Cohort study*—For matched studies, give matching criteria and number of exposed and unexposed<br><br>*Case-control study*—For matched studies, give matching criteria and the number of controls per case | NA | |
| Variables | 7 | Clearly define all outcomes, exposures, predictors, potential confounders, and effect modifiers. Give diagnostic criteria, if applicable | 2 | *the video length (in seconds), views, persistence time on YouTubeTM (in days), likes, comments, subscribers, view ratio (defined as the ratio between the number of views and the persistence time on YouTubeTM), and number of videos with disabled comments. Furthermore, we collected additional information on (a)target audience; (b) authoring institution; (c) topic; and (d)* |

| | | | | |
|---|---|---|---|---|
| | | | | *year of upload (<2014 vs. 2015–2017 vs. 2018–2019 vs. 2020–2022).*<br><br>*PEMAT A/V; DISCERN, GQS* |
| Data sources/ measurement | 8* | For each variable of interest, give sources of data and details of methods of assessment (measurement). Describe comparability of assessment methods if there is more than one group | *3* | *Videos were from YouTube Video content was independently assessed by a psychiatrist (LG) and two psychologists in training* |
| Bias | 9 | Describe any efforts to address potential sources of bias | 3 | *Two additional researchers, a licensed psychologist … a consensus was reached among all reviewers* |
| Study size | 10 | Explain how the study size was arrived at | 3 | 67 videos |

| | | | | |
|---|---|---|---|---|
| Quantitative variables | 11 | Explain how quantitative variables were handled in the analyses. If applicable, describe which groupings were chosen and why | 3 | *likes, comments, subscribers, view ratio (defined as the ratio between the number of views and the persistence time on YouTubeTM), and number of videos with disabled comments. PEMAT A/V; DISCERN, GQS* |
| Statistical methods | 12 | (*a*) Describe all statistical methods, including those used to control for confounding | 4 | *Descriptive statistics were presented as medians and interquartile ranges (IQR) for continuously coded variables or counts and percentages for categorically coded variables.*<br><br>*Chi-square distribution was used to test the statistical significance of proportions' differences. The ANOVA and the Kruskal–Wallis tests were used to examine the statistical significance of medians', means', and distributions' differences* |
| | | (*b*) Describe any methods used to examine subgroups and interactions | 4 | *The ANOVA and the Kruskal–Wallis tests were used to* |

| | | | | |
|---|---|---|---|---|
| | | | | *examine the statistical significance of medians', means', and distributions' differences* |
| | | (*c*) Explain how missing data were addressed | NA | |
| | | (*d*) *Cohort study*—If applicable, explain how loss to follow-up was addressed<br>*Case-control study*—If applicable, explain how matching of cases and controls was addressed<br>*Cross-sectional study*—If applicable, describe analytical methods taking account of sampling strategy | NA | |
| | | (*e*) Describe any sensitivity analyses | NA | |
| **Results** | | | | |
| Participants | 13* | (a) Report numbers of individuals at each stage of study—eg numbers potentially eligible, examined for eligibility, confirmed eligible, included in the study, completing follow-up, and analysed | 4 | *Of all the 1081 videos collected, 67 were deemed suitable for the analyses* |
| | | (b) Give reasons for non-participation at each stage | NA | |
| | | (c) Consider use of a flow diagram | 3 | Figure 1 |
| Descriptive data | 14* | (a) Give characteristics of study participants (eg demographic, clinical, social) and information on exposures and potential confounders | 4 | "*The mean length and views recorded were 1358.9 (Standard deviation [SD]: 201.0) and 42 … PEs and PCa diagnosis, and 11 (16.4%) to tools for managing PCa PEs.*" |
| | | (b) Indicate number of participants with missing data for each variable of interest | NA | |
| | | (c) *Cohort study*—Summarise follow-up time (eg, average and total amount) | NA | |
| Outcome data | 15* | *Cohort study*—Report numbers of outcome events or summary measures over time | | |
| | | *Case-control study*—Report numbers in each exposure category, or summary measures of exposure | | |
| | | *Cross-sectional study*—Report numbers of outcome events or summary measures | *4-6* | *Video Quality Assessment* |
| Main results | 16 | (*a*) Give unadjusted estimates and, if applicable, confounder-adjusted estimates and their precision (eg, 95% confidence interval). Make clear which confounders were adjusted for and why they were included | 6 | *21 (31.3%) of the eligible YouTube™ videos showed a "generally poor" quality, 12 (17.9%) were "poor",* |

| | | | | |
|---|---|---|---|---|
| | | (*b*) Report category boundaries when continuous variables were categorized | NA | |
| | | (*c*) If relevant, consider translating estimates of relative risk into absolute risk for a meaningful time period | NA | |
| Other analyses | 17 | Report other analyses done—eg analyses of subgroups and interactions, and sensitivity analyses | 7 | *Video Quality Assessment over Time* |
| Key results | 18 | Summarise key results with reference to study objectives | 8 | *Of the 67 eligible videos, most are aimed at PCa patients, but the quality of their educational information is low* |
| Limitations | 19 | Discuss limitations of the study, taking into account sources of potential bias or imprecision. Discuss both direction and magnitude of any potential bias | 9 | *Notwithstanding the fact that our methodology followed standardized research criteria shared in the scientific literature on content analysis of YouTubeTM videos [37–39], the current study has some limitations.* |
| Interpretation | 20 | Give a cautious overall interpretation of results considering objectives, limitations, multiplicity of analyses, results from similar studies, and other relevant evidence | 9 | *Multidisciplinary agreement is needed to define high-quality standards and improve communication in order to provide essential information for mental health care awareness after a PCa diagnosis and, therefore, support a key factor of adherence to medical treatments and the maintenance of a good quality of life.* |
| Generalisability | 21 | Discuss the generalisability (external validity) of the study results | 9 | *YouTube™ search results are influenced by Google's own search algorithms,*<br><br>*Second, only English-language videos were included in this study, but content in other languages could provide different information and highlight cultural imbalances.* |
| **Other information** | | | | |

| | | | | |
|---|---|---|---|---|
| Funding | 22 | Give the source of funding and the role of the funders for the present study and, if applicable, for the original study on which the present article is based | 10 | *Funding: This research received no external funding.* |

Table S125. Quality Evaluation for Included Studies Using STROBE

49.STROBE Statement—checklist of items that should be included in reports of observational studies

| | Item No. | Recommendation | Page No. | Relevant text from manuscript |
|---|---|---|---|---|
| **Title and abstract** | 1 | (*a*) Indicate the study's design with a commonly used term in the title or the abstract | 1 | Cross-sectional search |
| | | (*b*) Provide in the abstract an informative and balanced summary of what was done and what was found | 1 | Methods and Results |
| **Introduction** | | | | |
| Background/rationale | 2 | Explain the scientific background and rationale for the investigation being reported | 2 | *While English is the commonly used language for scientific research and the communication of research findings, less than a quarter of the world population speaks English [9]. Thereby, omitting a large number of videos available may lead to a potential distraught representation of the information available.* |
| Objectives | 3 | State specific objectives, including any prespecified hypotheses | 2 | perform a systemic and comparative assessment of the available urological videos on YouTube in 4 of the most commonly spoken languages in Europe. |
| **Methods** | | | | |
| Study design | 4 | Present key elements of study design early in the paper | 2 | Cross-sectional search |
| Setting | 5 | Describe the setting, locations, and relevant dates, including periods of recruitment, exposure, follow-up, and data collection | 2 | Independent search for videos concerning benign prostatic hyperplasia (BPH), prostate cancer (PCa), and urinary stone disease (USD) on YouTube was performed in October 2020 in English, French, German, and Italian by all participant authors. |
| Participants | 6 | (*a*) *Cohort study*—Give the eligibility criteria, and the sources and methods of selection of participants. Describe methods of follow-up | 2 | |

| | | | | |
|---|---|---|---|---|
| | | *Case-control study*—Give the eligibility criteria, and the sources and methods of case ascertainment and control selection. Give the rationale for the choice of cases and controls<br>*Cross-sectional study*—Give the eligibility criteria, and the sources and methods of selection of participants | | |
| | | (*b*) *Cohort study*—For matched studies, give matching criteria and number of exposed and unexposed<br>*Case-control study*—For matched studies, give matching criteria and the number of controls per case | NA | |
| Variables | 7 | Clearly define all outcomes, exposures, predictors, potential confounders, and effect modifiers. Give diagnostic criteria, if applicable | 2 | *Parameters assessed included basic data … compared to currently available evidence* |
| Data sources/ measurement | 8* | For each variable of interest, give sources of data and details of methods of assessment (measurement). Describe comparability of assessment methods if there is more than one group | 2 | *Videos were from YouTube*<br>*Four of the authors performing the analysis were board-certified urologists (Swiss Society of Urology) and 2 were senior residents.* |
| Bias | 9 | Describe any efforts to address potential sources of bias | 2 | *In cases of differing judgment between 2 authors, arbitration was performed by a third author (M.P. and P.B.)* |
| Study size | 10 | Explain how the study size was arrived at | 3 | 240 videos |

| | | | | |
|---|---|---|---|---|
| Quantitative variables | 11 | Explain how quantitative variables were handled in the analyses. If applicable, describe which groupings were chosen and why | 2 | *length, number of views, DISCERN* |
| Statistical methods | 12 | (*a*) Describe all statistical methods, including those used to control for confounding | 3 | *Descriptive analysis using medians and ranges were calculated for all results. Box plots were used to illustrate the DISCERN total scores.* |
| | | (*b*) Describe any methods used to examine subgroups and interactions | 4, 5 | Table 2, 3 |
| | | (*c*) Explain how missing data were addressed | NA | |
| | | (*d*) *Cohort study*—If applicable, explain how loss to follow-up was addressed | NA | |

| | | | | |
|---|---|---|---|---|
| | | *Case-control study*—If applicable, explain how matching of cases and controls was addressed<br>*Cross-sectional study*—If applicable, describe analytical methods taking account of sampling strategy | | |
| | | (*e*) Describe any sensitivity analyses | NA | |
| **Results** | | | | |
| Participants | 13* | (a) Report numbers of individuals at each stage of study—eg numbers potentially eligible, examined for eligibility, confirmed eligible, included in the study, completing follow-up, and analysed | 3 | A total of 240 videos were included to this analysis, all of which were uploaded between January 2008 and June 2020. Amongst the languages assessed, videos in English had the most views (median views 271,878 [65,313–2,513,007]); |
| | | (b) Give reasons for non-participation at each stage | NA | |
| | | (c) Consider use of a flow diagram | NA | |
| Descriptive data | 14* | (a) Give characteristics of study participants (eg demographic, clinical, social) and information on exposures and potential confounders | 3-4 | The video with the single most views concerned … declaration of COI or were deemed to provide commercial bias (Fig. 1). |
| | | (b) Indicate number of participants with missing data for each variable of interest | | |
| | | (c) *Cohort study*—Summarise follow-up time (eg, average and total amount) | | |
| Outcome data | 15* | *Cohort study*—Report numbers of outcome events or summary measures over time | | |
| | | *Case-control study*—Report numbers in each exposure category, or summary measures of exposure | | |
| | | *Cross-sectional study*—Report numbers of outcome events or summary measures | *4* | *Using the DISCERN questionnaire, the median overall quality for … PCa resulted in the lowest scores (36.5 points/36.75 points) (Fig. 3).* |
| Main results | 16 | (*a*) Give unadjusted estimates and, if applicable, confounder-adjusted estimates and their precision (eg, 95% confidence interval). Make clear which confounders were adjusted for and why they were included | 4 | *Using the DISCERN questionnaire, the median overall quality for all videos assessed showed a moderate* |

| | | | | |
|---|---|---|---|---|
| | | | | *quality (2.5–3.4 points) for question 16 ("Based on the answers to the above questions, rate the overall quality").* |
| | | (*b*) Report category boundaries when continuous variables were categorized | NA | |
| | | (*c*) If relevant, consider translating estimates of relative risk into absolute risk for a meaningful time period | NA | |
| Other analyses | 17 | Report other analyses done—eg analyses of subgroups and interactions, and sensitivity analyses | 4 | Median total DISCERN score of all videos assessed by language showed similar results: English (39.75 points), French (38 points), German (39.5 points), and Italian (39 points) (Fig. 2). |
| Key results | 18 | Summarise key results with reference to study objectives | 5 | The level of quality across the different urological diseases and different languages assessed by use of the DISCERN questionnaire seemed to be low to moderate. |
| Limitations | 19 | Discuss limitations of the study, taking into account sources of potential bias or imprecision. Discuss both direction and magnitude of any potential bias | 6 | *Our present study has some limitations which must be addressed.* |
| Interpretation | 20 | Give a cautious overall interpretation of results considering objectives, limitations, multiplicity of analyses, results from similar studies, and other relevant evidence | 7 | *These findings therefore further highlight the importance of unbiased patient information and underline the role international and national medical societies should play in distributed patient-directed information.* |
| Generalisability | 21 | Discuss the generalisability (external validity) of the study results | 6 | *as 20 videos per topic and language were analyzed, some videos were included which had a relatively low number of views compared to the most popular videos* |
| **Other information** | | | | |

| | | | | |
|---|---|---|---|---|
| Funding | 22 | Give the source of funding and the role of the funders for the present study and, if applicable, for the original study on which the present article is based | 7 | *No funding was received to conduct this trial and no direct or indirect commercial incentive associated with publishing this article* |

Table S126. Quality Evaluation for Included Studies Using STROBE

50.STROBE Statement—checklist of items that should be included in reports of observational studies

| | Item No. | Recommendation | Page No. | Relevant text from manuscript |
|---|---|---|---|---|
| **Title and abstract** | 1 | (*a*) Indicate the study's design with a commonly used term in the title or the abstract | 1 | Quality analysis |
| | | (*b*) Provide in the abstract an informative and balanced summary of what was done and what was found | 1 | The search was performed by using term 'testicular cancer' on YouTube … There is a positive correlation between the video length, DISCERN, JAMA scores and GQS. |
| **Introduction** | | | | |
| Background/rationale | 2 | Explain the scientific background and rationale for the investigation being reported | 1 | *Although many people can access information on the Internet, it is not possible for all of them to evaluate the quality and accuracy of this information. Videos on YouTube do not go through an evaluation process and are not regularly updated.* |
| Objectives | 3 | State specific objectives, including any prespecified hypotheses | 2 | *investigate the content, reliability and quality of YouTube contents, accessed by use of the keyword 'testicular cancer'.* |
| **Methods** | | | | |
| Study design | 4 | Present key elements of study design early in the paper | 2 | Cross-sectional search |
| Setting | 5 | Describe the setting, locations, and relevant dates, including periods of recruitment, exposure, follow-up, and data collection | 2 | We searched the term 'testicular cancer' on YouTube (http://www.youtu be.com) on 15 February 2021 without filters |
| Participants | 6 | (*a*) *Cohort study*—Give the eligibility criteria, and the sources and methods of selection of participants. Describe methods of follow-up | 2 | After the duplicate videos, non-English videos, advertisements and videos without audio found during the YouTube search were |

| | | | | |
|---|---|---|---|---|
| | | *Case-control study*—Give the eligibility criteria, and the sources and methods of case ascertainment and control selection. Give the rationale for the choice of cases and controls<br><br>*Cross-sectional study*—Give the eligibility criteria, and the sources and methods of selection of participants | | excluded; a total of 152 videos were included in the study. |
| | | (*b*) *Cohort study*—For matched studies, give matching criteria and number of exposed and unexposed<br><br>*Case-control study*—For matched studies, give matching criteria and the number of controls per case | NA | |
| Variables | 7 | Clearly define all outcomes, exposures, predictors, potential confounders, and effect modifiers. Give diagnostic criteria, if applicable | 2 | *The length, upload date, number of comments, likes, dislikes and views of each video were recorded. To assess the popularity of videos, the like ratio (like/[like + dislike]) and Video Power Index (VPI)*<br><br>*(like ratio × view ratio [view per day/100]) were calculated.* |
| Data sources/ measurement | 8* | For each variable of interest, give sources of data and details of methods of assessment (measurement). Describe comparability of assessment methods if there is more than one group | *2* | *Videos were from YouTube*<br><br>*Two independent urologists with board certification (Fellow of the European Board of Urology) viewed and analysed the videos.* |
| Bias | 9 | Describe any efforts to address potential sources of bias | 2 | *Both urologists were unaware of the results of each other's evaluation.* |
| Study size | 10 | Explain how the study size was arrived at | 2 | 152 videos |

| | | | | |
|---|---|---|---|---|
| Quantitative variables | 11 | Explain how quantitative variables were handled in the analyses. If applicable, describe which groupings were chosen and why | 2 | *number of comments, likes, dislikes and views of each video were recorded. To assess the popularity of videos, the like ratio and Video Power Index* |

| | | | | |
|---|---|---|---|---|
| | | | | *GQS, modified DISCERN, JAMA scoring* |
| Statistical methods | 12 | (*a*) Describe all statistical methods, including those used to control for confounding | 2 | *Mean, standard deviation, median, minimum, maximum, frequency and percentage were used as descriptive methods. Shapiro–Wilk test was performed to assess the normality of the distribution. For the comparison of categorical data, the chi-square test was applied. The Kruskal–Wallis test was used for the comparison of means. For pairwise analysis, Bonferroni correction was carried out. Spearman test was performed for correlation analysis.* |
| | | (*b*) Describe any methods used to examine subgroups and interactions | 2 | *The Kruskal–Wallis test was used for the comparison of means.* |
| | | (*c*) Explain how missing data were addressed | NA | |
| | | (*d*) *Cohort study*—If applicable, explain how loss to follow-up was addressed<br>*Case-control study*—If applicable, explain how matching of cases and controls was addressed<br>*Cross-sectional study*—If applicable, describe analytical methods taking account of sampling strategy | NA | |
| | | (*e*) Describe any sensitivity analyses | NA | |
| **Results** | | | | |
| Participants | 13* | (a) Report numbers of individuals at each stage of study—eg numbers potentially eligible, examined for eligibility, confirmed eligible, included in the study, completing follow-up, and analysed | 2 | The number of analysed videos was 152. Table 1 shows the various characteristics of the videos. |
| | | (b) Give reasons for non-participation at each stage | NA | |
| | | (c) Consider use of a flow diagram | NA | |
| Descriptive data | 14* | (a) Give characteristics of study participants (eg demographic, clinical, social) and information on exposures and potential confounders | 2 | The number of analysed videos was 152. Table 1 shows the |

| | | | | |
|---|---|---|---|---|
| | | | | various characteristics of the videos. |
| | | (b) Indicate number of participants with missing data for each variable of interest | NA | |
| | | (c) *Cohort study*—Summarise follow-up time (eg, average and total amount) | NA | |
| Outcome data | 15* | *Cohort study*—Report numbers of outcome events or summary measures over time | | |
| | | *Case-control study*—Report numbers in each exposure category, or summary measures of exposure | | |
| | | *Cross-sectional study*—Report numbers of outcome events or summary measures | *2-3* | *The mean JAMA score was 1.59 ± 1.11, … dislikes, comments, views, VPI and view per day (p > 0.05).* |
| Main results | 16 | (*a*) Give unadjusted estimates and, if applicable, confounder-adjusted estimates and their precision (eg, 95% confidence interval). Make clear which confounders were adjusted for and why they were included | 2 | *According to modified DISCERN classification, 63.2% were 'poor', 26.9% were 'fair', and 9.9% were 'good'.* |
| | | (*b*) Report category boundaries when continuous variables were categorized | NA | |
| | | (*c*) If relevant, consider translating estimates of relative risk into absolute risk for a meaningful time period | NA | |

| | | | | |
|---|---|---|---|---|
| Other analyses | 17 | Report other analyses done—eg analyses of subgroups and interactions, and sensitivity analyses | 3 | Correlation analyses for modified DISCERN scores, GQS score and JAMA score are presented in Table 4. |

| | | | | |
|---|---|---|---|---|
| Key results | 18 | Summarise key results with reference to study objectives | 3 | YouTube videos were accessed by millions of people, and the quality and reliability of TC information presented on YouTube was poor and inadequate. |
| Limitations | 19 | Discuss limitations of the study, taking into account sources of potential bias or imprecision. Discuss both direction and magnitude of any potential bias | 6 | *One of the limitations of this study is the relatively subjective …* |
| Interpretation | 20 | Give a cautious overall interpretation of results considering objectives, limitations, multiplicity of analyses, results from similar studies, and other relevant evidence | 6 | *Considering the importance of early diagnosis in TC, which has a high cure rate, uploading high-* |

| | | | | |
|---|---|---|---|---|
| | | | | *quality videos of optimum length to relevant platforms and having videos containing quality information is important in terms of both patient and public health, as well as for cost.* |
| Generalisability | 21 | Discuss the generalisability (external validity) of the study results | 6 | *the evaluation of YouTube videos in only English language* |
| **Other information** | | | | |
| Funding | 22 | Give the source of funding and the role of the funders for the present study and, if applicable, for the original study on which the present article is based | NA | |

Table S127. Quality Evaluation for Included Studies Using STROBE

51.STROBE Statement—checklist of items that should be included in reports of observational studies

| | Item No. | Recommendation | Page No. | Relevant text from manuscript |
|---|---|---|---|---|
| **Title and abstract** | 1 | (*a*) Indicate the study's design with a commonly used term in the title or the abstract | 1 | Quality evaluation |
| | | (*b*) Provide in the abstract an informative and balanced summary of what was done and what was found | 1 | Methods and Results |
| **Introduction** | | | | |
| Background/rationale | 2 | Explain the scientific background and rationale for the investigation being reported | 2 | *The video-sharing platform, YouTube™, has become a source of information for patients seeking medical information, including prostate cancer[4]. However, material in the Arabic language is scarce.* |
| Objectives | 3 | State specific objectives, including any prespecified hypotheses | 2 | *This study aims to objectively evaluate the quality and reliability of the information on YouTube™ of prostate cancer videos published in Arabic.* |
| **Methods** | | | | |
| Study design | 4 | Present key elements of study design early in the paper | 2 | Cross-sectional search study |
| Setting | 5 | Describe the setting, locations, and relevant dates, including periods of recruitment, exposure, follow-up, and data collection | 2 | *The default search setting on YouTube™ was used, which automatically sorts videos by relevance.*<br>*A total of 100 videos were collected, over 4 months (26.06.2022–27.10.2022) during the searches for the search terms* |
| Participants | 6 | (*a*) *Cohort study*—Give the eligibility criteria, and the sources and methods of selection of participants. Describe methods of follow-up<br>*Case-control study*—Give the eligibility criteria, and the sources and methods of case ascertainment and control selection. Give the rationale for the choice of cases and controls | 2 | *Videos included were in Arabic, and others were excluded if they were recurrent in part or whole, not uploaded in Arabic language, or did not contain* |

| | | | | |
|---|---|---|---|---|
| | | *Cross-sectional study*—Give the eligibility criteria, and the sources and methods of selection of participants | | *information on prostate cancer.* |
| | | (*b*) *Cohort study*—For matched studies, give matching criteria and number of exposed and unexposed<br>*Case-control study*—For matched studies, give matching criteria and the number of controls per case | NA | |
| Variables | 7 | Clearly define all outcomes, exposures, predictors, potential confounders, and effect modifiers. Give diagnostic criteria, if applicable | 2 | *Several video characteristics were abstracted, including the publisher, the speaker (health worker, non-medical), upload location, number of views, length (minutes), number of likes, number of comments, and days available on YouTube groups according to content as useful, misleading, personal experience, or irrelevant.*<br>*GQS, modified DISCERN tool* |
| Data sources/ measurement | 8* | For each variable of interest, give sources of data and details of methods of assessment (measurement). Describe comparability of assessment methods if there is more than one group | *2* | *Videos were from YouTube* |
| Bias | 9 | Describe any efforts to address potential sources of bias | 2 | *Videos included were in Arabic, and others were excluded if they were recurrent in part or whole, not uploaded in Arabic language, or did not contain information on prostate cancer.* |
| Study size | 10 | Explain how the study size was arrived at | 2 | 100 videos |

| | | | | |
|---|---|---|---|---|
| Quantitative variables | 11 | Explain how quantitative variables were handled in the analyses. If applicable, describe which groupings were chosen and why | 2 | *number of views, length (minutes), number of likes, number of comments, GQS, modified DISCERN tool* |
| Statistical methods | 12 | (*a*) Describe all statistical methods, including those used to control for confounding | 3 | *Descriptive statistics were used to report the outcomes of this study. Continuous variables* |

| | | | | |
|---|---|---|---|---|
| | | | | *were reported as a mean, range and SD. Categorical variables were summarised as a number and percentages. The two-sample t-test was used to assess the quality analysis and misinformation tools, and a p-value of <0.05 was considered statistically significant. Mann-Whitney U test was also used to compare distributions when the data were not normally distributed.* |
| | | (*b*) Describe any methods used to examine subgroups and interactions | NA | |
| | | (*c*) Explain how missing data were addressed | NA | |
| | | (*d*) *Cohort study*—If applicable, explain how loss to follow-up was addressed<br>*Case-control study*—If applicable, explain how matching of cases and controls was addressed<br>*Cross-sectional study*—If applicable, describe analytical methods taking account of sampling strategy | NA | |
| | | (*e*) Describe any sensitivity analyses | NA | |
| **Results** | | | | |
| Participants | 13* | (a) Report numbers of individuals at each stage of study—eg numbers potentially eligible, examined for eligibility, confirmed eligible, included in the study, completing follow-up, and analysed | 3 | *A total of 110 videos were initially collected, and 10 videos in total were excluded. Seven were excluded due to different languages other than Arabic language, and three excluded for duplicated in part or whole.* |
| | | (b) Give reasons for non-participation at each stage | NA | |
| | | (c) Consider use of a flow diagram | NA | |
| Descriptive data | 14* | (a) Give characteristics of study participants (eg demographic, clinical, social) and information on exposures and potential confounders | 3 | *The majority of the included videos were published by independent users … irrelevant in 6%, and depicted personal experience in 4% of videos.* |
| | | (b) Indicate number of participants with missing data for each variable of interest | NA | |

| | | | | |
|---|---|---|---|---|
| | | (c) *Cohort study*—Summarise follow-up time (eg, average and total amount) | NA | |
| Outcome data | 15* | *Cohort study*—Report numbers of outcome events or summary measures over time | | |
| | | *Case-control study*—Report numbers in each exposure category, or summary measures of exposure | | |
| | | *Cross-sectional study*—Report numbers of outcome events or summary measures | *3* | *The median of the modified DISCERN score of videos presented by health workers was higher compared to videos presented by non-health workers (4 versus 2; p < 0.00001). A significant proportion of 78% of low-reliability videos (<=2) was presented by non-health workers. The GQS tool revealed that videos developed by health workers compared to videos presented by non-health workers had higher moderate to excellent levels of quality (80.5% versus 21.7%, p < 0.05)* |
| Main results | 16 | (*a*) Give unadjusted estimates and, if applicable, confounder-adjusted estimates and their precision (eg, 95% confidence interval). Make clear which confounders were adjusted for and why they were included | 3 | *The median of the modified DISCERN score of videos presented by health workers was higher compared to videos presented by non-health workers (4 versus 2; p < 0.00001). A significant proportion of 78% of low-reliability videos (<=2) was presented by non-health workers.* |
| | | (*b*) Report category boundaries when continuous variables were categorized | NA | |
| | | (*c*) If relevant, consider translating estimates of relative risk into absolute risk for a meaningful time period | NA | |
| Other analyses | 17 | Report other analyses done—eg analyses of subgroups and interactions, and sensitivity analyses | NA | |

| | | | | |
|---|---|---|---|---|
| Key results | 18 | Summarise key results with reference to study objectives | 4 | *videos about prostate cancer in Arabic were uploaded by medical personnel, and 92% were deemed useful. While videos were uploaded by non-medical professionals, 78% had low reliability, and only 56% were deemed useful. This shows the positive and critical role of specialists in the region to educate the public.* |
| Limitations | 19 | Discuss limitations of the study, taking into account sources of potential bias or imprecision. Discuss both direction and magnitude of any potential bias | 5 | *This study has several limitations including the fact…* |
| Interpretation | 20 | Give a cautious overall interpretation of results considering objectives, limitations, multiplicity of analyses, results from similar studies, and other relevant evidence | 5 | *The medical community needs to be engaged directly on social media platforms and provide a credible voice to the conversation and highlight reliable sources to the public from those sites providing misinformation, as physicians, and specialised allied healthcare professionals remain the single most credible voice in healthcare.* |
| Generalisability | 21 | Discuss the generalisability (external validity) of the study results | 5 | *uploaded video content and dates might not be aligned with the most updated recommendations for prostate cancer treatment and screening. Additionally, using a cross-sectional study is limited by the continuously changing content on the internet which cannot be accurately captured* |
| **Other information** | | | | |
| Funding | 22 | Give the source of funding and the role of the funders for the present study and, if applicable, for the original study on which the present article is based | 5 | *the work was not funded or supported by any organisation.* |

Table S128. Quality Evaluation for Included Studies Using STROBE

52.STROBE Statement—checklist of items that should be included in reports of observational studies

| | Item No. | Recommendation | Page No. | Relevant text from manuscript |
|---|---|---|---|---|
| **Title and abstract** | 1 | (*a*) Indicate the study's design with a commonly used term in the title or the abstract | 1 | Cross-sectional search |
| | | (*b*) Provide in the abstract an informative and balanced summary of what was done and what was found | 1 | We reviewed the first 150 YouTube videos about "bladder cancer" … advice (20%), provide medical advice to others (9%), or give support (19%). |
| **Introduction** | | | | |
| Background/rationale | 2 | Explain the scientific background and rationale for the investigation being reported | 1 | Previous studies have highlighted the spread of biased and/or misinformative content about urological conditions on YouTube |
| Objectives | 3 | State specific objectives, including any prespecified hypotheses | 2 | characterize the quality of information and presence of misinformation about bladder cancer on YouTube. |
| **Methods** | | | | |
| Study design | 4 | Present key elements of study design early in the paper | 2 | Cross-sectional evaluation |
| Setting | 5 | Describe the setting, locations, and relevant dates, including periods of recruitment, exposure, follow-up, and data collection | 2 | We reviewed the first 150 of 242 000 YouTube videos on "bladder cancer" |
| Participants | 6 | (*a*) *Cohort study*—Give the eligibility criteria, and the sources and methods of selection of participants. Describe methods of follow-up<br>*Case-control study*—Give the eligibility criteria, and the sources and methods of case ascertainment and control selection. Give the rationale for the choice of cases and controls<br>*Cross-sectional study*—Give the eligibility criteria, and the sources and methods of selection of participants | 2 | We reviewed the first 150 of 242 000 YouTube videos on "bladder cancer" |
| | | (*b*) *Cohort study*—For matched studies, give matching criteria and number of exposed and unexposed | | |

| | | | | |
|---|---|---|---|---|
| | | *Case-control study*—For matched studies, give matching criteria and the number of controls per case | | |
| Variables | 7 | Clearly define all outcomes, exposures, predictors, potential confounders, and effect modifiers. Give diagnostic criteria, if applicable | NA | |
| Data sources/ measurement | 8* | For each variable of interest, give sources of data and details of methods of assessment (measurement). Describe comparability of assessment methods if there is more than one group | *2* | *Videos were from YouTube*<br>*Videos were independently coded by urologists and urology trainees with random coding checks to verify intercoder reliability.* |
| Bias | 9 | Describe any efforts to address potential sources of bias | 2 | *Videos were independently coded by urologists and urology trainees with random coding checks to verify intercoder reliability.* |
| Study size | 10 | Explain how the study size was arrived at | 2 | 150 videos |

| | | | | |
|---|---|---|---|---|
| Quantitative variables | 11 | Explain how quantitative variables were handled in the analyses. If applicable, describe which groupings were chosen and why | 2 | *PEMAT, DISCERN, five-point Likert scale* |
| Statistical methods | 12 | (*a*) Describe all statistical methods, including those used to control for confounding | 2 | *Descriptive statistics*<br>*Pearson correlation coefficients were used to examine the relationship between viewer engagement (views per month) and misinformation.* |
| | | (*b*) Describe any methods used to examine subgroups and interactions | 3 | Table 2 |
| | | (*c*) Explain how missing data were addressed | NA | |
| | | (*d*) *Cohort study*—If applicable, explain how loss to follow-up was addressed<br>*Case-control study*—If applicable, explain how matching of cases and controls was addressed<br>*Cross-sectional study*—If applicable, describe analytical methods taking account of sampling strategy | NA | |
| | | (*e*) Describe any sensitivity analyses | NA | |

| | | | | |
|---|---|---|---|---|
| **Results** | | | | |
| Participants | 13* | (a) Report numbers of individuals at each stage of study—eg numbers potentially eligible, examined for eligibility, confirmed eligible, included in the study, completing follow-up, and analysed | 2 | The first 150 videos about bladder cancer covered a range of topics, most commonly treatment followed by signs/ detection (Table 1). |
| | | (b) Give reasons for non-participation at each stage | NA | |
| | | (c) Consider use of a flow diagram | NA | |
| Descriptive data | 14* | (a) Give characteristics of study participants (eg demographic, clinical, social) and information on exposures and potential confounders | 2 | Most of the videos were published by hospitals/clinics, foundations/advocacy groups, and health/ wellness channels, and 57% featured a doctor. The median number of views was 2288, and most videos were aimed at the public. |
| | | (b) Indicate number of participants with missing data for each variable of interest | NA | |
| | | (c) *Cohort study*—Summarise follow-up time (eg, average and total amount) | NA | |
| Outcome data | 15* | *Cohort study*—Report numbers of outcome events or summary measures over time | | |
| | | *Case-control study*—Report numbers in each exposure category, or summary measures of exposure | | |
| | | *Cross-sectional study*—Report numbers of outcome events or summary measures | *2-3* | *However, the median understandability and actionability of videos were 71% and 33%, respectively …* |
| Main results | 16 | (*a*) Give unadjusted estimates and, if applicable, confounder-adjusted estimates and their precision (eg, 95% confidence interval). Make clear which confounders were adjusted for and why they were included | 2 | *Overall, the quality of information was moderate to poor in 67% of videos (scores of 1–3 out of 5 on the overall DISCERN criteria), and these videos had >1.8 million views* |
| | | (*b*) Report category boundaries when continuous variables were categorized | NA | |
| | | (*c*) If relevant, consider translating estimates of relative risk into absolute risk for a meaningful time period | NA | |

| | | | | |
|---|---|---|---|---|
| Other analyses | 17 | Report other analyses done—eg analyses of subgroups and interactions, and sensitivity analyses | NA | |
| Key results | 18 | Summarise key results with reference to study objectives | 3 | *Our finding of misinformation in 29% of the top bladder cancer videos on YouTube is in line with previous studies showing that 40% of YouTube videos about prostate cancer and 35% about colonoscopy contain misinformation* |
| Limitations | 19 | Discuss limitations of the study, taking into account sources of potential bias or imprecision. Discuss both direction and magnitude of any potential bias | 3 | A limitation of our study is that… |
| Interpretation | 20 | Give a cautious overall interpretation of results considering objectives, limitations, multiplicity of analyses, results from similar studies, and other relevant evidence | 3 | *Healthcare providers should recommend trustworthy sources of additional information for patients and should actively participate in social media for dissemination of evidence based medicine.* |
| Generalisability | 21 | Discuss the generalisability (external validity) of the study results | 3 | *we evaluated videos on YouTube only and not on other online networks;* |
| **Other information** | | | | |
| Funding | 22 | Give the source of funding and the role of the funders for the present study and, if applicable, for the original study on which the present article is based | 3 | Funding/Support and role of the sponsor: Stacy Loeb is supported by the Edward Blank and Sharon Cosloy-Blank Family Foundation. |

Table S129. Quality Evaluation for Included Studies Using STROBE

53.STROBE Statement—checklist of items that should be included in reports of observational studies

| | Item No. | Recommendation | Page No. | Relevant text from manuscript |
|---|---|---|---|---|
| **Title and abstract** | 1 | (*a*) Indicate the study's design with a commonly used term in the title or the abstract | 1 | *cross-sectional descriptive study* |
| | | (*b*) Provide in the abstract an informative and balanced summary of what was done and what was found | 1 | Materials and Methods<br>Results |
| **Introduction** | | | | |
| Background/rationale | 2 | Explain the scientific background and rationale for the investigation being reported | 1 | *YouTube is one of the most popular social networks used as a source of advice [1, 2, 3]. However, the dissemination of information found on this platform is often erroneous, creating a problem of misinformation.* |
| Objectives | 3 | State specific objectives, including any prespecified hypotheses | 1 | *characterise the quality of information and the presence of misinformation about bladder cancer in videos published on the YouTube platform in Spanish.* |
| **Methods** | | | | |
| Study design | 4 | Present key elements of study design early in the paper | 1 | *cross-sectional descriptive study* |
| Setting | 5 | Describe the setting, locations, and relevant dates, including periods of recruitment, exposure, follow-up, and data collection | 1 | *The first 50 videos in Spanish published on YouTube, searching on 9 March 2021 in incognito mode, without a logged-in session, using 'bladder cancer' as the search criterion.* |
| Participants | 6 | (*a*) *Cohort study*—Give the eligibility criteria, and the sources and methods of selection of participants. Describe methods of follow-up | 1 | *Those with duration <4 minutes were filtered, sorting them by number of views and excluding videos without text or voice,* |

| | | | | |
|---|---|---|---|---|
| | | *Case-control study*—Give the eligibility criteria, and the sources and methods of case ascertainment and control selection. Give the rationale for the choice of cases and controls<br><br>*Cross-sectional study*—Give the eligibility criteria, and the sources and methods of selection of participants | | |
| | | (*b*) *Cohort study*—For matched studies, give matching criteria and number of exposed and unexposed<br><br>*Case-control study*—For matched studies, give matching criteria and the number of controls per case | | |
| Variables | 7 | Clearly define all outcomes, exposures, predictors, potential confounders, and effect modifiers. Give diagnostic criteria, if applicable | 2 | *the year of publication, video duration, number of 'likes' and 'dislikes', views, and comments, video content, target audience, main topic, description provided, author of the channel and protagonist of the video.* |
| Data sources/ measurement | 8* | For each variable of interest, give sources of data and details of methods of assessment (measurement). Describe comparability of assessment methods if there is more than one group | 2 | *Videos were from YouTube*<br><br>*The videos were evaluated by three urologists using two validated questionnaires* |
| Bias | 9 | Describe any efforts to address potential sources of bias | 2 | *The videos were evaluated by three urologists using two validated questionnaires* |
| Study size | 10 | Explain how the study size was arrived at | 1 | 38 videos |

| | | | | |
|---|---|---|---|---|
| Quantitative variables | 11 | Explain how quantitative variables were handled in the analyses. If applicable, describe which groupings were chosen and why | 2 | *video duration, number of 'likes' and 'dislikes', views, and comments, video content, target audience*<br><br>*PEMAT and DISCERN* |
| Statistical methods | 12 | (*a*) Describe all statistical methods, including those used to control for confounding | 2 | *Univariate analysis was performed according to the DISCERN questionnaire classification (poor quality vs. moderate/good quality).* |
| | | (*b*) Describe any methods used to examine subgroups and interactions | NA | |

| | | | | |
|---|---|---|---|---|
| | | (*c*) Explain how missing data were addressed | NA | |
| | | (*d*) *Cohort study*—If applicable, explain how loss to follow-up was addressed<br>*Case-control study*—If applicable, explain how matching of cases and controls was addressed<br>*Cross-sectional study*—If applicable, describe analytical methods taking account of sampling strategy | NA | |
| | | (*e*) Describe any sensitivity analyses | NA | |
| **Results** | | | | |
| Participants | 13* | (a) Report numbers of individuals at each stage of study—eg numbers potentially eligible, examined for eligibility, confirmed eligible, included in the study, completing follow-up, and analysed | 1 | *Those with duration <4 minutes were filtered, sorting them by number of views and excluding videos without text or voice, leaving 38 for analysis.* |
| | | (b) Give reasons for non-participation at each stage | NA | |
| | | (c) Consider use of a flow diagram | 3 | Figure 1 |
| Descriptive data | 14* | (a) Give characteristics of study participants (eg demographic, clinical, social) and information on exposures and potential confounders | 2 | *The descriptive analysis of the characteristics of the videos is shown in …* |
| | | (b) Indicate number of participants with missing data for each variable of interest | NA | |
| | | (c) *Cohort study*—Summarise follow-up time (eg, average and total amount) | NA | |
| Outcome data | 15* | *Cohort study*—Report numbers of outcome events or summary measures over time | | |
| | | *Case-control study*—Report numbers in each exposure category, or summary measures of exposure | | |
| | | *Cross-sectional study*—Report numbers of outcome events or summary measures | *3* | *Regarding the validated questionnaires for consumer health information: videos had a median score of 71.6% (16.5–100%) in PEMAT…* |
| Main results | 16 | (*a*) Give unadjusted estimates and, if applicable, confounder-adjusted estimates and their precision (eg, 95% confidence interval). Make clear which confounders were adjusted for and why they were included | 3 | *26/38 videos (66.7%) were of poor quality and 12/38 (30.8%) were of moderate or good quality* |
| | | (*b*) Report category boundaries when continuous variables were categorized | NA | |

| | | | | |
|---|---|---|---|---|
| | | (*c*) If relevant, consider translating estimates of relative risk into absolute risk for a meaningful time period | NA | |
| Other analyses | 17 | Report other analyses done—eg analyses of subgroups and interactions, and sensitivity analyses | NA | |
| Key results | 18 | Summarise key results with reference to study objectives | 3 | *the median understanding and action of the videos was 71% and 33% respectively (according to the PEMAT questionnaire); nd that the quality of the information was moderate to poor in 67% of the videos (scores of 1–3 out of 5 in the general DISCERN criteria) [1]. These data coincide with the results obtained in our study.* |
| Limitations | 19 | Discuss limitations of the study, taking into account sources of potential bias or imprecision. Discuss both direction and magnitude of any potential bias | 4 | *A limitation of our study is that we only …* |
| Interpretation | 20 | Give a cautious overall interpretation of results considering objectives, limitations, multiplicity of analyses, results from similar studies, and other relevant evidence | 4 | *The findings of this study lead us to consider improving the quality of information posted on social media as a quality objective for health service providers, such as health services or scientific societies.* |
| Generalisability | 21 | Discuss the generalisability (external validity) of the study results | 4 | *we only evaluated videos on YouTube and not on other social networks, as well as using the filter of videos in Spanish and <4 minutes long* |
| **Other information** | | | | |
| Funding | 22 | Give the source of funding and the role of the funders for the present study and, if applicable, for the original study on which the present article is based | NA | |

Table S130. Quality Evaluation for Included Studies Using STROBE

54.STROBE Statement—checklist of items that should be included in reports of observational studies

| | Item No. | Recommendation | Page No. | Relevant text from manuscript |
|---|---|---|---|---|
| **Title and abstract** | 1 | (*a*) Indicate the study's design with a commonly used term in the title or the abstract | 1 | Cross-sectional search |
| | | (*b*) Provide in the abstract an informative and balanced summary of what was done and what was found | 1 | "*A search of YouTube was performed on February 12, 2018, using the search terms … by YouTube videos pertaining to thyroid cancer based on DISCERN and JAWA scores*" |
| **Introduction** | | | | |
| Background/rationale | 2 | Explain the scientific background and rationale for the investigation being reported | 1 | *YouTube has a potential to influence patients' perceptions about the diagnosis and expectations regarding treatment outcome along with a capacity to influence patient-clinician interactions. Accordingly, the unregulated nature of its content and lack of peer review increases the likelihood of the information received to be neither accurate nor free of bias, raising a concern about the quality of provided information and thus its safe use in medical conditions* |
| Objectives | 3 | State specific objectives, including any prespecified hypotheses | 2 | *assess the quality of information available on YouTube videos pertaining to thyroid cancer.* |
| **Methods** | | | | |
| Study design | 4 | Present key elements of study design early in the paper | 2 | Cross-sectional search |
| Setting | 5 | Describe the setting, locations, and relevant dates, including periods of recruitment, exposure, follow-up, and data collection | 2 | *A search of YouTube was performed on February 12, 2018, using the search terms* |

| | | | | |
|---|---|---|---|---|
| | | | | *'Bthyroid cancer' and 'Bthyroid cancer treatment.'* |
| Participants | 6 | (*a*) *Cohort study*—Give the eligibility criteria, and the sources and methods of selection of participants. Describe methods of follow-up<br>*Case-control study*—Give the eligibility criteria, and the sources and methods of case ascertainment and control selection. Give the rationale for the choice of cases and controls<br>*Cross-sectional study*—Give the eligibility criteria, and the sources and methods of selection of participants | 2 | *The first 50 videos that appeared on each search were reviewed. Of 100 videos initially involved, 52 videos were considered to be eligible to be included in the analysis with exclusion of 48 videos related to advertisements (n = 2), duplicate videos (n = 13), non-English videos (n = 8), and videos with less than 5000 views (n = 25)* |
| | | (*b*) *Cohort study*—For matched studies, give matching criteria and number of exposed and unexposed<br>*Case-control study*—For matched studies, give matching criteria and the number of controls per case | NA | |
| Variables | 7 | Clearly define all outcomes, exposures, predictors, potential confounders, and effect modifiers. Give diagnostic criteria, if applicable | 2 | *Video characteristics including publishing source of upload (academic, non-expert, expert, and media), continent of origin, number of views, number of likes, number of dislikes, and presence of animation were recorded. The quality of information provided was assessed using the DISCERN [24] and JAMA benchmark [25] scores, while video power index was calculated using the formula: Bnumber of likes/(number of likes + dislikes) Å~ 100.^* |
| Data sources/ measurement | 8* | For each variable of interest, give sources of data and details of methods of assessment (measurement). Describe comparability of assessment methods if there is more than one group | 2 | *Videos were from YouTube*<br>*Videos were independently analyzed by two authors* |
| Bias | 9 | Describe any efforts to address potential sources of bias | 2 | *exclusion of 48 videos related to advertisements (n = 2), duplicate videos (n* |

| | | | | |
|---|---|---|---|---|
| | | | | *=13), non-English videos (n = 8),* |
| Study size | 10 | Explain how the study size was arrived at | 2 | 52 videos |
| Quantitative variables | 11 | Explain how quantitative variables were handled in the analyses. If applicable, describe which groupings were chosen and why | 2 | *number of views, number of likes, number of dislikes, DISCERN [24] and JAMA benchmark [25] scores, video power index* |
| Statistical methods | 12 | (*a*) Describe all statistical methods, including those used to control for confounding | 2 | *Descriptive statistics were reported including Bmean (standard deviation; SD)^ and median (min-max) medians for continuous variables and percentages for categorical variables. Mann- Whitney U test was used for the numerical variables, while correlation analysis was performed using Pearson correlation analysis.* |
| | | (*b*) Describe any methods used to examine subgroups and interactions | 4 | Table 3 |
| | | (*c*) Explain how missing data were addressed | NA | |
| | | (*d*) *Cohort study*—If applicable, explain how loss to follow-up was addressed<br>*Case-control study*—If applicable, explain how matching of cases and controls was addressed<br>*Cross-sectional study*—If applicable, describe analytical methods taking account of sampling strategy | NA | |
| | | (*e*) Describe any sensitivity analyses | NA | |
| **Results** | | | | |
| Participants | 13* | (a) Report numbers of individuals at each stage of study—eg numbers potentially eligible, examined for eligibility, confirmed eligible, included in the study, completing follow-up, and analysed | 2 | *Overall, most of the videos (51.9%) were uploaded by nonexperts, as followed by experts (26.9%), media (17.3%), and academic institutions (3.8%). The continent of origin was North America in 88.5%of videos,* |

| | | | | |
|---|---|---|---|---|
| | | | | *while 21.2% were animated videos (Table 1).* |
| | | (b) Give reasons for non-participation at each stage | NA | |
| | | (c) Consider use of a flow diagram | 3 | Fig. 1 |
| Descriptive data | 14* | (a) Give characteristics of study participants (eg demographic, clinical, social) and information on exposures and potential confounders | 2 | *Average duration of videos was 12.8 (range 0.9–79.7) min.* *Median (min-max) number of views, likes, and dislikes were 17,424 (5865–506,525), 89.0 (0–871), and 5.0 (0–57), respectively.* *The median (min-max) video power index was 94.0 (0–100)* |
| | | (b) Indicate number of participants with missing data for each variable of interest | NA | |
| | | (c) *Cohort study*—Summarise follow-up time (eg, average and total amount) | NA | |
| Outcome data | 15* | *Cohort study*—Report numbers of outcome events or summary measures over time | | |
| | | *Case-control study*—Report numbers in each exposure category, or summary measures of exposure | | |
| | | *Cross-sectional study*—Report numbers of outcome events or summary measures | *3* | *"The median (min-max) DISCERN score was … power index, and DISCERN and JAMA scores"* |
| Main results | 16 | (*a*) Give unadjusted estimates and, if applicable, confounder-adjusted estimates and their precision (eg, 95% confidence interval). Make clear which confounders were adjusted for and why they were included | 3 | *Both reviewers indicated DISCERN score ≥ 60 only for five (9.6%) videos* |
| | | (*b*) Report category boundaries when continuous variables were categorized | NA | |
| | | (*c*) If relevant, consider translating estimates of relative risk into absolute risk for a meaningful time period | NA | |

| | | | | |
|---|---|---|---|---|
| Other analyses | 17 | Report other analyses done—eg analyses of subgroups and interactions, and sensitivity analyses | 3 | *Correlations Between Descriptive Parameters* |

| | | | | |
|---|---|---|---|---|
| Key results | 18 | Summarise key results with reference to study objectives | 4 | *Our findings related to poor quality of information provided by YouTube videos pertaining to thyroid cancer with DISCERN scores of ≥ 60 and JAMA score of 4 only for 10% of videos seem consistent with data from past studies indicating the unreliability and poor quality of YouTube videos related to various diseases* |
| Limitations | 19 | Discuss limitations of the study, taking into account sources of potential bias or imprecision. Discuss both direction and magnitude of any potential bias | 5 | *Certain limitations to this study should be considered …* |
| Interpretation | 20 | Give a cautious overall interpretation of results considering objectives, limitations, multiplicity of analyses, results from similar studies, and other relevant evidence | 5 | *Future studies with a longitudinal or field-based approach are needed to address the quality of health information provided by YouTube videos as a source for patients within health literacy gap and an effective medium for healthcare communication with potential to improve optimal management* |
| Generalisability | 21 | Discuss the generalisability (external validity) of the study results | 5 | *results might vary with use of different search terms as well as according to the search date and time.* |
| **Other information** | | | | |
| Funding | 22 | Give the source of funding and the role of the funders for the present study and, if applicable, for the original study on which the present article is based | NA | |

Table S131. Quality Evaluation for Included Studies Using STROBE

55.STROBE Statement—checklist of items that should be included in reports of observational studies

| | Item No. | Recommendation | Page No. | Relevant text from manuscript |
|---|---|---|---|---|
| **Title and abstract** | 1 | (*a*) Indicate the study's design with a commonly used term in the title or the abstract | 1 | Cross-sectional search |
| | | (*b*) Provide in the abstract an informative and balanced summary of what was done and what was found | 1 | Methods and Results |
| **Introduction** | | | | |
| Background/rationale | 2 | Explain the scientific background and rationale for the investigation being reported | 2 | *Although patients can access medical information via YouTube, not everyone can assess the quality, reliability, and accuracy of this information. Biased or conflicting advice can not only lower the credibility of physicians but also harm patients, especially when discussing different treatment choices* |
| Objectives | 3 | State specific objectives, including any prespecified hypotheses | 2 | *evaluated the quality of YouTube videos as a source of patient education.* |
| **Methods** | | | | |
| Study design | 4 | Present key elements of study design early in the paper | 2 | cross-sectional study |
| Setting | 5 | Describe the setting, locations, and relevant dates, including periods of recruitment, exposure, follow-up, and data collection | 2 | A search was performed on YouTube on January 14, 2021, using the keywords "meningioma treatment," "meningeal tumor treatment," "meningioma brain tumor treatment," "meningioma cure," and "meningioma therapy." |
| Participants | 6 | (*a*) *Cohort study*—Give the eligibility criteria, and the sources and methods of selection of participants. Describe methods of follow-up | 2 | The review included: (a) English language videos and (b) videos reporting how the treatment works and the benefits, risk factors, and |

| | | | | |
|---|---|---|---|---|
| | | *Case-control study*—Give the eligibility criteria, and the sources and methods of case ascertainment and control selection. Give the rationale for the choice of cases and controls<br>*Cross-sectional study*—Give the eligibility criteria, and the sources and methods of selection of participants | | possible treatment choices for meningioma. We excluded all duplicate or irrelevant videos, where the latter were defined as videos not containing any information about meningioma treatment. |
| | | (*b*) *Cohort study*—For matched studies, give matching criteria and number of exposed and unexposed<br>*Case-control study*—For matched studies, give matching criteria and the number of controls per case | NA | |
| Variables | 7 | Clearly define all outcomes, exposures, predictors, potential confounders, and effect modifiers. Give diagnostic criteria, if applicable | 3 | *We gathered information on the number of views; average daily views (number of views/day); video duration (seconds); the number of comments, likes, dislikes, channel subscribers, and referrers (the number of external webpages linking to the video); and time since the upload date* |
| Data sources/ measurement | 8* | For each variable of interest, give sources of data and details of methods of assessment (measurement). Describe comparability of assessment methods if there is more than one group | *2* | *Videos were from YouTube* |
| Bias | 9 | Describe any efforts to address potential sources of bias | 2 | *The video searches were conducted after clearing the search history and without providing a user id and a password.* |
| Study size | 10 | Explain how the study size was arrived at | 3 | 61 videos |

| | | | | |
|---|---|---|---|---|
| Quantitative variables | 11 | Explain how quantitative variables were handled in the analyses. If applicable, describe which groupings were chosen and why | 2 | *Average daily views (number of views/day); video duration (seconds); the number of comments, likes, dislikes, channel subscribers, and* |

| | | | | |
|---|---|---|---|---|
| | | | | *referrers (the number of external webpages linking to the video);*<br>*The like ratio was calculated according to: [number of likes/ (number of likes þ number of dislikes)]    100, whereas VPI was calculated according to: ratio of likes    ratio of views/100.*<br>*DISCERN* |
| Statistical methods | 12 | (*a*) Describe all statistical methods, including those used to control for confounding | 3 | *The Shapiro-Wilk test was performed to test the normality of data. The Mann-Whitney and Kruskal-Wallis tests were used to determine statistically significant differences between 2 or more than 2 groups of an independent variable, respectively. The Dunn-Bonferroni post hoc method was used after a significant Kruskal-Wallis test for pairwise comparisons.*<br>*For correlation analysis, Spearman's test was performed.* |
| | | (*b*) Describe any methods used to examine subgroups and interactions | 3 | *The Mann-Whitney and Kruskal-Wallis tests were used to determine statistically significant differences between 2 or more than 2 groups of an independent variable, respectively.* |
| | | (*c*) Explain how missing data were addressed | NA | |
| | | (*d*) *Cohort study*—If applicable, explain how loss to follow-up was addressed<br>*Case-control study*—If applicable, explain how matching of cases and controls was addressed<br>*Cross-sectional study*—If applicable, describe analytical methods taking account of sampling strategy | NA | |

| | | | | |
|---|---|---|---|---|
| | | (*e*) Describe any sensitivity analyses | NA | |
| **Results** | | | | |
| Participants | 13* | (a) Report numbers of individuals at each stage of study—eg numbers potentially eligible, examined for eligibility, confirmed eligible, included in the study, completing follow-up, and analysed | 3 | Thirty videos were analyzed for each of the 5 keywords (meningioma treatment, meningeal tumor treatment, meningioma brain tumor treatment, meningioma cure, and meningioma therapy), and 83 duplicates were removed. After screening, using our inclusion and exclusion criteria, 61 videos underwent further analysis |
| | | (b) Give reasons for non-participation at each stage | NA | |
| | | (c) Consider use of a flow diagram | 3 | Figure 1 |
| Descriptive data | 14* | (a) Give characteristics of study participants (eg demographic, clinical, social) and information on exposures and potential confounders | 2 | Table 1 |
| | | (b) Indicate number of participants with missing data for each variable of interest | NA | |
| | | (c) *Cohort study*—Summarise follow-up time (eg, average and total amount) | NA | |
| Outcome data | 15* | *Cohort study*—Report numbers of outcome events or summary measures over time | | |
| | | *Case-control study*—Report numbers in each exposure category, or summary measures of exposure | | |
| | | *Cross-sectional study*—Report numbers of outcome events or summary measures | *3-5* | *The mean total DISCERN score was 36.4 (standard deviation [SD]: 14.0). … showing excellent agreement between observers.* |
| Main results | 16 | (*a*) Give unadjusted estimates and, if applicable, confounder-adjusted estimates and their precision (eg, 95% confidence interval). Make clear which confounders were adjusted for and why they were included | 3 | *According to DISCERN groupings, 34.4% of the YouTube videos were classified as very poor, 32.8% as poor, 11.5% as fair, 16.4% as good, and 4.9% as excellent* |
| | | (*b*) Report category boundaries when continuous variables were categorized | NA | |

| | | | | |
|---|---|---|---|---|
| | | (*c*) If relevant, consider translating estimates of relative risk into absolute risk for a meaningful time period | NA | |
| Other analyses | 17 | Report other analyses done—eg analyses of subgroups and interactions, and sensitivity analyses | 4 | There were no significant correlations between DISCERN scores and view count, time since upload, like ratio, or dislikes. |
| Key results | 18 | Summarise key results with reference to study objectives | 5 | The low total DISCERN scores indicated that the quality of information on meningioma treatment on YouTube is poor, and the video content requires improvement. |
| Limitations | 19 | Discuss limitations of the study, taking into account sources of potential bias or imprecision. Discuss both direction and magnitude of any potential bias | 6 | *This study had a number of limitations…* |
| Interpretation | 20 | Give a cautious overall interpretation of results considering objectives, limitations, multiplicity of analyses, results from similar studies, and other relevant evidence | 7 | *Because there are only a few reliable and unbiased YouTube videos on meningioma treatment, neurosurgical societies should be encouraged to provide high-quality patient education videos, posted on their websites, but then disseminated through their own channels on popular websites frequently viewed by patients* |
| Generalisability | 21 | Discuss the generalisability (external validity) of the study results | 6 | *Only English-language videos from 1 video-sharing site (YouTube) were considered.* |
| **Other information** | | | | |
| Funding | 22 | Give the source of funding and the role of the funders for the present study and, if applicable, for the original study on which the present article is based | NA | |

Table S132. Quality Evaluation for Included Studies Using STROBE

56.STROBE Statement—checklist of items that should be included in reports of observational studies

| | Item No. | Recommendation | Page No. | Relevant text from manuscript |
|---|---|---|---|---|
| **Title and abstract** | 1 | (*a*) Indicate the study's design with a commonly used term in the title or the abstract | 1 | Cross-sectional search |
| | | (*b*) Provide in the abstract an informative and balanced summary of what was done and what was found | 1 | Methods and Results |
| **Introduction** | | | | |
| Background/rationale | 2 | Explain the scientific background and rationale for the investigation being reported | 2 | *Black Americans with low electronic health literacy have been found to have high perceived trust in the health information available on YouTube.20 The quality of general PrCA screening information available on YouTube is low, and the content is potentially misleading.* |
| Objectives | 3 | State specific objectives, including any prespecified hypotheses | 2 | evaluate the information regarding PrCA screening for Black men available on YouTube videos. |
| **Methods** | | | | |
| Study design | 4 | Present key elements of study design early in the paper | 2 | Cross-sectional search |
| Setting | 5 | Describe the setting, locations, and relevant dates, including periods of recruitment, exposure, follow-up, and data collection | 2 | Internet searches were performed for YouTube videos using the term "prostate cancer screening in Black men." |
| Participants | 6 | (*a*) *Cohort study*—Give the eligibility criteria, and the sources and methods of selection of participants. Describe methods of follow-up<br>*Case-control study*—Give the eligibility criteria, and the sources and methods of case ascertainment and control selection. Give the rationale for the choice of cases and controls<br>*Cross-sectional study*—Give the eligibility criteria, and the sources and methods of selection of participants | 2 | A total of 50 videos were identified, meeting the following criteria: over 1000 views, between 1 and 10 minutes long, appears on the first search result screen. Videos shorter than 1 minute or longer than 10 minutes were excluded |

| | | | | |
|---|---|---|---|---|
| | | (*b*) *Cohort study*—For matched studies, give matching criteria and number of exposed and unexposed<br>*Case-control study*—For matched studies, give matching criteria and the number of controls per case | NA | |
| Variables | 7 | Clearly define all outcomes, exposures, predictors, potential confounders, and effect modifiers. Give diagnostic criteria, if applicable | 2 | *Study-specific constructs included the video's perceived target audience (all men or Black men), viewers' engagement metrics (number of views, comments, thumbs up or down), the presence or absence of commercial bias (ie, advertising products or services), and whether the videos addressed disparities (yes, no, or unclear).* |
| Data sources/ measurement | 8* | For each variable of interest, give sources of data and details of methods of assessment (measurement). Describe comparability of assessment methods if there is more than one group | *2* | *Videos were from YouTube*<br>*Four raters from the Medical University of South Carolina in Charleston, South Carolina, independently assessed the videos* |
| Bias | 9 | Describe any efforts to address potential sources of bias | 2 | *Inter-rater reliability testing with a 2-way mixed model was performed for absolute agreement between the 4 raters for the 50 videos.* |
| Study size | 10 | Explain how the study size was arrived at | 2 | 50 videos |

| | | | | |
|---|---|---|---|---|
| Quantitative variables | 11 | Explain how quantitative variables were handled in the analyses. If applicable, describe which groupings were chosen and why | 2 | *DISCERN, PEMAT, viewers' engagement metrics (number of views, comments, thumbs up or down)* |
| Statistical methods | 12 | (*a*) Describe all statistical methods, including those used to control for confounding | 2 | *Chi-squares and analysis of variance (ANOVA) were used to examine whether this specific point was related to video characteristics. Kendall's t* |

| | | | | |
|---|---|---|---|---|
| | | | | *correlations were used to evaluate whether PEMAT or DISCERN scores were associated with the length of the video and/or viewer engagement metrics. T-test was used to evaluate the relationship between video quality and target audience (Black men vs all men).* |
| | | (*b*) Describe any methods used to examine subgroups and interactions | 2 | *ANOVA was run to determine the effect of perceived presenter race (Black vs White) or commercial bias on the video ratings and on viewer engagement metrics.* |
| | | (*c*) Explain how missing data were addressed | NA | |
| | | (*d*) *Cohort study*—If applicable, explain how loss to follow-up was addressed<br>*Case-control study*—If applicable, explain how matching of cases and controls was addressed<br>*Cross-sectional study*—If applicable, describe analytical methods taking account of sampling strategy | NA | |
| | | (*e*) Describe any sensitivity analyses | NA | |
| **Results** | | | | |
| Participants | 13* | (a) Report numbers of individuals at each stage of study—eg numbers potentially eligible, examined for eligibility, confirmed eligible, included in the study, completing follow-up, and analysed | 3 | We scanned through 190 videos to identify 50 that met the inclusion criteria. There were 6 videos excluded for being under 1 minute and 18 excluded for being longer than 10 minutes. An additional 116 videos were excluded for having fewer than 1000 views. |
| | | (b) Give reasons for non-participation at each stage | NA | |
| | | (c) Consider use of a flow diagram | 3 | Figure1 |
| Descriptive data | 14* | (a) Give characteristics of study participants (eg demographic, clinical, social) and information on exposures and potential confounders | 3 | We found 88%and 78%overlap between the top 50 videos using 3 different user profiles. The |

| | | | | |
|---|---|---|---|---|
| | | | | vast majority (96%) of videos included in the analysis came from hospitals, clinics, doctors, or professional and health organizations. |
| | | (b) Indicate number of participants with missing data for each variable of interest | | |
| | | (c) *Cohort study*—Summarise follow-up time (eg, average and total amount) | | |
| Outcome data | 15* | *Cohort study*—Report numbers of outcome events or summary measures over time | | |
| | | *Case-control study*—Report numbers in each exposure category, or summary measures of exposure | | |
| | | *Cross-sectional study*—Report numbers of outcome events or summary measures | *3-4* | *Shared decision making was not related to viewer engagement metrics, … The date of changes in AUA recommendations did not affect the video scores.* |
| Main results | 16 | (*a*) Give unadjusted estimates and, if applicable, confounder-adjusted estimates and their precision (eg, 95% confidence interval). Make clear which confounders were adjusted for and why they were included | 3 | *The average DISCERN (quality of information for decision making) score was 40.24 (12.49 SD) and ranged from 21.50 to 70.00 out of 80, with 16.0% of the videos above the quality threshold of 54.4.* |
| | | (*b*) Report category boundaries when continuous variables were categorized | NA | |
| | | (*c*) If relevant, consider translating estimates of relative risk into absolute risk for a meaningful time period | NA | |
| Other analyses | 17 | Report other analyses done—eg analyses of subgroups and interactions, and sensitivity analyses | 4 | DISCERN and PEMAT averages differed depending on presenter perceived race, with videos presented by a Black person scoring lower on average (33.59 and 8.79) than White |
| Key results | 18 | Summarise key results with reference to study objectives | 4 | *Our findings are consistent with previous studies reporting the overall poor informational quality* |

| | | | | |
|---|---|---|---|---|
| | | | | *of YouTube videos about prostate cancer.* |
| Limitations | 19 | Discuss limitations of the study, taking into account sources of potential bias or imprecision. Discuss both direction and magnitude of any potential bias | 5 | *One limitation of this study is that only YouTube was examined, …* |
| Interpretation | 20 | Give a cautious overall interpretation of results considering objectives, limitations, multiplicity of analyses, results from similar studies, and other relevant evidence | 5 | *Medical organizations should capitalize on the reach of these platforms by producing high-quality videos and implementing strategies to increase traffic to their sites so that the videos have far reach.* |
| Generalisability | 21 | Discuss the generalisability (external validity) of the study results | 5 | *only YouTube was examined, and other websites or social media outlets were not evaluated.* |
| **Other information** | | | | |
| Funding | 22 | Give the source of funding and the role of the funders for the present study and, if applicable, for the original study on which the present article is based | NA | |

Table S133. Quality Evaluation for Included Studies Using STROBE

57.STROBE Statement—checklist of items that should be included in reports of observational studies

| | Item No. | Recommendation | Page No. | Relevant text from manuscript |
|---|---|---|---|---|
| **Title and abstract** | 1 | (*a*) Indicate the study's design with a commonly used term in the title or the abstract | 1 | *Observational study* |
| | | (*b*) Provide in the abstract an informative and balanced summary of what was done and what was found | 1 | Methods and Results |
| **Introduction** | | | | |
| Background/rationale | 2 | Explain the scientific background and rationale for the investigation being reported | 2 | *Although many health-related videos on YouTube are deemed educationally useful and high quality, some studies reveal that this is not always the case and some have commercial content designed to sell products or services, which may have serious implications for consumer attitudes and medical decision-making* |
| Objectives | 3 | State specific objectives, including any prespecified hypotheses | 1 | *Objective: The aim of this observational study was to assess the quality of YouTube videos, accessible for any patient, about exercises after BC surgery.* |
| **Methods** | | | | |
| Study design | 4 | Present key elements of study design early in the paper | 2 | Cross-sectional search |
| Setting | 5 | Describe the setting, locations, and relevant dates, including periods of recruitment, exposure, follow-up, and data collection | 2 | On March 31, 2019, a search was conducted on http://www.youtube.com using the following search term: "Exercises after breast cancer surgery." |
| Participants | 6 | (*a*) *Cohort study*—Give the eligibility criteria, and the sources and methods of selection of participants. Describe methods of follow-up | 2 | Exclusion criteria were non-English language, less than 5,000 views, duplicated videos, and/or related with advertisements. Video URLs were used to identify videos |

| | | | | |
|---|---|---|---|---|
| | | *Case-control study*—Give the eligibility criteria, and the sources and methods of case ascertainment and control selection. Give the rationale for the choice of cases and controls<br><br>*Cross-sectional study*—Give the eligibility criteria, and the sources and methods of selection of participants | | for subsequent screening and coding. Finally, 51 videos were assigned to two different examiners who viewed, analyzed, and evaluated them independently over a period of 5 weeks (Fig. 1). |
| | | (*b*) *Cohort study*—For matched studies, give matching criteria and number of exposed and unexposed<br><br>*Case-control study*—For matched studies, give matching criteria and the number of controls per case | | |
| Variables | 7 | Clearly define all outcomes, exposures, predictors, potential confounders, and effect modifiers. Give diagnostic criteria, if applicable | 3 | *Descriptive characteristics of each video (view counts, likes, dislikes, origin, days online, author, and duration) were collected. Video popularity was assessed using the Video Power Index (VPI) [(like count/(dislike count + like count)) Å~ 100)] [18, 24, 25] and view ratio (views count/days online) [26].* |
| Data sources/ measurement | 8* | For each variable of interest, give sources of data and details of methods of assessment (measurement). Describe comparability of assessment methods if there is more than one group | *3* | *Videos were from YouTube* |
| Bias | 9 | Describe any efforts to address potential sources of bias | 5 | *intraclass correlation coefficient (ICC) analysis was conducted to gauge inter-examiner concordance*<br><br>*The average obtained through both independent assessments was used for each video. DISCERN, HONcode, and GQS scores of both observers were averaged to calculate the mean scores for each video.* |
| Study size | 10 | Explain how the study size was arrived at | 3 | 51 videos |

| | | | | |
|---|---|---|---|---|
| Quantitative variables | 11 | Explain how quantitative variables were handled in the analyses. If applicable, describe which groupings were chosen and why | 3 | *view counts, likes, dislikes, Video popularity and view ratio, DISCERN instrument (Quality Criteria for Consumer Health Information) [27] and the Global Quality Scale (GQS)* |
| Statistical methods | 12 | (*a*) Describe all statistical methods, including those used to control for confounding | 4-5 | The first one is machine learning techniques.<br><br>The second analysis was a statistical one using a t test and the Wilcoxon test, illustrating how significant the differences between both classes are from the perspective of each variable. |
| | | (*b*) Describe any methods used to examine subgroups and interactions | 5 | The statistical analysis also shows the relevance of each variable regarding the grouping of the samples into C1 and C2, in each of the three cases. |
| | | (*c*) Explain how missing data were addressed | NA | |
| | | (*d*) *Cohort study*—If applicable, explain how loss to follow-up was addressed<br><br>*Case-control study*—If applicable, explain how matching of cases and controls was addressed<br><br>*Cross-sectional study*—If applicable, describe analytical methods taking account of sampling strategy | NA | |
| | | (*e*) Describe any sensitivity analyses | NA | |
| **Results** | | | | |
| Participants | 13* | (a) Report numbers of individuals at each stage of study—eg numbers potentially eligible, examined for eligibility, confirmed eligible, included in the study, completing follow-up, and analysed | 3 | Based on Fig.1 |
| | | (b) Give reasons for non-participation at each stage | NA | |
| | | (c) Consider use of a flow diagram | 3 | Fig. 1 |
| Descriptive data | 14* | (a) Give characteristics of study participants (eg demographic, clinical, social) and information on exposures and potential confounders | 5 | Most of the videos (33%) were produced by health institutions, … data examined |

| | | | | |
|---|---|---|---|---|
| | | | | by descriptive statistics for the videos included. |
| | | (b) Indicate number of participants with missing data for each variable of interest | NA | |
| | | (c) *Cohort study*—Summarise follow-up time (eg, average and total amount) | NA | |
| Outcome data | 15* | *Cohort study*—Report numbers of outcome events or summary measures over time | | |
| | | *Case-control study*—Report numbers in each exposure category, or summary measures of exposure | | |
| | | *Cross-sectional study*—Report numbers of outcome events or summary measures | *5* | *Considering average* *results, the mean DISCERN score was 50.97 …* |
| Main results | 16 | (*a*) Give unadjusted estimates and, if applicable, confounder-adjusted estimates and their precision (eg, 95% confidence interval). Make clear which confounders were adjusted for and why they were included | 5 | *According to the mean DISCERN scores of both observers, the quality of the videos was deemed very poor in 2% of the cases, poor in 10%, average in 22%, high in 59%, and very high in 8%* |
| | | (*b*) Report category boundaries when continuous variables were categorized | NA | |
| | | (*c*) If relevant, consider translating estimates of relative risk into absolute risk for a meaningful time period | NA | |
| Other analyses | 17 | Report other analyses done—eg analyses of subgroups and interactions, and sensitivity analyses | 8 | how significant the differences between both classes are from the perspective of each variable. |
| Key results | 18 | Summarise key results with reference to study objectives | 8 | *although variable in source and content, the quality of the information offered on YouTube about recommended postoperative shoulder exercises for BC patients is high.* |
| Limitations | 19 | Discuss limitations of the study, taking into account sources of potential bias or imprecision. Discuss both direction and magnitude of any potential bias | 10 | *Bias …* |

| | | | | |
|---|---|---|---|---|
| Interpretation | 20 | Give a cautious overall interpretation of results considering objectives, limitations, multiplicity of analyses, results from similar studies, and other relevant evidence | 10 | *A verification and validation process of the information available on the web is considered necessary, as well as educational programs to facilitate people's access to the most reliable information.* |
| Generalisability | 21 | Discuss the generalisability (external validity) of the study results | 10 | *our search was limited to the first 150 videos. As other studies have previously explained, our methodology was designed to replicate the average patient's search attempt* |
| **Other information** | | | | |
| Funding | 22 | Give the source of funding and the role of the funders for the present study and, if applicable, for the original study on which the present article is based | 10 | *This study did not receive any specific grant from funding agencies in the public, commercial, or not-for-profit sectors.* |

Table S134. Quality Evaluation for Included Studies Using STROBE

58.STROBE Statement—checklist of items that should be included in reports of observational studies

| | Item No. | Recommendation | Page No. | Relevant text from manuscript |
|---|---|---|---|---|
| **Title and abstract** | 1 | (*a*) Indicate the study's design with a commonly used term in the title or the abstract | 1 | Cross-sectional quality assessment |
| | | (*b*) Provide in the abstract an informative and balanced summary of what was done and what was found | 1 | "*YouTube was searched in May 2022 for German … a mediocre quality and actionability, and a low reliability.*" |
| **Introduction** | | | | |
| Background/rationale | 2 | Explain the scientific background and rationale for the investigation being reported | 2 | *YouTube is an open access video-sharing platform, which is increasingly … also challenges as the quality of unfiltered information posted can be of low scientific quality* |
| Objectives | 3 | State specific objectives, including any prespecified hypotheses | 2 | *identify YouTube videos on SCS and to assess the quality, reliability, usability, and understandability.* |
| **Methods** | | | | |
| Study design | 4 | Present key elements of study design early in the paper | 2 | Cross-sectional search |
| Setting | 5 | Describe the setting, locations, and relevant dates, including periods of recruitment, exposure, follow-up, and data collection | 2 | *A video search on YouTube was conducted in May 2022, using German SCS-related keywords* |
| Participants | 6 | (*a*) *Cohort study*—Give the eligibility criteria, and the sources and methods of selection of participants. Describe methods of follow-up<br>*Case-control study*—Give the eligibility criteria, and the sources and methods of case ascertainment and control selection. Give the rationale for the choice of cases and controls<br>*Cross-sectional study*—Give the eligibility criteria, and the sources and methods of selection of participants | 2 | Eligibility criteria<br>"*Videos had to meet the following inclusion criteria to be eligible for evaluation: contain information referring …*" |

| | | | | |
|---|---|---|---|---|
| | | (*b*) *Cohort study*—For matched studies, give matching criteria and number of exposed and unexposed<br><br>*Case-control study*—For matched studies, give matching criteria and the number of controls per case | NA | |
| Variables | 7 | Clearly define all outcomes, exposures, predictors, potential confounders, and effect modifiers. Give diagnostic criteria, if applicable | 2 | *The available baseline information (URL, title, name of the provider, length, and year of upload) of each selected video was documented. Additionally, the number of views, likes, and dislikes was extracted. DISCERN tool, PEMAT-A/V, (JAMA) benchmark criteria* |
| Data sources/ measurement | 8* | For each variable of interest, give sources of data and details of methods of assessment (measurement). Describe comparability of assessment methods if there is more than one group | 2 | *Videos were from YouTube*<br><br>*Two reviewers (LR, AM) independently assessed the videos' quality of information, reliability, and understandability.* |
| Bias | 9 | Describe any efforts to address potential sources of bias | 2 | *All search results were screened for duplicates, and the predefined eligibility criteria were applied. Two reviewers (LR, AM) independently assessed …* |
| Study size | 10 | Explain how the study size was arrived at | 3 | 38 videos |

| | | | | |
|---|---|---|---|---|
| Quantitative variables | 11 | Explain how quantitative variables were handled in the analyses. If applicable, describe which groupings were chosen and why | 2 | *the number of views, likes, and dislikes was extracted. DISCERN tool, PEMAT-A/V, (JAMA) benchmark criteria* |
| Statistical methods | 12 | (*a*) Describe all statistical methods, including those used to control for confounding | 3 | *Descriptive analyses included mean (SD) or median and interquartile ranges (IQR). Subgroup differences were explored using the Kruskal–Wallis test. The relationship between the individual items of* |

| | | | | |
|---|---|---|---|---|
| | | | | *the tests was examined using Spearman's correlation.* |
| | | (*b*) Describe any methods used to examine subgroups and interactions | 3 | *Subgroup differences were explored using the Kruskal–Wallis test.* |
| | | (*c*) Explain how missing data were addressed | NA | |
| | | (*d*) *Cohort study*—If applicable, explain how loss to follow-up was addressed<br>*Case-control study*—If applicable, explain how matching of cases and controls was addressed<br>*Cross-sectional study*—If applicable, describe analytical methods taking account of sampling strategy | NA | |
| | | (*e*) Describe any sensitivity analyses | NA | |
| **Results** | | | | |
| Participants | 13* | (a) Report numbers of individuals at each stage of study—eg numbers potentially eligible, examined for eligibility, confirmed eligible, included in the study, completing follow-up, and analysed | 3 | *Our search identified 464 videos. Two reviewers (TS, LR) screened the videos for duplicates and checked them for compliance with the pre-defined eligibility criteria. Finally, 38 videos were considered for assessment* |
| | | (b) Give reasons for non-participation at each stage | NA | |
| | | (c) Consider use of a flow diagram | 3 | Fig.1 |
| Descriptive data | 14* | (a) Give characteristics of study participants (eg demographic, clinical, social) and information on exposures and potential confounders | 3-4 | "*Most videos were provided by health professionals (39.5%, 15/38), … Therefore, long videos were also included in the search.*" |
| | | (b) Indicate number of participants with missing data for each variable of interest | NA | |
| | | (c) *Cohort study*—Summarise follow-up time (eg, average and total amount) | NA | |
| Outcome data | 15* | *Cohort study*—Report numbers of outcome events or summary measures over time | | |
| | | *Case-control study*—Report numbers in each exposure category, or summary measures of exposure | | |

| | | | | |
|---|---|---|---|---|
| | | *Cross-sectional study*—Report numbers of outcome events or summary measures | *5* | Quality: DISCERN and GQS Results<br><br>Understandability and Actionability: PEMAT Results<br><br>Accuracy, Utility, and Reliability: JAMA Results<br><br>Harms and Benefit |
| Main results | 16 | (*a*) Give unadjusted estimates and, if applicable, confounder-adjusted estimates and their precision (eg, 95% confidence interval). Make clear which confounders were adjusted for and why they were included | 5 | *Videos rated as useful showed a significantly better quality in comparison to those rated as neutral (DISCERN: p = 0,001; GQS: p = 0,002) or harmful (DISCERN, GQS: p = 0,001).* |
| | | (*b*) Report category boundaries when continuous variables were categorized | NA | |
| | | (*c*) If relevant, consider translating estimates of relative risk into absolute risk for a meaningful time period | NA | |
| Other analyses | 17 | Report other analyses done—eg analyses of subgroups and interactions, and sensitivity analyses | 5 | Correlation Analysis |
| Key results | 18 | Summarise key results with reference to study objectives | 6 | *Compared to the other criteria, the videos' usability was rated best, particularly for using everyday language* |
| Limitations | 19 | Discuss limitations of the study, taking into account sources of potential bias or imprecision. Discuss both direction and magnitude of any potential bias | 7 | *It should be acknowledged that the videos evaluated had different formats….* |
| Interpretation | 20 | Give a cautious overall interpretation of results considering objectives, limitations, multiplicity of analyses, results from similar studies, and other relevant evidence | 7 | *An improvement of freely available informational videos on SCS, especially with regard to the reliability criteria, is urgently needed.* |
| Generalisability | 21 | Discuss the generalisability (external validity) of the study results | 7 | *videos that still contained correct information at the time they were created may now be outdated. Therefore, the* |

| | | | | |
|---|---|---|---|---|
| | | | | *comparability between the videos is limited.* |
| **Other information** | | | | |
| Funding | 22 | Give the source of funding and the role of the funders for the present study and, if applicable, for the original study on which the present article is based | 7 | *This study has been funded by the German Skin Cancer Council* |

Table S135. Quality Evaluation for Included Studies Using STROBE

59.STROBE Statement—checklist of items that should be included in reports of observational studies

| | Item No. | Recommendation | Page No. | Relevant text from manuscript |
|---|---|---|---|---|
| **Title and abstract** | 1 | (*a*) Indicate the study's design with a commonly used term in the title or the abstract | 1 | Qualitative assessment |
| | | (*b*) Provide in the abstract an informative and balanced summary of what was done and what was found | 1 | *"An independent search for surgical therapy or radiotherapy of PCa on YouTube was performed … less misinformation and were judged to be of higher quality."* |
| **Introduction** | | | | |
| Background/rationale | 2 | Explain the scientific background and rationale for the investigation being reported | 1 | *In times of limited resources and social distancing [12], new healthcare services, such as consultations via telephone or video have been introduced [13]. Due to the unprecedented disruption of health care services with postponed treatments [14] online sources such as YouTube gain in importance* |
| Objectives | 3 | State specific objectives, including any prespecified hypotheses | 1 | *The specific objective of the following study was to perform a systematic and comparative assessment of available videos guiding patients on their choice for the optimal treatment of their localized PCa* |
| **Methods** | | | | |
| Study design | 4 | Present key elements of study design early in the paper | 2 | Cross-sectional search |
| Setting | 5 | Describe the setting, locations, and relevant dates, including periods of recruitment, exposure, follow-up, and data collection | 2 | *An independent search for surgical therapy or radiotherapy (RT) of PCa on YouTube was performed in March 2021, in English, by four of the authors* |

| | | | | |
|---|---|---|---|---|
| Participants | 6 | (*a*) *Cohort study*—Give the eligibility criteria, and the sources and methods of selection of participants. Describe methods of follow-up<br><br>*Case-control study*—Give the eligibility criteria, and the sources and methods of case ascertainment and control selection. Give the rationale for the choice of cases and controls<br><br>*Cross-sectional study*—Give the eligibility criteria, and the sources and methods of selection of participants | 2 | *The video search was*<br><br>*conducted using associated keywords (see Supplementary text box). Only videos with an English audio track were included, and videos solely targeting healthcare professionals (i.e., surgical techniques, instructional explanation of device use) were excluded* |
| | | (*b*) *Cohort study*—For matched studies, give matching criteria and number of exposed and unexposed<br><br>*Case-control study*—For matched studies, give matching criteria and the number of controls per case | NA | |
| Variables | 7 | Clearly define all outcomes, exposures, predictors, potential confounders, and effect modifiers. Give diagnostic criteria, if applicable | 2 | *Basic data (video upload date, length, number of views, quality of audio and video, disclosure) was assessed for every video. Further parameters documented were: type of video provider (consumer/patient; healthcare [doctor, clinic, hospital, university]; industry; news media; society/organization [foundation, governmental, academic journal]; unclear); primary and secondary topics of the video (overall procedure; surgery indications; benefits; risks/side effects; technical aspects); degree of misinformation compared to currently available evidence (no; very little; moderate; high; extreme); and depiction of real intervention (yes/no).*<br><br>*DISCERN, PEMAT tool* |

| | | | | |
|---|---|---|---|---|
| Data sources/ measurement | 8* | For each variable of interest, give sources of data and details of methods of assessment (measurement). Describe comparability of assessment methods if there is more than one group | *2* | *Videos were from YouTube* |
| Bias | 9 | Describe any efforts to address potential sources of bias | 2 | *Two of the authors were board-certified urologists (Fellow of the European Board of Urology), one author was a board-certified radiation oncologist and one a senior resident.* |
| Study size | 10 | Explain how the study size was arrived at | 2 | 80 videos |

| | | | | |
|---|---|---|---|---|
| Quantitative variables | 11 | Explain how quantitative variables were handled in the analyses. If applicable, describe which groupings were chosen and why | 2 | *DISCERN, PEMAT tool* |
| Statistical methods | 12 | (*a*) Describe all statistical methods, including those used to control for confounding | 2 | *Descriptive analysis and Mann–Whitney U tests* |
| | | (*b*) Describe any methods used to examine subgroups and interactions | 3 | Table 3 |
| | | (*c*) Explain how missing data were addressed | NA | |
| | | (*d*) *Cohort study*—If applicable, explain how loss to follow-up was addressed<br>*Case-control study*—If applicable, explain how matching of cases and controls was addressed<br>*Cross-sectional study*—If applicable, describe analytical methods taking account of sampling strategy | NA | |
| | | (*e*) Describe any sensitivity analyses | NA | |
| **Results** | | | | |
| Participants | 13* | (a) Report numbers of individuals at each stage of study—eg numbers potentially eligible, examined for eligibility, confirmed eligible, included in the study, completing follow-up, and analysed | 2 | Results paragraph 2 |
| | | (b) Give reasons for non-participation at each stage | NA | |
| | | (c) Consider use of a flow diagram | NA | |
| Descriptive data | 14* | (a) Give characteristics of study participants (eg demographic, clinical, social) and information on exposures and potential confounders | 2 | *Most of the videos concerning radiotherapy (60%) and surgery (48%) … much less misinformation than surgery* |

| | | | | |
|---|---|---|---|---|
| | | | | *videos as demonstrated in Figure 2.* |
| | | (b) Indicate number of participants with missing data for each variable of interest | NA | |
| | | (c) *Cohort study*—Summarise follow-up time (eg, average and total amount) | NA | |
| Outcome data | 15* | *Cohort study*—Report numbers of outcome events or summary measures over time | *3* | *"The median overall quality of the videos, according to DISCERN … the radiotherapy videos than for the surgery videos (U = 65, p = 0.018)."* |
| | | *Case-control study*—Report numbers in each exposure category, or summary measures of exposure | *NA* | |
| | | *Cross-sectional study*—Report numbers of outcome events or summary measures | *NA* | |
| Main results | 16 | (*a*) Give unadjusted estimates and, if applicable, confounder-adjusted estimates and their precision (eg, 95% confidence interval). Make clear which confounders were adjusted for and why they were included | 3 | *The median overall quality of the videos, according to DISCERN, was found to be low for surgery videos (2 out of 5 points for question 16; Table 2), while radiotherapy results reached a moderate quality* |
| | | (*b*) Report category boundaries when continuous variables were categorized | NA | |
| | | (*c*) If relevant, consider translating estimates of relative risk into absolute risk for a meaningful time period | NA | |

| | | | | |
|---|---|---|---|---|
| Other analyses | 17 | Report other analyses done—eg analyses of subgroups and interactions, and sensitivity analyses | NA | |

| | | | | |
|---|---|---|---|---|
| Key results | 18 | Summarise key results with reference to study objectives | 6 | *Overall, the RT videos contained less misinformation (Figure 2) and were judged to be of higher quality than the surgery videos* |
| Limitations | 19 | Discuss limitations of the study, taking into account sources of potential bias or imprecision. Discuss both direction and magnitude of any potential bias | 7 | *Our present study had some limitations. …* |
| Interpretation | 20 | Give a cautious overall interpretation of results considering objectives, limitations, multiplicity of analyses, results from similar studies, and other relevant evidence | 7 | *The majority of the provided videos about surgery and radiotherapy of localized PCa* |

| | | | | |
|---|---|---|---|---|
| | | | | *offer insufficient quality of content and are potentially subject to commercial bias, without reporting their possible conflict of interest. Thus, most of the available videos on YouTube informing PCA patients about possible treatment methods are not suited for a balanced patient education or as a basis for the patient's decisio* |
| Generalisability | 21 | Discuss the generalisability (external validity) of the study results | 7 | *First, only English videoswere included in our assessment. Second, video analyses always faced significant subjectivity* |
| **Other information** | | | | |
| Funding | 22 | Give the source of funding and the role of the funders for the present study and, if applicable, for the original study on which the present article is based | 8 | *Funding: This research received no external funding.* |

Table S136. Quality Evaluation for Included Studies Using STROBE

60.STROBE Statement—checklist of items that should be included in reports of observational studies

| | Item No. | Recommendation | Page No. | Relevant text from manuscript |
|---|---|---|---|---|
| **Title and abstract** | 1 | (*a*) Indicate the study's design with a commonly used term in the title or the abstract | 1 | *observational, retrospective, cross-sectional, time-limited study* |
| | | (*b*) Provide in the abstract an informative and balanced summary of what was done and what was found | 1 | *"The information from the videos was extracted through an API search tool, … a value that reflects a good reliability of videos from these users."* |
| **Introduction** | | | | |
| Background/rationale | 2 | Explain the scientific background and rationale for the investigation being reported | 2 | *Concerns about diet and food intake have increased in recent years to the current situation where 60% of the population admit to being worried about long-term risks of the food they eat there are no previous studies that focus on the role of the real food movement and its association with cancer patients on YouTube* |
| Objectives | 3 | State specific objectives, including any prespecified hypotheses | 2 | *analyze the quality and validity of the existing videos on YouTube that relate the consumption of "real food" and cancer….* |
| **Methods** | | | | |
| Study design | 4 | Present key elements of study design early in the paper | 3 | *observational, retrospective, cross-sectional* |
| Setting | 5 | Describe the setting, locations, and relevant dates, including periods of recruitment, exposure, follow-up, and data collection | 3 | *The data extraction system used in the present study has been through an API (Application Programming Interface) search tool* |

| | | | | |
|---|---|---|---|---|
| Participants | 6 | (*a*) *Cohort study*—Give the eligibility criteria, and the sources and methods of selection of participants. Describe methods of follow-up<br>*Case-control study*—Give the eligibility criteria, and the sources and methods of case ascertainment and control selection. Give the rationale for the choice of cases and controls<br>*Cross-sectional study*—Give the eligibility criteria, and the sources and methods of selection of participants | 3 | *the criteria to select the videos in YouTube were: (i) the keywords "real food", "realfood" and "cancer" and the hashtags #realfood and #cancer were selected. (ii) videos in English. (iii) videos available on 1 December 2022. The exclusion criteria were: (i) non-English videos, (ii) advertisements, (iii) videos not related to real food and cancer in humans.* |
| | | (*b*) *Cohort study*—For matched studies, give matching criteria and number of exposed and unexposed<br>*Case-control study*—For matched studies, give matching criteria and the number of controls per case | NA | |
| Variables | 7 | Clearly define all outcomes, exposures, predictors, potential confounders, and effect modifiers. Give diagnostic criteria, if applicable | 3 | *The following data was retrieved: upload date, number of views, number of likes.*<br>*Moreover, two indexes were calculated to compare the videos with each other; (i) the View Ratio (number of views/days from the upload to the moment of the data collection), (ii) the Viewers interaction (number of likes + comments/number of views)* |
| Data sources/ measurement | 8* | For each variable of interest, give sources of data and details of methods of assessment (measurement). Describe comparability of assessment methods if there is more than one group | *3* | *Videos were from YouTube*<br>*This analysis was conducted by two researchers (S.S.-F. and M.d.C.L.-E.) and then corroborated by a third one (P.J.J.H.).* |
| Bias | 9 | Describe any efforts to address potential sources of bias | 3 | *The videos were reviewed by a group of experts including physicians, nurse, and a pharmaceutic-nutritionist, so that any differences in approach and focus were* |

| | | | | |
|---|---|---|---|---|
| | | | | *always discussed and resolved with full agreement* |
| Study size | 10 | Explain how the study size was arrived at | 4 | 72 videos |
| Quantitative variables | 11 | Explain how quantitative variables were handled in the analyses. If applicable, describe which groupings were chosen and why | 4 | *GQS score and DISCERN scale* |
| Statistical methods | 12 | (*a*) Describe all statistical methods, including those used to control for confounding | 4 | *Descriptive statistics are presented, medians were used for quantitative variables and proportions were used for qualitative variables. Spearman's nonparametric correlation coefficient (Spearman's Rho) was used for correlational analysis. Mann Whitney's U was used to compare the numerical variables. Multivariate linear regression was used to characterize relationships between video characteristics, upload source, content category, reliability (DISCERN), and educational quality (GQS).* |
| | | (*b*) Describe any methods used to examine subgroups and interactions | 5 | Compare HRU and non-HRU |
| | | (*c*) Explain how missing data were addressed | NA | |
| | | (*d*) *Cohort study*—If applicable, explain how loss to follow-up was addressed<br>*Case-control study*—If applicable, explain how matching of cases and controls was addressed<br>*Cross-sectional study*—If applicable, describe analytical methods taking account of sampling strategy | NA | |
| | | (*e*) Describe any sensitivity analyses | NA | |
| **Results** | | | | |
| Participants | 13* | (a) Report numbers of individuals at each stage of study—eg numbers potentially eligible, examined for eligibility, confirmed eligible, included in the study, completing follow-up, and analysed | 3 | *The analysis of the data obtained was performed … the subject matter required in the study* |

| | | | | |
|---|---|---|---|---|
| | | (b) Give reasons for non-participation at each stage | NA | |
| | | (c) Consider use of a flow diagram | 4 | Figure 1 |
| Descriptive data | 14* | (a) Give characteristics of study participants (eg demographic, clinical, social) and information on exposures and potential confounders | 5 | *Of the 72 videos selected after review by the researchers, the total number of views was found to be 44,682,055. Each video was viewed a total of 620,584.09 (CI95%: 177,449.33– 1,068,942.71) times (Table 1). Regarding the remaining totals, it was found that 32,956 comments were obtained, with 708,351 likes and only 191 dislikes.* |
| | | (b) Indicate number of participants with missing data for each variable of interest | NA | |
| | | (c) *Cohort study*—Summarise follow-up time (eg, average and total amount) | NA | |
| Outcome data | 15* | *Cohort study*—Report numbers of outcome events or summary measures over time | | |
| | | *Case-control study*—Report numbers in each exposure category, or summary measures of exposure | | |
| | | *Cross-sectional study*—Report numbers of outcome events or summary measures | *5-6* | *3.1 Description of the Sample*<br>*3.2. Analysis According to the Type of Videos and Users* |
| Main results | 16 | (*a*) Give unadjusted estimates and, if applicable, confounder-adjusted estimates and their precision (eg, 95% confidence interval). Make clear which confounders were adjusted for and why they were included | 5 | *The DISCERN value in the total number of videos viewed was 2.25 (0.88) points, indicating low reliability.* |
| | | (*b*) Report category boundaries when continuous variables were categorized | 6 | *3.2. Analysis According to the Type of Videos and Users: subtype DISCERN scale* |
| | | (*c*) If relevant, consider translating estimates of relative risk into absolute risk for a meaningful time period | NA | |

| | | | | |
|---|---|---|---|---|
| Other analyses | 17 | Report other analyses done—eg analyses of subgroups and interactions, and sensitivity analyses | 6-9 | *3.2. Analysis According to the Type of Videos and Users* |

| | | | | |
|---|---|---|---|---|
| | | | | *3.3. Correlation Analysis between Popularity Indexes, DISCERN and GQS* |
| | | | | |
| Key results | 18 | Summarise key results with reference to study objectives | 10 | *Most of the content shared in the videos came from users who did not identify themselves as healthcare professionals, and only a small portion was shared by these professionals. As seen in previous studies, the information they provide is more reliable, and they are also the ones who provide most external links to scientific evidence* |
| Limitations | 19 | Discuss limitations of the study, taking into account sources of potential bias or imprecision. Discuss both direction and magnitude of any potential bias | 11 | *On another note, the study has several limitations mainly …* |
| Interpretation | 20 | Give a cautious overall interpretation of results considering objectives, limitations, multiplicity of analyses, results from similar studies, and other relevant evidence | 12 | *Faced with this situation, where platforms and institutions do not act, it seems to be of utmost importance that healthcare professionals understand the need to be more present in social media from a professional point of view. In this way, they can become key figures in the creation and dissemination of reliable information from a scientific point of view, aimed at health care.* |
| Generalisability | 21 | Discuss the generalisability (external validity) of the study results | 11 | *that the content is constantly changing, which means that a cross-sectional design cannot be applied. Moreover, conducting the study in one single social network, YouTube, is a limitation that must be considered, since it is possible that the topics of real food and cancer can be addressed in other audiovisual social networks* |
| **Other information** | | | | |

| | | | | |
|---|---|---|---|---|
| Funding | 22 | Give the source of funding and the role of the funders for the present study and, if applicable, for the original study on which the present article is based | 12 | *Funding: This research was funded by Fundación Banco Santander and Fundación Alfonso X el Sabio, grant number* |

Table S137. Quality Evaluation for Included Studies Using STROBE

61.STROBE Statement—checklist of items that should be included in reports of observational studies

| | Item No. | Recommendation | Page No. | Relevant text from manuscript |
|---|---|---|---|---|
| **Title and abstract** | 1 | (*a*) Indicate the study's design with a commonly used term in the title or the abstract | 1 | Cross-sectional search |
| | | (*b*) Provide in the abstract an informative and balanced summary of what was done and what was found | 1 | Methods and Results |
| **Introduction** | | | | |
| Background/rationale | 2 | Explain the scientific background and rationale for the investigation being reported | 1 | *over 1 billion hours of videos watched daily.8 This phenomenon is even more accentuated during the last year due to the severe acute respiratory syndrome coronavirus 2 pandemic outbreak.9,10 Nevertheless, available information could result in misleading and scant.* |
| Objectives | 3 | State specific objectives, including any prespecified hypotheses | 1 | *evaluate the quality of information on TCA uploaded on YouTubeTM videos and how it changed during the last decades.* |
| **Methods** | | | | |
| Study design | 4 | Present key elements of study design early in the paper | 2 | Cross-sectional search |
| Setting | 5 | Describe the setting, locations, and relevant dates, including periods of recruitment, exposure, follow-up, and data collection | 2 | On April 10th 2021, from 9:00 a.m. to 6:00 p.m. UTC-4, YouTubeTM systematic research was performed. Using "Testicular cancer" as a keyword, the first 150 videos were collected. |
| Participants | 6 | (*a*) *Cohort study*—Give the eligibility criteria, and the sources and methods of selection of participants. Describe methods of follow-up<br>*Case-control study*—Give the eligibility criteria, and the sources and methods of case ascertainment and control selection. Give the rationale for the choice of cases and controls | 2 | The following exclusion criteria were applied (Fig. 1): (i) non-English language (n = 2); (ii) length >50 min (n = 3); (iii) duplicate videos (n = 23); |

| | | | | |
|---|---|---|---|---|
| | | *Cross-sectional study*—Give the eligibility criteria, and the sources and methods of selection of participants | | and (iv) no information reported on TCA (n = 1). |
| | | (*b*) *Cohort study*—For matched studies, give matching criteria and number of exposed and unexposed<br>*Case-control study*—For matched studies, give matching criteria and the number of controls per case | | |
| Variables | 7 | Clearly define all outcomes, exposures, predictors, potential confounders, and effect modifiers. Give diagnostic criteria, if applicable | 2 | *length (seconds), number of views, thumbs-up, thumbs-down, number of comments, number of videos with disabled comments, channel subscribers, persistence on YouTube TM (days), and video author (defined as a foundation, medical institutions, nonmedical channel, single individual). VPI, estimating video popularity, 13 was also determined.* |
| Data sources/ measurement | 8* | For each variable of interest, give sources of data and details of methods of assessment (measurement). Describe comparability of assessment methods if there is more than one group | *2* | *Videos were from YouTube*<br>*The quality of videos was assessed by two investigators* |
| Bias | 9 | Describe any efforts to address potential sources of bias | 2 | *To avoid research bias, any personal account was logged out and a proxy located in the United States (via a Virtual Private Network software) was set.* |
| Study size | 10 | Explain how the study size was arrived at | 3 | 121 videos |

| | | | | |
|---|---|---|---|---|
| Quantitative variables | 11 | Explain how quantitative variables were handled in the analyses. If applicable, describe which groupings were chosen and why | 2 | *length (seconds), number of views, thumbs-up, thumbs-down, number of comments, number of videos with disabled comments, channel subscribers* |

| | | | | |
|---|---|---|---|---|
| | | | | *PEMAT A/V14, DISCERN , 6-item Misinformation scale,* |
| Statistical methods | 12 | (*a*) Describe all statistical methods, including those used to control for confounding | 2 | *Descriptive statistics were presented as medians and IQRs for continuously coded variables or counts and percentages for categorically coded variables. Chi-squared test and Kruskal–Wallis tests examined the statistical significance in proportions' and medians' differences. Pearson's test was used to assess a potential correlation between the variables.* |
| | | (*b*) Describe any methods used to examine subgroups and interactions | 3 | a subgroup analysis was performed in YouTube TM videos mentioning treatment options. |
| | | (*c*) Explain how missing data were addressed | NA | |
| | | (*d*) *Cohort study*—If applicable, explain how loss to follow-up was addressed<br>*Case-control study*—If applicable, explain how matching of cases and controls was addressed<br>*Cross-sectional study*—If applicable, describe analytical methods taking account of sampling strategy | NA | |
| | | (*e*) Describe any sensitivity analyses | NA | |
| **Results** | | | | |
| Participants | 13* | (a) Report numbers of individuals at each stage of study—eg numbers potentially eligible, examined for eligibility, confirmed eligible, included in the study, completing follow-up, and analysed | 3 | *Of all 150 videos examined, 121 were selected for the analyses (Table 1).* |
| | | (b) Give reasons for non-participation at each stage | NA | |
| | | (c) Consider use of a flow diagram | 2 | Figure 1 |
| Descriptive data | 14* | (a) Give characteristics of study participants (eg demographic, clinical, social) and information on exposures and potential confounders | 3 | *The videos were stratified according to uploading year: 59 (48.8%) … were uploaded by single individual (P = 0.02), respectively.* |

| | | | | |
|---|---|---|---|---|
| | | (b) Indicate number of participants with missing data for each variable of interest | NA | |
| | | (c) *Cohort study*—Summarise follow-up time (eg, average and total amount) | NA | |
| Outcome data | 15* | *Cohort study*—Report numbers of outcome events or summary measures over time | | |
| | | *Case-control study*—Report numbers in each exposure category, or summary measures of exposure | | |
| | | *Cross-sectional study*—Report numbers of outcome events or summary measures | *3-4* | *Video quality assessment for overall videos* |
| Main results | 16 | (*a*) Give unadjusted estimates and, if applicable, confounder-adjusted estimates and their precision (eg, 95% confidence interval). Make clear which confounders were adjusted for and why they were included | 3 | *According to PEMAT A/V (Table 2), the overall PEMAT Understandability score was 60% (IQR 45.5–75) and the overall PEMAT Actionability score was 100% (IQR 66.7–100).* |
| | | (*b*) Report category boundaries when continuous variables were categorized | NA | |
| | | (*c*) If relevant, consider translating estimates of relative risk into absolute risk for a meaningful time period | NA | |

| | | | | |
|---|---|---|---|---|
| Other analyses | 17 | Report other analyses done—eg analyses of subgroups and interactions, and sensitivity analyses | 4 | Variable correlations |

| | | | | |
|---|---|---|---|---|
| Key results | 18 | Summarise key results with reference to study objectives | 5 | *Taken together, according to the PEMAT A/V tool, Misinformation scale, and DISCERN tool, today the quality of the information provided on YouTubeTM videos on TCA is low in the past as well in the present.* |
| Limitations | 19 | Discuss limitations of the study, taking into account sources of potential bias or imprecision. Discuss both direction and magnitude of any potential bias | 5 | *Our study is not devoid of limitations…* |
| Interpretation | 20 | Give a cautious overall interpretation of results considering objectives, limitations, multiplicity of analyses, results from similar studies, and other relevant evidence | 6 | *YouTubeTM cannot be recommended as a reliable source of information on TCA. The official medical institutions should standardize the medical* |

| | | | | |
|---|---|---|---|---|
| | | | | *contents: disseminating evidence-based information and sharing high-quality materials with users to promote health decision-making process and awareness of TCA.* |
| Generalisability | 21 | Discuss the generalisability (external validity) of the study results | 6 | *quality assessment videos were subjectively evaluated* |
| **Other information** | | | | |
| Funding | 22 | Give the source of funding and the role of the funders for the present study and, if applicable, for the original study on which the present article is based | NA | |

Table S138. Quality Evaluation for Included Studies Using STROBE

62.STROBE Statement—checklist of items that should be included in reports of observational studies

| | Item No. | Recommendation | Page No. | Relevant text from manuscript |
|---|---|---|---|---|
| **Title and abstract** | 1 | (*a*) Indicate the study's design with a commonly used term in the title or the abstract | 1 | Analysis of YouTube videos |
| | | (*b*) Provide in the abstract an informative and balanced summary of what was done and what was found | 1 | Methods and Results |
| **Introduction** | | | | |
| Background/rationale | 2 | Explain the scientific background and rationale for the investigation being reported | 1 | *An online documentary called Healing Cancer with Cannabis: The Rick Simpson Story [2] currently has over 150,000 views on YouTube* |
| Objectives | 3 | State specific objectives, including any prespecified hypotheses | 2 | *we sought to characterize the quality of information patients attain from popular YouTube videos concerning THC and skin cancer.* |
| **Methods** | | | | |
| Study design | 4 | Present key elements of study design early in the paper | 2 | Cross-sectional search |
| Setting | 5 | Describe the setting, locations, and relevant dates, including periods of recruitment, exposure, follow-up, and data collection | 2 | *On June 5, 2020, we searched YouTube using the phrase "THC skin cancer."* |
| Participants | 6 | (*a*) *Cohort study*—Give the eligibility criteria, and the sources and methods of selection of participants. Describe methods of follow-up<br>*Case-control study*—Give the eligibility criteria, and the sources and methods of case ascertainment and control selection. Give the rationale for the choice of cases and controls<br>*Cross-sectional study*—Give the eligibility criteria, and the sources and methods of selection of participants | 2 | *Only nonduplicate videos with over 1000 views were analyzed in order to obtain accurate representation and capture the most popular videos that had reached the largest YouTube audiences.* |
| | | (*b*) *Cohort study*—For matched studies, give matching criteria and number of exposed and unexposed<br>*Case-control study*—For matched studies, give matching criteria and the number of controls per case | NA | |

| | | | | |
|---|---|---|---|---|
| Variables | 7 | Clearly define all outcomes, exposures, predictors, potential confounders, and effect modifiers. Give diagnostic criteria, if applicable | 2 | *For each video, the top 3 comments determined by YouTube according to the number of "thumbs up" ratings were additionally assessed for whether the comment was favorable, neutral, or unfavorable toward the video content. The source and date of the comment were also recorded.*<br><br>*GQS score and DISCERN scale, classified as useful or misleading* |
| Data sources/ measurement | 8* | For each variable of interest, give sources of data and details of methods of assessment (measurement). Describe comparability of assessment methods if there is more than one group | *2* | *Videos were from YouTube*<br><br>*Two independent reviewers viewed and evaluated all videos* |
| Bias | 9 | Describe any efforts to address potential sources of bias | 2 | *any discrepancies between reviewers were discussed and resolved in a consensus meeting. All reviewers were experienced in skin cancer pathogenesis, clinical presentation, and treatment* |
| Study size | 10 | Explain how the study size was arrived at | 2 | *10 videos* |

| | | | | |
|---|---|---|---|---|
| Quantitative variables | 11 | Explain how quantitative variables were handled in the analyses. If applicable, describe which groupings were chosen and why | 2 | *GQS score and DISCERN scale* |
| Statistical methods | 12 | (*a*) Describe all statistical methods, including those used to control for confounding | NA | |
| | | (*b*) Describe any methods used to examine subgroups and interactions | NA | |
| | | (*c*) Explain how missing data were addressed | NA | |
| | | (*d*) *Cohort study*—If applicable, explain how loss to follow-up was addressed<br><br>*Case-control study*—If applicable, explain how matching of cases and controls was addressed | NA | |

| | | | | |
|---|---|---|---|---|
| | | *Cross-sectional study*—If applicable, describe analytical methods taking account of sampling strategy | | |
| | | (*e*) Describe any sensitivity analyses | NA | |
| **Results** | | | | |
| Participants | 13* | (a) Report numbers of individuals at each stage of study—eg numbers potentially eligible, examined for eligibility, confirmed eligible, included in the study, completing follow-up, and analysed | 2 | *The 10 videos surveyed (Multimedia Appendix 1) had a total view count of 645,821 views, with an average of 64,582 views per video.* |
| | | (b) Give reasons for non-participation at each stage | NA | |
| | | (c) Consider use of a flow diagram | NA | |
| Descriptive data | 14* | (a) Give characteristics of study participants (eg demographic, clinical, social) and information on exposures and potential confounders | 2 | *Video length ranged from around 2 minutes to over 107 minutes. Sources of videos were varied, and included cannabis companies, Rick Simpson affiliates, and patient perspectives. The surveyed videos had positive social engagement, with a cumulative "thumbs up" score of 4923, and a "thumbs down" score of 183* |
| | | (b) Indicate number of participants with missing data for each variable of interest | NA | |
| | | (c) *Cohort study*—Summarise follow-up time (eg, average and total amount) | NA | |
| Outcome data | 15* | *Cohort study*—Report numbers of outcome events or summary measures over time | | |
| | | *Case-control study*—Report numbers in each exposure category, or summary measures of exposure | | |
| | | *Cross-sectional study*—Report numbers of outcome events or summary measures | *2* | *Results paragraph 2-3* |
| Main results | 16 | (*a*) Give unadjusted estimates and, if applicable, confounder-adjusted estimates and their precision (eg, 95% confidence interval). Make clear which confounders were adjusted for and why they were included | 2 | *Overall, 10/10 videos (100%) had a GQS score of 1, corresponding to "poor quality, poor flow of the video, most information missing, not at all useful for patients."* |
| | | (*b*) Report category boundaries when continuous variables were categorized | NA | |

| | | | | |
|---|---|---|---|---|
| | | (*c*) If relevant, consider translating estimates of relative risk into absolute risk for a meaningful time period | NA | |
| Other analyses | 17 | Report other analyses done—eg analyses of subgroups and interactions, and sensitivity analyses | NA | |
| Key results | 18 | Summarise key results with reference to study objectives | 3 | *assessment of the information presented as uniformly misleading to viewers, along with a GQS of 1 assigned to all videos, demonstrating the pervasiveness of poor-quality information,* |
| Limitations | 19 | Discuss limitations of the study, taking into account sources of potential bias or imprecision. Discuss both direction and magnitude of any potential bias | NA | |
| Interpretation | 20 | Give a cautious overall interpretation of results considering objectives, limitations, multiplicity of analyses, results from similar studies, and other relevant evidence | 3 | *the importance of accessible, trustworthy, and engaging educational content curated by medical professionals for patients seeking information about skin cancer treatment online.* |
| Generalisability | 21 | Discuss the generalisability (external validity) of the study results | NA | |
| **Other information** | | | | |
| Funding | 22 | Give the source of funding and the role of the funders for the present study and, if applicable, for the original study on which the present article is based | NA | |

Table S139. Quality Evaluation for Included Studies Using STROBE

63.STROBE Statement—checklist of items that should be included in reports of observational studies

| | Item No. | Recommendation | Page No. | Relevant text from manuscript |
|---|---|---|---|---|
| **Title and abstract** | 1 | (*a*) Indicate the study's design with a commonly used term in the title or the abstract | 1 | Cross-sectional content assessment |
| | | (*b*) Provide in the abstract an informative and balanced summary of what was done and what was found | 1 | Methods and Results |
| **Introduction** | | | | |
| Background/rationale | 2 | Explain the scientific background and rationale for the investigation being reported | 2 | In recent years there has been a surge of investigations questioning the usefulness of YouTube videos on health-related topics with various results,[11–13] a great deal however report findings of misleading content |
| Objectives | 3 | State specific objectives, including any prespecified hypotheses | 2 | assess the content, viewer engagement and usefulness of the most viewed YouTube videos pertaining to lung cancer. |
| **Methods** | | | | |
| Study design | 4 | Present key elements of study design early in the paper | 2 | Cross-sectional search |
| Setting | 5 | Describe the setting, locations, and relevant dates, including periods of recruitment, exposure, follow-up, and data collection | 2 | A search of YouTube (http://www.youtube.com) was performed between the 12 July 2021 and 6 August 2021, using the search term 'lung cancer.' |
| Participants | 6 | (*a*) *Cohort study*—Give the eligibility criteria, and the sources and methods of selection of participants. Describe methods of follow-up<br>*Case-control study*—Give the eligibility criteria, and the sources and methods of case ascertainment and control selection. Give the rationale for the choice of cases and controls | 2 | The filter 'sorted by views' was applied and all videos down to 30 000 views were initially reviewed. This resulted in a total of 167 of the most viewed videos, of which 143 were considered eligible to be included in the |

| | | | | |
|---|---|---|---|---|
| | | *Cross-sectional study*—Give the eligibility criteria, and the sources and methods of selection of participants | | analysis with exclusion of non-English videos (*n* = 18) and non-related videos (for example music videos or videogames) |
| | | (*b*) *Cohort study*—For matched studies, give matching criteria and number of exposed and unexposed<br>*Case-control study*—For matched studies, give matching criteria and the number of controls per case | | |
| Variables | 7 | Clearly define all outcomes, exposures, predictors, potential confounders, and effect modifiers. Give diagnostic criteria, if applicable | 2 | Video characteristics including publishing source of upload, MD/oncologist participation in the video, duration of the video, number of views, comments, likes, dislikes and URL were recorded. |
| Data sources/ measurement | 8* | For each variable of interest, give sources of data and details of methods of assessment (measurement). Describe comparability of assessment methods if there is more than one group | *2* | *Videos were from YouTube*<br>The usefulness of the information provided was assessed by two independent physicians |
| Bias | 9 | Describe any efforts to address potential sources of bias | 2 | The search was performed without logging in to an account as well as in Incognito mode to prevent any influence from earlier searches and activity on YouTube. |
| Study size | 10 | Explain how the study size was arrived at | 2 | 143 videos |

| | | | | |
|---|---|---|---|---|
| Quantitative variables | 11 | Explain how quantitative variables were handled in the analyses. If applicable, describe which groupings were chosen and why | 2 | participation in the video, duration of the video, number of views, comments, likes, dislikes |

| | | | | |
|---|---|---|---|---|
| Statistical methods | 12 | (*a*) Describe all statistical methods, including those used to control for confounding | 2 | Descriptive statistics were reported using median (interquartile range; IQR) for continuous variables and percentages for categorical variables. Chi-square test was used to compare proportion between two groups and Mann–Whitney *U* test was used to compare differences for non-parametric data. |
| | | (*b*) Describe any methods used to examine subgroups and interactions | 2 | Chi-square test was used to compare proportion between two groups |
| | | (*c*) Explain how missing data were addressed | NA | |
| | | (*d*) *Cohort study*—If applicable, explain how loss to follow-up was addressed<br>*Case-control study*—If applicable, explain how matching of cases and controls was addressed<br>*Cross-sectional study*—If applicable, describe analytical methods taking account of sampling strategy | NA | |
| | | (*e*) Describe any sensitivity analyses | NA | |
| **Results** | | | | |
| Participants | 13* | (a) Report numbers of individuals at each stage of study—eg numbers potentially eligible, examined for eligibility, confirmed eligible, included in the study, completing follow-up, and analysed | 2 | The filter 'sorted by views' was applied and all videos down to 30 000 views were initially reviewed. This resulted in a total of 167 of the most viewed videos, of which 143 were considered eligible to be included in the analysis with exclusion of non-English videos (*n* = 18) and non-related videos (for example music videos or videogames) |
| | | (b) Give reasons for non-participation at each stage | NA | |
| | | (c) Consider use of a flow diagram | 3 | Fig. 1 |

| | | | | |
|---|---|---|---|---|
| Descriptive data | 14* | (a) Give characteristics of study participants (eg demographic, clinical, social) and information on exposures and potential confounders | 2 | The total number of views were 60 290 040 … The distribution of views, comments, likes and dislikes by source are presented in Table 2. |
| | | (b) Indicate number of participants with missing data for each variable of interest | NA | |
| | | (c) *Cohort study*—Summarise follow-up time (eg, average and total amount) | NA | |
| Outcome data | 15* | *Cohort study*—Report numbers of outcome events or summary measures over time | | |
| | | *Case-control study*—Report numbers in each exposure category, or summary measures of exposure | | |
| | | *Cross-sectional study*—Report numbers of outcome events or summary measures | *2-3* | Aside from the videos that was posted directly by medical doctors, … compared with the misleading group, 317 versus 48, *P* = 0.015 and 430 versus 24, *P* < 0.001 respectively. |
| Main results | 16 | (*a*) Give unadjusted estimates and, if applicable, confounder-adjusted estimates and their precision (eg, 95% confidence interval). Make clear which confounders were adjusted for and why they were included | 3 | 89 (62%) videos were classified as useful.<br>8 (5.6%) videos were considered misleading. |
| | | (*b*) Report category boundaries when continuous variables were categorized | NA | |
| | | (*c*) If relevant, consider translating estimates of relative risk into absolute risk for a meaningful time period | NA | |
| Other analyses | 17 | Report other analyses done—eg analyses of subgroups and interactions, and sensitivity analyses | NA | |
| Key results | 18 | Summarise key results with reference to study objectives | 4 | majority (62%) of the lung cancer-related videos on YouTube were considered useful, however the viewer engagement differed significantly between videos in the misleading and useful group. Videos with misleading content had significantly more views and |

| | | | | |
|---|---|---|---|---|
| | | | | elicited more comments, likes and dislikes than videos providing more accurate information. |
| Limitations | 19 | Discuss limitations of the study, taking into account sources of potential bias or imprecision. Discuss both direction and magnitude of any potential bias | 6 | This study has a number of limitations. … |
| Interpretation | 20 | Give a cautious overall interpretation of results considering objectives, limitations, multiplicity of analyses, results from similar studies, and other relevant evidence | 6 | the number of YouTube videos on lung cancer is increasing, and nearly half of the videos have been uploaded in the past five years. The videos are popular in terms of viewers' interaction and around two thirds of the videos were classified as useful. |
| Generalisability | 21 | Discuss the generalisability (external validity) of the study results | 6 | we included only videos in English or at least with English subtitles. Finally, we did not review all existing videos lung cancer on YouTube, but set a lower limit of 30 000 views as the cut-off value. |
| **Other information** | | | | |
| Funding | 22 | Give the source of funding and the role of the funders for the present study and, if applicable, for the original study on which the present article is based | 6 | Howraman Meteran reports fees from GlaxoSmithKline (lectures and advisory board), Teva (advisory board), Novartis (lectures) and |

Table S140. Quality Evaluation for Included Studies Using STROBE

64.STROBE Statement—checklist of items that should be included in reports of observational studies

| | Item No. | Recommendation | Page No. | Relevant text from manuscript |
|---|---|---|---|---|
| **Title and abstract** | 1 | (*a*) Indicate the study's design with a commonly used term in the title or the abstract | 1 | *cross-sectional study* |
| | | (*b*) Provide in the abstract an informative and balanced summary of what was done and what was found | 2 | Methods and Results |
| **Introduction** | | | | |
| Background/rationale | 2 | Explain the scientific background and rationale for the investigation being reported | 2 | *At present, by the increasing access to the Internet, patients tend to utilize YouTube to obtain medical information*<br><br>*However, the advertisement and non-scientific content uploaded on YouTube raise concerns about the quality and accuracy of medical videos* |
| Objectives | 3 | State specific objectives, including any prespecified hypotheses | 2 | *evaluate the quality of contents on "radioactive iodine treatment" and "radyoaktif iyot tedavisi" by analyzing YouTube videos* |
| **Methods** | | | | |
| Study design | 4 | Present key elements of study design early in the paper | 2 | *cross-sectional study* |
| Setting | 5 | Describe the setting, locations, and relevant dates, including periods of recruitment, exposure, follow-up, and data collection | 2 | *For video selection, the terms "radioactive iodine treatment" and "radyoaktif iyot tedavisi" were used to search for related videos on June 2021* |
| Participants | 6 | (*a*) *Cohort study*—Give the eligibility criteria, and the sources and methods of selection of participants. Describe methods of follow-up<br><br>*Case-control study*—Give the eligibility criteria, and the sources and methods of case ascertainment and control selection. Give the rationale for the choice of cases and controls | 2 | *The inclusion criteria were as follows: English videos on "radioactive iodine treatment" and Turkish videos on "radyoaktif iyot tedavisi."*<br><br>*The exclusion criteria were as follows: Duplicate videos,* |

| | | | | |
|---|---|---|---|---|
| | | *Cross-sectional study*—Give the eligibility criteria, and the sources and methods of selection of participants | | *inaccessible videos, contents unrelated to RAI treatment, and videos in a language other than English and Turkish.* |
| | | (*b*) *Cohort study*—For matched studies, give matching criteria and number of exposed and unexposed<br>*Case-control study*—For matched studies, give matching criteria and the number of controls per case | NA | |
| Variables | 7 | Clearly define all outcomes, exposures, predictors, potential confounders, and effect modifiers. Give diagnostic criteria, if applicable | 2 | *Video duration (seconds), time passed since video upload (days), total views, total comments, number of comments per year, number of likes and dislikes, video like ratio [like/ (like+dislike) ×100], and video view ratio (number of views/ days) were recorded during the evaluation procedure. Video power index (VPI) (like ratio × view ratio/100)* |
| Data sources/ measurement | 8* | For each variable of interest, give sources of data and details of methods of assessment (measurement). Describe comparability of assessment methods if there is more than one group | 2 | *Videos were from YouTube* |
| Bias | 9 | Describe any efforts to address potential sources of bias | 2 | *All URLs retracted were recorded in an Excel sheet and assessed by a nuclear medicine specialist experienced in RAI treatment.* |
| Study size | 10 | Explain how the study size was arrived at | 2 | *56 videos (30 in English, 26 in Turkish) were included in the study* |

| | | | | |
|---|---|---|---|---|
| Quantitative variables | 11 | Explain how quantitative variables were handled in the analyses. If applicable, describe which groupings were chosen and why | 2-3 | *Video duration (seconds), time passed since video upload (days), total views, total comments, number of comments per year, number of likes and dislikes, video like* |

| | | | | |
|---|---|---|---|---|
| | | | | *ratio [like/ (like+dislike) .100], and video view ratio (number of views/ days) were recorded during the evaluation procedure.*<br><br>*Video power index (VPI) (like ratio . view ratio/100)*<br><br>*American Medical Association (JAMA) benchmark criteria,*<br><br>*DISCERN Scale, and global quality scale (GQS).* |
| Statistical methods | 12 | (*a*) Describe all statistical methods, including those used to control for confounding | 3 | *The Kolmogorov-Smirnov test was used to determine whether the obtained parameters conformed to a normal distribution. Descriptive analyses were used, and values*<br><br>*were presented as mean ± standard deviation (SD). The Kruskal-Wallis and Mann-Whitney U tests were used to compare continuous variables. While investigating the associations between non-normally distributed or ordinal variables, the correlation coefficients (r) and their significance (p) were calculated using the Spearman test.* |
| | | (*b*) Describe any methods used to examine subgroups and interactions | 2 | *Video sources were analyzed into five categories as physician, patient, nuclear medicine physicist, nurse, and nutritionist* |
| | | (*c*) Explain how missing data were addressed | NA | |
| | | (*d*) *Cohort study*—If applicable, explain how loss to follow-up was addressed<br><br>*Case-control study*—If applicable, explain how matching of cases and controls was addressed<br><br>*Cross-sectional study*—If applicable, describe analytical methods taking account of sampling strategy | NA | |
| | | (*e*) Describe any sensitivity analyses | NA | |

| | | | | |
|---|---|---|---|---|
| **Results** | | | | |
| Participants | 13* | (a) Report numbers of individuals at each stage of study—eg numbers potentially eligible, examined for eligibility, confirmed eligible, included in the study, completing follow-up, and analysed | 3 | *We have evaluated a total of 88 videos. Of these videos, 31.81% (n=28) had unrelated content, and 4.5% (n=4) were videos uploaded in languages other than English and Turkish. We have analyzed a total of 56 videos according to the inclusion and exclusion criteria.* |
| | | (b) Give reasons for non-participation at each stage | NA | |
| | | (c) Consider use of a flow diagram | NA | |
| Descriptive data | 14* | (a) Give characteristics of study participants (eg demographic, clinical, social) and information on exposures and potential confounders | 3 | *"The average duration of videos was 455.07 (SD: 410.11) seconds. … The average VPI was 17.04 (SD: 22.05) (Table 2)."* |
| | | (b) Indicate number of participants with missing data for each variable of interest | NA | |
| | | (c) *Cohort study*—Summarise follow-up time (eg, average and total amount) | NA | |
| Outcome data | 15* | *Cohort study*—Report numbers of outcome events or summary measures over time | | |
| | | *Case-control study*—Report numbers in each exposure category, or summary measures of exposure | | |
| | | *Cross-sectional study*—Report numbers of outcome events or summary measures | *3-4* | *"The average durations of low-, intermediate-, and…"* |
| Main results | 16 | (*a*) Give unadjusted estimates and, if applicable, confounder-adjusted estimates and their precision (eg, 95% confidence interval). Make clear which confounders were adjusted for and why they were included | 5 | *The average JAMA (1.92, SD: 0.50), DISCERN (34.31, SD: 14.33), and GQS scores (2.61, SD: 0.99) were highest in physician-made videos.* |
| | | (*b*) Report category boundaries when continuous variables were categorized | 3 | Quality groups (high, intermediate, low) based on score ranges |
| | | (*c*) If relevant, consider translating estimates of relative risk into absolute risk for a meaningful time period | NA | |

| | | | | |
|---|---|---|---|---|
| Other analyses | 17 | Report other analyses done—eg analyses of subgroups and interactions, and sensitivity analyses | NA | |
| Key results | 18 | Summarise key results with reference to study objectives | 5 | *the most viewed and commented videos, the most liked, and the most popular videos (highest VPI scores) were made by non-physicians. In addition, videos with the highest quality based on JAMA, DISCERN, and GQS scores were physician-made videos* |
| Limitations | 19 | Discuss limitations of the study, taking into account sources of potential bias or imprecision. Discuss both direction and magnitude of any potential bias | 6 | *This study has several limitations …* |
| Interpretation | 20 | Give a cautious overall interpretation of results considering objectives, limitations, multiplicity of analyses, results from similar studies, and other relevant evidence | 6 | *physicians should provide detailed verbal and written information to patients about their disease and treatment and refer patients to scientific sources which they can obtain reliable information.* |
| Generalisability | 21 | Discuss the generalisability (external validity) of the study results | 6 | *Inclusion of videos in other languages and all videos in English and Turkish about RAI treatment may change our findings,* |
| **Other information** | | | | |
| Funding | 22 | Give the source of funding and the role of the funders for the present study and, if applicable, for the original study on which the present article is based | 6 | *The author declared that this study has received no financial support.* |

Table S141. Quality Evaluation for Included Studies Using STROBE

65.STROBE Statement—checklist of items that should be included in reports of observational studies

| | Item No. | Recommendation | Page No. | Relevant text from manuscript |
|---|---|---|---|---|
| **Title and abstract** | 1 | (*a*) Indicate the study's design with a commonly used term in the title or the abstract | 1 | Cross-sectional search |
| | | (*b*) Provide in the abstract an informative and balanced summary of what was done and what was found | 1 | Using the search terms … misleading information 40% and 20% of the time, respectively. |
| **Introduction** | | | | |
| Background/rationale | 2 | Explain the scientific background and rationale for the investigation being reported | 2 | *interest in safety aspects of health information provided by social media has gained importance and a vast number of investigations have been published on various aspects of health information delivered by YouTube and the like* |
| Objectives | 3 | State specific objectives, including any prespecified hypotheses | 2 | investigate health information related to sun protection, skin cancer prevention, and related topics presented in YouTube videos. |
| **Methods** | | | | |
| Study design | 4 | Present key elements of study design early in the paper | 3 | cross-sectional analysis |
| Setting | 5 | Describe the setting, locations, and relevant dates, including periods of recruitment, exposure, follow-up, and data collection | 3 | for each search term, we employed the following structured search procedure: a) Open Google Chrome (version 32 or later) on a personal computer |
| Participants | 6 | (*a*) *Cohort study*—Give the eligibility criteria, and the sources and methods of selection of participants. Describe methods of follow-up<br>*Case-control study*—Give the eligibility criteria, and the sources and methods of case ascertainment and control selection. Give the rationale for the choice of cases and controls | 3 | Inclusion and exclusion criteria: Videos in Danish, English, French, German, Spanish, or Swedish with |

| | | | | |
|---|---|---|---|---|
| | | *Cross-sectional study*—Give the eligibility criteria, and the sources and methods of selection of participants | | direct reference to the search term and duration of video > 5 minutes were included. Duplicate videos were excluded. |
| | | (*b*) *Cohort study*—For matched studies, give matching criteria and number of exposed and unexposed<br>*Case-control study*—For matched studies, give matching criteria and the number of controls per case | | |
| Variables | 7 | Clearly define all outcomes, exposures, predictors, potential confounders, and effect modifiers. Give diagnostic criteria, if applicable | 2 | *General information: For each video, language, the niform Resource Locator (URL), title, authorship, date of upload, duration of video, and date of search ere collected.*<br>*Popularity driven measures: Number of views, likes, dislikes, and comments were noted and the number of views, likes, and dislikes per month were calculated.* |
| Data sources/ measurement | 8* | For each variable of interest, give sources of data and details of methods of assessment (measurement). Describe comparability of assessment methods if there is more than one group | *3* | *Videos were from YouTube*<br>*Experts including dermatologists, pharmacists, public health professionals, biologists, and physicists were acquainted with the questionnaire and received a detailed research manual guide for video analysis.* |
| Bias | 9 | Describe any efforts to address potential sources of bias | 3 | *Experts including dermatologists, pharmacists, public health professionals, biologists, and physicists were acquainted with the questionnaire and received a detailed research manual guide for video analysis.* |

| | | | | |
|---|---|---|---|---|
| Study size | 10 | Explain how the study size was arrived at | 4 | 281 videos |
| Quantitative variables | 11 | Explain how quantitative variables were handled in the analyses. If applicable, describe which groupings were chosen and why | 3 | *Popularity driven measures: Number of views, likes, dislikes, and comments were noted and the number of views, likes, and dislikes per month were calculated.* |
| Statistical methods | 12 | (*a*) Describe all statistical methods, including those used to control for confounding | 3 | *For quantification of data variability, means and standard deviation (SD) were reported for normally distributed and medians with inter-quartile ranges (IQR) for non-normally distributed variables. Total scores were calculated where possible. Comparison of more than two groups of means was evaluated using univariate analysis of variance (ANOVA). Chi² statistics were used to identify variables that were ignificantly associated with misleading or inaccurate information. P-values below 0.05 were considered significant.* |
| | | (*b*) Describe any methods used to examine subgroups and interactions | NA | |
| | | (*c*) Explain how missing data were addressed | NA | |
| | | (*d*) *Cohort study*—If applicable, explain how loss to follow-up was addressed<br>*Case-control study*—If applicable, explain how matching of cases and controls was addressed<br>*Cross-sectional study*—If applicable, describe analytical methods taking account of sampling strategy | NA | |
| | | (*e*) Describe any sensitivity analyses | NA | |

**Results**

| | | | | |
|---|---|---|---|---|
| Participants | 13* | (a) Report numbers of individuals at each stage of study—eg numbers potentially eligible, examined for eligibility, confirmed eligible, included in the study, completing follow-up, and analysed | 4 | 281 videos with running times of less than 5 minutes were cluded. For details on search term and language see Tables 1a and 1b. |
| | | (b) Give reasons for non-participation at each stage | NA | |
| | | (c) Consider use of a flow diagram | NA | |
| Descriptive data | 14* | (a) Give characteristics of study participants (eg demographic, clinical, social) and information on exposures and potential confounders | 4 | Popularity driven measures |
| | | (b) Indicate number of participants with missing data for each variable of interest | NA | |
| | | (c) *Cohort study*—Summarise follow-up time (eg, average and total amount) | NA | |
| Outcome data | 15* | *Cohort study*—Report numbers of outcome events or summary measures over time | | |
| | | *Case-control study*—Report numbers in each exposure category, or summary measures of exposure | | |
| | | *Cross-sectional study*—Report numbers of outcome events or summary measures | *5-7* | From the 281 videos analyzed, 160 (57.3%) mentioned up to… individuals were exhibited in 65 (23.1%) video-excerpts. |
| Main results | 16 | (*a*) Give unadjusted estimates and, if applicable, confounder-adjusted estimates and their precision (eg, 95% confidence interval). Make clear which confounders were adjusted for and why they were included | 7 | Videos retrieved with the search term “tanning bed” were included (n=50): 18 (36.0%) explicitly recommended the use of tanning beds and 9 (18.0%) recommended tanning bed use for vitamin D synthesis. In addition, 21 (42.0%) of the videos contained up to 4 false or misleading information. |
| | | (*b*) Report category boundaries when continuous variables were categorized | NA | |
| | | (*c*) If relevant, consider translating estimates of relative risk into absolute risk for a meaningful time period | NA | |

| | | | | |
|---|---|---|---|---|
| Other analyses | 17 | Report other analyses done—eg analyses of subgroups and interactions, and sensitivity analyses | 5-7 | Comparison by theme |

| | | | | |
|---|---|---|---|---|
| Key results | 18 | Summarise key results with reference to study objectives | 10 | In our investigation, we confirm observations made in other medical disciplines that the general quality of YouTube contributions is often inferior and these videos do not deliver sustainable information. |
| Limitations | 19 | Discuss limitations of the study, taking into account sources of potential bias or imprecision. Discuss both direction and magnitude of any potential bias | 8 | Owing to the international assessment and the number (8) of researchers actively involved in the assessment, it was not possible to determine an overall inter-rate reliability. Sixteen variables were detected as unreliable in the English and German reliability assessment and were removed them from he entire assessment (all languages). |
| Interpretation | 20 | Give a cautious overall interpretation of results considering objectives, limitations, multiplicity of analyses, results from similar studies, and other relevant evidence | 10 | *Based on current rules, the launch of a YouTube channel by a competent organization may be an effective path to disseminate peer reviewed information to both professionals and laypersons.* |
| Generalisability | 21 | Discuss the generalisability (external validity) of the study results | 8 | Owing to the international assessment and the number (8) of researchers actively involved in the assessment, it was not possible to determine an overall inter-rate reliability. |
| **Other information** | | | | |
| Funding | 22 | Give the source of funding and the role of the funders for the present study and, if applicable, for the original study on which the present article is based | NA | |

Table S142. Quality Evaluation for Included Studies Using STROBE

66.STROBE Statement—checklist of items that should be included in reports of observational studies

| | Item No. | Recommendation | Page No. | Relevant text from manuscript |
|---|---|---|---|---|
| **Title and abstract** | 1 | (*a*) Indicate the study's design with a commonly used term in the title or the abstract | 1 | cross-sectional study |
| | | (*b*) Provide in the abstract an informative and balanced summary of what was done and what was found | 1 | Methods and Results |
| **Introduction** | | | | |
| Background/rationale | 2 | Explain the scientific background and rationale for the investigation being reported | 1 | *the medical content available on YouTube is controversial and not properly examined* |
| Objectives | 3 | State specific objectives, including any prespecified hypotheses | 2 | *report an evaluation of the quality, reliability, and content of videos related to breast cancer on YouTube in the Arabic language* |
| **Methods** | | | | |
| Study design | 4 | Present key elements of study design early in the paper | 2 | *cross-sectional study* |
| Setting | 5 | Describe the setting, locations, and relevant dates, including periods of recruitment, exposure, follow-up, and data collection | 2 | *YouTube was searched on the 0th of May 2020, using the keyword "breast cancer" in rabic* |
| Participants | 6 | (*a*) *Cohort study*—Give the eligibility criteria, and the sources and methods of selection of participants. Describe methods of follow-up<br>*Case-control study*—Give the eligibility criteria, and the sources and methods of case ascertainment and control selection. Give the rationale for the choice of cases and controls<br>*Cross-sectional study*—Give the eligibility criteria, and the sources and methods of selection of participants | 2 | *Videos were sorted by view count. The first 100 videos were selected for preliminary analysis*<br>*The exclusion criteria were entertainment …* |
| | | (*b*) *Cohort study*—For matched studies, give matching criteria and number of exposed and unexposed<br>*Case-control study*—For matched studies, give matching criteria and the number of controls per case | NA | |
| Variables | 7 | Clearly define all outcomes, exposures, predictors, potential confounders, and effect modifiers. Give diagnostic criteria, if applicable | 2 | *upload date, number of views, number of likes, numberof dislikes, and* |

| | | | | |
|---|---|---|---|---|
| | | | | *duration ideo power index, viewers' interaction* |
| Data sources/ measurement | 8* | For each variable of interest, give sources of data and details of methods of assessment (measurement). Describe comparability of assessment methods if there is more than one group | *2* | *Videos were from YouTube*<br>*The evaluation of all videos was performed independently by two examiners* |
| Bias | 9 | Describe any efforts to address potential sources of bias | 2 | *The evaluation of all videos was performed independently by two examiners (GA and EC). After comparing the data, discrepancies were discussed with a third examiner (HRK).* |
| Study size | 10 | Explain how the study size was arrived at | 3 | 60 videos |

| | | | | |
|---|---|---|---|---|
| Quantitative variables | 11 | Explain how quantitative variables were handled in the analyses. If applicable, describe which groupings were chosen and why | 2 | *GQS score, DISCERN score, video content coverage* |
| Statistical methods | 12 | (*a*) Describe all statistical methods, including those used to control for confounding | 2 | *Data distribution was assessed using a Shapiro–Wilk test. Descriptive statistics were reported including medians (1st quartile–3rd uartile) for continuous variables and percentages for categorical ariables. Mann–Whitney U test was used for the numerical variables. The correlation analysis was performed using Pearson correlation analysis. For the comparison of categorical variables, the chi-square was used.* |
| | | (*b*) Describe any methods used to examine subgroups and interactions | 2 | *For the comparison of categorical variables, the chi-square was used* |
| | | (*c*) Explain how missing data were addressed | NA | |
| | | (*d*) *Cohort study*—If applicable, explain how loss to follow-up was addressed | NA | |

| | | | | |
|---|---|---|---|---|
| | | *Case-control study*—If applicable, explain how matching of cases and controls was addressed<br><br>*Cross-sectional study*—If applicable, describe analytical methods taking account of sampling strategy | | |
| | | (e) Describe any sensitivity analyses | NA | |
| **Results** | | | | |
| Participants | 13* | (a) Report numbers of individuals at each stage of study—eg numbers potentially eligible, examined for eligibility, confirmed eligible, included in the study, completing follow-up, and analysed | 3 | *A total of 40 videos were excluded as they were entertainment shows (n: 14), related to another type of cancer (n:8), not in Arabic (n: 7), music videos (n: 3), religious verses (n:3), related to male breast cancer (n:2), advertisements (n: 2), or a duplicate (n: 1). The remaining 60 videos were included in the study for further analysis* |
| | | (b) Give reasons for non-participation at each stage | NA | |
| | | (c) Consider use of a flow diagram | 4 | Figure 2 |
| Descriptive data | 14* | (a) Give characteristics of study participants (eg demographic, clinical, social) and information on exposures and potential confounders | 3 | Results paragraph 2<br><br>"*Of the 60 included videos, 27 videos (45%) … and the median views/day per video was 210 (98–431).*" |
| | | (b) Indicate number of participants with missing data for each variable of interest | NA | |
| | | (c) *Cohort study*—Summarise follow-up time (eg, average and total amount) | NA | |
| Outcome data | 15* | *Cohort study*—Report numbers of outcome events or summary measures over time | | |
| | | *Case-control study*—Report numbers in each exposure category, or summary measures of exposure | | |
| | | *Cross-sectional study*—Report numbers of outcome events or summary measures | *3* | *Results paragraph 3-*<br><br>*"At least one misleading information was found in 25 videos… and 10 (17%) as moderately useful."* |

| | | | | |
|---|---|---|---|---|
| Main results | 16 | (*a*) Give unadjusted estimates and, if applicable, confounder-adjusted estimates and their precision (eg, 95% confidence interval). Make clear which confounders were adjusted for and why they were included | 3 | *A statistically significant difference was found in favor of the videos ploaded by the blogs source category for the views, the views/day, and the video power index (p < 0.05). Videos uploaded by the legal person source category scored significantly higher on GQS score, reliability score, and content score than those uploaded by the blogs source category (p < 0.05).* |
| | | (*b*) Report category boundaries when continuous variables were categorized | NA | |
| | | (*c*) If relevant, consider translating estimates of relative risk into absolute risk for a meaningful time period | NA | |
| Other analyses | 17 | Report other analyses done—eg analyses of subgroups and interactions, and sensitivity analyses | 3-4 | *according to the source category* |
| Key results | 18 | Summarise key results with reference to study objectives | 5 | *In our study, the high number of total views (21,514,441) emphasizes the popularity of the subject. However, the videos evaluated were poorly reliable, and their content is poorly useful for patients. Their global quality was poor to moderate.* |
| Limitations | 19 | Discuss limitations of the study, taking into account sources of potential bias or imprecision. Discuss both direction and magnitude of any potential bias | 6 | *Certain limitations to this study should be considered.* |
| Interpretation | 20 | Give a cautious overall interpretation of results considering objectives, limitations, multiplicity of analyses, results from similar studies, and other relevant evidence | 7 | *Governments and healthcare providers are encouraged to upload more intelligibly informational videos, guide the population to reliable sources, and encourage regulations on YouTube medical content.* |
| Generalisability | 21 | Discuss the generalisability (external validity) of the study results | 7 | *may not be representative of all the population in countries that have Arabic as an official* |

| | | | | |
|---|---|---|---|---|
| | | | | *language, as users may opt for content in other languages.* |
| **Other information** | | | | |
| Funding | 22 | Give the source of funding and the role of the funders for the present study and, if applicable, for the original study on which the present article is based | 7 | *The authors declare no competing interests.* |

Table S143. Quality Evaluation for Included Studies Using STROBE

67.STROBE Statement—checklist of items that should be included in reports of observational studies

| | Item No. | Recommendation | Page No. | Relevant text from manuscript |
|---|---|---|---|---|
| **Title and abstract** | 1 | (*a*) Indicate the study's design with a commonly used term in the title or the abstract | 1 | *descriptive cross-sectional design* |
| | | (*b*) Provide in the abstract an informative and balanced summary of what was done and what was found | 1 | "*A descriptive cross-sectional design was used, … and 30% as inappropriate sources of information.*" |
| **Introduction** | | | | |
| Background/rationale | 2 | Explain the scientific background and rationale for the investigation being reported | 2 | *Little is known about the kind of information parents of children with cancer find when searching for clinical trials information on YouTube* |
| Objectives | 3 | State specific objectives, including any prespecified hypotheses | 2 | *analyse the content, quality and reliability of YouTube videos focused on clinical trials for paediatric cancer.* |
| **Methods** | | | | |
| Study design | 4 | Present key elements of study design early in the paper | 3 | *descriptive, cross-sectional study design* |
| Setting | 5 | Describe the setting, locations, and relevant dates, including periods of recruitment, exposure, follow-up, and data collection | 3 | *Using the search phrases 'clinical trials for children with cancer' and 'paediatric cancer clinical trials', a search was conducted (on July 3 and 6 2020) in the English language, using filters for 'videos' and 'view count'; thus, videos were listed according to popularity* |
| Participants | 6 | (*a*) *Cohort study*—Give the eligibility criteria, and the sources and methods of selection of participants. Describe methods of follow-up<br>*Case-control study*—Give the eligibility criteria, and the sources and methods of case ascertainment and control selection. Give the rationale for the choice of cases and controls | 2 | *Inclusion and exclusion criteria* |

| | | | | |
|---|---|---|---|---|
| | | *Cross-sectional study*—Give the eligibility criteria, and the sources and methods of selection of participants | | |
| | | (*b*) *Cohort study*—For matched studies, give matching criteria and number of exposed and unexposed<br><br>*Case-control study*—For matched studies, give matching criteria and the number of controls per case | NA | |
| Variables | 7 | Clearly define all outcomes, exposures, predictors, potential confounders, and effect modifiers. Give diagnostic criteria, if applicable | 4 | *The videos included in this study were evaluated in detail, with noted characteristics that included the country of origin, length, the date of upload, number of views and 'thumbs up' or 'thumbs down' (a way for viewers to convey like or dislike of the video).* |
| Data sources/ measurement | 8* | For each variable of interest, give sources of data and details of methods of assessment (measurement). Describe comparability of assessment methods if there is more than one group | *3* | *Videos were from YouTube*<br><br>*Two researchers (TG and SE), experienced in clinical trial research ethics, ranked the first twelve sampled videos cooperatively* |
| Bias | 9 | Describe any efforts to address potential sources of bias | 4 | *In uncertain cases SE crosschecked the ranking independently to ensure consistent and intersubjective rankings.* |
| Study size | 10 | Explain how the study size was arrived at | 6 | *40 videos* |

| | | | | |
|---|---|---|---|---|
| Quantitative variables | 11 | Explain how quantitative variables were handled in the analyses. If applicable, describe which groupings were chosen and why | 4 | GQS and DISCERN scores |
| Statistical methods | 12 | (*a*) Describe all statistical methods, including those used to control for confounding | 6 | Descriptive statistics: means, standard deviations, frequencies |
| | | (*b*) Describe any methods used to examine subgroups and interactions | 5 | *Clinical trial phases discussed were categorised into three groups* |

| | | | | |
|---|---|---|---|---|
| | | (*c*) Explain how missing data were addressed | NA | |
| | | (*d*) *Cohort study*—If applicable, explain how loss to follow-up was addressed<br><br>*Case-control study*—If applicable, explain how matching of cases and controls was addressed<br><br>*Cross-sectional study*—If applicable, describe analytical methods taking account of sampling strategy | NA | |
| | | (*e*) Describe any sensitivity analyses | NA | |
| **Results** | | | | |
| Participants | 13* | (a) Report numbers of individuals at each stage of study—eg numbers potentially eligible, examined for eligibility, confirmed eligible, included in the study, completing follow-up, and analysed | 6 | *The search process resulted in eighty-seven videos. Thirty-seven of these were excluded with reason (see Figure 1 for the criteria) and the resulting 50 were screened according to GQS criteria. After excluding 10 videos because of poor audio-visual quality and flow, 40 unique videos with acceptable audio-visual quality and flow remained. Of the 40 videos,* |
| | | (b) Give reasons for non-participation at each stage | NA | |
| | | (c) Consider use of a flow diagram | 5 | Figure 1 |
| Descriptive data | 14* | (a) Give characteristics of study participants (eg demographic, clinical, social) and information on exposures and potential confounders | 6 | "*22 of them were in the excellent … perceived of as useful by most viewers*" |
| | | (b) Indicate number of participants with missing data for each variable of interest | NA | |
| | | (c) *Cohort study*—Summarise follow-up time (eg, average and total amount) | NA | |
| Outcome data | 15* | *Cohort study*—Report numbers of outcome events or summary measures over time | *NA* | |
| | | *Case-control study*—Report numbers in each exposure category, or summary measures of exposure | *NA* | |
| | | *Cross-sectional study*—Report numbers of outcome events or summary measures | *7* | *Paragraph 2-4* |
| Main results | 16 | (*a*) Give unadjusted estimates and, if applicable, confounder-adjusted estimates and their precision (eg, 95% confidence interval). Make clear which confounders were adjusted for and why they were included | 7 | *The quality of the videos, expressed by the mean overall DISCERN rating scores, was generally low on a scale of 1 to* |

| | | | | |
|---|---|---|---|---|
| | | | | *5, with 5 being the maximum. None of the videos was classified as excellent* |
| | | (*b*) Report category boundaries when continuous variables were categorized | NA | |
| | | (*c*) If relevant, consider translating estimates of relative risk into absolute risk for a meaningful time period | NA | |
| Other analyses | 17 | Report other analyses done—eg analyses of subgroups and interactions, and sensitivity analyses | NA | |
| Key results | 18 | Summarise key results with reference to study objectives | 8 | *The average quality of YouTube videos on clinical trials for paediatric cancer was low when scored using DISCERN.* |
| Limitations | 19 | Discuss limitations of the study, taking into account sources of potential bias or imprecision. Discuss both direction and magnitude of any potential bias | 10 | *Some study limitations should be noted…* |
| Interpretation | 20 | Give a cautious overall interpretation of results considering objectives, limitations, multiplicity of analyses, results from similar studies, and other relevant evidence | 10-11 | *healthcare providers should be encouraged to INFORMATION, COMMUNICATION & SOCIETY 725 … information about paediatric cancer trials.* |
| Generalisability | 21 | Discuss the generalisability (external validity) of the study results | 10 | *the inclusion of videos uploaded earlier than 2010 and videos with <100 views could give other results, affecting a minor group of viewers. Additionally, non English language videos were excluded, which reduces the generalisability of our results to videos in other languages.* |
| **Other information** | | | | |
| Funding | 22 | Give the source of funding and the role of the funders for the present study and, if applicable, for the original study on which the present article is based | 11 | *No potential conflict of interest was reported by the author(s).* |

Table S144. Quality Evaluation for Included Studies Using STROBE

68.STROBE Statement—checklist of items that should be included in reports of observational studies

| | Item No. | Recommendation | Page No. | Relevant text from manuscript |
|---|---|---|---|---|
| **Title and abstract** | 1 | (*a*) Indicate the study's design with a commonly used term in the title or the abstract | 1 | *descriptive study* |
| | | (*b*) Provide in the abstract an informative and balanced summary of what was done and what was found | 1 | Materials and Methods<br>Results |
| **Introduction** | | | | |
| Background/rationale | 2 | Explain the scientific background and rationale for the investigation being reported | 2 | *According to the Health Information National Trends Survey data, there has been a significant increase in internet use to access health-related information, with recent research revealing that eight of 10 internet users access health information online. However, due to the absence of a mechanism that controls video content, videos uploaded on YouTube can be misleading for users* |
| Objectives | 3 | State specific objectives, including any prespecified hypotheses | 2 | *evaluate the content and reliability of the top 100 videos about Merkel cell carcinoma on YouTube.* |
| **Methods** | | | | |
| Study design | 4 | Present key elements of study design early in the paper | 2 | descriptive study |
| Setting | 5 | Describe the setting, locations, and relevant dates, including periods of recruitment, exposure, follow-up, and data collection | 2 | videos about Merkel cell carcinoma were searched on www.youtube.com on July 1, 2022, using the term "Merkel cell carcinoma". |
| Participants | 6 | (*a*) *Cohort study*—Give the eligibility criteria, and the sources and methods of selection of participants. Describe methods of follow-up | 2 | Since internet searchers rarely display over 10 results, it was assumed that examining 100 videos would provide sufficient information. |

| | | | | |
|---|---|---|---|---|
| | | *Case-control study*—Give the eligibility criteria, and the sources and methods of case ascertainment and control selection. Give the rationale for the choice of cases and controls<br><br>*Cross-sectional study*—Give the eligibility criteria, and the sources and methods of selection of participants | | The top 100 videos were ordered and recorded. |
| | | (*b*) *Cohort study*—For matched studies, give matching criteria and number of exposed and unexposed<br><br>*Case-control study*—For matched studies, give matching criteria and the number of controls per case | | |
| Variables | 7 | Clearly define all outcomes, exposures, predictors, potential confounders, and effect modifiers. Give diagnostic criteria, if applicable | 2 | *The videos were watched, and parameters such as video length, number of likes, number of comments, number of views, and video upload date were recorded.* |
| Data sources/ measurement | 8* | For each variable of interest, give sources of data and details of methods of assessment (measurement). Describe comparability of assessment methods if there is more than one group | *2* | *Videos were from YouTube*<br><br>*The videos were evaluated by researchers* |
| Bias | 9 | Describe any efforts to address potential sources of bias | 2 | *Cookies and browsing history were cleared before each search to ensure results would not be affected by previous searches.* |
| Study size | 10 | Explain how the study size was arrived at | 2 | 100 videos |

| | | | | |
|---|---|---|---|---|
| Quantitative variables | 11 | Explain how quantitative variables were handled in the analyses. If applicable, describe which groupings were chosen and why | 2 | *video length, number of likes,*<br><br>*number of comments, number of views*<br><br>*The Global Quality scale (GQS),*<br><br>*The modified DISCERN tool* |
| Statistical methods | 12 | (*a*) Describe all statistical methods, including those used to control for confounding | 2 | *Categorical measurements were summarized as numbers and percentages, and continuous measurements as median and minimum-maximum values.* |

| | | | | |
|---|---|---|---|---|
| | | | | *The Shapiro-Wilk test was used to determine whether the parameters in the study showed a normal distribution. The chi-square and Fisher's exact tests were used to compare categorical data. The Mann-Whitney U test was conducted to analyze the differences between the groups.* |
| | | (*b*) Describe any methods used to examine subgroups and interactions | 2 | *The Mann-Whitney U test was conducted to analyze the differences between the groups.* |
| | | (*c*) Explain how missing data were addressed | NA | |
| | | (*d*) *Cohort study*—If applicable, explain how loss to follow-up was addressed<br>*Case-control study*—If applicable, explain how matching of cases and controls was addressed<br>*Cross-sectional study*—If applicable, describe analytical methods taking account of sampling strategy | NA | |
| | | (*e*) Describe any sensitivity analyses | NA | |
| **Results** | | | | |
| Participants | 13* | (a) Report numbers of individuals at each stage of study—eg numbers potentially eligible, examined for eligibility, confirmed eligible, included in the study, completing follow-up, and analysed | 2 | *Of the videos about Merkel cell carcinoma, 72% were related to treatment, 36% diagnosis, 19% pathogenesis, and 16% risk factors.* |
| | | (b) Give reasons for non-participation at each stage | NA | |
| | | (c) Consider use of a flow diagram | NA | |
| Descriptive data | 14* | (a) Give characteristics of study participants (eg demographic, clinical, social) and information on exposures and potential confounders | 2 | *The content of the remaining videos is presented in Table 1. Some videos had more than one content feature (Table 1). When the videos were grouped as useful and misleading, the two groups were found to have similar characteristics in terms of video length (min), number of views, number of views/day,* |

| | | | | |
|---|---|---|---|---|
| | | | | *number of likes, number of likes/day, and number of comments.* |
| | | (b) Indicate number of participants with missing data for each variable of interest | NA | |
| | | (c) *Cohort study*—Summarise follow-up time (eg, average and total amount) | NA | |
| Outcome data | 15* | *Cohort study*—Report numbers of outcome events or summary measures over time | | |
| | | *Case-control study*—Report numbers in each exposure category, or summary measures of exposure | | |
| | | *Cross-sectional study*—Report numbers of outcome events or summary measures | *2* | *The useful videos had significantly higher GQS and DISCERN scores compared to the misleading videos. The videos were aimed at healthcare professionals, followed by patients. The majority of the videos were narrated by a medical journal, organization, or doctor. The USA and the United Kingdom were the top countries in terms of upload source. The detailed data are shown in Tables 2 and 3.* |
| Main results | 16 | (*a*) Give unadjusted estimates and, if applicable, confounder-adjusted estimates and their precision (eg, 95% confidence interval). Make clear which confounders were adjusted for and why they were included | 2 | *The useful videos had significantly higher GQS and DISCERN scores compared to the misleading videos.* |
| | | (*b*) Report category boundaries when continuous variables were categorized | NA | |
| | | (*c*) If relevant, consider translating estimates of relative risk into absolute risk for a meaningful time period | NA | |
| Other analyses | 17 | Report other analyses done—eg analyses of subgroups and interactions, and sensitivity analyses | NA | |
| Key results | 18 | Summarise key results with reference to study objectives | 4 | *YouTube videos on Merkel cell carcinoma were examined, and 81% were evaluated as useful and 19% as misleading. The GQS and DISCERN scores of* |

| | | | | |
|---|---|---|---|---|
| | | | | *the useful videos were statistically significantly higher than those of the misleading videos (p<0.001).* |
| Limitations | 19 | Discuss limitations of the study, taking into account sources of potential bias or imprecision. Discuss both direction and magnitude of any potential bias | 4 | *Although this study is the first to examine the content and reliability of YouTube videos on Merkel cell carcinoma, it has the limitation of the videos being evaluated by a single rater.* |
| Interpretation | 20 | Give a cautious overall interpretation of results considering objectives, limitations, multiplicity of analyses, results from similar studies, and other relevant evidence | 4 | *In the selection of videos on health-related topics, those published by experts (such as medical journals, doctors, and universities) should be prioritized, and YouTube should not be the only source of information.* |
| Generalisability | 21 | Discuss the generalisability (external validity) of the study results | 4 | *The videos being evaluated by a single rater.* |
| **Other information** | | | | |
| Funding | 22 | Give the source of funding and the role of the funders for the present study and, if applicable, for the original study on which the present article is based | 4 | *Financial Disclosure: The author declared that this study received no financial support.* |

Table S145. Quality Evaluation for Included Studies Using STROBE

69.STROBE Statement—checklist of items that should be included in reports of observational studies

| | Item No. | Recommendation | Page No. | Relevant text from manuscript |
|---|---|---|---|---|
| **Title and abstract** | 1 | (*a*) Indicate the study's design with a commonly used term in the title or the abstract | 1 | A Content Quality and Audience Engagement Analysis |
| | | (*b*) Provide in the abstract an informative and balanced summary of what was done and what was found | 1 | *A YT search was conducted for the following … Misleading information was presented in 35 productions.* |
| **Introduction** | | | | |
| Background/rationale | 2 | Explain the scientific background and rationale for the investigation being reported | 1 | *videos published on YT should be regularly checked, in terms of their quality, and improved, as they constitute a source of medical knowledge for a large group of recipients without medical education.* |
| Objectives | 3 | State specific objectives, including any prespecified hypotheses | 2 | *evaluate the quality of YT videos about meningiomas for patients, their families, and other concerned individuals, using appropriately validated scientific scoring systems* |
| **Methods** | | | | |
| Study design | 4 | Present key elements of study design early in the paper | 2 | Cross-sectional search |
| Setting | 5 | Describe the setting, locations, and relevant dates, including periods of recruitment, exposure, follow-up, and data collection | 2 | *A YT search was conducted on 10 April 2021 using a Google Chrome browser in "incognito mode".* |
| Participants | 6 | (*a*) *Cohort study*—Give the eligibility criteria, and the sources and methods of selection of participants. Describe methods of follow-up<br>*Case-control study*—Give the eligibility criteria, and the sources and methods of case ascertainment and control selection. Give the rationale for the choice of cases and controls | 2 | *Duplicates were removed and videos in parts were treated as one production (with views, time, and comments added together). Non-English, "pronunciation psammoma" videos and non-meningioma materials were* |

| | | | | |
|---|---|---|---|---|
| | | *Cross-sectional study*—Give the eligibility criteria, and the sources and methods of selection of participants | | *excluded. Any material on brain tumors in general was assessed only on the part dedicated to meningiomas.* |
| | | (*b*) *Cohort study*—For matched studies, give matching criteria and number of exposed and unexposed<br>*Case-control study*—For matched studies, give matching criteria and the number of controls per case | NA | |
| Variables | 7 | Clearly define all outcomes, exposures, predictors, potential confounders, and effect modifiers. Give diagnostic criteria, if applicable | 2 | *The source of the upload (physician, hospital/clinic channel, health channel, or patient), video duration, and substantive content of every video were analyzed. A comparable protocol of video content was extracted (symptoms, treatment, animations, diagrams, etc.) according to previous studies quantitative information, the "VidIQ Vision for YouTube" plug-in was used. Audience engagement content (likes, dislikes, views, and channel subscriptions) was extracted on 11 December 2021* |
| Data sources/ measurement | 8* | For each variable of interest, give sources of data and details of methods of assessment (measurement). Describe comparability of assessment methods if there is more than one group | *2* | *Videos were from YouTube*<br>*Two neurosurgery specialists with over 15 and 40 years of clinical experience assessed the videos to detect any substantive fallacies* |
| Bias | 9 | Describe any efforts to address potential sources of bias | 2 | *A cleared search history, no Google account attachment, and "relevance based ranking" was used to avoid personalization of the results.*<br>*If any disagreements occurred between the authors, the doubts were discussed in detail.* |

| | | | | |
|---|---|---|---|---|
| Study size | 10 | Explain how the study size was arrived at | 3 | 119 videos |
| Quantitative variables | 11 | Explain how quantitative variables were handled in the analyses. If applicable, describe which groupings were chosen and why | 2 | *DISCERN (Quality Criteria for Consumer Health Information), GQS (Global Quality Score), and JAMA (the Journal of the American Medical Association) scoring systems quantitative information, the "VidIQ Vision for YouTube" plug-in was used. Audience engagement content (likes, dislikes, views, and channel subscriptions)* |
| Statistical methods | 12 | (*a*) Describe all statistical methods, including those used to control for confounding | 3 | *Data were verified with the Shapiro–Wilk test and subsequently analyzed according to the test results. The descriptive statistics included arithmetic mean, median, range, and standard deviation (std).*<br>*Spearman's rank correlation coefficient, the Kruskal–Wallis test with Dunn–Bonferroni post hoc analysis, and Mann–Whitne* |
| | | (*b*) Describe any methods used to examine subgroups and interactions | NA | |
| | | (*c*) Explain how missing data were addressed | NA | |
| | | (*d*) *Cohort study*—If applicable, explain how loss to follow-up was addressed<br>*Case-control study*—If applicable, explain how matching of cases and controls was addressed<br>*Cross-sectional study*—If applicable, describe analytical methods taking account of sampling strategy | NA | |
| | | (*e*) Describe any sensitivity analyses | NA | |
| **Results** | | | | |
| Participants | 13* | (a) Report numbers of individuals at each stage of study—eg numbers potentially eligible, examined for eligibility, confirmed eligible, included in the study, completing follow-up, and analysed | 3 | *After applying the exclusion criteria, 119 videos were analyzed (Figure 1)* |

| | | | | |
|---|---|---|---|---|
| | | (b) Give reasons for non-participation at each stage | NA | |
| | | (c) Consider use of a flow diagram | 3 | Figure 1 |
| Descriptive data | 14* | (a) Give characteristics of study participants (eg demographic, clinical, social) and information on exposures and potential confounders | 3 | *The largest number of videos were provided by health channels (n = 69), followed by hospital/clinic channels (n = 24), physicians (n = 16), and patients (n = 10) (Figure 2).* |
| | | (b) Indicate number of participants with missing data for each variable of interest | NA | |
| | | (c) *Cohort study*—Summarise follow-up time (eg, average and total amount) | NA | |
| Outcome data | 15* | *Cohort study*—Report numbers of outcome events or summary measures over time | | |
| | | *Case-control study*—Report numbers in each exposure category, or summary measures of exposure | | |
| | | *Cross-sectional study*—Report numbers of outcome events or summary measures | 3-7 | *3.1. Video Quality Analysis*<br>*3.2 Misleading information*<br>*3.3. Audience Engagement*<br>*3.4. Top five DISCERN/VPI Videos* |
| Main results | 16 | (*a*) Give unadjusted estimates and, if applicable, confounder-adjusted estimates and their precision (eg, 95% confidence interval). Make clear which confounders were adjusted for and why they were included | 4 | *From the 119 evaluated videos, 47.9% of the videos were graded as "poor", 21.85% achieved "average", 21.01% were "very poor", and only 9.24% were "good".* |
| | | (*b*) Report category boundaries when continuous variables were categorized | NA | |
| | | (*c*) If relevant, consider translating estimates of relative risk into absolute risk for a meaningful time period | NA | |

| | | | | |
|---|---|---|---|---|
| Other analyses | 17 | Report other analyses done—eg analyses of subgroups and interactions, and sensitivity analyses | NA | |
| Key results | 18 | Summarise key results with reference to study objectives | 7 | This study confirms that YT material presented on the topic of meningiomas is also of low |

| | | | | |
|---|---|---|---|---|
| | | | | quality. The mean DISCERN score for these meningiomas videos is classified in the ”poor” bracket, where the information presented to patients seeking knowledge on the most common CNS tumors is not fully reliable. |
| Limitations | 19 | Discuss limitations of the study, taking into account sources of potential bias or imprecision. Discuss both direction and magnitude of any potential bias | 9 | *4.3. Limitations…* |
| Interpretation | 20 | Give a cautious overall interpretation of results considering objectives, limitations, multiplicity of analyses, results from similar studies, and other relevant evidence | 9 | *Much of the current information is misleading, and does not provide a sufficient amount of information for a non-medical viewer to fully understand the most common primary CNS tumors.* |
| Generalisability | 21 | Discuss the generalisability (external validity) of the study results | 9 | *Our analysis only included English videos, with only the first 75 results for every search term. Moreover, in more than 50% of videos, the country of origin was undefinable, due to a lack of data on the video provider channel.* |
| **Other information** | | | | |
| Funding | 22 | Give the source of funding and the role of the funders for the present study and, if applicable, for the original study on which the present article is based | 9 | *Funding: This research received no external funding.* |

Table S146. Quality Evaluation for Included Studies Using STROBE

70.STROBE Statement—checklist of items that should be included in reports of observational studies

| | Item No. | Recommendation | Page No. | Relevant text from manuscript |
|---|---|---|---|---|
| **Title and abstract** | 1 | (*a*) Indicate the study's design with a commonly used term in the title or the abstract | 1 | Systematic review |
| | | (*b*) Provide in the abstract an informative and balanced summary of what was done and what was found | 1 | Methods and Results |
| **Introduction** | | | | |
| Background/rationale | 2 | Explain the scientific background and rationale for the investigation being reported | 2 | *With high Internet use among family caregivers,[20] the quality of information provided via the Internet or other social media outlets is a concern for providers,[21] and there is a need to determine the availability of videos that address the caregiving role.* |
| Objectives | 3 | State specific objectives, including any prespecified hypotheses | 2 | *aimed to evaluate the availability of instructional videos related to managing cancer-related pain through instructional videos on YouTube. As a secondary goal, we aimed to assess whether the scope and design of instructional content included the caregiver's role* |
| **Methods** | | | | |
| Study design | 4 | Present key elements of study design early in the paper | 2 | *systematic review* |
| Setting | 5 | Describe the setting, locations, and relevant dates, including periods of recruitment, exposure, follow-up, and data collection | 2 | *"YouTube videos was conducted on May 9, 2013. Three different searches were entered into …"* |
| Participants | 6 | (*a*) *Cohort study*—Give the eligibility criteria, and the sources and methods of selection of participants. Describe methods of follow-up | 3 | *"To qualify for inclusion in the study, videos had to be in English …"* |

| | | | | |
|---|---|---|---|---|
| | | *Case-control study*—Give the eligibility criteria, and the sources and methods of case ascertainment and control selection. Give the rationale for the choice of cases and controls<br><br>*Cross-sectional study*—Give the eligibility criteria, and the sources and methods of selection of participants | | |
| | | (*b*) *Cohort study*—For matched studies, give matching criteria and number of exposed and unexposed<br><br>*Case-control study*—For matched studies, give matching criteria and the number of controls per case | | |
| Variables | 7 | Clearly define all outcomes, exposures, predictors, potential confounders, and effect modifiers. Give diagnostic criteria, if applicable | 3-4 | Literacy tools use; caregiver role inclusion; quality of information … |
| Data sources/ measurement | 8* | For each variable of interest, give sources of data and details of methods of assessment (measurement). Describe comparability of assessment methods if there is more than one group | *3* | *Videos were from YouTube*<br><br>*two elements of educational video format. Instructional design was assessed using a three-part coding scheme derived from Morain and Swarts*<br><br>*The Federal Plain Language Guidelines published by Plain Language in March 2011[25] were used to identify the use of literacy tools …* |
| Bias | 9 | Describe any efforts to address potential sources of bias | 4 | Three training sessions were held.<br><br>The first author reviewed coding for the overall sample as an additional reliability check, and agreement of two among three coders ensured consistency of the evaluation. |
| Study size | 10 | Explain how the study size was arrived at | 4 | *1118 unique videos.* |
| Quantitative variables | 11 | Explain how quantitative variables were handled in the analyses. If applicable, describe which groupings were chosen and why | 3-4 | Descriptive analysis of categorical features. |

| | | | | |
|---|---|---|---|---|
| Statistical methods | 12 | (*a*) Describe all statistical methods, including those used to control for confounding | 5 | Frequencies, proportions calculated. |
| | | (*b*) Describe any methods used to examine subgroups and interactions | NA | |
| | | (*c*) Explain how missing data were addressed | NA | |
| | | (*d*) *Cohort study*—If applicable, explain how loss to follow-up was addressed<br><br>*Case-control study*—If applicable, explain how matching of cases and controls was addressed<br><br>*Cross-sectional study*—If applicable, describe analytical methods taking account of sampling strategy | NA | |
| | | (*e*) Describe any sensitivity analyses | NA | |
| **Results** | | | | |
| Participants | 13* | (a) Report numbers of individuals at each stage of study—eg numbers potentially eligible, examined for eligibility, confirmed eligible, included in the study, completing follow-up, and analysed | 4 | *From the three key phrase searches, a total of 1500 videos were made available, and after duplications were removed, there were a total of 1118 unique videos.* |
| | | (b) Give reasons for non-participation at each stage | NA | |
| | | (c) Consider use of a flow diagram | 4 | Fig 1 |
| Descriptive data | 14* | (a) Give characteristics of study participants (eg demographic, clinical, social) and information on exposures and potential confounders | 4-6 | *The information on the individual videos used in the study is summarized in Table 1…* |
| | | (b) Indicate number of participants with missing data for each variable of interest | NA | |
| | | (c) *Cohort study*—Summarise follow-up time (eg, average and total amount) | NA | |
| Outcome data | 15* | *Cohort study*—Report numbers of outcome events or summary measures over time | | |
| | | *Case-control study*—Report numbers in each exposure category, or summary measures of exposure | | |
| | | *Cross-sectional study*—Report numbers of outcome events or summary measures | 6-7 | *"As shown in Table 2, most videos were listed under the Education category … negative health-care experiences (2%)."* |

| | | | | |
|---|---|---|---|---|
| Main results | 16 | (*a*) Give unadjusted estimates and, if applicable, confounder-adjusted estimates and their precision (eg, 95% confidence interval). Make clear which confounders were adjusted for and why they were included | 6 | *most videos were listed under the Education category (56%), followed by Science and Technology (28%).* |
| | | (*b*) Report category boundaries when continuous variables were categorized | NA | |
| | | (*c*) If relevant, consider translating estimates of relative risk into absolute risk for a meaningful time period | NA | |
| Other analyses | 17 | Report other analyses done—eg analyses of subgroups and interactions, and sensitivity analyses | NA | |
| Key results | 18 | Summarise key results with reference to study objectives | 7 | *videos were less available on topics of pain management and assessment for cancer patients and caregivers. Most videos resulting from our search did not meet study inclusion criteria because they did not include specific steps or how-to explanations about pain management.* |
| Limitations | 19 | Discuss limitations of the study, taking into account sources of potential bias or imprecision. Discuss both direction and magnitude of any potential bias | 9 | "*Although this study sheds light on the availability of instructional cancer pain management videos as…"* |
| Interpretation | 20 | Give a cautious overall interpretation of results considering objectives, limitations, multiplicity of analyses, results from similar studies, and other relevant evidence | 9 | *Future research will need to address the appropriate length of instructional videos, who should be featured, and how to ensure that literacy tools are used so that pain management instruction is understandable for informal caregivers* |
| Generalisability | 21 | Discuss the generalisability (external validity) of the study results | 9 | *we were not\ able to capture the entire number of videos available through YouTube as the maximum results for any search is restricted to 500 videos by YouTube.* |

| | | | | |
|---|---|---|---|---|
| **Other information** | | | | |
| Funding | 22 | Give the source of funding and the role of the funders for the present study and, if applicable, for the original study on which the present article is based | 10 | *This project was supported by Award Number* |

Table S147. Quality Evaluation for Included Studies Using STROBE

71.STROBE Statement—checklist of items that should be included in reports of observational studies

| | Item No. | Recommendation | Page No. | Relevant text from manuscript |
|---|---|---|---|---|
| **Title and abstract** | 1 | (*a*) Indicate the study's design with a commonly used term in the title or the abstract | 1 | Retrospective review |
| | | (*b*) Provide in the abstract an informative and balanced summary of what was done and what was found | 1 | Methods and Results |
| **Introduction** | | | | |
| Background/rationale | 2 | Explain the scientific background and rationale for the investigation being reported | 2 | *Although there are studies that show the role of YouTube™ in improving patients' attitudes towards cancer [10], the videos are not controlled for both content and quality. While some of this content is uploaded by academic institutions or by physicians, none of the videos are peer reviewed for content validity* |
| Objectives | 3 | State specific objectives, including any prespecified hypotheses | 2 | *we aim to review whether the information currently available on YouTube™ is adequate and thorough by reviewing the videos in the platform with guidance of particular questions.* |
| **Methods** | | | | |
| Study design | 4 | Present key elements of study design early in the paper | 2 | Retrospective cross-sectional |
| Setting | 5 | Describe the setting, locations, and relevant dates, including periods of recruitment, exposure, follow-up, and data collection | 2 | *Two separate YouTube™ searches were performed on January 6, 2019 by using the terms "throat cancer" and "larynx cancer" in default mode* |
| Participants | 6 | (*a*) *Cohort study*—Give the eligibility criteria, and the sources and methods of selection of participants. Describe methods of follow-up | 2 | *Selection of videos …* |

| | | | | |
|---|---|---|---|---|
| | | *Case-control study*—Give the eligibility criteria, and the sources and methods of case ascertainment and control selection. Give the rationale for the choice of cases and controls<br>*Cross-sectional study*—Give the eligibility criteria, and the sources and methods of selection of participants | | |
| | | (*b*) *Cohort study*—For matched studies, give matching criteria and number of exposed and unexposed<br>*Case-control study*—For matched studies, give matching criteria and the number of controls per case | NA | |
| Variables | 7 | Clearly define all outcomes, exposures, predictors, potential confounders, and effect modifiers. Give diagnostic criteria, if applicable | 2-3 | *audiovisual quality, accuracy scores, upload source and education types of the videos, topic domains, usefulness scores and larynx-specific questions items.* |
| Data sources/ measurement | 8* | For each variable of interest, give sources of data and details of methods of assessment (measurement). Describe comparability of assessment methods if there is more than one group | 2 | *Videos were from YouTube*<br>*All three raters watched each video thoroughly and evaluated the content using pre-defined criteria* |
| Bias | 9 | Describe any efforts to address potential sources of bias | 2 | *Analysis of videos*<br>*"If two identical answers and one … the link was not included in the study.* |
| Study size | 10 | Explain how the study size was arrived at | 3 | *152 videos* |

| | | | | |
|---|---|---|---|---|
| Quantitative variables | 11 | Explain how quantitative variables were handled in the analyses. If applicable, describe which groupings were chosen and why | 2-3 | *audiovisual quality, accuracy scores, usefulness scores* |
| Statistical methods | 12 | (*a*) Describe all statistical methods, including those used to control for confounding | 3 | *Statistical analysis paragraph 2-4* |
| | | (*b*) Describe any methods used to examine subgroups and interactions | 3 | *A Fisher's exact test (2 Å~ c) was used to determine if there were differences* |
| | | (*c*) Explain how missing data were addressed | NA | |

| | | | | |
|---|---|---|---|---|
| | | (*d*) *Cohort study*—If applicable, explain how loss to follow-up was addressed<br><br>*Case-control study*—If applicable, explain how matching of cases and controls was addressed<br><br>*Cross-sectional study*—If applicable, describe analytical methods taking account of sampling strategy | NA | |
| | | (*e*) Describe any sensitivity analyses | NA | |
| **Results** | | | | |
| Participants | 13* | (a) Report numbers of individuals at each stage of study—eg numbers potentially eligible, examined for eligibility, confirmed eligible, included in the study, completing follow-up, and analysed | 3 | *After excluding the videos that did not meet the criteria (n = 28) or could not be categorized by type or uploader (n = 20), a total of 152 videos were included in the study. The shortest video lasted 10 s and the longest was 68 min. Furthermore, while the most watched video had 619,9426 views, the lowest view count for a video was 8.* |
| | | (b) Give reasons for non-participation at each stage | NA | |
| | | (c) Consider use of a flow diagram | NA | |
| Descriptive data | 14* | (a) Give characteristics of study participants (eg demographic, clinical, social) and information on exposures and potential confounders | 3 | *the testimonial and educational groups consisted of 64 and 88 videos, respectively. After sub-categorization of the videos by the uploader, the health-care group included 82 videos, while the university, individual users, TV channel/news and undetermined groups included 11, 36, 12 and 11 videos, respectively.* |
| | | (b) Indicate number of participants with missing data for each variable of interest | | |
| | | (c) *Cohort study*—Summarise follow-up time (eg, average and total amount) | | |
| Outcome data | 15* | *Cohort study*—Report numbers of outcome events or summary measures over time | | |
| | | *Case-control study*—Report numbers in each exposure category, or summary measures of exposure | | |
| | | *Cross-sectional study*—Report numbers of outcome events or summary measures | *3-4* | *Results paragraph 3- …* |

| | | | | |
|---|---|---|---|---|
| Main results | 16 | (*a*) Give unadjusted estimates and, if applicable, confounder-adjusted estimates and their precision (eg, 95% confidence interval). Make clear which confounders were adjusted for and why they were included | 4 | *The median value of usefulness score for educational videos (3.00) was statistically significantly higher than for testimonial videos (1.00, p < 0.001, U = 1.423).* |
| | | (*b*) Report category boundaries when continuous variables were categorized | NA | |
| | | (*c*) If relevant, consider translating estimates of relative risk into absolute risk for a meaningful time period | NA | |
| Other analyses | 17 | Report other analyses done—eg analyses of subgroups and interactions, and sensitivity analyses | 5 | *There were statistically significant difference … between the groups based on the education type, …* |
| Key results | 18 | Summarise key results with reference to study objectives | 7 | *The heterogeneity of the videos available on You- Tube™ is vast, and these materials differ in quality, accuracy and content. Our results indicate that the audiovisual quality of videos uploaded by a university-affiliated account is significantly higher than those uploaded by health-care institutions and individual users.* |
| Limitations | 19 | Discuss limitations of the study, taking into account sources of potential bias or imprecision. Discuss both direction and magnitude of any potential bias | 8 | *The limitations of our study include that we only …* |
| Interpretation | 20 | Give a cautious overall interpretation of results considering objectives, limitations, multiplicity of analyses, results from similar studies, and other relevant evidence | 8 | *To increase the benefits of online sources and to reduce information pollution by misleading data, patient information videos discussing common health problems should be prepared and disseminated only by universities or health-care institutions.* |
| Generalisability | 21 | Discuss the generalisability (external validity) of the study results | 8 | *given that YouTube™ is not the only platform that the public can use to access videos, further* |

| | | | | |
|---|---|---|---|---|
| | | | | *studies that evaluate and compare other social media platforms should be planned* |
| **Other information** | | | | |
| Funding | 22 | Give the source of funding and the role of the funders for the present study and, if applicable, for the original study on which the present article is based | 8 | *Funding No funding was taken for this study.* |

Table S148. Quality Evaluation for Included Studies Using STROBE

72.STROBE Statement—checklist of items that should be included in reports of observational studies

| | Item No. | Recommendation | Page No. | Relevant text from manuscript |
|---|---|---|---|---|
| **Title and abstract** | 1 | (*a*) Indicate the study's design with a commonly used term in the title or the abstract | 1 | Quality assessment |
| | | (*b*) Provide in the abstract an informative and balanced summary of what was done and what was found | 1 | Methods and Results |
| **Introduction** | | | | |
| Background/rationale | 2 | Explain the scientific background and rationale for the investigation being reported | 2 | *The use of the Internet as a source of information about health problems and treatments has increased in recent years, and YouTube is a widely used effective tool to convey medical information (Liu et al., 2020). However, YouTube is primarily a commercial platform, and videos uploaded are only examined for copyright infringement.* |
| Objectives | 3 | State specific objectives, including any prespecified hypotheses | 2 | *evaluating the reliability, quality and content of the most watched YouTube videos in English language concerning shoulder/ arm exercises that can be undertaken after BC treatment.* |
| **Methods** | | | | |
| Study design | 4 | Present key elements of study design early in the paper | 2 | *descriptive study* |
| Setting | 5 | Describe the setting, locations, and relevant dates, including periods of recruitment, exposure, follow-up, and data collection | 2 | Based on the results of this preliminary search, the terms 'shoulder exercise and BC surgery', 'arm exercise and BC surgery' and 'physiotherapy/physical therapy and BC surgery' were selected. The search |

| | | | | |
|---|---|---|---|---|
| | | | | was performed on 10 February 2021 |
| Participants | 6 | (*a*) *Cohort study*—Give the eligibility criteria, and the sources and methods of selection of participants. Describe methods of follow-up<br>*Case-control study*—Give the eligibility criteria, and the sources and methods of case ascertainment and control selection. Give the rationale for the choice of cases and controls<br>*Cross-sectional study*—Give the eligibility criteria, and the sources and methods of selection of participants | 2 | *Videos that were not relevant to the topic, those in a non-English language and those without sound were excluded from the study. Duplicated and multipart videos were counted as a single video* |
| | | (*b*) *Cohort study*—For matched studies, give matching criteria and number of exposed and unexposed<br>*Case-control study*—For matched studies, give matching criteria and the number of controls per case | NA | |
| Variables | 7 | Clearly define all outcomes, exposures, predictors, potential confounders, and effect modifiers. Give diagnostic criteria, if applicable | 3 | *length, total views, time since upload and daily views were recorded. The number of likes, dislikes and comments related to the interaction level of the videos were determined* |
| Data sources/ measurement | 8* | For each variable of interest, give sources of data and details of methods of assessment (measurement). Describe comparability of assessment methods if there is more than one group | *2* | *Videos were from YouTube*<br>*each search term were evaluated independently by a surgeon (PB) and a physiotherapist (SG) experienced in BC surgery and post-surgical training programmes, respectively.* |
| Bias | 9 | Describe any efforts to address potential sources of bias | 3 | *In case of inconsistency in the process of the video scoring of two researchers, the opinion of a third independent researcher (a medical doctor specialised in physical therapy) (ST) was sought in order to evaluate and finalise the video.* |
| Study size | 10 | Explain how the study size was arrived at | 4 | 82 videos |

| | | | | |
|---|---|---|---|---|
| Quantitative variables | 11 | Explain how quantitative variables were handled in the analyses. If applicable, describe which groupings were chosen and why | 3 | *DISCERN, Global Quality Scale, Comprehensiveness* |
| Statistical methods | 12 | (*a*) Describe all statistical methods, including those used to control for confounding | 4 | *Descriptive analyses were presented as median [interquartile range (IQR)], and percentages (%) for continuous variables.*<br>*Categorical variables are expressed as counts and percentages. The results were statistically analysed using Kruskal–Wallis, a nonparametric one-way analysis. Dunn's post hoc multiple comparison test was used to calculate pairwise comparisons between groups.* |
| | | (*b*) Describe any methods used to examine subgroups and interactions | 4 | *Dunn's post hoc multiple comparison test was used to calculate pairwise comparisons between groups.* |
| | | (*c*) Explain how missing data were addressed | NA | |
| | | (*d*) *Cohort study*—If applicable, explain how loss to follow-up was addressed<br>*Case-control study*—If applicable, explain how matching of cases and controls was addressed<br>*Cross-sectional study*—If applicable, describe analytical methods taking account of sampling strategy | NA | |
| | | (*e*) Describe any sensitivity analyses | NA | |
| **Results** | | | | |
| Participants | 13* | (a) Report numbers of individuals at each stage of study—eg numbers potentially eligible, examined for eligibility, confirmed eligible, included in the study, completing follow-up, and analysed | 4 | *A total of 16,462 videos were obtained using determined search terms, and the 180 most viewed videos were included in the study.*<br>*Of these videos, 82 were found to meet our criteria and included in the sample for further analysis. Included videos, 40 were evaluated in the useful information category and 42 in* |

| | | | | |
|---|---|---|---|---|
| | | | | *the misleading information category. All information about video numbers is in Figure 1. The kappa statistic for inter-observer agreement was 0.79* |
| | | (b) Give reasons for non-participation at each stage | NA | |
| | | (c) Consider use of a flow diagram | 4 | Figure 1 |
| Descriptive data | 14* | (a) Give characteristics of study participants (eg demographic, clinical, social) and information on exposures and potential confounders | 4 | *The data of the related videos were classified according to the level of usefulness, and the results are given in Table 2.* |
| | | (b) Indicate number of participants with missing data for each variable of interest | NA | |
| | | (c) *Cohort study*—Summarise follow-up time (eg, average and total amount) | NA | |
| Outcome data | 15* | *Cohort study*—Report numbers of outcome events or summary measures over time | | |
| | | *Case-control study*—Report numbers in each exposure category, or summary measures of exposure | | |
| | | *Cross-sectional study*—Report numbers of outcome events or summary measures | *4* | *When the useful and misleading videos were compared, … videos uploaded from reliable sources ($p < 0.05$) (Table 4).* |
| Main results | 16 | (*a*) Give unadjusted estimates and, if applicable, confounder-adjusted estimates and their precision (eg, 95% confidence interval). Make clear which confounders were adjusted for and why they were included | 4 | *The data of the related videos were classified according to the level of usefulness, and the results are given in Table 2. When the useful and misleading videos were compared, a statistically significant difference ($p < 0.05$) was found in favour of the useful videos in relation to the number of views, video length, YouTube upload time, number of dislikes, reliability, GQS score and comprehensiveness.* |
| | | (*b*) Report category boundaries when continuous variables were categorized | NA | |
| | | (*c*) If relevant, consider translating estimates of relative risk into absolute risk for a meaningful time period | NA | |

| | | | | |
|---|---|---|---|---|
| Other analyses | 17 | Report other analyses done—eg analyses of subgroups and interactions, and sensitivity analyses | 5 | *Pairwise comparisons showed a statistically significant difference in the number of daily views, number of likes, reliability, GQS score and comprehensiveness in favour of the videos uploaded from reliable sources ($p < 0.05$) (Table 4).* |
| Key results | 18 | Summarise key results with reference to study objectives | 6 | This distribution shows that about half the video content encountered by YouTube users may contain misleading information, and it may not be easy to access accurate information. |
| Limitations | 19 | Discuss limitations of the study, taking into account sources of potential bias or imprecision. Discuss both direction and magnitude of any potential bias | 7 | *Our study had certain limitations….* |
| Interpretation | 20 | Give a cautious overall interpretation of results considering objectives, limitations, multiplicity of analyses, results from similar studies, and other relevant evidence | 7 | *in order to better guide their patients, healthcare professionals should be aware that the control mechanism of YouTube videos is not sufficient and they may contain inaccurate information so they should better address patients to pay attention to the source of the video.* |
| Generalisability | 21 | Discuss the generalisability (external validity) of the study results | 7 | *However, individuals can use different keywords, and this can be considered as a limitation.*<br>*Lastly, the search language was only English* |
| **Other information** | | | | |
| Funding | 22 | Give the source of funding and the role of the funders for the present study and, if applicable, for the original study on which the present article is based | NA | |

Table S149. Quality Evaluation for Included Studies Using STROBE

73.STROBE Statement—checklist of items that should be included in reports of observational studies

| | Item No. | Recommendation | Page No. | Relevant text from manuscript |
|---|---|---|---|---|
| **Title and abstract** | 1 | (*a*) Indicate the study's design with a commonly used term in the title or the abstract | 1 | Cross-sectional: *search engine was queried* |
| | | (*b*) Provide in the abstract an informative and balanced summary of what was done and what was found | 1 | *"YouTube's search engine was queried with different search phrases relating to CRC … the unique parameter associated with a high score on the criteria list."* |
| **Introduction** | | | | |
| Background/rationale | 2 | Explain the scientific background and rationale for the investigation being reported | 1-2 | *YouTube videos contain misleading or biased information and highlighted the importance of designing interventions to guide eHealth consumers*<br>*Wasserman et al. found that sources were frequently incomplete and that their quality was variable* |
| Objectives | 3 | State specific objectives, including any prespecified hypotheses | 2 | *The purpose of this study was to describe the characteristics of YouTube videos and to assess their quality as patient education resources for colorectal cancer.* |
| **Methods** | | | | |
| Study design | 4 | Present key elements of study design early in the paper | 2 | Cross-sectional: *The search was conducted on March 3, 2018.* |
| Setting | 5 | Describe the setting, locations, and relevant dates, including periods of recruitment, exposure, follow-up, and data collection | 2 | *search engine was queried with three distinct key phrases in order to identify relevant videos: Bcolorectal cancer,^ Bcolon cancer,^ and Bbowel cancer.^ The search* |

| | | | | |
|---|---|---|---|---|
| | | | | *was conducted on March 3, 2018.* |
| Participants | 6 | (*a*) *Cohort study*—Give the eligibility criteria, and the sources and methods of selection of participants. Describe methods of follow-up<br><br>*Case-control study*—Give the eligibility criteria, and the sources and methods of case ascertainment and control selection. Give the rationale for the choice of cases and controls<br><br>*Cross-sectional study*—Give the eligibility criteria, and the sources and methods of selection of participants | 2 | *Exclusion criteria included videos not in English, news stories, patient testimonials, duration > 15 min, and duplicates. The study on YouTube prostate cancer videos only studied videos shorter than 10 min* |
| | | (*b*) *Cohort study*—For matched studies, give matching criteria and number of exposed and unexposed<br><br>*Case-control study*—For matched studies, give matching criteria and the number of controls per case | NA | |
| Variables | 7 | Clearly define all outcomes, exposures, predictors, potential confounders, and effect modifiers. Give diagnostic criteria, if applicable | 2 | *Methods paragraph 2*<br><br>*“Many methodological elements frequently collected in YouTube studies can be classified as popularity-driven measures or heuristic-driven measures…”* |
| Data sources/ measurement | 8* | For each variable of interest, give sources of data and details of methods of assessment (measurement). Describe comparability of assessment methods if there is more than one group | *2* | *Videos were from YouTube*<br><br>*Two attending physicians practicing in a quaternary center and a student in his third year of medical studies devised a list of assessment criteria for the videos. Similar to other studies* |
| Bias | 9 | Describe any efforts to address potential sources of bias | 3 | *Paragraph 1*<br><br>*“The two physicians also independently evaluated a sample of videos. To assess total score reliability, the intraclass … the three raters was 0.72, showing good agreement.* |

| | | | | |
|---|---|---|---|---|
| Study size | 10 | Explain how the study size was arrived at | 3 | 46 videos |
| Quantitative variables | 11 | Explain how quantitative variables were handled in the analyses. If applicable, describe which groupings were chosen and why | 2 | *a point-based rating tool was constructed. This tool had a total of 12 evaluation criteria that were each given the rating of 0 or 1 depending on the satisfaction of the assessment criterion.* |
| Statistical methods | 12 | (*a*) Describe all statistical methods, including those used to control for confounding | 3 | *Bivariate analysis using Mann-Whitney U tests was performed to determine differences in videos classified as useful* |
| | | (*b*) Describe any methods used to examine subgroups and interactions | NA | |
| | | (*c*) Explain how missing data were addressed | NA | |
| | | (*d*) *Cohort study*—If applicable, explain how loss to follow-up was addressed<br>*Case-control study*—If applicable, explain how matching of cases and controls was addressed<br>*Cross-sectional study*—If applicable, describe analytical methods taking account of sampling strategy | NA | |
| | | (*e*) Describe any sensitivity analyses | NA | |
| **Results** | | | | |
| Participants | 13* | (a) Report numbers of individuals at each stage of study—eg numbers potentially eligible, examined for eligibility, confirmed eligible, included in the study, completing follow-up, and analysed | 3 | *Our study included 46 videos. Of the identified videos using the specified search phrases, 27% were patient testimonials. Figure 1 presents the screening process.* |
| | | (b) Give reasons for non-participation at each stage | NA | |
| | | (c) Consider use of a flow diagram | 3 | Fig 1 |
| Descriptive data | 14* | (a) Give characteristics of study participants (eg demographic, clinical, social) and information on exposures and potential confounders | 3 | Results paragraph 2<br>*Table 1 summarizes the descriptive statistics measures …* |

| | | | | |
|---|---|---|---|---|
| | | (b) Indicate number of participants with missing data for each variable of interest | NA | |
| | | (c) *Cohort study*—Summarise follow-up time (eg, average and total amount) | NA | |
| Outcome data | 15* | *Cohort study*—Report numbers of outcome events or summary measures over time | | |
| | | *Case-control study*—Report numbers in each exposure category, or summary measures of exposure | | |
| | | *Cross-sectional study*—Report numbers of outcome events or summary measures | *3-4* | *Results paragraph 3-7* |
| Main results | 16 | (*a*) Give unadjusted estimates and, if applicable, confounder-adjusted estimates and their precision (eg, 95% confidence interval). Make clear which confounders were adjusted for and why they were included | 3 | *less than a quarter of videos (24%) satisfied at least half of the established criteria, with the mode being three. A single video could satisfy all 12 criteria.* |
| | | (*b*) Report category boundaries when continuous variables were categorized | NA | |
| | | (*c*) If relevant, consider translating estimates of relative risk into absolute risk for a meaningful time period | NA | |
| Other analyses | 17 | Report other analyses done—eg analyses of subgroups and interactions, and sensitivity analyses | 4 | *misleading videos have more views, are rated more frequently by viewers, and have lower total scores.* |
| Key results | 18 | Summarise key results with reference to study objectives | 4 | *there is a sparse number of comprehensive videos on colorectal cancer on YouTube and that none of the popularity parameters are associated with comprehensiveness of the videos.* |
| Limitations | 19 | Discuss limitations of the study, taking into account sources of potential bias or imprecision. Discuss both direction and magnitude of any potential bias | 5 | *There are limitations to our study…* |
| Interpretation | 20 | Give a cautious overall interpretation of results considering objectives, limitations, multiplicity of analyses, results from similar studies, and other relevant evidence | 5 | *If integrated appropriately in a clinical setting, YouTube could act as an effective userfriendly learning interface for colorectal cancer patients and their families.* |

| | | | | |
|---|---|---|---|---|
| Generalisability | 21 | Discuss the generalisability (external validity) of the study results | 5 | *Our study is crosssectional in nature and might not reflect the evolving nature of the site.*<br><br>*our study was limited to English videos.* |
| **Other information** | | | | |
| Funding | 22 | Give the source of funding and the role of the funders for the present study and, if applicable, for the original study on which the present article is based | 5 | *Conflicts of Interest The authors declare that they have no conflict of*<br><br>*interest.* |

Table S150. Quality Evaluation for Included Studies Using STROBE

74.STROBE Statement—checklist of items that should be included in reports of observational studies

| | Item No. | Recommendation | Page No. | Relevant text from manuscript |
|---|---|---|---|---|
| **Title and abstract** | 1 | (*a*) Indicate the study's design with a commonly used term in the title or the abstract | 1 | Retrospective cross-sectional |
| | | (*b*) Provide in the abstract an informative and balanced summary of what was done and what was found | 1 | Methods and Results<br>"*We searched YouTube for videos relating … with some videos having up to 10 such statements*" |
| **Introduction** | | | | |
| Background/rationale | 2 | Explain the scientific background and rationale for the investigation being reported | 1-2 | *Six in ten Americans turned to the Internet in the past year seeking health information for either themselves or someone they know studies are finding that YouTube is lacking key information that can better inform patients of their condition* |
| Objectives | 3 | State specific objectives, including any prespecified hypotheses | NA | |
| **Methods** | | | | |
| Study design | 4 | Present key elements of study design early in the paper | 2 | Retrospective cross-sectional |
| Setting | 5 | Describe the setting, locations, and relevant dates, including periods of recruitment, exposure, follow-up, and data collection | 2 | *search on 1/29/19 of YouTube, for the term "breast cancer". The search was designed using quotation marks as Boolean operators to provide a specific search for videos that pertain to only "breast cancer" and not simply "breast" OR "cancer".* |
| Participants | 6 | (*a*) *Cohort study*—Give the eligibility criteria, and the sources and methods of selection of participants. Describe methods of follow-up | 2 | *Videos that were not directly related to the clinical aspects of breast cancer, such as music videos and some* |

| | | | | |
|---|---|---|---|---|
| | | *Case-control study*—Give the eligibility criteria, and the sources and methods of case ascertainment and control selection. Give the rationale for the choice of cases and controls<br><br>*Cross-sectional study*—Give the eligibility criteria, and the sources and methods of selection of participants | | *breast cancer awareness campaign videos, were excluded.*<br><br>*Based on YouTube's definition of a "short video", videos longer than 20 minutes were excluded. Finally, duplicate videos were excluded as well as those that were unavailable in English, either narrated or subtitled* |
| | | (*b*) *Cohort study*—For matched studies, give matching criteria and number of exposed and unexposed<br><br>*Case-control study*—For matched studies, give matching criteria and the number of controls per case | NA | |
| Variables | 7 | Clearly define all outcomes, exposures, predictors, potential confounders, and effect modifiers. Give diagnostic criteria, if applicable | 4 | *Scoring system of YouTube "breast cancer" videos*<br><br>*Risk factors, classification, symptom …* |
| Data sources/ measurement | 8* | For each variable of interest, give sources of data and details of methods of assessment (measurement). Describe comparability of assessment methods if there is more than one group | *2* | *Videos were from YouTube*<br><br>*The rubric was developed in collaboration with 4 resident physicians and an attending board certified in obstetrics and gynecology* |
| Bias | 9 | Describe any efforts to address potential sources of bias | 3 | *Five investigators independently and blindly evaluated each video for the items in the rubric using a pilottested Google Form. In order to limit extreme interevaluator scores, the maximum and minimum scores for each video were excluded and the remaining three scores were used to calculate a "trimmed mean".* |
| Study size | 10 | Explain how the study size was arrived at | 3 | 133 videos |

| | | | | |
|---|---|---|---|---|
| Quantitative variables | 11 | Explain how quantitative variables were handled in the analyses. If applicable, describe which groupings were chosen and why | 4 | *Final score and mid part of continuous values* |
| Statistical methods | 12 | (*a*) Describe all statistical methods, including those used to control for confounding | 3 | *Descriptive statistics and t-test* |
| | | (*b*) Describe any methods used to examine subgroups and interactions | NA | |
| | | (*c*) Explain how missing data were addressed | NA | |
| | | (*d*) *Cohort study*—If applicable, explain how loss to follow-up was addressed<br>*Case-control study*—If applicable, explain how matching of cases and controls was addressed<br>*Cross-sectional study*—If applicable, describe analytical methods taking account of sampling strategy | NA | |
| | | (*e*) Describe any sensitivity analyses | NA | |
| **Results** | | | | |
| Participants | 13* | (a) Report numbers of individuals at each stage of study—eg numbers potentially eligible, examined for eligibility, confirmed eligible, included in the study, completing follow-up, and analysed | 3 | *Of the top-200 most-viewed YouTube videos about breast cancer, 133 were included in our sample. 26 videos were irrelevant to the topic, 20 videos were unavailable with English narrating or subtitles, 18 were longer than 20 minutes in length, 2 were duplicates and 1 was removed from YouTube* |
| | | (b) Give reasons for non-participation at each stage | NA | |
| | | (c) Consider use of a flow diagram | 2 | Fig 1 *Prisma diagram.* |
| Descriptive data | 14* | (a) Give characteristics of study participants (eg demographic, clinical, social) and information on exposures and potential confounders | 3 | *The number of views in our sample ranged from 39,010 to 7,583,240 (mean 547,572, median 274,496). The number of likes in our sample ranged from 0 to 226,000 (mean 3874, median 1100). Mean video length was 544 seconds (standard deviation 877 seconds).* |
| | | (b) Indicate number of participants with missing data for each variable of interest | NA | |

| | | | | |
|---|---|---|---|---|
| | | (c) *Cohort study*—Summarise follow-up time (eg, average and total amount) | NA | |
| Outcome data | 15* | *Cohort study*—Report numbers of outcome events or summary measures over time | | |
| | | *Case-control study*—Report numbers in each exposure category, or summary measures of exposure | | |
| | | *Cross-sectional study*—Report numbers of outcome events or summary measures | *3* | *Results paragraph 2-4* |
| Main results | 16 | (*a*) Give unadjusted estimates and, if applicable, confounder-adjusted estimates and their precision (eg, 95% confidence interval). Make clear which confounders were adjusted for and why they were included | 3 | *only 30/133 videos (22.56%) averaged greater than 7 points by the trimmed mean, which would be less than 1/3 of the possible points available by our scoring system.* |
| | | (*b*) Report category boundaries when continuous variables were categorized | NA | |
| | | (*c*) If relevant, consider translating estimates of relative risk into absolute risk for a meaningful time period | NA | |
| Other analyses | 17 | Report other analyses done—eg analyses of subgroups and interactions, and sensitivity analyses | 3 | Based on producer to see the difference |
| Key results | 18 | Summarise key results with reference to study objectives | 3 | *Less than 25% of the videos in our sample received a score of 7 or higher, representing less than 1/3 of the possible points available.* |
| Limitations | 19 | Discuss limitations of the study, taking into account sources of potential bias or imprecision. Discuss both direction and magnitude of any potential bias | 5 | *Our study contains several strengths and limitations….* |
| Interpretation | 20 | Give a cautious overall interpretation of results considering objectives, limitations, multiplicity of analyses, results from similar studies, and other relevant evidence | 6 | *While YouTube is an important source of information regarding breast cancer, few contain a significant amount of quality information and many of the videos contain false or misleading statements.* |
| Generalisability | 21 | Discuss the generalisability (external validity) of the study results | 5 | *Our results are not intended to generalize beyond the videos composing our sample. It is possible that other videos outside of our sample may* |

| | | | | |
|---|---|---|---|---|
| | | | | *contain either more or less evidence-based information regarding breast cancer.* |
| **Other information** | | | | |
| Funding | 22 | Give the source of funding and the role of the funders for the present study and, if applicable, for the original study on which the present article is based | 6 | *All authors certify that they are free from any conflicts of interest.* |

Table S151. Quality Evaluation for Included Studies Using STROBE

75.STROBE Statement—checklist of items that should be included in reports of observational studies

| | Item No. | Recommendation | Page No. | Relevant text from manuscript |
|---|---|---|---|---|
| **Title and abstract** | 1 | (*a*) Indicate the study's design with a commonly used term in the title or the abstract | NA | |
| | | (*b*) Provide in the abstract an informative and balanced summary of what was done and what was found | 1 | Methods and Results<br>"*We examined 62 still-images and 64 video Instagram posts using … misinformation. Most posts had comments offering social support*" |
| **Introduction** | | | | |
| Background/rationale | 2 | Explain the scientific background and rationale for the investigation being reported | 1 | *Struck et al reported that over a 1-month time period in 2016, there were 1081 posts about prostate cancer on Instagram, which reached more than 4 million viewers [1]. However, they did not characterize the quality of information that was being shared.* |
| Objectives | 3 | State specific objectives, including any prespecified hypotheses | 1 | *Our objective was to evaluate the quality of Instagram posts about PCa using validated criteria.* |
| **Methods** | | | | |
| Study design | 4 | Present key elements of study design early in the paper | 1 | Cross-sectional study |
| Setting | 5 | Describe the setting, locations, and relevant dates, including periods of recruitment, exposure, follow-up, and data collection | 1 | *examined 150 posts (75 still-image posts with the most likes and 75 video posts with the most views listed as "hot") for the hashtag #prostatecancer on Instagram as of 5/18/20* |
| Participants | 6 | (*a*) *Cohort study*—Give the eligibility criteria, and the sources and methods of selection of participants. Describe methods of follow-up | 1 | *(75 still-image posts with the most likes and 75 video posts with the most views listed as "hot") for the* |

| | | | | |
|---|---|---|---|---|
| | | *Case-control study*—Give the eligibility criteria, and the sources and methods of case ascertainment and control selection. Give the rationale for the choice of cases and controls<br>*Cross-sectional study*—Give the eligibility criteria, and the sources and methods of selection of participants | | *hashtag #prostatecancer on Instagram Private and non-English posts were excluded, leaving 62 images and 64 videos for analysis.* |
| | | (*b*) *Cohort study*—For matched studies, give matching criteria and number of exposed and unexposed<br>*Case-control study*—For matched studies, give matching criteria and the number of controls per case | NA | |
| Variables | 7 | Clearly define all outcomes, exposures, predictors, potential confounders, and effect modifiers. Give diagnostic criteria, if applicable | 1 | *Basic characteristics of the content and perceived demographics of publishers the DISCERN instrument for the quality of consumer health information and the PEMAT tool for understandability and actionability [6, 7]. We also rated misinformation compared to guidelines using a published 5-point Likert scale* |
| Data sources/ measurement | 8* | For each variable of interest, give sources of data and details of methods of assessment (measurement). Describe comparability of assessment methods if there is more than one group | *1* | *Posts were from Instagram*<br>*Content was reviewed by experts (physicians with clinical expertise in prostate cancer and experience conducting research on the topic of social media and healthcare)* |
| Bias | 9 | Describe any efforts to address potential sources of bias | 1 | *Inter-rater reliability was 97.2% between coders. Private and non-English posts were excluded* |
| Study size | 10 | Explain how the study size was arrived at | 1 | 126 posts (62 images and 64 videos) |
| Quantitative variables | 11 | Explain how quantitative variables were handled in the analyses. If applicable, describe which groupings were chosen and why | 1 | *DISCERN instrument for the quality of consumer health* |

| | | | | |
|---|---|---|---|---|
| | | | | *information and the PEMAT tool for understandability and actionability [6, 7]. We also rated misinformation compared to guidelines using a published 5-point Likert scale* |
| Statistical methods | 12 | (*a*) Describe all statistical methods, including those used to control for confounding | 1 | Descriptive statistics used; proportions reported. |
| | | (*b*) Describe any methods used to examine subgroups and interactions | NA | |
| | | (*c*) Explain how missing data were addressed | NA | |
| | | (*d*) *Cohort study*—If applicable, explain how loss to follow-up was addressed<br>*Case-control study*—If applicable, explain how matching of cases and controls was addressed<br>*Cross-sectional study*—If applicable, describe analytical methods taking account of sampling strategy | NA | |
| | | (*e*) Describe any sensitivity analyses | NA | |
| **Results** | | | | |
| Participants | 13* | (a) Report numbers of individuals at each stage of study—eg numbers potentially eligible, examined for eligibility, confirmed eligible, included in the study, completing follow-up, and analysed | 1 | Basic characteristics of the content and perceived demographics of publishers were examined as in previous studies |
| | | (b) Give reasons for non-participation at each stage | NA | |
| | | (c) Consider use of a flow diagram | NA | |
| Descriptive data | 14* | (a) Give characteristics of study participants (eg demographic, clinical, social) and information on exposures and potential confounders | 1 | Basic characteristics of the content and perceived demographics of publishers were examined as in previous studies |
| | | (b) Indicate number of participants with missing data for each variable of interest | NA | |
| | | (c) *Cohort study*—Summarise follow-up time (eg, average and total amount) | NA | |
| Outcome data | 15* | *Cohort study*—Report numbers of outcome events or summary measures over time | *1* | *Results* |

| | | | | |
|---|---|---|---|---|
| | | | | *"As showed in Fig.1, 90% … intended behavior change due to viewing the content."* |
| | | *Case-control study*—Report numbers in each exposure category, or summary measures of exposure | *NA* | |
| | | *Cross-sectional study*—Report numbers of outcome events or summary measures | *NA* | |
| Main results | 16 | (*a*) Give unadjusted estimates and, if applicable, confounder-adjusted estimates and their precision (eg, 95% confidence interval). Make clear which confounders were adjusted for and why they were included | 1 | *90% of content was scored as low to moderate quality 40% contained moderate to high amounts of misinformation.* |
| | | (*b*) Report category boundaries when continuous variables were categorized | NA | |
| | | (*c*) If relevant, consider translating estimates of relative risk into absolute risk for a meaningful time period | NA | |
| Other analyses | 17 | Report other analyses done—eg analyses of subgroups and interactions, and sensitivity analyses | NA | |
| Key results | 18 | Summarise key results with reference to study objectives | 1 | *Instagram content about PCa is easily understood but is generally of low-to-moderate quality and poorly actionable for health consumers.* |
| Limitations | 19 | Discuss limitations of the study, taking into account sources of potential bias or imprecision. Discuss both direction and magnitude of any potential bias | 2 | *Limitations of our study include the challenge…* |
| Interpretation | 20 | Give a cautious overall interpretation of results considering objectives, limitations, multiplicity of analyses, results from similar studies, and other relevant evidence | 2 | *We suggest that healthcare providers recommend outside sources of information to patients and their families and take an active role on social media networks* |
| Generalisability | 21 | Discuss the generalisability (external validity) of the study results | 2 | *we cannot assume that only patients with prostate cancer themselves are seeking information via social media, as family members and friends may also seek information.* |
| **Other information** | | | | |

| | | | | |
|---|---|---|---|---|
| Funding | 22 | Give the source of funding and the role of the funders for the present study and, if applicable, for the original study on which the present article is based | 3 | *The authors declare no competing interests.* |

Table S152. Ethical approval process report

| Paper No. | Ethnical approval |
|---|---|
| 1 | yes |
| 2 | yes |
| 3 | no |
| 4 | no |
| 5 | yes |
| 6 | no |
| 7 | no |
| 8 | yes |
| 9 | no |
| 10 | no |
| 11 | yes |
| 12 | no |
| 13 | yes |
| 14 | yes |
| 15 | no |
| 16 | yes |
| 17 | no |
| 18 | no |
| 19 | no |
| 20 | yes |
| 21 | yes |
| 22 | yes |
| 23 | yes |
| 24 | no |
| 25 | no |
| 26 | yes |
| 27 | no |
| 28 | no |
| 29 | yes |
| 30 | yes |
| 31 | no |
| 32 | no |
| 33 | yes |
| 34 | yes |
| 35 | no |
| 36 | yes |
| 37 | no |
| 38 | no |
| 39 | yes |
| 40 | yes |

| | |
|---|---|
| 41 | yes |
| 42 | no |
| 43 | no |
| 44 | yes |
| 45 | yes |
| 46 | no |
| 47 | yes |
| 48 | no |
| 49 | yes |
| 50 | yes |
| 51 | no |
| 52 | no |
| 53 | no |
| 54 | yes |
| 55 | yes |
| 56 | no |
| 57 | yes |
| 58 | no |
| 59 | no |
| 60 | yes |
| 61 | no |
| 62 | no |
| 63 | no |
| 64 | yes |
| 65 | no |
| 66 | no |
| 67 | no |
| 68 | yes |
| 69 | no |
| 70 | no |
| 71 | yes |
| 72 | yes |
| 73 | no |
| 74 | no |
| 75 | no |

Table S153. Assessments of certainty of evidence using GRADE

## Dissemination via social media / AI-chatbots compared to NA for Addressing Cancer-related information

| Certainty assessment | | | | | | |
|---|---|---|---|---|---|---|
| **Participants (studies) Follow-up** | **Risk of bias** | **Inconsistency** | **Indirectness** | **Imprecision** | **Publication bias** | **Overall certainty of evidence** |
| **Overall quality via DISCERN (assessed with: DISCERN)** | | | | | | |
| 3701 (44 non-randomised studies) | not serious | very serious[a] | not serious | serious[b] | none | ⊕◯◯◯ Very low[a,b] |
| **Overall quality via GQS (assessed with: GQS)** | | | | | | |
| 0 (26 non-randomised studies) | not serious | very serious[c] | not serious | not serious | none | ⊕◯◯◯ Very low[c] |
| **Technical quality via JAMA-BC (assessed with: JAMA-BC)** | | | | | | |
| 746 (12 non-randomised studies) | not serious | serious[d] | not serious | serious[e] | none | ⊕◯◯◯ Very low[d,e] |
| **Technical quality via HONcode (assessed with: HONcode)** | | | | | | |
| 155 (3 non-randomised studies) | not serious | very serious[f] | not serious | very serious[g] | none | ⊕◯◯◯ Very low[f,g] |
| **Understandability via PEMAT-U (assessed with: PEMAT-U)** | | | | | | |
| 1453 (16 non-randomised studies) | not serious | serious[h] | not serious | not serious | none | ⊕◯◯◯ Very low[h] |
| **Accuracy via Misinformation (assessed with: Misinformation)** | | | | | | |
| 345169 (44 non-randomised studies) | not serious | very serious[i] | serious[j] | not serious | publication bias strongly suspected[k] | ⊕◯◯◯ Very low[i,j,k] |
| **Completeness via Completeness (assessed with: Completeness)** | | | | | | |
| 1036 (8 non-randomised studies) | not serious | serious[l] | serious[m] | serious[n] | none | ⊕◯◯◯ Very low[l,m,n] |
| **Actionability via PEMAT-A (assessed with: PEMAT-A)** | | | | | | |

## Dissemination via social media / AI-chatbots compared to NA for Addressing Cancer-related information

| Certainty assessment | | | | | | |
|---|---|---|---|---|---|---|
| 1116 (16 non-randomised studies) | not serious | very serious[o] | not serious | very serious[p] | none | ⨁◯◯◯ Very low[o,p] |

### Actionability via Usefulness (assessed with: Usefulness)

| | | | | | | |
|---|---|---|---|---|---|---|
| 1427 (13 non-randomised studies) | not serious | very serious[q] | not serious | very serious[r] | none | ⨁◯◯◯ Very low[q,r] |

### Harmfulness via Harmfulness (assessed with: Harmfulness)

| | | | | | | |
|---|---|---|---|---|---|---|
| 395 (5 non-randomised studies) | not serious | very serious[s] | not serious | very serious[t] | none | ⨁◯◯◯ Very low[s,t] |

### Commercial bias via Commercial bias (assessed with: Commercial bias)

| | | | | | | |
|---|---|---|---|---|---|---|
| 4432 (11 non-randomised studies) | not serious | very serious[u] | not serious | not serious | none | ⨁◯◯◯ Very low[u] |

**CI:** confidence interval

## Explanations

a. Very serious inconsistency ($I^2 = 93.22\%$) with wide variability across studies. The pooled estimate ranged from ~20% to ~70% across included studies. No clear explanation for the heterogeneity was identified.

b. Serious imprecision due to a moderately wide confidence interval (37.8%–49.4%). Although the sample size is relatively large (n = 3,701), the uncertainty may affect the interpretation of the quality rating.

c. Very serious inconsistency ($I^2 = 90.1\%$) across 26 studies. The pooled quality estimates ranged widely and no clear source of heterogeneity was identified.

d. Serious inconsistency ($I^2 = 75.3\%$) was observed. Although results were generally in the same direction, the magnitude varied and heterogeneity could not be fully explained.

e. Serious imprecision, the 95% CI (38.9%–53.4%) is moderately wide, and the total sample size (n = 746) is relatively small. Imprecision may affect confidence in the pooled estimate.

f. Very serious inconsistency ($I^2 = 94.4\%$) among only 3 studies. The effect sizes varied greatly, and no clear explanation for the heterogeneity was found.

g. Very serious imprecision due to extremely wide confidence interval (19.7%–79.7%) and small total sample size (n = 155), which significantly reduces confidence in the pooled estimate.

h. Very serious inconsistency ($I^2 = 88.7\%$) across the 16 included studies. Effect sizes varied widely and heterogeneity could not be explained through subgroup analysis.

i. Very serious inconsistency ($I^2 = 99.8\%$) among 44 studies. Effect estimates varied extremely across studies and no clear source of heterogeneity was identified.

j. Serious indirectness was noted due to the use of diverse, non-standardized tools and definitions of misinformation across studies. The inconsistency in measurement reduces the comparability and directness of the outcome.

k. Suspected publication bias. Egger's regression test indicated significant small-study effects and asymmetry (bias coefficient = –

5.67, 95% CI: −9.63 to −1.71, $p = 0.006$), suggesting potential publication bias in the body of evidence on misinformation.

l. Serious inconsistency, considerable heterogeneity ($I^2 = 77.1\%$) was observed among 8 studies evaluating completeness. Downgraded for serious inconsistency as variability in effect estimates could not be fully explained.

m. Serious indirectness due to the use of non-standardized, study-specific definitions and tools to assess completeness. The outcome may not be directly comparable across studies.

n. Serious imprecision due to moderate sample size ($n = 1,036$) and relatively wide confidence interval (28%–40%), which may limit certainty in the pooled estimate.

o. Very serious inconsistency ($I^2 = 98.5\%$) among 16 studies. Substantial variability in effect sizes was observed, with no clear explanation for heterogeneity.

p. Very serious imprecision due to an extremely wide confidence interval (18.1%–58.7%) and limited total sample size ($n = 1,116$), which significantly reduces confidence in the pooled estimate.

q. Very serious inconsistency ($I^2 = 99.4\%$). Substantial variability in estimates was observed across 13 studies, and the source of heterogeneity could not be clearly identified.

r. Very serious imprecision due to a wide confidence interval (26.2%–71.5%) and moderate sample size ($n = 1,427$), resulting in substantial uncertainty around the pooled estimate.

s. Very serious inconsistency ($I^2 = 88.7\%$) across 5 studies. Considerable heterogeneity in effect estimates was observed, and sources of variability could not be adequately explained.

t. Very serious imprecision due to wide confidence interval (9%–36.5%) and small sample size ($n = 395$), which results in substantial uncertainty about the pooled estimate.

u. Very serious inconsistency ($I^2 = 94.8\%$). The effect sizes varied greatly across studies, and no clear source of heterogeneity was identified.

Table S154. Quality assessment of information using different tools across studies in a proportional rating system

| No. | Paper | DISCERN | GQS | JAMA-BC | HONcode | PEMAT-U | Completeness | PEMAT-A | Usefulness | Misinformation | Harmful | Commercial bias |
|---|---|---|---|---|---|---|---|---|---|---|---|---|
| 1 | Szczesniewski et al., 2023 [114] | 50 | | | | | 50 | | | | | |
| 2 | Chen et al., 2023 [115] | | | | | | | | | 34.3 | | |
| 3 | Pan et al., 2023 [116] | 81.25 | | | | 64.2 | | 20 | | 0 | | |
| 4 | Musheyev et al., 2023 [117] | 68.75 | | | | 67.18 | | 40 | | | | |
| 5 | Gage-Bouchard et al., 2018 [5] | | | | | | | | | 32.75 | | |
| 6 | Petukhova et al., 2020 [56] | | | | | | | | | 25.65 | | 0.8 |
| 7 | Johnson et al., 2021 [47] | | | | | | | | | 32.5 | 30.5 | |
| 8 | Iglesias-Puzas et al., 2021 [48] | | | | | | | | | 70 | | |
| 9 | Alsyouf et al., 2019 [49] | | | | | | | | | 30 | | |
| 10 | Massey et al., 2020 [60] | | | | | | | | | 44.14 | | |
| 11 | Wilner & Holton, 2020 [57] | | | | | | | | | 11.42 | | |
| 12 | Herbert et al., 2022 [58] | | | | | | | | | 12.26 | | 5.29 |
| 13 | Du et al., 2021 [59] | | | | | | | | | 25.63 | | |
| 14 | Wang et al., 2023 [107] | 61 | | | | | 21.625 | | | | | |
| 15 | Yang et al., 2023 [108] | 56 | 68 | | | 82.23 | | 44 | | | | |
| 16 | Zheng et al., 2023 [109] | 50.38 | 49.5 | | | | | | | | | 6.5 |
| 17 | Xu et al., 2021 [110] | 25 | | | | 75 | | 0 | | 14.55 | | 10.91 |
| 18 | Xue et al., 2022 [111] | 23.5 | | | 42.25 | | | | | | | |
| 19 | Hu et al., 2022 [112] | 30.45 | | | | | 25.5 | | 50 | | | |
| 20 | Nastasi et al., 2018 [50] | | | | | | | | | 14.69 | | |
| 21 | Kornides et al., 2023 [51] | | | | | | | | | 24.02 | | |
| 22 | Kureyama et al., 2023 [44] | | | | | | | | | 44 | 31 | |
| 23 | Tomaszewski et al., 2021 [52] | | | | | | | | | 42.62 | | |
| 24 | Park et al., 2016 [53] | | | | | | | | | 1.58 | | |
| 25 | Lee et al., 2016 [54] | | | | | | | | | 1.15 | | |
| 26 | Lavorgna & Carr, 2021 [45] | | | | | | | | | 2.77 | | |
| 27 | Shi et al., 2019 [55] | | | | | | | | | 25.29 | | |
| 28 | Yang et al., 2023 [62] | 43.75 | | | | | | | | | | |
| 29 | Chen et al., 2018 [63] | | | | | | | | | 33.87 | | |

| | | | | | | | | | | | | |
|---|---|---|---|---|---|---|---|---|---|---|---|---|
| 30 | Pan et al., 2020 [113] | | 40 | | | | | | 37.65 | 62.35 | | 25.88 |
| 31 | Yurdaisik, 2020 [64] | 19.5 | | 55.75 | | | | | | | | |
| 32 | Richardson et al., 2022 [65] | | 40 | 77.5 | | | | | | | | |
| 33 | Çapkınoğlu & İflazoğlu, 2023 [66] | 49 | 50.75 | | | | 33.33 | | 52.1 | 47.9 | | |
| 34 | Yalkin et al., 2022 [67] | 57.32 | 48.75 | | | | 40.21 | | 54.01 | 45.99 | | |
| 35 | Reinhardt et al., 2022 [68] | 63.33 | 60 | 41 | | | | | 50 | | | |
| 36 | Altan Şallı & Egil, 2020 [69] | | 40.25 | | | | | | 78.75 | 7.5 | | |
| 37 | Kharbat & Abu Daabes, 2023 [70] | 17.17 | | | | 34.5 | | 41.4 | | | | |
| 38 | Chai & Ingledew, 2023 [71] | 71.6 | | | | | | | | | | 6 |
| 39 | Steeb et al., 2022 [72] | 57.5 | 69 | 27.75 | | 70.84 | | 45.94 | 7 | | 49 | |
| 40 | Radadiya et al., 2020 [73] | | 26.75 | | | | 30.76 | | | | | |
| 41 | Morena et al., 2023 [74] | 36 | | | | 78.8 | | 43.1 | | | | |
| 42 | Loeb et al., 2019 [75] | | | | | 67 | | 75 | | 40.67 | | 27.33 |
| 43 | Sütcüoğlu et al., 2023 [76] | 25.08 | 39.5 | 37.5 | | | | | | | | |
| 44 | Didier et al., 2023 [77] | 28.59 | | 45 | | | 35 | | | | | |
| 45 | Kaplan & Solak, 2023 [78] | 75 | 75 | | | | | | 89.58 | 10.42 | | |
| 46 | Di Bello, Di Mauro, et al., 2022 [79] | 43.75 | | | | 40 | | 0 | | 56 | | |
| 47 | Bahar-Ozdemir et al., 2022 [80] | 42.8 | 44.5 | 50.75 | | | | | | | | |
| 48 | Muzii et al., 2023 [81] | 50.16 | 41 | | | 72.3 | | 58.6 | | | | |
| 49 | Pratsinis et al., 2021 [82] | 34.38 | | | | | | | | 10.8 | | |
| 50 | Duran & Kizilkan, 2021 [46] | 42.6 | 40.25 | 39.75 | | | | | | | | |
| 51 | Baqain et al., 2023 [83] | 59.25 | 68.5 | | | | | | 84 | 6 | | |
| 52 | Loeb et al., 2021 [84] | 46.25 | | | | 71 | | 33 | | 21 | | 17 |
| 53 | García-Cano-Fernández et al., 2022 [85] | 28.13 | | | | 71.67 | | 35.5 | | | | 13.2 |
| 54 | Aydin & Akyol, 2020 [86] | 14.3 | | 50 | | | | | | | | |
| 55 | Śledzińska et al., 2021 [87] | 35.67 | | | | | | | | | | |
| 56 | Shungu et al., 2021 [88] | 37.88 | | | | | | | | | | 10 |
| 57 | Rodriguez Rodriguez et al., 2022 [89] | 54.64 | 62.25 | | 78.3 | | | | | | | |
| 58 | Reinhardt et al., 2023 [90] | 52.5 | 68 | 37.25 | | 64.27 | | 58.22 | 18.4 | | 5.3 | |
| 59 | Vu et al., 2021 [91] | 37.11 | | | | 67.5 | | 50 | | 76.25 | | 18.75 |
| 60 | Segado-Fernández et al., 2023 [92] | 31.25 | 30.25 | | | | | | | | | |
| 61 | Di Bello, Collà Ruvolo, et al., 2022 [93] | 46.1 | | | | 71.4 | | 100 | | 56 | | |

| | | | | | | | | | | |
|---|---|---|---|---|---|---|---|---|---|---|
| 62 | Mamo et al., 2021 [94] | 2 | 0 | | | | | | | 100 |
| 63 | Meteran et al., 2023 [95] | | | | | | | | | 5.59 |
| 64 | Şan, 2022 [96] | 22.91 | 35.75 | 43.75 | | | | | | |
| 65 | Ruppert et al., 2017 [97] | | | | | | | | | 13.82 |
| 66 | Ayoub et al., 2021 [98] | 40 | 50 | | | | 36.36 | | 63 | 41.47 |
| 67 | Godskesen et al., 2023 [99] | 42.75 | 83.25 | | | | | | | |
| 68 | Yetişir, 2023 [100] | 80 | 75 | | | | | | | 18 |
| 69 | Krakowiak et al., 2022 [101] | 34.33 | 35 | 45 | | | | | | 29.41 |
| 70 | Wittenberg-Lyles et al., 2014 [102] | | | | 28 | | | | | |
| 71 | Enver et al., 2020 [103] | | | | | | | | 20 | 30.33 |
| 72 | Güloğlu et al., 2022 [104] | 59.6 | 49.5 | | | | 54.6 | | 48.78 | 51.22 |
| 73 | Sahin et al., 2019 [105] | | | | | | | | | 15.22 |
| 74 | Brachtenbach et al., 2020 [106] | | | | | | | | | 9.02 |
| 75 | Xu et al., 2022 [61] | | | | | 74 | | 0 | | 11.9 |

Figure S1. Funnel Plot for Assessing Publication Bias in Studies Evaluating DISCERN

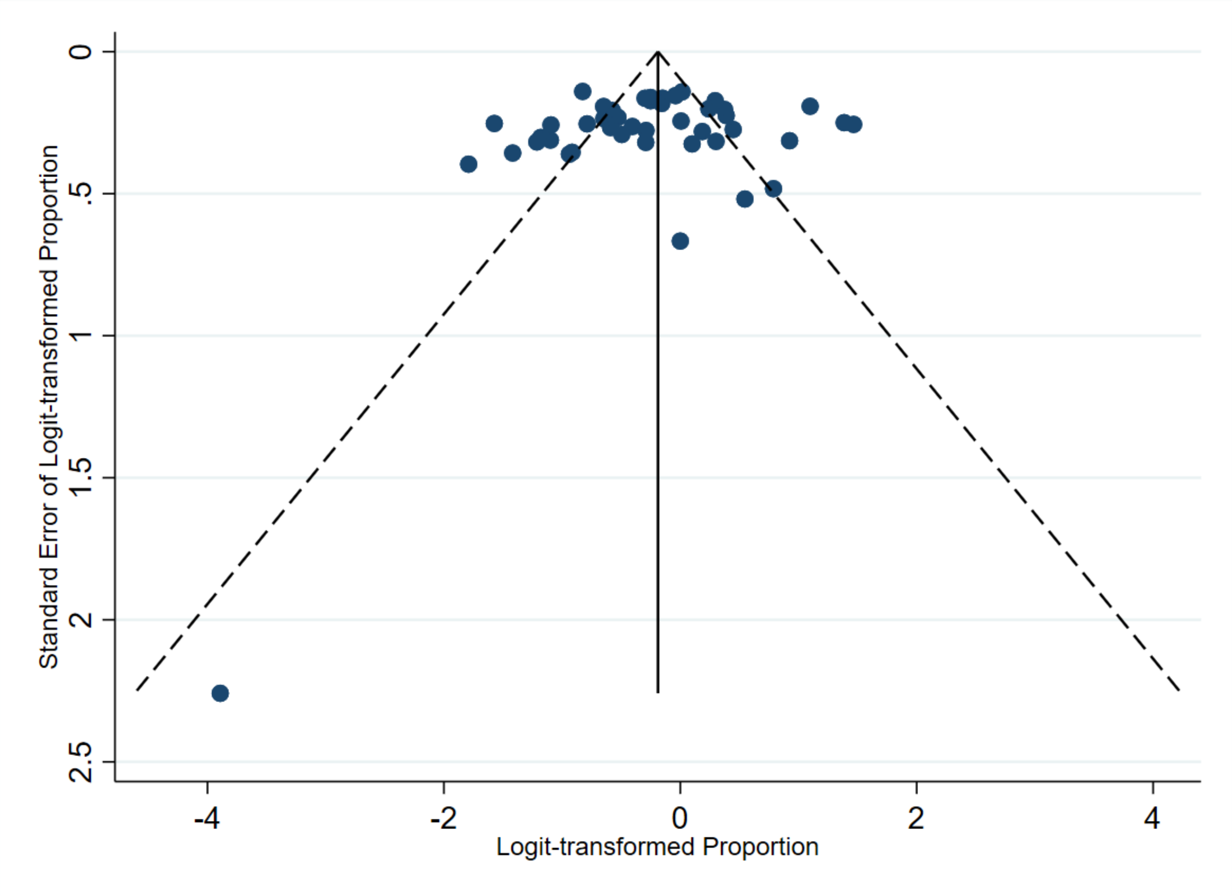


Assessment of publication bias

Egger’s regression test did not indicate significant asymmetry (bias coefficient = –1.01, 95% CI: -3.58 to 1.56, p = 0.433), suggesting no evidence of publication bias in studies evaluating DISCERN.

Figure S2. Funnel Plot for Assessing Publication Bias in Studies Evaluating GQS

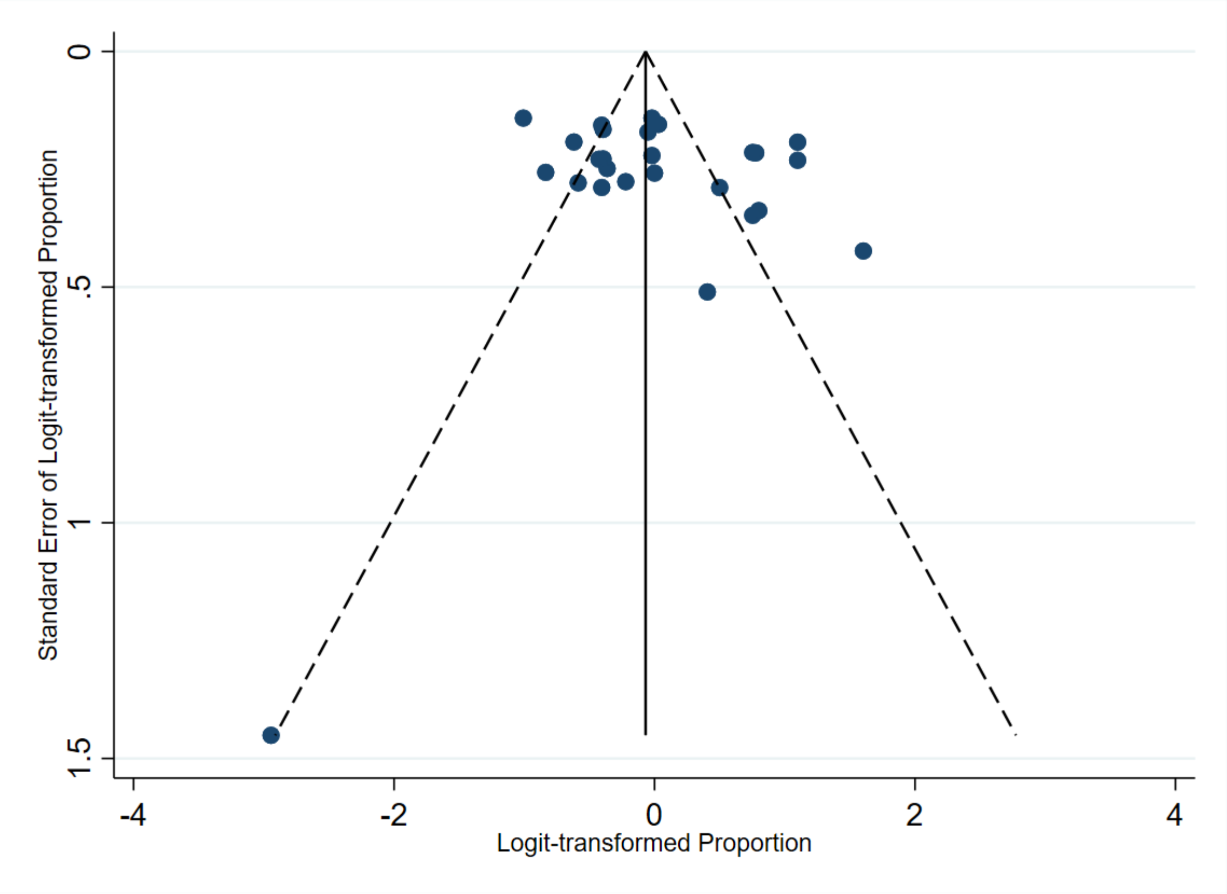


Assessment of Publication Bias

Egger's regression test did not indicate significant asymmetry (bias coefficient = 2.06, 95% CI: –1.45 to 5.57, p = 0.238), suggesting no evidence of publication bias in studies evaluating GQS.

Figure S3. Funnel Plot for Assessing Publication Bias in Studies Evaluating JAMA-BC

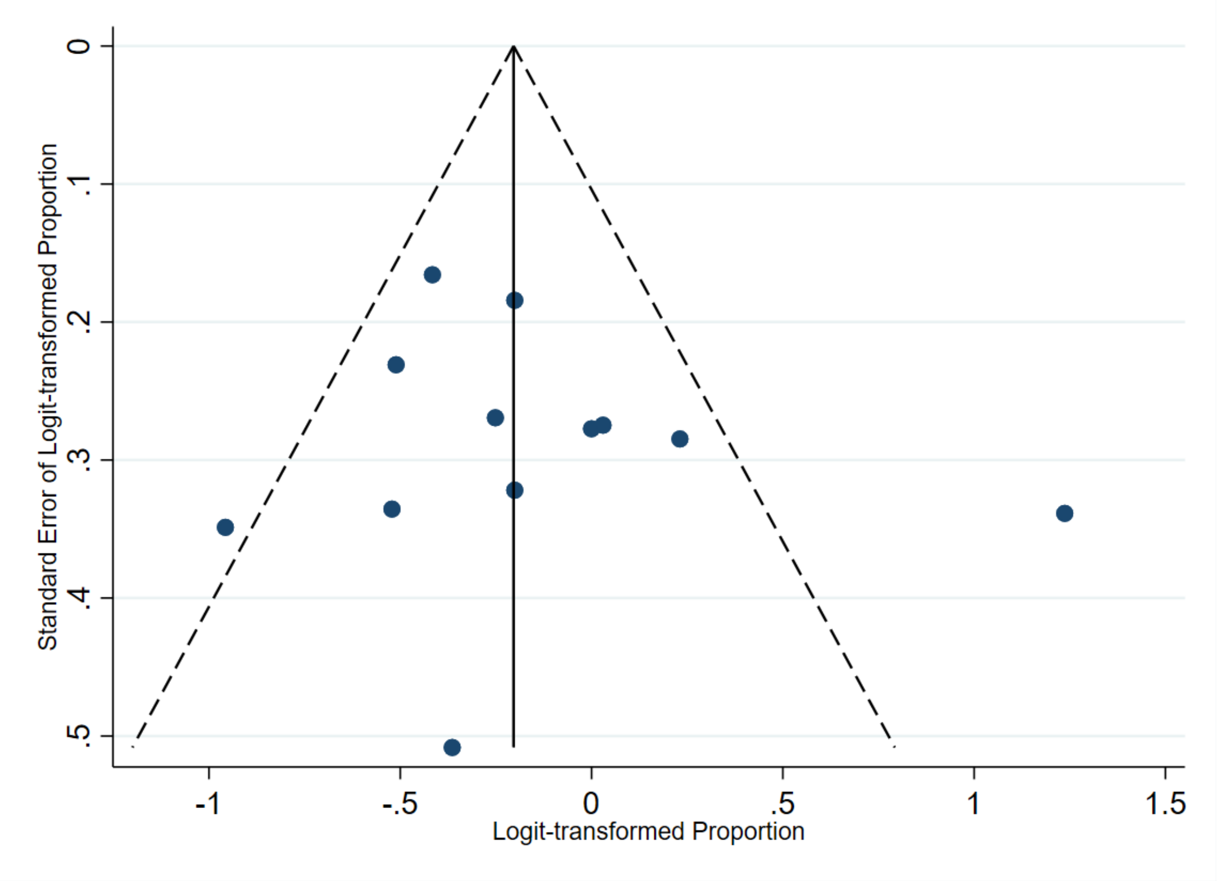


Assessment of Publication Bias

Egger's regression test did not indicate significant asymmetry (bias coefficient = 1.24, 95% CI: –2.67 to 5.15, p = 0.496), suggesting no evidence of publication bias in studies evaluating JAMA-BC.

Figure S4. Funnel Plot for Assessing Publication Bias in Studies Evaluating HONCode

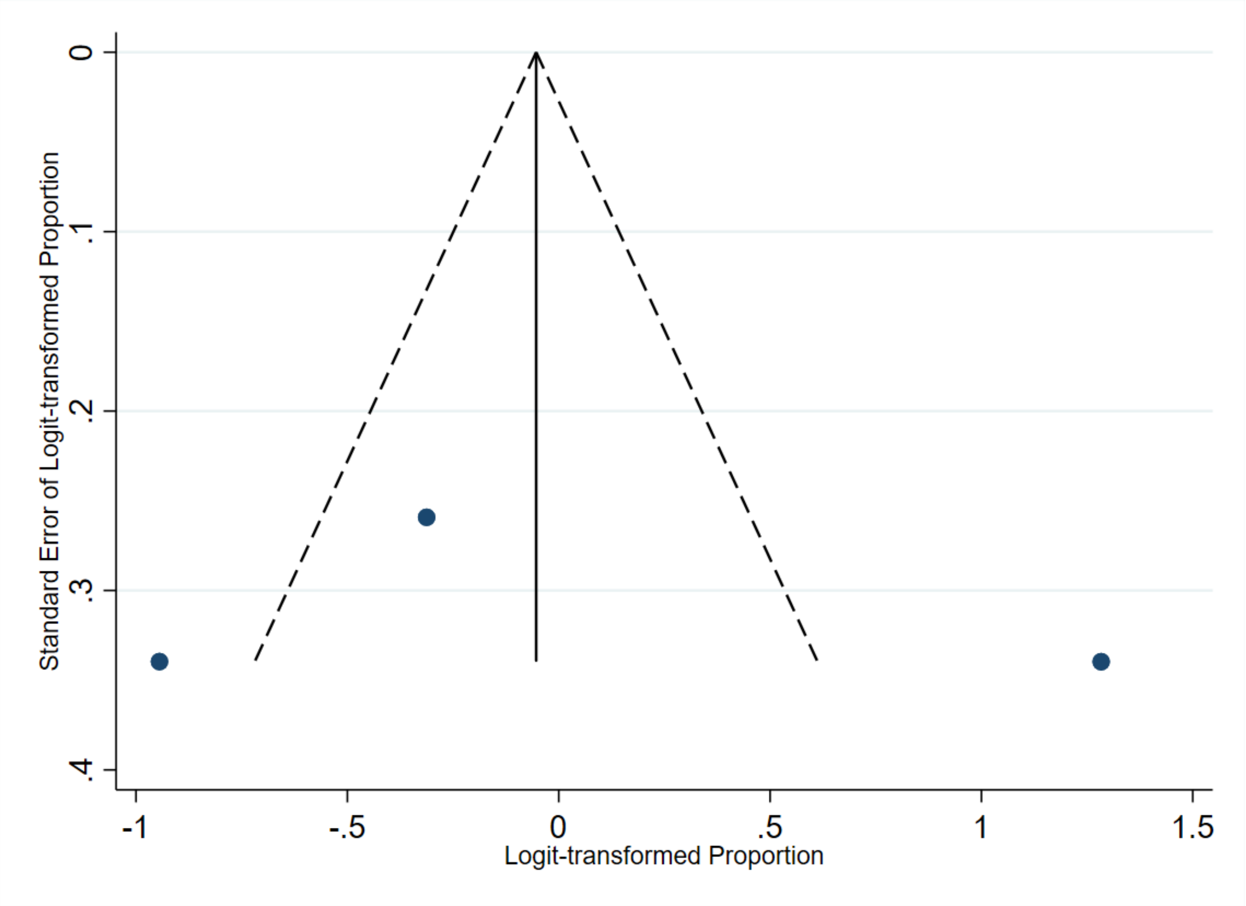


Assessment of Publication Bias

Egger’s regression test did not indicate significant asymmetry (bias coefficient = 6.00, 95% CI: –252.73 to 264.72, p = 0.818), suggesting no evidence of publication bias in studies evaluating HONCode.

Figure S5. Funnel Plot for Assessing Publication Bias in Studies Evaluating PEMAT-U

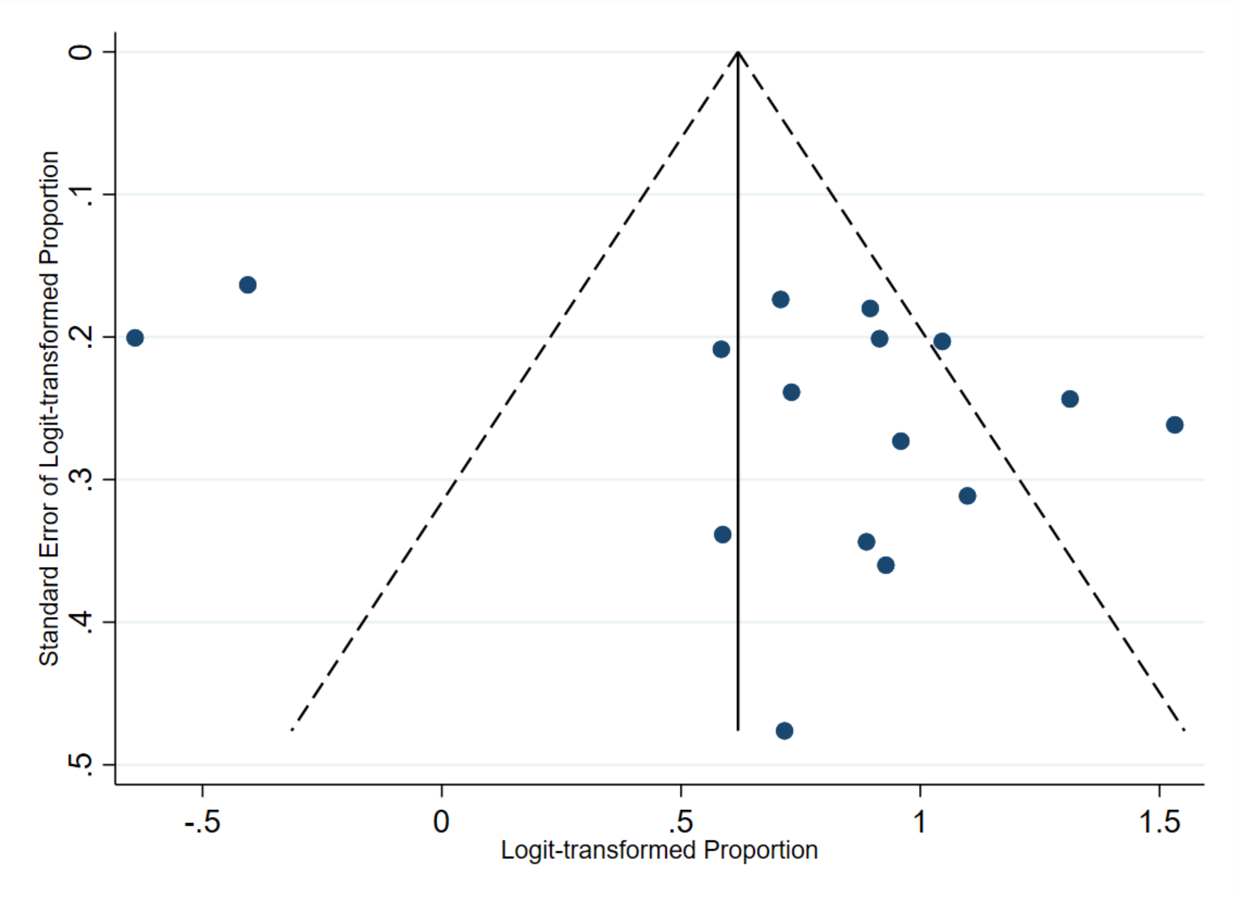


Assessment of Publication Bias

Egger's regression test did not indicate significant asymmetry (bias coefficient = 3.78, 95% CI: –1.57 to 9.13, $p = 0.152$), suggesting no evidence of publication bias in studies evaluating PEMAT-U

Figure S6. Funnel Plot for Assessing Publication Bias in Studies Evaluating Misinformation

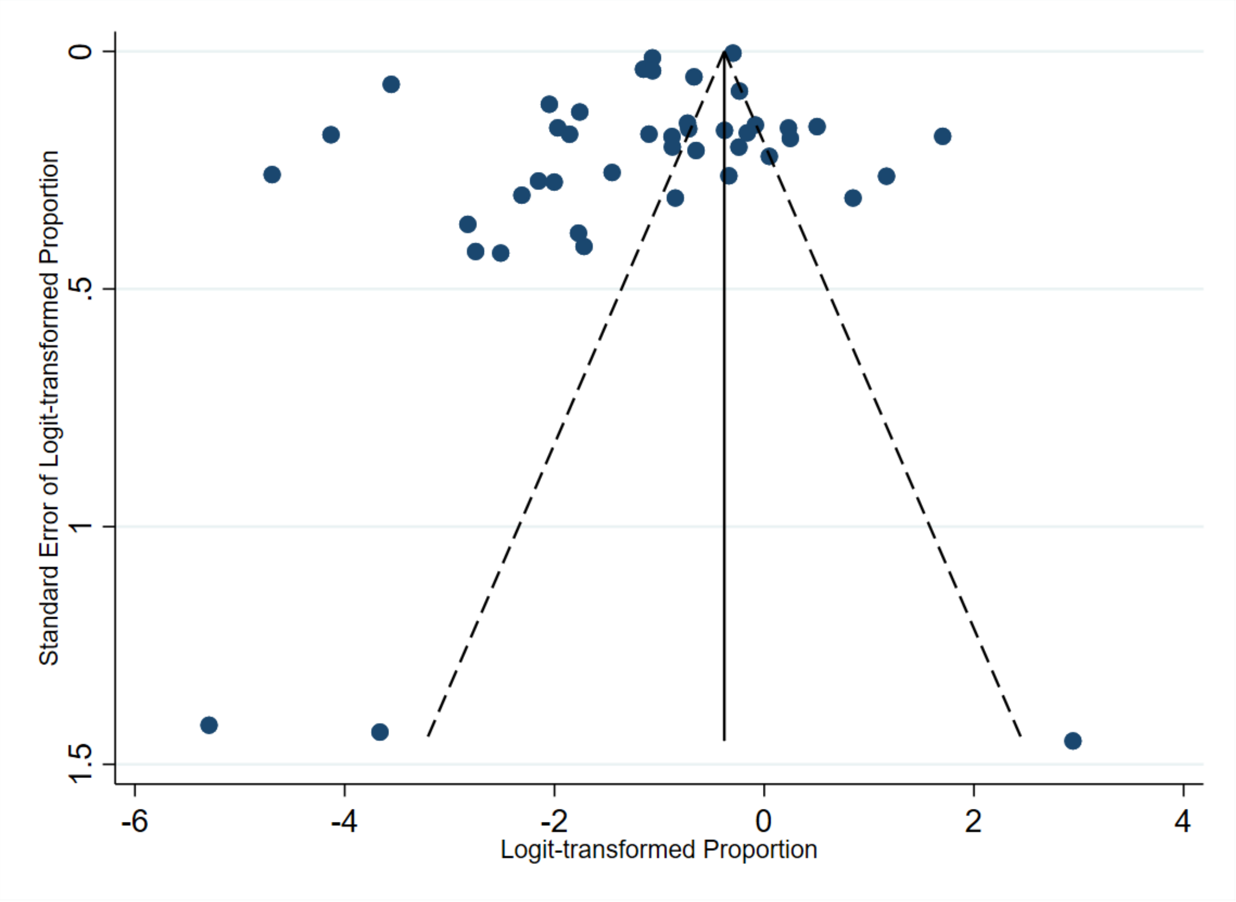


Assessment of Publication Bias

Egger’s regression test indicated significant asymmetry (bias coefficient = –5.67, 95% CI: –9.63 to –1.71, p = 0.006), suggesting potential publication bias in studies evaluating Misinformation.

Figure S7. Funnel Plot for Assessing Publication Bias in Studies Evaluating Completeness

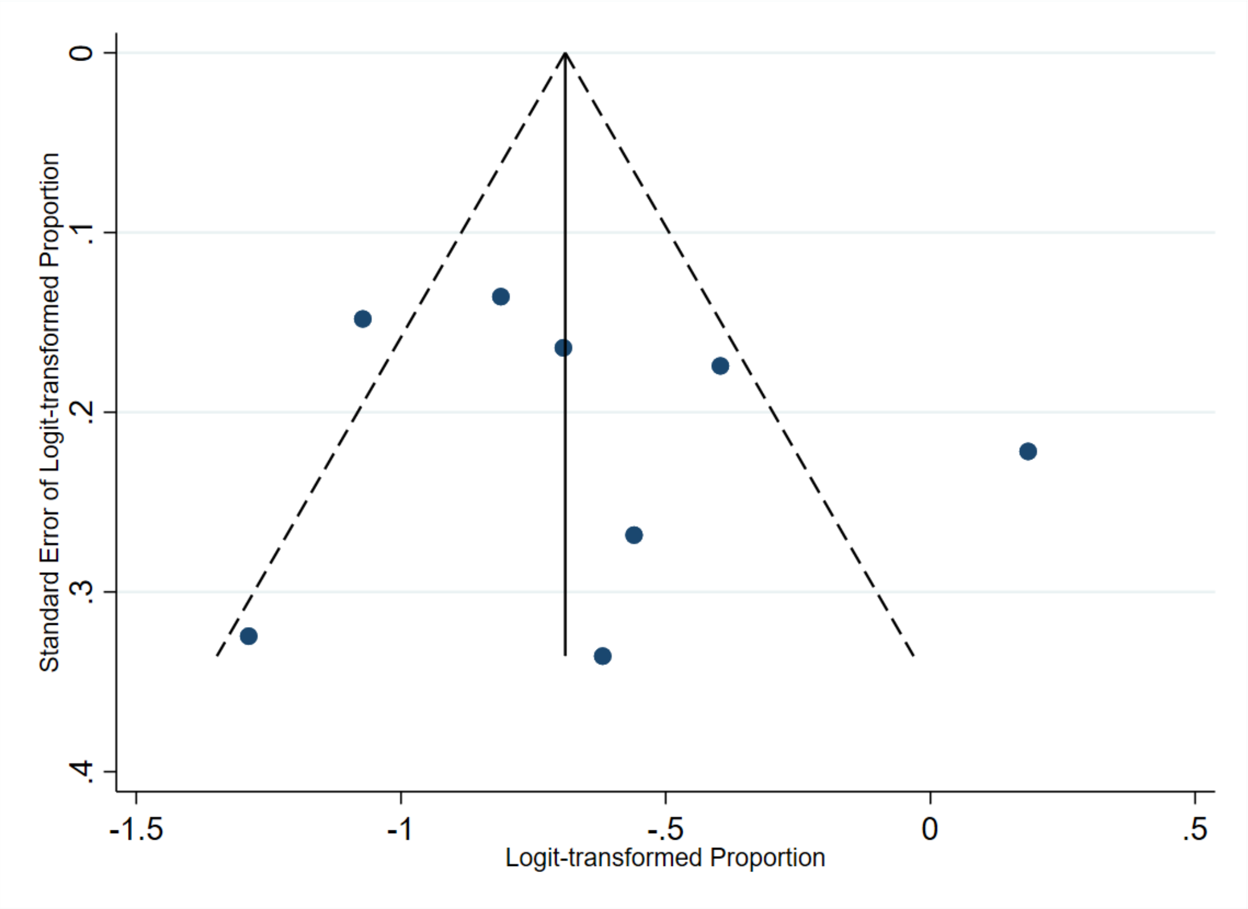


Assessment of Publication Bias

Egger's regression test did not indicate significant asymmetry (bias coefficient = 1.35, 95% CI: -4.89 to 7.60, p = 0.615), suggesting no evidence of publication bias in studies evaluating Completeness.

Figure S8. Funnel Plot for Assessing Publication Bias in Studies Evaluating PEMAT-A

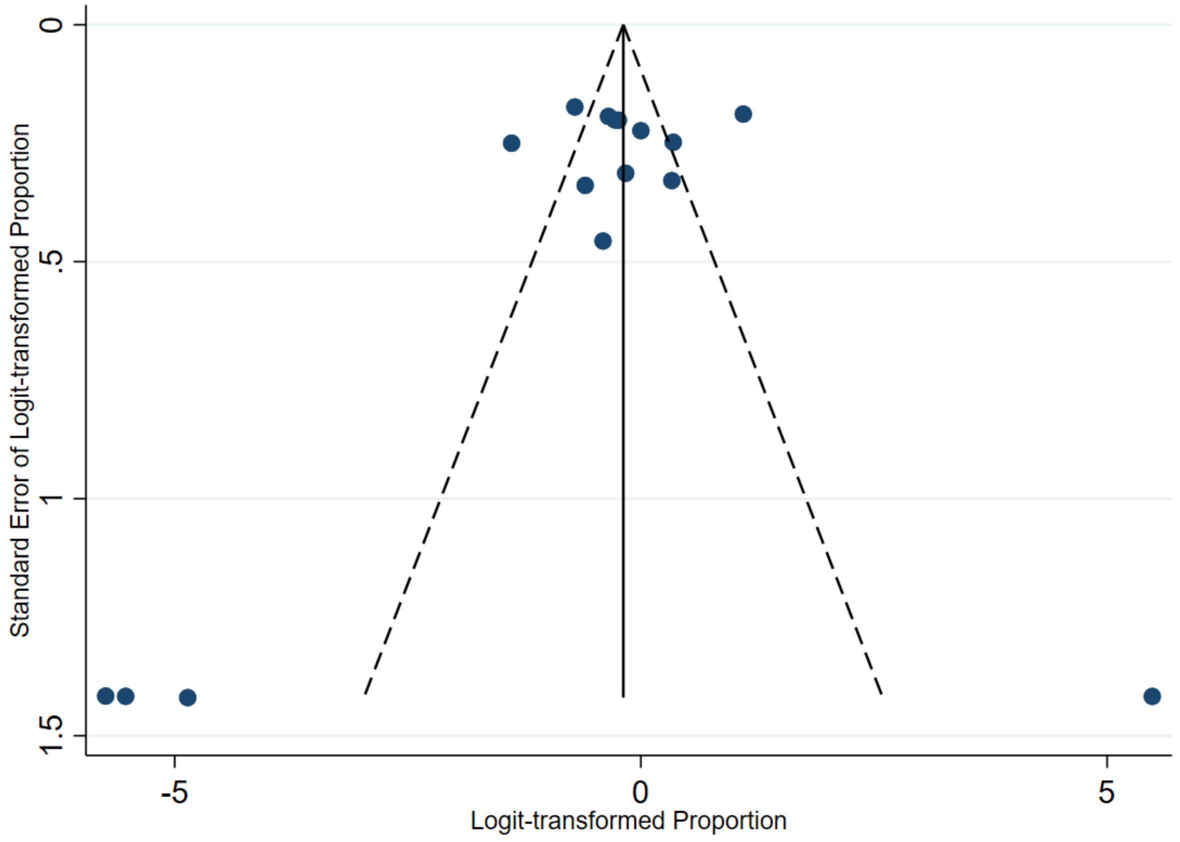


Assessment of Publication Bias

Egger's regression test did not indicate significant asymmetry (bias coefficient = –1.83, 95% CI: -5.34 to 1.69, p = 0.284), suggesting no evidence of publication bias in studies evaluating PEMAT-A.

Figure S9. Funnel Plot for Assessing Publication Bias in Studies Evaluating Usefulness

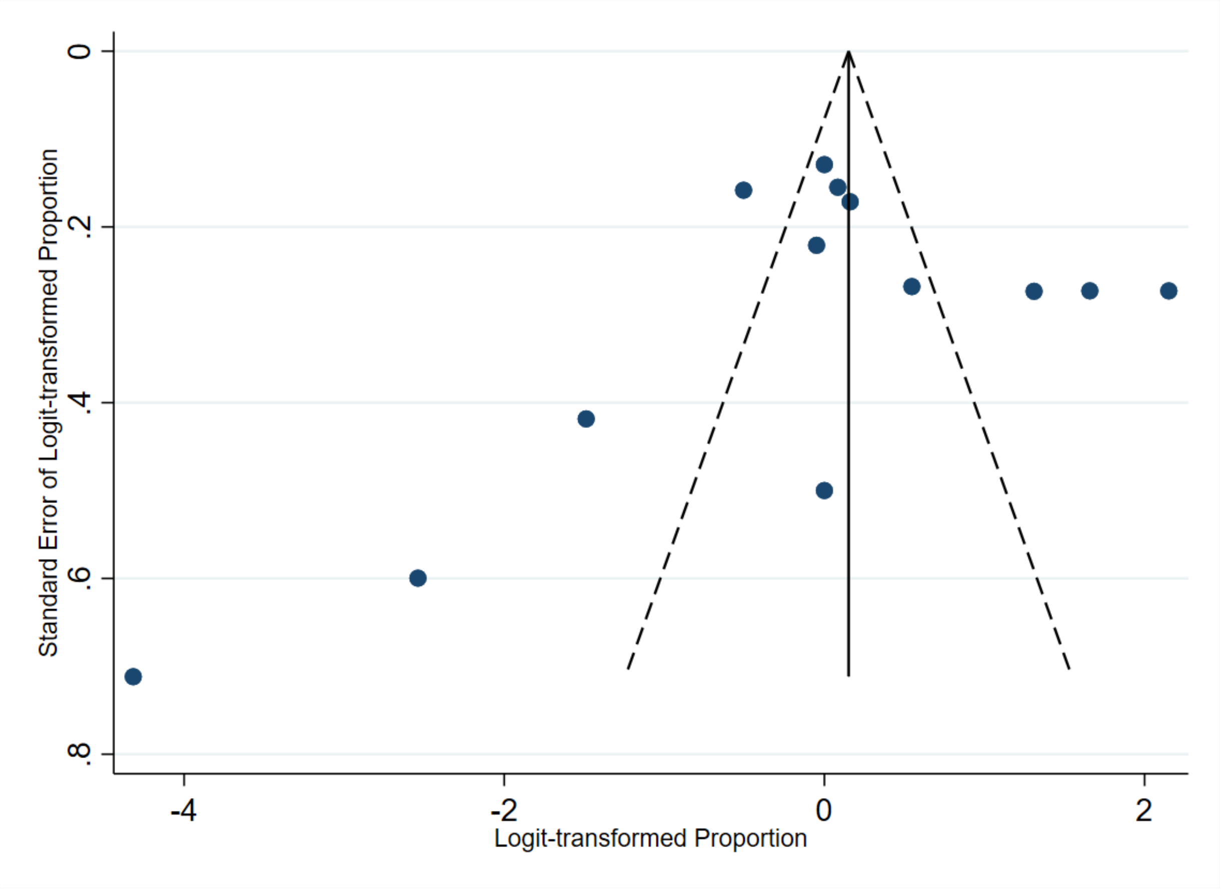


Assessment of Publication Bias

Egger’s regression test did not indicate significant asymmetry (bias coefficient = –1.28, 95% CI: –7.31 to 4.75, p = 0.650), suggesting no evidence of publication bias in studies evaluating Usefulness.

Figure S10. Funnel Plot for Assessing Publication Bias in Studies Evaluating Harmfulness

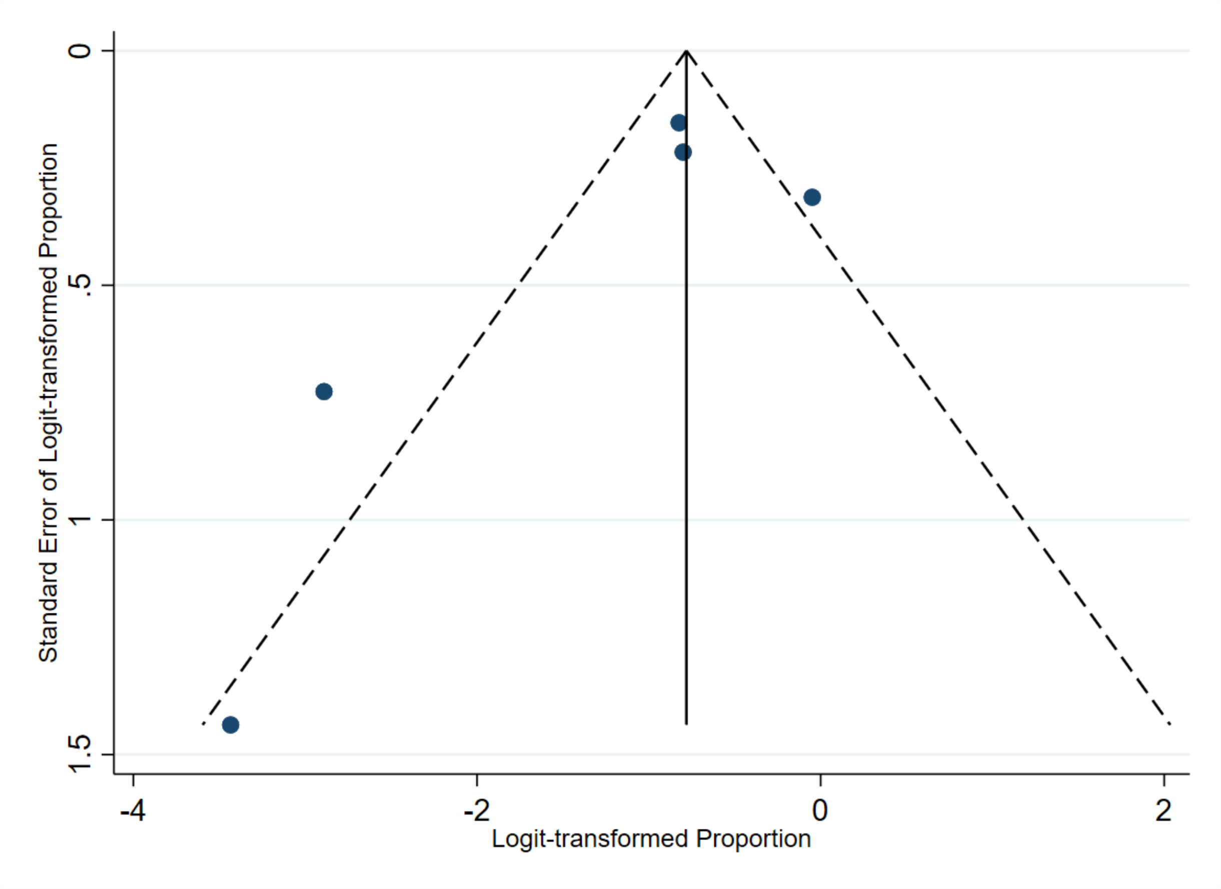


Assessment of Publication Bias

Egger's regression test did not indicate significant asymmetry (bias coefficient = –1.88, 95% CI: –7.16 to 3.41, p = 0.340), suggesting no evidence of publication bias in studies evaluating Harmfulness.

Figure S11. Funnel Plot for Assessing Publication Bias in Studies Evaluating Commercial Bias

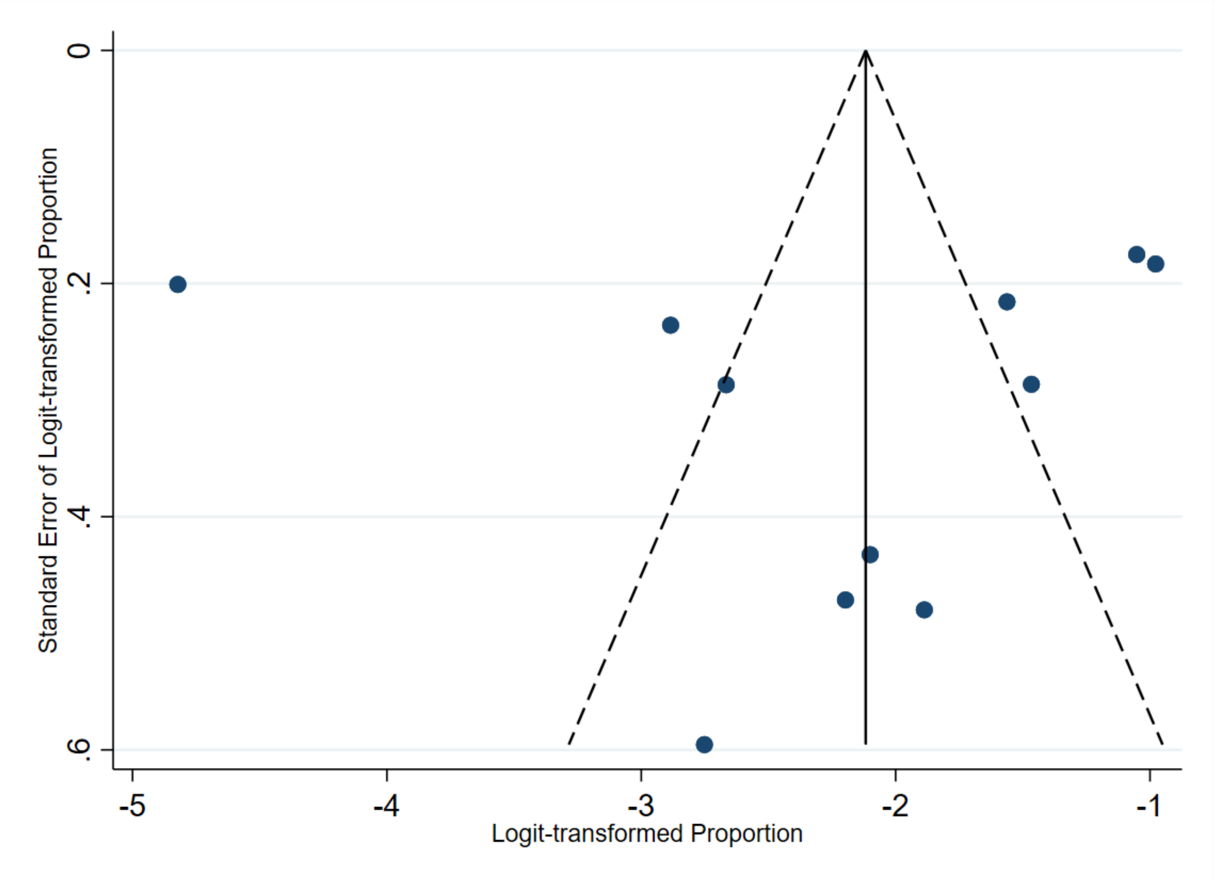


Assessment of Publication Bias

Egger's regression test did not indicate significant asymmetry (bias coefficient = –1.58, 95% CI: –12.33 to 9.17, p = 0.747), suggesting no evidence of publication bias in studies evaluating Commercial Bias.

Figure S12. Forest Plot of Proportion of DISCERN Assessment Tools in Pooled Analysis

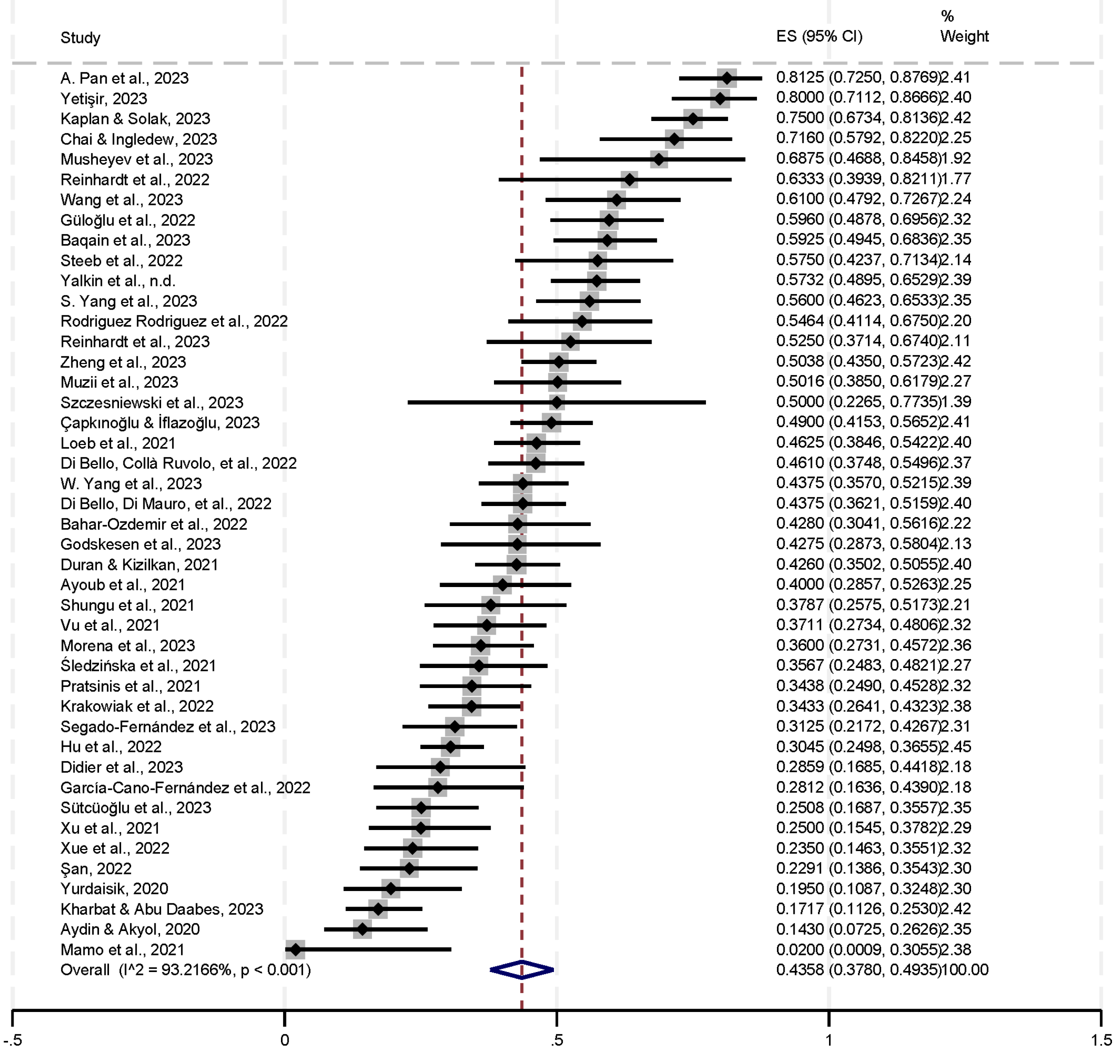

Figure S13. Forest Plot of Proportion of GQS Assessment Tools in Pooled Analysis

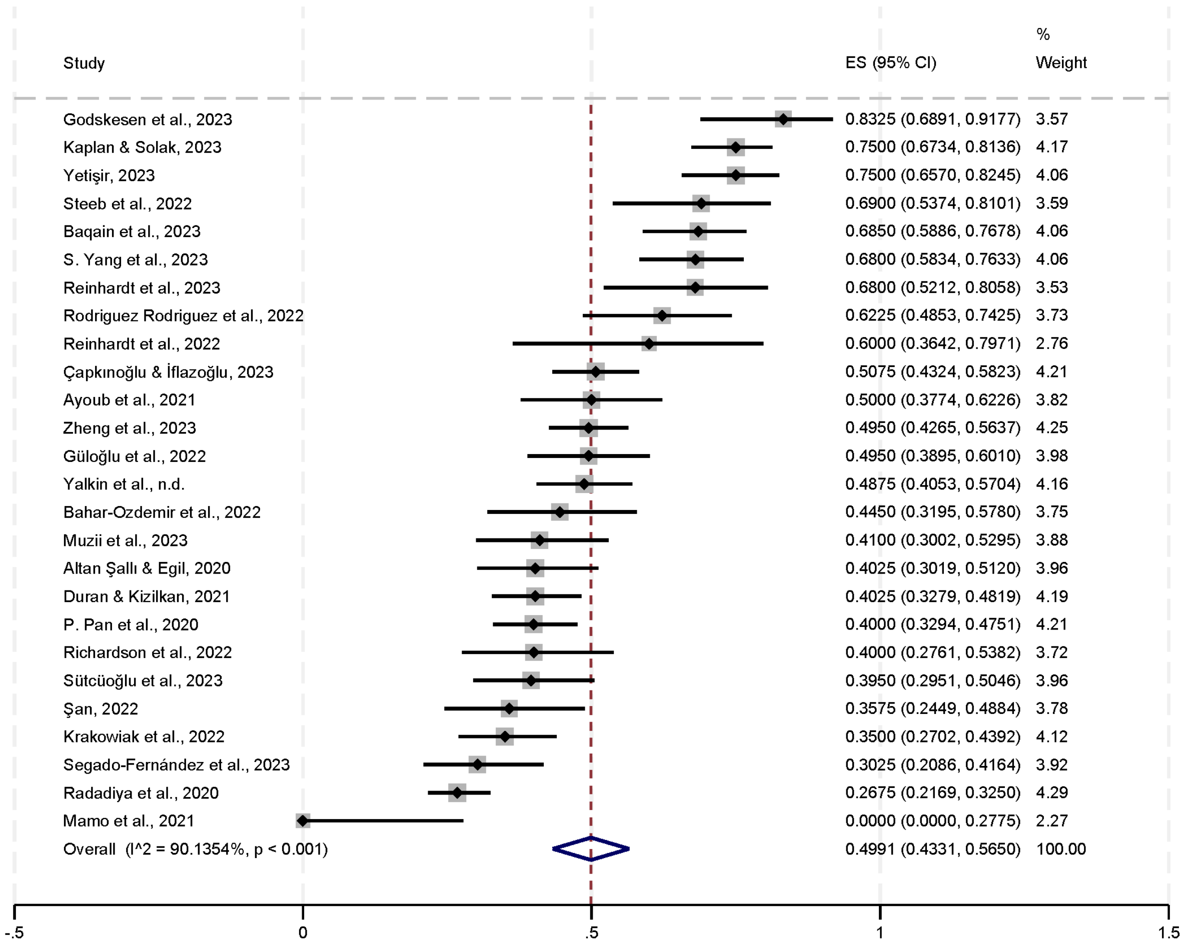

Figure S14. Forest Plot of Proportion of JAMA-BC Assessment Tools in Pooled Analysis

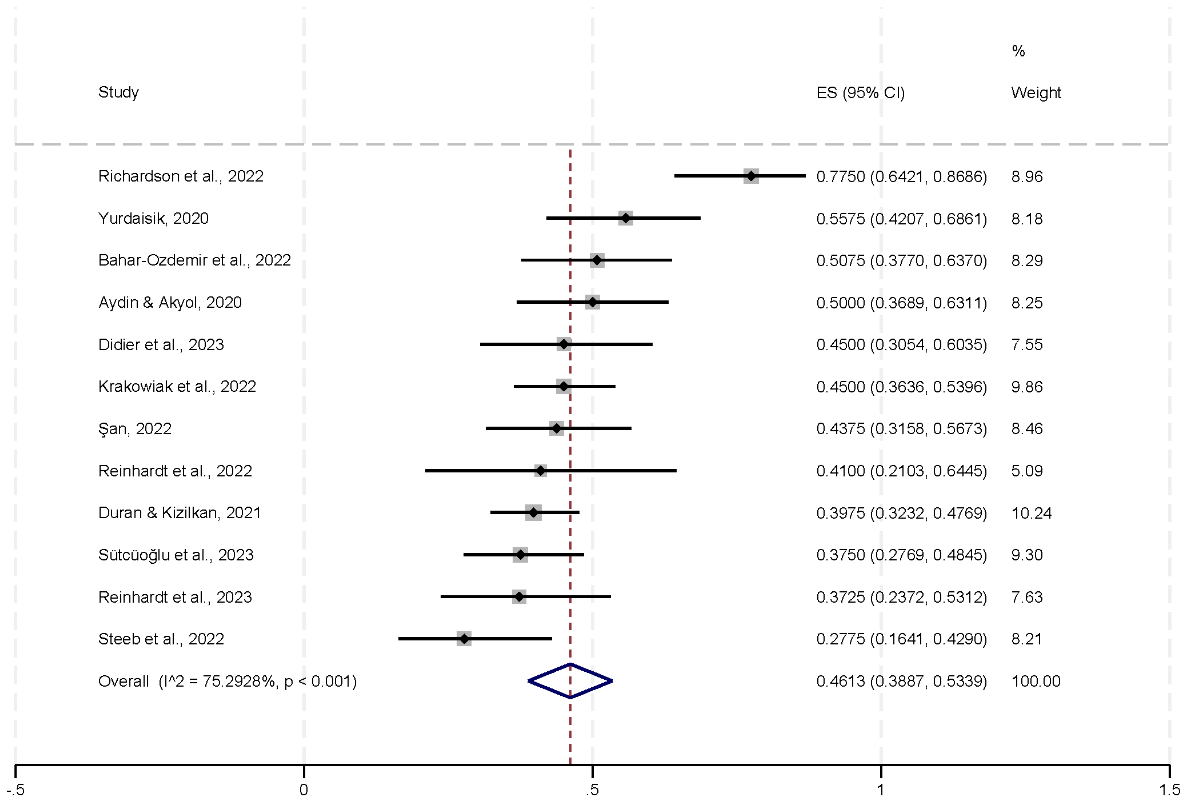

Figure S15. Forest Plot of Proportion of HONcode Assessment Tools in Pooled Analysis

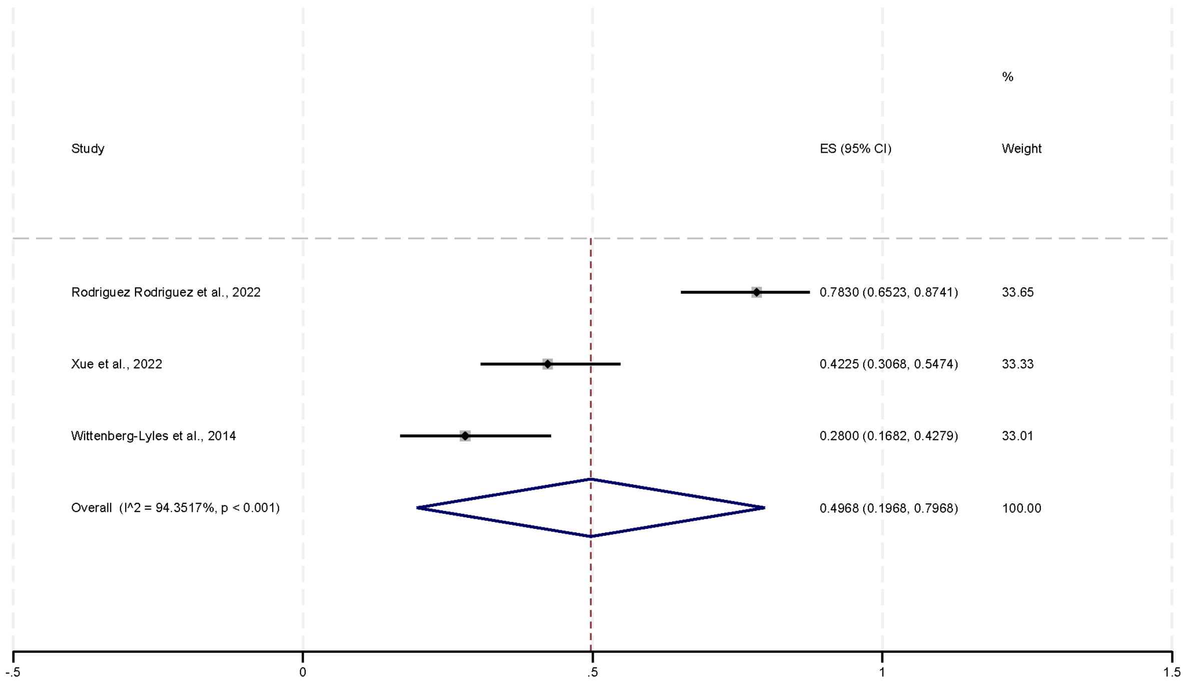

# Figure S16. Forest Plot of Proportion of PEMAT-U Assessment Tools in Pooled Analysis

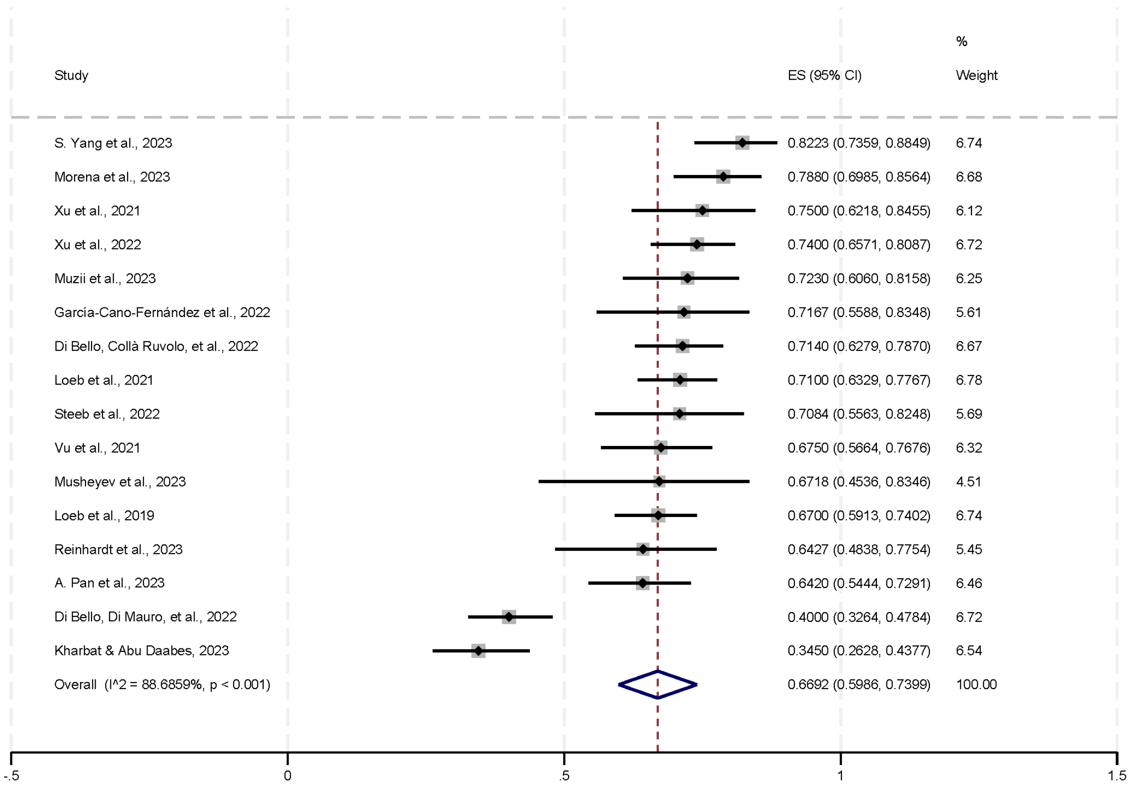

Figure S17. Forest Plot of Proportion of Misinformation Assessment Tools in Pooled Analysis

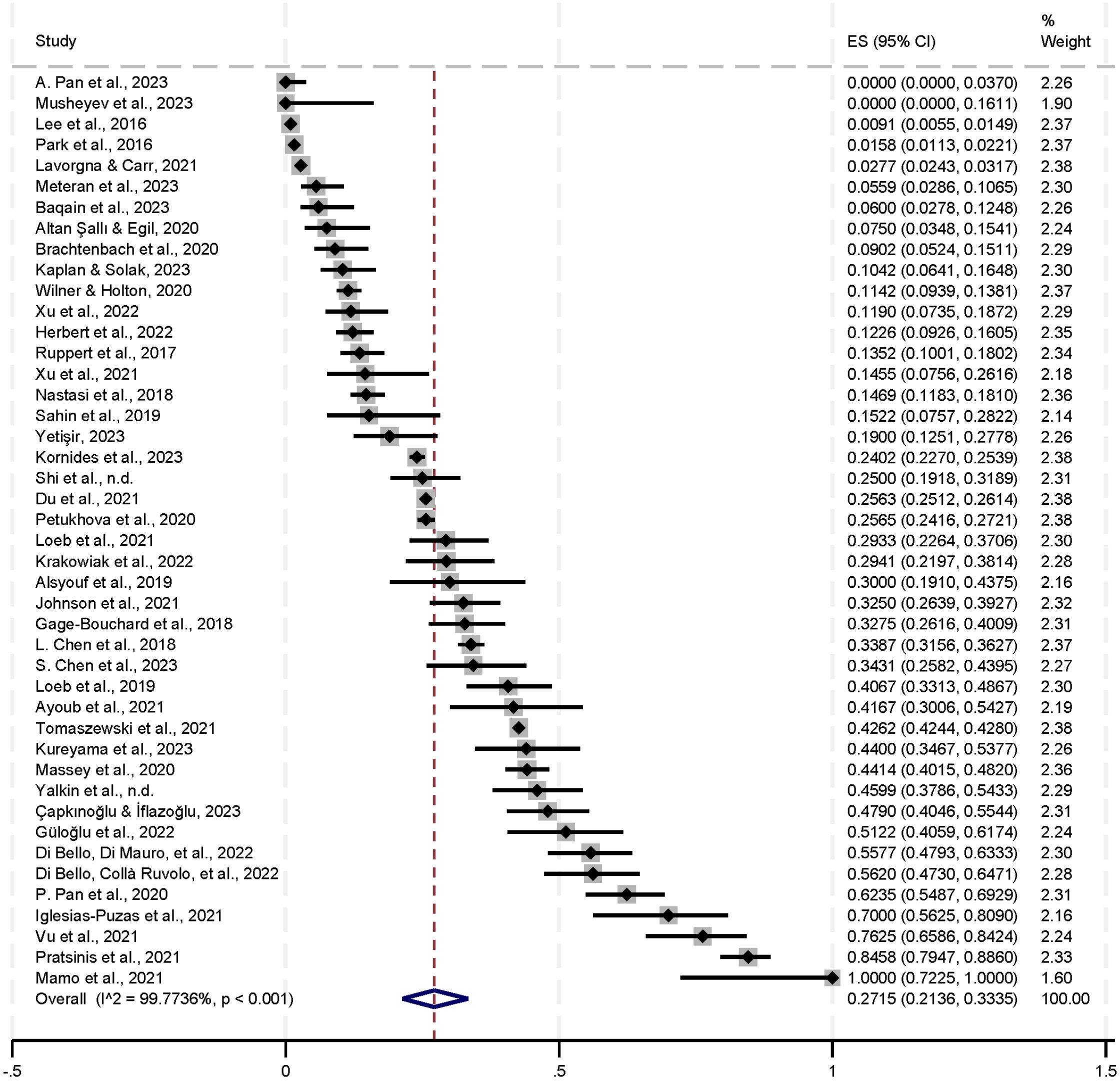

Figure S18. Forest Plot of Proportion of Completeness Assessment Tools in Pooled Analysis

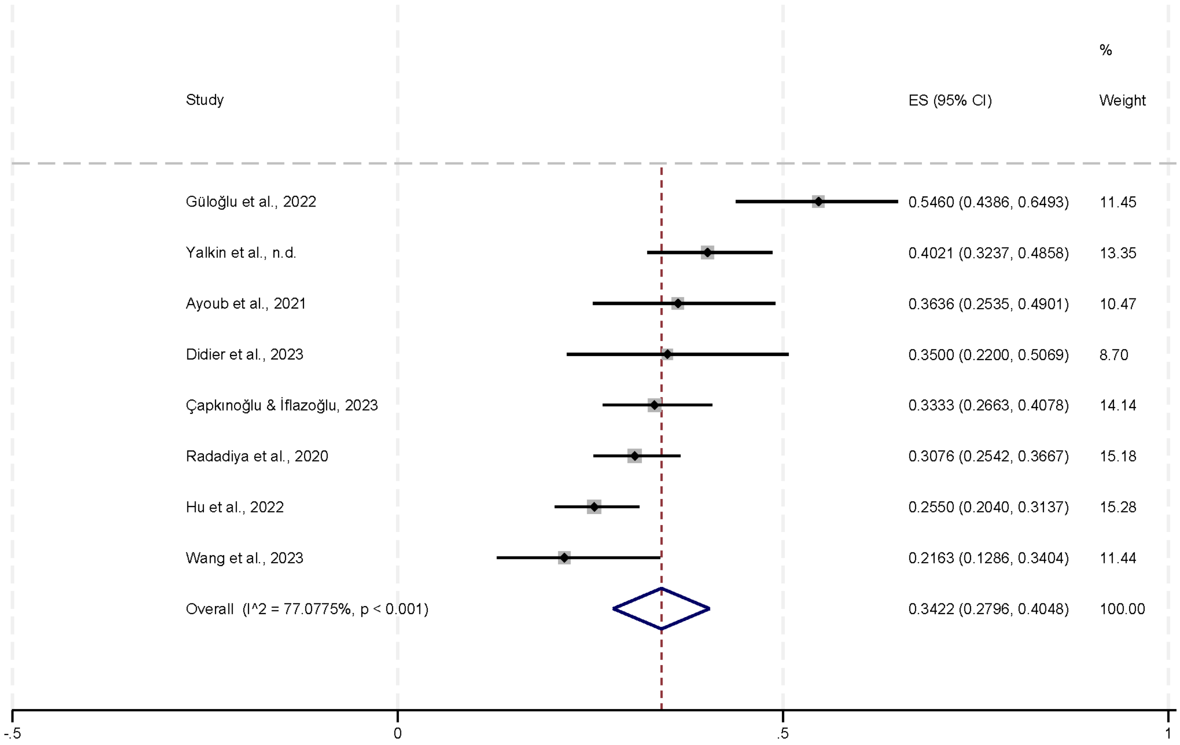

Figure S19. Forest Plot of Proportion of PEMAT-A Assessment Tools in Pooled Analysis

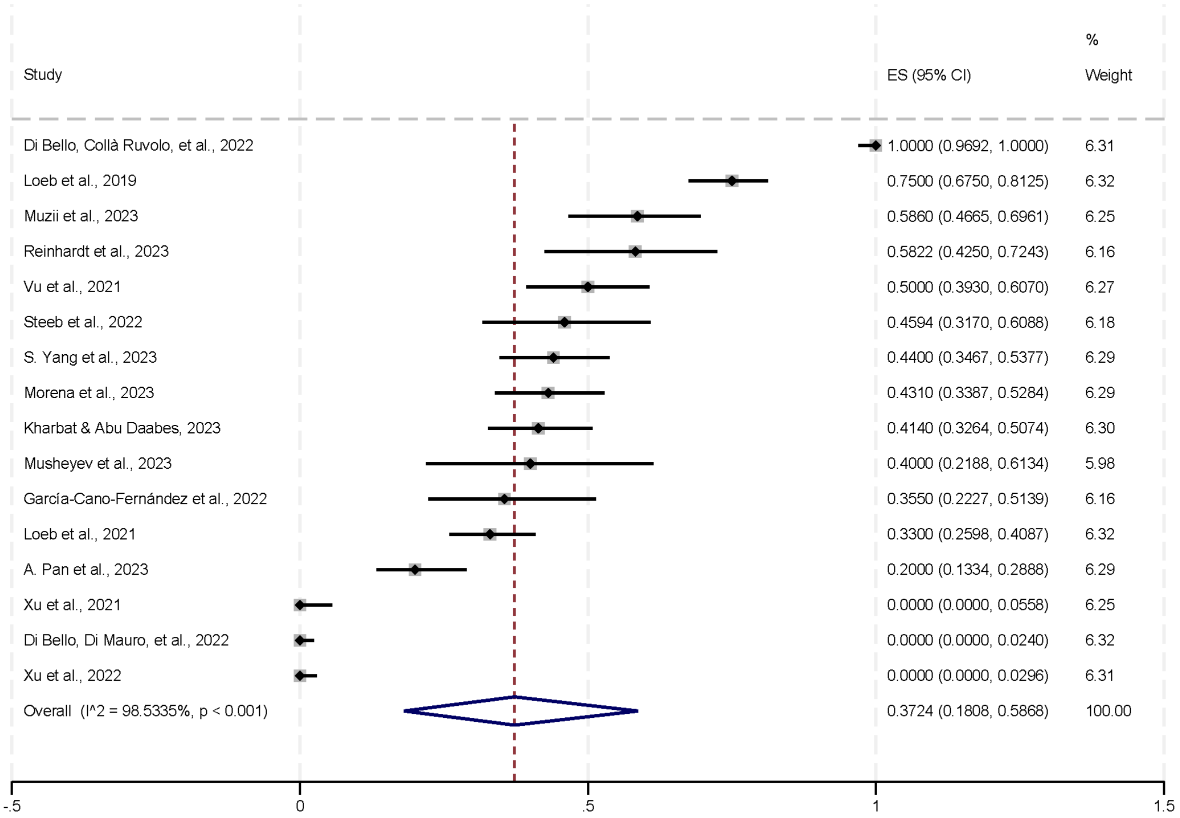

Figure S20. Forest Plot of Proportion of Usefulness Assessment Tools in Pooled Analysis

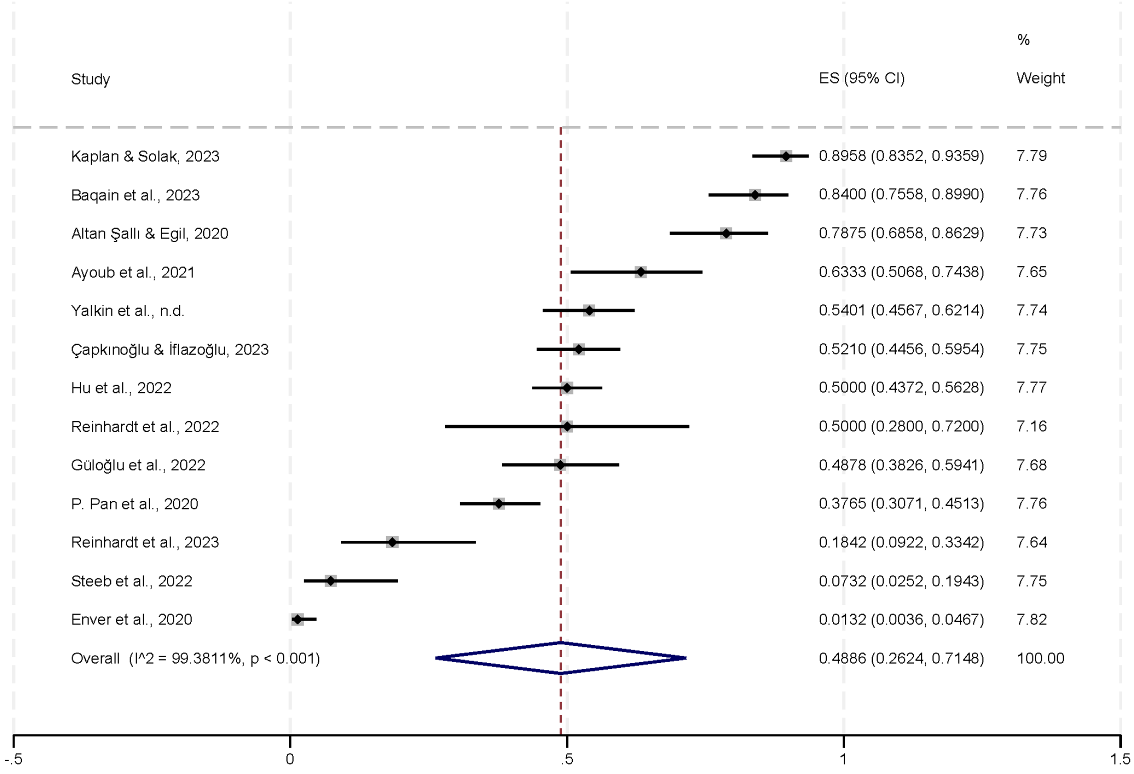

Figure S21. Forest Plot of Proportion of Harmfulness Assessment Tools in Pooled Analysis

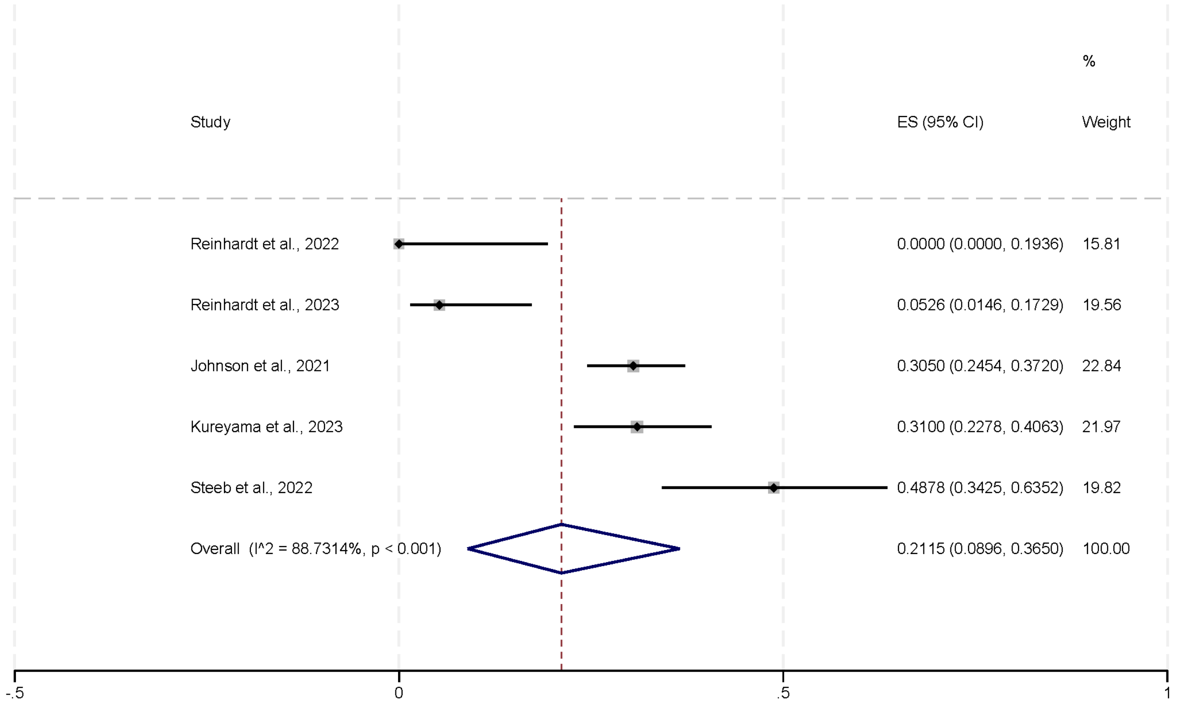

Figure S22. Forest Plot of Proportion of Commercial Bias Assessment Tools in Pooled Analysis

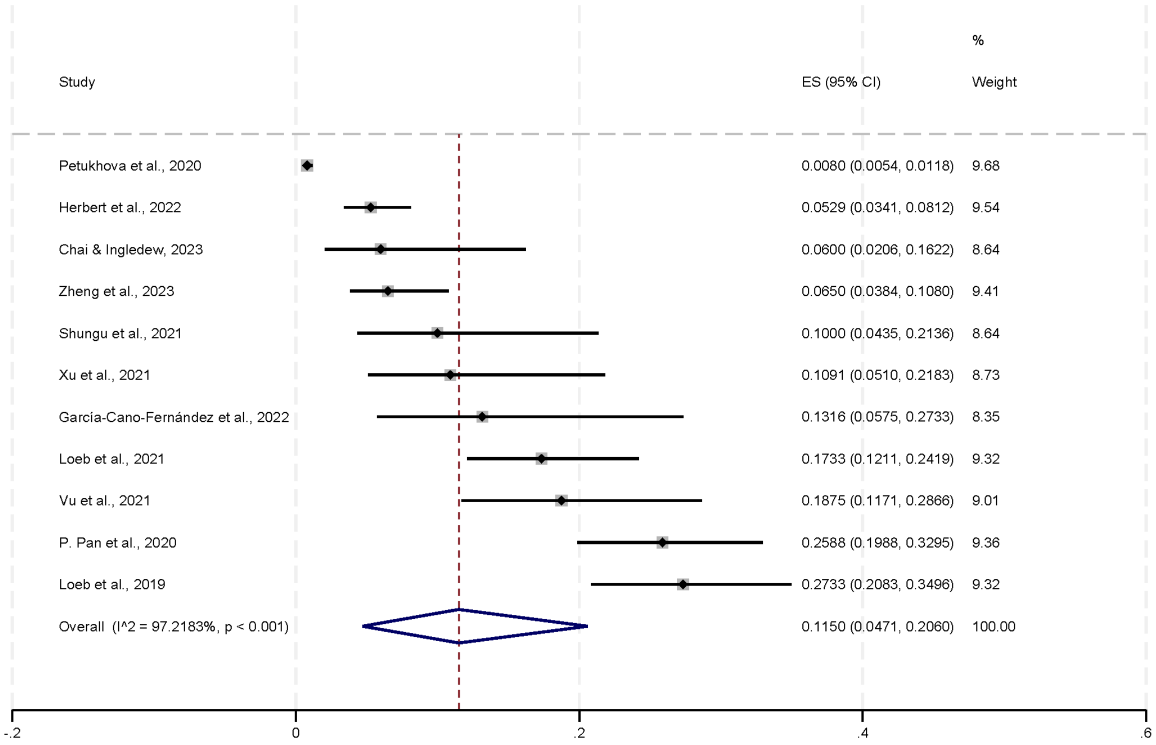

Figure S23. Leave-One-Out Sensitivity Analysis of Studies Assessing DISCERN Using a Random-Effects Model

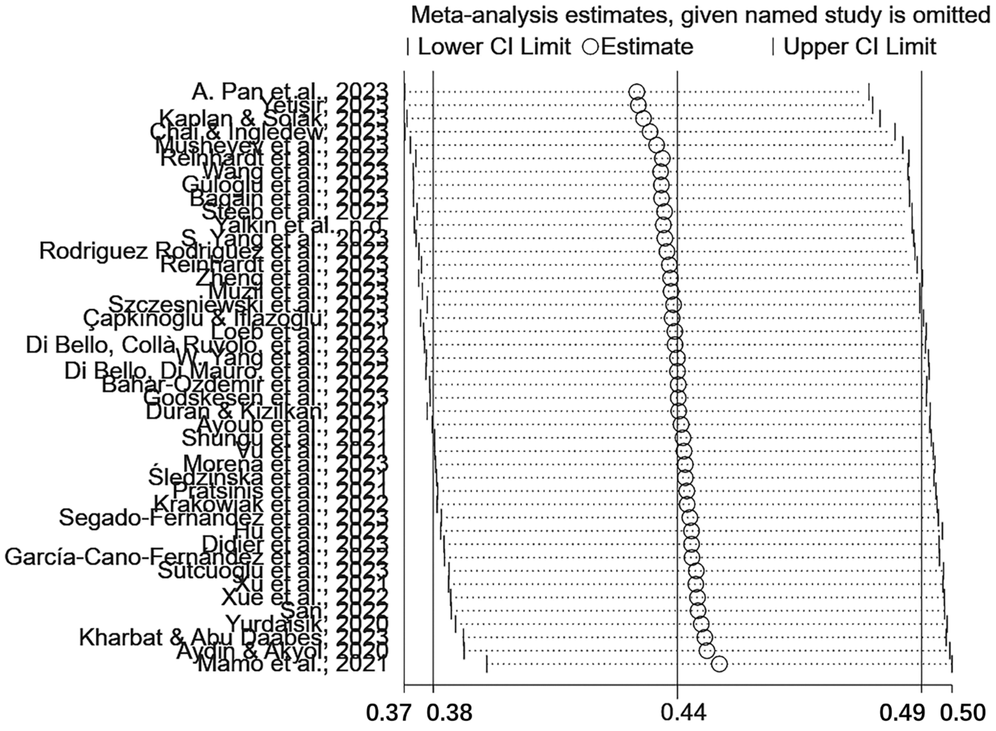

Figure S24. Leave-One-Out Sensitivity Analysis of Studies Assessing GQS Using a Random-Effects Model

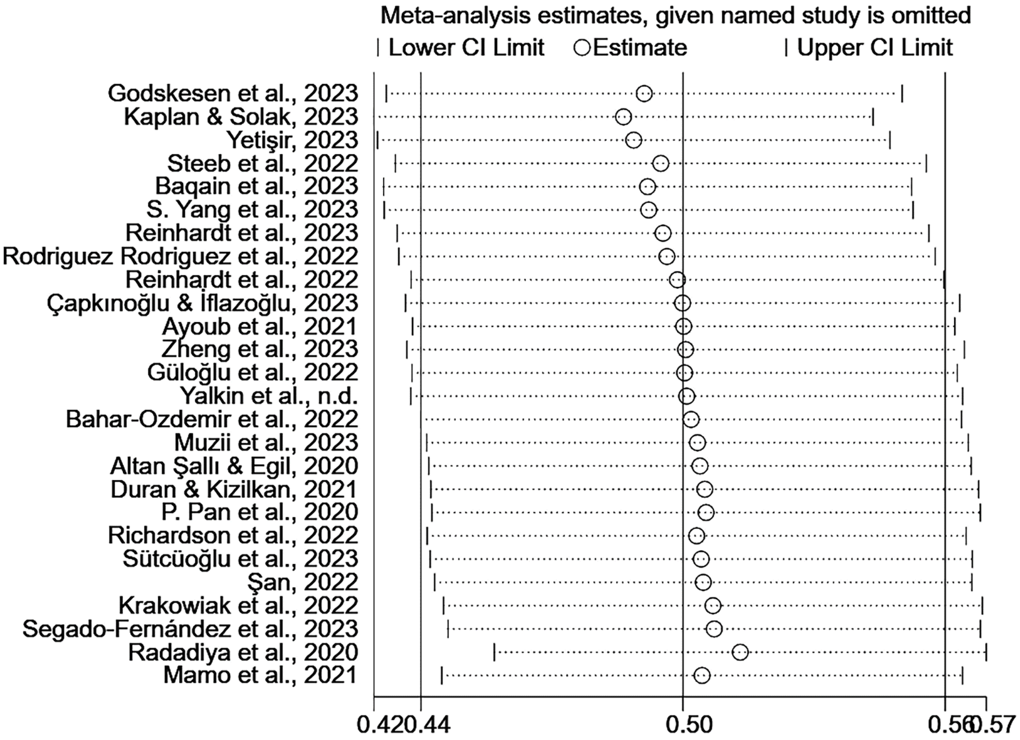

Figure S25. Leave-One-Out Sensitivity Analysis of Studies Assessing JAMA-BC Using a Random-Effects Model

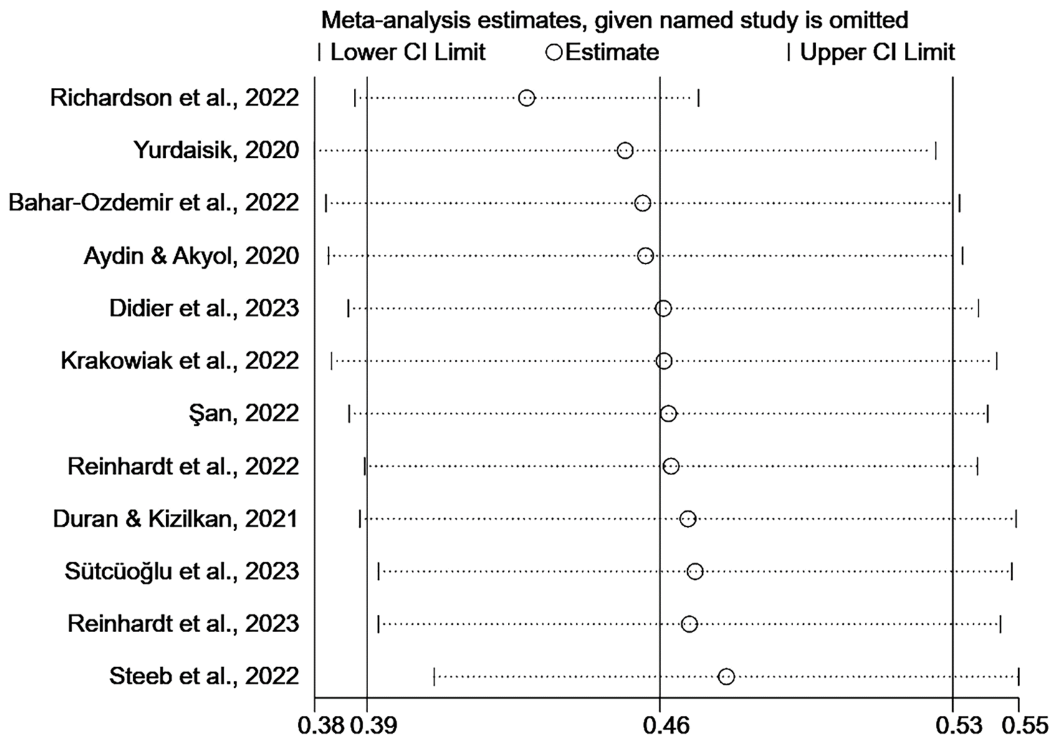

Figure S26. Leave-One-Out Sensitivity Analysis of Studies Assessing HONcode Using a Random-Effects Model

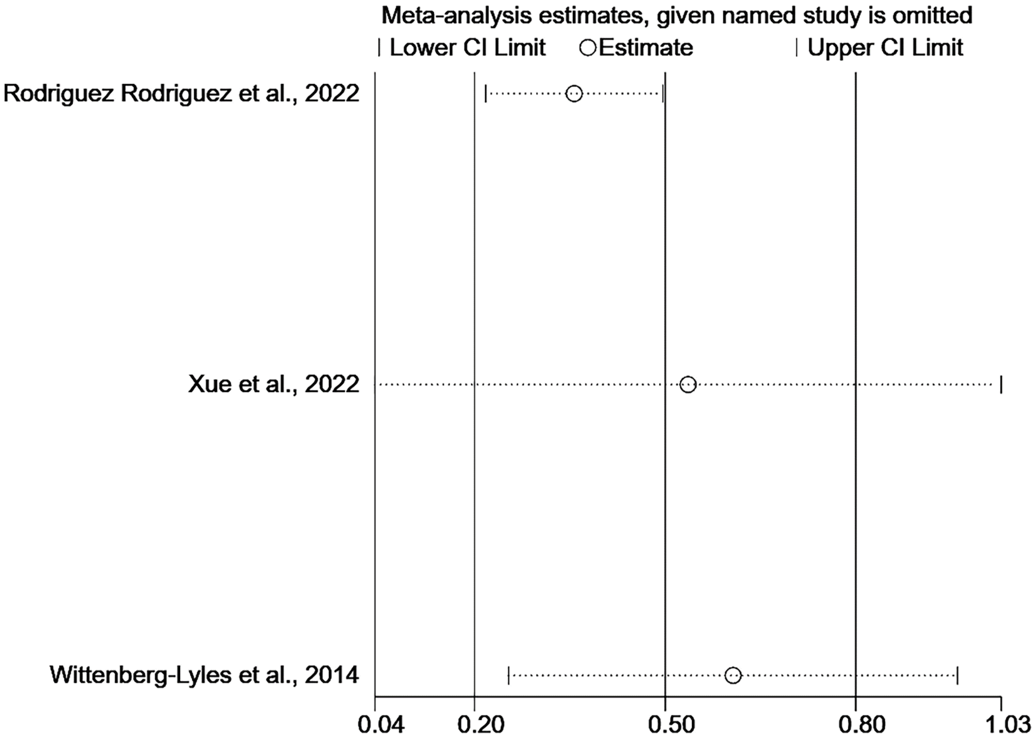

Figure S27. Leave-One-Out Sensitivity Analysis of Studies Assessing PEMAT-U Using a Random-Effects Model

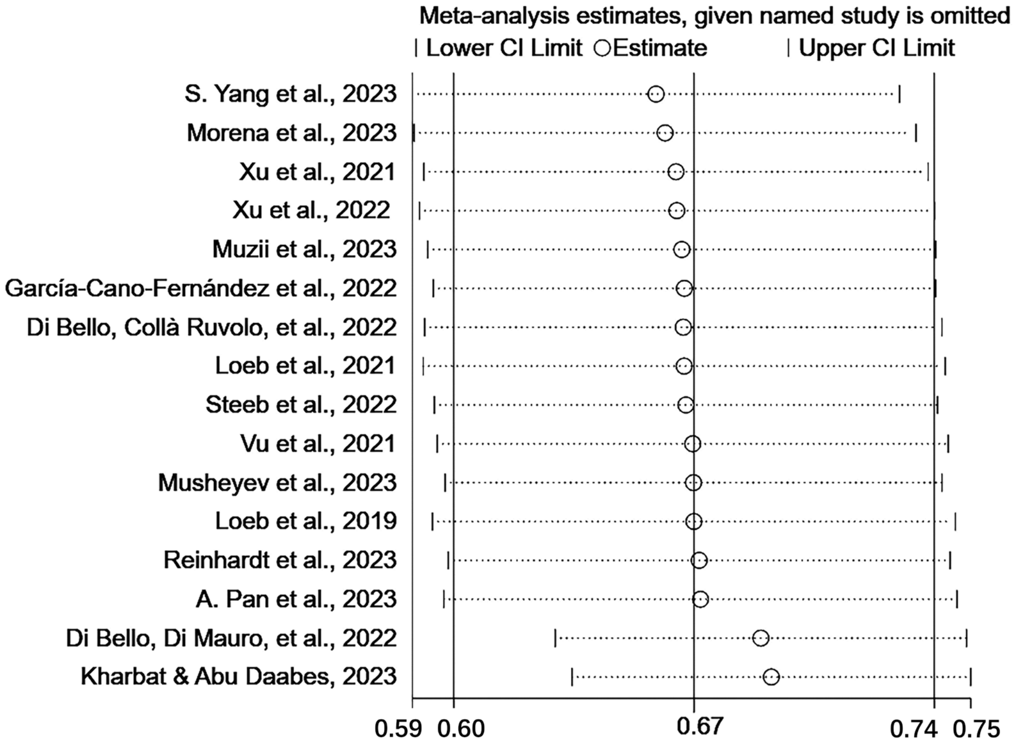

Figure S28. Leave-One-Out Sensitivity Analysis of Studies Assessing Misinformation Using a Random-Effects Model

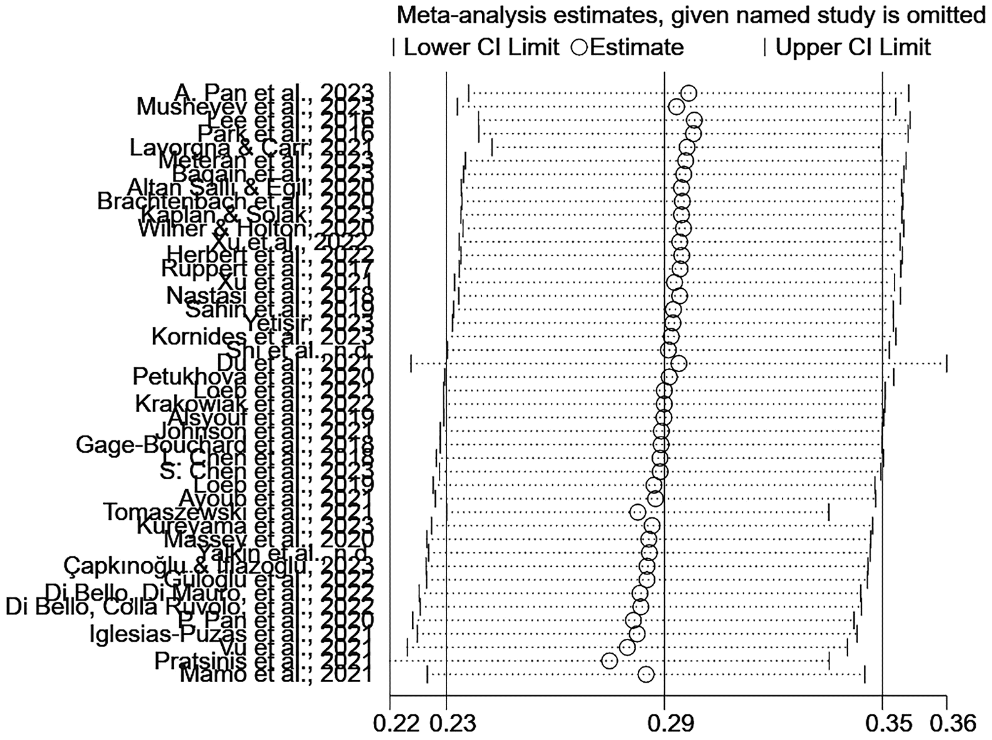

Figure S29. Leave-One-Out Sensitivity Analysis of Studies Assessing Completeness Using a Random-Effects Model

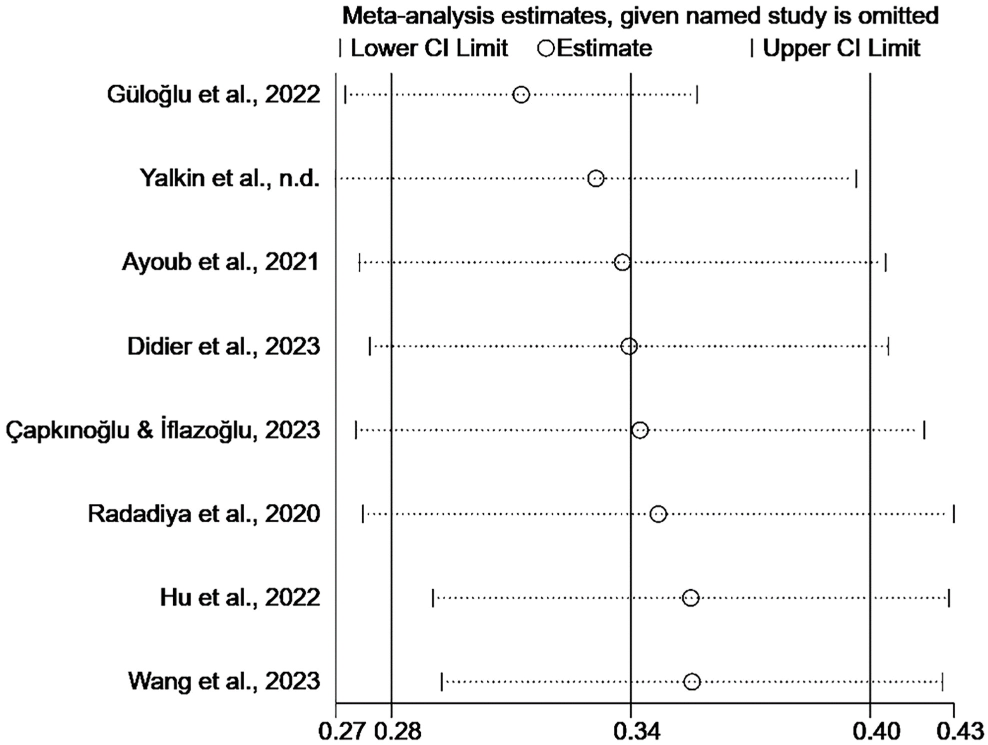

Figure S30. Leave-One-Out Sensitivity Analysis of Studies Assessing PEMAT-A Using a Random-Effects Model

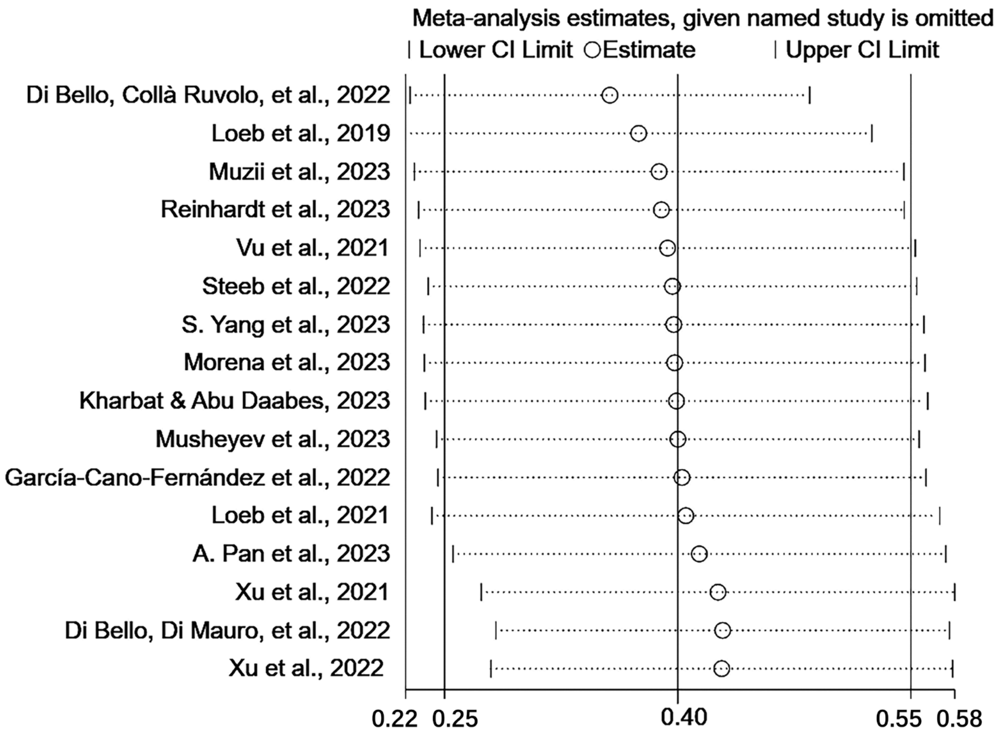

Figure S31. Leave-One-Out Sensitivity Analysis of Studies Assessing Usefulness Using a Random-Effects Model

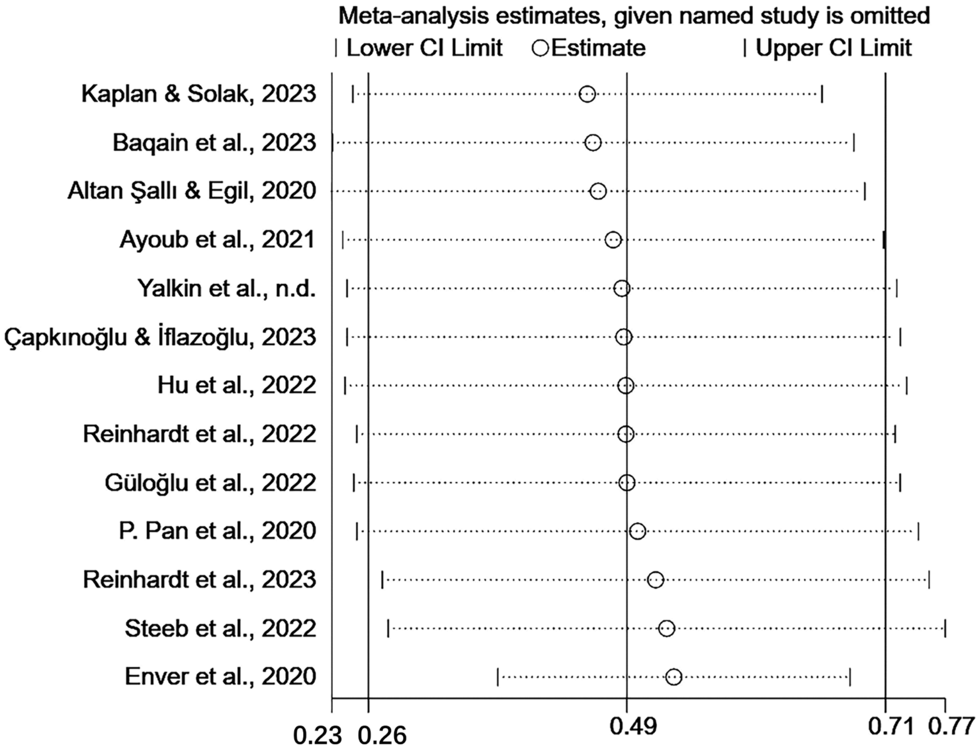

Figure S32. Leave-One-Out Sensitivity Analysis of Studies Assessing Harmfulness Using a Random-Effects Model

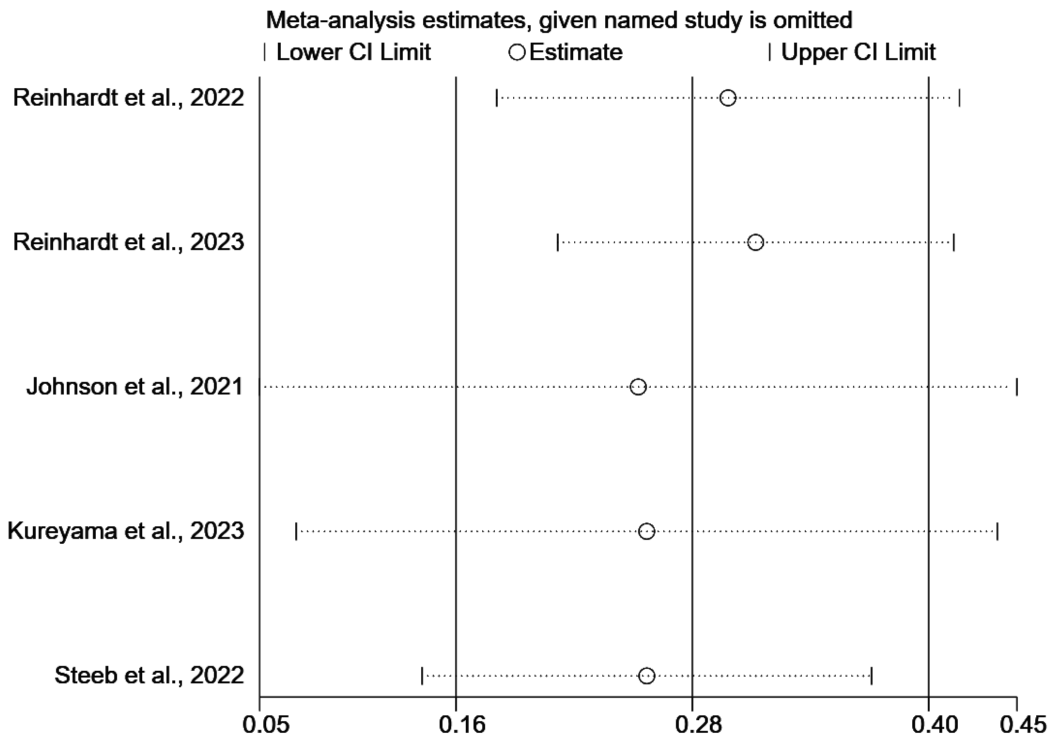

Figure S33. Leave-One-Out Sensitivity Analysis of Studies Assessing Commercial Bias Using a Random-Effects Model

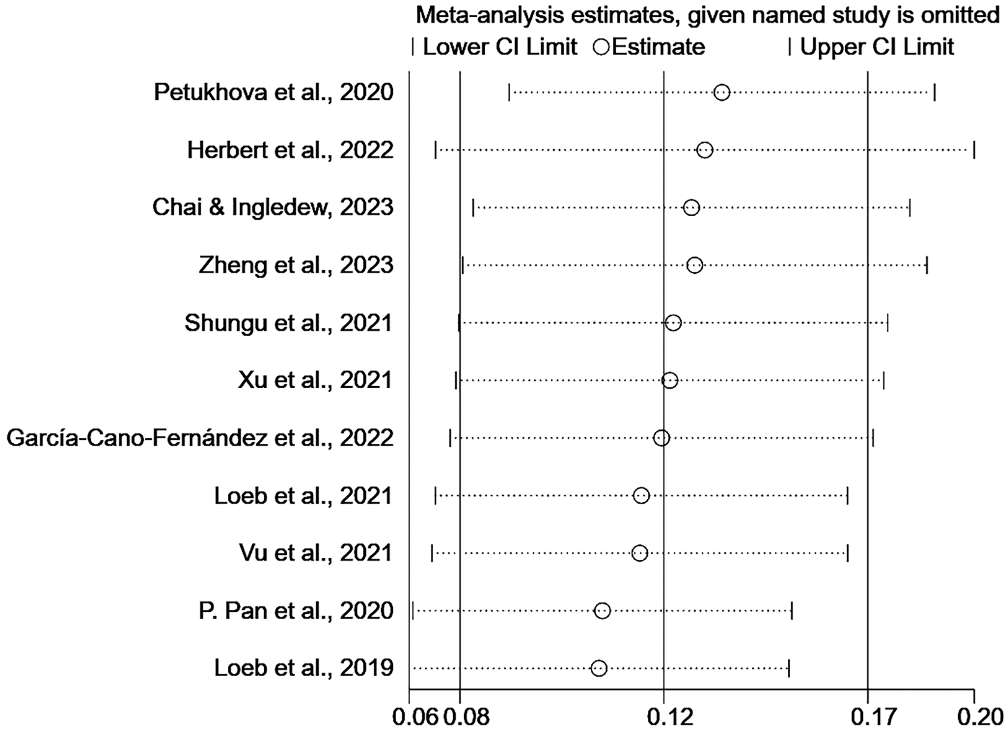

Supplement: Multimedia Appendix 2 [file jmir_v27i1e73185_app2.pdf]
